# Ancient Bacterial Class *Alphaproteobacteria* Cytochrome P450 Monooxygenases Can Be Found in Other Bacterial Species

**DOI:** 10.3390/ijms22115542

**Published:** 2021-05-24

**Authors:** Nomfundo Nzuza, Tiara Padayachee, Puleng Rosinah Syed, Justyna Dorota Kryś, Wanping Chen, Dominik Gront, David R. Nelson, Khajamohiddin Syed

**Affiliations:** 1 Department of Biochemistry and Microbiology, Faculty of Science and Agriculture, University of Zululand, KwaDlangezwa 3886, South Africa; nomfundonzuza11@gmail.com (N.N.); teez07padayachee@gmail.com (T.P.); 2 Department of Pharmaceutical Chemistry, College of Health Sciences, University of KwaZulu-Natal, Durban 4000, South Africa; prosinah@gmail.com; 3 Faculty of Chemistry, Biological and Chemical Research Center, University of Warsaw, Pasteura 1, 02-093 Warsaw, Poland; juchxd@gmail.com; 4 Department of Molecular Microbiology and Genetics, University of Göttingen, 37077 Göttingen, Germany; chenwanping1@foxmail.com; 5 Department of Microbiology, Immunology and Biochemistry, University of Tennessee Health Science Center, Memphis, TN 38163, USA

**Keywords:** cytochrome P450 monooxygenase, genome data mining, annotation, *Alphaproteobacteria*, biosynthetic gene clusters, CYP125, cholesterol oxidation

## Abstract

Cytochrome P450 monooxygenases (CYPs/P450s), heme-thiolate proteins, are well-known players in the generation of chemicals valuable to humans and as a drug target against pathogens. Understanding the evolution of P450s in a bacterial population is gaining momentum. In this study, we report comprehensive analysis of P450s in the ancient group of the bacterial class *Alphaproteobacteria*. Genome data mining and annotation of P450s in 599 alphaproteobacterial species belonging to 164 genera revealed the presence of P450s in only 241 species belonging to 82 genera that are grouped into 143 P450 families and 214 P450 subfamilies, including 77 new P450 families. Alphaproteobacterial species have the highest average number of P450s compared to *Firmicutes* species and cyanobacterial species. The lowest percentage of alphaproteobacterial species P450s (2.4%) was found to be part of secondary metabolite biosynthetic gene clusters (BGCs), compared other bacterial species, indicating that during evolution large numbers of P450s became part of BGCs in other bacterial species. Our study identified that some of the P450 families found in alphaproteobacterial species were passed to other bacterial species. This is the first study to report on the identification of CYP125 P450, cholesterol and cholest-4-en-3-one hydroxylase in alphaproteobacterial species (*Phenylobacterium zucineum*) and to predict cholesterol side-chain oxidation capability (based on homolog proteins) by *P. zucineum*.

## 1. Introduction

Cytochrome P450 monooxygenases (CYPs/P450s) are heme-thiolate proteins known to humans for more than five and a half decades [1]. P450s perform enzymatic reactions with stereo- and regio-specific oxidation and because of this capability these enzymes are subjected to various applications in all forms of biology [2,3]. Most living organisms and some non-living entities such as viruses have been found to have P450s in their genomes [4,5], indicating their important role in primary and secondary metabolism.

P450 reactions are critical in determining the drug toxicity of prodrugs and xenobiotic compounds [6]. P450s are well-known drug targets for some of the microbial pathogens [7,8]. Some of the P450s from microbes have been characterized and their application in the production of chemicals that are valuable to humans, such as secondary metabolites (compounds produced by the organisms that have no role in their growth, development or reproduction) has been explored [3,9,10,11,12,13]. Well-known examples of P450s’ involvement are antibiotic production, such as in the biosynthesis of the macrolide antibiotic erythromycin and glycopeptide antibiotics [14,15], the production of the anticancer drugs Taxol and perillyl alcohol [16,17] and the production of pharmaceutical compounds such as pravastatin [13].

P450s’ critical role in the synthesis of secondary metabolites has been thoroughly reviewed [13,18,19]. It was found that the coding sequences (genes) of enzymes involved in the synthesis of different secondary metabolites, including P450s, were part of gene clusters named biosynthetic gene clusters (BGCs) [20], where P450s were found to be critical in contributing to the diversity of the secondary metabolites [13,18,19]. In the pre-genome sequencing era, identification of such gene clusters and P450s that formed part of the clusters required tedious and laborious work. The current genome sequencing era has revolutionized the process and bioinformatics programs are available for the identification of BGCs in organisms [20]. However, to date, identification and annotation of P450s still involve manual sorting and naming as per the International P450 Nomenclature Committee rule, i.e., sequences with >40% identity are assigned to the same family as the named homolog P450 and sequences with >55% identity are assigned to the same subfamily as the named homolog P450 [21,22,23]. Proteins with <40% identity are assigned to a new P450 family. The current genome sequencing era has offered the opportunity to understand P450s’ profiles and thus evolution patterns, particularly in bacterial species where the impact of lifestyle has been found to affect the P450 profiles in an organism [24] profoundly.

The bacterial class *Alphaproteobacteria* contains the most abundant, extraordinarily diverse and ancient group of bacteria [25,26]. *Alphaproteobacteria* consist of species with a diversity of lifestyles, geographical distribution and genome size [26]. They inhabit diverse ecological niches, from water to soil, and form both extra- and intracellular associations with eukaryotes, including unicellular organisms, and multicellular plants and mammals [25,26]. Some species are in symbiotic relationships with plants such as species belonging to the genus *Agrobacterium*; species belonging to *Rickettsiaceae*, *Brucellaceae* and *Bartonellaceae* are human pathogens [27], of which a few are of ecological importance, playing a huge role in carbon, nitrogen and sulfur cycles [28]. These bacteria are found in large amounts in the upper layer of the ocean [29]. In spite of their differences, all alphaproteobacterial species are oligotrophs; therefore, they can survive in an environment with low nutrients [30]. *Alphaproteobacteria* contain species that have biotechnological significance, especially some of the species found to produce secondary metabolites, compounds that play no role in the growth or reproduction of organisms, but give the organism a selective advantage [31,32]. The biological properties of these secondary metabolites in the field of drug discovery are invaluable, and strenuous efforts to find new secondary metabolites with biotechnological potentials are being made around the world. Some of the secondary metabolites produced by alphaproteobacterial species and their biological function are presented in Table 1.

It is a well-known fact that P450s are involved in the production of secondary metabolites per se; they contribute to the diversity of secondary metabolites produced by an organism [13,18,19]. Recent genome data mining, annotation of P450s and analysis of P450s’ association with secondary metabolism in some bacterial species such as mycobacterial species [39], *Streptomyces* species [40], cyanobacterial species [41] and *Firmicutes* species [24] revealed that quite a large number of P450s are involved in the production of secondary metabolites. Apart from these bacterial species, none of the other bacterial species’ P450 repertoire has been analyzed. In addition to this, to date, the P450 repertoire of alphaproteobacterial species and their association with secondary metabolism has not been reported despite the fact that P450s, in general, are involved in the production of secondary metabolites and these species do produce secondary metabolites. Thus, in this study, we address these research gaps by performing genome-wide data mining, annotation and phylogenetic analysis of P450s and identification of P450s involved in secondary metabolism in the bacterial class *Alphaproteobacteria*.

## 2. Results and Discussion

### 2.1. Only 40% of Alphaproteobacterial Species Have P450s

**Figure 1:**
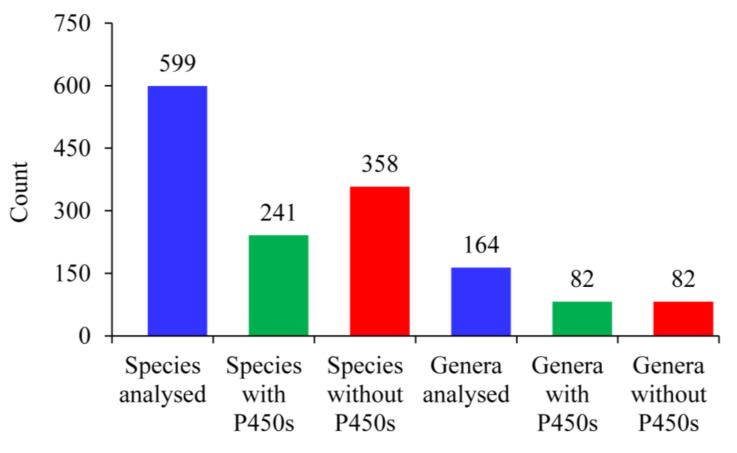
Comparative analysis of P450 statistics in the bacterial class *Alphaproteobacteria*. Detailed information is presented in Table S1.

Genome data mining and annotation of P450s in 599 alphaproteobacterial species belonging to 164 genera (Table S1) revealed the presence of P450s in only 241 species belonging to 82 genera (Figure 1). This indicates that only 40% of alphaproteobacterial species and 50% of *Alphaproteobacteria* genera have P450s (Figure 1). All the species analyzed in the study belonging to genera such as *Agrobacterium*, *Rhodopseudomonas*, *Hyphomicrobium*, *Ketogulonicigenium*, *Filomicrobium*, *Phenylobacterium, Roseobacter, Gluconacetobacter* and *Nitrobacter*, and most of the species belonging to genera such as *Sinorhizobium, Rhizobium*, *Bradyrhizobium* and *Sphingobium*, have P450s in their genomes (Table S1). A significant number of species belonging to genera such as *Rickettsia*, *Bartonella*, *Ehrlichia*, *Wolbachia* and *Anaplasma* were analyzed in this study; no P450s were found in these species, suggesting that species in these genera probably do not have P450s (Table S1). Detailed information on genera, species and P450 information is presented in Table S1. In total, 874 P450s apart from 52 short-P450s were identified in 241 alphaproteobacterial species (Table S2 and Supplementary Dataset 1). On average, four P450s were found in 241 alphaproteobacterial species; 65 species had a single P450 in their genome (Table S1). Of these 65 species, 30 were from the genus *Brucella* alone (Table S1). Comparative analysis of P450s in alphaproteobacterial species revealed that *Bradyrhizobium oligotrophicum* has the highest number of P450s (17 P450s) in its genome, followed by 16 P450s in the species *Bradyrhizobium japonicum* E109, *Novosphingobium aromaticivorans* and *Sphingomonas wittichii* and 15 P450s in *Bradyrhizobium diazoefficiens* USDA 110 (Table S2). Comparative analysis with other bacterial species revealed that alphaproteobacterial species have a higher average number of P450s compared to *Firmicutes* species [24] and cyanobacterial species [41], but a lower number compared to *Streptomyces* species [39,40] and mycobacterial species [42] (Table 2). A list of P450s and their sequences along with short-P450s is presented in Supplementary Dataset 1.

### 2.2. Alphaproteobacterial Species Have the Highest Number of P450 Families Next to Streptomyces

**Figure 2:**
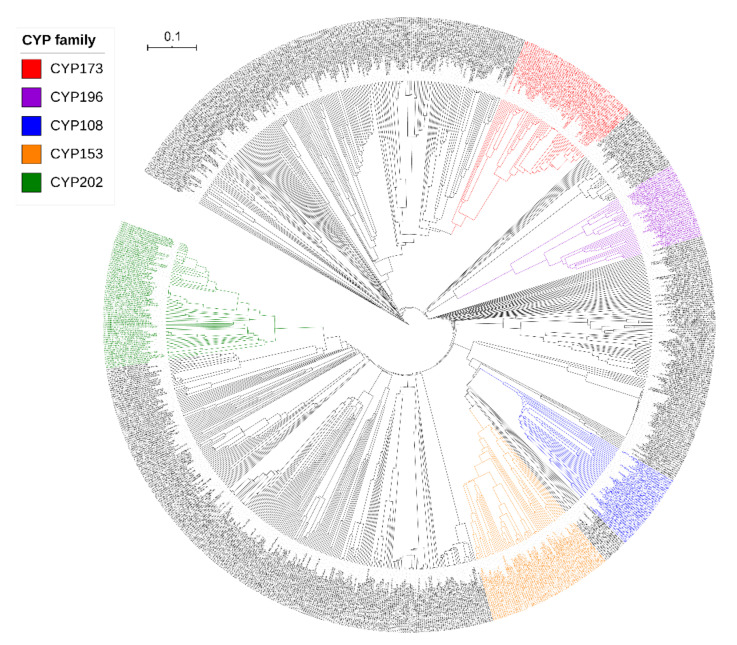
Phylogenetic analysis of alphaproteobacterial P450s. P450 families that are expanded in these species were highlighted in different colors and indicated in the figure. Alphaproteobacterial P450 protein sequences used to construct the phylogenetic tree are presented in Supplementary Dataset 1.

Following the International P450 Nomenclature Committee rules, 874 alphaproteobacterial species’ P450s were grouped into 143 P450 families and 214 P450 subfamilies (grouped in the same family when they share >40% and the same subfamily when they share >55%; with less than 40% identity species are assigned to a new P450 family) [21,22], based on the phylogenetic analysis of P450s (Figure 2) [22,44,45]. A list of P450 families and subfamilies, their count and percentage contribution to the total number of P450s is presented in Table 3.

**Figure 3:**
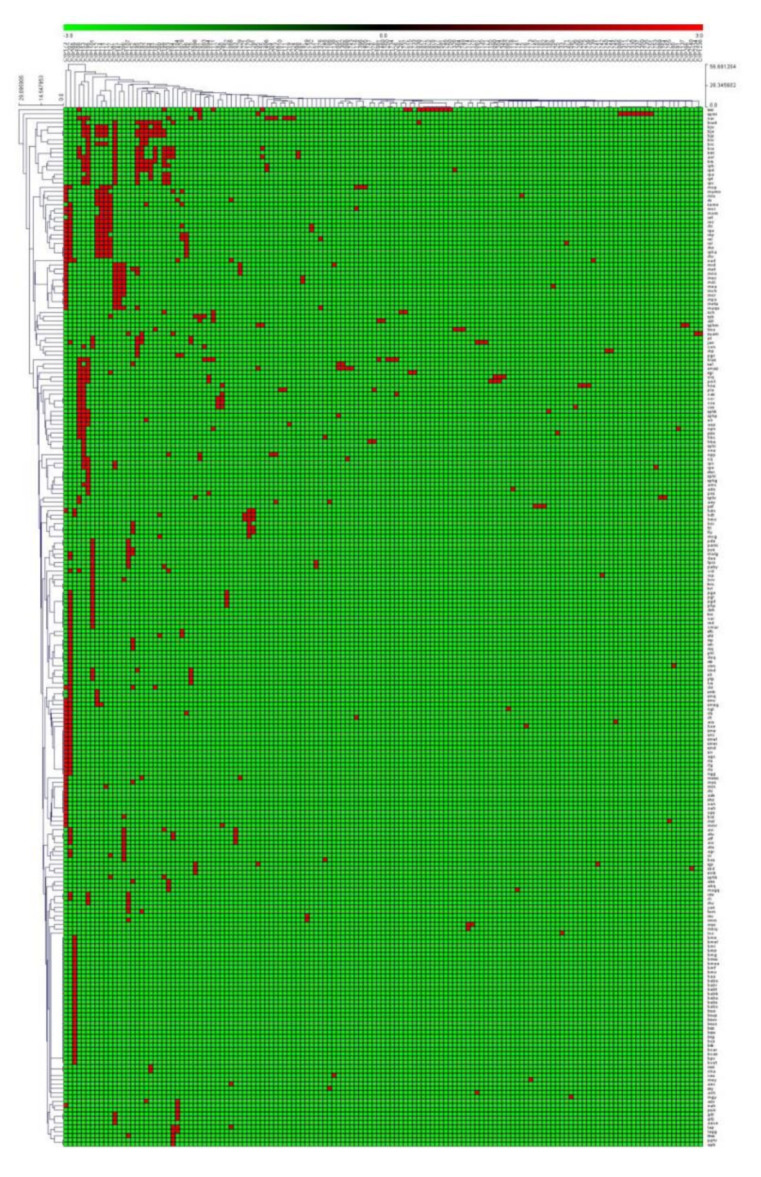
Heat map figure representing the presence or absence of cytochrome P450 families in 599 alphaproteobacterial species. The data have been represented as −3 for family absence (green) and 3 for family presence (red). One hundred and forty-three alphaproteobacterial species form the vertical axis and 214 P450 families form the horizontal axis. The respective data used in the generation of this figure are presented in Supplementary Dataset 2.

P450 family and subfamily-level comparative analysis with other bacterial species revealed that alphaproteobacterial species have more P450 families and subfamilies compared to *Firmicutes* species, mycobacterial species and cyanobacterial species but fewer than *Streptomyces* species (Table 2). The highest diversity of P450 families and subfamilies observed in alphaproteobacterial species is possibly due to their diverse lifestyle that led to the generation of diverse P450 families and subfamilies, as the lifestyle of an organism is known to have an impact on the P450 repertoire in the genomes [24]. Thus, for this reason, 77 new P450 families were observed in these species, contributing to the P450 family diversity (Table S3). In aligning with high P450 family and subfamily diversity, alphaproteobacterial species also showed the highest P450 diversity percentage compared to *Firmicutes* species and *Streptomyces* species, the same as mycobacterial species but lower than cyanobacterial species (Table 2). A point to be noted is that the number of cyanobacterial species analyzed is almost half of alphaproteobacterial species and thus cyanobacterial species have a slightly higher P450 diversity percentage (Table 2). P450 family-level analysis revealed that some P450 families are expanded in alphaproteobacterial species (Table 3). Among the P450 families, the CYP202 family has the highest number of P450s (70 P450s), followed by CYP153 and CYP173 (each 60 P450s), CYP108 and CYP196 (each 37 P450s) (Table 3). Sixty-five P450 families have a single member and 29 P450 families have two members, indicating the diversity of P450 families in alphaproteobacterial species (Table 3). Analysis of P450 families and subfamilies in alphaproteobacterial species revealed that the P450 family with the highest number of subfamilies was CYP108 (nine subfamilies) followed by CYP101, CYP152, CYP199, CYP173 (each with six subfamilies) and CYP206 (fivesubfamilies). Interestingly, some of the subfamilies are expanded in the dominant P450 families such as CYP202, CYP173, CYP153, CYP196, CYP289, CYP201, CYP1101, CYP112, CYP114 and CYP117, where subfamily “A” is dominant (Table 3). A detailed analysis of the subfamilies and their member count is presented in Table 3. Heat map analysis of the presence and absence of P450 families in different alphaproteobacterial species revealed that none of the P450 family is conserved in these species (Figure 3). Non-conservation of P450 families in bacterial species is not common; it was also observed in cyanobacterial species [41]. However, the co-presence of quite a number of P450 families was found in a large number of species, such as CYP173 and CYP202 in 32 species, CYP196 and CYP201 in 16 species, CYP195 and CYP199 in 10 species, CYP195 and CYP196 in 11 species, CY173, CYP117, CYP127, CYP112 and CYP114 in 10 species and CYP173, CYP147, CYP201 and CYP206 in 9 species (Figure 3). A point to be noted is that the co-presence of some P450 families was also observed in other bacterial species [24]. When compared to other bacterial species, the CYP202 family is dominant in alphaproteobacterial species, while CYP110 is dominant in cyanobacterial species, CYP125 in mycobacterial species, and CYP107 in both the *Firmicutes* species and *Streptomyces* species (Table 2).

### 2.3. Only a Few P450s Are Involved in Secondary Metabolism in Alphaproteobacterial Species

**Figure 4:**
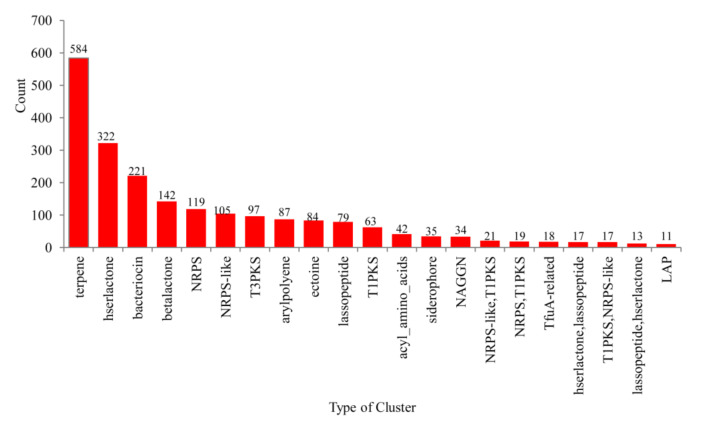
Comparative analysis of types of secondary metabolite BGCs in alphaproteobacterial species. BGCs that are populated in alphaproteobacterial species were presented in the figure. The number at the top of each bar represents the total number of clusters. Detailed information is presented in Supplementary Dataset 3.

Analysis of metabolite BGCs revealed that 504 species of 599 alphaproteobacterial species have secondary metabolite BGCs in their genomes (Supplementary Dataset 3). In total, 2262 secondary metabolite BGCs belonging to 93 types were found in 504 species (Supplementary Dataset 3). Among the 93 cluster types, terpene was the dominant cluster (270 clusters), followed by Homoserine lactone (hserlactone) (180 clusters), bacteriocin (118 clusters), betalactone (82 clusters) and nonribosomal peptides (NRPS) (79 clusters) in these species (Figure 4). Most similar known cluster analysis revealed that of 2262 BGCs, 642 BGCs showed similarity to 132 known clusters and 96 of these clusters showed 100% identity to known clusters, indicating their involvement in the specific secondary metabolite (Supplementary Dataset 3).

Analysis of P450s that are part of BGCs revealed that only 2.4% of alphaproteobacterial species P450s are involved in the production of secondary metabolites (Table 4). The percentage of P450s involved in the production of secondary metabolites in alphaproteobacterial species was found to be lowest compared to other bacterial species such as *Cyanobacteria* (8%), *Firmicutes* species (18%), mycobacterial species (11%) and *Streptomyces* species (23%) (Table 2). Twenty-one P450s from 19 alphaproteobacterial species were found to be part of BGCs (Table 4). Of the 16 P450 families that are part of BGCs, CYP206 is the dominant family (5 P450s-24%), followed by CYP1101 (2 P450s-10%) and the remaining 14 P450 families (CYP195, CYP1101, CYP2334, CYP199, CYP173, CYP153, CYP152, CYP1302, CYP127, CYP1246, CYP1138, CYP1104, CYP108, CYP107, CYP1326) have a single P450 (Table 4). Dominant P450 families such as CYP173, CYP153 and CYP108 have only one representative as part of BGCs, indicating no correlation between the dominant P450 family vs BGCs (Table 4). A point to be noted is that three P450s, CYP1326A2, CYP2334A1 and CYP195A21 from *Sulfitobacter* sp. AM1-D1, were found to be part of BGCs (Table 4). Most similar known cluster analysis revealed that CYP1101A27 from *Celeribacter indicus* was certainly involved in the production of ectoine, as the percentage identity with the most similar known cluster was 100% (Table 4).

### 2.4. Alphaproteobacterial P450 Families Can Be Found in Other Bacterial Species

**Figure 5:**
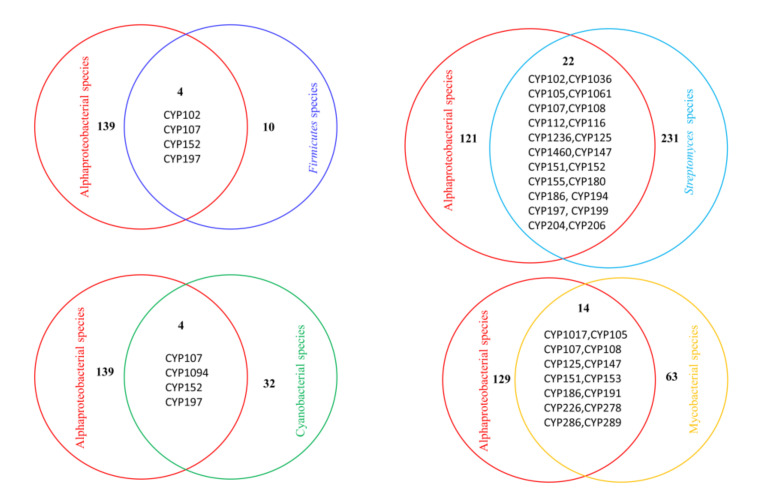
Comparative analysis of P450 families among different bacterial species. The number of P450 families indicated with a number (bold case) and P450 families that are common between alphaproteobacterial species and other bacterial species were listed in the figure.

It is a well-known fact that bacterial species in *Alphaproteobacteria* are regarded as an ancient group of bacteria [25,26]. A comparison of P450 families with other bacterial species will provide important P450 family evolutionary distribution patterns. To understand this aspect, we performed comprehensive comparative analysis of P450 families from different bacterial species (Figure 5). As shown in Figure 5, four P450 families were commonly found among alphaproteobacterial species, *Firmicutes* species and cyanobacterial species. Quite a large number of P450 families were found to be common among alphaproteobacterial species and *Streptomyces* species (22 P450 families), and mycobacterial species (14 P450 families) (Figure 5). Only the CYP107 family was found to be conserved among all bacterial species (Figure 5). CYP152 and CYP197 were commonly found in alphaproteobacterial species, *Firmicutes* species and *Streptomyces* species. Neither of these families is present in mycobacterial species. This indicates that the P450 families that are commonly found among alphaproteobacterial species and other bacterial species are indeed passed from alphaproteobacterial species and retained by other bacterial species throughout the speciation, suggesting the important role of these P450 families. One interesting observation is that the CYP125 P450 family, a cholesterol and cholest-4-en-3-one hydroxylase [47,48] and potential drug target against tuberculosis-causing bacteria *Mycobacterium tuberculosis* H37Rv [49], is commonly found in *Alphaproteobacteria* and mycobacterial species, and *Streptomyces* species (Figure 5), indicating that a cholesterol hydroxylation capability already existed in ancient bacteria as described elsewhere [50] and that these bacterial species retained this ability to survive better in the host environment, as mentioned elsewhere [39].

A point to be noted is that only one alphaproteobacterial species, namely *Phenylobacterium zucineum,* was found to have CYP125 P450 among 599 species used in the study, indicating two scenarios, i.e., loss of this P450 in other alphaproteobacterial species or gain by *P. zucineum*. This phenomenon needs further investigation. However, the presence of CYP125 P450 indicates cholesterol side-chain degradation and its subsequent utilization as carbon source *via* the beta-oxidation pathway [51] in *P. zucineum*. In order to identify the cholesterol side-chain oxidizing enzymes in *P. zucineum*, if any, we used *M. tuberculosis* H37Rv cholesterol side-chain oxidizing enzymes as listed in the literature [51]. Based on the homology percentage and annotation of enzymes at KEGG, we identify all possible cholesterol side-chain oxidizing enzymes (31 enzymes) in *P. zucineum*, indicating that this alphaproteobacterial species is indeed capable of oxidizing the cholesterol side-chain, where the CYP125 reaction will be critical in removing the side-chain from cholesterol (Table 5). As shown in Table 5, two homologs (one named *fadD*) were found for fadD36 (Rv1193) acyl-CoA synthetase. Our prediction on cholesterol side-chain degradation by *P. zucineum* is solely based on homolog proteins’ functions, including CYP125, and further validation by experimentation is required.

### 2.5. Functional Analysis of Alphaproteobacterial P450s

A literature survey on functional analysis of alphaproteobacterial P450s revealed that several P450s are functionally characterized from these species and are involved in oxidation of xenobiotic compounds (Table 6). These study results revealed that 21 P450s were found to be involved in synthesis, so secondary metabolites were CYP1101A27 from *C. indicus,* involved in the production of ectoine (Table 4). However, the physiological functional relevance of P450s in these species needs to be investigated.

## 3. Materials and Methods

### 3.1. Species and Database

In this study, 599 alphaproteobacterial species’ genomes that are available for public use at Kyoto Encyclopedia of Genes and Genomes (KEGG) database [52] were used (Table S1). Detailed information on species, species codes, genera and GenBank accession codes is presented in Table S1.

### 3.2. Genome Data Mining and Annotation of P450s

P450 mining in alphaproteobacterial species was carried out using the methods recently described by our laboratory [24,41]. Briefly, the complete proteome of alphaproteobacterial species was downloaded from KEGG and subjected to the NCBI Batch Web CD-Search Tool [68]. Proteins that belong to the P450 superfamily were selected and annotated, as per the International P450 Nomenclature Committee rule, i.e., proteins with >40% identity are grouped under the same family and proteins with >55% identity are grouped under the same subfamily [21,22,44]. Proteins with <40% identity with named P450s are assigned to a new P450 family.

### 3.3. Phylogenetic Analysis of P450s

Phylogenetic analysis of P450s was carried out following the procedure described recently by our laboratory [24,41]. The phylogenetic tree of P450s was constructed using alphaproteobacterial species P450 protein sequences. Firstly, the MAFFT v6.864 [69] was used to align the protein sequences that are part of the Trex web server [70]. The alignments were then be subjected to interpret the best tree by the Trex web server [70]. Lastly, a web-based tool, iTol, was used to create, visualize and color the tree [71].

### 3.4. Generation of P450 Profile Heat Maps

P450 profile heat maps were generated following the procedure described recently by our laboratory [24,41]. The heat map was generated using the P450 family data to show the presence or absence of P450s in alphaproteobacterial species. The data were represented as (−3) for family absence (green) and (3) for family presence (red). A tab-delimited file was imported into Mev (Multi-experiment viewer) [72]. Hierarchical clustering using a Euclidean distance metric was used to cluster the data. alphaproteobacterial species with P450s in their genome formed the vertical axis and P450 families formed the horizontal axis.

### 3.5. Secondary Metabolite BGC Analysis and Identification of P450s That Are Part of BGCs

Secondary metabolite BGC analysis and identification of P450s that are part of BGCs in alphaproteobacterial species was carried out following the procedure described recently by our laboratory [24,41]. Briefly, alphaproteobacterial species’ individual genome ID (Table S1) was submitted to anti-SMASH [46] for identification of secondary metabolite BGCs. Results were downloaded both in the form of Excel spreadsheets representing species-wise cluster information and gene cluster sequences in a Word file. P450s that are part of a specific gene cluster were identified by manually going through the BGCs sequence. Standard gene cluster abbreviation terminology present at the anti-SMASH database [46] was sustained in this study.

### 3.6. Identification of Cholesterol Side-Chain Oxidizing Genes/Proteins

Thirty-one cholesterol side-chain oxidizing genes/proteins from *M. tuberculosis* H37Rv [51] were used in the study to identify homologs in *P. zucineum*. Reference proteins were blasted individually against the *P. zucineum* genome and based on the percentage identity, homology, coverage and annotation at KEGG, the homolog proteins were identified in *P. zucineum*. A point to be noted is that *M. tuberculosis* and *P. zucineum* are distantly related and thus the lowest percentage identity between these two organisms’ proteins is expected, as previously observed [50].

### 3.7. Data Analysis

All calculations were carried out following the procedure described previously [24,41,73]. The average number of P450s was calculated using the formula: Average number of P450s = Number of P450s/Number of species. The P450 diversity percentage was calculated using the formula: 100 × Total number of P450 families/(Total number of P450s × Number of species with P450s). The percentage of P450s that formed part of BGCs was calculated using the formula: Percentage of P450s part of BGCs = 100 × Number of P450s part of BGCs/Total number of P450s present in species.

## 4. Conclusions

Cytochrome P450 monooxygenases (CYPs/P450s) have been well-known proteins for the last six decades. The stereo- and regio-specific oxidation of a variety of compounds by these enzymes led to their applications in quite large areas of biological research. Understanding the evolution of P450s in the bacterial population is now gaining momentum owing to the availability of a large number of bacterial genomes. This study is the first of its kind on the analysis of P450s in an ancient group of bacteria belonging to the class *Alphaproteobacteria*. Comparative analysis of P450s between different bacterial species revealed that during speciation a large number of P450s became part of secondary metabolite gene clusters (as observed in *Streptomyces* species and mycobacterial species) and some P450s were passed all the way from *Alphaproteobacteria* to other bacterial species. This study also reports the first identification of CYP125 P450 in alphaproteobacterial species and predicts that *P. zucineum* is capable of utilizing the cholesterol side-chain as carbon source. Future study should include thorough profiling of cholesterol-degrading genes/proteins and experimental validation of cholesterol degrading ability. Furthermore, comparative analysis of alphaproteobacterial species and more bacterial species should be carried out to deduce the evolution pattern of P450 families and to identify the loss/gain of new P450 families compared to *Alphaproteobacteria* with respect to habitat or geographical distribution. The results of this study will serve as reference for future genome data mining and annotation of P450s in species of *Alphaproteobacteria*.

## Figures and Tables

**Table 1:** Some of the secondary metabolites produced by alphaproteobacterial species and their biological functions.

Secondary Metabolite	Species (Source)	Biological Function	Reference
Didemnin B	*Tistrella mobilis* YIT 12409	Anti-viral and anti-tumor properties	[33]
Thalassospiramide	*Thalassospira* sp. CNJ-328	Immunosuppression	[34]
Tropodithietic acid	*Phaeobacter gallaeciensis* and *P. porticola*	Antibiotic, biocontrol and anti-cancer	[31]
Phytohormone	*Methylobacterium* sp. NC4	Improve plant growth	[35]
6-gingerol and curcumin	*Bradyrhizobium japonicum* CPAC 15 and *Bradyrhizobium diazoefficiens* CPAC 7	Antitumor activity	[36]
Gibberellins, salicylic acid, indole-3-acetic acid, zeatin, and abscisic acid	*Sphingomonas* species	Promote plant germination and growth	[37]
Cyclodipeptides	*Sulfitobacter* species	Bioactive agents	[38]

**Table 2:** Comparative analysis of main characteristics of P450s in different bacterial species.

Category	Alphaproteobacterial Species	*Firmicutes* Species	*Streptomyces* Species	Mycobacterial Species	Cyanobacterial Species
Total no of species analyzed	599	972	203	60	114
No of species that have P450s	241	229	203	60	114
No of P450s	874	712	5460	1784	341
No of families	143	14	253	77	36
No of subfamilies	214	53	698	132	79
Dominant P450 family	CYP202	CYP107	CYP107	CYP125	CYP110
Average no. of P450s	4	1	27	30	3
P450 diversity percentage	0.07	0.008	0.02	0.07	0.09
No of P450s part of BGCs	21	126	1231	204	27
Percentage of P450s part of BGCs	2.4	18	23	11	8
Reference(s)	This work	[24,43]	[39,40]	[39,42]	[41]

**Table 3:** Comparative analysis of P450 families and subfamilies in alphaproteobacterial species.

P450 Family	P450 Count	Percentage Count	Subfamily	P450 Count
CYP1010	1	0.11%	A	1
CYP1017	1	0.11%	A	1
CYP101	10	1.14%	B	1
			C	3
			D	2
			E	2
			Q	1
			R	1
CYP102	12	1.37%	A	10
			K	1
			AC	1
CYP1036	2	0.23%	B	1
			C	1
CYP103	4	0.46%	A	4
CYP104	13	1.49%	A	13
CYP105	4	0.46%	BF	3
			CX	1
CYP1061	2	0.23%	B	2
CYP1068	4	0.46%	A	4
CYP1075	1	0.11%	A	1
CYP1076	1	0.11%	A	1
CYP1077	1	0.11%	A	1
CYP1078	8	0.92%	A	7
			C	1
CYP107	10	1.14%	AN	8
			_	1
			JE	1
CYP1081	2	0.23%	B	2
CYP1082	1	0.11%	A	1
CYP1083	5	0.57%	A	3
			C	2
CYP1086	1	0.11%	B	1
CYP1088	2	0.23%	B	2
CYP1089	1	0.11%	A	1
CYP108	37	4.23%	A	2
			D	6
			G	18
			L	1
			P	2
			U	1
			V	1
			W	1
			X	5
CYP1090	2	0.23%	C	2
CYP1091	3	0.34%	A	3
CYP1094	4	0.46%	A	3
			B	1
CYP1098	6	0.69%	A	6
CYP1101	29	3.32%	A	29
CYP1104	10	1.14%	A	4
			C	3
			E	3
CYP1107	16	1.83%	A	12
			B	3
			C	1
CYP1111	7	0.80%	A	5
			B	1
			C	1
CYP1116	3	0.34%	B	3
CYP1118	1	0.11%	B	1
CYP111	2	0.23%	A	1
			B	1
CYP112	23	2.63%	A	23
CYP1135	1	0.11%	A	1
CYP1137	1	0.11%	A	1
CYP1138	2	0.23%	B	2
CYP1141	1	0.11%	A	1
CYP1145	1	0.11%	A	1
CYP114	22	2.52%	A	22
CYP1155	1	0.11%	B	1
CYP115	1	0.11%	A	1
CYP116	1	0.11%	B	1
CYP1170	7	0.80%	A	7
CYP1171	1	0.11%	A	1
CYP1173	1	0.11%	A	1
CYP1174	2	0.23%	A	2
CYP1175	1	0.11%	A	1
CYP117	22	2.52%	A	22
CYP1181	1	0.11%	A	1
CYP1182	1	0.11%	A	1
CYP1186	2	0.23%	A	2
CYP1187	1	0.11%	A	1
CYP1204	2	0.23%	A	2
CYP1206	1	0.11%	B	1
CYP1221	2	0.23%	B	2
CYP1229	4	0.46%	A	1
			B	2
			C	1
CYP1246	2	0.23%	A	2
CYP1250	1	0.11%	A	1
CYP1258	1	0.11%	A	1
CYP125	1	0.11%	P	1
CYP1275	1	0.11%	B	1
CYP127	19	2.17%	A	18
			C	1
CYP1281	1	0.11%	A	1
CYP1291	5	0.57%	B	3
			C	2
CYP1302	2	0.23%	A	2
CYP1311	2	0.23%	A	2
CYP1312	1	0.11%	A	1
CYP1326	2	0.23%	B	2
CYP1330	1	0.11%	A	1
CYP1337	1	0.11%	A	1
CYP133	1	0.11%	F	1
CYP1349	1	0.11%	A	1
CYP1350	1	0.11%	A	1
CYP1371	1	0.11%	B	1
CYP1376	2	0.23%	B	1
			C	1
CYP1384	1	0.11%	A	1
CYP1396	1	0.11%	A	1
CYP1405	1	0.11%	B	1
CYP1406	3	0.34%	B	2
			C	1
CYP1460	1	0.11%	C	1
CYP147	16	1.83%	D	16
CYP1515	2	0.23%	A	2
CYP151	2	0.23%	C	2
CYP152	13	1.49%	B	4
			C	3
			E	2
			P	1
			AA	1
			AB	2
CYP153	60	6.86%	A	44
			C	2
			D	14
CYP155	4	0.46%	J	3
			K	1
CYP1591	1	0.11%	A	1
CYP1597	1	0.11%	A	1
CYP1732	3	0.34%	A	3
CYP1733	2	0.23%	A	1
			B	1
CYP1734	1	0.11%	A	1
CYP1735	1	0.11%	A	1
CYP1736	1	0.11%	A	1
CYP1737	2	0.23%	A	2
CYP1738	1	0.11%	A	1
CYP1739	2	0.23%	A	2
CYP173	60	6.86%	A	45
			B	8
			C	3
			G	1
			H	1
			J	2
CYP1740	1	0.11%	A	1
CYP1741	1	0.11%	A	1
CYP1742	1	0.11%	A	1
CYP1743	1	0.11%	A	1
CYP1744	1	0.11%	A	1
CYP1745	1	0.11%	A	1
CYP1746	1	0.11%	A	1
CYP1747	1	0.11%	A	1
CYP1748	1	0.11%	A	1
CYP1749	2	0.23%	A	1
			B	1
CYP1750	1	0.11%	A	1
CYP1751	1	0.11%	A	1
CYP1752	4		A	4
CYP1753	1	0.11%	A	1
CYP1754	1	0.11%	A	1
CYP1755	1	0.11%	A	1
CYP180	1	0.11%	D	1
CYP186	7	0.80%	K	7
CYP191	1	0.11%	B	1
CYP192	3	0.34%	A	3
CYP193	17	1.95%	A	17
CYP194	10	1.14%	A	10
CYP195	21	2.40%	A	18
			D	1
			E	2
CYP196	37	4.23%	A	34
			B	2
			C	1
CYP197	2	0.23%	R	2
CYP199	16	1.83%	A	11
			B	1
			J	1
			K	1
			L	1
			M	1
CYP200	8	0.92%	A	5
			B	2
			C	1
CYP201	31	3.55%	A	25
			B	3
			C	3
CYP202	70	8.01%	A	46
			B	24
CYP203	13	1.49%	A	12
			B	1
CYP204	2	0.23%	A	2
CYP206	19	2.17%	A	9
			B	1
			C	3
			D	6
CYP219	2	0.23%	A	2
CYP223	3	0.34%	A	2
			E	1
CYP224	1	0.11%	A	1
CYP225	7	0.80%	A	7
CYP226	2	0.23%	C	1
			D	1
CYP278	2	0.23%	A	1
			C	1
CYP286	1	0.11%	C	1
CYP288	2	0.23%	B	2
CYP289	33	3.78%	A	33
CYP290	6	0.69%	A	5
			B	1
CYP2140	1	0.11%	A	1
CYP1236	1	0.11%	A	1
CYP2334	2	0.23%	A	2

**Table 4:** Identification of P450s that are part of the secondary metabolite BGCs in alphaproteobacterial species. Reference cluster information was obtained by performing BLAST at the anti-SMASH database [46], as indicated in the materials and methods section. The cluster type and most similar known cluster names available at the anti-SMASH database [46] were listed in the table.

Species Name	P450	Reference Cluster Information		
		Cluster Type	Most Similar Known Cluster	Similarity
Novosphingobium aromaticivorans	CYP153C1	Terpene	Astaxanthin dideoxyglycoside	75%
*Mesorhizobium japonicum* MAFF 303099	CYP127A3v1	Hserlactone	-	-
Agrobacterium fabrum	CYP206A1	Terpene	-	-
*Agrobacterium* sp. H13-3	CYP206A4	Terpene	-	-
*Agrobacterium tumefaciens*	CYP206A4	Terpene	-	-
*Agrobacterium rhizogenes*	CYP206A2	Terpene	-	-
*Rhizobium* sp. IRBG74	CYP206A3	Terpene	-	-
*Rhizobium* sp. NT-26	CYP107JE1	NAGGN	-	-
*Bradyrhizobium* sp. S23321	CYP199A26	Hserlactone, t2pks	Colabomycin	4%
*Bradyrhizobium oligotrophicum*	CYP108L2	NRPS	-	-
*Bosea vaviloviae*	CYP1101A30	T1PKS	S56-p1, NRPS	3%
*Beijerinckia indica*	CYP173J1	NRPS, T1PKS	-	-
*Pseudorhodoplanes sinuspersici*	CYP1104E2	T1PKS	Sphingan polysaccharide, saccharide	13%
*Rhodobacter sphaeroides* ATCC 17025	CYP152C2	hserlactone	Conglobatin, NRPS	10%
*Celeribacter indicus*	CYP1101A27	Ectoine	Ectoine, other	100%
*Hyphomonadaceae bacterium* UKL13-1	CYP1246A4	Terpene	-	-
*Sphingopyxis macrogoltabida* 203	CYP1302A1	NRPS	-	-
*Xanthobacter autotrophicus*	CYP1138B1	T1PKS	-	-
*Sulfitobacter* sp.AM1-D1	CYP1326A2	acyl_amino_acids	-	-
	CYP195A21	bacteriocin	-	-
	CYP2334A1	NRPS-like	-	-

**Table 5:** Identification of homolog proteins involved in cholesterol side-chain oxidation in *Phenylobacterium zucineum*. *Mycobacterium tuberculosis* H37Rv cholesterol side-chain degrading proteins from the published literature [51] were used to identify homolog proteins in *P. zucineum*. Gene and protein IDs and enzyme annotations were from KEGG [52].

Proteins Involved in Cholesterol Side-Chain Oxidation in *Mycobacterium tuberculosis* H37Rv			Homolog Proteins in *Phenylobacterium zucineum*			
Gene Name	Gene ID	Enzyme	Protein ID	% Identity	% Homology	Enzyme
*fadD36*	*Rv1193*	Acyl-CoA synthetase	PHZ_c1345 (fadD)	29	41	Long-chain acyl-CoA synthetase
			pzu:PHZ_c1155	38	52	Malonyl-CoA/methylmalonyl-CoA synthetase
*fadD19*	*Rv3515c*	Probable fatty-acid-CoA ligase	pzu:PHZ_c2065	39	57	Fatty-acid-CoA ligase
*fadD3*	*Rv3561*	Acyl-CoA synthetase (AMP forming)	pzu:PHZ_c1909	35	47	Long-chain-fatty-acid—CoA ligase
*fadD17*	*Rv3506*	Possible fatty-acid-CoA ligase	pzu:PHZ_c0597	30	46	Fatty-acyl-CoA synthase
*fadD19*	*Rv3515c*	Probable fatty-acid-CoA ligase	pzu:PHZ_c2065	39	57	Long-chain fatty acid:CoA ligase
*fadD10*	*Rv0099*	Fatty acid-CoA synthase	pzu:PHZ_c2512	29	43	Long-chain-fatty-acid--CoA ligase
*fadD9*	*Rv2590*	Fatty acid-CoA synthase	pzu:PHZ_c2123	25	38	Long-chain acyl-CoA synthetase
*fadD18*	*Rv3513c*	Possible fatty-acid-CoA ligase	pzu:PHZ_c2065	55	73	CoA-synthetase, long-chain fatty acid:CoA ligase
*fadE22*	*Rv3061c*	Acyl-CoA dehydrogenase	pzu:PHZ_c2678	40	54	Dehydrogenase family protein
*fadE23*	*Rv3140*	Acyl-CoA dehydrogenase	pzu:PHZ_c0890	33	51	Acyl-CoA dehydrogenase
*fadE24*	*Rv3139*	Acyl-CoA dehydrogenase	pzu:PHZ_c2365	32	48	Acyl-CoA dehydrogenase
*fadE25*	*Rv3274c*	Acyl-CoA dehydrogenase FADE25	pzu:PHZ_c1680	40	60	Acyl-CoA dehydrogenase
*fadE26*	*Rv3504*	Probable acyl-CoA dehydrogenase	pzu:PHZ_c2336	33	50	Acyl-CoA dehydrogenase
*fadE27*	*Rv3505*	Probable acyl-CoA dehydrogenase	pzu:PHZ_c2406	28	45	Isovaleryl CoA dehydrogenase
*fadE28*	*Rv3544c*	Probable acyl-CoA dehydrogenase	pzu:PHZ_c2498	26	43	Acyl-CoA dehydrogenase
*fadE29*	*Rv3543c*	Probable acyl-CoA dehydrogenase	pzu:PHZ_c2541	32	51	Acyl-CoA dehydrogenase
*fadE30*	*Rv3560c*	Probable acyl-CoA dehydrogenase	pzu:PHZ_c2541	41	57	Acyl-CoA dehydrogenase
*fadE31*	*Rv3562*	Probable acyl-CoA dehydrogenase	pzu:PHZ_c2678	36	53	Dehydrogenase family protein
*fadE32*	*Rv3563*	Probable acyl-CoA dehydrogenase	pzu:PHZ_c2498	32	45	Acyl-CoA dehydrogenase
*fadE33*	*Rv3564*	Probable acyl-CoA dehydrogenase	pzu:PHZ_c2679	33	48	Acyl-CoA dehydrogenase family protein
*fadE34*	*Rv3573c*	Probable acyl-CoA dehydrogenase	pzu:PHZ_c2678	38	56	Dehydrogenase family protein
*fadE5*	*Rv0244c*	Acyl-CoA dehydrogenase	pzu:PHZ_c3354	36	51	Acyl-CoA dehydrogenase
*mbtN (fadE14)*	*Rv1346*	Acyl-CoA dehydrogenase	pzu:PHZ_c0388	30	48	Acyl-CoA dehydrogenase
*echA9*	*Rv1071c*	3-Hydroxyisobutyryl-CoA hydrolase	pzu:PHZ_c1679	39	57	Enoyl-CoA hydratase/isomerase family protein
*echA19*	*Rv3516*	Possible enoyl-CoA hydratase	pzu:PHZ_c2535	38	54	Enoyl-CoA hydratase/carnithine racemase
*echA20*	*Rv3550*	Possible enoyl-CoA hydratase	pzu:PHZ_c3429	33	51	Enoyl-CoA hydratase/isomerase family protein
*fadB2*	*Rv0468*	hydroxybutyryl-CoA dehydrogenase	pzu:PHZ_c3152	43	60	3-Hydroxyacyl-CoA dehydrogenase
*fadB3*	*Rv1715*	Hydroxybutyryl-CoA dehydrogenase	pzu:PHZ_c3152	35	49	3-Hydroxyacyl-CoA dehydrogenase
*fadA5*	*Rv3546*	Acetoacetyl-CoA thiolase	pzu:PHZ_c2504	40	60	Acetyl-CoA C-acetyltransferase
*hsd4A*	*Rv3502c*	17β-Hydroxysteroid dehydrogenase (17β-HSD)	pzu:PHZ_c2008	36	49	Short-chain dehydrogenase/reductase SDR
*ltp2*	*Rv3540c*	Probable ketoacyl-CoA thiolase	pzu:PHZ_c3245	36	49	Acetyl-CoA C-acetyltransferase

**Table 6:** Functional analysis of alphaproteobacterial P450s.

P450	Function	Reference
CYP101B1	α-ionone, β-damascone, phenylcyclohexane and *p*-cymene hydroxylation	[53]
CYP101C1	Ionone derivative hydroxylation	[54]
CYP101D1	Terpenoid (camphor) hydroxylase	[55,56]
CYP101D2	Camphor 5-exo hydroxylase	[57]
CYP108D1	Aromatic hydrocarbon hydroxylase	[55,58]
CYP153C1, CYP153D1	Alkane hydroxylase	[59]
CYP111A2	Oxidizes linalool to 8-hydroxylinalool	[55]
CYP111B1	β-Ionone hydroxylation	[60]
CYP112A2	Oxidation of Rapamycin	[61]
CYP127A3	Hydroxylation of testosterone	[62]
CYP195A2	Degradation of 4-fluoro-, 4-chloro- and 4-methylsalicylic acid, and 3-chloro- and 3-methylsalicylic acid	[63]
CYP199A1	Hydroxylation of 2-naphthoic acid	[64]
CYP199A2	Hydroxylation of 2-naphthoic acid, para-substituted benzoic acids and involved in the degradation of ligninolic compounds	[64,65]
CYP199A4	Catalyzes heteroatom dealkylations, sulfoxidation, and amide and cyclic hemiacetal formation	[66]
CYP200A1	Hydroxylation of testosterone	[62]
CYP201A2	Involved in the biodegradation of tributyl phosphate	[67]

## Data Availability

Not applicable.

## Supplementary Materials

The following are available online at https://www.mdpi.com/article/10.3390/ijms22115542/s1, Table S1: Information on Alphaproteobacterial species and their respective genera used in the study, Table S2: Comparative analysis of P450s and those associated with secondary metabolite biosynthetic gene clusters in the bacterial class *Alphaproteobacteria*, Table S3: List of new P450 families identified in Alphaproteobacterial species., Dataset 1: P450 sequences identified in Alphaproteobacterial species; Dataset 2: P450 heat-map data; Dataset 3: Secondary metabolite biosynthetic gene clusters (BGCs) analysis and P450s associated with BGCs in Alphaproteobacterial species.
