# Supplementary material for: Ancient Bacterial Class Alphaproteobacteria Cytochrome P450 Monooxygenases Can Be Found in Other Bacterial Species
Source: Int J Mol Sci. 2021 May 24;22(11):5542. doi: 10.3390/ijms22115542 (PMC8197338; doi:10.3390/ijms22115542)
Supplement: Supplementary file 1 [file ijms-22-05542-s001.zip › Supplementary Dataset 1.pdf]

Type of the Paper (Article)

Ancient bacterial class *Alphaproteobacteria* cytochrome P450 monooxygenases can be found in other bacterial species

Nomfundo Nzuza [1], Tiara Padayachee [1], Puleng Rosinah Syed [2], Justyna Dorota Kryś [3], Wanping Chen [4], Dominik Gront [3*], David R Nelson [5*], Khajamohiddin Syed [1*]

[1] Department of Biochemistry and Microbiology, Faculty of Science and Agriculture, University of Zululand, KwaDlangezwa 3886, South Africa; nomfundonzuza11@gmail.com (N.N.); teez07padayachee@gmail.com (T.P.); khajamohiddinsyed@gmail.com (K.S.)
[2] Department of Pharmaceutical Chemistry, College of Health Sciences, University of KwaZulu-Natal, Durban 4000, South Africa; prosinah@gmail.com (P.R.S.)
[3] Faculty of Chemistry, Biological and Chemical Research Center, University of Warsaw, Pasteura 1, 02-093 Warsaw, Poland; juchxd@gmail.com (J.D.K); dgront@gmail.com (D.G)
[4] Department of Molecular Microbiology and Genetics, University of Göttingen, 37077 Göttingen, Germany; chenwanping1@foxmail.com (W.C.)
[5] Department of Microbiology, Immunology and Biochemistry, University of Tennessee Health Science Center, Memphis, TN, 38163; drnelson1@gmail.com (D.N.R.)
* Correspondence: dgront@gmail.com (D.G.), drnelson1@gmail.com (D.N.R.) and khajamohiddinsyed@gmail.com (K.S.)

Supplementary Dataset 1: P450 sequences identified in Alphaproteobacterial species.

Full-length P450s:

>CYP101B1(Saro_3533)*Novosphingobium aromaticivorans*

MEAPAHVPADRVVDIDIYMPPGLAEHGFHKAWSDLSAGNPAVVWTPRNEGHWIALGGEALQEVQSDPERFSSR
IIVLPKSVGEMHGLIPTTIDPPEHRPYRQLLNAHLNPGAIRGLSESIRQTAVDLIEGFAAQGHCNFTAQYAEQ
FPIRVFMALVGIEASEAPRIRHWAECMTRPGMDMTFDEAKAVFFDYVGPLVDARRETPGEDMISAMINADLGD
GRRLTRDEALSVVTQVLIAGLDTVVNVLGFIMRELAGNPALRADLRQRGADILPVVHELFRRFGLVSIAREVR
RDIEFHGVHLKAGDMIAIPTQVHGLDPRVNPDPLAIDPSRKRARHSTFGSGPHMCPGQELARKEVAITLEEWL
RRIPDFALGPNSDLSPVPGIVGALRRVELVWNT

>CYP101C1(Saro_2249)*Novosphingobium aromaticivorans*

MIPAHVPADRVVDFDIFNPPGVEQDYFAAWKTLLDGPGLVWSTANGGHWIAARGDVVRELWGDAERLSSQCLA
VTPGLGKVMQFIPLQQDGAEHKAFRTPVMKGLASRFVVALEPKVQAVARKLMESLRPRGSCDFVSDFAEILPL
NIFLTLIDVPLEDRPRLRQLGVQLTRPDGSMTVEQLKQAADDYLWPFIEKRMAQPGDDLFSRILSEPVGGRPW
TVDEARRMCRNLLFGGLDTVAAMIGMVALHLARHPEDQRLLRERPDLIPAAADELMRRYPTVAVSRNAVADVD
ADGVTIRKGDLVYLPSVLHNLDPASFEAPEEVRFDRGLAPIRHTTMGVGAHRCVGAGLARMEVIVFLREWLGG
MPEFALAPDKAVTMKGGNVGACTALPLVWRA

>CYP101C5(PP1Y_AT30146)*Novosphingobium* sp. PP1Y

MATVAANRPDHVPADRVVDFDIFAPPGSEEDYFAAWASVRGDHALVWTTANGGHWIAADGETTRKLWNDAENL
SNEALAVTPGLGEAMNFIPLQQDAPEHKPFRMAVMKGFANNHVVAMEPLIREVARELTAQITPHGECEFMEEY
AEVLPIHIFLTLIDVPTQDRLRLRPLGQQLTRPDGSMTVEQLRDAADDYLRPYIEERLRKPGQDLFSRILSIP
IEGREWTFDEAQRMCRNLLFGGLDTVVSMFGNIALHLARYPEDQNLLRNRPELIPQAADELMRRYPMVSVTRN
LVRDVEIDGITLRSGDLVYLNSSIHNLDTACFDNPHKVDFERNLSPVRHTTMGVGPHRCVGAGLARLEAILFI
EEWLKGIPSFRVKPGSPPRYRAGNVGALTELRLEWD

>CYP101C6(WG74_05650)*Citromicrobium* sp. JL477

MSAQPIPDHVPADRVVDFNVFAPPGGEQDFFAAWKSLLGGPGLVWTTENGGHWIGACGEVVRSLWGDAERFSN
EALAVTPGLGEVMEFIPLQQDPPEHKPFRMAVMKGFANNHVVAMEPLVREQARLLIEGLMGKGGCEFMSQFAE
ILPIHIFLTLIGVPPEDRAKLRPLGQQLTRPDGSMTVVELRDAADAYLEPYIRERLANPGSDLFSRILSVPIE
GRAWTFDEAQRMCRNLLFGGLDTVVAMMGNMIMHLARHPDDQDYLREDALRIPAAVDELMRRYPTVSVTRNCV
ETTEVDGVEVREGDLIYLPSVLHNLDPACFDDPEKVDFDRGLSPVRHTTMGVGPHRCVGAGLARLEAIVVLEE
WLARVPHFSLAEDVQPTFRGGNVGSLLQLQLELRWAE

>CYP101D1(Saro_0514)*Novosphingobium aromaticivorans*

MNAQTSTATQKHRVAPPPHVPGHLIREIDAYDLDGLEQGFHEAWKRVQQPDTPPLVWTPFTGGHWIATRGTLI
DEIYRSPERFSSRVIWVPREAGEAYDMVPTKLDPPEHTPYRKAIDKGLNLAEIRKLEDQIRTIAVEIIEGFAD
RGHCEFGSEFSTVFPVRVFLALAGLPVEDATKLGLLANEMTRPSGNTPEEQGRSLEAANKGFFEYVAPIIAAR
RGGSGTDLITRILNVEIDGKPMPDDRALGLVSLLLLGGLDTVVNFLGFMMIYLSRHPETVAEMRREPLKLQRG
VEELFRRFAVVSDARYVVSDMEFHGTMLKEGDLILLPTALHGLDDRHHDDPMTVDLSRRDVTHSTFAQGPHRC
AGMHLARLEVTVMLQEWLARIPEFRLKDRAVPIYHSGIVAAVENIPLEWEPQRVSA

>CYP101D2(Saro_1478)*Novosphingobium aromaticivorans*

MATNFDEAVRAKVERPANVPEDRVYEIDMYALNGIEDGYHEAWKKVQHPGIPDLIWTPFTGGHWIATNGDTVK
EVYSDPTRFSSEVIFLPKEAGEKYQMVPTKMDPPEHTPYRKALDKGLNLAKIRKVEDKVREVASSLIDSFAAR
GECDFAAEYAELFPVHVFMALADLPLEDIPVLSEYARQMTRPEGNTPEEMATDLEAGNNGFYAYVDPIIRARV
GGDGDDLITLMVNSEINGERIAHDKAQGLISLLLLGGLDTVVNFLSFFMIHLARHPELVAELRSDPLKLMRGA

EEMFRRFPVVSEARMVAKDQEYKGVFLKRGDMILLPTALHGLDDAANPEPWKLDFSRRSISHSTFGGGPHRCA
GMHLARMEVIVTLEEWLKRIPEFSFKEGETPIYHSGIVAAVENVPLVWPIAR

>CYP101E1(Swit_0361)*Sphingomonas wittichii*

MQDVMERALAPRPDHVPADLVRDFDLYDIPGAAEDIQAAYRAIQQASPDIFWTPHNGGHWVATRGEDIIEMQR
DYSRFSHRRIVLPPMPDAPRQIPLEMDPPEHARYRRPLMQALMPSIVAELEAKVRQVAIDAVERLLPRGECEF
VEDFAKVLPIHVFLALVELPLDDKAYLLTLAEDSVRGRDAETRGRSQQLMGAYLLPWIRARRETPGNDLLSKL
VNVDIGGERISEAEATSYATLVLFGGLDTVAGMIAFIARFLAQNPGQRRQLVERLDDDAFVRNAIEEMVRRHG
LANTARVIASDFDYKGVSFRAGDRILPANLFVGVDDRLNPDPLVVDFSREKPVHAAFGNGAHACPGAVLARRE
IRIFLQEWLSRIPDFRIRPGTSPVLATGMVNGVLRLELVWP

>CYP101E2(TZ53_05350)*Sphingobium* sp. YBL2

MTSVAEGELAPRPDHVPAALVRDFNIYDIPGSAEDVQAAYAAIQQANPDIFWTPHNGGHWVATRSDDIIAMQR
DYHHFSHKHIVLPPMPEGTPRQIPLEMDPPEHARYRRPLMQSLMPAVVSELESKVRDVAVEAIERVLPRGECE
FIEDFAKILPIHVFLELVDLPISDKHRLLPIAERSVRGHTAEIRLQAQQEMGGYLIDEIRARRDNPGEDLLSK
LVNVNVGEGRISEMEAVSYATLVLFGGLDTVAGMIGFIARFLALNPGHRRQLVERLDDEDFIKHAIEEMIRRH
GLANTSRVITENLEYKGVYFQAGDRILPANLWVGTDDRVNADPLVVDFSREKPVHAAFGNGAHACPGAVLARR
EIRIFLQEWLSRIPDFRIKPGTKPVLATGMVNGVLSLDLCWP

>CYP101Q1(K663_16320)*Sphingobium* sp. MI1205

MFSLASEVPHHVPPECVVDFDYYRLTDDGTDFFEAWKKLQRSVNTDMVWTPRNGGHWIAVRGKLIQALFTDNV
NLSSAVTGVPRETSIANFIPLQSDPPMHAIYRAPVVRGLGAKYILGLQGTIRSLTRELINAFKHRGGCEFVDD
FAEQMPFGVFLTLIGLPLSDREMLRQVGTQLSRPVDKTAEEVVALVSDYLRPYVIERLEQPGDDLISRILATP
IDGRAWTLDEAMRLSLNMLIGGLDTVAAMIGFVTYFLACSPDHQTAIREDREIIPRAADEFIRRFGMVTNARV
AVRDIQVGELTLRTDDIVLLPTMLHNLDDEAFPNAMKVDFNRGLCRHSTMGGGAHRCVGAGLAREELIAFLEE
WFDAVPEFELDPARSVRMSSGPVASMTCLPLRWA

>CYP101R1(AZL_b01800)*Azospirillum* sp. B510

MSGCPFAKLAHAADYPDHVPADRVFDVEVYAIPGADRDFHLALKAMHDHGLPDVFWTPRQGGHWVVTRYEDMM
GVLADPVNFSNGAIVVPKSRNPTRENGSLPLYPLTADLPEHTAYRALLSPTFSPRGVQQLSELARAVAIRLVE
ELKPRGSCEFITDFAQHLPIEIFMSIVDVPAEDREWLLALTDRMVRPAEPEDMHKTMGTLFGYVRDLVAKRKA
KPGNDLISSIIAGKVFGRPMDDDELTGMCALILIGGMDTVVSAMGFAAWFLARHPDHRRQLLENPELIPNAVD
EMLRRHSIVNIGRLVKNDVTVGGVAMKAGDMLVMPSPLGSMDERKFPDPLTVDFTRSNSGEYSTFGKGPHRCP
GANLGRAELRIFVEEWLRRIPDFHVRDEAAVGMSCGINGTIYSLPLEWTPQEWSCPGKVAARNVSTTVRQPEL

>CYP102A6(blr_2882)*Bradyrhizobium diazoefficiens* USDA 110

MSSKNRLDPIPQPPTKPVVGNMLSLDSAAPVQHLTRLAKELGPIFWLDMMGSPIVVVSGHDLVDELSDEKRFD
KTVRGALRRVRAVGGDGLFTADTREPNWSKAHNILLQPFGNRAMQSYHPSMVDIAEQLVQKWERLNADDEIDV
VHDMTALTLDTIGLCGFDYRFNSFYRRDYHPFVESLVRSLETIMMTRGLPFEQIWMQKRRKTLAEDVAFMNKM
VDEIIAERRKSAEGIDDKKDMLAAMMTGVDRSTGEQLDDVNIRYQINTFLIAGHETTSGLLSYTLYALLKHPD
ILKKAYDEVDRVFGPDVNAKPTYQQVTQLTYITQILKEALRLWPPAPAYGISPLADETIGGGKYKLRKGTFIT
ILVTALHRDPSVWGPNPDAFDPENFSREAEAKRPINAWKPFGNGQRACIGRGFAMHEAALALGMILQRFKLID
HQRYQMHLKETLTMKPEGFKIKVRPRADRERGAYGGPVAAVSSAPRAPRQPTARPGHNTPMLVLYGSNLGTAE
ELATRMADLAEINGFAVHLGALDEYVGKLPQEGGVLIICASYNGAPPDNATQFVKWLGSDLPKDAFANVRYAV
FGCGNSDWAATYQSVPRFIDEQLSGHGARAVYPRGEGDARSDLDGQFQKWFPAAAQVATKEFGIDWNFTRTAE
DDPLYAIEPVAVTAVNTIVAQGGAVAMKVLVNDELQNKSGSNPSERSTRHIEVQLPSNITYRVGDHLSVVPRN
DPTLVDSVARRFGFLPADQIRLQVAEGRRAQLPVGEAVSVGRLLSEFVELQQVATRKQIQIMAEHTRCPVTKP
KLLAFVGEEAEPAERYRTEILAMRKSVYDLLLEYPACELPFHVYLEMLSLLAPRYYSISSSPSVDPARCSITV
GVVEGPAASGRGVYKGICSNYLANRRASDAIYATVRETKAGFRLPDDSSVPIIMIGPGTGLAPFRGFLQERAA
RKAKGASLGPAMLFFGCRHPDQDFLYADELKALAASGVTELFTAFSRADGPKTYVQHVLAAQKDKVWPLIEQG
AIIYVCGDGGQMEPDVKAALVAIRHEKSGSDTATAARWIEEMGATNRYVLDVWAGG

>CYP102A10(ELI_00100)*Erythrobacter litoralis* HTCC2594

MDAPTALAPIPQPPGKPIVGNAFTVDSSRLIQSLMELAEEYGPIFQLEVMGTPLVFVSGADMVAEICDESRFD
KTVRGPLKRLRLIAGDGLFTGDTDDPNWAKAHHILLPSFSQKAMGSYLPMMTDIASQLMLKWERLNSDDVIDV
PMDMVRLTLDTIGVCGFGYRFNSFYREDFHPFIEALNRTLDTTQKMRGLPGEKLLKRQQIEQLNEDAAYMNNL
VDEIIRERRQTGESGQGDLLDFMLSGRDPVTGERLSDENIRYQINTFLIAGHETTSGLLSFTLYYLLKNRDVL
QRAYAEVDEVLGRNIDQTPTLSQIGRLPYIRAILSEALRLWPTAPAMGLAPFEDEVLGGKYAIAKGTFTTVLI
PSLHRDKLVWGENPEAFNPDNFSPKAEAARPPHAYKPFGNGQRACIGRQFAIQESILVLGMLLQRFELFDHAD
YQLRIKETLSIKPDGFTIKARLRHDVERGGVATVEPESKTPDQAAAVPSHGTPLLVLYGSNLGSSEGFARELA
QRGEFSGFDVTMAPLDAHVAKLPTDGAVAIACASYNGMPPDNAAKFVDWLEQADAADAPLSNVSYLVLGCGNS
DWAATFQVVPRKIDALMEQHGAERLVPAEELDARGDLDTQFHDWLDGLIPQLGDAFDIDLESGFDAVFEPLYT
VEITDSITGNTVADRVGAREVEVVANRELKDTSKDEGRSTRHLEVRLPEGMEYEPGDHLCVVPVNDPAVVDRL
LKRFGLDRDTFVRIESRSDMRGPFPSGSTFSVLNLAETAGELQAVATRKDIATLARYSECPNSRAALEALAAP
PSADGTDRYTSEVLEKRRSVLDMLEEFPACDVPLAVFLELIPFLSPRYYSISSAPEANQGLCSITVGVVKGPA
LAGTGEFKGTCSAYLADLPPGDRFRAVVRKPTAQFRLPDNPETPVIMIGPGTGVAPFRAFLQRRDHLQEDGAV
LGEAMLFFGCRHPDIDYLYREELDDYDQRGVATVHAAFSRHDGSRTYVQDLIAREADRVWELIEQDARIYVCG
DGARMEPDVRKALMAIYAEKKSSDEASAKAWIDDLVAQDRYLLDVWVG

>CYP102A12(RPB_3645)*Rhodopseudomonas palustris* HaA2

MSSSNKLAPIPHPPKQPVVGNMLSIDTKAPVQHLVRLAEELGPIFWLDMMGAPIVIVSGYDLVDEISDEKRFD
KAVRGALRRVRTVGGDGLFTADTSEPNWSKAHNILLTPFGGRAMQSYHPSMVDIAEQLVKKWERLNADDEIDV
VHDMTALTLDTIGLCGFDYRFNSFYRRDYHPFVESLVRSLETIMMTRGLPLENLWMKKRRDTLAEDVAFMNAM
VDEIIAERRKAAAVADKMDMLGAMMTGVDKVTGEPLDDVNIRYQINTFLIAGHETTSGLLSCAIYALLKHPEV
LQKAYDEVDRVLGADTSVEPSYQQVNQLGYITQILKETLRLWPPAPAYGVAPIQDETIGGQYHLKRGTFTTVL
VLALHRDPSIWGPNPDAFDPENFSREAESKRPANAWKPFGNGQRACIGRGFAMHEAALALGMILQRFKLIDHT
RYRMVLKETLTIKPEGFKIKVRPRSDKDRATRIASGVSHSVAPAPAAPRARPGHNTPLLVLYGSNLGTAEELA
HRVADLADLNGFATRLGALDQYVGQLPEEGGVLIFAASYNGAPPDNATQFVRWLSGDLPPDAFAKLRYAVFGC
GNRDWTATYQAIPRLIDERLAAHGGRNIFVRGEGDARDDLEGQFEAWFATLGPLAVKEFGIDAAFDRGADDTP
LYGIEPLAPAASQPLAATGVAVAMRVLENRELQDRAASGRSTRHIEIALPQGMSYRVGDHLSVIPRNDPALVA
AVAQRFGFAPDDQIRLSAAPGRRAQLPVGEAVSIGGLLGDHVELQQVATRKQIVALAAHTRCPQTRPKLQALA
GGDGAADDAYRAEVLGKRRSVFDLLQEHPACELPFAAYLEMLTPLQPRYYSISSSPARDPARASVTVAVVEGP
ALSGRGIYRGACSSWLAGRGSGDTVQATVRATKACFRLPDDDRVPLIMIGPGTGVAPFRGFLQERSARKVGGA
TLGPALLFFGCRHPAQDYLYADELQGFAADGIVELHAAFSRGDGPKTYVQHLIAAQKDRVFALIEQGAIVYVC
GDGGRMEPDVKAALCAIHRERSGADATAAAAWIADLGARDRYVLDVWASV

>CYP102A12(RPD_1820)*Rhodopseudomonas palustris* BisB5

MPSTNKLDPIPHPPKKPVVGNMLSLDTTAPVQHLVRLAKELGPIFWLDMMGAPLVIVSGYDLVDEISDEKRFD
KAVRGALRRARAVGGDGLFTADTKEPNWSKAHNILLTPFGGRAMQSYHPSMVDIAEQLVKKWERLNADDEIDV
VHDMTALTLDTIGLCGFDYRFNSFYRRDYHPFVESLVRSLETIMMTRGLPLENLWMKKRRETLADDVVFMNAM
VDEIIAERRKASESAADKKDMLGAMLAGVDRATGEPLDDVNIRYQINTFLIAGHETTSGLLSCAIYALLKHPD
VLQKAYDEVDRVLGSDTAVRPSYQQVNQLSYITQILKETLRMWPPAPAYGVAPIKDEVIGGKYHLKRGTFVTV
LVLALHRDPAIWGPNPDAFDPENFSREAESKRPANAWKPFGNGQRACIGRGFAMHEAALALGMILQRFQLIDH
QRYRMVLKETLTIKPEGFKIKVRPRSDKDRGDFVAAGASQVSTPALAQAAPRARPDHNTPLLVLYGSNLGTAE
ELATRVADLAELNGFSTRLGALDQYVGHLPEEGGVLIFTASYNGAPPDNATQFVQWLSGDLPKDAFAKLRYAV
FGCGNRDWTATYQAIPRLVDERLAAHGGRNIFLRGEGDARDDLEGQFESWFAKLGPLAVKEFGIDAKFARAVD
DAPLYRIEPVAPAAGNAVAAAGGAVPMKVLANRELQDCAASGRSTRHIEIALPEGISYRVGDHLSVMPRNDPA
LVAAVAQRLGFAPDDQIKLQVAPGRRAQLPIGEAISVGRLLGDFVELQQVATRKQIAVMAEHTRCPQTRPKLQ
ALAGGDGAADEAYRAGVLAKRKSVYDLMQEHPACELPLHAYLEMLSPLAPRYYSISSSPLRDPSRAAITVAVV
DGPALSGRGHYRGVCSTWLAGRSVGDTIHATVRATKAGFRLPDDDRVPLIMIGPGTGLAPFRGFLQERAARQQ
NGATLGPALLFFGCRHPAQDYLYADELQGFAAEGVVELHTAFSRGEGPKTYVQHLIAAQKDRVFTLIEQGAII
YVCGDGGKMEPDVRAALMAIHRERSGADAAAASTWIDDLGACNRYVLDVWASA

>CYP102A6(BJ6T_68860)*Bradyrhizobium japonicum* USDA 6

MSSKNRLDPIPQPPTKPVVGNMLSLDSAAPVQHLTRLAKELGPIFWLDMMGSPIVVVSGHDLVDELSDEKRFD
KTVRGALRRVRAVGGDGLFTADTREPNWSKAHNILLQPFGNRAMQSYHPSMVDIAEQLVNKWERLNADDEIDV
VHDMTALTLDTIGLCGFEYRFNSFYRRDYHPFVESLVRSLETIMMTRGLPFEQLWMQKRRKTLAEDVAFMNKM
VDEIIAERRKSAEGVDDKKDMLAAMMTGVDRSTGEQLDDINIRYQINTFLIAGHETTSGLLSYTLYALLKHPD
ILKKAYDEVDRVFGPDVNAKPTYQQVTQLTYITQILKEALRLWPPAPAYGISPLNDETIGGGNYRLRKGTFIT
ILVTALHRDPSVWGPNPDAFNPENFSREAEAKRPINAWKPFGNGQRACIGRGFAMHEAALALGMVLQRFKLID
HQRYQMHLKETLTMKPEGFKIKVRPRADRERGAYGGPVAVASSGPRAPRKPTTRPGHNTPMLVLYGSNLGTAE
ELATRMADLAEINGFAVHLGPLDDYVGKLPHEGGVLIICASYNGAPPDNATQFVKWLGDDLPEDAFANVRYAV
FGCGNSDWAATYQSVPRFIDEQLSKHGARAVYPRGEGDARSDLDGQFQKWFPAAAQVATKEFGIDWNFTRTAE
DDPLYAIEPVAVTAVNTIVAQGGAVAMKVLVNDELQNKFGANPSERSTRHIEVQLPSNITYRVGDHLSVVPRN
DPTLVDSVARRFGFLPADQIRLQVAEGRRAQLPVGDAVSVGRLLSEFVELQQVATRKQIQIMAEHTRCPVTKP
KLLAFVGEEAEPLERYRTEILARRKSVFDMLLEYPACELPFHVYLEMLSLLAPRYYSISSSPSVDPARCSVTV
GVVEGPAASGRGTYKGICSNYLSNRRAGDAIYATVRETKAGFRLPDESSVPIIMIGPGTGLAPFRGFLQERAA
RKAKGAALGPAMLFFGCRHPDQDFLYAEELKGLAASGITELFTAFSRADGPKTYVQHMLAAQKDRVWPLIEQG
AIIYVCGDGGKMEPDVKAALVAIHREKSGSDAAAAARWIEEMGAKNRYVLDVWAGG

>CYP102A6(RN69_33415)*Bradyrhizobium japonicum* E109

MSSKNRLDPIPQPPTKPVVGNMLSLDSAAPVQHLTRLAKELGPIFWLDMMGSPIVVVSGHDLVDELSDEKRFD
KTVRGALRRVRAVGGDGLFTADTREPNWSKAHNILLQPFGNRAMQSYHPSMVDIAEQLVNKWERLNADDEIDV
VHDMTALTLDTIGLCGFEYRFNSFYRRDYHPFVESLVRSLETIMMTRGLPFEQLWMQKRRKTLAEDVAFMNKM
VDEIIAERRKSAEGVDDKKDMLAAMMTGVDRSTGEQLDDINIRYQINTFLIAGHETTSGLLSYTLYALLKHPD
ILKKAYDEVDRVFGPDVNAKPTYQQVTQLTYITQILKEALRLWPPAPAYGISPLNDETIGGGNYRLRKGTFIT
ILVTALHRDPSVWGPNPDAFNPENFSREAEAKRPINAWKPFGNGQRACIGRGFAMHEAALALGMVLQRFKLID
HQRYQMHLKETLTMKPEGFKIKVRPRADRERGAYGGPVAVASSGPRAPRKPTTRPGHNTPMLVLYGSNLGTAE
ELATRMADLAEINGFAVHLGPLDDYVGKLPHEGGVLIICASYNGAPPDNATQFVKWLGDDLPEDAFANVRYAV
FGCGNSDWAATYQSVPRFIDEQLSKHGARAVYPRGEGDARSDLDGQFQKWFPAAAQVATKEFGIDWNFTRTAE
DDPLYAIEPVAVTAVNTIVAQGGAVAMKVLVNDELQNKFGANPSERSTRHIEVQLPSNITYRVGDHLSVVPRN
DPTLVDSVARRFGFLPADQIRLQVAEGRRAQLPVGDAVSVGRLLSEFVELQQVATRKQIQIMAEHTRCPVTKP
KLLAFVGEEAEPLERYRTEILARRKSVFDMLLEYPACELPFHVYLEMLSLLAPRYYSISSSPSVDPARCSVTV
GVVEGPAASGRGTYKGICSNYLSNRRAGDAIYATVRETKAGFRLPDESSVPIIMIGPGTGLAPFRGFLQERAA
RKAKGAALGPAMLFFGCRHPDQDFLYAEELKGLAASGITELFTAFSRADGPKTYVQHMLAAQKDRVWPLIEQG
AIIYVCGDGGKMEPDVKAALVAIHREKSGSDAAAAARWIEEMGAKNRYVLDVWAGG

>CYP102A6(S23_51340)*Bradyrhizobium* sp. S23321

MSSKNRLDPIPQPPTKPVVGNMLSLDSAAPVQHLTRLAKELGPIFWLDMMGSPIVVVSGHDLVDELSDEKRFD
KTVRGALRRVRAVGGDGLFTADTREPNWSKAHNILLQPFGNRAMQSYHPSMVDIAEQLVNKWERLNADDEIDV
VHDMTALTLDTIGLCGFDYRFNSFYRRDYHPFVESLVRSLETIMMTRGLPFEQLWMQKRRKTLGEDVAFMNKM
VDEIIAERRKSAEGIDDKKDMLAAMMTGVDRATGEQLDDVNIRYQINTFLIAGHETTSGLLSCTIYALLKHPD
ILKKAYDEVDRVFGPDVNAKPTYQQVTQLTYITQILKEALRLWPPAPAYGISPLKDEVIGGGKYRLRKGTFTT
ILVTALHRDPSVWGPNPDAFDPENFSREAEAKRPINAWKPFGNGQRACIGRGFAMHEAALALGMILQRFKLID
HQRYQMHLKETLTMKPEGFKIKVRPRADRERGAYGGPVAASAAPKAPRQPTARPGHNTPMLVLYGSNLGTAEE
LATRMADLAEINGFAVHLGPLDDYVGKLPQEGGVLIICASYNGAPPDNATQFVKWLGDDLPKDAFANVRYAVF
GCGNSDWAATYQSVPRFIDEQLSKHGGRAVYPRGEGDARSDLDGQFQKWFPAAAQVATKEFGIDWNFTRTAED
DPLYAIEPVAVTAVNTIVAQGGAVAMKVLVNDELQNKTGSNPSERSTRHIEVQLPANISYRVGDHLSVVPRND
PTLVDSVARRFGFLPADQIRLQVAEGRRAQLPVGDAVSVGRLLSEFVELQQVATRKQIQIMAEHTRCPVTKPK
LLAFVGEEPETLERYRTEILARRKSVFDLLLEYPACELPFHVYLEMLSLLAPRYYSISSSPSVDPARCSITVG
VVEGPAASGRGTYKGVCSNYLANRRAGDAIYATVRETKAGFRLPDDPSVPVIMIGPGTGLAPFRGFLQERAAR
KAKGAALGPAMLFFGCRHPDQDFLYKDELTALAASGITELFTAFSRADGPKTYVQHVLAAQKDKVWPLIEQGA
IVYVCGDGGKMEPDVKAALVAIHREKSGSDAVASARWIEEMGAKNRYVLDVWAGG

>CYP102A6(BCCGELA001_12905)*Bradyrhizobium* sp. CCGE-LA001

MSSKNRLDPIPHPPTKPVVGNMLSLDAAAPVQHLTRLAKELGPIFWLDMMGSPIVVVSGHDLVDELSDEKRFD
KTVRGALRRVRAVGGDGLFTADTREPNWSKAHNILLQPFGNRAMQSYHPSMVDIAEQLVQKWERLNADDEIDV
VHDMTALTLDTIGLCGFEYRFNSFYRRDYHPFVESLVRSLETIMMTRGLPFEQIWMQKRRKTLAEDVAFMNRM
VDEIIAERRKSSEGIDDKKDMLAAMMTGVDRATGEQLDDINIRYQINTFLIAGHETTSGLLSYTLYALLKHPD

ILKKAYDEVDRVFGPDVNAKPTYQQVTQLAYITQILKEALRLWPPAPAYGISPLNDETIGGGKYRLRKGTFTT
ILVTALHRDPSVWGPNPDAFDPENFSKEAEAKRPINAWKPFGNGQRACIGRGFAMHEAALALGMILQRFKLID
HQRYQMHLKETLTMKPEGFKIKVRPRADRDRGAYGGPVAAVASAPKAQRQPTTRPGHNTPMLVLYGSNLGTAE
ELATRMADLAEINGFAVHLGPLDDYVGKLPQEGGVLIICASYNGAPPDNATQFVKWLGSDLPNDAFANVRYAV
FGCGNSDWAATYQSVPRFIDEQLAKRGARAVYPRGEGDARSDLDGQFQKWFPAAAQVATKEFGIDWNFTRTAE
DDPLYAIEPVAVTAVNTIVAQGGAVAMKVLVNDELQNRSGANRSERSTRHIEVQLPSNLTYRVGDHLSVVPRN
DPTLVDSVARRFGFLPADQIRLQVAEGRRAQLPVGAAVSVGRLLSEFVELQQVATRKQIQTMAEHTRCPVTKP
KLLAFVGEEDEPLERYRSEILAKRKSIFDLLLEYPACELPFHVYLEMLSLLAPRYYSISSSPSVDPARCSVTV
GVVEGPAASGRGVYKGICSNYLANRRAGDAIYATVRETKAGFRLPDDPSVPIIMIGPGTGLAPFRGFLQERAA
RKAKGATLGPALLFFGCRHPDQDFLYADELKALAAGGITELFTAFSRADGPKTYVQHVLAAQKDKVWPLIEQG
AIIYVCGDGSKMEPDVKVALVAIHGEKSGSDAAASARWIDEMGATNRYVLDVWAGG

>CYP102A6(BF49_1812)*Bradyrhizobium* sp. BF49

MSSKNRLDPIPQPPTKPVVGNMLSLDSAAPVQHLTRLAKELGPIFWLDMMGSPIVVVSGHDLVDELSDEKRFD
KTVRGALRRVRAVGGDGLFTADTREPNWSKAHNILLQPFGNRAMQSYHPSMVDIAEQLVGKWERLNADDEIDV
VHDMTALTLDTIGLCGFDYRFNSFYRRDYHPFVESLVRSLETIMMTRGLPLEQLWMQKRRKTMAEDVDFMNKM
VDEIIAERRKGGDATDDKKDMLAAMMTGVDRSTGEQLDDVNIRYQINTFLIAGHETTSGLLSCTLYALLKHPD
ILKKAYDEVDLVLGPNVDVRPTYQQVTQLTYITQILKEALRLWPPAPAYGISPLNDETIGGGKYRLRKGTFTT
ILVTALHRDPSVWGPNPDAFDPENFSREAEAKRPINAWKPFGNGQRACIGRGFAMHEAALALGMILQRFRLID
HQRYQMHLKETLTMKPEGFKIKVRPRADRERGAYGGPVAVSSAPKAPRQPTARPGHNTPMLVLYGSNLGTAEE
LATRMADLAEINGFAVHLGPLDDYVGKLPQEGGVLIICASYNGAPPDNAIQFVKWLGDELPKDAFANVRYAVF
GCGNSDWAATYQSVPRFIDEQLSAHGARAVYPRGEGDARSDLDGQFQKWFPAAAQVATKEFGIDWNFTRTAED
DPLYAIEPVAVTAVNTIVAQGGAVAMKVLVNDELQNKAGSNPSERSTRHIEVQLPSNINYRVGDHLSVVPRND
PTLVDSVARRFGFLPADRIRLQVAEGRRAQLPVGDAVSVGRLLSEFVELQQVATRKQIQIMAEHTRCPVTQPK
LLAFVGEEPETLERYRSEILAKRKSVFDLLLEYPACELPFHVYLEMLSLLAPRYYSISSSPSVDPVRCSVTIG
VVEGPAASGRGTYKGICSNYLANRRAGDVIHATVRETKAGFRLPDDPSVPIVMIGPGTGLAPFRGFLQERAAR
KAKGAALGPAMLFFGCRHPDQDFLYADELKALAASGITELFTAFSRAEGPKTYVQHVLAAQKDKVWPLIEQGA
IVYVCGDGSKMEPDVKAALVAIHREKSGGDAATAARWIEEMGARNRYVLDVWAGG

>CYP102A28(LMTR13_28840)*Bradyrhizobium icense*

MASSNKLSPIPHPPKKPVVGNMLSLDPNAPVQHLVKLSKELGPIFWLDMMGAPIVIVAGHDLIDELSDEKRFD
KTVRGSLRRVRAVGGDGLFTADTTEPNWSKAHNILLQPFGNRAMQSYHPSMVDIAEQLVKKWERLNADEEIDV
VHDMTALTLDTIGLCGFDYRFNSFYRRDYHPFVESLVRSLETIMMTRGLPLENLWMQKRRKTLAQDVVFMNKM
VDEIIAERRGSAEATEAKKDMLGAMMTGIDRSTGEQLDDINIRYQINTFLIAGHETTSGLLSCTIYALLKHPE
VLKKAYEEVDRVLGPDINARPTYQQVTQLTYITQILKEALRLWPPAPAYGITPLQDEAIGGGKYKLKKNTFIT
VLVMALHRDPSVWGPNPDVFDPENFSREAEAKRPINAWKPFGNGQRACIGRGFAMHEAALAIGMILQRFKLVD
VHRYQMHLKETLTVKPDGFKIKVRPRGDKDRGVFAGRTAVAAAASSAAAPQARTRPGHNTPLLVLYGSNLGTA
EELATRVADLAEVNGFATKLGALDDYVGKLPEQGALMIFCASYNGAAPDNATQFVKWLDSGLPKDAFAKVRYA
VFGCGNSDWAATYQSVPRLIDEGLAVHGARSLYARGEGDARSDLDGQFEKWFAAAAPAAMKELGVDSGFARSA
DDAPLYSIEPVAPSAVHTIVAQGGVAPMKVLVNSELQNKTGASRSDRSTRHIEVELPPGIAYRVGDHLSVVPR
NDPALVDSVARRFGFLPADQIRLHVAEGRRAQLPVGDAVSVGRLLTEFVELQQIATRKQIQIMSEHTRCPVTK
PKLLAYVGDDGASTELYRSDVLAKRKSVFDLLEEHPACELPFHAYLEMLSLLAPRYYSISSSPSSDPARCSVT
VGVVEAPASSGRGVYKGVCSNYLAGRRVGDTVHATIRETKAGFRLPDDAAQPVIMIGPGTGLAPFRGFLQERA
HRKAQGAKLGPAMLFFGCRHPEQDFLYADELKAFANDGIAALHTAFSRAEGPKTYVQHQVTAQKDRVWSLIEQ
GAIVYVCGDGGKMEPDVKAALVAIYREKSGADAEAGLHWIDDLGTKNRYVLDVWAGG

>CYP102K1(AZC_3520)*Azorhizobium caulinodans*

MSETSTPPALKSVDLGLAAGTVRVGWFPGASREAVLAAVRGAAGLSGETAFHVETADGVTIALDDSVPAGTRL
RLVVQGEVPSSAAPALHRPVPGPKPYPVVGNLPELHNAEGLVAAVDALHAKHGEFFAFQVGGKRAYFCSDADI
ISEMCAAPDVFAKLVEGRGGLGNLAEKSVGSALFTASDNDPLWHQAHRILAPAFGATALKNYYGRIVEVADDL
LDHLDRLAPGESFLATDLMTRMTFEAISYAAFNRRYGAIDSPALPAFVEAMNVVLTDAMAEPKRLLPEVFYHE
ARKTRAAADKIMLEEVETIIAERRFAMAAGAPVPTDLLQVMLTTPDRVTGQKLPDDNIRGQLIVLLIAGHETT
SGMLAYALYHLWKYPETMEKLIAEVDEVLGRDFSYKPTYEDVGRLAYTQRVLKEALRLCPPVPMFPRYVTRDA
TVGNGRYDLKAGERIFVSLSAMQKNPRFWGADALVFRPERFGPEEEKHHHPHAYHPFGMGARSCIGFQFALVE
AKMVLARFIQRFTARPKDPHYVLCHKQALTVKPDHLDMLLERRPEVKGRFPVRTEAPKTQGAPLAVAQAGGRP

MRVLYGSNMGGCRDIALSLAQQAGARGFAATVAELDEQVGQPWLTDGPVVIVTSTYNGTPPDNAARFAKWLET
APAGVCAGVRHAVLGCGNTQWHQTFQKFPKTIASGLAALGGTALLDEGTADAAGDYEAAVEGWTAALWPALEA
AFGGAAGAAVIGSAEAAAEAPAVKVEVVNFAGAATGAAPRTGTRLDQGAQLSRIRVNRELLSPGAQGSTRHIE
IPLPAGTSYAAGDHLAVFPVNPPALVAAAAARCGLAPETQVLLTALHPDAASEAGLPFGVPVSVGELLAEHVD
LAGPVTRRDLRAWAQAAQCPPDRARIAGWLSDFPSAVAEAKPRMEDLLAQVPSVQLDLAALLTVRPALKPRYY
SISSSPLMSPDACALTVGVHQFTTADGVRHDGLCSSYLVSCGEDAPVRVLVKDTGSTFHLPADPAVPLILVGP
GTGLAPLRGFIQERHALRAQGRATGPVLLFFGCRDDGDYLYREELEAYRDEGTLSLLAVGFSRRPGTPRTYVQ
DLLRTHGDAVREQVAAGASILICGNARTMAPDVHAAFLELLGAGAVAELEAGGRYLQDVWASS

>CYP102AC1(SAMCFNEI73_pB0314)*Sinorhizobium americanum*

MSKLEEIPQPKGLPVLGHITSINPHAPVQSLIRLSKELGPIFRLQTFGRNVIIVGNQELTAELCDETRFCKAL
HPPLAELRTIGNDGLFTADNNESNWGKAHRLLTPAFGPLGLKSMFSKMVDVVDQMLVRWERFGSDAEIDVADN
MTRVALDTIALCAFDHRFNSFYRNERHSFVTAMEGALKEVSRRALFPPGASKLMFLRNRRFKAHTRTLRALAD
DLIDKRRLDARLGERSDLLDIMLTTKDQETGEMLSRENIGYQMITFLIAGHETTSGLLSFATWLLLKNPEELS
RLQAHVDDALGGEPPTAQDLGKLDCVEQALMETLRLWPTAPGFAVHPIENTTLAGSYRLTPDDILLILLPVLH
RDPKVWDEPDVFRPARFNFDHAKEALQHAWKPFGNGQRACLGRGFAMQEAVLVMAMMLQRFYISLVDDSYELV
VGETLTMKPIGLRIRAKRRHWSAGMPRRSAPAASKEQVLRRVPRSIADRGPVLIIYGSNTGMSESFAKRIGSE
AATRGYAPVVAPADDYSFGLPVDIPFVVVTASYEGLPPNNARRFLAWAESLPPDTLAGLPFAVFGCGNRHWVQ
TWQAVPKRLEAALARAGAVPVAKRGEADAGGDVLASLDAWSAELWEALAAEV

>CYP103A1(Ach5_52420)*Agrobacterium tumefaciens* Ach5

MIANSSTDVSVADQKFLNVAKSNQIDPDAVPISRLDSEGHSIFAEWRPKRPFLRREDGIFLVLRADHIFLLGT
DPRTRQIETELMLNRGVKAGAVFDFIDHSMLFSNGETHGKRRSGLSKAFSFRMVEALRPEIAKITECLWDDLQ
KVDDFNFTEMYASQLPALTIASVLGLPSEDTPFFTRLVYKVSRCLSPSWRDEEFEEIEASAIELQDYVRSVIA
DSGRRMRDDFLSRYLKAVREAGTLSPIEEIMQLMLIILAGSDTTRTAMVMVTALALQNPALWSSLRGNQSYVA
AAVEEGLRFEPPVGSFPRLALKDIDLDGYVLPKGSLLALSVMSGLRDEKHYEHPQLFDVGRQQMRWHLGFGAG
VHRCLGETLARIELQEGLRTLLRRAPNLAVVGDWPRMMGHGGIRRATDMMVKLSFDL

>CYP103A2(Atu_6150)*Agrobacterium fabrum*

MITSSISGTDQQFQNATQPKELDPDAVPVSRLDSEGHEIFAEWRPKRPFLRREDGVFLVLRADDIFLLGTDPR
TRQIETELMLNRGVTRGAVFDLIRYSMLFSNGEVHVKRRSAFAKTFAFRMIDALRPEITKLTEHLWDDVPRVD
DFDFAEMYASKLPALTIASVLGLPFGDAPFFTRLVYNVSRCLSPSWGEDDFPEIEASAVELQDYVRAVVADRS
RRISDDFLSCYLKAVREEGTLSPIEEIMQLVFLILAGSDATRNAMVMLPTLLLQNPVVWSSLCHDQSGVAAAV
EEGLRFEPSVGSFPRLALEDIDLDGYVLPKGSFLALSIMSGLRDERHYEHPQLFDIKRKQMRRHLGFGAGVHR
CLGEALARIELQEGLRTLLRRAPSLRVTGDWPRMIGHGGARRATGMTVNLGVDR

>CYP103A4(Avi_8251)*Agrobacterium vitis*

MISELNPENVSVAALDRFGHEIFKEWRPKRPFLHRQDGVYVVLRADDVLGLSSDPRTRQIETELMLNRGVNEG
AVFDFVRYSMLFSNKETHRRRRSPFTRTFAFRMIENLRPQIRQLTEMLFRDLKELGSFNFVAEYSSKLPAVTI
ASLLGLPSSDIPYFTQLVYRVARCLSPSWRDADFPDIEKSAVEFKNYVQAVINDRRTDPRDNFLSSYINATRE
AEDLSPVEGLTQLMLIILAGTDTTKTGLTALTGQLLQHRQAWDALLKDVTLVPAAVEEGLRFEPPVGSYPRSA
RVDIDLDGFILPKGSLLALCTMSALRDEKHYAHPQLFDIHRKQMRWHMVFGAGEHRCLGEALARLEMQEGLAT
VLRHAPNLSIEGEWQGIQGHGGVRRIAEMRVGFMREN

>CYP104A1(Ach5_52430)*Agrobacterium tumefaciens* Ach5

MLFAPVDDVTTIDDLTLDPYPIYRRMRVQNPVVHVASVRRTFLTKAFDTKMVKDDPSRFSSDDPSTPMKPAFQ
AHTLMRKDGTEHARERMAMARAFAPKAIADHWAPIYRDIVNEYLDRLPRGDTVDLFAEICGPVAARILAHILG
ICEASDVEIIRWSQRLIDGAGNFGWRSELFERSDEANAEMNCLFNDLVKKHRSAPNPSAFATMLNAPDPIPLS
QIYANIKIAIGGGVNEPRDALGTILYGLLTNPEQLEEVKRQQCWGQAFEEGLRWVAPIQASSRLVREDTEIRG
FIVPKGDIVMTIQASANRDEDVFEDGESFNVFRPKSAHQSFGSGPHHCPGAQISRQTVGAIMLPILFDRFPDM
ILPHPELVQWRGFGFRGPINLPVTLR

>CYP104A2(Atu_6151)*Agrobacterium fabrum*

MQLAPVDRVTTIDDLTLDPYPIYRRMRAQTPVVRVASVMRTFLTKASDTKMVKDQPRRFSSDDPNTPMKPAFQ
AHTLMRKDGAEHARERMAMAKAFAPKTTAEHWAAIYRDIVNEYLDRLSRGSTVDLFAEICGPVAARILAHILG
ISEASDAEMIRWSQRLIEGAGNFGWRPEPFERANEANAEMNRLFDNLVEKHRAEPNPSAFAIMVTASDPIPMS
QIYANIKIAVGGGINEPRDALGTIIYGLLTNPEQFEEVKRQQCWGQVFEEGVRWVAPIQASSRLVLEDTEIRG
FLVPKGDTVMTIQASANRDEDVFDDGERFNVFRQNNAHQSFGSGPHHCAGAQISRQTVGAIMLPTLFERFPNM
TLTNPDAVQWRGFGFRGPISLPVTLI

>CYP104A5(A6B35_33065)*Mesorhizobium amorphae*

MRLVPVDRVSTIDELTFDPYPIYRRMRAQTPVVHVASVQRTFLTKASDTKMVKDQPERFSSDDPNTPMKPAFQ
AHTLMRKDGAEHARERMAMAKAFAPKTIAEHWAPIYRDIVNEYLDRLPRGGTVDLFAEICGPVAARILAHILG
ICGASDAEMIRWSQRLIDGAGNFGWQPEPFERSDEANAEMNRLFDNLIEKHRAEPNPSAFAIMVNAPDPIPMS
QMYANIKIAIGGGINEPRDALGTIVYGLLTNPEQFEEVQRQQCWGQAFEEGVRWIAPIQASSRLVREDTEIRG
FLIPKGDTVMTIQASANRDEEVFEDGERFNVFRQKIPHQSFGSGPHHCAGAQISRQTVGAIMLPMLFERFPNM
TLPDPQVVQWRGFGFRGPINLPVKLS

>CYP104A6(APZ00_12855)*Pannonibacter phragmitetus*

MPLAPLDETTTIAQLTDDPYPVYRRMRAETPVIRVASVKRTFLTKAVHTKAVKDNPELFSSDDPNTPMKPAFQ
AHTLMRKDGEEHRRERMAMQPAFTPKAIRCNWAELYQSIAEDYVSRLPRGQTVDLFTDLCGPVAARILAHILG
VEEATDEEMQRWSQLLIDGAGNFGWRPEPFEASDKANAEMNALFDRLQARHKAEPNASAYSIMLNAADPIPLS
QIYANIKIAIGGGINEPRDALATIIYGLLSNPDQLAEVRRQEAWEQAFEEGVRWVAPIQASSRRVLEDTEIGG
FEIPKGDTVMTIQASANRDEDIYQNGEEFFVFRDKNPHQAFGSGPHHCAGAHIARRTVGQIMLPLLFDRFPDM
ELPDPSAVKWKGFGFRGPINLPVKLN

>CYP104A7(ACP90_00230)*Labrenzia* sp. CP4

MAVAPVDETLTLADLTRDPYPIYKRMRAETPIVQVPAANRIFLTKAKHTKMVKDDPELYSSDDPVTPMRRAFL
AHTLMRKDGEEHRRERMAMQPAFSPKVIKTDWADLYRKIAAEYLDRLPRGETVDLFTDLCGPVAARILAHILG
LDEVGDADMQRWSQVLIDGAGNFGFADEPFARSDAANAEMDAVFDSLIARHLAEPNNSAFSVMLNARDPIPRS
QIYANIKIAIGGGINEPRDALATIIYGLLTNPDQLEEVKRQNAWADAFEEGVRWVAPIQASSRRATRDLEIDG
IDIPEGVTLMTIQASANRDEDLYEDGESFNVFRKKAPHQAFGNGPHHCAGAHISRRTVGEIMLPMLFERFPGM
TLDKPEDVVWSGFGFRGPLNLPVKLN

>CYP104A7(B0E33_11740)*Labrenzia aggregate*

MAVAPVDETLTLADLTRDPYPIYKRMRAETPIVQVPAANRIFLTKAKHTKMVKDDPELYSSDDPVTPMRRAFL
AHTLMRKDGEEHRRERMAMQPAFSPKVIKTDWADLYRKIAAEYLDRLPRGETVDLFTDLCGPVAARILAHILG
LDEVGDADMQRWSQVLIDGAGNFGFADEPFARSDAANAEMDAVFDSLIARHLAEPNNSAFSVMLNARDPIPRS
QIYANIKIAIGGGINEPRDALATIIYGLLTNPDQLEEVKRQDTWADAFEEGVRWVAPIQASSRRATRDLEIDG
IDIPEGVTLMTIQASANRDEDLYEDGESFNVFRKKAPHQAFGSGPHHCAGAHISRRTVGEIMLPMLFERFSGM
TLDKPEDVVWSGFGFRGPLNLPVKLS

>CYP104A8(S58_14830)*Bradyrhizobium oligotrophicum*

MLTASLAPLDETITIADLTRDPYPIYQRLRREAPVLRVKSVGRTFLTKAADTKYVKDNAALFSSDDPNTPMKR
AFLAHTLMRKDHDEHRAERMAMMPALMPKTIEAAWEPLYRQFATDYLDRLPRGEVVDLFPALAGPLAARILAH
VMGVPDASDADMQRWSQTLIDGAGNFGWTPGPFDASDVANAEMDRCIRANMARVRAMPDSSALSFMVNAKNPI
PESQIIANIKIAIGGGINEPRDALLTILYGLLTNPEQLEAVRAQDKWRSAFEEGVRWVAPIQASSRLVMEDTE
IRGCLIPKGDTVMTIQASANRDEELFTDGESYNALRDPNPHQAFGNGPHHCAGAHLSRRTVGAILLPMLFERF
PRMELPEPAGVRWHGFGFRGPLNLPVRLQ

>CYP104A9(BBta_6721) *Bradyrhizobium* sp. BTAi1

MPIASLAPLDETITIAELTRDPYPIYQRLRRDAPVLRVKSVGRTFLTKAADTKYVKDNAALFSSDDPNTPMKR
AFLAHTLMRKDHDEHRAERMAMMPALMPKTIEQEWGPLYTTFAADYLDRLPRGEVVDLFPALAGPLAARILAH

VMGIPDASDADMQRWSQTLIDGAGNFGWTPGPFDATDIANAEMDRCIRANMARVRNEPDSSALSFMVNAKKPI
PESQIIANIKIAIGGGINEPRDALLTILYGLLTNPEQLEAVRAQDKWRSAFEEGVRWVAPIQASSRLVMEDTE
IRGCLIPKGDTMMTIQASANRDEDLFEDGETYNALRDPNPHQSFGNGPHHCAGAHLSRRTVGAILLPMLFERF
PKMTLPDPASVRWHGFGFRGPLNLPVRLQ

>CYP104A10(BRADO_1377)*Bradyrhizobium* sp. ORS 278

MLTASLAPLDETITIAELTRDPYPIYQRLRREAPVLRVKSVGRTFLTKAADTKYVKDNAALFSSDDPNTPMKR
AFLAHTLMRKDHDEHRAERMAMMPALMPKTIEAVWEPLYTKFANEYLDRLPRGETVDLFPALAGPLAARILAE
VMGVPDASDEEMQRWSQTLIDGAGNFGWTPGPFDASDRANAEMDRCIRANMERVRAEPDSSALSFMVNAKNPI
PESQIIANIKIAIGGGINEPRDALLTILYGLLTNPEQLEAVRADNTWRAAFEEGVRWVAPIQASSRLVMEDTE
IRGCLIPKGDTVMTIQASANRDEELFTDGEHYNALRDPNPHQAFGNGPHHCAGAHLSRRTVGAILLPLLFERF
PKMELVDAASVRWHGFGFRGPLGLAVRMA

>CYP104A11(BMG03_04545) *Thioclava nitratireducens*

MALAPLDEEITLAQLTTDPYPIYRRLRAESPVLRVASAGRTFLTTAELTKKVKDNPELFSSDDPNTPMKPAFR
AHTLMRKDGEEHLRERMAMAPALTPKRIQTDWGPLYEKFAEEYVGRLPRGETVDLFSELCGPLAARILAHVLG
IPEASDAQMQRWSQHLIDGAGNFGWQPEPFAISDKANDEMDALFTELREKRIAEPDGSAYSVMLNAENPIPMS
QIWANIKIAIGGGINEPRDALATIIYGLLTNPDQLEQVRAEGAWSKAFEEGVRWVAPIQVSSRLVTEDTELGG
FEIPKGDTVMTIQASANRDESIYENGEEYFVFRDKNPHQAFGNGPHHCAGAHIARRTVGKIILPLLFDRFPDM
RLPDPDAVIWRGFGFRGPINLPVELN

>CYP104A12(BF49_3851)*Bradyrhizobium* sp. BF49

MTYVTDRSSPLDETITIADLVRDPYPIYKRLRRQAPVLRVKSVGRTFLTKAADTKYVKDNPALFSSNDPNTPM
KRAFLAHTLMRKDHEEHRSERMAMMPALMPKTIESVWAALYADLAEAYLDRLPRGEVVDLFPALAGPLAARIL
AHAMGVPDASDEDMQRWSQMLIDGAGNFGWTPGPFQATDIANAEMDRCIRANAERVRAEPDSSALSFMVNAKN
PIPESQMIANVKIAIGGGINEPRDALLTVLYGLLTNPDQLEAVRAGGKWRAAFEEGIRWVAPIQASSRLVVED
TEIRGCFIPKGDTVMTIQASANRDEDVFQDGENYNALREPNPHQAFGNGPHHCAGAHLSRRTVGDILLPLLFE
RFPKMTLPDPVSVRWHGFGFRGPLNLPILLQ

>CYP104A13(BOO69_11765)*Sulfitobacter* sp. AM1-D1

MADLAPLDEKITLQELDRDPYPIYRRLRREAPVLRVKSAGRTLLTKAEDTRYVKDTPEIFSSDDPNTPMKRAF
WAHTLMRKDGEDHKRERMAMAPAFAPRVIKEDWMPQYMRIAEQYVSRLPRGEIVDLFSILSGAYAARGLAILL
GMPEASDEEMMEWSQALIEGAGNFGWADAPFERADRANTQIHALLDHIQERHRAEPDNSAFSVMLNADDPIEE
KQIYSNIKIAIGGGINEPRDSLNTVIYGLLTHPDQLAEVRRHDHWEQAFEEAVRWVAPIQVSSRLVTQDTELR
GFLIPKGDTVMTVQASANRDEDIYHDGESFNVYRDKAPHQAFGNGPHFCQGTHVARRAVGQIMLPMLFDRFPK
MSIPDRDDVIWKGFGFRGPIQIPVLLK

>CYP104A14(SAR116_2093)*Candidatus Puniceispirillum marinum*

MPLAPCDDTVTIADLTYDPYRFYKTARHHHPVVNVSSVKRTMLTKAEDTKFVKDNWQIFSSDDPSTPMKRAFQ
AHTLMRKDGAEHMRERTAMTAAFSQRNIRDVWVPLYEKIVDDYIGRLPRGETVDLFHALAAPVSARCLSHLIG
LEHASDTDLCRWSQTLIDGAGNFGWADAPFAATDRANDEINQSIQKTVDGHDANETPHAIAAMLNLEVPMDID
VIRSNIKIVIGGGINEPRDAMLTAIYGVLTNPDQLADAKSDNSLWHHAFEESVRWVAPIQVSSRRTLEDVEIR
GYLIPKGDVVMTVQASANHDEDMYEDGHLFNLHRPKQPHQAFGNGPHFCLGTHIARRTVADIVLPRLFDRFPN
LALDLGAPVLFQGFGFRGPINLPVKLH

>CYP105BF3(G432_19820)*Sphingomonas* sp. MM-1

MTTETQIQEKPVPETTFPWERGTYDPPPAYAWLREHEPVRRVVLHDGTPAWLVTRYNDVRSILADPRVSSNQN
LPGFPQIELLPRPSEEESTFLNMDAPRHTLFRRLISKHFIVKKLEVMRPRIQALVDEHIDHIIDRSEPFDFVE
EIALPVPSTVIAWLLGVPPSDHPFFNRETEALLAASLGTEEAIERATEAYANINDYVDRLIAEREKLDDPGDD
ILGDLVRASREGQIERRDVLNTAWLLLVAGHDTTANMIGLGMLTLLEHPDQLAQLQAEPALIPDAIEELLRYL
TVVHLIILRIATEDIEIGGVTIPAGEGIIPLNFAANRDDGHFPDAAKFDIRRRPRDHVAFGYGVHQCIGQALA
RIELQIVFETLLRRIPNVRLATDPADIQFKSHASINGIARLPVAL

>CYP105BF3(K663_19828)*Sphingobium* sp. MI1205

MTTETQIQEKPVPETTFPWERGTYDPPPAYAWLREHEPVRRVVLHDGTPAWLVTRYNDVRSILADPRVSSNQN
LPGFPQIELLPRPSEEESTFLNMDAPRHTLFRRLISKHFIVKKLEVMRPRIQALVDEHIDHIIDRSEPFDFVE
EIALPVPSTVIAWLLGVPPSDHPFFNRETEALLAASLGTEEAIERATEAYANINDYVDRLIAEREKLDDPGDD
ILGDLVRASREGQIERRDVLNTAWLLLVAGHDTTANMIGLGMLTLLEHPDQLAQLQAEPALIPDAIEELLRYL
TVVHLIILRIATEDIEIGGVTIPAGEGIIPLNFAANRDDGHFPDAAKFDIRRRPRDHVAFGYGVHQCIGQALA
RIELQIVFETLLRRIPNVRLATDPADIQFKSHASINGIARLPVAL

>CYP105BF3(SJA_C2-04970)*Sphingobium japonicum*

MTTETQIQEKPVPETTFPWERGTYDPPPAYAWLREHEPVRRVVLHDGTPAWLVTRYNDVRSILADPRVSSNQN
LPGFPQIELVPRPSEEESTFLNMDAPRHTLFRRLISKHFIVKKLEVMRPRIQALVDEHIDHIIDRSEPFDFVE
EIALPVPSTVIAWLLGVPPSDHPFFNRETEALLAASLGTEEAIERATEAYANINDYVDRLIAEREKLDDPGDD
ILGDLVRASREGQIERRDVLNTAWLLLVAGHDTTANMIGLGMLTLLEHPDQLAQLQAEPALIPDAIEELLRYL
TVVHLIILRIATEDIEIGGVTIPAGEGIIPLNFAANRDDGHFPDAAKFDIRRRPRDHVAFGYGVHQCIGQALA
RIELQIVFETLLRRIPNVRLATDPADIQFKSHASINGIARLPVAL

>CYP105CX1(LH19_26270)*Sphingopyxis macrogoltabida* 203

MSNTATELPYFPMPRDSKCPFSPPPELKRIQQETPVSKVRIWDGREAWLVTRYDDVRFVLRDQRFSVNPHHPG
WPPMSAGQGATRRDLKPNIFALDNPVHNELRRMQSADFRIAQIEGWRPRVQQLVDEAIDAMLEMPKPVDLWEA
FALKIPSIVICELLGVAYEDSEFFQHHSDRLVKTGSTSEEIGESMDALRGFLRDLVEKKVVTPTEDILSRMAT
QQVKEGLLTASEVADLCVMLLIAGHDTTSNTICLATILLLQHPDQLEKFRNGDDEVVAKGVEEILRFLDVPHL
GRRRVALEDVEVGGQLIRAGEGVIAAQMIADRDETAFEDPDKFDIDRDARHHLGFGFGTHQCLGQPLVRVEVQ
IALATLFRRIPTLQLAVPMEEIRFDYDALVYGVHELPMTW

>CYP107AN1(bll_6537)*Bradyrhizobium diazoefficiens* USDA 110

MVTPGSGAAIGVFVSCGNRFEVTMNEQAQPAGGDPLFNPLSPDFIRNPYPHYDRLRAIDPIHVTPFGQFVASR
HADVSLVMRDKRFGKDFVERSKRRYSEKIMDEPVFRSMSHWMLQADPPDHTRLRGLVVKAFTARRVEDMRPRI
QEIVDEAIDAVIDRGHMDLIEDFAFRLPVTIICDMLGIPEDHREVFYKSSRDGGRLLDPVPLTPEEIAKGNAG
NMMAQMYFQQLFELRRRNPADDLTTQLVQAEEDGNKLTNEELTANIILLFGAGHETTVNLIGNGLLALHRNPD
QLALLKARPELMVNAIEEFLRYDSSVQMTGRVTLEDIDDLGGRKIPKGETVLCLLGSANRDPAVYPDRPDRLD
VTRPNVKPLSFGGGIHFCLGAQLARIEAEIAIATLLRRLPDLRIDDVENPEWRPTFVLRGLKSLPASW

>CYP107AN1(BJ6T_28960)*Bradyrhizobium japonicum* USDA 6

MNEQVQPAGGPLFNPLSPDFIRDPYPHYDRLRTIEPIHVTPFGQFVTSRHADVSLVMRDKRFGKDFVERSKRR
YSPAIMDEPVFRSMSHWMLQSDPPDHTRLRGLVVKAFTARRVEDMRPRIQAIVDQAIDAVIERGHMDLIEDFA
FRLPVTIICDMLGIPEDHRETFYNSSRDGGRLLDPVPLTPEEIAKGNAGNMMAQMYFQQLFELRRRNPGDDLT
TQLVQAEEDGNKLTNEELTANIILLFGAGHETTVNLIGNGLLALHRNPDQLALLKARPELMVNAIEEFLRYDS
SVQMTGRVALEDIADLGGKSIPKGETVLCLLGSANRDPAVYPDRPDRLDVTRPNVKPLSFGGGIHFCLGAQLA
RIEAEIAIATLLRRLPDLRIDDVENPEWRPTFVLRGLKRLPASW

>CYP107AN1(RN69_14145)*Bradyrhizobium japonicum* E109

MNEQVQPAGGPLFNPLSPDFIRDPYPHYDRLRTIEPIHVTPFGQFVTSRHADVSLVMRDKRFGKDFVERSKRR
YSPAIMDEPVFRSMSHWMLQSDPPDHTRLRGLVVKAFTARRVEDMRPRIQAIVDQAIDAVIERGHMDLIEDFA
FRLPVTIICDMLGIPEDHRETFYNSSRDGGRLLDPVPLTPEEIAKGNAGNMMAQMYFQQLFELRRRNPGDDLT
TQLVQAEEDGNKLTNEELTANIILLFGAGHETTVNLIGNGLLALHRNPDQLALLKARPELMVNAIEEFLRYDS
SVQMTGRVALEDIADLGGKSIPKGETVLCLLGSANRDPAVYPDRPDRLDVTRPNVKPLSFGGGIHFCLGAQLA
RIEAEIAIATLLRRLPDLRIDDVENPEWRPTFVLRGLKRLPASW

>CYP107AN1(BF49_5136)*Bradyrhizobium* sp. BF49

MNEHVQGTGGPLFNPLSPDFIRDPYPHYERLRAIFPIHVTPFGQFVTSRHADVSLVMRDKRFGKDFVERSKRR
YSEKIMEEPVFRSMSHWMLQADPPDHTRLRGLVVKAFTARRVEDMRPRIQEIVDQTIDAVVDRGHMDLIEDFA
FRLPVTIICDMLGIPEDHRETFYNSSRDGGRLLDPVPLSPEEIAKGNAGNMMAQMYFQQLFELRRKNPGDDLI
TQLVQAEEDGNKLTNEELTANIILLFGAGHETTVNLIGNGLLALHRNPDQLALLKARPELMVGAIEEFLRYDS
SVQMTGRVALEDIDDLGGAKIPKGETVLCLLGSANRDPAVYPDRPDRLDVTRQNVRPLSFGGGIHFCLGAQLA
RLEAEIAIATLLRRLPDLRIDDVENPEWRPTFVLRGLKRLPASW

>CYP107AN1(S23_17940)*Bradyrhizobium* sp. S23321

MNEQAQPAGGDPLFNPLAPDFIRDPYPHYDRLRTLDPIHVTPFGQFVASRHADVSLVMRDKRFGKDFVERSKR
RYSPAIMDEPVFRSMSHWMLQADPPDHTRLRGLVVKAFTARRAEDMRPRIQEIVDQAIDAVINRGHMDLIEDF
AFRLPVTIICDMLGIPEDHREVFYKSSRDGGRLLDPVPLSKEEIAKGNAGNMMAQMYFQQLFELRRKTPGDDL
ITQLVQAEEDGNKLSNEELTANIILLFGAGHETTVNLIGNGLLALHRNPDQLALLKARPELMVNAIEEFLRYD
SSVQMTGRVALEDIDDLGGKKIPKGETVLCLLGSANRDPAVYPDHPDRLDVLRQNVRPLSFGGGIHFCLGAQL
ARIEAEIAIATLLRRLPDLHIDDVENPKWRSTFVLRGLTELPASW

>CYP107AN2(BRADO_5601)*Bradyrhizobium* sp. ORS 278

MSELWERDHMNEHVQAAKAAPLFNPLSPEFIRNPYPFYQQLRDNDPVHVTPFGSFLASRHAESSLVLRDKRFG
KDFVARSIRRYGPEIMNEPIFRSMSHWMLQQDPPDHTRLRGLVVKAFTARRVEDMRPRIQAIVDSTLDEIIPR
GRMDLIEDFAFKLPVTVICDMLGIPEEHREAFYKSSREGGRLLEPVPLSKAEIEEGNAGNAVSRAYFQHLFEL
RRKQPGDDLTTQLLQAEEDGAKLSHEELTANIILLFGAGHETTVNLIGNGLLALYRNPDQLALLKARPELMTN
AIEEFLRYDSSVQLTGRVALEDIEDLGGRRIPAGETVLCLLGSANRDPAVYPDNPEQLDITRANVKPLSFGGG
IHHCLGAQLARIEAEVAIGTLLRRLPELKLDDPDNPEWRPTFVLRGLKRLPAHW

>CYP107AN2(S58_20060)*Bradyrhizobium oligotrophicum*

MNEHVQANKAAPLFNPLSPEFIRDPYPFYAQLRDNDPMHVTPFGAFLASRHAESSLVLRDKRFGKDFVARSIR
RYGPDIMNEPVFQSMSHWMLQQDPPDHTRLRGLVVKAFTARRVEDMRPRIQEIVDRTLDEVIPQGRMDLIEDF
AFKLPVTVICDMLGIPEEHREAFYKSSREGGRLLEPVPLSKAEIAEGNAGNAVSKAYFQHLFELRRKQPGDDL
TTQLLQAEEDGAKLSHDELTANIILLFGAGHETTVNLIGNGLLALHRNPDQLALLKARPELITNAIEEFLRYD
SSVQLTGRVALEDIEDLGGRRIPKGETVLCLLGSANRDPAVYPDDPEKLDIVRANVKPLSFGGGIHHCLGAQL
ARIEAEVAIGTLLRRLPELKLDDPDSPEWRPTFVLRGLKRLPAHW

>CYP107AN2(BBta_6122)*Bradyrhizobium* sp. BTAi1

MNELVQAAKAAPLFNPLSPEFIRDPYPFYQQLRDNDPMHVTPFGSVLASRHAEASLVLRDKRFGKDFVDRSIR
RYGPEIMQEPVFRSMSHWMLQQDPPDHTRLRGLVVKAFTARRVEDMRPRIQQIVDRTLDEVIPHGRMDLIEDF
AFKLPVTVICDMLGIPEEHREAFYRSSREGGRLLDPVPLSKAEIAEGNAGNAVSTAYFQHLFELRRKHPGDDL
TTQLLQAEEDGAKLSHEELTANIILLFGAGHETTVNLIGNGLLALHRNPDQLALLKARPELITNAIEEFLRYD
SSVQLTGRVALEDIEDLGGRRIPKGEAVLCLLGSANRDPAVYPDAPEQLDITRANVKPLSFGGGIHHCLGAQL
ARIEAEVAIGTLLRRLPELKLDDPDNPEWRPTFVLRGLKRLPAHW

>CYP107JE1(NT26_0569)*Rhizobium* sp. NT-26

MRGRSEADALPLEAPLDLLDPSFVCDPYTIYRRLRTHDPVHRSRNGGWILTRHRDIVSALGDPSLGNAPSRYS
VVAPRNRGRYVCANVAQNILPFLDKPEHVRPRRILSRVLSTHMKDNPLDIAQIARQLLDPQLARRQMDAIADF
GTPLSAEVMCRMLGIPAPDRDRMLEYSHYFFFLFAPIPSDQIRQKADEALTAFRGYFADLVQERRRKPQADLI
SLLLSAEDEGERLSDCQLVDNCMLLFSDGVENVDAGIANILLALGRHPAEMKRLRDAPELIANAVAEGLRYDS
PAQLIARVARDDVEIGGQTIKRDSTVFLALGSANRDPAVFADPDRFNISRNTSPLLSFGKGGHSCIGASLVRS
QSVEAVRSLLQTTTVVEVDQDGLEWLPRVGHRWLKTLPIRLFPS

>CYP107-fragment(TMO_3392)*Tistrella mobilis* KA081020-065

MGIAGERFEDLHPLLAAITRGHDMAPTEADRMRGRFAQQTMGRWLAPRVLAAPEGSLAAAVRDIAAGRNDDGR
MVVGWAAMLVYSGSTTIRDFLVNVVALLVDRPDVAARLAAEPAILGTMVEECLRLEGPVRGTGRVAAADVVIG
ETGIPAGSVLHLMFDHANRDPERFSDPDVFDPVRSPNPHLSFGHGNTFCLGAHLARLETRALILRILPHLHRL
RRTHPPIWTSVRLLREQARLTLSCH

>CYP108A3(BSY17_2029)*Sphingobium* sp. RAC03

MATSLDADVRPIIPDDIARIVIAPKSYTDDATIYGAFRWLREHMPLGIAQVPGYDPVWVVTKHADIKEVERNG
RLFHNADHNPILVDQASDAFTRDINGGSIRILSSLTYMDPPEHASYRTLTSNWFLPGKISKLEDKIRVLARQS
VDNLLSFDGECDFVRDFALHYPLRVIMTLFGVPPEDEPRMLKLTQEFFGVHDPEEQRPEMAADPVVAAKQWAA
SIEDFYTYFDKLSADRRERPTDDLLSLIANSRINGAPIPRDEANGYYVAIATAGHDTTSSSTAGGLHGMMVYP
ENWAKVKADPSLIPGLVDEAIRWTSPVKHFMRNATADTQVRGMDIRAMERLMICYPSGNRDEAVFDDADRFDI
TRSPNPHIAFGFGPHMCLGQHLAKLEMRILFEELLPHLSSVEMAGDPRMVETNFVGGYKALPIRFTKN

>CYP108A4(WYH_03331)*Altererythrobacter atlanticus*

MASATQTASGPLIPQHIAETVFSAKSYTDDAVIYDAFKWLRENMPLGIAEVEGFDPVWVVTRHADIRQVEKDA
KLFHNADFNPILNDRASDAFTREMTGGSVSGLASLTYMDPPVHQQYRMLTANWFLPGKISKLEDQIRVLARQS
VEHFLSFDGECDFVRDFALHYPLRVIMTLFGVPPEDEPKMLKLTQEFFGGHDPEEQRAELREDPIVAAKMWAA
TLEDFFQYFDALAEKRRAEPQDDLLTLIANSRINGSPIGRDEANGYFVAIATAGHDTTSSSVAGGMHGMIAFP
ENFAKVKADPALIPGLVDEAIRWTSPVKHFMRNATQDTEVGGQLIRSMDRLMICYPSGNRDEAVFEHPDRFDI
TRSPNRHVAFGFGPHMCLGQHLAKLEMRILFEELLPHLAAVELAGSPKLVETNFVGGYKSLPIRFRKA

>CYP108D1(Saro_3162)*Novosphingobium aromaticivorans*

MDSIPMVPAEVGRAVIDPKSYGTWEPLLDRFDALRAEAPVAKVVAPDDEHEPFWLVSSFDGVMKASKDNATFL
NNPKSTVFTLRVGEMMAKAITGGSPHLVESLVQMDAPKHPKLRRLTQDWFMPKNLARLDGEIRKIANEAIDRM
LGAGEEGDFMALVAAPYPLHVVMQILGVPPEDEPKMLFLTQQMFGGQDEDMNKSGLKDLPPEQISQIVAGAVA
EFERYFAGLAAERRRNPTDDVATVIANAVVDGEPMSDRDTAGYYIITASAGHDTTSASSAGAALALARDPDLF
ARVKADRNLLPGIVEEAIRWTTPVQHFMRTAATDTELCGQKIAAGDWLMLNYVAANHDPAQFPEPRKFDPTRP
ANRHLAFGAGSHQCLGLHLARLEMRVLLDVLLDRVDSLELAGEPKRVNSTFVGGFKSLPMRWKAA

>CYP108D4(Ga0102493_11805)*Erythrobacter litoralis* DSM 8509

MASAGDTKQPAALPPETAREVIDPKAYAEWDGLLDTFDRLREEAPVAKVMPETEGLFEPFWLVTRYDDVMRIS
KDNAAFLNNPRPVVFSFNQAIEFSRAATGSDMLVDSLVVFDAPIHPKYRRLTQDWFMPRNLGRIEEEIRALAH
RTVDAMIEKGGEIDFVKEVSGPYPLRVVMQILGVPPEDEPRMQMLTQQLFGGQDADLSGTGMENMTPEQVVQL
VAGAVKSFEDYFAKLAEDRRAHPTEDVASVIANATVDGEPLPPRDMAGYYIIVATAGHDTTSASTAGAMQALA
NDPEQWERVKADRSLLPGIVEEAIRWTTPVQHFMRTAAEDCEVGGQQVKKGDWLMINYVAANHDPAQFDNPRK
FDAARSPNRHLAFGAGAHQCLGLHLARLEMRLLFEALLDKVASVAPAGEAKRASSTFVGGLKTLPLRITPE

>CYP108D5(BG023_11389)*Porphyrobacter* sp. LM 6

MSGEAVLPGDLARRVIDPHAYAQWDGLLDTFDHIRAETPVVKVQPDTPGLFDPFWLVTRYDDVMRISKDNATF
LNNPRPVVFSFNQAIEFSRAATGSNMLVDSLVVFDAPIHPKYRKLTQEWFMPRNLARLEDELRALAARTVDRM
LEQAKTSGDEVDFVKEVSGPYPLRVVMQILGVPPEDEFRMQMLTQQLFGGQDKDLSGSGMDQMTPEQVVQIVA
GAVKTFEDYFAGIAEDRRKNPTEDVASVIANALVDGQPLPRATWRDITSSLPPPGMTPPRQAPQARCRRSPAT
PNNTPASAPIARCFPALSRRRSAGPAPCSTSCAPRPRTSNWAGSR

>CYP108D7(AMC99_02398)*Altererythrobacter epoxidivorans*

MSGEAILPPDVAREVIDPHAYAEWNGLLDTFDRLRQESPVALVKPEDGELFDPFWLVTRYDDVMRLSKDNKTF
LNNPRPVVFSFRQAIEFSRQATGSDMLVDSLVVFDAPIHPKYRRLTQDWFMPRNLAKMEAEIRNIAEATVDRL
VAAGSEADFVKLVSGPYPLHVVMQILGVPEEDEPRMQMLTQQLFGGQDADLSGTGMENMSPEQVVQLVAGAVK
TFEGYFAELAAQRRANPTDDVASVIANATVDGEPLPERDMAGYYIIVATAGHDTTSASTAGAMMALAQDPEQW
ARVRADRSLLPGIVEEAIRWTTPVQHFMRTAAEDVEVGGQQVKKGDWLMLNYVSANHDPSQFDDPRRFDAARS
PNRHLAFGAGAHQCLGLHLARMEMKILFETLLDRIETIELAGEPKRATSTFVGGLKTLPVRFKAA

>CYP108D8(ELI_12320)*Erythrobacter litoralis* HTCC2594

MMASGTPILPPDTAREVIDPHSYAEWGSLLDTFDRLRAESPVAKIVPDEEGLFEPFWLVTRYEDVMRMSKDNR
TFLNNPRPVVFSLNSAIEFSRAATGSDMLVDSLVVFDAPVHPKYRRLTQDWFMPRNLAKIEGEIRIIANRVVD

KLLEAGPELDFCKLVSAPYPLHVVMQILGVPEEDEPRMLMLTQQMFGGQDADLSGTGMENMTPEQVLQLVSGA
VKSFEDYFAEIANQRRENPTDDVASVIANATVDGEPLPDRDMAGYYIIVATAGHDTTSASTAGAMQALAQDPE
QWAKVKADRSLLPGIVEEAIRWTTPVQHFMRTAAEDTELGNQAIKKGDWLMMSYVAANHDPAQFENPRKFDAA
RSPNRHLAFGAGAHQCLGLHLARLEMRILFEALLDRVETVELAGEPKRSTSTFVGGLKTLPLRITPA

>CYP108D9(BSY18_1523)*Blastomonas* sp. RAC04

MATIAPATLPSEIASAIVNPVAYGQWDDLKTKFRWARDNMPVGLVQAEGYMPFWAITRHEDIMTVSKDNARFL
NAPKSVVLGPVAVQMLTHMITGGSPHLVRSLVTMDAPEHMDYRKLTQSWFMPKNLAGSEARIREIARASIDAM
VATGGACDFVHQVSALYPLHVVMQILGVPHEDEPLMLKLTQEMFGGEDPDLNRARSVDLTPEQVMQFVVDAVR
DFEGYFMKLAADRRASPRDDVASVIANATIDGEPISDRNAAGYYIIVAAAGHDTTSASTAGAMWALANDPEQF
ARIKADRSLLPGLIEEAIRWTTPVQHFMRTAAEDCEIGGQAIAKDDWLMLCYVSGNHDERVFDDPDRFDAARG
PNRHVAFGAGVHQCLGLHLARMEMRILFEELLDRVESVELAGTPQRASSTFVGGPKTLPIRFVAS

>CYP108G1(CC_2494) *Caulobacter vibrioides* CB15

MTISTDIANTIIDPKAYADGDRIDQAFAHLRREAPLAVAQPDGFDPFWVVTRHADILEVERQNELFHNGDRAT
VVTTIEPDKKVREMMGGSPHLVRSLVQMDNPDHFAYRKITQGALLPQNLRALEARIREIARGFVDRMAEHGDR
CDFARDVAFLYPLHVIMEVLGVPESDEPRMLKLTQELFGNADPDLNRTGKSVTDVGEGVDSIQSVVMDFMMYF
NAITEDRRANPRDDLATLIANGKINGEPMGHLEAMSYYIIAATAGHDTTSSTTAGALWALAENPDQFAKVKAD
PSLIPGLIEESIRWVTPVKHFMRTATADAELGGQKIAKGDWIMLSYPSGNRDEAVFEDPFTFRVDRTPNKHVA
FGYGAHICLGQHLARMEMRVLWEELFARLDHVELDGAPTRMVANFVCGPKSVPIRFKMH

>CYP108G1(CCNA_02579) *Caulobacter vibrioides* NA1000

MTISTDIANTIIDPKAYADGDRIDQAFAHLRREAPLAVAQPDGFDPFWVVTRHADILEVERQNELFHNGDRAT
VVTTIEADKKVREMMGGSPHLVRSLVQMDNPDHFAYRKITQGALLPQNLRALEARIREIARGFVDRMAEHGDR
CDFARDVAFLYPLHVIMEVLGVPESDEPRMLKLTQELFGNADPDLNRTGKSVTDVGEGVDSIQSVVMDFMMYF
NAITEDRRANPRDDLATLIANGKINGEPMGHLEAMSYYIIAATAGHDTTSSTTAGALWALAENPDQFAKVKAD
PSLIPGLIEESIRWVTPVKHFMRTATADAELGGQKIAKGDWIMLSYPSGNRDEAVFEDPFTFRVDRTPNKHVA
FGYGAHICLGQHLARMEMRVLWEELFARLDHVELDGAPTRMVANFVCGPKSVPIRFKMH

>CYP108G3(Plav_1544)*Parvibaculum lavamentivorans*

MTDKTIDNAIVNPKTYAHVDEFHRLFTQLRKEEPVRWTEPDGFRPFWTVSKHADIMEVERQNDKFLNDPRLTL
QTIEVEEEVKKFTGGNSKLIRSLVDMDNPDHRNYRGLTQAWFMPPNLKAISARVEALAEKYIDRLEAKGGECD
FVSDVAVWYPLRVIMTVLGVPAEDEPIMLKLTQELFGSTDPDMKRPDATETVNTVTEFFNYFTAMTEDRRKNP
KDDVASVIANATIDGEPIGHLEAISYYIIVATAGHDTTSSTAAGGLLALMQNPEEFAKLKANPEGLLGGAIDE
MIRWTTPVKHFFRTAAVDYELRGQKIKAGDNLLMCYWSANRDEEAFDDPFSFKIERSPNKHLAFGYGAHLCLG
QHLAKMEIRALYKELLARLDHIELAGDPAWVEASFVSGLKRLPIRYSMKRKAA

>CYP108G6(Cseg_1214)*Caulobacter segnis*

MTIPADIAKDIVDPTAYADGDRVDQAFAWLRREAPLDIAQPEGFDPFWVVTRHADILEVERQNELFHNGDRAT
VLTTIEADRKVREMMGGSPHLVRSLVQMDNPDHFAYRKVTQGALLPQNLRALEARIREIARGFVDRMAEHGDR
CDFARDVAFLYPLHVIMEVLGVPESDEPRMLKLTQELFGNADPDLNRSGKTITDAGEGVDSIQSVVMDFMMYF
NAITEDRRAKPRDDLASLIANGKINGEPMGHLEAMSYYIIAATAGHDTTSSTTAGALWALAENPEQFAKVKAD
PSLISGLIEESIRWVTPVKHFMRTATADAELAGKKIAKGDWLFLSYPSGNRDETVFEDPFSFKVDRAPNKHLA
FGYGAHICLGQHLARMEMRVLWEELLSRLDSVELDGTPTRMTANFVCGPKAVPIRFKMH

>CYP108G7(Caul_3446)*Caulobacter* sp. K31

MTIPMDIANTIVDPTAYADGDRIDKAFAQLRRESPLEVAQPEGFDPFWVVTRHADILEVERQNELFHNGDRAT
IVTNIEADKKVREMMGGSPHLVRSLVQMDNPDHFAYRRVTQGSLLPQNLRTLEARIREIARGFVDRMAEQGDR
CDFARDVAFLYPLHVIMEVLGVPEADEPRMLKLTQELFGNADPDLNRAGKTFTDISEGLDSLQSVVMDFMMYF
NAMTEDRRANPREDLASIIANGKIDGQPMGHLEAMGYYIIAATAGHDTTSSTTAGALWALAESPDQFARLKAD
PGLIPGLVEESIRWVTPVKHFMRTATADAELAGRKIAKGDWMMLSYPSGNRDEAVFEDPFSFRIDRTPNKHVA
FGYGAHVCLGQHLARMEMRVLWEELFARLDSVELDGQPTRMTANFVCGPKSVPIRFKMN

>CYP108G8(PHZ_c0168)*Phenylobacterium zucineum*

MIPQDVANTIVDPKAYAAWTPIEEAFGYLRREAPLEQAQPEGFDPFWVVTRQDDIRAVELQNELFHNGDRSTV
VTTIEADKKVRALMGGSPHLVRSLVQMDNPDHGNYRRLTQGWFQPQNLRALEERIRVIARGFVDRMAAHGGRC
DFAKDVAFLYPLHVIMEVLGVPEIDEPRMLKLTQELFGNADPELNRSAGQLQDVDQALLALQGTIADFIAYFN
EMTEDRRAKPRQDVASIIANGMIEGKPLGHLEAMSYYIIVATAGHDTTSSTTASALWALAENPAQFRAVKDDL
SLVKGLIEESIRWETPVKHFMRTATDDAELAGKKIAKGDWLFLSYPSGNRDEAVFDNPYAFDVTRSPNKHVAF
GYGAHVCLGQHLARMEMRILWEELLPRLESVELDGQPRRMEASFVCGPKSVPVRFKMN

>CYP108G9(HNE_0569)*Hyphomonas neptunium* ATCC 15444

MAIPEHIAREVIDPKAYADGKRVDDAFTWLRANAPLDVATVEGFDPFWVVTRHADILNIERQNDLFHNEDRSA
TLTTIESDKRVREMMGGSPNILRSLVQMDNPDHMQYRRLTQAWFMPQNLRKLEDRVREIARGFIDQMAAKGTE
CDFARDVAFLYPLHVIMEVLGVPPIDEPRMLKLTQELFGTQDPDVNRAGKEVGTTDDALKMINETIFDFMTYF
NAMTEDRRQNPREDLASVIANGAVYGQPLGHLEAMSYYIIAATAGHDTTSNTTAGAMWALAENPDQFRKVKDD
LSLIPSMVEESIRWETPVKHFMRTATDDTEVAGQKIAKNDWLFLAYPSGNRDEAVFDDPFTFKVDRSPNKHVA
FGYGAHVCIGQHLARMEMRVLWEELLPRLHSIEMAGKPQRTQANFVSGPKSVPIRFKMS

>CYP108G10(BBta_7865)*Bradyrhizobium* sp. BTAi1

MGAAASADEAIPQDIAAALVDPACYADHRIHDAYRWLRANNPLGRAQLDAFDPFWVVTRHADILAISRQNDLF
HNADRATTLTNKAVLERVHKITGQPNLVRSLVQMDAPDHPKYRALTQGWFMPANIAKFEPRIREIARATVDRM
AAKAAANGGVCDFVADVALGYPLHVIMEILGVPEADEPRMLKLTQELFGPQDPDTARVREALSAEVFAAMLQA
VVADFAAYFQRITEDRRAHPRDDLATVIANAKINGEDMPLHDRTSYYMIVATAGHDTTSSSTAGAIWALAEDP
AQFARVKADPELIPGLVDEAIRWMTPVKHFMRSATADTELGGRRIAKGDWLMLCYASGNRDEAVFEEADRFRC
DRRPNRHVAFGYGAHLCLGQYLAKLEMRILFEELLPRLSALSLAGPVKMTQATFVNGPKTLPIRVTMA

>CYP108G11(S58_72760)*Bradyrhizobium oligotrophicum*

MGAAAQAEETIPAHVATALVDAACYADHRIHDAYRWLRANNPLGRVRLEAFDPFWVVTRHADILSISRQNDLF
HNADRATTLTNKAVLERVHKITGQPNLVRSLVQMDAPDHPKYRALTQGWFMPANIARFEPRIREIARATVDRM
AAKAAANGGVCDFVEDVALGYPLHVIMEILGVPEEDEPRMLKLTQELFGPQDPDTARVREALSAELFAAMLQA
VVADFAAYFQRITEDRKANPREDLATVIANAKINGEDMPLHDRTSYYMIVATAGHDTTSSSTAGAIWALAEDP
EQFARVKAEPGLIPGLVDEAIRWMTPVKHFMRSATADTELGGRHIARGDWLMLCYASGNRDEAVFDEPDAFRC
DRKPNRHVAFGYGAHLCLGQHLARLEMRILFEELLQRLSSLAPVGPVKMTQATFVNGPKKLPIRVEMS

>CYP108G12(AEM38_10480)*Hyphomonadaceae bacterium* UKL13-1

MSADPTLPEELSRAVVSPKTYADQAVMDATFAKIRTDYPLAKTQMSDFEPFWFVSKHADILEISRQNDLFLSG
ELPTTLTTKSVDEKVRKQTGGSPHLIRTLVQMDAPDHPKFRVMTQAWFMPQNLRKLEDQIRKIARMYVDKMAG
MDGQCDFLNDIALFFPLRVIMEILGVPEVDEPRMLKLTQELFGAQDPEMNRSGGELQDEQKALADIQAVLMDF
FMYFNQITEARRANPTEDVATVIANAKMDGAPIGAFEAVSYYTIIATAGHDTTSSSTAAGLFGLIQNPDQLKA
VQQDPNLIHGLVDESIRWATPVRHFMRSATADYALRGQTIKKGDWLMLSYPSGNRDEEVFDDPYAFRIDRTPN
KHLAFGYGAHLCLGQHLAKLEMRIFWEELLPRLKSVEFAGEPKLSEANFVGGLKNLPIRYVMD

>CYP108G13(BRADO_7134)*Bradyrhizobium* sp. ORS 278

MSAALADEVIPADIAVTLVDPACYADHRIHDAYRWLRANNPLGRAELETFDPFWVVTRHADILAISRQNELFH
NADRATTLTNKAVLERVHKITGQPNLVRSLVQMDAPDHPKYRALTQGWFMPANIAKFEPRIREIARATVDRMA
AKAAAHGGACDFVADVALGYPLHVIMEILGVPEEDEPRMLKLTQELFGPQDPDTARVREGLSAEVFAAMLQAV
VADFAAYFERITADRRARPRDDLATVIANARINGEEMPLHDRTSYYMIVATAGHDTTSSSTAGRDLGAGAGSR
AVRAREGRCRTDSGTGRRGDPLDDAGQAFHAQRDRRHRARRPADREGRLADAVLRLGKSRRGGV

>CYP108G14(HNE_2207)*Hyphomonas neptunium* ATCC 15444

MDHSHEASGPDLPDWVSQRLVSPKAYATDQIDEAYRWARTNNPLGVARAEGYDPFWVLTKHDDILTVSRNNSL
FHSADRATTLLNQAAIERTTRITGGSPNLVRSLVQMDAPDHMKYRTLTQGWFMPANLRKREEEVGQIADAAVA

SFLKQNGRCDFVRDVALNYPLHVVMNILGVPPEDFPRMLRLTQELFGSQDPDTARQLDALSAEQFGAMIQAVV
QDFAVYFNAISEDRRRDPKDDLATIIANATIDGRPIGPMEATSYYIIVATAGHDTTSSATSMAMWALATQAGL
LQRLQAQPESIPGFVEEAIRWATPVKTFMRSATKDTELRGRRIAKGDWLMLCYASGNRDEEVFSDPYTFDIDR
KPNRQLAFGNGAHVCIGQHLARLEMRVLFEKLIPALKSVSLDGDIKFVESYFVSGPKTLPIRFEVM

>CYP108G15(S58_21260)*Bradyrhizobium oligotrophicum*

MVGRIDDQVVDPATYGTPHKYDEIFAELRRSDPVRWTEPAGYRPFWTIAKHSDILEIERQNDKFINDPRTSLV
PIEEEERRVRAGNAKLVRTLITMDEPDHRAYRGITQAWFMPVNLRKIEGEIKNLAGEFVALMDERGGRCDFVN
DVALWYPLRIIMTILGVPREDDAKMLKFTQALLASSDKDLSGGSNNFQSRQVAAQEFFAYFGAIAEDRRRNPK
DDLASIIANASVGGNPAGVFESLSYYLVIATAGHDTTSSAMAGGLHALIEHPAEFRRLRESPELLNSAVEEML
RWTSPVKHFFRTATQDYQLRGKQIRAGDSLMMCFPSGNRDEEVFVDPFAFRVDRTPNRHIAFGHGVHQCLGLN
LARMEMKALFTEIVARVDSIELAGEPAWIEANLVSGPKRLPITYRMKSCPVQ

>CYP108G16(LH19_04405)*Sphingopyxis macrogoltabida* 203

MSDTLSGNIDEIIVDPKTYGDEHSYHRAFDRLRRDDPVHWTEPKDYRPFWAVTRHADIMAVELNAKNFLNDPR
QFLVTRADEDLLLEQTGSHKFAHNLVAMDDPEHRAFRSLTSAWFGAKSIRGLEEEIAALARETVDRMVEMGGS
CDFAAEIAAWFPLRTIMIVLGVPREDEPLMLHLSQKLFGSVGGVDGMAGMVDAFNAFNDYFGKVTEDRRRNPR
GDVATILATATIDGEPIGEAERNAYYLIVAAAGHDTTSSSISGGLLALIQNPDQMAKLRADPALIGTAVDEFI
RWTSPVKHFFRTAVADCEVGGKQIRAGDNLMMCYPSGNRDASVFDDPYAFRVDRPDARKHLSFGYGPHLCLGN
ALAKLEIRILFTEMLRRIDDFELAGDPAWVEASFVSGLKHLPIRYKVRETGA

>CYP108G17(LH19_15640)*Sphingopyxis macrogoltabida* 203

MANMLADNIDEIITDPATYADEALYHQTFAELRRTDPVHWCNPAQYHPFWAVTRHADIMRVELDAANFLNEPR
QFLVTIDDQKMMEEQTGESNFARNLVAMDNPDHKAYRALTADWFGAKSVRALEEKITELARETVDRMVAKGGT
CDFAKEIAAWYPLRTIMIVLGVPREDEPLMLELSQKVFGGSDADTGGGEGMTSMLEAFAAFNDYFGRVVADRL
VNPQDDVATLLSTATIDGAPIGEAERNAYFLIIAAAGHDTTSSAISGGLLALIRNPDQMAKLRANPDLMPQAV
DEFIRWTTPVKHFFRTAVADCEVGGQAVKAGDSLMMCYPSANRDEAAFDDPMEFRIDRKPNRQLAFGYGPHVC
LGQFLAKMEIRILFTELLARIEEIELAGEPAWVKANFVSGLKRLPIRYRCEGAASVTGG

>CYP108G18(JI59_08785)*Novosphingobium pentaromativorans*

MTVLSEIGPMLVDPKAYADGSIYEAYRWLRRNDPLASIEVEGYQPFRVVTLYSDVQYVSRHNALFHSSPNPTL
SPISRMKLVRELTGSTSPIKSLVDMDPPEHGKYRALTSPWFQPGNLKSLEGRIRDIAREMVERMVDARCQCDF
ARLVAHEFPLQVILEILGLPREDHPVMLRLTQEMFGATDPDVLGNRSATNDSASASDLGAVREMMAYFDKLTE
DRRAHPTSDLASVIANARIDGEPMGHLETMGYYVITATAGHDTTSSSIGGAMWALAEHPDQFARLKADMSLLP
TMIDEAIRWTTPVKHFMRTATADTEIGGRPVREGERLMLCYGSANRDESVFDDPDTFRLDRKPNRHLAFGYGG
HLCLGQYLARIEMRILFEELLPRLSSLALDGEPAMTEAIFVSGPKRLPIRYEIAPPVGAPRVADAEPNCPA

>CYP108G19(S58_21370)*Bradyrhizobium oligotrophicum*

MKEGPGLSAANFPIPDNIVAVLTDPAAYGTQRIFDAYRWLRVNQPLGKASPRGFNPFWVVTRHADIQTISQQS
QLFRSSDRPVALLSSKAEAKIEAATGGNGLLRTLVQMDAPDHPKFRALTQKWFTPGNLRAIEGRIRDIARSYA
DRLTASGQHCDFVSEIALGFPLRVVMTILGVDPDHEPKMLRLTQEFFGTKDLDNARKGRNDGATALSDVVLEF
GEFFSSVVADRRANPSDDLASVIANAQIDGESISDLDALSYFVTIATAGHDTTSSSSAGGMWALCEHPQQFER
LKANPGLIPGFVDEAIRWTTPVKHFMRTAAEDTELDGRSIRRGDWLMLCYASANRDEAVFQDPDTFRIDRSPN
RHLAFGYGAHLCLGQYLAKLEMRILFEELLQRISCVELDGEGATTVSWFVNGPKRLPIRFQLR

>CYP108G20(HNE_0939)*Hyphomonas neptunium* ATCC 15444

MSDSAVLAVPPDTKKDPARRMSEAAAATCDQIVTPAVYADPPTLDAAFKRLRAEDPLAWCEPEGFRPFWAVTK
HADIMDVSRQNQLFTNGQREMLSYHEAEKGVYAKFGQPHLLKTLVQLDDPSHYKLRHLTQEWFMPQNVKKRDD
AVRSIAKQYVDRMEDLGGECDFQRDIALYYPLRVIMQILGVPESDEPMMLKLTQEIFGAQDEDLMRDKSLAEE
RDVEKGLASIQATLAEFFMYFTNITADRRANPKDDVSTVIANGMIDGEPIGQLEAMSYYIITAAAGHDTTSAS
TGGGVLAMLQSPSELKKMMDDPKGMSRTAVDEAIRWTTPVKHFMRTAQDDYTLRGKNILKGESLLLCYPSGNR

DEEVFDDPYSFKADRSPNKLISFGYGGHMCLGMHLARLEMQAIYEELFSRVKSIELAGEPSFIKANFVGGPKS
IPVRYRF

>CYP108L2(S58_21390)*Bradyrhizobium oligotrophicum*

MQDTSLDQAIVDHETYGALDRQHILFSTLRKEDPVHWTEPPGFRPFWTISKHQDVIEIERQNEKFINAPRTKL
LSIDFETKVRDAMAGRPMLVRAINQMDNPDHAKYKKITHAWFQPREVRHLEDRIAVIARTSVDQMLEKGTECD
FYKDVAGWYPLRVIMLILGIPDEHGPRLLDITRAYFGGGDAEVQRGNDLIDATFAYVNFFKEVARERRRNPTD
DLASIIATAEVDGEPIGEFETSSYYVALASAGHDTISATLSGGLLALIENPGEMRKLKQNPDLIPAAVEEMVR
WVSPVKHFFRTATETYTLRGKTIKQGDHLLLAYPSANRDEDAFESPFAFVADRTPNRHVGFGFGIHACLGLYL
AKVEMIAFMRELLSRVDGIELAGEPAWTETSFVGGLKRLPIRFSRLH

>CYP108P1(BRADO_6084)*Bradyrhizobium* sp.ORS 278

MSAGWSRIDRSGAVARAVDVLISARGHASPARLEHACALLRERAPLLWVERPGVRPFWAVTRYADIVSMETRS
GQFAAGPRTYLASETSEAVLQRVTGKPQLVRSLTEMDPPDHGVYRAILQNAFAPPALREMEAWLARWAAEIID
RVAARGSVCDFACDVAVPFTFRVIGHMLGTPEADDAQLARLAQAFVGAEDPQRRLAEAPGDTMRMAMLALRDY
FEAVVADRRAQPRADLATLIARAEPHGRAMPHYELISFFILLVTAGHDTTAFALSGGLEALLAAPDQFERLRT
APDLLDSAIEEMLRWTTPVRHFMRTALCDTEVGGRRIREGEALALFFHSANRDEAVFADAGEFRIDRSPNPHI
AFGRGPHICMGLQLARMQMRAMFAELLRRTDRIERAGRVRRVQSQFMSGVGALPVRISLCAARRSTVA

>CYP108P2(S58_62710)*Bradyrhizobium oligotrophicum*

MEVLISSRGHASPSRLERACALLRREAPLHWVERPDVRPFWAVTRYADIVSIETRSGQFAAGPRTYLASETSE
AVLQRVTGKPQLVRSLTEMDPPDHAVYRAIIQGAFAPPALRELETWLARWAAEIIDRIAACSGPIDFAADVAL
PFTFRVIGRMLGTPEADDARLARLAQAFVGAEDPQRRLAESPGDTMRMAMLALRDYFEAVVADRLAYPRDDLT
SLIARAEPHGAAMPHYELISYFILLVTAGHDTTALALGGGLEALLAAPDQFARLRAEPDLLDSAIEEMLRWTT
PVRHFMRTALGDTEVAGQPIREGDALALFFHSANRDESVFESAGAFLIDRSPNPHIAFGRGPHVCMGLQLARM
QMRAMFAELLRRTERIERAGRVRRVQSPFMSGVSRLPVRIAFRASFDAAAE

>CYP108U1(SGRAN_1188)*Sphingopyxis granuli*

MSAQNLVTQPQEQGNAPQLPADMASRLTDPAVWADEAGLHELLATIRAQYPLAIADVPGFRPFWVVSKYQDIM
EISRQNSLFHSGDLSAVLMPEEEYQKLAGGGEDAEKVHRSIVYMDPPEHGRYRMLTQAWFQPGSVRKREEEIR
AIAKKTVERMRAAGGTCDFVSDIAVHYPLEVIMNILGIPDEHYSRVLRLTQEVFSVADEELGREGDDTFSLGS
DNVFRDFNAFLRPFHEDRKVNPRDDVLSIIANAQIDGQPVPEIEALSYYVTVATAGHDTTSSTSATALWALSR
SPELFRELKEDPSRIPALIDEALRWTTPVRHFMRSATEDYELRGQLIRKGDWLMLCYPSGNRDEEIFENPQQF
QPDRSPNKHVAFGYGAHVCLGQHLAKLEIRILLEELLANLDAIEPAGPPKYTKAIFVGGLKSLPIRYQTA

>CYP108V1(P73_0333)*Celeribacter indicus*

MDQQTTSNAIADPKTYGDPSKMYALFERLRREEPVRWCEPEGYRPFWAIARHAEIMEIERQPDLFISAPRTAL
RTFAQEENIRKMTGSYQMVRTLLQMDPPDHRKYRALSQAWFMPGKLRSLEPAMKRLAGEFIDRMESLDGNCDF
AKDIASLYPLRAILQILGVPPEDEPMVLKLTQQFFAGTDPSTFDEDNEETDITSAAAQLFPYFNEKVAERRKE
PKNDLFTLLADAEVDGQPISDFERNSYFFIVAVAGHDTTSLTMASFMHALVEHPDQLRRLQENPALLEDAINE
AVRWASPVFHFMRTATADYEIGGKTIRKDDSIMLLYPSANRDESVFEAPEEFRIDRQPNRHVGFGYGPHLCLG
QHFAKLELRMFFGQLLPRLREVSLDGEPQWLETNFVGGLKTLPISYRLNTPELV

>CYP108W1(Swit_3069)*Sphingomonas wittichii*

MNGATRTIDKPDVERAIVDPATYMDRAAYDAIFTGLRRDDPIRWIEPEGFRPFWLVTRSADIVDIERRATIFL
NGPRAVLKPLAFEERLARQQRGHSEIIKSMNNMDGEAHRANRAVTSRDFLRPNIERMAAQLEPVAADFVQRLV
DKAPTCDFAADVTMWYPLRVILTLLGVAPEHDARILALTQRIFLDPGDGADPAAAQADKMAAVEGFFDFFRPI
VADRRANPRDDIASKVANARIDGEPMDEFETLSYFLTLATAGHDTTAASIAGGLLALIRNPGEMEKLRGDPGL
LETAADEMIRWNAPVKHFFRTAAEDFEIGGKAIRAGDSVMLAFASACRDEDLFEDPFAFRVDRTPNRHLALGS
GPHACLGQHLAKLEIRLFFRHLLDRIDHIELVGEPRSNPSSFISGIVSLPIRYRVRR

>CYP108X1(LH20_00540)*Sphingopyxis* sp. 113P3

MTKPILPGEVAAAVIDPTAYAAWEPLHEQLAWARANAPLAVAENPTHDPFWLVTRHADIMAISRDPQRFANGI
RPTVLTDRDGEALARAATPGGDGHLIRSLVQMDAPDHMKYRLLTQGWFMPKNLRIVEERIREIARETVDHMLS
LGDTCDFARDVAAHYPLRVIMDILGVPPEDEPRMLMLTQQLFGSTDPELNRGREAVKSTEQVIAMLHYVIADF
ENYFRNLTADRRANPREDIATVIANATIDGGQIPDRELAGYYMIVATAGHDTTSASTAGAMMELAKCPALFAR
FRDAASDKGGLIEEAIRWSTPVQHFMRSAKEDVEIGGQTIRQGDWLMLNYVSANRDEAVFADPFTFDPDRARN
QQIAFGFGAHVCLGQHLARMEMRILMEELLPRLKSVELAGEPARVESVFVGGLKRLPIRFEAA

>CYP108X2(SGRAN_3743)*Sphingopyxis granuli*

MTKPILPGEVAAAVVDPVAYGEWDGLHQQLAWARAHAPLAVAENPDHDPFWLVTRHADIMAISRDPQRFANGV
RQTVLTNREGEALARAATPGNDGHLIRSLVQMDAPDHMKYRLLTQAWFAPKNLRIVEDRVRKLAREAVDHMLS
FDGECDFARDVAAHYPLRVIMDILGVPPEDEPRMLMLTQQLFGSTDPDLNRSREAITSAEQAIAMLHYVIADF
ETYFRELTADRRASPREDIATVIANATIDGGPIPDRELAGYYMIVATAGHDTTSASTAGAMMELAKNPALFER
FRDAETEKAGLIEEAIRWSTPVQHFMRSAKADVEVGGQTIREGDWLMLNYVSANRDEAVFDDPFTFDPDRRKN
QQIAFGFGAHVCLGQYLARMEMRILMEELLPRLKKIELAGEPARVESVFVGGLKRLPIRFEAA

>CYP108X3(Sala_2021)*Sphingopyxis alaskensis*

MTKPILPGEVAAAVVNPAAYGAWKPLHEQLAWARANMPLAVAENPNHDPFWLVTRHADVMAISRDPQRFANGI
RPTVLTDRAGEALARAATPGGDGHLVRSLVQMDAPDHMKYRLLTQSWFMPRNLKTIEDRIRQIARDTVEHMLE
AGGSCDFARDVAAHYPLRVIMDILGVPPEDEPRMLMLTQQLFGPTDPELNRSREAITSSEQAIAMLHYVIADF
EAYFGALTADRRANPREDIATVIANAMVDGEQIPDRELAGYYMIIATAGHDTTSASTAGAIMELARNPTLFQR
FRDAESDKAGLIEEAIRWTTPVQHFMRSARQDVEMGGQTIREGDWLMLNYVSANRDEGVFVDPFMFDPDRAKN
PQIAFGFGAHVCLGQHLARLEMRILMEELLPRLTSLELAGEPARVESVFVGGLKRLPIRFEAA

>CYP108X4(LH19_00225)*Sphingopyxis macrogoltabida* 203

MFTRSTNAPNRGSFGCMTKPILPGEVAAAVINPVAYGQWEPLHEQLAWARANAPLAVAENPNHDPFWLVTRHA
DIMAISRDPQRFANGIRPTVLTDRDGEALARAATPNNDGHLIRSLVQMDAPDHMKYRLLTQSWFMPKNLKIVE
ERIREIARDTVDHMLEAGGECDFARDVAAHYPLRVIMDILGVPPEDEPRMLMLTQQLFGPTDPELNRSREAIT
SAEQAIAMLNYVIADFENYFGALTADRRANPREDIATVIANAMVGGEQIPDRELAGYYMIIATAGHDTTSAST
AGAMMELAKNPALFERFRSAESDKAGLIEEAIRWTTPVQHFMRSAKEDVEIGGQTIHAGDWLMLNYVSGNRDE
EVFTDPFAFDPDRVKNQQIAFGFGAHVCLGQHLARMEMRILMEELLPRLKSLELAGEPARVESVFVGGLKRLP
LHFRTH

>CYP108X5 (SKP52_00530)*Sphingopyxis fribergensi*

MTKPILPGEVAAAVVDPVAYGNWDPLHEQLTWARANMPLGVAENHDHDPFWLVTRHADIMAISRDPQRFANGI
RPTVLTNRDGEALARAATPNNDGHLIRSLVQMDAPDHMKYRLLTQSWFMPKNLKIVEERIRQIARETVDHMLE
QGGTCDFARDVAAHYPLRVIMDILGVPPEDEPRMLMLTQQLFGPTDPELNRSREAITSAEQAIAMLDYVIADF
TQYFGALTADRRANPREDIATVIANATINGEQIPDREMSGYYMIVATAGHDTTSASTAGAMMELAKNPALFER
FRSADSDKAGLIEEAIRWTTPVQHFMRSAKEDVEIGGQTIREGDWLMLNYVSANRDEGVFGEPFAFNPDREKN
QQIAFGFGAHVCLGQHLARMEMRILMEELLPKLKSVELAGEPARVESVFVGGLKRLPVRFEAA

>CYP111A2(Saro_0759)*Novosphingobium aromaticivorans*

MLDLKNPDTYQGGVPYAALQDLRAEGPVHWNPESDGAGFWAVLGHDEIVAVSRQPDLFSSAFENGGHRIFNEN
QVGLTGAGESAIGIPFISRDPPSHTQYRKFVMPALSPARLQGIEERIAKRVERLFAQVPLGETVNILPLLTVP
LPLLTLAELLGVPADLWPDLHRWTDAFVGEDDPDFRQSPEAMQAVLAEFMGFATALFEDRRANPGPDIASLLA
NTEIRGEPAPLRDFIANLILALVGGNETTRNSINHTMIALAENPGQWDILRADPSLMTAAVKEMVRFASPVIH
MRRTAMRDTQLGQQAICKGDKVVIFYPAGNRDPAVFENPDRFEITRPVRQHLAFGSGAHVCVGSRLAEMQLRL
AFAEMARHVRAFEVVGEPSRVRSNFINGFKRLEVRLLV

>CYP111B1(Plav_0029)*Parvibaculum lavamentivorans*

MLRPQAKAIDLNGKPDLKDPDLYLHEQHHEAFRRLRAEEPVYWNPEADGPGFWAVTRYDDIEAVSKNPKLFSS
AKHNGGHRIFNENEIGGNDTDASMISMDPPAHAGYRRMVTPGFVPKRISNMEERIRARVTRLLDAMPKTGEAE

FISAVAAALPIEVLAELFGVPESDGPKLFEWSNATVGEDDPELRVSDEYMQKCIMEMAGYAAGLWQQRLETPG
DDLISMLAHSKIGGEAMNFPTYIGTFILLVVAGNETTRNSISGGLLALSENPGERQKLLDDPSLIPSAVQEIV
RWVSPVLHMRRTATEDTELRGQKIRKGDKVVMWYASGNRDEAQWADPYRFDVARYAAPGVPAQIGFGVGQHFC
LGSRLAELQLTILFEELLRRFPDINVSGPIRRLRSNFIYGIKEMPVSYTPEG

>CYP112A2(NGR_a02700)*Sinorhizobium fredii* NGR234

MPEQPLPTLPMWRVDHIEPSPTMLALRANGPIHNVRFPRGHEGWWVTGYDEAKAVLSDAAFRPAGMPPAAFTP
DCVILGSPGWLVSHEGGEHARLRTIVAPAFSDRRVKLLAQQVEAIAAQLFETLAAQPQPADLRRHLSFPLPAM
VISALMGVLYEDHAFFAGLSDEVMTHQHESGPRSASRLAWEELRAYIRGKMRDKRQDPGDNLLTDLLAAVDRG
EATEEEAIGLAAGMLVAGHESTVAQIEFGLLAMLRHPQQRERLVGNPSLVDKAVEEILRMYPPGAGWDGIMRY
PRTDVTIAGVHIPAESKVLVGLPATSFDPRHFEDPEIFDIGRDAKPHLAFSYGPHYCIGMALARLELKVVFGS
IFQRFPALRLAVAPEELKLRKEIITGGFEEFPVLW

>CYP112A3v1(mlr_6364)*Mesorhizobium loti* MAFF 303099

MSEQPLPTLPMWRVDHIEPSPEMLALRANGPIHHVRFPSGHEGWWVTGYDEAKAALSDAAFRPAGMPPAAFTP
DSVILGSPGWLVSHEGGEHARLRTIVAPAFSNRRVKVLAQQVEAIAAQLFETLAAQPQPADLRRHLSFPLPAM
VISALMGVLYEDHAFFAGLSDEVMTHQHESGPRSASRLAWEELRAYIRGKMWDKRQDPGDNLLTDLLAAVEQG
NATEEEAIGLAAGMLVAGHESTVAQIEFGLLAMFRHPQQRERLVGDPSLVDKAVEEILRMYPPGAGWDGIMRY
PRTDVTIAGVHIPAESKVLVGLPATSFDPRHFDDPEIFDIGRDENPHLTFSHGPHYCIGMALARLELKVVVGS
IFQRFPALRLAVAPEELKLRKEIITGGFEEFPVLW

>CYP112A4(RHE_PD00252)*Rhizobium etli* CFN 42

MSEQSLPTLPMWRVDHIEPSPEMLALRAKGPIHRVRLPSGHECWWVTGYDEAKAVLSDAAFLPAGMPPADFTP
DSVILGSPGWLVSHEGDEHARLRTIVAPAFSNSRVKLLTQQVEAITVQLFDTLAVQPQPADLRRHLSFPLPAK
VISALMGVPFEEHAFFAGLSDEVMTHQHESGPRSASGLAWEELRAYIHGKIRGKRQDPGDNLLTDLLAAVDQG
KATEEEAIGLAAGVLVAGHESTVAQIEFGLLAMFRHPQQRERLVRDPSLVDKAVEEILRMYSPGAGWDGIMRY
PRTDVTIAGVHIPAESKVLVGLPATSFDPCHFKDPEVFDIGRDANPHLAFSYGQHNCIGAALARLELKAIFGS
IFQRFPALRLAVAPEELKLRKEIITGGFEEMPVLWCGRPPASQSSHLAAPGAHRSDQPLDR

>CYP112A4(AMC79_PC00281)*Rhizobium phaseoli*

MSEQSLPTLPMWRVDHIEPSPEMLALRAKGPIHRVRLPSGHECWWVTGYDEAKAVLSDAAFLPAGMPPADFTP
DSVILGSPGWLVSHEGDEHARLRTIVAPAFSNSRVKLLTQQVEAITVQLFDTLAVQPQPADLRRHLSFPLPAK
VISALMGVPFEEHAFFAGLSDEVMTHQHESGPRSASGLAWEELRAYIHGKIRGKRQDPGDNLLTDLLAAVDQG
KATEEEAIGLAAGVLVAGHESTVAQIEFGLLAMFRHPQQRERLVRDPSLVDKAVEEILRMYSPGAGWDGIMRY
PRTDVTIAGVHIPAESKVLVGLPATSFDPCHFKDPEVFDIGRDANPHLAFSYGQHNCIGAALARLELKAIFGS
IFQRFPALRLAVAPEELKLRKEIITGGFEEMPVLWCGRPPASQSSHLAAPGAHRSDQPLDR

>CYP112A4(AMK02_PC00299)*Rhizobium* sp. N731

MSEQSLPTLPMWRVDHIEPSPEMLALRAKGPIHRVRLPSGHECWWVTGYDEAKAVLSDAAFLPAGMPPADFTP
DSVILGSPGWLVSHEGDEHARLRTIVAPAFSNSRVKLLTQQVEAITVQLFDTLAVQPQPADLRRHLSFPLPAK
VISALMGVPFEEHAFFAGLSDEVMTHQHESGPRSASGLAWEELRAYIHGKIRGKRQDPGDNLLTDLLAAVDQG
KATEEEAIGLAAGVLVAGHESTVAQIEFGLLAMFRHPQQRERLVRDPSLVDKAVEEILRMYSPGAGWDGIMRY
PRTDVTIAGVHIPAESKVLVGLPATSFDPCHFKDPEVFDIGRDANPHLAFSYGQHNCIGAALARLELKAIFGS
IFQRFPALRLAVAPEELKLRKEIITGGFEEMPVLWCGRPPASQSSHLAAPGAHRSDQPLDR

>CYP112A1(blr_2144)*Bradyrhizobium diazoefficiens* USDA 110

MSEQQPLPTLPMWRVDHIEPSPEMLALRANGPIHRVRFPSGHEGWWVTGYDEAKAVLSDAAFRPAGMPPAAFT
PDSVILGSPGWLVSHEGREHARLRAIVAPAFSDRRVKLLVQQVEAIAAHLFETLAAQPQPADLRRHLSFPLPA
MVISALMGVLYEDHAFFAGLSDEVMTHQHESGPRSASRLAWEELRAYIRGKMRDKRQDPDDNLLTDLLAAVDQ
GKASEEEAVGLAAGMLVAGHESTVAQIEFGLLAMFRHPQQRERLVGDPSLVDKAVEEILRMYPPGAGWDGIMR
YPRTDVTIAGEHIPAESKVLVGLPATSFDPHHFDDPEIFDIERQEKPHLAFSYGPHACIGVALARLELKVVFG
SIFQRLPALRLAVAPEQLKLRKEIITGGFEQFPVLW

>CYP112A1(BJ6T_77060)*Bradyrhizobium japonicum* USDA 6

MSEQQPLPTLPMWRVDHIEPSPEMLALRANGPIHRVRFPSGHEGWWVTGYDEAKAVLSDAAFRPAGMPPAAFT
PDSVILGSPGWLVSHEGREHARLRAIVAPAFSDRRVKLLVQQVEAIAAHLFETLAAQPQPADLRRHLSFPLPA
MVISALMGVLYEDHAFFAGLSDEVMTHQHESGPRSASRLAWEELRAYIRGKMRDKRQDPDDNLLTDLLAAVDQ
GKASEEEAVGLAAGMLVAGHESTVAQIEFGLLAMFRHPQQRERLVGDPSLVDKAVEEILRMYPPGAGWDGIMR
YPRTDVTIAGEHIPAESKVLVGLPATSFDPHHFDDPEIFDIERQEKPHLAFSYGPHACIGVALARLELKVVFG
SIFQRLPALRLAVAPEQLKLRKEIITGGFEQFPVLW

>CYP112A1(RN69_37420)*Bradyrhizobium japonicum* E109

MSEQQPLPTLPMWRVDHIEPSPEMLALRANGPIHRVRFPSGHEGWWVTGYDEAKAVLSDAAFRPAGMPPAAFT
PDSVILGSPGWLVSHEGREHARLRAIVAPAFSDRRVKLLVQQVEAIAAHLFETLAAQPQPADLRRHLSFPLPA
MVISALMGVLYEDHAFFAGLSDEVMTHQHESGPRSASRLAWEELRAYIRGKMRDKRQDPDDNLLTDLLAAVDQ
GKASEEEAVGLAAGMLVAGHESTVAQIEFGLLAMFRHPQQRERLVGDPSLVDKAVEEILRMYPPGAGWDGIMR
YPRTDVTIAGEHIPAESKVLVGLPATSFDPHHFDDPEIFDIERQEKPHLAFSYGPHACIGVALARLELKVVFG
SIFQRLPALRLAVAPEQLKLRKEIITGGFEQFPVLW

>CYP112A1(RGR602_PB00446)*Rhizobium gallicum*

MSEQPLPTLPMWRVDHIEPSPEMLALLANGPIHRVRFPSGHEGWWVTGYDEAKAVLSDAAFRPAGMPPAAFTP
DSVILGSPGWLVSHEGEEHARLRTIVAPAFSNRRVELLAQQVEAIAAQLFETLAAQPQPADLRRHLSFPLPAM
VISALMGVLYEDHAFFAGLSDEVMTHQHESGPRSASRLAWEELRAYIRGKMRDKRQDPGANLLTDLLAAVDQG
EATEEEAIGLAAGMLVAGHESTVAQIEFGLLAMFRHPQQRERLVGDPSLVDKAVEEILRMYPPGAGWDGIMRY
PRTDVTIAGVHIPAESKVLVGLPATSFDPRHFDDPEIFDIGRDGKPHLAFSYGPHNCIGVALARLELKAVFGS
IFQRFPGLRLAVAPEELKLRKEIITGGFEEMPVLW

>CYP112A3(LMTR13_26575)*Bradyrhizobium icense*

MSEQSLPTLPMWRVDHIEPSPEMLALRANGPIHRVRFPSGHEGWWVTGYDEAKAALSDTAFRPAGMPPAAFTP
DSVILGSPGWLVSHEGGEHARLRTIVAPAFSNRRVKLLAQQVEAIAAQLFETLAAQPQPADLRRHLSFPLPAM
VISALMGVLYEDHAFFAGLSDEVMTHQHESGPRSASRLAWEELRAYIRGKMRDKRQDPGDNLLTDLLAAVDQG
KATEEEAIGLAAGMLVAGHESTVAQIEFGLLAMFRHPQQRERLVGDPSLVDKAVEEILRMYPPGAGWDGIMRY
PRTDVTIAGVHIPAESKVLVGLPATSFDPRHFDDPEIFDIGRQEKPHLAFSYGPHSCIGAALARLELKAVFGS
IFQRFPALRLAVAPEELKLRKEIITGGFEEFPVLW

>CYP112A3(SAMCFNEI73_pB0340)*Sinorhizobium americanum*

MSEQPVPTLPMWRVDHIEPSPEMLALRANGPIHRVRFPSGHEGWWVTGYDEAKAALSDAAFRPAGMPPAAFTP
DSVILGSPGWLVSHEGCEHARLRTIVAPAFSDRRVKLLAQQVEAIAAQLFETLAAQPQPADLRRHLSFPLPAM
VISALMGVLYEDHAFFAGLSDEVMTHQHESGPRSASRLAWQELRAYIRGKMRDKRQDPGDNLLTDLLAAVDQG
KATEEEAIGLAAGMLVAGHESTVAQIEFGLLAMFRHPQQRERLVGDPSLVDKAVEEILRMYPPGAGWDGIMRY
PRIDVTIAGVHIPAESKVLVGLPATSFDPRHFAHPEIFDIGRDEQPHLAFSHGPHYCIGAALARLELKVVFGS
IFQRFPALRLAVAPEELKLRKEIITGGFEEFPVLW

>CYP112A3(A6B35_33010)*Mesorhizobium amorphae*

MSEQPLPTLPMWRVDHIEPSPEMLALSANGPIHRVRFPSGHEGWWVTGYDEAKAVLSNAAFRPAGMPPAAFTP
DSVILGSPGWLVSHEGGEHARLRTIVAPAFSNGRVKQLAQQVEAIAAQLFETLAAQPQPADLRCHLSFPLPAM
VISALMGVLYEDHAFFAGLSDEVMTHQHESGPRSASRLAWEELRAYIRGKMRDKRQDPGDNLLTDLLAAVDEG
KATEEEAIGLAAGMLVAGHESTVAQIEFGLLAMFRHPQQRERLVGDPSLLDKAVEEILRMYPPGAGWDGIMRY
PRTDVTIAGVHIPAESKVLVGLPATSFDPRHFENPEIFDIGRDEKPHLAFSYGPHYCIGMALARLELKVVFGS
IFQRFPELRLAVAPEELKLRKEIITGGFEEFPVLW

>CYP112A4(RHECIAT_PB0000291)*Rhizobium etli* CIAT 652

MSEQSLPTLPMWRVDHIEPSPEMLALRAKGPIHRVRLPSGHECWWVTGYDEAKAVLSDAAFRPAGMPPADFTP
DSVILGSPGWLVSHEGDEHARLRTIVAPAFSNSRVKLLTQQVEAITVQLFDTLAVQPQPADLRRHLSFPLPAK
VISALMGVPFEEHAFFAGLSDEVMTHQHESGPRSASGLAWEELRAYIHGKIRGKRQDPGDNLLTDLLAAVDQG
KATEEEAIGLAAGVLVAGHESTVAQIEFGLLAMFRHPQQRERLVRDPSLVDKAVEEILRMYSPGAGWDGIMRY
PRTDVTIAGVHIPAESKVLVGLPATSFDPRHFKDPEVFDIGRDANPHLAFSYGQHNCIGAALARLELKAIFGS
IFQRFPALRLAVAPEELKLRKEIITGGFEEMPVLWCGRPPASQSSHLAAPGAHRSDQPLDR

>CYP112A4(IE4803_PB00399) *Rhizobium etli* bv. Phaseoli IE4803
MSEQSLPTLPMWRVDHIEPSPEMLALRAKGPIHRVRLPSGHECWWVTGYDEAKAVLSDAAFLPAGMPPADFTP
DSVILGSPGWLVSHEGDEHARLRTIVAPAFSNSRVKLLTQQVEAITVQLFDTLAVQPQPADLRRHLSFPLPAK
VISALMGVPFEEHAFFAELSDEVMTHQHESGPRSASGLAWEELRAYIHGKIRGKRQDPGDNLLTDLLAAVDQG
EATEEEAVGLAAGVLVAGHESTVAQIEFGLLAMFRHPQQRERLVRDPSLVDKAVEEILRMYSPGAGWDGIMRY
PRTDVTIAGVHIPAESKVLVGLPATSFDPRHLKDPEVFDIGRDANPHLAFSYGQHNCIGAALARLELKAVFGS
IFQRFPALRLAVAPEELKLRKEIITGGFEEMPVLWCGRPPASQSSHLTAPGAGRSDQPPDR

>CYP112A4(AMJ98_PC00304)*Rhizobium* sp. N1341

MSEQSLPTLPMWRVDHIEPSPEMLALRAKGPIHRVRLPSGHECWWVTGYDEAKAVLSDAAFLPAGMPPADFTP
DSVILGSPGWLVSHEGDEHARLRTIVAPAFSNSRVKLLTQQVEAITVQLFDTLAVQPQPADLRRHLSFPLPAK
VISALMGVPFEEHAFFAELSDEVMTHQHESGPRSASGLAWEELRAYIHGKIRGKRQDPGDNLLTDLLAAVDQG
EATEEEAVGLAAGVLVAGHESTVAQIEFGLLAMFRHPQQRERLVRDPSLVDKAVEEILRMYSPGAGWDGIMRY
PRTDVTIAGVHIPAESKVLVGLPATSFDPRHLKDPEVFDIGRDANPHLAFSYGQHNCIGAALARLELKAVFGS
IFQRFPALRLAVAPEELKSRKEIITGGFEEMPVLWCGRPPASQSSHLTAPGAGRSDQPPDR

>CYP112A7(RTCIAT899_PB00455)*Rhizobium tropici*

MSEQPLPTLPMWRLNHIEPSPEMLTLSANGPIHRVRFPSGHEGWWVTGYDEAKAVLSDTAFRPAGMPPAAFTP
DSVILGSPGWLVSHEGDEHARLRTIVGRAFSDRNMELLAQKVEAIAAQLFETLAAQPQPADLRLHLSFPLPAI
VISALMGVLYEDHAFFAGLSDEVMTHQHESGPRSASRLAWEELRAYIRGKMRDKREDPGDNLLTDLLAAVDQG
KATEEEAIGLAAGMLVAGHESTVAQIEFGLLAMFRHPQQRERLVGDPSLVDKTVEEILRMYPPGAGWDGVMRY
PRTDVTIAGVRIPAESKVLVGLPATAFDPRHFEDPEIFDIGREGKPHLAFSYGPHYCIGVALARLELKAVFGS
IFQRFPALRLAVAPEKLKLRKEIITGGFEEFPVFW

>CYP112A8(IE4771_PB00363)*Rhizobium etli* bv. Mimosa IE4771

MFEQPLPTLPMWRVDHIEPSPEMLALRAKGPIHRVRFPSGHEGWWVTGYDEAQAVLSDAAFRPAGMPPETFTP
DSVILGSPGWLVSHEGGKHAWLRMIVAPAFSNRRVKLLAQQVEAIAAQLFETLAAQPQPADLRRHLSFPLPAM
VISALMGVLYEDHIFFAGLSDEVMTHQHESGPRSASRVAWEELRTYICRKMRGKREEPGDNLLTDLLAAVDHG
KATEEEAVGLAAGVLVAGHESTVAQIEFGLLAMFRHPQQRERLVRDPFLADKAVEEILRMYSPGAGWDGIMRY
PRTDVTIAGMDIPAESKVLVGLPATSFDPRHFEDPEVFDIGRDPNPHLAFSYGPHNCIGAALARLELKVVFGS
IFQRFPALRLAVAPEELKLRKEIITGGFEEFPVLW

>CYP112A8(REMIM1_PE00083)*Rhizobium etli*

MFEQPLPTLPMWRVDHIEPSPEMLALRAKGPIHRVRFPSGHEGWWVTGYDEAQAVLSNAAFRPAGMPPETFTP
DSVILGSPGWLVSHEGGKHAWLRTIVAPAFSNRRVKLLAQQVEMVAAQLFDTLAAQPQPADLRCHLSFPLPAM
VISALMGVLYEDHTFFAGLSDEVMTHQHESGPRSASSVAWEELRTYICRKMRGKREEPGDDLLTDLLAAVDRG
KATEEEAVGLAAGVLVAGHESTVAQIEFGLLAMFRHPQQRKRLTRDPSLADRAVEEILRMYSPGAGWDGIMRY
PRIDVTIAGVDIPAESKVLVGLPATSFDPRHFEDPEVFDIGRDPNPHLAFSYGPHNCIGAALARLELKVVFGS
IFQRFPALRLAVAPEELKLRKEIITGGFEEFPVLW

>CYP112A9(Sinme_5616)*Sinorhizobium meliloti* AK83

MSEQPLPMLPMWRVDHIEPSPEMLALRVKGPIHRVRFPSGDEGWWVTGYDEAKAVLSDAAFRPSGMPPAEFTP
ATVILGSPGWLGSHEGSEHARLRTIVAPAFSSGRVKLLAQQVEAIAAELFETLAAQPQPADLRRHLSFPLPAM
VISALMGVLYEDHAFFAGLSDKVMTHQHESGPRSAARLAWEELRAYIRGKMRDKRQDPGDNLLTDLLAAVDQG
KATEEEAIGLAAGMLVAGHETTVAQIEFGLLAMFRHPQQRERLVGDPSLVDKAVEEILRMYPPGAGWDGIMRY

PRTDVTIAGVHIPAESKVLVGLPATSFDPRHFDDPEIFDVGREEKPHLAFSYGPHYCIGVELARLELRVVFGS
IFQRFPALRLAVAPEELKLRKAIITGGFEAFPVLW

>CYP112A9(SM11_pC0151)*Sinorhizobium meliloti* SM11

MSEQPLPMLPMWRVDHIEPSPEMLALRVKGPIHRVRFPSGDEGWWVTGYDEAKAVLSDAAFRPSGMPPAAVTS
ATVILGSPGWLGSHEGSEHARLRTIVAPAFSSGRVKLLAQQVEAIAAELFETLAAQPQPADLRRHLSFPLPAM
VISALMGVLYEDHAFFAGLSDKVMTHQHESGPRSAARLAWEELRAYIRGKMRDKRQDPGDNLLTDLLAAVDQG
KATEEEAIGLAAGMLVAGHETTVAQIEFGLLAMFRHPQQRERLVGDPSLVDKAVEEILRMYPPGAGWDGIMRY
PRTDVTIAGVHIPAESKVLVGLPATSFDPRHFDDPEIFDVGREEKPHLAFSYGPHYCIGVELARLELRVVFGS
IFQRFPALRLAVAPEELKLRKAIITGGFEAFPVLW

>CYP112A9(SinmeB_5267)*Sinorhizobium meliloti* BL225C

MSEQPLPMLPMWRVDHIEPSPEMLALRAKGPIHRVRFPSGDEGWWVTGYDEAKAVLSDAAFRPSGMPPAAVTS
ATVILGSPGWLGSHEGSEHARLRTIVAPAFSSGRVKLLAQQVEAIAAELFETLAAQPQPADLRRHLSFPLPAM
VISALMGVLYEDHAFFAGLSDKVMTHQYESGPRSAARLAWEELRAYIRGKMRDKRQDPGDNLLTDLLAAVDQG
KATEEEAIGLAAGMLVAGHETTVAQIEFGLLAMFRHPQQRERLVGDPSLVDKAVEEILRMYPPGAGWDGIMRY
PKTDVTIAGVHIPAESKVLVGLPATSFDPRHFDDPEIFDVGREEKPHLAFSYGPHYCIGVELARLELRVVFGS
IFQRFPALRLAVAPEELKLRKAIITGGFEAFPVLW

>CYP112A9(C770_GR4pB020)*Sinorhizobium meliloti* GR4

MSEQPLPMLPMWRVDHIEPSPEMLALRAKGPIHRVRFPSGDEGWWVTGYDEAKAVLSDAAFRPSGMPPAAVTS
ATVILGSPGWLGSHEGSEHARLRTIVAPAFSSGRVKLLAQQVEAIAAELFETLAAQPQPADLRRHLSFPLPAM
VISALMGVLYEDHAFFAGLSDKVMTHQYESGPRSVARLAWEELRAYIRGKMRDKRQDPGDNLLTDLLAAVDQG
KATEEEAIGLAAGMLVAGHETTVAQIEFGLLAMFRHPQQRERLVGDPSLVDKAVEEILRMYPPGAGWDGIMRY
PRTDVTIAGVHIPAESKVLVGLPATSFDPRHFDDPEIFDVGREEKPHLAFSYGPHYCIGVELARLELRVVFGS
IFQRFPALRLAVAPEELKLRKAIITGGFEAFPVLW

>CYP112A-fragment(C770_GR4pB120)*Sinorhizobium meliloti* GR4

MTEEEAIGLAAGMLVAGHETTVTQIEFGLLSMFRHPQQRERLVGDPSLVDKAVEEILRMYPPGAGWDGIMRYP
KTDVTIAGMHIPAESKVLVDCRRRRSIRAISTTRKSSTSDARKSRTWRSPTARTIASAWSRPGWNSRWCSVRF
SSAFPRCAWPWRQKCLLALDGLAKSNRLDAVVIARYIADLPTLEMRIDPLREKPIRQHRKICSSGGTPTAVA

>CYP114A2(NGR_a02710)*Sinorhizobium fredii* NGR234

MDMQETTTACADAFAELASPACIDDPYPFMRWLREHDPVHRAASGLFLLSRHADICWALKATGDAFRGPAPGE
LARYFPRAATSLSLNLLASTLAMKEPPTHTRLRRLISRDFTMREIDNLRPSIARFVAARLDGMAPALERGEAV
DLHRQFALALPMLVFAELFGMPQDDMFGLAAGIGAILEGLSPHASDPQLAAADAASARMKAYFGDLIQRKCID
PRHDIVATLVGAHDDDADTLSDAELISMLWGMLLGGFATTAATIDHAVLAMLAYPDQRHWLQGDAAGVEAFVE
EVLRCDAPAMFSSIPRIAQSDIELSGVVIPKNADVRVLIAAGNRDPDAFADPDRFDPARFYGTSPGMSTDGKI
MLSFGHGIHFCLGAQLARVQLAESLPRIQARFPTLTVAEQPTREPSAFLRTFRALPVRLHAQGDSPRLTSAFL
NGQRGVEGGASFEHGDGERRSATDRRAQP

>CYP114A3v1(mlr_6365)*Mesorhizobium loti* MAFF 303099

MDVQETTAACRDAFAELASPACIQDPYTFMRWLREHDPVHRAASGLFLLSRHADIYWALKATGDVFRGPAPGE
LARYFPRAETSLSLNLLASTLAMKEPPTHTRLRRLISRDFTIRQIDNLRPSIARIVAARLDGMAPALERGEAV
DLHWEFALAVPILVFAELFGMPQDDMFGLAAGIGAILEGLSPHASDPQLAEADAASARVQAYFGDLIQRKRTD
PRNDIVSMVVGAHDDDADTLSDAELISMLWGMLLGGFATTAATIDHAVLAMLAYPEQRHWLQGDAVGVKAFVE
EVLRCDAPAMFSSIPRIAQRDIELGGVVIPKNADVRVLIAAGNRDPDAFSDPDRFDPARFYGTTPGMSTDGKI
MLSFGHGIHFCLGAQLARVQLAESLPRIEARFPTLALAEQPTREPSAFLRTFRALPVRLHAQGG

>CYP114A3(A6B35_33015)*Mesorhizobium amorphae*

MDVQEATAACRDAFAELASPACIHDPYPFMRWLREHDPVHRAASGLFLLSRHADIYWALKATGDAFRGPAPGE
LARYFPRAATSLSLNLLASTLAMKEPPTHTRLRRLISRDFTMCQIDNLRPSIALIVAARLDGIAPALERGEAV
DLHREFALALPMLVFAELFGMPQDDMFGLAAGIGAILEGLSPHASDPQLAAADAASARVQAYFGDLIQRKRTD
PRHDIVSMLVGAHDDDADTLSDAELISMLWGMLLGGFATTAASIDHAVLAMLAYPEQRHWLQGDAVGVKAFVE
EVLRCDAPAMFSSIPRIAQRDIELDGVVIPKNADVRVLLAAGNRDPDAFADPDRFDPARFYGTSPGMSTDGKI
MLSFGHGIHFCLGAQLARVQLAESLPRIQARFPTLALAEQPTREPSAFLRTFRALPVRLHAQGG

>CYP114A4(RHE_PD00251)*Rhizobium etli* CFN 42

MDVQDTTAACHDAFAELASPACIQDPYPFMRWLREHDPVHRAASGLFLLSRHADIYWAFKATGDAFRGPAPSE
LARYFPRAASSLSLNLLASTLAMKEPPTHTRLRRLISRDFTVGQIDNLRPSIARIVAARLDGMAPALERGEAV
DLHREFALALPMLVFAELFGMPQDDVFELSAIVSAILEGLSPHASDPQLAAADVASARVKAYFGDLILRKRAD
PRRDIVSTLVGAHTDDADTLSDAELISMLWGMLLGGFATTAATIDHAVLAMLAYPEERHWLQGDAAGVEAFVE
EVLRCEAPAMFSSIPRIAQRDIELHGVVIPKDADVRVLIAAGNRDPDAFADPDRFDPVRFYGTRPGMSSDGKI
MLSFGHGIHFCLGAQLARVQLAESLPQIQARFPTLALAEQPTREPSAFLRTFRALPVRLHAQAAAEVRVVVDQ
DLCGTTGQCVLTLPGTFRQREPDGVAEVCMATVPQALHAAVRLAASQCPVAAIRVIESEAGDDHCTNPGPTPS
PADAERHAAKDLRNPGEHDGTI

>CYP114A4(AMK02_PC00298)*Rhizobium* sp. N731

MDVQDTTAACHDAFAELASPACIQDPYPFMRWLREHDPVHRAASGLFLLSRHADIYWAFKATGDAFRGPAPSE
LARYFPRAASSLSLNLLASTLAMKEPPTHTRLRRLISRDFTVGQIDNLRPSIARIVAARLDGMAPALERGEAV
DLHREFALALPMLVFAELFGMPQDDVFELSAIVSAILEGLSPHASDPQLAAADVASARVKAYFGDLILRKRAD
PRRDIVSTLVGAHTDDADTLSDAELISMLWGMLLGGFATTAATIDHAVLAMLAYPEERHWLQGDAAGVEAFVE
EVLRCEAPAMFSSIPRIAQRDIELHGVVIPKDADVRVLIAAGNRDPDAFADPDRFDPVRFYGTRPGMSSDGKI
MLSFGHGIHFCLGAQLARVQLAESLPQIQARFPTLALAEQPTREPSAFLRTFRALPVRLHAQAAAEVRVVVDQ
DLCGTTGQCVLTLPGTFRQREPDGVAEVCMATVPQALHAAVRLAASQCPVAAIRVIESEAGDDHCTNPGPTPS
PADAERHAAKDLRNPGEHDGTI

>CYP114A4(AMC79_PC00280)*Rhizobium phaseoli*

MDVQDTTAACHDAFAELASPACIQDPYPFMRWLREHDPVHRAASGLFLLSRHADIYWAFKATGDAFRGPAPSE
LARYFPRAASSLSLNLLASTLAMKEPPTHTRLRRLISRDFTVGQIDNLRPSIARIVAARLDGMAPALERGEAV
DLHREFALALPMLVFAELFGMPQDDVFELSAIVSAILEGLSPHASDPQLAAADVASARVKAYFGDLILRKRAD
PRRDIVSTLVGAHTDDADTLSDAELISMLWGMLLGGFATTAATIDHAVLAMLAYPEERHWLQGDAAGVEAFVE
EVLRCEAPAMFSSIPRIAQRDIELHGVVIPKDADVRVLIAAGNRDPDAFADPDRFDPVRFYGTRPGMSSDGKI
MLSFGHGIHFCLGAQLARVQLAESLPQIQARFPTLALAEQPTREPSAFLRTFRALPVRLHAQAAAEVRAVVDQ
DLCGTTGQCVLTLPGTFRQREPDGVAEVCMATVPQALHAAVRLAASQCPVAAIRVIESEAGDDHCTNPGPTPS
PADAERHAAKDLRNPGEHDGTI

>CYP114A4(RHECIAT_PB0000290)*Rhizobium etli* CIAT 652

MDVQDTTAACHDAFAELASPACIQDPYPFMRWLREHDPVHRAASGLFLLSRHADIYWAFKATGDAFRGPAPSE
LARYFPRAASSLSLNLLASTLAMKEPPTHTRLRRLISRDFTVGQIDNLRPSIARIVAARLDGMAPALERGEAV
DLHREFALALPMLVFAELFGMPQDDVFELSAIVSAILEGLSPHASDPQLAAADVASARVKAYFGDLILRKRAD
PRRDIVSTLVGAHTDDADTLSDAELISMLWGMLLGGFATTAATIDHAVLAMLAYPEERHWLQGDAAGVEAFVE
EVLRCEAPAMFSSIPRIAQRDIELHGVVIPKDADVRVLIAAGNRDPNAFADPDRFDPVRFYGTRPGTSSDGKI
MLSFGHGIHFCLGAQLARVQLAESLPQIQARFPTLALAEQPTREPSAFLRTFRALPVRLHAQAAAEVRVVVDQ
DLCGTTGQCVLTLPGTFRQREPDGVAEVCMATVPQALHAAVRLAASQCPVAAIRVIESEAGDDHCTNPGPTPS
PADAERHAAKDLRNPGEHDGTI

>CYP114A4(IE4803_PB00398)*Rhizobium etli* bv. Phaseoli IE4803

MDVQETKAACHDAFAELASPACIQDPYPFMRWLQEHDPVHRAASGLFLLSRHADIYWAFKATGDAFRGPAPSE
LARYFPRAASSLSLNLLASTLAMKEPPTHTRLRRLISRDFTVGQIDNLRPSIARIVAARLDGMAPALERGEAV
DLHREFALALPMLVFAELFGMPQDDMFELSAIVSAILEGLSPHASDPQLAAADVASARVKAYFGDLILRKRAD
PRRDIVSTLVGAHSDDADTLSDAELISMLWGMLLGGFATTAATIDHAVLAMLAYPEERHWLQGDAAGVEAFVE
EVLRCEAPAMFSSIPRIAQRDIELHGVVIPKDADVRVLIAAGNRDPDAFADPDRFDPVRFYGTRPGMSSDGKI

MLSFGHGIHFCLGAQLARVQLTESLPQIQARFPTLALAEQPTRERSAFLRTFRALPVRLRAQAAAEVRVVVDQ
DLCGTTGQCVLTLQGAFRQREPDGVAEVCMATVPQALHAAVRLAASQCPVAAIG

>CYP114A4(AMJ98_PC00303)*Rhizobium* sp. N1341

MDVQETKAACHDAFAELASPACIQDPYPFMRWLQEHDPVHRAASGLFLLSRHADIYWAFKATGDAFRGPAPSE
LARYFPRAASSLSLNLLASTLAMKEPPTHTRLRRLISRDFTVGQIDNLRPSIARIVAARLDGMAPALERGEAV
DLHREFALALPMLVFAELFGMPQDDMFELSAIVSAILEGLSPHASDPQLAAADVASARVKAYFGDLILRKRAD
PRRDIVSTLVGAHSDDADTLSDAELISMLWGMLLGGFATTAATIDHAVLAMLAYPEERHWLQGDAAGVEAFVE
EVLRCEAPAMFSSIPRIAQRDIELHGVVIPKDADVRVLIAAGNRDPDAFADPDRFDPVRFYGTRPGMSSDGKI
MLSFGHGIHFCLGAQLARVQLTESLPQIQARFPTLALAEQPTRERSAFLRTFRALPVRLRAQAAAEVRVVVDQ
DLCGTTGQCVLTLQGAFRQREPDGVAEVCMATVPQALHAAVRLAASQCPVAAIG

>CYP114A7(SAMCFNEI73_pB0339)*Sinorhizobium americanum*

MRWLREHDPVHRAASGLFLLSRHADIYWALKATGDALRGPAPGELARYFPRAATSLSLNLLASTLAMKDPPTH
TRLRRLISRDFTMGQIDNLRPSIARNVAARLDGMAPALERGEAVDLHREFALALPMLVFAELFGMAQDDMFGL
AAGIGAILEGLSPHASDPQLAAADAASARVQAYFGDLIQRKRTDPLHDIVSMLVGAHDDDADTLSDAELISML
WGMLLGGFATTAATIDHAVLALLAYPEQRHWLQGDAVGIEAFVEEVLRCDAPAMFSSIPRIAQRDIELNGVVI
PKNADVRVLIAAGNRDPDAFADPDRFDPARFYGTSPGMSTDGKIMLSFGHGIHFCLGAQLARVQLAESLPRIE
ARFPTLVLAGKPTREPSAFLRTFRSLPVRLHAQAGG

>CYP114A8(BJ6T_77050)*Bradyrhizobium japonicum* USDA 6

MDVQETTAACRDAFAELASPACIHDPYPFMRWLREHDPVHRAASGLFLLSRHADIYWAFKATGDAFRGPAPGE
LARYFSRAATSPSLNLLASTLAMKDPPTHTRLRRLISRDFTMGQIDNLRPSIARIVAARLDGITPALERGEAV
DLHREFALALPMLVFAELFGMPQDDMFELAAGIGTILEGLGPHASDPQLAAADAASARVQAYFGDLIQRKRTD
PRRDIVSMLVGAHDDDADTLSDAELISMLWGMLLGGFVTTAASIDHAVLAMLAYPEQRHWLQADAARVRAFVE
EVLRCDAPAMFSSIPRIAQRDIELGGVVIPKNADVRVLIASGNRDPDAFADPDRFDPARFYGTSPGMSTDGKI
MLSFGHGIHFCLGAQLARVQLAESLPRIQARFPTLAFAGQPTREPSAFLRTFRTLPVRLHAQGS

>CYP114A8(RN69_37415)*Bradyrhizobium japonicum* E109

MDVQETTAACRDAFAELASPACIHDPYPFMRWLREHDPVHRAASGLFLLSRHADIYWAFKATGDAFRGPAPGE
LARYFSRAATSPSLNLLASTLAMKDPPTHTRLRRLISRDFTMGQIDNLRPSIARIVAARLDGITPALERGEAV
DLHREFALALPMLVFAELFGMPQDDMFELAAGIGTILEGLGPHASDPQLAAADAASARVQAYFGDLIQRKRTD
PRRDIVSMLVGAHDDDADTLSDAELISMLWGMLLGGFVTTAASIDHAVLAMLAYPEQRHWLQADAARVRAFVE
EVLRCDAPAMFSSIPRIAQRDIELGGVVIPKNADVRVLIASGNRDPDAFADPDRFDPARFYGTSPGMSTDGKI
MLSFGHGIHFCLGAQLARVQLAESLPRIQARFPTLAFAGQPTREPSAFLRTFRTLPVRLHAQGS

>CYP114A8(blr_2145)*Bradyrhizobium diazoefficiens* USDA 110

MDVQETTAACRDAFAELASPACIHDPYPFMRWLREHDPVHRAASGLFLLSRHADIFWAFKATGDAFRGPAPGE
LARYFSRAATSPSLNLLASTLAMKDPPTHTRLRRLISRDFTMGQIDNLRPSIARIVAARLDGITPALERGEAV
DLHREFALALPMLVFAELFGMPQDDMFELAAGIGTILEGLGPHASDPQLAAADAASARVQAYFGDLIQRKRTD
PRRDIVSMLVGAHDDDADTLSDAELISMLWGMLLGGFVTTAASIDHAVLAMLAYPEQRHWLQADAARVRAFVE
EVLRCDAPAMFSSIPRIAQRDIELGGVVIPKNADVRVLIASGNRDPDAFADPDRFDPARFYGTSPGMSTDGKI
MLSFGHGIHFCLGAQLARVQLAESLPRIQARFPTLAFAGQPTREPSAFLRTFRTLPVRLHAQGS

>CYP114A9(Mesau_05697)*Mesorhizobium australicum*

MDVQETKTACRDAFAELASPACIHDPYPFMRWLREHDPVHRAASGLFLLSRHADIYWALTATGDAFRGPAPGE
LARYFPRAATSLSLNLMASTIAMKDPPTHTRLRRLISRDFTMRQIDNLRPSIARIVAARLDGMAPALERGEAV
DLHREFALALPMLVFAELFGLPQDDMFGLAAGIGAIVEGLSPHASDPQLAAADAGSARVQAYFGDLIQRKRTD
PRHDIVSMLVGAHDDDADTLSDAELISMLWGMLLGGFTTTAAAIDHAVLAMLAYPEQRHWLQGDAVEVKAFVE
EVLRRDAPVLFSATPRIAQRDIELGGVVIPKNADVRVLIAAGNRDPDGFADPDRFDPARFYGTNPGMSTDGKI
MLSFGHGIHFCPGAQLARMQLAESLPRIQARFPTLALAEQPTREPSAFLRTFLALPVRLNAQAGG

>CYP114A9(Mesop_6228)*Mesorhizobium opportunistum*

MDVQETKTACRDAFAELASPACIHDPYPFMRWLREHDPVHRAASGLFLLSRHADIYWALTATGDAFRGPAPGE
LARYFPRAATSLSLNLMASTIAMKDPPTHTRLRRLISRDFTMRQIDNLRPSIARIVAARLDGMAPALERGEAV
DLHREFALALPMLVFAELFGLPQDDMFGLAAGIGAIVEGLSPHASDPQLAAADAGSARVQAYFGDLIQRKRTD
PRHDIVSMLVGAHDDDADTLSDAELISMLWGMLLGGFTTTAAAIDHAVLAMLAYPEQRHWLQGDAVEVKAFVE
EVLRRDAPVLFSATPRIAQRDIELGGVVIPKNADVRVLIAAGNRDPDGFADPDRFDPARFYGTNPGMSTDGKI
MLSFGHGIHFCPGAQLARMQLAESLPRIQARFPTLALAEQPTREPSAFLRTFLALPVRLNAQAGG

>CYP114A9(Mesci_5645)*Mesorhizobium cicero*

MDVQETKTACRDAFAELASPACIHDPYPFMRWLREHDPVHRAASGLFLLSRHADIYWALTATGDAFRGPAPGE
LARYFPRAATSLSLNLMASTIAMKDPPTHTRLRRLISRDFTMRQIDNLRPSIARIVAARLDGMAPALERGEAV
DLHREFALALPMLVFAELFGLPQDDMFGLAAGIGAIVEGLSPHASDPQLAAADAGSARVQAYFGDLIQRKRTD
PRHDIVSMLVGAHDDDADTLSDAELISMLWGMLLGGFTTTAAAIDHAVLAMLAYPEQRHWLQGDAVEVKAFVE
EVLRRDAPVLFSATPRIAQRDIELGGVVIPKNADVRVLIAAGNRDPDGFADPDRFDPARFYGTNPGMSTDGKI
MLSFGHGIHFCPGAQLARMQLAESLPRIQARFPTLALAEQPTREPSAFLRTFLALPVRLNAQAGG

>CYP114A9(LMTR13_26570)*Bradyrhizobium icense*

MDVQETTAACRDAFAELASPACIHDPYPFMRWLREHDPVHRAASGLFLLSRHADIYWALKATGDAFRGPAPGE
LARYFPRAATSLSLNLLASTLAMKDPPTHTRLRRLISRDFTMRQIDNLRPSIARIVAARLDGMAPALERGEAV
DLHREFALVLPMLVFAELFGMPQDDMFGLAAGIDAILEGLSPHASDPQLAAADAASARVQAYFGDLIQRKRTG
PRHDIVSMLVGAHDDDADTLSDAELISMLWGMLLGGFATTAATIDHAVLAMLAYPEQRHWLQGDAVGVKAFVE
EVLRCDAPAMFSSIPRIAQRDIELGGVVIPKNADVRVLIAAGNRDPHAFADPDRFDPARFYGTSPGMSTDGKI
MLSFGHGIHFCLGAQLARVQLAECLPRIQARFPTLALAEQPTREPSAFLRTFRALPVRLHAQGAEMRVVVDQD
LCGTTGQCVLTLPGTFRQREPDGVAEVCGATVPQALHAAVRLAASQCPVAAIRVIESDAGDDGRASADPAPSP
AEAERHAAKDQRNPGGHDGTV

>CYP114A10(RGR602_PB00447)*Rhizobium gallicum*

MDVKETTAVCRDAFAELASPACIHDPYPFMRWLREHDPVHRATSGLFLLSRHADIYWAFTATGDAFRGPAPGE
LARYFPRAATSLSLSLLASTLAMKEPPTHTRLRRLISRDFTMREIDNLRPSIARIVAARLDGIAPALERGEAV
DLHREFALALPMLVFAELFGMPQEDMFGLAAIIGAILEGLSPHASDSQLAAADAASAKVKAYFGDLIQRKRTD
PRQDIVSMLVGAHGDDADMLSDAELISMLWGMLLGGFTTTAATIDHAVLAMLAYPEQRHWLQGDAMGIEAFVE
EVLRCDAPAMFSSIPRIAQRDIELCGVVIPKDADVRVLIAAGNRDPDAFADPDRFDPARFYGTSPGMSTDGKI
MLSFGHGIHFCLGAQLARVQLAESLPRIEARFPTLVLAEKPTREPSAFLRTFRALPVRLNALGEA

>CYP114A11(RTCIAT899_PB00450)*Rhizobium tropici*

MDVKETTAVCRDAFAELASPACIHDPYPFMRWLREHDPVHRAASGLFLLSRHADIYWALKATGDAFRGPAPGE
LARYFPRAATSLSLNLLASTLAMKDPPTHTRLRRLISRDFTMREIDNLRPSIVHIVAARLDGMAHALERGEAV
DLHREFALALPMLVFAELFGIPQEDMFGLAANIGAILEGLSPHASDPQLAEADAASAKVKAYFGDLIQRRRTD
PRQDVVSMLVGAHGDDADMLSDVELISMLWGMLLGGFATTAATIDHAVLAMLAHPEQRHWLQGDAKGVEAFVE
EVLRCNAPAMFSSIPRIAQRDIELGGVVIPKNADVRVLIAAGNRDPDAFADPDRFDPVRFYGTSPGMSIDGKI
MLSFGHGIHFCLGAQLARVQLAESLPRIEARFPTLVLADQPTLEPSAFLRTFRALPVRLHAQAGR

>CYP114A12(REMIM1_PE00082)*Rhizobium etli*

MDVQETTAVCKDAFAELASPACIQNPYPFMRWLREHDPVHRSASGLFLLSRHADIYWALKATGDEFRGPAPGE
LARYFPRAATSLSLNLLASTLAMKEPPTHTRLRRLISRDFTMRQIDNLRPSIARIVAARLDGMAPALERGEAV
DLHREFALALPMLVFAELFGMPQEDIFELSAVISTILEGLSPHASDLQLAAADAASARVQAYFGDLILRKRTD
PHQDIVSTLVSAHSDDSDTLSDAELISMLWGMLLGGFATTAATVDHAVLAMLAYPEQRHWLQGDAAGVEAFVE
EVLRCEAPAMFSSIPRIAQRDIELRGVVIPKGADVRVLMAAGNRDPDAFADPDRFDPARFHGTRPGMSNDGQI
MLSFGHGIHFCLGAQLARVQLAESLPQIQARFPTLALAEQPTREPSAFLRTFRALPVRLHAHGAAEVHVVVDQ
DLCGTTGQCVLTLPGAFRQRESDGVAEVSVATVPQALHAAVRLAASQCPVAAIRVIESDAGNDHRTISDSAPS
PEEAARHVAKDLRNPGGHDGTI

>CYP114A12(IE4771_PB00364)*Rhizobium_etli* bv. mimosae IE4771

MDVQETTAVCKDAFAELASPACIQNPYPFMRWLREHDPVHRSASGLFLLSRHADIYWALKATGDEFRGPAPGE
LARYFPRAATSLSLNLLASTLAMKEPPTHTRLRRLISRDFTMRQIDNLRPSIARIVAARLDGMAPALERGEAV
DLHREFALALPMLVFAELFGMPQEDIFELSAVISTILEGLGPHASDLQLAAADVASARVQAYFGDLILSKRTD
PHQDIVSTLVGAHSDDSDTLSDAELISMLWGMLLGGFATTAATVDHAVLAMLAYPEQRHWLQGHAAGVEAFVE
EVLRCEAPAMFSSIPRIAQRDIELRGVVIPKGADVRVLMAAGNRDPDAFADPDRFDPARFHGTSPGMSSDGQI
MLSFGHGIHFCLGAQLARVQLAESLPQIQARFPTLALAERPTREPSAFLRTFRALPVRLHSQGAAEVHVVVDE
DLCGTTGQCVLTLPGAFRQRESDGVAEVSVATVPQALHAAVRLAANQCPVAAIRVIESNAGDDNRNNDPAPSP
AEVERHAAKDLRNPGGHDGTI

>CYP114A13(C770_GR4pB021)*Sinorhizobium meliloti* GR4

MDVPETTAACRDAFAEMASPTCIHDPYPFMRWLREHDPVHRAESGLFLLSRHADIHWALKATGDAFRGPTPGE
LARYFPRAATSLSLNLLASTLAMKDPPTHTRLRRLISRDFTRRQIDNLRPSIARIVAARLDGMASTLERGEVV
DLHREFALALPMLVFAELFGLPQDDMFGLAAGIGAILEGLSPHASDPQLAAADAASARVQAYFGDLIQRKRTD
PCHDIVSMLVGAHDDDADTLSDAELISMLWGMLLGGFATTAATIDHAVLAMLAYPEQRRWLQGDAVGASERAR
NNSGQSYWLRGLHGPPFAVVNRYRVYLK

>CYP115A2v1(mll_6125)*Mesorhizobium loti* MAFF 303099

MPAAPTQLDRLSSAILRQGGMARVSLPGDVVTWAAARHQTLRQMLSDQRFNKDWRQWRALQDGEIPEDHPLIG
ICKVDNMTTAHGADHRRLRGLLSSSFAPSRIALLAPRVEQCVDRLLAEMAQRGGSADLMSEFAAPLPTNVIAE
LFGLPDEQREEIVALTYSLASTSATAEEVRQTRQRIPEFFRRLIALKRGQLGDDLASALIVARDKGELVSDTE
LIDMLFMVLSAGFVTTAGVIGNGVLALLTHPQQLHLVRSGQVPWSQAIEEILRWGTSAANLPFRYATQDVEID
GCLVRRGDAVLMAFHAANRDEKAFGPGANRFDVTRRHNPHLSFGEGPHSCLGAALARLELRCAFPPLFGRLED
LALTIAAEDVVYMPSYVIRCPQRLPVSFRPSVA

>CYP116B183(IMCC20628_00551)*Hoeflea* sp. IMCC20628 Hoeflea sp. IMCC20628

MTDDKAAVCPAHGRADGCPVSPQAAAFDPFAPAYLANPAEALRWSREQEPVFYSPQLGYWVVSRYDDVKAVFR
DNDTFSPSIALERVVPPSQEAVETLKRYNYAMNRTLVNEDEPAHMERRRLLMDHFLPANLAEHEAMIRRLTRE
KVDAFVEKGRVDLVEAMLWEIPLTVALHFLGVPKEDMDSLKEFSVAHTVNTWGKPTPEQQVAVADTVGRFWQF
AGKVLDKMRKEPDGTGWMHDAIRKNADYPDIVTDSYLHSMMMAIIVAGHETTSLASTNALKLLLSNRLVWERI
CKDPALIPNAVEECLRLEGSVVAWRRKATRETEISGVTIPKDAKLLIVSGSANHDRRHFENGDFLDIYRDSAV
EHLTFGYGSHQCMGKNFARLEMRIFIEELTRRLPHMQLVADQQFTYLPSLSFRGPDRLLVEWDPANNPEHADP
SLAASSISFPVGAPSAKEIARSVRVAETKLIAEGVKLVRLEDPDGLPLPPWTPGAHIDVVFDEFSRKYSLCGD
PSDPLYSIAVLRENEGRGGSRFVHDQLVPGMTLHMRGPKNYFRLEEDADRHILIAGGIGITPIVAMADRLKAS
GCDYEVHYAGRSRDRMAFLDRMVADHGANVVLHVSGEGTRAQMNSIVDGWSVGTRIHACGPRGLLGELESLCT
HLPEGAFKCEAFSTDAGSGAHADDTAFVVELKDSDLTLTVPTGSTLLETVRAAGIDVPSDCEEGLCGSCEVRV
LEGAIDHRDKVLSSAERAVSDRMMACCSRAAGGKLKLAL

>CYP117A2(NGR_a02740)*Sinorhizobium fredii* NGR234

MNVLLNPLNRRHRLRYDIPVMPGAFPLVGHLPAIVCDLPRLLRRAERTLGSHFWLDFGPAGHLMTCVDPHAFA
LLRHKDVSSALIEEIAPELLGGTLVAQDGGAHRQARDAIKAAFLPEGLTQAGIGDLFAPVIRARVQAWRDRGD
VTILPETGDLMLKLIFTLMGVPAQDLPGWHRKYRQLLQLIVAPSVDLPGLPLRRGRAARDWIDAQLRQFVRDA
RAHAARTGLINDMVSAFDRSDDALSDDLLVANIRLLLLAGHDTTASTMAWMVIELARQPMLWDALVEEAQRVG
AVPTRHADLEQCPVAEALFRETLRVHPATTLLPRRALQELQLGQRRIPAGTHLCIPLLHFSTSALLHEAPDQF
RLARWLQRTEPIRPVDMLQFGTGPHVCIGYHLVWLELVQFSIALALTMHKAGVRPLLLSGVEKGRRYYPTAHP
SMTIRIGFS

>CYP117A2(A6B35_33030)*Mesorhizobium amorphae*

MDMLLNPLNRRHRLRYDIPVVPGAFPLVGHLPAIVGDLPRLLRRAERTLGSHFWLDFGPAGQLMTCVDPAAFA
LLRHKDVSSALIEEIAPELLGGTLVAQDGGAHRQARDAIKAAFLPKGLTQAGIGDLFAPVIRARVQAWRDRGD
VTILRETGDLMLKLIFSLMGIPAQDLPGWHRKYRQLLQLIVAPPIDLPGLPLRRGRAARDWIDAQLRQFVRDA
RAHAARTGLINDMVSAFDRSDDALSDDVLVANIRLLLLGGHETTASTMAWMVIELARQPVLWDALVEEAQRVG

AVPTRHADLAQCPVAEALFRETLRLHPAVTLLPRRALQELQLGQRRIPAGTHLCIPLLHFSTSALLHEAPDQF
RLARWLQRTEPIRPVDMLQFGTGPHVCIGYHLVWLELVQFCIALALTMHKAGVRPRLLSGVEKGRRYYPTAQP
SMTIRIGFS

>CYP117A3(mlr_6367)*Mesorhizobium loti* MAFF 303099

MDMLLNPLDRRHRLRDDIPVVPGAFPLVGHLPAIVCDLPRLLRRAERTLGSHFWLDFGPAGHLMTCVDPDAFA
LLRHKDVSSALIEEIAPELLGGTLVAQDGGAHRQARDAIKAAFLPKGLTQAGIGNLFAPVIQARVQAWRDRGD
VTILRETGDLMLKLIFSLMGIPAQDLPGWHRKYRQLLQLIVAPPVDLPGLPLRRGRAARDWIDAQLRQFVRDA
RAHAARTGLINDMVSSFDRGDDALSDDVLVANIRLLLLAGHDTTASTMAWMVIELARQPGLWDALVEEAQRVG
AVPTRHADLAQCPVAEALFRETLRVHPATTLLPRRALQELQLGQRRIPAGTPLCIPLLHFSTSALLHEAPDQF
RLARWLQRTEPIRPVDMLQFGTGPHVCIGYHLVWLEMVQFCIALALTMHKAGVRPRLLSAVEKGRRYFPTAHP
SMKIRIGFS

>CYP117A3(A9174_30935)*Mesorhizobium loti* NZP2037

MDMLLNPLDRRHRLRDDIPVMPGAFPLVGHLPAIVCDLPRLLRRAEWTLGSHFWLDFGPAGHLMTCVDPDAFA
LLRHKDVSSALIEEIAPELLGGTLVAQDGGAHRQARDAIKAAFLPKGLTQAGIGNLFAPVIQARVQAWRDRGD
VTILRETGDLMLKLIFSLMGIPAQDLPGWHRKYRQLLQLIVAPPVDLPGLPLRRGRAARDWIDAQLRQFVRDA
RAHAARTGLINDMVSAFDRGDDALSDDVLVANIRLLLLAGHDTTASTMAWMVIELARQPGLWDALVEEAQRVG
AVPTRHPDLAQCPVAEALFRETLRVHPATTLLPRRALQELQLGQRRIPAGTPLCIPLLHLSTSALLHEAPDQF
RLARWLQRTEPIRPVDMLQFGTGPHVCIGYHLVWLEMVQFCIALALTMHKAGVRPRLLSAAEKGRRYFPTAHP
SMKIRIGFS

>CYP117A3(LMTR13_26560)*Bradyrhizobium icense*

MDMLLNPLDRRHRLRHDIPVVPGAFPLVGHLPAIVCDLPRLLRRAERTLGSHFWLDFGPAGHLMTCLDPDAFA
LLRHKDVSSALIEEIVPELFGGTLVAQDGGAHRQARDAIQAAFLPKGLTQAGIGDLFAPVIRARVQAWRDRGD
VTILRETTDLMLKLIFSLMGIPAQDLPGWHRKYRQLLQLIVAPPVDLPGLPLRRGRAARDWIDAQLRQFVRDA
REHAARTGLINDMVSAFDRSDDALSDDVLVANIRLLLLGGHDTTASTMAWMVIELARQPGLWDALVEEAQRVG
AVPTRHADLAQCPVAEALFRETLRVHPATTLLPRRALQELQLGQRRIPAGTHLCIPLLHFSTSALLHEAPDQF
RLARWLQRTEPIRPVDMLQFGTGPHVCIGYHLVWLELVQFCIALALTMHKAGVRPRLASGVEKGRRYYPTAHP
SMTIRIGFS

>CYP117A4(RHE_PD00249)*Rhizobium etli* CFN 42

MDMLLNPLNRWRRLRDDIPVMPGAFPLVGHLPAIVCDLPRLLRRAERTLGSHFWLDFGPAGHLMTCLDPDALA
LLRHKEVSSALIEEMAPDILGGTLVTLDGSAHRQARDGIKAAFLPRGLTEAGIGELFEPIIRAQVKAWRDRGE
VAILPDTRNLMLKLTFSLMGIPAQDLSEWHRKYRQLLQLMVAPPIDLPGMPLRRGRAARDWIDAQSRQFIRDA
RARAARTGLINDMVSAFDCSDGALSDDVLVANIRLLLLAGHETSASTIAWMVIELAQHPELWDALVEEAQRVG
AVPTGHEDLAQCPVAEALFRETLRMHPASSLVPRRAMQELQLGQRRIPSGTHLCIPLLHFSTSPLLHEAPDQF
RLGRWLQRTEPIRPVDMLQFGAGPHVCMGYHLVWLELVQFSIALALTMQEAGVRPRLMSGVEKGRRYYPTAHP
SMTVRIGFS

>CYP117A4(AMK02_PC00296)*Rhizobium* sp. N731

MDMLLNPLNRWRRLRDDIPVMPGAFPLVGHLPAIVCDLPRLLRRAERTLGSHFWLDFGPAGHLMTCLDPDALA
LLRHKEVSSALIEEMAPDILGGTLVTLDGSAHRQARDGIKAAFLPRGLTEAGIGELFEPIIRAQVKAWRDRGE
VAILPDTRNLMLKLTFSLMGIPAQDLSEWHRKYRQLLQLMVAPPIDLPGMPLRRGRAARDWIDAQSRQFIRDA
RARAARTGLINDMVSAFDCSDGALSDDVLVANIRLLLLAGHETSASTIAWMVIELAQHPELWDALVEEAQRVG
AVPTGHEDLAQCPVAEALFRETLRMHPASSLVPRRAMQELQLGQRRIPSGTHLCIPLLHFSTSPLLHEAPDQF
RLGRWLQRTEPIRPVDMLQFGAGPHVCMGYHLVWLELVQFSIALALTMQEAGVRPRLMSGVEKGRRYYPTAHP
SMTVRIGFS

>CYP117A4(AMC79_PC00278)*Rhizobium phaseoli*

MDMLLNPLNRWRRLRDDIPVMPGAFPLVGHLPAIVCDLPRLLRRAERTLGSHFWLDFGPAGHLMTCLDPDALA
LLRHKEVSSALIEEIAPDILGGTLVTLDGSAHRQARDGIKAAFLPRGLTEAGIGELFEPIIRAQVKAWRDRGE

VAILPDTRNLMLKLTFSLMGIPAQDLSEWHRKYRQLLQLMVAPPIDLPGMPLRRGRAARDWIDAQSRQFIRDA
RARAARTGLINDMVSAFDCSDGALSDDVLVANIRLLLLAGHETSASTIAWMVIELAQHPELWDALVEEAQRVG
AVPTGHEDLAQCPVAEALFRETLRMHPASSLVPRRAMQELQLGQRRIPSGTHLCIPLLHFSTSPLLHEAPDQF
RLGRWLQRTEPIRPVDMLQFGAGPHVCMGYHLVWLELVQFSIALALTMQEAGVRPRLMSGVEKGRRYYPTAHP
SMTVRIGFS

>CYP117A4(IE4803_PB00396)*Rhizobium etli* bv. Phaseoli IE4803

MDMLLNPLNRWRRLRDDIPVMPGAFPLVGHLPAIVCDLPRLLRRAERTLGSHFWLDFGPAGHLITCLDPDALA
LLRHKEVSSALIEEMAPDILGGTLVTLDGSAHRQARDGIKAAFLPRGLSEAGIGELFEPIIRAQVKAWRDRGE
VAILPDTRNLMLKLTFSLMGIPAQDLSEWHRKYRQLLQLMVATPIDLPGMPLRRGRAARDWIDAQSRQFIRDA
RAPAARTGLINDMVSAFDCSDGALSDDVLVANIRLLLLAGHETSASTMAWMVIELAQHPELWDALVEEAQRVG
AVPTGHEDLAQCPVAEALFRETLRMHPASSLVPRRAMQELQLGPRRIPAGTHLCIPLLHFSTSPLLHEAPDPF
RLGRWLQRTEPIRPVDMLQFGAGPHVCMGYHLVWLELVQFSIALALTMQEAGVRPRLMSGVEKGRRYYPTASV
HDNPHRILMSWDPLPRVKRQRRRR

>CYP117A4(AMJ98_PC00301)*Rhizobium* sp. N1341

MDMLLNPLNRWRRLRDDIPVMPGAFPLVGHLPAIVCDLPRLLRRAERTLGSHFWLDFGPAGHLITCLDPDALA
LLRHKEVSSALIEEMAPDILGGTLVTLDGSAHRQARDGIKAAFLPRGLSEAGIGELFEPIIRAQVKAWRDRGE
VAILPDTRNLMLKLTFSLMGIPAQDLSEWHRKYRQLLQLMVATPIDLPGMPLRRGRAARDWIDAQSRQFIRDA
RAPAARTGLINDMVSAFDCSDGALSDDVLVANIRLLLLAGHETSASTMAWMVIELAQHPELWDALVEEAQRVG
AVPTGHEDLAQCPVAEALFRETLRMHPASSLVPRRAMQELQLGPRRIPAGTHLCIPLLHFSTSPLLHEAPDPF
RLGRWLQRTEPIRPVDMLQFGAGPHVCMGYHLVWLELVQFSIALALTMQEAGVRPRLMSGVEKGRRYYPTASV
HDNPHRILMSWDPLPRVKRQRRRR

>CYP117A4(RHECIAT_PB0000288)*Rhizobium etli* CIAT 652

MDMLLNPLNRWRRLRDDIPVMPGAFPLVGHLPAIVCDLPRLLRRAERTLGSHFWLDFGPAGHLMTCLDPDALA
LLRHKEVSSALIEEMAPDILGGTLVTLDGSAHRQARDGIKAAFLPRGLTEAGIGELFEPIIRAQVKAWRDRGE
VAILPDTRNLMLKLTFSLMGIPAQDLSEWHRKYRQLLQLMVAPPIDLPGMPLRRGRAARDWIDAQSRQFIRDA
RARAARTGLINDMVSAFDCSDGALSDDVLVANIRLLLLAGHETSASTIAWMVIELAQHPELWDALVEEAQRVG
AVPTGHEDLAQCPVAEALFRETLRMHPASSLVPRRAMQELQLGQRRIPSGTHLCIPLLHFSTSPLLHEAPDQF
RLGRWLQRTEPIRPVDMLQFGAGPHVCMGYHLVWLELVQFSIALALTMQEAGGAAAIDERRRKRPALLPDRTS
VHDSPHRILMSWDPLPRVKRQRRRRWAALLHSARARGATPAAPRRLAARRGILCQVEGDEQHAEGFHAKRRPN
WRFPVRRIGRAGVRRIAGARVHRHWRAMRRRGGCRALLCTAHWIAIAPVSALYCRWSRMTRQRMTRRWASPRQ
ACGGEESNRRADHEAACSAPVSAGPMARGRGSHHLAAPACGAVGAGRAAGQKGICSSTRRAHWRRSARRRRRT
AAIGRRHR

>CYP117A7(Mesci_5648)*Mesorhizobium cicero*

MLLNPLNRRHRLRHDIPVVPGAFPLVGHLPAIICDLPRLLRRAERTLGSYFWLDFGPAGHLMTCMDPDAFALL
RHKDVSSALIEGIAPELLGGTMVAQDGGAHRQARDAIKPAFLPKGLTQAGIGDLFEPVIRARVQAWRDRGDVA
ILRETGDLMLKLIFSLMGIPAQDLAGWHRKYRQLVQLIVAPPIDLPGLPLRRGRAARDWIDAQLHQFVRDARA
HAARTGLIKDMVSAFDRSDDALSDDVLVANIRLLLLGGHETTASTMAWMVIELARQPVLWDTLVEEAQRVGAV
PTRHADLAQCPVAEALFRETLRLHPAVTLLPRQALQELQLGQWRIPAGTHLCIPLLHFSTSALLHEAPDQFRL
ARWLQRTEPIRPVDMLQFGTGPHVCIGYHVVWLELVQFCIALALTMHKAGVRPRLLSDVEKGRRYYPTANPSM
KIRIGFS

>CYP117A7(Mesop_6231)*Mesorhizobium opportunistum*

MLLNPLNRRHRLRHDIPVVPGAFPLVGHLPAIICDLPRLLRRAERTLGSYFWLDFGPAGHLMTCMDPDAFALL
RHKDVSSALIEGIAPELLGGTMVAQDGGAHRQARDAIKPAFLPKGLTQAGIGDLFEPVIRARVQAWRDRGDVA
ILRETGDLMLKLIFSLMGIPAQDLAGWHRKYRQLVQLIVAPPIDLPGLPLRRGRAARDWIDAQLHQFVRDARA
HAARTGLIKDMVSAFDRSDDALSDDVLVANIRLLLLGGHETTASTMAWMVIELARQPVLWDTLVEEAQRVGAV
PTRHADLAQCPVAEALFRETLRLHPAVTLLPRQALQELQLGQWRIPAGTHLCIPLLHFSTSALLHEAPDQFRL
ARWLQRTEPIRPVDMLQFGTGPHVCIGYHVVWLELVQFCIALALTMHKAGVRPRLLSDVEKGRRYYPTANPSM
KIRIGFS

>CYP117A7(Mesau_05700)*Mesorhizobium australicum*

MLLNPLNRRHRLRHDIPVVPGAFPLVGHLPAIICDLPRLLRRAERTLGSYFWLDFGPAGHLMTCMDPDAFALL
RHKDVSSALIEGIAPELLGGTMVAQDGGAHRQARDAIKPAFLPKGLTQAGIGDLFEPVIRARVQAWRDRGDVA
ILRETGDLMLKLIFSLMGIPAQDLAGWHRKYRQLVQLIVAPPIDLPGLPLRRGRAARDWIDAQLHQFVRDARA
HAARTGLIKDMVSAFDRSDDALSDDVLVANIRLLLLGGHETTASTMAWMVIELARQPVLWDTLVEEAQRVGAV
PTRHADLAQCPVAEALFRETLRLHPAVTLLPRQALQELQLGQWRIPAGTHLCIPLLHFSTSALLHEAPDQFRL
ARWLQRTEPIRPVDMLQFGTGPHVCIGYHVVWLELVQFCIALALTMHKAGVRPRLLSDVEKGRRYYPTANPSM
KIRIGFS

>CYP117A8(blr_2147)*Bradyrhizobium diazoefficiens* USDA 110

MDMLLNPLNRRHRLRHDIPVVPGAFPLVGHLPAVVCDLPRLLRRAERTLGSHFWLDFGPAGHLMTSLDPDALA
LLRHKDVSSGLIEDIAPELFGGTLVAQDGIAHRQARDAIQAALLPKGLTLAGIGELFAPVIRARVQRWRERGD
VTILRETGDLMLKLIFSLMGIPAQDLPGWHRKYRQLLQLIVAPPVDLPGLPLRRGRAARDWIDARLREFVRAA
REHASRTGLINDMVSAFDRSDDALSDDVLVANIRLLLLGGHDTTASTMAWMVIELARQPGLWDALVEEAQRVG
AVPTRHADLAQCPVAEALFRETLRVHPATPLLVRRALRELRIGQQRIPTGTDLCIPLLHFSTSALLHEAPDQF
RLARWLQRTEPIRPVDMLQFGTGPHFCMGYHLVWLELVQFCIALALTMHEAGVRPRLLSGVEKGRRYYPTAHP
SMTIRIGFS

>CYP117A8(BJ6T_77020)*Bradyrhizobium japonicum* USDA 6

MDMLLNPLNRRHRLRHDIPVVPGAFPLVGHLPAVVCDLPRLLRRAERTLGSHFWLDFGPAGHLMTSLDPDALA
LLRHKDVSSGLIEDIAPELFGGTLVAQDGIAHRQARDAIQAALLPKGLTLAGIGELFAPVIRARVQRWRERGD
VTILRETGDLMLKLIFSLMGIPAQDLPGWHRKYRQLLQLIVAPPVDLPGLPLRRGRAARDWIDARLREFVRAA
REHASRTGLINDMVSAFDRSDDALSDDVLVANIRLLLLGGHDTTASTMAWMVIELARQPGLWDALVEEAQRVG
AVPTRHADLAQCPVAEALFRETLRVHPATPLLVRRALRELRIGQQRIPTGTDLCIPLLHFSTSALLHEAPDQF
RLARWLQRTEPIRPVDMLQFGTGPHFCMGYHLVWLELVQFCIALALTMHEAGVRPRLLSGVEKGRRYYPTAHP
SMTIRIGFS

>CYP117A8(RN69_37400)*Bradyrhizobium japonicum* E109

MDMLLNPLNRRHRLRHDIPVVPGAFPLVGHLPAVVCDLPRLLRRAERTLGSHFWLDFGPAGHLMTSLDPDALA
LLRHKDVSSGLIEDIAPELFGGTMVAQDGIAHRQARDAIQAALLPKGLTLAGIGELFAPVIRARVQRWRERGD
VTILRETGDLMLKLIFSLMGIPAQDLPGWHRKYRQLLQLIVAPPVDLPGLPLRRGRAARDWIDARLREFVRAA
REHASRTGLINDMVSAFDRSDDALSDDVLVANIRLLLLGGHDTTASTMAWMVIELARQPGLWDALVEEAQRVG
AVPTRHADLAQCPVAEALFRETLRVHPATPLLVRRALRELRIGQQRIPTGTDLCIPLLHFSTSALLHEAPDQF
RLARWLQRTEPIRPVDMLQFGTGPHFCMGYHLVWLELVQFCIALALTMHEAGVRPRLLSGVEKGRRYYPTAHP
SMTIRIGFS

>CYP117A9(SAMCFNEI73_pB0337)*Sinorhizobium americanum*

MLLNPLNRRHRLRYDIPVMPGAFPVVGHLPAIICDLPRLLRRAERTLGSHFWLDFGPAGHLMTCLDPDALALL
RHKDVSSALIEEIAPEILGRTLVTLNGSAHRQARDGIKAAFLPRGLTEAGIGELFEPAIWARVQAWRDRGEVT
ILRETADLMLKLTFSLMGIPAQDLPEWHRKYRQLLQLIVAPPVDLPGMPLRRGRAARDWIDAQSREFIRDARA
HAVRTGLINDMVSAFDRSEDALSDDVLVANIRLLLLAGHETTASTMAWMVIELARRPELWDALVEEAQRVGAV
PTRHADLAQCPVAEALFRETLRMHPASSLLPRRATQELQLGERRIPAGTSLCIPLLHFSTSALLHEAPDEFRL
ARWLQRTGPIGPVDMLQFGSGPHVCIGYHLVWLELVQFSIALALTMHKAGVRPRLLSDAEKGRRYYPTAQPSM
KIRIEFS

>CYP117A9(RGR602_PB00450)*Rhizobium gallicum*

MDMLLNPLNRRHRLRHDIPVMPGAFPMVGHLPAIVTDMPRLLRRAERTLGSHFWLDFGPAGQLMTCLDPDALA
LLRHKDVSSTLIAEIAPEILGGTLVTLNGSAHRQARDGIKAAFLPRGLTEAGIGELFAPVIRARVQAWRDRGE
ITILRETGDLMLKLTFYLMGIPAQDLPEWHRKYRQLLQLIVAPPVDLPGTPLRRGRAARDWIDAQSRQFIRDA
RAHSARTGLINDMVSAFDRSEDALSDDVLVANVRLLLLAGHETTASTMAWIVIELARQPELWDALVEEAQRVG
AVPTRHADLAQCPVAEALFRETLRMHPASSLVPRRATQELQLGQRRIPAGTSLGIPLLHFSTSALLHEAPDQF

RLARWLQRTGPIGPVDMLQFGSGPHVCIGYHLVWLELVQFSIALALTMHKAGVRPRLLSDAEKGRRYYPTAQP
SMKVRIGFS

>CYP117A9(RTCIAT899_PB00435)*Rhizobium tropici*

MLLNPLNRRHRLRDDIPVMPGAFPLVGHLPAIVCDLPRLLRRAERTLGSHFWLDFGPAGQLMTCLDPDAFALL
RHKDVSSALIEEIAPEILGGTLVTLNGSAHRQARDGIKSAFLPRGLTEAGIGELFEPVIRARVQAWRERGEVT
ILPETGDLMLKLTFYLMGIPAQDLPEWHRKYRQLLQLMLAPPIDLPGTPFRRGRAARDWIDAQSRQFIRGARA
HAARTGLINDMVSAFDRSEGALSDDVLVANVRLLLLAGHETTASTMAWMVIELARQPELWDSLVEEAQRVGAV
PTRHADLSQCPVAEALFRETLRMHPASSLLPRRATQELQLGQRRIPAGTSLGIPLLHFSTSPLLHEAPDQFRL
ARWLQRTGPIRPVDMLQFGSGPHVCIGYHLVWLELVQFGIALALTMHKAGVRPRLLSDAEKGRRYYPTAQPSM
KIRIGFS

>CYP117A10(IE4771_PB00366)*Rhizobium etli* bv. mimosae IE4771

MDMLLNPLNRWHRLRDDIPVMPGAYPLVGHLPAIVCDLPRLLQRAERTLGSHFWLDFGPAGHLMTCLDPDAFA
LLRYKEVSSALIEEMAPDILGGTLVTLNGSAHRQARDGIKAAFLPRGLTEAGIGELFEPVIRARVQAWRDRGE
VTILPETGDLMLKLTFSLMGIPARDLPEWHRKYRQLLQLMVAPPIDLPGMPLRRGRAARDWIDVQSRQFIRDA
RACAARTGLINDMVSAFDRSEDALSDDVLVANIRLLLLAGHETTASTMAWMVIELARQPELWDALVEEAQCVG
AVPTRHADLAQCPVAEALFRETLRMHPASSLVPRRATQELQLGQRRIPAGTRLGIPLLHFSTSALLHEAPDQF
RLTRWLERTGPIRPLDMLQFGSGPHVCIGYHLVWLELVQFSTALALTMHKAGVRPRLLSDAAKGRRYFPTAQP
SMKIRIGFS

>CYP117A10(REMIM1_PE00080)*Rhizobium etli*

MDVLLNPLNRWHRLRDDIPVMPGAYPLVGHLPAIVCDLPRLLRRAERTLGSHFWLDFGPAGHLMTCLEPDAFA
LLWHKEVSSALIEEMAPDILGGTLVTLNGSAHRQARDGIKAAFLPRGLTEAGIGELFEPVIRARVQAWRDRGE
VTILPETGDLMLKLTFSLMGIPARDLPQWHRKYRQLLQLMVAPPIDLPGMPLRRGRAARDWIDVQSRQFIRDA
RACAARTGLIDDMVSAFDRSEDALSDDVLVANIRLLLLAGHETTASTMAWMVIELARQPELWDALVEEAQCVG
AVPTRHADLAQCPVAEALFRETLRMHPASSLVPRRATQELQLGQRRIPAGTRLGIPLLHFSTSALLHEAPDQF
RLTRWLERTGPIRPLDMLQFGSGPHVCIGYHLVWLELVQFSTALALTMHKAGLRPRLLSDAEKGRRYFPTAQP
SMKIRIGFS

>CYP125P1(PHZ_c0594)*Phenylobacterium zucineum*

MEIDLLSPASFAGGQPHAQFAWLREHAPVFRHAEPDGPGFWAVTRHADVRAVDRDFQTFSSEPTVMIPDPAAE
AAAAFGPYKMMLMMDPPEHTAFRKLIRSEFTEPQARLRAERIRALARQIVDAVVHKGECDFVAEVAGEMPSFV
IAELMGLPLDDGRELYKLTETIHTAPEALPPGAGAAAVMKMFEYGAQVMAQKRARPGDDLASRLLACEVDGRR
LEDMEFLLFFLLLIDAGGDTTRNLLSGGLLALMEHPEQLAWLTADLPARLPAAREELLRYVSPVIYMRRTARR
DVELGGRRIAAGDKVVMYFGAANRDPAAIERPDALDLSREETAHLAFGNGPHVCLGQHIARVEIDAMLEEVLS
RMTDFAPAGEVEWLASNFISGPKVMPLRFRAAA

>CYP127A1(NGR_a01170)*Sinorhizobium fredii* NGR234

MSDLRRKRVKTNPIPDHVPPALVRHFSLFTSPGMAPTPNGDPHAAVACVHDDGPPIFYSPSNTRDGRGTWVIT
RARDQRRVLEDTETFSSHRSIFASALGEHWPVIPLELDPPAHGVFRALLNPLFSSRRVLALEPTIHARAGALI
DCIAKEKTSCDVMKDFALPFTFSVFLSFLGLSQRRSEVLVGWVSDLLHGNAEKRRAAARSVVAFIDEMAAMRR
KSPAVDFMTFVVQAKIEGRSLTEEEVRGIGVLFLVAGLDTVAAAIGFDMAYLARNPKHQELLRNEPARLGLAA
EELLRAYSTVQIIRVATKDIEFEGVPIREGDYVSCPAMIANRDPSEFKCPNTIDLARQDNQHTAFGYGPHLCH
GAHLARREIVIGLREWLARIPAFRIKEGTAPITHGGHVFGISNIILTWA

>CYP127A3v1 (mlr_5876)*Mesorhizobium loti* MAFF 303099

MAINPVPDHVPPEMVRDFSLFTSPGMPPTPNGDPHAAVACAHDGPPIFYSPYNTQDGRGTWVITRAADQRKVL
QDTETFSSHRSIFSSILGETWPTIPLELDPPAHGAFRSLLSPLLSPKRVTALEPAVRERAIALIDRITASATS
CDVMKDFAFPFTVSIFLRFLGLPDQGLDTFVGWAKDLLHGDDVERPVAARKIVAFIDELATNRRKDPVDDLMT
FIVQAQIEGRRLTDGEIRGIGVLVFVAGLDTVAAAIGFDLAYLARNLKDQELLRSEPARILLATEELLRAYPP

IQLIRVATKDIDFEGAPIRKGDYVSCATMIANRDPEEFESPNTVDLARDHNRHAAFGYGPHRCLGSHLARREI
VIGLEEWLARIPTFRIKEGTAPITCGGHVFGIENLILDWS

>CYP127A4(RHE_PD00215)*Rhizobium etli* CFN 42

MHLCSERIYRKRGTRENPMSTGRAGEASKKFRLRPTKQRGFRAARRSDRCIACHWRLALLRLEIWRSTILLAP
SPRRIRSRRRGFDDRRKAVATIRVPEHVPPEMVKDFSLFTSPGMERMPNGDPHAAVACLHNGPRIFYSPCNTR
DGRGTWVIVRAQDQRKLLQDTGTFSSHRSLFASALGENWPLIPLELDPPAHSVFRSLLNPLLSPRRIMELEPA
VRDRAIALISKISASSTSCDILTDFAFPFAVSIFLRLLGLSDERLNTFVGWGKDLLHGDGIRRTAAARTILAF
IDELAAMRRKEPADDFMTFVVQAKVDGRLLRDQEIHGIGVLLFVAGLDTVATAIGFDLAYLARNPTEQELLRS
KPDRIVLAAEELLRAYSTVQMIRVATKDINFEGAPIRKGDYISCATMIANRDPVEFENPNTIDLAREDNRHTA
FAYGPHRCLGSHLARREIIIGLEEWLSRIPDFRIKDGTAPITYGGHVFGMENLILDWS

>CYP127A4(AMJ98_PC00266)*Rhizobium* sp. N1341

MATIRVPEHVPPEMVKDFSLFTSPGMERMPNGDPHAAVACLHNGPRIFYSPCNTRDGRGTWVIVRAQDQRKLL
QDTGTFSSHRSLFASALGENWPLIPLELDPPAHSVFRSLLNPLLSPRRIMELEPAVRDRAIALISKISASSTS
CDILTDFAFPFAVSIFLRLLGLSDERLNTFVGWGKDLLHGDGIRRTAAARTILAFIDELAAMRRKEPADDFMT
FVVQAKVDGRLLRDQEIHGIGVLLFVAGLDTVATAIGFDLAYLARNPTEQELLRSKPDRIVLAAEELLRAYST
VQMIRVATKDINFEGAPIRKGDYISCATMIANRDPVEFENPNTIDLAREDNRHTAFAYGPHRCLGSHLARREI
VIGLEEWLSRIPDFRIKDGTAPITYGGHVFGMENLILDWS

>CYP127A4(AMC79_PC00243)*Rhizobium phaseoli*

MATIRVPEHVPPEMVKDFSLFTSPGMERMPNGDPHAAVACLHNGPRIFYSPCNTRDGRGTWVIVRAQDQRKLL
QDTGTFSSHRSLFASALGENWPLIPLELDPPAHSVFRSLLNPLLSPRRIMELEPAVRDRAIALISKISASSTS
CDILTDFAFPFAVSIFLRLLGLSDERLNTFVGWGKDLLHGDGIRRTAAARTILAFIDELAAMRRKEPADDFMT
FVVQAKVDGRLLRDQEIHGIGVLLFVAGLDTVATAIGFDLAYLARNPTEQELLRSKPDRIVLAAEELLRAYST
VQMIRVATKDINFEGAPIRKGDYISCATMIANRDPVEFENPNTIDLAREDNRHTAFAYGPHRCLGSHLARREI
VIGLEEWLSRIPDFRIKDGTAPITYGGHVFGMENLILDWS

>CYP127A4(AMK02_PC00261)*Rhizobium* sp. N731

MATIRVPEHVPPEMVKDFSLFTSPGMERMPNGDPHAAVACLHNGPRIFYSPCNTRDGRGTWVIVRAQDQRKLL
QDTGTFSSHRSLFASALGENWPLIPLELDPPAHSVFRSLLNPLLSPRRIMELEPAVRDRAIALISKISASSTS
CDILTDFAFPFAVSIFLRLLGLSDERLNTFVGWGKDLLHGDGIRRTAAARTILAFIDELAAMRRKEPADDFMT
FVVQAKVDGRLLRDQEIHGIGVLLFVAGLDTVATAIGFDLAYLARNPTEQELLRSKPDRIVLAAEELLRAYST
VQMIRVATKDINFEGAPIRKGDYISCATMIANRDPVEFENPNTIDLAREDNRHTAFAYGPHRCLGSHLARREI
VIGLEEWLSRIPDFRIKDGTAPITYGGHVFGMENLILDWS

>CYP127A4(RHECIAT_PB0000259)*Rhizobium etli* CIAT 652

MHLCSERIYRKRGTRENPMSTGRAGEASKKFRLRPTKQRGFRAARRSDRCIACHWRLALLRLEIWRSTILLAP
SPRRIRSRRRGFDDRRKAVATIRVPEHVPPEMVKDFSLFTSPGMERMPNGDPHAAVACLHNGPRIFYSPCNTR
DGRGTWVIVRAQDQRKLLQDTGTFSSHRSLFASALGENWPLIPLELDPPAHSVFRSLLNPLLSPRRIMELEPA
VRDRAIALISKISASSTSCDILTDFAFPFAVSIFLRLLGLSDERLNTFVGWGKDLLHGDGIRRTAAARTILAF
IDELAAMRRKEPADDFMTFVVQAKVDGRLLRDQEIHGIGVLLFVAGLDTVATAIGFDLAYLARNPTEQELLRS
KPDRIVLAAEELLRAYSTVQMIRVATKDINFEGAPIRKGDYISCATMIANRDPVEFENPNTIDLAREDNRHTA
FAYGPHRCLGSHLARREIVIGLEEWLSRIPDFRIKDGTAPITYGGHVFGMENLILDWS

>CYP127A5(BN877_p0289)*Rhizobium* sp. IRBG74

MKSNTIPDHVPRELVRDFSLFSSPGMAPTPNGDPHAAVACVHDGPPIFYSPSNTRDGRGTWVITRARDQRRVL
QDTRTFSSHRRIFASALGEHWPVIPLELDPPTHGVFRALLDPLFSSKRVIALEPTIRERARALIDRIARERTS
CDVLRDFALPFTASVFLSFLGLSQRRSEVFVGWVSDLLHGNADKRTAAARSIVGFIDEIAARRRRTPAFDFMT
FVVLAEIDGRSLTEEEVRGIGVLFLVAGLDTVAAAIGFDMAYLARNPKDQELLRNEPDRVMLAVEELLRAYST
VQMIRVAAKDIVFEGVPIRKGDYVSCATMIANRDPSEFECPETIDLARRGNHHTAFGYGPHLCLGAHLARREI
VVGLREWLARIPTFRIKEGTAPITHGGHVFGIKDLILTWT

>CYP127A6(SAMCFNEI73_pB0103)*Sinorhizobium americanum*

MACKPVPDHVPPEMVKDFSLFTSPGMERMPNGDPHAAVACLHDAPPIFYSPNNTRDGRGTWVITRAKDQRRVL
QDTATFSSHRSIFASALGENWPMIPLELDPPAHGVFRSLLNPLFSPKRVMVLEPVIRERAITLIDRILAASTS
CDVMKDFAFPFAVSIFLGFLGLSDKERDTFVGWGRDLLHGENVKRTAAALAIVGFIDELAAMRRKEPAGDFMS
FAVGAQVNGRPLTDEEVRGIGVLLFVAGLDTVATAIGFDLAHLARNLKDQQLLRSEPDRIGLASEELLRAYST
VQMIRVATKDIDFDGAPIRKGDYISCATMIANRDPEEFDCPNTIDLAREDNRHTAFAHGPHRCLGSHLARREI
VIGLEEWLSRIPDFRIKGGTAPITFGGHVFGIENLILEWS

>CYP127A7(RTCIAT899_PB00985)*Rhizobium tropici*

MGDPIPDHVPREMVSDFSLFTSPGMQRVSNGDPHGAAARVHAGPPVFYSLHNTRDGRGTWLRTRAKDQRKVLQ
DPDTFSSNRSIFASALGDDWPLIPLELDPPAHGIFRSLLNPLFSPKRVMALEASIRAQAITIVEKISVSGKSC
EVMKDFAIPFTVGVFLQFLGLPNERLDVFVGWAKALLHGDKVQRSAAARSILEFIDELAALRRKEPAGDFMTF
VVQAQIDSRLLAENEVRGIGVLLFVAGLDTVAAAIGFDLAHLARNPKDQNLLRSEPERIVLATEELLRAYSTV
QMIRVATKDIDFEGAPIRKGDYVCCATMIANRDPAEFEDPNVIDLAREDNCHTAFAAGPHRCLGSHLARREII
IGLEEWLSHIPHFRIKEGTAPITHGGHVFGIENLVLEWS

>CYP127A8(Mesci_5828)*Mesorhizobium cicero*

MVANPVPDHVPPEMVGDFSLFTSPCMSPTPNGDPHAAVACVHAGPPIFYSPWNTRAGQGTWVITRAADQRRVL
QETEVFSSHRSIFASALGEDWPMIPLELDPPYHGVFRSLLNPLLSPQRVAEFEPTIRERAITLIDSIASRGTS
CDVMKDFAFPFAVNIFLRFLGLPDHRLDTFVGWAKDLLHGDDLRRPAAARTIVAFIDELAAMRRKEPVDDFMS
FIVHAQVEGRGLTDQEVRGIGVLTFVAGLDTVAAAIGFDLAYLARNLEDQELLRNEPDRIVLAAEELLRAYPS
VQMIRVARKDIDFKGAPIRKGDFVSCATMIANRDPTEFEAPNTVDLTREGNPHAAFGYGPHRCLGSHLARREI
VVGLEEWMARIPPFRIKEATAPITHGGHVFGIENLVLDWARPRPRGTGHIRGK

>CYP127A8(Mesop_6403)*Mesorhizobium opportunistum*

MVANPVPDHVPPEMVGDFSLFTSPCMSPTPNGDPHAAVACVHAGPPIFYSPWNTRAGQGTWVITRAADQRRVL
QETEVFSSHRSIFASALGEDWPMIPLELDPPYHGVFRSLLNPLLSPQRVAEFEPTIRERAITLIDSIASRGTS
CDVMKDFAFPFAVNIFLRFLGLPDHRLDTFVGWAKDLLHGDDLRRPAAARTIVAFIDELAAMRRKEPVDDFMS
FIVHAQVEGRGLTDQEVRGIGVLTFVAGLDTVAAAIGFDLAYLARNLEDQELLRNEPDRIVLAAEELLRAYPS
VQMIRVARKDIDFKGAPIRKGDFVSCATMIANRDPTEFEAPNTVDLTREGNPHAAFGYGPHRCLGSHLARREI
VVGLEEWMARIPPFRIKEATAPITHGGHVFGIENLVLDWARPRPRGTGHIRGK

>CYP127A8(Mesau_05876)*Mesorhizobium australicum*

MVANPVPDHVPPEMVGDFSLFTSPCMSPTPNGDPHAAVACVHAGPPIFYSPWNTRAGQGTWVITRAADQRRVL
QETEVFSSHRSIFASALGEDWPMIPLELDPPYHGVFRSLLNPLLSPQRVAEFEPTIRERAITLIDSIASRGTS
CDVMKDFAFPFAVNIFLRFLGLPDHRLDTFVGWAKDLLHGDDLRRPAAARTIVAFIDELAAMRRKEPVDDFMS
FIVHAQVEGRGLTDQEVRGIGVLTFVAGLDTVAAAIGFDLAYLARNLEDQELLRNEPDRIVLAAEELLRAYPS
VQMIRVARKDIDFKGAPIRKGDFVSCATMIANRDPTEFEAPNTVDLTREGNPHAAFGYGPHRCLGSHLARREI
VVGLEEWMARIPPFRIKEATAPITHGGHVFGIENLVLDWARPRPRGTGHIRGK

>CYP127A9(TZ53_19625)*Sphingobium* sp. YBL2

METFAARPIPAHVPPDMVGDFSLFSSPGMQPTPNGDPQAAAAVVHQGPRIFYSPVNTRDGRGTWVITRAQDQR
KVLQDPETFSSHRSIFASALGESWPMIPLELDPPDHGKFRSLLNPLLSPKRVMALESAVRERAIFLIDRIAAA
GTSCHVMEDFAFPFAVNIFLRFLGLSDDRLDEFVRWANALLHGDNVQRPAAARTIIGFIDELSAERRRRPVDD
FMSFLVQSKIDDRPLTDMEIRGTGVLLFIAGLDTVAAAIGFDLNYLARHMDDQQLLRSDPSRIVLAAEELLRA
YPTVQMIRVATRDIDFEGAPIRRGDYVSCATMIANRDPLEFPHPERIDLAREDNRHTAFAYGPHRCLGSHLAR
REIVVGLEEWLKRIPPFRIKQGTAPVTFGGHVFGIENLVLDWS

>CYP127A10(EP837_00046)*Sphingobium* sp. EP60837

METLATRPIPAHVPPEMVGDFSLFSSPGMQPTPNGDPQAASAVVHQGPRIFYSPVNTRDGRGTWVVTRAQDQR
RVLQDPETFSSHRSIFASALGESWPMIPLELDPPDHGKFRSLLNPLLSPRRVMALETAVRERAVSLIDGIAAS
GTSCDVMQDFAFPFAVNIFLRFLGLSDDRLQEFVRWANDLLHGDNVQRPAAARTIIAFIDDLAAERRKRPVDD
FVSFLVQAKIDDRPLTDAEIRGTGVLLFVAGLDTVAAAIGFDLNYLARHPSDQQWLRDDPSRIVLAAEELLRA
YPTVQMIRVATKDIDFEGAPIRKGDYVSCATMIANRDPNEFPEPEKIDLTREDNRHTAFAYGPHRCLGSHLAR
REIVVGLEEWLKRIPTFRIKEGTAPITYGGHVFGIEKLILDWS

>CYP127A11(RGR602_PB00271)*Rhizobium gallicum*

MPSNRVPDHVPPEMVRDFSLFTSAAMQRTKNGDPHAAVACAHAGPPIFFSPYNTRDGRGTWVITRANDQRKLL
QDTATFSSHRSIFASALGENWPMIPLELDPPAHGVFRSLLNPLLSPKRVMALEPAVRERAIALIDMISASSTS
CDVMKDFAFPFAVSIFLRFLGLSDERLDTFVGWAKDLLHGDGIKRTAAARTIVAFIDDLAAMRRKEPADDFMT
FVVQAKVDGRPLRDEEIRGIGVLLFVAGLDTVATAIGFDLAYLARNPRDQELLRSEPDRIVLAAEELLRAYST
VQMIRVATKDIDFEGAPIRKGDYISCPTMIANRDPAEFECPNTIDLAREDNRHTAFAYGPHRCLGSHLARREI
VIALEEWLSRIPDFRIKKGTAPITYGGHVFGIENLILDWS

>CYP127A12(IE4771_PB00206)*Rhizobium etli* bv. mimosa IE4771

MTFQPNGDLIAASHIVRSSRDLSLEVSRSTIWSVPSSPGRHFRRRGFDGRRKAVASHRVPEHVPPEMVKDFSL
FTSPGMERTPNGDPHAAVACVHDGPPIFYSPCNTRDGRGTWVITRAEDQRRLLQDTETFSSHRSLFASALGEN
WPLIPLELDPPAHGAFRSLLNPLLSPKRVIELEPAIRDRAIALISKISASSTSCDVMKDFAFPFAVSIFLRLL
GISDERLDTFVGWGKHLLHGDGVKRTAAARTILAFIDELAATRRIKPADDFMTFVVQANVDGRPLRDEEIRGM
GVLLFVAGLDTVATAIGFDLAHLARNPKEQELLRSEPDRIVLAAEELLRAYSTVQMIRVATKDISFEGAPIRK
GDYISCATMIANRDPAEFERPNTIDLARKDNRHTAFASGPHRCLGSHLARREIVIGLEEWLSRIPEFRIKEGT
APITFGGHVFGMENLILDWS

>CYP127A13(REMIM1_PE00312)*Rhizobium etli*

MCCSGRILPEGRNPVKLQCGRGDHQLQPQPITSDDFPAEGRSDRCIAHRPLVARSEVGSVALNDLFIPSSPRR
HFRRRGFDSRRNAVASHRVPDHVPPEMVNDFSLFTSPGMERTPNGDPHAAVACVHDGPPIFYSPCNTRDGRGT
WVITRAQDQRRLLQDTETFSSHRSLFASALGENWPLIPLELDPPAHGVFRSLLNPLLSPKRVTELEPAIRNRA
IAVISKISASSTSCDVMKDFAFPFAVSIFLRLLGISDERLDTFVDWGKHLLHGDGIKRTAAARTILAFIDELA
ALRRNEPADDFMTFAVQANVDGRALRDEEIRGMGVLLFVAGLDTVATAIGFDLAYLARNPKEQELLRSEPDRI
VPAVEELLRAYSTVQMIRVATKDVNFEGAPIRKGDYISCATMIANRDPAEFKCPNTIDLAREDNRHTAFAYGP
HRCLGSHLARREIVIGLEEWLSRIPDFRIKEGTAPITFGGHVFGMENLILDWS

>CYP127C1(SGRAN_1061)*Sphingopyxis granuli*

MSGGCPSRPDIAPDMVRRWDLARETEAVGCPFVAASKLHDGPDIFFNLSDDLRNVDGGSWIVTRADLQREVLQ
KSELFSSRGISGFSAILGESWPLMPVELDAPDHMRYRKLINPLFSPRRMDEIEAGVRESAIELIEAVRHRGGC
EFMTAYARELPVNVFLRLMGLPLAEVDTFRKWEDEMMHSDDVAVRAGAAHALKAYLLDIIGRRRAEPTDDLIS
HLVTATLDDRPLDDDAVLGLCFFLYIAGLDTVAATLSFIFATLAEQPDLQNRLRSTPAEIPEAVEELIRAFGT
VVTSRYLTEDTVFHGVAMRKGDRVITPLGVASRDPAEYEDANTLNIDRLSTRNISFGAGPHRCIGSHLARREI
TLTLEEWLARIPEFRLAPNGKPVPNVVTVWGHDTLQLEW

>CYP133F1(B488_03070)*Liberibacter crescens*

MDLSQFSSPEFIINPYPFYEEVRSLGAVVPISPHIVISARYESVREMLHDSRIGKDHIQSIRIRYGEEASQGP
VFQSFSRMMLMLDPPVHYKVRSLLTKAFTAQRVESLRETIQNISNRLINNLVNQLKKHKEIDLAAEYAFKLPV
EIICKLLNIPVQDGEKYGEAARKSVQALNFTPLTAQQISEANAAILSLENYFSKVIAERHKKTGDDLISALLT
TKENDIFLSDDEIISNVILMFIAGHETTSNMISNALISLYKQPEQLNLLRNDSSLMPKAVTECLRLDSSVQIV
GRVALNDLEISGFHIKCGTIIFLMIGAANRDP

>CYP147D2(Mnod_0160)*Methylobacterium nodulans*

MPDPNLFAAVKDPANRPNPYPLYARLRETPVALQHDGSYVVTTHAALRALVFDPRLSSEDLPPISHPRTGNPF
KDWIVNPIKDYVRTKHRPFIFRDPPDHDVLRRHVTRQFTPERLRTMHGRVDALVADLLDRCRDARTIDLVDHF
SYPLPVMVICELLGVPHEDEPRFHGWATQLATALEPDAREDEETKGRNIETFDVIAAYMQDLIREKRRHPQDD

LLTGLATYEDPKVGRMGDYDLIATAILLLIAGHETTVNLITNGMLTLLRFPEHLERLRADPEIAPRLIEELLR
YEPPVHFRTRKALADIRMEGVTIPQGAPIVLLFASGSRDPARFPDPDRFDPDREDNQHFGFGGSLHFCVGAPL
ARMEAEAALVALAQRLRNPRLIEDPPPYRPGASLRGPQHLRLAIEGVA

>CYP147D3(Mex_1p4388)*Methylobacterium extorquens* AM1

MCRSPSPVSGSTLFDQILDPANRANPYPLYAELRKVPVARQEDGTYVVSGHAELARLISDPRISSDDLPDPQK
FRWTGHPVTDLLVRPVRAEIRKRHRPFIFRDPPDHDRLRGQVMRCFTPERVRGMRAKTQEITDDLIGKMRGKT
QIDLVDDFSYPLPVTVICELLGVPPEDEAQFHGWATQLATALEPNQRGDAETQAKNEVCFNEIADYLQGLIKE
KRKDPQQDILSDLATDKEGMNDFDLIATAVLLLVAGHETTVNLITNGMLTLLRFPEHGERLRAEPDLAPRLIE
ELLRYEPPVHYRTRVALTDIPVAGITIPKDAPVIFLLAAANRDPIRFPDPDRFDPDRPDNRHLGFGGGLHYCV
GAPLARIEAEVALVSLVRRLKGVSLIEDPPPYRPGASLRGPRHLRLALEGVAEA

>CYP147D3(METDI_4994)*Methylobacterium extorquens* DM4

MSGSTLFDQILDPANRANPYPLYAELRKIPVARQEDGTYVVSGHAELARLISDPRISSDDLPDPQKFRWTGHP
VTDLLVRPVRAEIRKRHRPFIFRDPPDHDRLRGQVMRCFTPERVRGMRAKTQEITDDLIGKMRGKTQIDLVDD
FSYPLPVTVICELLGVPPEDEAQFHGWATQLATALEPNQRGDAETQAKNEVCFNEIADYLQGLIKEKRKDPQE
DILSDLATDKEGMNDFDLIATAVLLLVAGHETTVNLITNGMLTLLRFPEHGERLRAEPDLAPRLIEELLRYEP
PVHYRTRVALTDIPVAGITIPKDAPVIFLLAAANRDPIRFPDPDRFDPDRPDNRHLGFGGGLHYCVGAPLARI
EAEVALISLVRRLKGVSLIEDPPPYRPGASLRGPRHLRLALEGVAEE

>CYP147D3(Mext_4000)*Methylobacterium extorquens* PA1

MSGSTLFDQILDPANRANPYPLYAELRKFPVARQEDGTYVVSGHAELARLISDPRISSDDLPDPQKFRWTGQP
VTDLLVRPVRAEIRKRHRPFIFRDPPDHDRLRGQVMRCFTPERVRGMRAKTQEITDDLIGKMRGKTQIDLVDD
FSYPLPVTVICELLGVPPEDEAQFHGWATQLATALEPNQRGDAETKAKNEVCFNEIADYLQGLIKEKRKDPQQ
DILSDLATDKEGMNDFDLIATAVLLLVAGHETTVNLITNGMLTLLRFPEHGERLRAEPDLAPRLIEELLRYEP
PVHYRTRVALTDIPVAGITIPKDAPVIFLLAAANRDPARFPDPDRFDPDRPDNRHLGFGGGLHYCVGAPLARI
EAEVALISLVRRLKGVSLIEDPPPYRPGASLRGPRHLRLALEGVAEE

>CYP147D3(Mchl_4369)*Methylobacterium extorquens* CM4

MSGSTLFDQILDPANRANPYPLYAELRKIPVARQEDGTYVVSTHAELARLISDPRISSDDLPDPQKFRWTGHP
VTDLLVRPVRAEIRKRHRPFIFRDPPDHDRLRGQVMRCFTPERVRGMRAKTQEITDDLIGKMRGKTRIDLVDD
FSYPLPVTVICELLGVPPEDEAQFHGWATQLATALEPNQRGDEETQAKNEVCFTEIADYLQGLIKEKRKDPQQ
DILSDLATDKEGMNDFDLIATAVLLLVAGHETTVNLITNGMLTLLRFPEHGERLRAEPDLAPRLIEELLRYEP
PVHYRTRVALTDIPVAGITIPKDAPVIFLLAAANRDPIRFPDPDRFDPDRPDNRHLGFGGGLHYCVGAPLARI
EAEVALVSLVRRLKGVSLIEDPPPYRPGASLRGPRHLRLALEGVAEA

>CYP147D3(Mpop_4481)*Methylobacterium populi*

MSGSTLFEQILDPAHRANPYPLYAEMRRVPVARQADGTYVVSTHTELARLISDPRISSDDLPDPQKFRWTGHP
VTDLLVRPVRAEIRKRHRPFIFRDPPDHDRLRGQVMRCFTPERVRGMRGKTQTITDDLIGKMRGKTRIDLVDD
FSYPLPVTVICELLGVPPEDEAQFHGWATQLATALEPNQRGDEETQAKNEVCFNEIADYLQGLIKEKRKDPQQ
DILSDLANDGDGMNDFDLIATAVLLLVAGHETTVNLITNGMLTLLRFPEHGARLRAEPDLAPRLIEELLRYEP
PVHYRTRLALADIPVAGITIPKDAPVIFLLAAANRDPTRFPDPDRFDPDRPDNRHLGFGGGLHYCVGAPLARI
EAEVALVSLVRRLKGVSLIEDPPPYRPGASLRGPRHLRLALEGVAE

>CYP147D3(Y590_19915)*Methylobacterium* sp. AMS5

MSGSTLFEQILDPANRANPYPLYAELRKAPVARQADGTYVVSTHAELARLISDPRISSDDLPDPQKFRWTGHP
VTDLLVRPVRAEIRKRHRPFIFRDPPDHDRLRGQVMRCFTPERVRGMRAKTQAITDDLIGKMQGKSRIDLVDD
FSYPLPVTVICELLGVPPEDEAQFHGWATQLATALEPNQRGDEENQAKNEVCFNEIADYLQGLIKEKRKDRQE
DILSDLANEKEGMNDFDLIATAVLLLVAGHETTVNLITNGMLTLLRFPEHLERLRAEPETAPRLIEELLRYEP
PVHYRTRLALTDIPVAGITIPKDAPVIFLLAAANRDPIRFPDPDRFDPDRPDNRHLGFGGGLHYCVGAPLARI
EAEVALVSLVRRLKGVSLTEDPPPYRPGASLRGPRHLRLALEGVAGE

>CYP147D4(M446_4599)*Methylobacterium* sp. 4-46

MPDPTLFAAVKDPANRPNPYPLYARLREAPVALQDDGTYVVTTHAALRALLFDPRISSEDLPSTSHPRTGNPF
KDWIINPFKDHVRTKHRPFIFRDPPDHDVLRRHVMREFTPERIRAMHGRVDGLVADLLDRRRDARAIDLVDDF
SYPLPVTVICELLGVPPEDEGRFHGWATQLATALEPDARQDEAALGRNIETFDAIAAYMQDLIKDKRRHPKDD
LLTGLATREDPQAGRMGDYDLIATSILLLIAGHETTVNLITNGMLTLLRFPAQLERLRAEPEIAPRLIEELLR
YEPPVHFRTRKALAEIELAGTTIPAGAPVVLLFASGSRDPARFADPDRFDPDREDNQHFGFGGSLHFCVGAPL
ARLEAEAALVALARRLRNPRLLDDPPPYRPGASLRGPEHLRLAIDGVSPPDA

>CYP147D5(Maq22A_c00735)*Methylobacterium aquaticum*

MTDTDGTLFAQVRDPENRADPYPLYTRLRAQPVARQDDGTWVASGYAAIRALLHDPRVSSEDLPPAEHPATGN
PIKDFFVNPIKDWITDRHRPFIFRDPPDHGLLRRQVMGQFTVARVRGMKRRTEALVDECLRARAGATRFDLVD
DLAYPLPVTVICEMLGVPREDEARFHAWATQLATALEPESRNDEDLRARNTETFDAIAAYMRDLIREKRRAPA
DDMLSGLATTKDAQAGRMSDPDLVATAILLLIAGHETTVNLITNGMLTLLRHPDELARLRADPERAPRVIEEL
LRYEPPVHFRTRLTRAPIPVGDVTIPEGVPLVLLFAAGNRDPARFPDPDRFDPDREDNQHFGFGGALHYCVGA
PLARIEAEAALTALVARLREPRLVEDPPPYRAGASLRGPERLLLDIRGIA

>CYP147D6(MOC_4430)*Methylobacterium oryzae*

MSDDALFAAVMDPANRADPYPLYARLRQRPVSRQRDGSYVVSTHAAIRGLLFDPRLSSEDLPPPRRPRTGNPL
KDLILNPIKNRISATHRPFIFRDPPDHDRLRSLVMREFSIARVQALRPRIAREVDALIDTCRGRREVCLVSDL
SYPLPVTVICELLGVPREDEPRFQAWATQLATAVEPDARHDDTTRRKIVGAFDEIAAYMRDLIREKRRRPTDD
MLSGLAAPGPDGRKQLSDFDLLSTAVLLLVAGHETTVNLITNAMLTLLRHPEQLERLRADPERAPRLIEEVLR
YEPPVHFRTRKALGAITVAGETIPKGAPVILLFAAANRDPARFDAPDRFDPDRSDIEHFGFGGGLHYCVGAPL
ARIEAEIALVALSRRLIAPRLPQDPPPYRPGASLRGPEQLPIGIDGIA

>CYP147D6(Mrad2831_3880)*Methylobacterium radiotolerans*

MSDDALFAAVMDPANRADPYPLYRRMRQQPVSRQRDGSYVVSTHAAIRSLLFDPRLSSEDLPPPRRPRTGNPL
KDLILNPIKNRISATHRPFIFRDPPDHDRLRSLVMREFSIARVQALRPRIAREVDGLIDACRERREVCLVSDL
SYPLPVTVICELLGVPRADEPRFQAWATQLATAVEPDARHDDGTRRQIVGAFDEIAAYMRDLIREKRRRPADD
MLSGLAAPGPDGRKQMSDFDLLSTAVLLLVAGHETTVNLITNAMLTLLRHPEELERLKADPERAPRLIEEVLR
YEPPVHFRTRKALGAIAVAGEMIPKGAPVILMFAAANRDPDRFAAPDRFDPDRGDIEHFGFGGGLHYCVGAPL
ARLEAEIALVALSRRLVAPRLAVDPPPYRPGASLRGPEQLRIGIDGVA

>CYP147D7(Mpop_5420)*Methylobacterium populi*

MGDDTLFAQVLDPRNRPDPYPLYGRLRETPVSRQADGSFVVSTHAAVRSLIFDPRLSSEDLPPSRRPPTGNPL
RDWILNPIRNRVVNAHRPLIFRDPPDHDTLRDFVMKEFTIARVRESHAKVARLVDTLIDACRGRDTVCLVDDL
SYPLPVAVICELLGVPEADEAQFHAWAIRLATALEPDARHDEAGRREITATFDAISDYMRRLIREKRRRPADD
MLSGLAARGGQGRPAMGDIDLISTAILLLVAGHETTVNLITNGMLTLLRHPDELERLKVDPARAPRVIEEMLR
YEPPVHFRTRKALGAIDVAGVTIPAGSPVILLFASASRDPARFADADRFDPDRRDNQHFGFGGGLHYCAGAPL
ARIEAEIALVTLCRRLVAPRLLDDPPPYRPGASLRGPKNLGTSASGLYLFFYYLGGLAGTAVLGQVFERWGWG
ACVLGVAASLALAAALTLGLREPSSARGVT

>CYP147D8(MOC_2231)*Methylobacterium oryzae*

MPDATLLNQVKDFANRPNPYPIYARLRENPVSRQDDGTEEGTWVAATHGTIASLLQDPRVSSETLPPADRPRT
GNPLTDLIVKPLKDWMMDRHRVFIFRDPPDHDALRSAVMHQFSRERVQAMRARSDQLVADLLDEKCGAREIDA
VDDLAYPLPVTVICELFGVPREDEPKFHGWATQLATALEPDSLSDPEIRAENSRTFDAIGGYMADLIKDKRKH
PGDDMLSGLANHATPAGVKMGDYDLIATSILMLVAGHETTVNLITNGLLTLLRHPDELERLRQDPLRAPRLIE
ELLRYEPPVQFRTRRTLSAIDIAGVTIPEGADLVLLLASGNRDGAVFPDPDRFDPDRAGLRHLGFGGSLHYCV
GAPLARFEAEAALTALARRLKAPRLIEDPPPYRSGAALRGPEHLRVAIDGIA

>CYP147D8(Mrad2831_1827)*Methylobacterium radiotolerans*

MPDATLLDQVKDFANRPNPYPVYAKLRENPVSRQDDGTEAGTWVAASHGTIARLLQDPRVSSDTLPPADRPRT
GNPLTDLIVKPLKDWMMDRHRVFIFRDPPDHDALRSAVMHQFSRERVQAMRARSDRLVADLLDEKCGAREIDA
VDDLAYPLPVTVICELFGVPREDEPKFHGWATQLATALEPDSLSDPEIRAANSRTFDAIGGYMGDLIKEKRRH
PQDDMLSGLANHATPAGVKMGDYDLIATSILMLVAGHETTVNLITNGLLTLLRHPDELERLRQDPLRAPRLIE
ELLRYEPPVQFRTRRTLSPIDIAGVTVPEGADLVLLLASGNRDAAVFPDPDRFDPDRAGTRHLGFGGGLHYCV
GAPLARFEAEAALTALARRLKAPRLIEDPPPYRPGAALRGPEHLRVAIDGIA

>CYP147D9(MOC_0533)*Methylobacterium oryzae*

MSDETLFAQVLAPKNRADPYPLYARMRETPVSRQADGSYLVSTQAALRSLIFDPRLSSEDLPPSRRPATGNPP
KDWILNPIRHRVVNAHRPLIFRDPPDHDTLRGIVMKEFTIARVRDSHAKVATLVDALIDKCRGRDTVCLVDDL
SCPLPVAVICELLGVPEADERQFHAWATRMATALEPDARHDEAGRREIAATFDAISDYMRRLIREKRRHPADD
MLSGLAARGGRGRLAMGDIDLISTAILLLVAGDETTVNLISNGMLTLLRHPDELEKLKADPKRAPCVIEEILR
HEPPVHFRTRKALGAIEVAGATIPAGSSVVLMLAAGNRDPARFTQPDRFDPDRNDNQHFGFGGGLHYCVGAPL
ARVEAEIALVTLCQRLVAPRLLDDPPPYRAGASLRGPKNLGIAIAGIA

>CYP147D10(Maq22A_1p34675)*Methylobacterium aquaticum*

MNGTLFQQILDPANRADPYPLYAEMRRTPVSRQDDGSYVVSTHAELYRLLHDPRISSEDLPDPPLFRWTGHPL
SDLFVRPVRSRITARHRPFIFRDPPDHDLLRRQVMREFAPARVRAMRRKTEQLTHTLLDGLRDRTNLDLVDDF
SYPLPVTVICELLGVPREDEPRFQGWATQLATALEPDRRTDRENAGRTEEAFTAISDYLRGLIKGKRRAPQDD
LLSGLAAEHDGERMNDHDLVATAVLLLVAGHETTVNLITNGILTLLRFPEHLERLRREPEVAPRLIEELLRYE
PPVHYRTRLARADIAIGGTTIPEGAPVVLLLASGSRDPMRFADPDRFVPDRANNQHFGFGGSLHYCVGAPLAR
IEAEVALVALAQRLQAPRLLADPPPYRPGASLRGPRHLLLAIDAVAPGASAELAA

>CYP151C1(Ga0080559_TMP4891) *Salipiger profundus*

MPAMSLEAQFPDAPFLDVANPAFSLRSEPVREARAQSWYARTPYGLAVLRHKEMGELLTHKSLIQGSHAWPAL
NGVTSGTFADWWNSSILVTEGDDHWRLRRLVNPAFSPKVVKALMPEFERIANDLADGFIESGSCDFMAEFADP
YAARVLCLLLGLPETEAPFILRTSATMGLALGVNFPDLVDEIEAATVELGDYISEVLKARETDPGDDILSTLV
QVRAEGDRLSHDELWNIALMLAFAGVDTTRNQLGLGMSMFAAHPEQWETLAADPSLDMAAATEVMRMRPTITW
VSREATEDFEYGGVTIPQGTVLHLYSESAGTDPQVMGEAPFDITAKRARNYGFGGGIHHCLGNLVARNDMAVA
YRVLSARMGPPVVGEGATWLADSGNTGPISLPITFAPR

>CYP151C2(Ga0080574_TMP4712)*Pelagibaca abyssi*

MSAETLFADAPYLDVADPAFSLRSEPVREARRDSWFARTPYGLAVLRHKEMGELLTHKSLIQGSHAWPALNGI
TEGSFAEWWNKSILVTEGDDHWRLRKLVNPAFSPKVVKALMPEFERIATDLADGFIAKGACDFMEDFADPYAA
RVLCLLLGLPESEAPFILRTSATMGLALGVNFKAEAENIEAATEELGAYIAQVLADRQATPGEDILSIMVQAS
EDGDRLSYDELHNLALMLAFAGVDTTRNQLGLGMSMFAAHPEQWEALAADPSLDMAAATEVMRMRPTITWVSR
EAIEAFDYGGITIPKGTVLHLYSESAGTDPAVMGEAGFDITQKRARNYGFGGGIHHCLGNLVARNDMAVAYRV
LSAGMGAPSIAEGATWLADSGNTGPISLPITFLPR

>CYP152B3(Meso_4213)*Chelativorans* sp. BNC1

MPRIPRDRSLDSTIALMGDPYRFISNRSRRYRSDLFETRLLLRKTICMTGPEAAQLFYDPSRFVRTGAMPKAI
QKTLLGVGGVQGLDDDAHRHRKQMFMSLMTPERIEQLVQLTAAEWQIRVLKWGSMDEVVLYPELHLLLTRAVC
AWAGVPLADSEVDARTKEIAALFDHAGAVGLRHLWSRWARKQADRWIADIIDQIRSGRIRPPEQSAAHIIAWH
RDLNGELLTPQIAAVELINVIRPTVAVSVYMLFVAHALHAHPKIRERLQADEDSYSRRFVQEVRRYYPFFPAV
AARTRQAFDWNGYQFTAGRRVLLDLYGTNQDPRTWKRPEEFEPERFRHWDESPFNFIPQGGGDHYVNHRCPGE
WIAIELMKLTADFLSRSMSYEVPQQDLRIDWSRLPALPRSRFVIRNVRES

>CYP152B4(Sala_2556)*Sphingopyxis alaskensis*

MSRIPRDKSLDSTIALMGDPYRFISNRCRRYRSDLFETRLLLRKTICMTGPEAARLFYDQSRFARHGAMPKAI
QKTLLGVGGVQGLDEGAHRHRKQMFMSLMTPEGIEKLVRLTSAEWQIRVRKWASMNAVVLYPELHALLTRAAC
AWAGVPLADSEVEPRTRQVTALFDHAGSIGLRHLWSRRARKRADSWAADIVEQIRSGRLRAPEHSAAHVVAWH
RDFSGALLTPQVAAVELINVIRPTVAVSVYMIFVAHALHTHPQVREKLQAGDEDYAECFVQEVRRYYPFFPAV

AAQTRLAFEWNGYQFPAGRRILLDLYGTNQDPRTWERPEDFEPERFGRRDGSPFNFIPQGGGDHYFDHRCPGE
WIAIELMKLTADFLTRRMSYEVPGQDLRIEWSRLPALPRSRFVLSNVRECQELHLQRTRWRRR

>CYP152B6(NT26_p10171)*Rhizobium* sp. NT-26

MSRIPRDRSFDSTLALLLDPYGFISKRCRRYGADLFQTRLLLRPTICMTGPEAAELFYRQDLFARSGAAPGRV
QKTLFGQGGVQGLDGEAHRHRKQMFMSLMTPERIEALKELTAEWWRTYAQKWTTMDRVILYDEVQELLTRAVC
AWVGVPLPEAEAGRRAAELTALFDYAGSVGPKHWWSRLSRRRCESWIEGIVEQIRAGDLSPPEGSAARVIATW
RDQDGALLSPRIAAVELLNILRPTVAVAVFIVFAAHALHGFPACRPKLQTGDEYPELFVQEIRRLYPFFPAVM
ARTRHNFEWNGYRFPRGRRVMLDLYGTNRDARAWDAPEEFRPDRFRSWDGSPFNFIPQGGGDHHMNHRCPGEW
MTIELMKLSCRFLAASIEYDLPDQDLRIDITRLPAIPKSRFVMSNLRYAIGGAS

>CYP152B7(MALG_04698)*Marinovum algicola*

MSSIPSATGFDSTLALLRNPYGFIRQTCRHLDADLFETRILLHKTICMTGAAAAEAFYREEQLIREGSMPSRI
QKTLLGEGGVQGLDGATHQHRKKMFMSMMGTEHIAALQGMSLHMLDNFAPDWEAKNEVVFYDEVREMLTRAVC
AWCGVPIPEAEVATRTAQLTSLFQDAGAIGPKHWAARLARHRLEKWAARMIQQVRDGELQPTQESALHFIATW
RDLDGELLNPRVAAVELLNVLRPTVAVSVFIVQTAHALYRYPEWQEKLRNDEGLLEPFVQEVRRLYPFFPAVA
ARVKSTFEWQGYRFPKGHRVLLDLYGTNTDRRSWDAPVEFRPERFRGRAENPYDFIPQGGGNHYTNHRCPGEW
IAISQMKAFCRYLVNDIDYDVPDQDLELDPGELPSLPQNRFIMRNVRRRQ

>CYP152C1(RSP_2378)*Rhodobacter sphaeroides* 2.4.1

MTTDEGRRPEEPGTPASLREMPRDPRIDASMALMSEGYRFVSNLCDRMDSDAVATRLRLREVVCLRGSAAARL
LYGAEGLTRVGAMPSTVLHLLQDKGSVQQLEGPAHRHRKALFLSICMDPARVEALVSEMRLAWRERLPAWEAE
GRIVLQQEAARLLTRAGCRWAGVAHQPEAQLADEIFDMIDKAGSVGPRNWLAQMRRAGTEKRLRTLVEEVRAG
EVVPEAATALHAIAFHREEDGTLLDPSVAAVELLNLLRPIVAVGRYITFAALALHRETTWRELFRSGNLELAG
DFAEEVRRASPFFPFTAAVTTRPITWEGYDFPEGQWLLLDLYGTTHDPRHFPEPTRFRAERMLSWTGQDEAFI
PQGAGDVARTHRCPGEMITVELMKEAIRLLCCEMDYEVPAQDLGVRLNRMPAQPRSGMILSAISRRAGTEASR
NG

>CYP152C1(Rsph17029_1038)*Rhodobacter sphaeroides* ATCC 17029

MTTDEGRRPEEPGTPASLREMPRDPRIDASMALMSEGYRFVSNLCDRMDSDAVATRLRLREVVCLRGSAAARL
LYGAEGLTRVGAMPSTVLHLLQDKGSVQQLEGPAHRHRKALFLSICMDPARVEALVSEMRLAWRERLPAWEAE
GKIVLQQEAARLLTRAACRWAGVAHQPEAQLADEIFDMIDKAGSVGPRNWLAQMRRAGTEKRLRTLVEEVRAG
EVVPEAATALHAIAFHREEDGALLDPSVAAVELLNLLRPIVAVGRYITFAALALHRETTWRELFRSGNLELAG
DFAEEVRRAAPFFPFTAAVTTRPITWEGYDFPEGQWLLLDLYGTTHDPRHFPEPTRFRAERMLSWTGQDEAFI
PQGAGDVARTHRCPGEMITVELMKEAIRLLCCEMDYEVPAQDLGVRLNRMPAQPRSGMILSAISRRAGTEASR
DG

>CYP152C2(Rsph17025_1898)*Rhodobacter sphaeroides* ATCC 17025

MSNSDARHEAGPSDTTCGREIPRDPRLDASMGLLSEGYRFVSNLCDQLDSDIVSTRIRLREVICLRGGTAARL
LYGAEGLTRVGAMPSSVLHLLQDKGSVQQLEGPAHRHRKALFLGICMDPARVEALVAEFRAAWRAALDEWEEA
DSIVLQQEAARVLTRAACRWAGVENQPEARLAEEIFDMIDKAGSIGPRNWLAQMRRSGTERRLRKLIEQVRAG
EVVPGSATALHAIAFHREADGALLDDTVAAVELLNVLRPIVAVGRYITFTALALHRETNWRDLFRSGTLELAA
DFAEEVRRISPFFPFTAAVTTRPLQWEGYDLPADQWLLLDLYGTLHDARNFPDPARFRAERMLSWSGQDDCFV
PQGGGEVAVTHRCPGEMITVELLKEAIRLLCLEMDYEIPPQDLGVRLNRLPAQPRTGMVLASIRRRPGTEPCV
AG

>CYP152E3(AZOBR_p440090)*Azospirillum brasilense* Sp245

MHPIPRDASPDSTLALLSEGYRFVMNRCERHGSDIFETRLMLRKAICVMGEEAAGMFYEPDRFTRKGAMPITT
LMLLQDRGAAQTLDGEAHRWRKRMLMALMEPENIRRLCGEVAAEWRAALPGWERAERVVLLDAVRAILCRAVC
RWAGVPLAEEEAGPRTAEFAAMIDGAGAVGLRNWRGLLLRTRTERWARGLIEDVRAGRLVMTEGDAESRAIHA
IATHRGLDGNRLDTVTASVELINILRPVVAIAQYVVFAAMALHEHPEQRAALQDASPEEVEHFVQEVRRFYPF

FPLVAGRVRTPFDWRGHRFAEGTWVLLDLYGTCHDPRIWNDPERFRPERFRGREPGAFALIPQGGGGHLRGHR
CAGEWITIEVVKTALRLLTREMRYSVPAQDLALDLSRMPAVPRSRFVITDVAPRRKADAVLDPPVALSRRAH

>CYP152P2(M446_4307) *Methylobacterium* sp. 4-46

MFAIPRDPAPDSTLALLREGYGFIPGRCRNFGSDLFTTRLMLTNVVCMTGADAAAQFYAPDRFTRRGALPRIS
FTLIQDNGSVMEVMDGEAHRRRKAMFLSLMSPEALQRLADLTTQAWRARMRRWAAQETIPLFDEAHVPFCAAV
CAWAGLPLNEAEAERSREFLAMIEGTGSIGPRNWRGHLLRARAERWMRDAIRQIREGSRAVPEGSAAHVIAHH
RDAEGRLLDVRTSAVELINILRPTVANARYVVFAAHALHCYPESREALQAAEGVEPFVQEVRRFYPFIPFIGG
RALQTVEFHGHRFAVGDWVLMDLYGTNRDPNRWGQPERFIPDRFAQWDHDPNGFIPQGGGAYETGHRCPGEWI
TIEQMRAVVPLLAREMRYVVPEQDLTIDLGRIPAMPKSRFVITKVAG

>CYP152AA1(W911_03615)*Hyphomicrobium nitrativorans*

MKEPPRDPFPDSTVALAWEGYRFASNRLRRFGTDAFSCRLMMKKAVCIGGPAAAALFYDSGSVERKDVAPGRI
EKTLLGQGGIHGLDGLAHRHRKEMYLELMGPQSLSSFTPILVRTWREAAASWQPGTRLNLFSEAQTVLFRTMC
EWAGIPLGPRETQQRAADAAAMVDAFGGIAWRNWKGRFARRRSERWMHGIVSATRDRTSAPSPTSALARIANF
RDVDGNLLPAEVTAVELLNVIRPALAIPYFLDLAALKLSEISEMRDQVASNDEMLDCFVQELRRFCPFTPFLG
ARSRREIAWQGFRIPSDTLVVLDVYGIHRDERVWQNAAAFAPLRFQGVGDYRFSLLQQGGGDHLSGHRCAGEW
FTIEALKFFSRELARMPYAVIPASSSFSLTRIPSRLIPPVALRLG

>CYP152AB1(BN1229_v1_0635)*Filomicrobium* sp. W

MRSGSLPRLKGFDHTIAFLHDGYEFVSRRCDRLNSDAFLARLMLQKVVCARGEDAAQLFYEGGRFTRRGAMPP
TTMRLLQDLGSVQQLDGAAHRHRKAMFVRLLMGADAEQQITELFRGEWRNAAKEWSSRPSIVLFDQANVILTK
TICRWMQIPASKPYAELANEFSSMIENSGSAGFAVILALIRRRSTERYFERLVRSIREGTAIVATDSPLAYVV
NYRDMDGSLLSERSAAVELINILRATVAIGRFVIFVAMALEQQPKWKEKLHSADDAAYEHFAEEVRRLYPFFP
VIGGRAIESFEWRGHEFDCGDWFILDLYGTNHFRRLFPEPHQFNPARRLAWRTQGYDFIPQGGGDSRTDHRCP
GEQITVGIMREACRLLVEDMQYELPEQDLTLSLHEIPARPRSGVVLSNIRLNI

>CYP152AB1(BN1229_v1_0639)*Filomicrobium* sp. Y

MRSGSLPRLKGFDHTIAFLHDGYEFVSRRCDRLNSDAFLARLMLQKVVCARGEDAAQLFYEGGRFTRRGAMPP
TTMRLLQDLGSVQQLDGAAHRHRKAMFVRLLMGADAEQQITELFRGEWRNAAKEWSSRPSIVLFDQANVILTK
TICRWMQIPASKPYAELANEFSSMIENSGSAGFAVILALIRRRSTERYFERLVRSIREGTAIVATDSPLAYVV
NYRDMDGSLLSERSAAVELINILRATVAIGRFVIFVAMALEQQPKWKEKLHSADDAAYEHFAEEVRRLYPFFP
VIGGRAIESFEWRGHEFDCGDWFILDLYGTNHFRRLFPEPHQFNPARRLAWRTQGYDFIPQGGGDSRTDHRCP
GEQITVGIMREACRLLVEDMQYELPEQDLTLSLHEIPARPRSGVVLSNIRLNI

>CYP153A2(CC_0063) *Caulobacter vibrioides* CB15

MMSQNTDPREDLMSDGSIDLKADARARAYSIPLEDYHVADPALFQADAMWPYFERLRKEAPVHYSKGDEEVGP
YWSVTRYNDIMTVDTTHQVFSSDAHLGGITIRNFDEDFVLPMFIAMDQPKHDIQRKTVSPIVSPANLGRLEGI
IRERVCGILDALPINEPFDWVDKVSIELTTQMLATLFDFPWEERRKLTRWSDIATASPESGLIESEEARRAEL
LECLAYFTNLWNERVNLTEPGNDLISMLAHGEATRDMPPMEYLGNVILLIVGGNDTTRNSLTGGLYALSKNPQ
EEAKLRADPGLIPNMVSEIIRWQTPLAHMRRTALEDYELAGQTIKKGDKVVMWYVSGNRDDTVIENADQFIVD
RPNARRHLSFGFGIHRCVGNRLAEMQLKIVWEEILKRFPKIEVLEEPKRVYSTFVKGYERMMVRIPERI

>CYP153A2(CCNA_00061) *Caulobacter vibrioides* NA1000

MSDGSIDLKADARARAYSIPLEDYHVADPALFQADAMWPYFERLRKEAPVHYSKGDEEVGPYWSVTRYNDIMT
VDTTHQVFSSDAHLGGITIRNFDEDFVLPMFIAMDQPKHDIQRKTVSPIVSPANLGRLEGIIRERVCGILDAL
PINEPFDWVDKVSIELTTQMLATLFDFPWEERRKLTRWSDIATASPESGLIESEEARRAELLECLAYFTNLWN
ERVNLTEPGNDLISMLAHGEATRDMPPMEYLGNVILLIVGGNDTTRNSLTGGLYALSKNPQEEAKLRADPGLI
PNMVSEIIRWQTPLAHMRRTALEDYELAGQTIKKGDKVVMWYVSGNRDDTVIENADQFIVDRPNARRHLSFGF
GIHRCVGNRLAEMQLKIVWEEILKRFPKIEVLEEPKRVYSTFVKGYERMMVRIPERI

>CYP153A3(blr_7242)*Bradyrhizobium diazoefficiens* USDA 110

MHYCKDSMFGPYWSVTRYNDIMEIETNHSVFSSASALGGITIRDIDPDLRRESFISMDPPRHAAQRKTVAPMF
TPTHLDNLALNIRARSAECLDNLPRGEVFDWVDRVSIELTTQMLAVLFDFPWEDRRKLTRWSDIATTIPGPDG
LVATEDERQAELTECAGYFARLWKERIEQPPKSDLLSMMAHGAATRDMDAKNFLGNLVLLIVGGNDTTRNTMS
GSIYALSQHPEQYRKLRENPALLDSFVPEVIRWQTPLAHMRRTALSDFEFRGKQIKKGDKVVMWYVSGNRDEE
AIEKPYDFIIDRARPRTHLSFGFGIHRCVGLRLAELQLKIIWEEILKRFDHIDVVGEPKRVYSSFVKGLETLP
VKIAA

>CYP153A3(BJ6T_21500)*Bradyrhizobium japonicum* USDA 6

MHGTIESAAKLDELRARATSLPLEQFDPGDPELFRTDTFWPYFDRLRRDDPVHYCKDSMFGPYWSVTRYNDIM
EIETNHSVFSSASALGGITIRDIDPDLRRESFISMDPPRHAAQRKTVAPMFTPTHLDNLALSIRERSAECLDN
LPRGEVFDWVDRVSIELTTQMLAVLFDFPWEDRRKLTRWSDIATTIPGPDGLVATEDERQAELTECAGYFARL
WKERIEQPPKSDLLSMMAHGAATRDMDAKNFLGNLILLIVGGNDTTRNTMSGSIYALSQHPEQYRKLRENPGL
LDSFVPEVIRWQTPLAHMRRTALADFEFRGKQIKQGDKVVMWYVSGNRDEEAIEKPYDFIIDRARPRTHLSFG
FGIHRCVGLRLAELQLKIIWEEILKRFDHIDVVGEPKRVYSSFVKGLETLPVKISA

>CYP153A3(RN69_10530)*Bradyrhizobium japonicum* E109

MHGTIESAAKLDELRARATSLPLEQFDPGDPELFRTDTFWPYFDRLRRDDPVHYCKDSMFGPYWSVTRYNDIM
EIETNHSVFSSASALGGITIRDIDPDLRRESFISMDPPRHAAQRKTVAPMFTPTHLDNLALSIRERSAECLDN
LPRGEVFDWVDRVSIELTTQMLAVLFDFPWEDRRKLTRWSDIATTIPGPDGLVATEDERQAELTECAGYFARL
WKERIEQPPKSDLLSMMAHGAATRDMDAKNFLGNLILLIVGGNDTTRNTMSGSIYALSQHPEQYRKLRENPGL
LDSFVPEVIRWQTPLAHMRRTALADFEFRGKQIKQGDKVVMWYVSGNRDEEAIEKPYDFIIDRARPRTHLSFG
FGIHRCVGLRLAELQLKIIWEEILKRFDHIDVVGEPKRVYSSFVKGLETLPVKISA

>CYP153A3(S23_58660)*Bradyrhizobium* sp. S23321

MHGTIESAAKLDALRERASSLPLEQFDPGDPELFRTDTFWPYFDRLRREDPVHYCKDSMFGPYWSVTRYNDIM
EIETNHSVFSSASSLGGITIRDIDPDLRRESFISMDPPRHAAQRKTVAPMFTPTHLDNLALNIRKRSAECLDN
LPRGEVFDWVDRVSIELTTQMLAVLFDFPWEDRRKLTRWSDIATTIPGPDGLVATEDERQAELAECAGYFARL
WKERIAQPPKSDLLSMMAHGAATRDMDAKNFLGNLILLIVGGNDTTRNTMSGSLHALSQHPEQYRKLRENPEL
LDSFVPEVIRWQTPLAHMRRTALADFEFRGKQIRKGDKVVMWYVSGNRDEEAIERPYDFIIDRARPRTHLSFG
FGIHRCVGLRLAELQLKIIWEEILKRFDHIDVVGEPKRVYSSFVKGFEALPVKIAA

>CYP153A3(BF49_2772)*Bradyrhizobium* sp. BF49

MHGTIENAAKLDALRARATSLPLEQFDPGDPELFRTDTFWPYFDRLRREDPVHYCKDSMFGPYWSVTRYNDIM
EIETNHSVFSSASSLGGITIRDIDPDLRRESFISMDPPRHAAQRKTVAPMFTPTHLDNLALSIRQRSAECLDN
LPRGEVFDWVDHVSIELTTQMLAVLFDFPWEDRRKLTRWSDIATTIPGPDGLVATEDERQAELTECAGYFARL
WKERIAQPPKSDLLSMMAHGAATRDMDAKNFLGNLILLIVGGNDTTRNTMSGSLHALSQHPEQYRKLRENPAL
LDSFVPEVIRWQTPLAHMRRTALADFEFRGKQIKKGDKVVMWYVSGNRDADAIEKPYEFIIDRARPRTHLSFG
FGIHRCVGLRLAELQLKIIWEEILKRFDHIEVVGEPKRVYSSFVKGLETLPVKIAA

>CYP153A4(blr_7243)*Bradyrhizobium diazoefficiens* USDA 110

MDGRRRRPMPLPQAGEVRKTTGATTMNIQTPVKVDKAERMRRARGEAYATPLAQFHPGAPRLFQDDTLWPWFE
RLRKEEPVHYCTNAPIEPYWSVVKYNDIMHVDTNHGIFSSDSTLGGISIRDVPEGYDYPSFIAMDQPRHSAQR
KTVSPMFTPTHLDELAKLIRQRSQTVLDNLPRNETFNFVERVSIELTTQMLATLFDFPWEERRKLTRWSDVST
ALPKSGIVASAEERRREMDECYAYMSKLWNERVNSAPRNDLLSLMAHNDATRFMDPDNLMGNIILLIVGGNDT
TRNTMTGSVLALNENPEQYDKLRANPALIDSMVPEVIRWQTPLAHMRRTALQDTEIGGKQIKKGDRVVMWYVS
GNRDEEAIDRPNEFIIDRPRPRTHLSFGFGIHRCVGMRLAELQLKIVWEEMLKRFDRIEVVGEPKRIYSSFIK
GYESLPVRIPG

>CYP153A4(S23_58670)*Bradyrhizobium* sp. S23321

MNIQTPVKTDKAERMRRAREEAYATPLAQFHPGAPRLFQDDTLWPWFERLRKEEPVHYCTNAPIEPYWSVVKY
DDIMHVDTNHGIFSSDSTLGGIGIRDVPQGYDWPSFIAMDQPRHSSQRKTVSPMFTPAHLDELAKLIRQRSQT

VLDNLPRNETFNFVERVSIELTTQMLATLFDFPWDERRKLTRWSDVATALPKSGIVASAEERRREMDECYAYM
SKLWNERVNSAPRNDLLSLMAHNDATRFMDPDNLMGNIILLIVGGNDTTRNTMTGSVLALNENPEQYDKLRAN
PELIDSMVPEVIRWQTPLAHMRRTALQDTEIGGKHIKKGDRVVMWYVSGNRDEEMFEKPNEFIIDRPRPRTHL
SFGFGIHRCVGMRLAELQLRIVWEEMLKRFDRIEVVGEPKRIYSSFIKGYESLPVRIPG

>CYP153A4(BF49_2773)*Bradyrhizobium* sp. BF49

MNIQAPVQVDKAERMRRAREEAYATPLAQFHPGAPRLFQDDTLWPWFERLRKEEPVHYCTNAPIEPYWSVVKY
DDIMHVDTNHGLFSSDSTLGGIGIRDVPEGYDWPSFIAMDQPRHSSQRKTVSPMFTPTHLDELAKLIRQRSQT
VLDNLPRNETFNFVERVSIELTTQMLATLFDFPWEERRKLTRWSDVATALPKSGIVASAEERRREMDECYAYM
SRLWNERVNSAPRNDLLSLMAHNDATRFMDPDNLMGNIILLIVGGNDTTRNTMTGSVLALNENPEQYDRLRAN
PELIDSMVPEVIRWQTPLAHMRRTALQDTEVGGKHIKKGDRVVMWYVSGNRDEEMFERPNEFVIDRPRPRTHL
SFGFGIHRCVGMRLAELQLKIVWEEMLKRFDRIEVVGEPKRIYSSFIKGYESLPVRIPG

>CYP153A4(BJ6T_21490)*Bradyrhizobium japonicum* USDA 6

MNIQTPVKTDKAERMRRAREEAYATPLAQFHPGAPRLFQDDTLWPWFERLRKEEPVHYCTNAPIEPYWSVVKY
NDIMHVDTSHGIFSSDSTLGGIGIRDVPQGYDWPSFIAMDQPQHSAQRKTVSPMFTPTHLDELAKLIRQRSQT
VLDNLPRNETFNFVERVSIELTTQMLATLFDFPWDERRKLTRWSDVATALPKSGIVASAEERRREMDECYAYM
SKLWNERVNSAPRNDLLSLMAHNDATRFMDPDNLMGNIILLIVGGNDTTRNTMSGSVLALSENPEQFDKLRAN
PELIDSMVPEVIRWQTPLAHMRRTALADTEIGGKHIKKGDRVVMWYVSGNRDEEMFEKPNDFIIDRPRPRTHL
SFGFGIHRCVGMRLAELQLRIVWEEMLKRFDRIEVVGEPKRIYSSFIKGYESLPVRIPG

>CYP153A4(RN69_10525)*Bradyrhizobium japonicum* E109

MNIQTPVKTDKAERMRRAREEAYATPLAQFHPGAPRLFQDDTLWPWFERLRKEEPVHYCTNAPIEPYWSVVKY
NDIMHVDTSHGIFSSDSTLGGIGIRDVPQGYDWPSFIAMDQPQHSAQRKTVSPMFTPTHLDELAKLIRQRSQT
VLDNLPRNETFNFVERVSIELTTQMLATLFDFPWDERRKLTRWSDVATALPKSGIVASAEERRREMDECYAYM
SKLWNERVNSAPRNDLLSLMAHNDATRFMDPDNLMGNIILLIVGGNDTTRNTMSGSVLALSENPEQFDKLRAN
PELIDSMVPEVIRWQTPLAHMRRTALADTEIGGKHIKKGDRVVMWYVSGNRDEEMFEKPNDFIIDRPRPRTHL
SFGFGIHRCVGMRLAELQLRIVWEEMLKRFDRIEVVGEPKRIYSSFIKGYESLPVRIPG

>CYP153A5(Rpal_1803)*Rhodopseudomonas palustris* TIE-1

MHGTIETGKAARLRAAREEAYATPLKDFHPGAPRHFRDDTLWPWFERLRAEEPVHYCTNAPIEPYWSVTKYND
IMHVDTNHQIFSSDSTLGGISIRDAPVGYDWPSFIAMDEPRHSAQRKTVSPMFTPQHLDELAVLIRGRTQKVL
DGLPRGETFNFVDRVSIELTTQMLATLFDFPFDERRKLTRWSDVATALPKSGVVDSEQQRRDELNECAAYFAR
MWNERVNSEPRNDLLSMMAHHDATRTMDRDNLIGNILLLIVGGNDTTRNTMSGSVLALNENPHEFEKLRANPK
LIDTMVPEVIRWQTPLAHMRRTALQDTELGGKTIRKGDRVVMWYVSGNRDDEVIERPEEFIIDRARARIHLSF
GFGIHRCVGMRLAELQLRIVWEEMLKRFERIEVVGEPKRVYSSFVKGYESLPVRVS

>CYP153A5(Rpdx1_3910)*Rhodopseudomonas palustris* DX-1

MHGTIESGKAARLRAAREEAYATPLKDFHPGAPRHFRDDTLWPWFERLRKEEPVHYCTNAPIEPYWSVTKYND
IMHVDTSHQIFSSDSTLGGISIRDAPVGYDWPSFIAMDEPRHSAQRKTVSPMFTPDHLDELAVLIRGRTQKVL
DGLPRGETFNFVDRVSIELTTQMLATLFDFPFDERRKLTRWSDVATALPKSGVVESEQQRRDELNECAAYFAR
MWNERVNSEPRNDLLSMMAHHDATREMDRDNLIGNILLLIVGGNDTTRNTMSGSVLALNENPHEFDKLRANPK
LIDTMVPEVIRWQTPLAHMRRTALVDTELGGKTIRKGDRVVMWYVSGNRDDEVIERPDEFIIDRPRARIHLSF
GFGIHRCVGMRLAELQLRIVWEEMLKRFERIEVVGEPKRVYSSFVKGYESLPVQVS

>CYP153A9(blr_1853)*Bradyrhizobium diazoefficiens* USDA 110

MNRRLEIHRADDGYIIPLSELDVSEGKRFQDDSIWGCFERLRREDPVHYCQNSAHGPYWSITKYRDIVAVDTN
HHAFSSQQGVTIVEVPDKHWTPSFIKMGPPQHAEQRNTVSPIVGPESLTRLETLIRSRVRMILDGLPRNEVFN
WVTKVSIELTTQTLATLFDFPFEDRRLLTYWSDAAVTTPKAGYAIDSWDKRSTILSECLDYFTRLWNERINAE
PRLDLISLMAHSPVTRHMEPTEFLGNLILLIVGGNDTTRNSITGGLLFMSQYPSELRKLTDNPKLISSAVSEI
IRYQTPIAHMRRTAAIDSIVGGKPIRTGDKVVMWYISGNRDEEVIENANSFVIDRKNVRQHLSFGFGIHRCLG
RHLAELQLRVLWEEILDGGLKIKVVGEPERIASNFVHGYSALPVRIEA

>CYP153A9(RN69_38720)*Bradyrhizobium japonicum* E109

MEIHRADDGYIIPLSELDVSEGKRFQDDSIWGCFERLRREDPVHYCQNSAHGPYWSITKYRDIVAVDTNHHAF
SSQQGVTIVEVPDKHWTPSFIKMGPPQHAEQRNTVSPIVGPESLTRLETLIRSRVRMILDGLPRNEVFNWVTK
VSIELTTQTLATLFDFPFEDRRLLTYWSDAAVTTPKAGYAIDSWDKRSTILSECLDYFTRLWNERINAEPRLD
LISLMAHSPVTRHMEPTEFLGNLILLIVGGNDTTRNSITGGLLFMSQYPSELRKLTDNPKLISSAVSEIIRYQ
TPIAHMRRTAAIDSIVGGKPIRTGDKVVMWYISGNRDEEVIENANSFVIDRKNVRQHLSFGFGIHRCLGRHLA
ELQLRVLWEEILDGGLKIKVVGEPERIASNFVHGYSALPVRIEA

>CYP153A9(BJ6T_79720)*Bradyrhizobium japonicum* USDA 6

MPDKHWTPSFIKMGPPQHAEQRNTVSPIVGPESLTRLETLIRSRVRMILDGLPRNEVFNWVTKVSIELTTQTL
ATLFDFPFEDRRLLTYWSDAAVTTPKAGYAIDSWDKRSTILSECLDYFTRLWNERINAEPRLDLISLMAHSPV
TRHMEPTEFLGNLILLIVGGNDTTRNSITGGLLFMSQYPSELRKLTDNPKLISSAVSEIIRYQTPIAHMRRTA
AIDSIVGGKPIRTGDKVVMWYISGNRDEEVIENANSFVIDRKNVRQHLSFGFGIHRCLGRHLAELQLRVLWEE
ILDGGLKIKVVGEPERIASNFVHGYSALPVRIEA

>CYP153A15(HNE_2042)*Hyphomonas neptunium* ATCC 15444

MTEREPLHPHNLRFGANIPPAADIDLSTLDLIDGELWRQGKYWDRFERLRNEDPLHYCPDSFPGPFWSVTRYE
DVMAIDTDHKRFSSSWEYGGITLGEPISDFEMPMFIAMDEPRHSEQRKTVQPAVAPDMLKVYEPLIRSRTQGL
LDSLPVNEPFDWVDKVSVELTTMMLATLFDYPFENRRDLTHWSDVATGMHNPDICPGGEEEWKATMMKCLMTF
MGIYQERQQQPQKPDLMSLLAHGEKTKNMTPMELLGNVILLIVGGNDTTRNSMTASVFALNKFPKEYEKLKAD
PSIIPNMVSETIRWQTPLAYMRRTALEDVEMHGKTIKKGDQVAMWYVSANRDERFWDKPNDFIADRPEARRHI
SFGFGIHRCVGNRLAELQLRILWEEIMERFEHIEVLAEPSLNQNAFVKGYNWMPVVLHPK

>CYP153A26(Plav_0025)*Parvibaculum lavamentivorans*

MSDAAVKYEADEARDEAYSIPLAEINVADPSLFRDYKMWPYFERLRAEAPVHYSQGNEDTGPYWSVTRYNDIM
SVDTNHQVFSSEGGITLRDQDEDFKLPMFIAMDPPKHDDQRKVVSPIVAPGNLAKLEGTIRERAGKILDELPT
GEPVDWVDRVSIELTTQMLATLFDFPFEERRKLTRWSDVATATEESGIIESEEQRRAELLECAEYFMGLWNER
VNAREPGNDLISMLAHGESTRNMDKMEYLGNLILLIVGGNDTTRNSISGGLYALNKFPDQYKKLLANPGIIPN
MVPEIIRWQTPLAYMRRTALQDAEVGGQTIRKGEKVAMWYVSGNRDESVIQNPNELVIDRERPRQHLSFGFGI
HRCVGNRLAEMQLRIIWEEILKRWDQPIRVLSEPQRVHSSFVKGYEKMMVELPKRKH

>CYP153A27(Plav_1765)*Parvibaculum lavamentivorans*

MSETLVAARDKSIADAYAIPLEKIDVSNPELFRANAIWPYFERLRREDPVHYCKESEYGSFWSVTKYKDIMHV
DTNHGIYSSEATLGGVALRNQEEGFFLPMFIMMDPPKHDAQRKVVSPIVAPGNLAKLEGTIRERAGNILDSLP
VNETFDWVDRVSIELTTQMLATLFDFPWEERRKLTRWSDVAAAGTAFGDEETEKARRNELRDCAAYFTELWNQ
RVNATEPGNDLITMLAQGEATKNMGPMEYLGNVLLLIVGGNDTTRNSITGGLLALNENPVQYKKLRDNPSLVE
SMVPEIIRWQTPLSHMRRTALQDTELGGKQIKKGDKVVMWYVSGNRDEEAIENANSFIIDRKHPRQHLSFGFG
IHRCVGNRLAEMQLRVVWEEILKRWPDKPIEVVGEPTRVFSNLIKGYSSMPVRIPG

>CYP153A29(Plav_1782)*Parvibaculum lavamentivorans*

MTTDTLVGDTASTVPLSQMDVSDPKLFQSDMIGSYFARLRREDPVHYCPESAYGPYWSVTKFNDIMQVEVNHQ
TFSSEAKLGGIALQDMQSGEAALELEMFIAMDPPKHDAQRKAVSPAVAPSNLVLLEPIIRERAGIILDSLPIG
EEIDWVDRVSIELTTMTLATLFDFPWEERRKLTRWSDVATSTPETGVVSSFEQRREELLECAHYFKGLWDQRV
NEPPKPDLISMMVHSPATRDMPYLEFLGNLILLIVGGNDTTRNSISGGVLALNQNPAEYRKLMADPDLIPKMI
PEIIRWQTPLTHMRRTALMDAEIGGKKIRKGDKVVMWYLSGNRDDEVIDRPNEFIINRPNSRHHLSFGFGVHR
CMGNRVAELQLRIIWEEILKRFSKVEVVGAPERTLSNFIRGFTHLPVKLYVH

>CYP153A30(Plav_1951)*Parvibaculum lavamentivorans*

MSEQVSVGRDYTRSNADLAAKWKIDVNRDPWSIPLEELDPAHDELFAANTVLPYFERLRKEDPVHLNETGPYG
RYWSVTKYEDIMHVDTNHKIFSSDIRNGGIRLGGQRIEGEPDPLTYLPMFIMEDPPKHDEQRKAVQPMFTPQN

LADLEPLIRERAGLILDALPRGETFNWVRQVSVELTGRTLATLFNVPQEDRHKLIHWSDTVERLGDPEYFETP
EEGFKELWNCWEYFDGVWKDRLANPGSDLISLLAHSPSTKNMPPNEYLGNMLLLIVGGNDTTRNSITGGVLAL
NQYPDEYAKLIANPDIIPNMVSEIIRWQSPVAHMCRTATEDTELGGKKIRKWDKVAMWYVSGNRDDSKIDRAN
EFLIDREGARHHLSFGFGIHRCIGNRVGEMQLRILWEEIMKRFKKVEVVGDPKYLRSNFIRGITELPVIVRE

>CYP153A31(Plav_2128)*Parvibaculum lavamentivorans*

MTATASAAAPAGIDDPYALSLDQIDVSKPELFERNAEGAYFARLRREDPVHFCADSAYGPYWSITKYKDIMAV
DTNHQVFSSEAGLGGIIIEDGIQKSSGEGSIDLPNFIAMDPPKHDGQRKAVSPIVAPANLANLEGTIRERVGR
VLDGLPVGEEFDWVPAVSIELTTQMLATLFDFPFEERSKLTRWSDVATAEPGSGIVDSWEQRTSEIMECAECF
QGLWNERMEKPGMDLVSMMAHSPATHDMTPQNYLGNVLLLIVGGNDTTRNSMTGGVLALHQNPAEFAKLKANH
GLIDSMVSETIRWQTPLAHMRRTALADAEVGGKTIRKGDKVVMWYVSGNRDKDVIIQPDDFIIDRERPRQHLS
FGFGVHRCVGNRLAEMQLKILWEEILKRFSRIEVTSEPVRTRSNFVRGYASMPVRLHA

>CYP153A77(TQ29_05625)*Confluentimicrobium* sp. EMB200-NS6

MAWLCTCRSNNSDCRVKEKAMTSIKDIPLSDLDPSVQTRFQSGEHFEVFDRLRAEAPVYFHETTQYGPFWSIS
KYKDIIEVEANPKVFSSDAAYGGIWIKEQPKDTIRKSFMTADQPEHDQQRKVVNPIVSPSNLNDMEQKVRDIV
TLVLDDLPLNEEIDWVERVSVEVTGRVLCELMDFPVEERMDLTTWSDIVNTDVDAGTEITDEYVKLEKLKPMI
IKFKELFAERTGMPPQNDLISMLAHSQETANMEPQQFVGMLVLLMVGGNDTTRNSISGGLDALNKFPEEYKKL
RENPELVESMVPEIIRWVTPVAHMRRTALEDIEFHGQQIKKGDKVIMWYCSGNRDPEVIEDPYTFKIDRKNPR
QHVSFGFGIHRCLGNRMAELQLRVLWEEILKRGWVIETTGEPERRFSNFVVGIDSLKAIIRKPE

>CYP153A78(JI59_24805)*Novosphingobium pentaromativorans*

MSRFVAGIPLAEIDVSRPSLFQSDKIGAFFERLRREDPVHYCAESSYGPYWSITRYDDIMAVDTNHKVYSSEA
KLGGIAIQDMHRDQSNLELEMFIAMDQPKHDAQRKAVTPAVAPSNLLLLEPVIRERAGAILDALPVGEEIDWV
KCVSVELTTMTLATLFDFPWDERARLTRWSDVTTAIPGAGIVDSFEQRRAELIECAMYFKGLWDQRIDQPEGN
DLISMMANSPATRDMPFLEFLGNLLLLIVGGNDTTRNSISGGVLALNRHPDQYDKLRRDPAVISSMVPEIIRW
QTPLTHMRRTALEDSEIGGKRIAKGDKVVMWYLSGNRDETAIDRPEEFIIDRKNPRQHLSFGYGIHRCMGNRL
AELQLRIIWEEIQKRFDFVEVVGEPERLLSNLVRGITRLPVKLHAH

>CYP153A78(AB433_17955)*Croceicoccus naphthovorans*

MTTLTISPDQDSAELSRFVAGIPLAEIDVSRPSLFQSDKIGAFFERLRREDPVHYCAESSYGPYWSITRYDDI
MAVDTNHKVYSSEAKLGGIAIQDMHRDQSNLELEMFIAMDQPKHDAQRKAVTPAVAPSNLLLLEPVIRERAGA
ILDALPVGEEIDWVKCVSVELTTMTLATLFDFPWDERARLTRWSDVTTAIPGAGIVDSFEQRRAELIECAMYF
KGLWDQRIDQPEGNDLISMMANSPATRDMPFLEFLGNLLLLIVGGNDTTRNSISGGVLALNRHPDQYDKLRRD
PAVISSMVPEIIRWQTPLTHMRRTALEDSEIGGKRIAKGDKVVMWYLSGNRDETAIDRPEEFIIDRKNPRQHL
SFGYGIHRCMGNRLAELQLRIIWEEIQKRFDFVEVVGEPERLLSNLVRGITRLPVKLHAH

>CYP153A78(PP1Y_AT31178)*Novosphingobium* sp. PP1Y

MAVDTNHKVYSSEAKLGGIAIQDMHSDQSNLELEMFIAMDQPKHDAQRKAVTSAVAPSNLLLLEPVIRERAGA
ILDALPVGEEIDWVKCVSVELTTMTLATLFDFPWDERARLTRWSDVTTAIPGAGIVDSFEQRREELIECAMYF
KGLWDQRIDQPGGNDLISMMANSPATRDMPFLEFLGNLLLLIVGGNDTTRNSISGGVLALNKHPDQYEKLRRD
PALIGSIVPEIIRWQTPLTHMRRTALEDSEIGGKRIAKGDKVVMWYLSGNRDETAIDRPEEFIIDRKNPRQHL
SFGYGIHRCMGNRLAELQLRIIWEEIQKRFDFVEVVGEPERLLSNLVRGITRLPVKLHAH

>CYP153A79(BSY18_3828)*Blastomonas* sp. RAC04

MAAQAIPTDLDEADLARAIAAIPLAEIDVARPSLFQSDKIGAFFERLRREDPVHYCAESAYGPYWSITRYNDI
MAVDTNHKAFSSEAKLGGIAIQDMHSDQSNLELEMFIAMDQPKHDDQRKAVTPAVAPSNLLLLEPVIRERAGA
ILDSLPVGEEIDWVQSVSVELTTMTLATLFDFPWDERARLTRWSDVTTAIPGAGIVGSFEQRRKELIECAMYF
KGLWDQRIDQPGGNDLISIMANSPATREMPFLEFLGNLLLLIVGGNDTTRNSISGGVLALNRHPQQYDKLRRD
PGLIGSMVPEIIRWQTPLTHMRRTALEDSEIGGKRISKGDKVVMWYLSGNRDETVIDRPEEFIIDRKNPRQHL
SFGYGIHRCMGNRLAELQLRITWEEIQKRFHFVEVVGEPERLLSNLVRGITRLPVKLHAH

>CYP153A80(Hbal_0836)*Hirschia baltica*

MPDSSCVIGPDIGASRGGNEILEAVSRGRFNTGGVIAGPDQLDMDKVFLLHPELWRRGEYFPYFKWLRDNSPV
HYTAESPEGPFWSITRYEDIVAVDKNHQQFSSDSKYGGITIADLNQGFQLPMFIAMDRPEHTRYRKAVQPVMD
AASLKNFEHLIRERTIQTLDSLPIHEPFDFVDKVSVELTTMMLATLFDFPQKDRRKLSRWSDIATNKYNPEIC
PGGEAQWQKELTECLMYFMTLWQERGQREEPGHDLLSALAHDANTKNMTPQELLGNLILLIVGGNDTTRNSMT
GSVYAWNLFPKEFEKVKADRSLVPNLAAETLRWQTSLAYMRRTAVEDVEMHGKLIKKGDKVLMWYVSGNRDER
AIERPDEIWIDRPNVRRHLAFGFGIHRCVGNRLAEMQMSILWEEILKRYSYIEVLQEPERLPNSFVKGYKSMN
VMLHPA

>CYP153A81(Cseg_0011)*Caulobacter segnis*

MSDGSVDLKEAARAQAYATPLEDIHVANPALFQADAMWPYFERLRKEAPVHYSQGDEETGPYWSITRYNDIMT
VDTTHQVFSSDAHLGGITIRNFDEDFVLPMFIAMDPPKHDIQRKTVSPIVSPQNLGRLEGIIRERVQRILDDL
PVGEPFDWVDKVSIELTTQMLATLFDFPWEERRKLTRWSDVATASPESGIVESEDARRAELLECLGYFTNLWN
ERVNQTEPGSDLISMLAHGEATRDMPPMEYLGNVILLIVGGNDTTRNSITGGLYALSKNPQEEAKLRADPSLI
PNMVSEIIRWQTPLAHMRRTALEDYELSGQTIKKGDKVVMWYVSGNRDDTVIEDADKFIIDRPSARRHLSFGF
GIHRCVGNRLAEMQLRIVWEEILQRFPRIEVLEEPRRVYSTFVKGYERMMVRIPERV

>CYP153A82(Caul_0020)*Caulobacter* sp. K31

MSDGSIDFGDDARAKAWSIPLEDYHVADPALFQADAMWPYFERLRKEDPVHWSRGIEETGPYWSITKYNDIMA
VDTNHQVFSSDAHLGGITIRDFDEDFVLPMFIAMDPPKHDIQRKTVSPIVSPQNLARLEGIIRERVCTILDGL
PIGETFDWVDKVSIELTTQMLATLFDFPWEERRKLTRWSDVATASPESGIIESEEARRAELLECLAYFTNLWN
ERVNATEPGDDLISMLAHGEATRDMPPMEYLGNIILLIVGGNDTTRNSLTGGLYALSKNPEQEAKLRADPELI
PSMVSEIIRWQTPLAHMRRTALADIELGGKQIRKGDKVVMWYVSGNRDDTVIENPDAFIIDRENPRRHLSFGF
GIHRCVGNRLAEMQLKIVWEEILKRFPKIEVLGEPKRVYSSFVKGYESLPVRIPTRL

>CYP153A83(AEM38_08110)*Hyphomonadaceae bacterium* UKL13-1

MADGNFNPAEFKSVQEHHAKRAWSIPLEEIDVAMPELWTHDCHWPYFERLRKEDPIHWCESQEEKVGGYWSVT
RYKDIMAVDTNHHQFSSDAFLGGITIVGFDEDFILPMFIAMDPPKHDAQRKVVQPIVGPDNLAKMESTIRERV
NLIFDELPIDQEFDWVDKVSVELTTQMLATLFDFPWEDRRKLTRWSDVATAQPIPGGLIETEDQRRAELFECL
AYFTNLWNERVNNSGGNDLISMLAHGEATRNMEPLEYLGNLILLIVGGNDTTRNSLTGGLYALSKNPEEFKKL
RADPSVIPNMVSEIIRWQTPLAHMRRTALEDVELGGKQIKKGDKVVMWYVSGNRDDEMIERPNDFWIDRPRAR
NHLSFGFGIHRCVGNRLAEMQLRIVWEEALKRYSDIQVVSEPTRIRSAFVKGYESMRVILRK

>CYP153A84(Caul_5296)*Caulobacter* sp. K31

MSDGAIDLRAEARAKAYAMPLEDINLADTELWRTDTVWPYLERLRKEDPVHLHPAHHHPDGAFWSITKYADIM
AVDINHEVFSSEPSITIFDPKEDFTLPMFIAMDPPKHDVQRKTVSPIVSPANLHLMEPLIRSRITKTLDELPI
GEPFDWVDRVSIELTTQMLATLFDFPWDERRKLTRWSDIATAVPESGLFVTDDYETERRMELFGCVDYFTRLW
NERVNAPPKGDLISMLAHGEATRNMDRMEYLGNLLLLIIGGNDTTRNTMTGSILAMNQNPDQLRKLRENPSLI
PSMVSETIRWQTPLSNMRRTATQDFELGGKLIKKGDKVLIWYASGNRDEEAIENPEAYIIDRERPRNHLSFGF
GIHRCVGNRLAELQLRILWEEILPRFPEIQVLEDPQRVPSVLIRGYSSMPVMIPARS

>CYP153A85(PHZ_c0813)*Phenylobacterium zucineum*

MDDASIDLQRAARDAAYSMPIEEINPADPELFRTDTMWPYFERLRKEDPVHWGVSPHEDVGGYWSVTKYNDIM
AVDTNHEVFSSEPTIVLPDPADDFTLPMFIAMDPPKHDVQRKTVQPIVAPNHLAYLEPIIRERAGKILDDLPI
GEEINWVDKVSIELTTMTLATLFDFPWEDRRKLTFWSDVATSAPESGILGTTDPEEHENLRRQTLFECVDYFM
RLWNERVNAPPKPDLISMLAHGESTKNMDRMEYLGNLILLIVGGNDTTRNTISGSVLALHQNPDQDRKLRENP
GLIPAMVSETIRWQTPLAYMRRRAKRDFELGGKTIREGDKVAMWYVSGNRDEEVIDRPNDYWIERPRVRQHLS
FGFGVHRCVGNRLAELQLKIIWEEILARFPRLEVVGPPRRVYSSFVKGYEELPVVIPTRN

>CYP153A86(SKP52_24460)*Sphingopyxis fribergensis*

MATSAVHNQTVDSIPLGEINVANPYLFQNDTVGEYFSRLRREDPVHYCAESRFGPYWSVTKFNDIMAVDTNHK
VFSSEAKLGGISVQDMHSDESALELEMFIAMDQPKHDQQRKAVSPVVAPSNLLLLEPIIRERAGSILDALPVG
EEIDWVKLVSVELTTMTLATIFNFPWEERAKLTRWSDVTTASPETGIVESFEQRREELIECAMYFKGLWDQRI
DQPGGNDLISMMAHSPATRDMPFLEFLGNLLLLIVGGNDTTRNSISGGVLALNQNPGEYQKLRNNPALVSSMI
PEIIRWQTPLTHMRRTALEDVKIGGKQIRKGDKVVMWYLSGNRDDTVIDRAEEFIIDRENPRHHLSFGFGIHR
CMGNRLAELQLRIIWEEILKRYSFVEVVGEPERLLSNLVRGITRLPVKLHVH

>CYP153A87(S58_15730)*Bradyrhizobium oligotrophicum*

MHGSTITAETRRHAATGKPAIDEAASRAASLPLAEFDPGHPELFRTDTHWPYFDRLRREEPVHYCKNSMFGPY
WSITRYDDIMEIETNHAVFSSASALGGITIRDVDPELRRESFIAMDPPRHAAQRKTVAPMFTPTHLDELAINI
RKRSAECLDNLPVGEVFDWVDRVSIELTTQMLAVLFDFPWEDRRKLTRWSDIATTIPGPDGLVATEDERQAEL
MECARYFARLWQERASQPPKSDLLSMMAHSEATRNMDPKNFLGNLVLLIVGGNDTTRNTMSGSIYALSQHPEQ
YRKLKDNPALIDSFVPEVIRWQTPLAHMRRTALEDFEFRGRQIRKGDKVVMWYVSGNRDETSIERPYDFIIDR
IRPRQHLSFGFGIHRCVGLRLAELQLKIIWEEILRRFDHIDVMDEPKRVYSSFVKGYETLPVRIAV

>CYP153A88(S58_15720)*Bradyrhizobium oligotrophicum*

MTVQAALRSDHADRLRDARLEAYSTPIEDFHPGAPRLFQNDTIWPWFERLRKEAPVHYCTKAPIEPYWSVTRY
HDIMHVDTNHAIFSSDVSHGGITIRDVAPGYEWPSFIAMDEPVHGEQRKTVSPMFTPDHLDELAVLIRQRSAK
VLDALPRNETFNWVEKVSIELTTQMLATLFDFPFEERRKLTRWSDVSTALPKSGIVSSEEERRRELAECAETF
GRMWKARQAEPGRDLISMMAHSEATRHMTPDNLMGNLILLIVGGNDTTRNSMTASVLALNENPAEYQKLRDNP
ALVETMVPEVIRWQTPLAHMRRTALVDTELGGKTIKKGDRVVMWYVSGNRDEEVIENPDAFIIDRKRPRIHLS
FGFGIHRCVGMRLAELQLRIVWQEILKRFDRIEVVGEPKRVYSSFVRGYESLPVRIPG

>CYP153A89(RPB_3934)*Rhodopseudomonas palustris* HaA2

MHGTIEIDNAARQRAAREEAYATPLSQFHPGAPRHFRDDTLWPWFERLRAEEPVHYCTNAPIAPYWSVTKYND
IMHVDTSHQIFSSDSTLGGISIRDAPQGYDWPSFIAMDEPRHSAQRKTVSPMFTPDHLDELAVLIRGRTQKVL
DGLPRNETFNFVERVSIELTTQMLATLFDFPFAQRRKLTRWSDVATALPKSMIVASEEERRSELNECAATFAA
MWNERVNSEPRNDLLSMMAHHDATRQMDRDNLIGNILLLIVGGNDTTRNTMTGSVLALNQNPDQFAKLRANPA
LIDTMVPEVIRWQTPLAHMRRTALQDTELGGKTIKKGDRVVMWYVSGNRDDEVIERPNEFIIDRKRAKIHLSF
GFGIHRCVGMRLAELQLKIVWEEMLKRFERIEVVGEPKRVYSSFVKGYESLPVRIS

>CYP153A89(RPD_3694)*Rhodopseudomonas palustris* BisB5

MHGTIEIDKAARQRAAREEAYSTPLAQFHPGAPRHFRDDTLWPWFERLRAEEPVHYCTNAPIEPYWSVTKYND
IMHVDTHHGIFSSDSTLGGIAIRDAPVGYDWPSFIAMDEPRHSAQRKTVSPMFTPQHLDELAVLIRGRTEKVL
DALPRNETFNFVERVSIELTTQMLATLFDFPFEERRKLTRWSDVATALPKSLIVASEEERRTVLNECAATFIK
LWNERVNSEPRNDLLSMMAHHDATRQMDRDNLIGNILLLIVGGNDTTRNTMSGSVLALNENPDQFAKLRANPA
LIDTMVPEVIRWQTPLAHMRRTALEDTELGGKTIKKGDRVVMWYVSGNRDDEVIERPNEFIIDRKRPKIHLSF
GFGIHRCVGMRLAELQLKIVWEEMLKRFDRIEVVGEPKRVYSSFVKGYESLPVRIS

>CYP153A90(RPE_4309)*Rhodopseudomonas palustris* BisA53

MHGSIESGKAARLRAAREEAYATPLKDFHPGAPRLFRDDTLWPWFERLRNEEPVHYCTNAPIEPYWSVTKYHD
IMHVDTHHQLFSSDQSLGGISIRDTPAGYDWPSFIAMDEPRHTAQRKTVSPMFTPDHLDELAVLIRSRTRTVL
DGLPKNETFDFVERVSIELTTQMLATLFDFPFEQRRKLTRWSDVATALPKSGVVESESQRRAEMSECATYFTK
MWNERVNSEPRNDLLSMMAHHDATRTMDHDNLLGNIILLIVGGNDTTRNSMTGSVLALNENPEQFAKLRANPA
LIDTLVPEVIRWQTPLAHMRRTALEDSELGGKTIRKGDRVVMWYVSGNRDEEVIERPDEFIIDRKRPKIHLSF
GFGIHRCVGMRLAELQLRIVWEEMLGRFENIDVVGDPKRVYSSFVKGYEALPVRLS

>CYP153A91(RPC_4264)*Rhodopseudomonas palustris* BisB18

MHGSVDSEKADRLRAARAEAYATPLKDFHPGAPRHFRDDTLWPWFERLRAEAPVHYCTNAPIEPYWSVTKYHD
IMNVDTHHEIFSSDSTLGGISIRDAPQGYDWPSFIAMDQPRHAEQRKTVSPMFTPEHLDELAVLIRQRAGQVL
DALPKNETFNFVEKVSIELTTQMLATLFDFPFDERRKLTRWSDVATALPKSGVVESETQRRTEMSECASYFTK
LWNERINAAPRNDLISMMAHHDATRQMDHDNLIGNIILLIVGGNDTTRNTMTGSVLALNENPDQFRKLRENPA

LLDSMVPEVIRWQTPLAHMRRTALVDSELGGKLIRKGERVVMWYVSGNRDDEAIEAPNDFIIDRARPRTHLSF
GFGIHRCVGMRLAELQLRIVWEEMLKRFDHIQVVGEPRRVYSSFVRGYECLPVRIS

>CYP153A92(SKP52_13370)*Sphingopyxis fribergensis*

MSIDTMELDTAGKVAAIPIDEIDVARPSLFQQDTIGLYFDRLRKEEPVHYCRESYVGPYWSITKFDDIMAVDT
NHKVFSSEAKLGGIAIQDMHSVEGALELEMFIAMDPPKHDQQRKAVTPAVAPSNLLLLEPTIRERACQILDDL
PIGEEFDWVDKVSVELTTMTLATLFDFPWEERRKLTRWSDVTTAAPETGIVESYEARREELIECAMYFKGLWE
QRINEEPKNDLISIMAHSPATRDMPFLEFLGNLLLLIVGGNDTTRNSISGGVLALNQNPDEYRKLNDDPSLIA
SMVPEIIRWQTPLTHMRRTALQDWEIGGKQIKKGDKVVMWYLSGNRDETVIDRADRFIIDRKNPRHHLSFGYG
IHRCMGNRLAELQLRIIWEEIHKRFAKVEVTGEPERLFSNLVRGITNLPVRLHAR

>CYP153A93(SGRAN_1597)*Sphingopyxis granuli*

MDSQIAEPDAAARIASIPIEEIDVARPSLFQNDTIGLFFDRLRAEEPVHYCRESYVGPYWSITKFDDIMAVDT
NHKVFSSEAKLGGIAIQDMHSVEGALELEMFIAMDPPKHDQQRKAVTPAVAPSNLQLLEPIIRKRAGEILDEL
PIGEDFDWVDKVAIELTTMTLATLFDFPWEERRKLTRWSDVATAAPETGIVESYEARREELIGCAMYFKTLWD
ERINEEPKNDLISMMAHSPATRDMPFLEFLGNLMLLIVGGNDTTRNSISGGVLALNQSPDEYAKLDADPSLIS
KMVPEIIRWQTPLTHMRRTALEDWEIGGKQIKKGDKVVMWYLSGNRDESAIERADQFIIDRKNPRHHLSFGYG
IHRCMGNRLAELQLRIIWEEIHKRFSRVEVTGEPERLFSNLVRGITRLPVRLRAR

>CYP153A93(ELI_14945)*Erythrobacter litoralis* HTCC2594

MDSQIAEPDAAARIASIPIEEIDVARPSLFQNDTIGLFFDRLRAEEPVHYCRESYVGPYWSITKFDDIMAVDT
NHKVFSSEAKLGGIAIQDMHSVEGALELEMFIAMDPPKHDQQRKAVTPAVAPSNLQLLEPIIRKRAGEILDEL
PIGEDFDWVDKVAIELTTMTLATLFDFPWEERRKLTRWSDVATAAPETGIVESYEARREELIGCAMYFKTLWD
ERINEEPKNDLISMMAHSPATRDMPFLEFLGNLMLLIVGGNDTTRNSISGGVLALNQSPDEYAKLDADPSLIS
KMVPEIIRWQTPLTHMRRTALEDWEIGGKQIKKGDKVVMWYLSGNRDESAIERADQFIIDRKNPRHHLSFGYG
IHRCMGNRLAELQLRIIWEEIHKRFSRVEVTGEPERLFSNLVRGITRLPVRLRAR

>CYP153C1(Saro_1821)*Novosphingobium aromaticivorans*

MAATLAPDRAINPHDVSLNALYTEDRWREPFRWLRENMPVSYRAESPFGAYWSVVTHDLIQQVELDPGTYSSS
WQRGNITIADSVNETEFPNFIAQDPPIHTAQRKVIAPAFGPSQMVKLERLVRERTTQLLDGLPMGEEFDWVER
VSIPLTLGMLLILFDMPFDEWRDIKRWSDWASGVSEDSLNDAYRAEFVQQMGQMLMRFDRELEARRALPPSDD
LLSRMVHSDAMGHLTPPERIANIALLIVGGNDTTRNSMSGLIEALHRYPAELDKLRADPALSANAAQEIIRWQ
SPVTHMRRTLTRDAELGGQRLAEGDKIVMWYISGNRDENVFPDAERFDVTRENARRHIGFGHGIHRCVGARLA
EVQIAAVIEEIATRRLRITPQGAPTRLASPFLHGFTAMPVVMSRD

>CYP153C2(ELI_09815)*Erythrobacter litoralis* HTCC2594

MATAATDARAIAPVDVSREALYVENRWHEPFAQLRREMPVSWTPESPYGPYWSVVTHELVSKVELDTETFSSS
WERGNITIADPAPETNLPNFIAADPPLHTAQRKVIQPAFAPSQMRIREQQVRERCAYLLDKVPVGETFDWVTE
VSIPQTMGMLCIIFGMDPDAESEDLKRWSDFASGVGENSQTEEYRAEWLTQMHAMLARFDQLLDERRQAEPGD
DLLSRMVHSEAMGNLLPMERLANLALLLVGGNDTTRNSASAIVRGFDMYPEQLDVLYGDASLIPNAAQEVIRW
QSPVLHMRRTTTRDTELAGQHIPEGEKVVLWYISANRDEAVFEDADRFDVARANARRHLAFGHGIHRCVGARL
AEIQIGTLIEEIVGRGWRIVPQGEPTRQASPFLRGFTSMPVKIERRG

>CYP153D1(Saro_0220)*Novosphingobium aromaticivorans*

MATQLAPEVPQFTYHSSPTATEAFAAWLKDNPQAIPAHSHPWDVSRSDIYVEDRWQPIFAEMRAKAPVNRVPD
SPYGAYWNVASHKAIMHVESLPELFSSSWQYGGITIGDPPEDVDPQKLAERQLPMFIAMDRPDHTGQRRTVAP
AFTPAKMVEMEAEIRRRTASVLDSLPWGERFDWVDKVSIELTTGMLAILFGFPWADRRLLTFWSDWAGDVELT
LARELADTRFGFLGEMAHYFQRLWGARMQAPPSGDLISMMIHSEAMNHMSPQEFMGNLVLLIVGGNDTTRNTM
SGIVHALDKFPDQRELLERDASLIPNAVQECIRYVTPLAHMRRTATADTELFGNQIKAGEKVILWYISANRDE
TVFENPDKLMVDRPNARRHLSFGHGIHRCVGARLAELQLRILLEEMHERRMRVRVAGEVERVRANFVHGFRKL
EVELEKR

>CYP153D2(LH19_23840)*Sphingopyxis macrogoltabida* 203

MATVIRETPADPHPLDLSRADLWREDQWQEPMRQLRAESPIYYCEDSKFGPYWSVTTYKPIQHIEALPKIFSS
SWEYGGITVAGDGIEHLKEGEIPMPMFIAMDPPQHTAQRRTVAPAFGPSEIERMRADTQARTAALIDTLPVGE
AFDWVERLSIELTTDMLAILFDFPWENRHNLTRWSDALGDIESFNTLEERQQRLATAFEMGAAFKELWDHKAK
NPGKHDLISIMLQSDAMNHMSHEEFMGNLILLIVGGNDTTRNSMSAYAYGLHCFPEERAKLEANHDPDLAVNA
MHEIIRWQTPLAHMRRTALEDTELFGHQIRARDKIALWYASANRDESVFPDGDRIIVDRENARRHLAFGYGIH
RCVGARVAELQLTSLISEMQKRRLRVNVLAEPERVNASFVHGYRHMQVELERY

>CYP153D20(BG023_11368)*Porphyrobacter* sp. LM 6

MATIAPTPASQGIQRPQVRREPSAYDALKAHFAEHPEERLRHSHPWDVSRSEIYAEDKWQPIFAEMRKAGQLH
WIPESPFGPYWAVVGHKAIQHIEALPETFSSSWEHGGITILNRLSEEELAASNIERRELPMFIAMDRPQHTGQ
RRTVAPKFTPTSIADMEAEIRQRTGELLDSLPRGEVFDWVDTVSIELTTGMLAILFGFPWEDRRLLTFWSDWA
GDTELGAVRALDEVRWGILQEMAAYFQTLWIERTMDKEPGNDLISMMIHSPAMNQMSPEEFMGNLVLLIVGGN
DTTRNTMSGIIHALDKFPDQRKLFEEQPELIPNAVQEILRMQVPLAHMRRTCTEDTEVFGQTIKAGDKVVLWY
ISANRDDEVFENPDKLDITRENARRHLAFGYGIHRCVGARLAELQLRVLLEELHKRRMRVHVAGDVERVRANF
VHGFRKLEVEITTF

>CYP153D21(Ga0102493_11827)*Erythrobacter litoralis* DSM 8509

MATIAPKRPEVRRSPTAYEALKQHYDKHPDERLNHPHKWDVSRSDIYFEDRWQPIFKEMQDAGPLHYIPESPY
GPYWAVVDHKAIQHIEALPETFSSSWEYGGITILERLTEEEAAASNIDTRELPMFIAMDRPQHTGQRRTVAPK
FTPTAMADMEGDIRARTGELLDSLPRGETFDWVDKVSIELTTGMLAILFGFPWEDRRLLTLWSDWAGDTELAT
VRDLDQERWSFLQEMAAYFQSLWVERTQDPSGDDLISMMIRSEAMNQMRPEEFMGNLVLLIVGGNDTTRNTMS
GIVHALDKFPDQRKAFEENPELIPNAVQECLRYITPLAHMRRTCTEDTDVFGQTVKKGDKVVLWYLGANRDEK
VFENPHKLDIRRENARRHIAFGYGIHRCVGARLAEMQLRLLLEEMHKRRMRVHVAGDVERVRANFVHGFRKLE
VEITEF

>CYP153D22(AMC99_02425)*Altererythrobacter epoxidivorans*

MATVAPERPKCRTEPTAYKALQQHLEEHPEDKLDHPHKWDVSRSDIYAENTWHPIFREMRAAGPLHYIPESPF
GPYWAVVGHKAIQHIEALPDIFSSSWEHGGITILERLTDEQLAERGLEERRELPMFIAMDRPQHTGQRRTVAP
KFTPSGMAEMEGEIRQRTGELLDTLPRGEVFDWVDKVSIELTTGMLAILFGFPWEDRRLLTYWSDWSGDTELA
TVRELDDMRWGILQEMAAYFQSLWIERTHDKEPGDDLISMMIHSEAMNQMSPQEFMGNLILLIVGGNDTTRNS
MSGFVYQLDKNPDQRKLFEQDPSVIPNAVQEMLRMQTPLAHMRRTCVEDTEVFGQTIKAGDKVVLWYLSANRD
EEVFENPDKLDLTRENARRHIAFGYGIHRCVGARLAELQLRVLLEEMHKRRMRVHLAGDVERVKANFVHGFRK
LEVEITSF

>CYP153D23(ELI_12445)*Erythrobacter litoralis* HTCC2594

MATIAPQRPECRSEPTAYEALKKHFEEHPEERPQHTHKWDVSRSDIYYENTWHPIFREMREAGPLHYIPESPF
GPYWAVVGHKAIQHIEALPDVFSSSWEHGGITILNRLSDEEREESGVDRRELPMFIAMDRPEHTGQRRTVAPK
FTPSGMAEMEGEIRQRTGELLDSLPRGEVFDWVDKVSIELTTGMLAILFGFPWEDRRLLTFWSDWSGDTELAT
VRELDEMRWGFLHEMAAYFQSLWVERTHDKEPGDDLISMMIHSPAMNQMRPEEFMGNLVLLIVGGNDTTRNSM
SGIIYQLDKNPDQRKLFEQQPELIPNAVQEILRMQTPLAHMRRTCTEDTEVFGQKIKKGDKVVLWYLSANRDE
EVFENPDKLDITRENARRHIAFGYGIHRCVGARLAELQLRVLLEELHKRRMRVHLAGDVERVRANFVHGFRKL
EVEVTEF

>CYP153D24(WG74_00870)*Citromicrobium* sp. JL477

MATTAQSPAGTHQWSRSPSAHKALEAHYAANPDTRPDYPHKWDTSRSDIYFENRWEPIFKEMRAAGPVHYIPE
SPFGPYWSVVGHKAVQHTEALPDIFSSSWEYGGITILDDLPDIPEEERMRLPMFIAMDRPKHTGQRKTVAPAF
TPAEMKRLEDDIRQRTGETLDALPRGEVFDWVNTVSIPLTTGMLAILFDFPWEDRDLLTHWSDWAGDTELATV
RALDETRRGMLHEMAAYFQLLWAERAQKGEAPDLISRMIHSPAMNQMEPQEFMGNLVLLIVGGNDTTRNTMSG
IVHAFDRFPDQRKAFEEDASLIPNAVQECIRYQTPLKHMRRTATQDTELFGQNIAAGDKVLLWYNSANRDESV
FEDAEKLDITRENARRHLAFGYGIHRCVGARLAELQLRVLLEEMHKRRLRVHVAGDVQRVRANFVEGFRKLEV
EVTEF

>CYP153D25(TS85_00425)*Sphingomonas* sp. WHSC-8

MATLAPETAPGSRVDPLDVTRPELYRDDTWHEPFRQLRAEAPVRYTPDSAFGPYWSVSSYKGIVAVESLPELY
SSEAGGITVADLQEGDIKMPMFIAMDRPKHTGQRRTVAPAFTPSEMARMRENIRARSAEILDSLPVGETFDWV
DSVSIELTTQMLAILFDFPWADRRKLTFWSDWAGDIEIAKDPIKKQERLQHLFECAAYFGTLWQSKLGKPPTP
DLISMMIHSDAMSHMDQHEFLGNLILLIVGGNDTTRNSMSAFAYGLDLFPDERAKLEADPGLIPNAVQEIIRW
QTPLSHMRRTATADTELEGQTIRAGDKLALWYVSANRDESVFADADRIRLDRENARRHLAFGHGIHRCVGARL
AELQIGILLEEMARRRLRVNVVGAPERVAACFVHGYRKLPVELSRY

>CYP153D26(BSY18_3190)*Blastomonas* sp. RAC04

MATVFKDPGAELTYDMSDASWYVEDRWQEPFAQLRAHDPIHWTQGGSFGDFWNVTSHKAIQHVEALPEIFSSS
YEFGGITLADRIDDGTELIMPMFIAMDRPKHTGQRRTVAPAFTPSEMKRMSDDIRMRTAELLDTLPWDKPFDW
VDTVSIELTTQMLAILFDFPWADRRKLTEWSDWAGDIELVHSEELRAERLKHLYEMGAYFKNLWDAKINAEPT
PDLISMMIHSDAMAEMDELEFMGNLILLIVGGNDTTRNSMSGLAYCLHQFPEQREKLEQNPSLIPNAVQEIIR
WQTPLAHMRRTALADYDLFGKTIKKGDKLALWYISGNRDETVFEDADSIIVDRENARRHLAFGYGIHRCVGAR
LAELQIAILLEEMAKRRMRVNVLAEPERVRACFVHGYRSMQVQLSKY

>CYP153D27(AM2010_2294)*Altererythrobacter marensis*

MATQFAEQPRQYRTSPIAVEALREHLALHPEEKWSHTHPYDVSRSDIYFEDRWQPIFADMRAKGPIHWVDESP
VGPYWNVVGHKAIGHVEALPELFSSSWEHGGITILNRLDEEQLAAQGRERFELPMFIAMDRPKHTGQRRTVAP
AFTPAEMKRLDDDIRHRTGELLDSLPKGEVFDWVDRVSIELTTGMLAILFDFPWEDRRLLTFWSDWAGDTEIA
MVRDLDEMRQGILREMGAYFQQLWADRANKEPGPDLISMMIHSPAMNQMSPQEFMGNLVLLIVGGNDTTRNTM
SGIVHAFDTFPDQRKLFEENPDLIPNAVQECIRYQTPLAHMRRTCTEDTELFGKKIKAGDKLVLWYLSANRDE
DVFENPDKLDITRENARRHLAFGYGIHRCVGARLAELQLRVLLEEMHARRMRVHVAGDVERVRANFVHGFRKL
EVEVTSF

>CYP153D28(SGRAN_0250)*Sphingopyxis granuli*

MATVIREAPETVNPLDVSRAELWREDRWQEPMRRLRAESPIHYCEDSKFGPYWSVTTYKPIQHVEALPKIFSS
SWEYGGITVAGDGVEHLQEGEVPMPMFIAMDPPQHTAQRRTVAPAFGPSETERMRADTLKRTSDVLDSLPVGE
PFDWVEKLSIELTTQMLAILFDFPWEERHKLTAWSDALGDIESFNTAEERQQRLAIAYEMGAAFKELWDRKAQ
NPGQHDLISIMLQSDAMSHMSHEEFMGNLILLIVGGNDTTRNSMSAYAYGLHQFPEERAKLEANHDPDLAVNA
MHEILRWQTPLAHMRRTATEDTELFGHQIRKRDKIALWYASANRDESVFPDGDRIIVDRENARRHLAFGYGIH
RCVGARVAELQLTALISEMQKRRLRVNVLAEPERVNACFVHGYRHMQVELERY

>CYP153D29(LH20_02570)*Sphingopyxis* sp. 113P3

MATQIRVAPENPDPLDVSRAELWSEDRWQEPMARLRAESPIHYCEDSKFGPYWSVTTYKPIQHVEALPKIFSS
SWEYGGITVAGDGIEHLQEGEVPMPMFIAMDPPQHTAQRRTVAPAFGPSEIERMRADTLARTSAVLDTLPIGE
AFDWVEKLSIELTTQMLAILFDFPWEERHKLTAWSDALGDIESFNTLELRQKRLAIAYEMGAAFKELWDRKAK
APGPHDLISIMLQSDAMSHMSHEEFMGNLILLIVGGNDTTRNSMSAYAYGLHCFPEERAKLEANHDPELAVNA
MHEIIRWQTPLAHMRRTATEDTELFGHQIKKRDKIALWYASANRDESIFPDGDRIIVDRENARRHLAFGYGIH
RCVGARVAELQLTALISEMQRRRLRVNVVGEPERVHASFVHGYRHLPVVLEQY

>CYP153D30(LH20_20865)*Sphingopyxis* sp. 113P3

MATQIRVAPENPDPLDVSRAELWSEDRWQEPMARLRAESPIHYCEDSKFGPYWSVTTYKPIQHVEALPKIFSS
SWEYGGITVAGDGIEHLQEGEVPMPMFIAMDPPQHTAQRRTVAPAFGPSEIERMRADTLARTSAVLDTLPIGE
AFDWVEKLSIELTTQMLAILFDFPWEERHKLTAWSDALGDIESFNTLELRQKRLAIAYEMGAAFKELWDRKAK
APGPHDLISIMLQSDAMSHMSHEEFMGNLILLIVGGNDTTRNSMSAYAYGLHCFPEERAKLEANHDPELAVNA
MHEIIRWQTPLAHMRRTATEDTELFGHQIKKRDKIALWYASANRDESIFPDGDRIIVDRENARRHLAFGYGIH
RCVGARVAELQLTALISEMQRRRLRVNVVGEPERVHASFVHGYRHLPVVLEQY

>CYP153D31(LH19_27210)*Sphingopyxis macrogoltabida* 203

MATQALTKGDLATLDVSRGELYADDSWRPLFAQLRAEIPIHYCPDSVFGPYWSVTRYDDIVAVEADPQTFSSS
FEHGGIVLFDMQDTNVKLAMFIAMDDPEHGAQRKTVAPAFGPSEIAGHAVALRERTARLLDSLPVGETFDWVE
RVSIELTTSMIATLFDWPWDDRHRLPVWSDWTAKIDIGPDPVLNAERETHIFEMAAAFKQLFEERKAMPPKGD
LLSIMAHSEVMADMDEQRFIGAIALLLVGGNDTTRNSMSGLVEQMNKFPDQWERLKADRSLISTAATETIRLQ
TPICHMRRTVVQDTELGGQKLKAGDKVALWYNSANRDEAVFPDGDLWDVGRSNSRRHLSFGYGIHRCLGARLA
ELQLQVLIEELLARDMTVEIAGEPERIPSCFTRGFHHLPVRIRKG

>CYP155J1(S58_40390)*Bradyrhizobium oligotrophicum*

MRIDSFAGAREVLRCPDVRQAGFNAELVARFIGPAHAPVLYQDGEPHQKQRSATARFFAPRVVATRYRELIER
TSHELVARFRAAGRGRLDDMSLELAVAVAADIVGLTESDRARMAVRLNRFFSAPVSGHGTIATLRQALLGQWR
MLSFHWFDVRPAIRVRRRTPREDVISHLIQEGYSDREILTECLTYGAAGMATTREFIVMAAWRLLEREDLRAR
FLQGDEQDRMTILEEVLRLEPVVSTLKRRATRDLVVDSGAGPMAIAANSLVEIEVGAANRDPSAAGPCPHQLD
PDRARPAKASASLMSFGDGPHRCPGAAVALHEAAIFLDQLLRVPGLHLEQVPAMTINPVSSGYELRGAVIAVA
PQR

>CYP155J2(BRADO3466)*Bradyrhizobium* sp. ORS 278

MTAQSSQHASAGAPVGHDGTPVIRIDSFAAARAVLRSGDVRQAGFNAELVHRFIGPRHAPVLYQDGEPHQRQR
SATARFFTPRVVATRYCRLMETTSRELVAQFVTARRARLDDLSLTLAVAVAADIIGLTDSSRAAMAARLNRFF
AAPVGQGAIATLRNAVFGQLRSLAFYWFDVRPAIRARRARPREDVISHLLAEGYSDREILTECLTYGAAGMAT
TREFIVMAGVRLFEDDALRARFLAGNEPQRMAILEEVLRLEPVVSTLKRRTACEMTLESDAGATTIPARSLLE
IDVGAANRDEAAAGACPHRLDPDRTRPAKGSASLMSFGDGPHRCPGAAVALHEAAIFLDQLLRVPGLRLERQP
TKTVNPVSSGYELRDAVIVVG

>CYP155J3(G432_18345)*Sphingomonas* sp. MM-1

MMLVNAQAHSEADCPFHSGDDHRKSARASAGGGIIDADIWTDTFDRARAILRSDGAKQAGFLAEQVGKIGRAT
RQPILFLEGEAHRVRRGATARFFTPRIVATRYRELMIRLSDELVERFRQQKRARLDDLSLELAVAVAGDIVGL
TASDPNGLARRLDIFFTGNFDPPRGRFRALVKFLRSQYRVLSFHYRDVVPAIRARKKKRQDDVISHLIDEEYT
DREILTECLLYGAAGMATTREFIVMAAWHMLERDELRAAFQQADEAGKIAILEEILRLEPVVGYIFRRMRTDL
PAADVRAGNAVAVDVRAANTDETAFGTCPREIMAGREVSAKYGNAGMSFGDGEHRCPGAQVALHESCLFLDRL
LRVPGLKLERAPDLAWNRLVTSYELRNAVIICS

>CYP155K1(Swit_3367)*Sphingomonas wittichii*

MDATIEPDERRTAFGRIPSRIAVERDAAGVWHVRGFAAARALLRGDSTRQAGFKAELIERVPGRGKVPILFLE
GRPHHEQRRLTAPFFTPRAVDARYRPLMARLADRVIGDFRRRRRGELSAMGMEMAVGVAAEVVGLTDSRRPGL
DRRINAFFADGLEQGGGRLRRIRQALVAQFRLLRFYWIDVRPSMAARRAQPREDVISHLLAQGYSGTEVLTEC
ITYGAAGMVTTREFITVCAWHLIDDAALCAQYLAADEPARHALLEELLRLEPVVGTLFRRTTCPVTLGGAEIP
EGAMVAVDVRAVNADPAVSGERPLVRQPGRCPAERAGAPVLSFGDGAHRCPGSFLAIQEADIFLTRLLAIPGL
RFEREPQVGWNDLVTGYELREAWLACD

>CYP173A1(mlr5192)*Mesorhizobium loti* MAFF 303099

MDTQPAPFVPPAPKPRTSPPSTLEMIRIVYRNPLELWGEPTYNEPWISANGVGGHLIVANDPGLIRHVLVDNA
KNYKMATVRQKILRPILRDGLLTAEGEVWKRSRKAMAPVFTPRHIFGFAQPMLKRTKEFVTRYEEGGASDIAH
DMTLLTYDILAETLFSGEIAGEPGSFANEIDRLFETMGRVDPLDLLRAPDWLPRLTRIRGRKTMAYFRKIVTD
TVKMREEKFRRDPDAVPQDFLTLLLKAEGPDGLTRSEVEDNIITFIGAGHETTARALGWTLYCLAESPWERNR
VEQEIDEVLAREPDPTKWLDAMPLTRAAFDEALRLYPPAPSINREPIEPEMWKDLYIPRHAAVLVMPWVVHRH
RKLWDRPDAFLPERFHPGNREKIDRFQYLPFGAGPRVCIGASFAMQEAIIALAILLSRFRFDTTAETKPWPVQ
KLTTQPQGGLPMQVTPR

>CYP173A1(A9174_01555)*Mesorhizobium loti* NZP2037

MDIQPAPFVPPAPKPRTTPPSTLEMIRIVYRNPLELWGEHTYNEPWISANGVGGHLIVANDPGLIRHVLVDNA
KNYKMATVRQKILRPILRDGLLTAEGEVWRRSRKAMAPVFTPRHIFGFAQPMLKRTKEFVTRYEAGGTSDIAH
DMTLLTYDILAETLFSGQIAGEPGSFANEIDRLFETMGRVDPLDLLRAPDWLPRLTRIRGRKTMAYFRKIVTD

TVKMREEKFKRDPDAVPQDFLTLLLKAEGPDGLTRSEVEDNIITFIGAGHETTARALGWTLYCLAESPWERNR
VEQEIDEVLARESDPTKWLDAMPLTRAAFDEALRLYPPAPSINREPIEPEMWKDLYIPKHAAVLVMPWVVHRH
RKLWDRPDAFLPERFHPGNREKIDRFQYLPFGAGPRVCIGASFAMQEAIIALAILLSRFRFDTTAETKPWPVQ
KLTTQPQGGLPMQVTPR

>CYP173A1(Mesau_00295)*Mesorhizobium australicum*

MDTQPAPFVPPAPKPRTSPPSTLEMIRIVYRNPLELWGEPTYNEPWISAKGAGGPLVIANDPGLIRHVLVDNA
KNYKMATVRQKILRPILRDGLLTAEGEVWKRSRKAMAPVFTPRHIFGFVQPMLKRTLEFVTRYEAGGTSDIAH
DMTLLTYDILAETLFSGEIAGEPGSFANEIDRLFETMGRVDPLDLLRAPDWLPRLTRIRGRKTMAYFRKIVTD
TVKMREEKVRRDPDAVPQDFLTLLLKAEGPDGLTRPEVEDNIITFIGAGHETTARALGWTLYCLAESPWERDR
VEQEIDRVLAREPDPAKWLDAMPLTRAAFDEALRLYPPAPSINREPIVPEMWKDLYIPKHAAVLVMPWVVHRH
RKLWDRPDAFLPERFHPGNREKIDRFQYLPFGAGPRVCIGASFAMQEAIIALAILLSRFRFDATAETKPWPVQ
KLTTQPQGGLPMGVTPRPR

>CYP173A1(Mesop_0306)*Mesorhizobium opportunistum*

MDTQPAPFVPPAPKPRTSPPSTLEMIRIVYRNPLELWGEPTYNEPWISANGAGGPLVIANDPGLIRHVLVDNA
KNYKMATVRQKILRPILRDGLLTAEGEVWKRSRKAMAPVFTPRHIFGFAQPMLKRTREFATRYEAGGMSDIAH
DMTLLTYDILAETLFSGEIAGEQGSFANEIDRLFETMGRVDPLDLLRAPDWLPRLTRIRGRKTMAYFRKIVTD
TVKMREEKVRRDPDAVPQDFLTLLLKAEGPEGLTRAEVEDNIITFIGAGHETTARALGWTLYCLAESPWERNR
VEQEIDQVLAREADPTKWLDAMPLTRAAFEEALRLYPPAPSINREPIVPEMWKDLYIPKHAAVLVMPWVVHRH
RKLWDRPDAFLPERFHPGNREKIDRFQYLPFGAGPRVCIGASFAMQEAIIALAILLSRFRFDVTAETKPWPVQ
KLTTQPHGGLPMQVTPR

>CYP173A1(Mesci_0325)*Mesorhizobium cicero*

MDIQPAPFIPPAPKPRTTPPSTLEMIRIVYRNPLELWGEHTYNEPWISANGVGGHLIVANDPALIRHVLVDNA
RNYKMATVRQKILRPILRDGLLTAEGEVWKRSRKAMAPVFTPRHIFGFAQPMLRRTQDFVTRYEDGGTSDIAH
DMTLLTYDILAETLFSGEISGEPGSFANEIDRLFETMGRVDPLDLLRAPDWLPRLTRIRGRKTMAYFRKIVTD
TVKMREAKFKRDPDAVPQDFLTLLLKAEGPDGLTRAEVEDNIITFIGAGHETTARALGWTLYCLAESPWERNR
VEQEIDQVLAREPDPTKWLDAMPFTRAAFDEALRLYPPAPSINREPIEPEMWKDLHIPKHAAVLVMPWVVHRH
RKLWDRPDAFLPERFHPGNREKIDRFQYLPFGAGPRVCIGASFAMQEAIIALAIMLSRFRFDTTAETKPWPVQ
KLTTQPQGGLPMQVTPR

>CYP173A2(SMc02579)*Sinorhizobium meliloti* 1021

MDTRPEPFEPPAPVPRTGIPSRLEIIRTVLRNPLELWGEPSYTLPWIETKFINQRTLIVNDPGLIRYILVENA
ANYEMSNVRRLILRPILRDGLLTAEGEVWKRSRKAMAPVFTPRHAQGFAGQMLRVCEAFVDRYAGASSEPFVT
NVAVDMTELTFEILAETLFSGEIAVEKQGFAANVEELLHRMGRVDPMDLLVAPSWVPRLTRIGGRKVLDRFRG
VVSETMSLRRRRTTEAPGDVPNDFLTLLLQLEGPDGLSTSEIEDNILTFIGAGHETTARALAWCFYCVANTPA
YRETMEQEIDSVLASGADPVDWLGRMPHVLAAFEEALRLYPPAPSINRAAIEEDAWTSPEGERVPIRKGISVL
VMPWTLHRHALYWQKPRAFMPERFLPENRDKINRFQYLPFGAGPRVCIGATFALQEAVIALAVLMHRFRFDLT
DETHPWPVQRLTTQPRGGLPMKVSARVK

>CYP173A2(SM11_chr3525)*Sinorhizobium meliloti* SM11

MDTRPEPFEPPAPVPRTGIPSRLEIIRTVLRNPLELWGEPSYTLPWIETKFINQRTLIVNDPGLIRYILVENA
ANYEMSNVRRLILRPILRDGLLTAEGEVWKRSRKAMAPVFTPRHAQGFAGQMLRVCEAFVDRYAGASSEPFVT
NVAVDMTELTFEILAETLFSGEIAVEKQGFAANVEELLHRMGRVDPMDLLVAPSWVPRLTRIGGRKVLDRFRG
VVSETMSLRRRRTTEAPGDVPNDFLTLLLQLEGPDGLSTSEIEDNILTFIGAGHETTARALAWCFYCVANTPA
YRETMEQEIDSVLASGADPVDWLGRMPHVLAAFEEALRLYPPAPSINRAAIEEDAWTSPEGERVPIRKGISVL
VMPWTLHRHALYWQKPRAFMPERFLPENRDKINRFQYLPFGAGPRVCIGATFALQEAVIALAVLMHRFRFDLT
DETHPWPVQRLTTQPRGGLPMKVSARVK

>CYP173A2(C770_GR4Chr0060)*Sinorhizobium meliloti* GR4

MDTRPEPFEPPAPVPRTGIPSRLEIIRTVLRNPLELWGEPSYTLPWIETKFINQRTLIVNDPGLIRYILVENA
ANYEMSNVRRLILRPILRDGLLTAEGEVWKRSRKAMAPVFTPRHAQGFAGQMLRVCEAFVDRYAGASSEPFVT
NVAVDMTELTFEILAETLFSGEIAVEKQGFAANVEELLHRMGRVDPMDLLVAPSWVPRLTRIGGRKVLDRFRG
VVSETMSLRRRRTTEAPGDVPNDFLTLLLQLEGPDGLSTSEIEDNILTFIGAGHETTARALAWCFYCVANTPA
YRETMEQEIDSVLASGADPVDWLGRMPHVLAAFEEALRLYPPAPSINRAAIEEDAWTSPEGERVPIRKGISVL
VMPWTLHRHALYWQKPRAFMPERFLPENRDKINRFQYLPFGAGPRVCIGATFALQEAVIALAVLMHRFRFDLT
DETHPWPVQRLTTQPRGGLPMKVSARVK

>CYP173A2(SM2011_c02579)*Sinorhizobium meliloti* 2011

MDTRPEPFEPPAPVPRTGIPSRLEIIRTVLRNPLELWGEPSYTLPWIETKFINQRTLIVNDPGLIRYILVENA
ANYEMSNVRRLILRPILRDGLLTAEGEVWKRSRKAMAPVFTPRHAQGFAGQMLRVCEAFVDRYAGASSEPFVT
NVAVDMTELTFEILAETLFSGEIAVEKQGFAANVEELLHRMGRVDPMDLLVAPSWVPRLTRIGGRKVLDRFRG
VVSETMSLRRRRTTEAPGDVPNDFLTLLLQLEGPDGLSTSEIEDNILTFIGAGHETTARALAWCFYCVANTPA
YRETMEQEIDSVLASGADPVDWLGRMPHVLAAFEEALRLYPPAPSINRAAIEEDAWTSPEGERVPIRKGISVL
VMPWTLHRHALYWQKPRAFMPERFLPENRDKINRFQYLPFGAGPRVCIGATFALQEAVIALAVLMHRFRFDLT
DETHPWPVQRLTTQPRGGLPMKVSARVK

>CYP173A2(DU99_00295)*Sinorhizobium meliloti* RMO17

MDTRPEPFEPPAPVPRTGIPSRLEIIRTVLRNPLELWGEPSYTLPWIETKFINQRTLIVNDPGLIRYILVENA
ANYEMSNVRRLILRPILRDGLLTAEGEVWKRSRKAMAPVFTPRHAQGFAGQMLRVCAAFVDRYAGASSEPFVT
NVAVDMTELTFEILAETLFSGEIAVEKQGFAANVEELLHRMGRVDPMDLLVAPSWVPRLTRIGGRKVLDRFRG
VVSETMSLRRRRTTEAPGDVPNDFLTLLLQLEGPDGLSTSEIEDNILTFIGAGHETTARALAWCFYCVANTPA
YRETMEQEIDSVLASGADPVDWLGRMPHVLAAFEEALRLYPPAPSINRAAIEEDAWTSPEGERVPIRKGISVL
VMPWTLHRHALYWQKPRAFMPERFLPENRDKINRFQYLPFGAGPRVCIGATFALQEAVIALAVLMHRFRFDLT
DETHPWPVQRLTTQPRGGLPMKVSARVK

>CYP173A2(BN406_03194)*Sinorhizobium meliloti* Rm41

MDTRPEPFEPPAPVPRTGIPSRLEIIRTVLRNPLELWGEPSYTLPWIETKFINQRTLIVNDPGLIRYILVENA
ANYEMSNVRRLILRPILRDGLLTAEGEVWKRSRKAMAPVFTPRHAQGFAGQMLRVCEAFVDRYAGASSEPFVT
NVAVDMTELTFEILAETLFSGEIAVEKQGFAANVEELLHRMGRVDPMDLLVAPSWVPRLTRIGGRKVLDRFRG
VVSETMSLRRRRMTEAPGDVPNDFLTLLLQLEGPDGLSTSEIEDNILTFIGAGHETTARALAWCFYCIANTPA
YRETMEQEIDSVLASGADPVDWLGRMPHVLAAFEEALRLYPPAPSINRAAIEEDAWTSPEGERVPIRKGISVL
VMPWTLHRHALYWQKPRAFMPERFLPENRDKINRFQYLPFGAGPRVCIGATFALQEAVIALAVLMHRFRFDLT
DETHPWPVQRLTTQPRGGLPMKVSARVK

>CYP173A4(A6B35_06230)*Mesorhizobium amorphae*

MDTQPAPFVPPPAPKPRTTPPSTLEMMRIVYRNPLELWGEHTYNEPWISVTGVGGPLVVANDPGLIRHVLVDNA
RNYKMATVRQMILRPILRDGLLTAEGEVWKRSRKAMAPVFTPRHIFGFAQPMLKRTLDFVSRYEAGGTSDIAH
DMTLLTYDILAETLFSGEISGEPGSFAREIDRLFETMGRVDPLDLLRAPEWLPRLTRIRGRKTMAYFRKIVTD
TVRMREERLKRDPDAVPQDFLTLLLKAEEPDGLTRAEVEDNIITFIGAGHETTARALGWTIYCLAEAPWEREK
VEREIDTVLAREPDPTKWLDAMPLTRAAFEEALRLYPPAPSINREPIEPETWNELYIPRRAAVLVMPWVVHRH
RKLWDRPDAFMPERFHPGNREKIDRFQYLPFGAGPRICIGASFAMQEAIIALAIMLSRFRFDTTAETKPWPVQ
KLTTQPQGGLPMQITPRVPIFK

>CYP173A5(AA2016_0104)*Aminobacter aminovorans*

MDTRPEPFVPPAPTPRTTPPSTLEMMRIVYRNPLELWGEPSYNEPWISVNGVGGPLVIANDPGLIRHVLVDNA
RNYKMATVRQMILRPILRDGLLTAEGDVWKRSRKAMAPVFTPRHIFGFAQPMLARTLDFVKRYEDASGTVDVA
HDMTMLTYEILAETLFSGEIAGEAGSFAHEIDRLFETMGRVDPLDLLRAPEWLPRLTRIRGRKTMAYFRKIVT
DTVAMRSDKLVRDAAGAPEDFLTLLLRAEGPDGLSRQEIEDNIITFIGAGHETTARALGWTIYCLAEATWERD
RIEQEIDAVLAREPDPVRWLDAMPLTRAAFEEALRLYPPAPSINREPIAEDEYKGFKIPRKAQVLVMPWTVHR
HRKLWDKPDAFLPERFHPENRDRIDRYQYLPFGAGPRVCIGMSFAMQEAIIALAILLSRFRFDTTDETRPWPV
QKLTTQPQGGLPMRVTRR

>CYP173A6(BSQ44_05960)*Mesorhizobium* sp. B7

MDATPAPFVPPAPKPRTTPPSLLQMLRIVYRNPLELWGEPSYNEKWVSISTGVGGPLVIANDPGLIRHVLVDN
AKNYKMARVRQKILRPILRDGLLTAEGEVWKRSRKAMAPVFTPRHINGFAAPMLRRSEAFAERYEGETGTVDI
ARDMTLLTYDILAETLFSGEIAGEPGSFAKQVDRLFETMGRVDPLDLLGAPDWLPRFTRIRGARAMAFFRRLV
ADTARMRKEKLECDPEGAPNDFLTLLLKAEGPQGLSRDEIADNIITFIGAGHETTARALGWTLYCLAEAPWER
ERVEAEIDAVLAREADPAKWLDAMPVTRAAFEEAMRLYPPAASISREALADEFYEDLHIRKGAQVQVMPWTVH
RHRKLWDNPEAFMPERFLPHNRNRIDRFQYLPFGAGPRICIGASFAMQEAMIALGVLLSRFRFDTVAETKPWP
VQKLTTQPEGGLPMRVTRRR

>CYP173A7(Meso_4009)*Chelativorans* sp. BNC1

MATPTDLVAASFRPPAPSPRQTPPSTLQMMRIVYRNPLELWGEPSYNEPWVQLTGGIGGPLLIANDPGLIRHV
LVDNARNYKMARVRQKILRPILRDGLLTAEGEVWRRSRKAMAPVFTPRHISGFAGPMLQKAEAFAERYEQAIG
GTVDVSRDMTMLTFDILSETLFSGEVAGDPGGFAHQVDRLFETMGRVDPLDLLGAPDWLPRITRILGRNSLAF
FRNIVARTMEMRKEKLAGGDAEVPEDFLTLLLRAEGPQGLSRGEIEDNIITFIGAGHETTARALGWTLYCLAN
LPEERERIEAEIDSVLSSEPDPVKWLDAMPLTRAAFEEAMRLYPPAPSINREAIENDSYSDLKIAAGTQVLVM
PWTVHRHRKLWDDPDAYRPSRFHPENREKINRFQYLPFGAGPRVCIGATFAMQEAVIALAVLMSRYRFDLLPE
TKPWPVQKLTTQPQGGLPMGVSRR

>CYP173A8(Oant_0140)*Ochrobactrum anthropic* ATCC 49188

MEIANVMSERPVPFVPPAPRPREKPVGRFEMMRVVYKNPLELWGEPSYNERWISVSWFGMKTIIANDPGLIRH
VLVDNAANYPMSAIRQRVLRPLLRDGLLTAEGQVWKRSRKAMAPVFTPRHIGGFAEAMRDRSLQFVERYKTPG
TITDVAHDMTLLTYDILAETLFSGEIAGDPNDFAHQIDKLFETMGRVDPFDLLGLPDWLPRLTRLRGQQSLGF
FRELVSNTIELRKTRMDRQEDVPSDFLTLLLRAEGPDGLSRSEIEDNIITFIGAGHETTARALGWTLYLLAKA
PQERELIELEIDGFFAKGGRDLPPQEWLASLPFARAAFEEAMRLYPPAPSINREAATDDTYDDLKIPKGATVL
VMPWVIHRHRLYWDQPDAFMPERFWPENRDKLDRFQYLPFGAGPRVCIGASFALQEAVIALAILLDGRRFEVL
ETTKPWPVQKLTTQPQGGLPMKVMKR

>CYP173A8(DR92_107)*Ochrobactrum anthropic* OAB

MSERPVPFVPPAPRPREKPVGRFEMMRVVYKNPLELWGEPSYNERWISVSWFGMKTIIANDPGLIRHVLVDNA
ANYPMSAIRQRVLRPLLRDGLLTAEGQVWKRSRKAMAPVFTPRHIGGFAEAMRDRSLQFVERYKTPGTITDVA
HDMTLLTYDILAETLFSGEIAGDPNDFAHQIDKLFETMGRVDPFDLLGLPDWLPRLTRLRGQQSLGFFRELVS
NTIELRKTRMDRQEDVPSDFLTLLLRAEGPDGLSRSEIEDNIITFIGAGHETTARALGWTLYLLAKAPQEREL
IELEIDGFFAKGGRDLPPQEWLASLPFARAAFEEAMRLYPPAPSINREAATDDTYDDLKIPKGATVLVMPWVI
HRHRLYWDQPDAFMPERFWPENRDKLDRFQYLPFGAGPRVCIGASFALQEAVIALAILLDGRRFEVLETTKPW
PVQKLTTQPQGGLPMKVMKR

>CYP173A9(A8A54_00395)*Ochrobactrum pseudogrignonense*

METTIAMKDAAQPFVPPAPRPREKPVGRFEMMRVVYKNPLELWGEPSYNERWVSVSWLGIKTIIANDPGLIRH
VLVDNAANYPMSAIRQRVLRPLLRDGLLTAEGQVWKRSRKAMAPVFTPRHIGGFAEAMRDRSRQFVERYKTSG
IITDVAHDMTLLTYDILAETLFSGEIAGDPNDFARQIDKLFETMGRVDPFDLLGLPDWLPRFTRLRGNQSLGF
FRELVSDTIALRKARMDKGEDVPNDFLTLLLRAEGPDGLSRAEIEDNIITFIGAGHETTARALGWTLYLLAKA
PQERERVEAEIDAFFADGGNDLPPQEWLAKLPFTRAAFEEAMRLYPPAPSINREAATDDTYGDLKIAKGTAVL
VMPWVIHRHRLYWDQPDAFMPERFWPENRDKLDRFQYLPFGAGPRVCIGASFALQEAVIALATLLDGRRFDVL
ETTKPWPVQKLTTQPQGGLPMKVVRR

>CYP173A10(Smed_3268)*Sinorhizobium medicae*

MDTRPEPFEPPAPVPRTGIPSRLEIIRTVLRNPLELWGEPSYTLPWIETKFINQRTLIVNDPGLIRYILVENA
ANYEMSNVRRLILRPILRDGLLTAEGEVWRRSRKAMAPVFTPRHAKGFAGQMLRVSEEFVGRYARAASEPFVT
NAAVDMTELTFEILAETLFSGEIAVEKHGFAANVEELLHRMGRVDPMDLLVAPGWIPRLTRIGGRKVLDRFRG
VVSETMALRRRRMSETPGNVPNDFLTLLLQLEGPDGLSTSEIEDNILTFIGAGHETTARALAWCLYCIANTPT
YRETMEKEIDKVIAGGADPVDWPGRMPNVLAAFEEALRLYPPAPSINRAAIEEDEWISPQGERVPIRKGISVL

IMPWTLHRHVAYWQKPRAFMPERFLPENRDKIHRFQYLPFGAGPRICIGATFALQEAVIALAVLMHRFRFDVT
DETHPWPVQRLTTQPRGGLPMRVSARVR

>CYP173A11(NGR_c34090)*Sinorhizobium fredii* NGR234

MDTRPEPFEPPAPVPRTHIPSRIEIIRTVLRNPLELWGEPSYTLPWIETKFINQRTLIVNDPGLIRYILVENA
SNYEMSNVRRLILRPILRDGLLTAEGDVWKRSRKAMAPVFTPRHAKGFAGQMLRVSEHFRERYERAASEPFVS
NVAVDMTELTFEILAETLFSGEIAVEKEGFAANVEELLHRMGRVDPMDLLVAPPWVPRLTRIGGRKVLDRFRG
VVSATMAERRRRMADEPDKVPNDFLTLLLQLERPEGLSTSEIEDNILTFIGAGHETTARALAWTLYCIANTSA
YRETMEKEIDSALASGAEPVEWLERMPHVLAAFEEALRLYPPAPSINRAAIEEDAWTSPEGEVVPIRKGISVL
IMPWTLHRHALYWQKPRAFMPERFLPENRDKINRFQYLPFGAGPRVCIGATFALQEAVIALAVLMHRFRFDLT
EATHPWPVQRLTTQPKGGLPMKVSVRAA

>CYP173A12(FA04_18030)*Ensifer adhaerens Casida* A

MDMRPEPFEPPAPVPRVGIPSYLKIIRTVLRNPLELWGEPSYTLPWIETKFVTQRTLIVNDPGLIRHILVDNV
ANYEMSKVRRLILRPILRDGLLTAEGEVWKRSRKAMAPVFTPRHAKGFAGQMLRSSEQFVERYAGAQEAPFTT
NVANDMTELTFDILAETLFSGEVAVEREGFVQSVEELLHRMGRVDPMDILLAPEWIPRPTRIGGRKVMNRFRS
IVSETMALRRRKMAEDPKGTPQDFLTLLLEIEGSQGLSTAEIEDNILTFIGAGHETTARALAWTLYCVANTPA
YREIMEAEIDTAMASGAEPVEWLGLMPHVLAAFEEAMRLYPPAPSINRAAIEEDAWTSPTGERVRIPKGIAVL
IMPWTLHRHVLYWDKPRAFMPERFLPENREKLNRYQYLPFGAGPRVCIGATFALQEAVIALAVMMHRYRFDTT
AETNPWPVQRLTTQPRGGLPMRVSAR

>CYP173A13(OV14_0942)*Ensifer adhaerens* OV14

MDMRPEPFEPPAPIPRVGVPSYLKIIKTVLRNPLELWGEPSYTLPWIETKFITQRTLIVNDPGLIRYILVENV
SNYEMSKVRRLILRPILRDGLLTAEGEIWKRSRKAIAPVFTPRHAKGFAGQMLRRSEEFVERYAHAKVKPFTT
NVASDMTELTFDILAETLFSGEVAVEREGFVQSVEELLHRMGRVDPMDILLAPEWLPRLTRIGGKKVMNRFRS
IVSETMAMRRQRMATDPAGTPNDFLTLLLQTEGANGLSTQEIEDNILTFIGAGHETTARALAWTLYCVANTPA
FRETMEAEIDTVLKSGAEPVEWLNLMPHVLAAFEEAMRLYPPAPSINRAAIEEDEWTSPSGERVRIPKGIAVL
VMPWTLHRHVLYWDRPRAFMPERFLPENREKLHRFQYLPFGAGPRICIGATFALQEAVIALAVLMHRFRFDVT
DETRPWPVQRLTTQPRGGLPMRVSAR

>CYP173A14(RG1141_CH40350)*Neorhizobium galegae* bv. officinalis bv. officinalis HAMBI 1141

MDIRPEPFVPPAPPHRDRLLSPIEVIWTALRNPLELWGKVSYTLPWLETKFFKERTLIVNHPGLIRHILVDNV
SNYVMSEIRQLVLRPILRDGLLTAEGEVWKRSRKAMAPVFTPKHSRGFAGQMLNQTEEFSGKYAEAGADGAVR
DISVDMTELTYAILAETLFSGEVAGNKETVADDVDQLLHRMGRIDPMDMLRAPPWVPRVTRIGGRRILDKFRR
VVAETMLMRQERMRKHPDDVPQDFLTLLLQLAGPDGLTMDEIEDNILTFIGAGHETTARALAWTLYCVAESPS
VRQNMEEEIDRVLASGADPVEWLELMPWVRAAFEEALRLYPPAPSINREAISDDSWTSPEGKTIRIDAGVTVL
VMPWTLHRHELYWDKPRAFMPERFLPENRGKLNRFQYLPFGAGPRTCIGATFALQEAVIALAVLMGRYRFDVT
PETKVWPVQKLTTQPRDGLLMRVSPRHQRSPLVN

>CYP173A14(RG540_CH40900)*Neorhizobium galegae* bv. orientalis HAMBI 540

MDIRPEPFVPPAPPHRERLLGPIEVIWTALRNPLELWGKVSYTLPWLETKFFKERTLIVNHPGLIRHILVDNV
SNYVMSEIRQLVLRPILRDGLLTAEGEVWKRSRKAMAPVFTPKHSRGFAGQMLSQTEEFSRKYAAAGAGGAVR
DISVDMTELTYAILAETLFSGEVAGNKETVADDVDQLLHRMGRIDPMDMLRAPPWVPRVTRIGGRRILDKFRR
VVAETMLMRQERMRKHPDDVPQDFLTLLLQLAGPDGLTMDEIEDNILTFIGAGHETTARALAWTLYCVAESPS
VRQAMEEEIDRVLASGADPVEWLELMPWVRAAFEEALRLYPPAPSINREAISDDSWTSPEGKTIRIDAGVTVL
VMPWTLHRHELYWEKPRAFMPERFLPENRGKLNRFQYLPFGAGPAPVSARPLPCRKR

>CYP173A15(LPU83_0051)*Rhizobium* sp. LPU83

MDMRPHPFEPPAPLPRTVPPGRLEIIRTILRNPLELWGEPSHTLPWIHTRFFRERTLIVNDPGLIRHVLVDNA
ANYRMSDIRQLILRPILRDGLLTAEGEVWKRSRKAVAPVFTPRHANGFAGQMLRQSEDYARKYQAAGSEGKVF
EIAADMTELTFAILADTLFSGEIVTSSDSFADDVNALLHRMGRVDPMDLLRAPSWVPRLTRIGGQKVLDKFRN

IVRQTMNARLARMQANRAATPDDFLTLLLDQAASGGLTNDEIEDNILTFIGAGHETTARALAWTLYCVANTQH
IREAMEEEIDRVLARDVEPVKWLDLMPYTRAAFEEALRLYPPAPSINRAAIAADRWTDAKGETVEIEAGVTVL
IMPWTLHRHELYWDKPRAYLPERFLPENRGKIGRFQFLPFGAGPRVCIGATFALQEAVIALAVLMHRFRFDLT
DQTKPWPVQKLTTQPQNGLPMCVTPRSLPKSA

>CYP173A16 (RGR602_CH00052)*Rhizobium gallicum*

MDMRPEPFVPPAPLPRSVPPSPLEIIRTILRNPLELWGEPSYTLPWIKTRFFHDSTIIVNDPGLIKHVLVDNV
ANYRMADIRQLVLRPILRDGLLTAEGEVWKRSRKAVAPVFTPRHAKGFAGQMLRQSEVYARKYEAVAGESQVF
EIASDMTELTFAILADTLFSGEIVTSSDSFAEDVNALLHRMGRVDPMDLLRAPSWVPRVTRIGGQKVLDKFRT
IVRETMDARLRRMAADRGSAPEDFLTLLLDRAATGGLTNEEIEDNILTFIGAGHETTARALAWTLYCVANSPH
IRDAMEEEIGRVLASGAEPVEWLDLMPFTRAAFEEALRLYPPAPSIDRAAIADDSWTDSKGEKVDIHAGMTVL
VMPWTLHRHELYWEKPRSYMPERFFPENRGKIGRFQFLPFGAGPRVCIGATFAMQEAVIVLAVLMSRFRFDVT
EKTNPWPVQKLTTQPQNGLPMRVTERTVSLSA

>CYP173A17(RHECIAT_CH0000054)*Rhizobium etli* CIAT 652

MDMRPDPFVPPAPLPRTVPPSRLDIIRIILRNPLELWGEPSYTLPWIKTSFFGQHTLIVNDPGLIKHVLVDNA
NNYRMSDIRQLVLRPILRDGLLTAEGQVWKRSRKAVAPVFTPRHAQGFAGQMLRQSEDYARKYESAGAAGAIF
DISIDMTELTFAILADTLFSGEIVTSSGSFADDVNALLHRMGRIDPMDLMRAPSWVPRVTRIGGQKVLEKFRA
IVRDTMDMRLAKMKADRAAAPDDFLTLLLEQAGPDGLTKEEIEDNILTFIGAGHETTARALAWTLYCVSNSPH
IREAMETEIDAVLATGAEPVEWLDLMPQTRAAFEETLRLYPPAPSINRAAISDDSWTNPKGERIEIEAGVTVL
IMPWTLHRHEFYWEKPRAYMPERFLPENRGSIGRFQFLPFGAGPRVCIGATFALQEAVIALAVLMHRYRFDST
DETNPWPVQKLTTQPQNGLPMRVTPRTVSSAV

>CYP173A17(AMC79_CH00054)*Rhizobium phaseoli*

MDMRPDPFVPPAPLPRTVPPSRLDIIRIILRNPLELWGEPSYTLPWIKTSFFGQHTLIVNDPGLIKHVLVDNA
NNYRMSDIRQLVLRPILRDGLLTAEGQVWKRSRKAVAPVFTPRHAQGFAGQMLRQSEDYARKYERAEPAGAIF
DISIDMTELTFAILADTLFSGEIVTSSGSFADDVNALLHRMGRIDPMDLMRAPSWVPRVTRIGGQKVLEKFRA
IVRDTMDMRLAKMKADRAAAPDDFLTLLLEQAGPDGLTKEEIEDNILTFIGAGHETTARALAWTLYCVSNSPH
IREAMETEIDAVLATGAEPVEWLDLMPQTRAAFEETLRLYPPAPSINRAAISDDSWTNPKGERIEIEAGVTVL
IMPWTLHRHEFYWEKPRAYMPERFLPENRGSIGRFQFLPFGAGPRVCIGATFALQEAVIALAVLMHRYRFDST
DETNPWPVQKLTTQPQNGLPMRVTPRTVSSAA

>CYP173A17(Rleg2_3985)*Rhizobium leguminosarum* bv. trifolii WSM2304

MDMRPEPFVPPAPLPRTVPPSRLEIIRTILRNPLELWGEPSYTLPWIKTSFFGQHTLIVNDPGLIKHVLVDNA
GNYRMSDIRQLVLRPILRDGLLTAEGSVWKRSRKAVAPVFTPRHAQGFAGQMLRQSEDYAGKYQSEAGPIFDI
STDMTELTFAILADTLFSGEIVTSSGHFADDVNALLHRMGRVDPMDLMRAPSWVPRVTRIGGQKVLEKFRAIV
RDTMDMRTAKMKADRATAPEDFLTLLLEQAGPDGLTKEEIEDNILTFIGAGHETTARALAWTLYCVSNSPHIR
EAMETEIDAVLATGAEAVEWLDMMPQTRAAFEETLRLYPPAPSINRAAISDDSWTSPEGERVELEAGVTVLIM
PWTLHRHELYWDRPRAYMPERFLPENRGSIGRFQFLPFGAGPRVCIGATFALQEAVIALAVLMHRYRFDSTDE
TNPWPVQKLTTQPQNGLPMRVTPRIISR

>CYP173A18(IE4803_CH00051)*Rhizobium etli* bv. phaseoli IE4803

MDMRPDPFVPPAPLPRTVPPSRLEIIRIILRNPLELWGEPSYTLPWIKTSFFGQHTLIVNDPGLIKHVLVDNA
ANYRMSDIRQLVLRPILRDGLLTAEGQVWKRSRKAVAPVFTPRHAKGFAGQMLRQSEEYVGKYEGAGPAGQVF
DIAADMTDLTFAILAETLFSGEIVSSSGHFADDVNQLLHRMGRVDPMDLLRAPSWVPRLTRIGGQKVLEKFRA
IVRDTMDMRLAKMKADRAGAPDDFLTLLLEQAGPDGLTKDEIEDNILTFIGAGHETTARALAWTLYCVANSPH
IREAMETEIDAVLATGADSVEWLDLMPVTRAAFEEALRLYPPAPSINRAAIADDVWINAKGERVEIPADITVL
IMPWTLHRHELYWDKPRAYMPERFFPENRGSIGRFQFLPFGAGPRVCIGATFALQEAVIALAVMMHRFRFDQT
EATDPWPVQKLTTQPQNGLPMRVTPRIISQAA

>CYP173A18(IE4771_CH00052)*Rhizobium etli* bv. mimosa IE4771

MDMRPDPFVPPAPLPRTVPPSRLEIIRIILRNPLELWGEPSYTLPWIRTSFFGQHTLIVNDPGLIKHVLVDNA
ANYRMSDIRQLVLRPILRDGLLTAEGQVWKRSRKAVAPVFTPRHAKGFAGQMLRQSEEYVRKYEGAGPAGQVF
DIAADMTDLTFAILAETLFTGEIVSSSGHFADDVNELLHRMGRVDPMDLLRAPSWVPRLTRIGGQKVLEKFRA
IVRDTMDMRLAKMKADRAAAPDDFLTLLLEQAGPDGLTKEEIEDNILTFIGAGHETTARALAWTLYCVANSPH
IREAMETEIEAVLATGADPVEWLDLMPVTRAAFEEALRLYPPAPSINRAAIADDVWTSAKGERVEIPADITVL
IMPWTLHRHELYWDRPRAYMPERFFPENRGSIGRFQFLPFGAGPRVCIGATFALQEAVIALAVMMHRFRFDQT
EATNPWPVQKLTTQPQNGLPMRVTRRIISQAA

>CYP173A18(AMJ98_CH00052)*Rhizobium* sp. N1341

MDMRPDPFVPPAPLPRTVPPSRLDIIRIILRNPLELWGEPSYTLPWIKTSFFGQHTLIVNDPGLIKHVLVDNA
ANYRMSDIRQLVLRPILRDGLLTAEGQVWKRSRKAVAPVFTPRHAKGFAGQMLRQSEDFVRKYENVDAAGQVF
DIASDMTDLTFAILAETLFSGEIVSSSGHFADDVNQLLHRMGRIDPMDLLRAPAWVPRLTRIGGQKVLEKFRA
IVRDTMDMRLAKMKADRAAAPEDFLTLLLEQAGPDGLTKEEIEDNILTFIGAGHETTARALAWTLYCVANSPH
IREAMETEIDAVLATGAEPVEWLDLMPVTRAAFEEALRLYPPAPSINRAAIADDLWINAKGEKVEIPADITVL
IMPWTLHRHELYWEKPRAYMPERFLPGNRGSIGRFQFLPFGAGPRVCIGATFALQEAVIALAVMMHRFRFDQT
ATTNPWPVQKLTTQPQNGLPMRVTSRIISRTA

>CYP173A18(AMK02_CH00052)*Rhizobium* sp. N731

MDMRPDPFVPPAPLPRTVPPSRLDIIRIILRNPLELWGEPSYTLPWIKTSFFGQHTLIVNDPGLIKHVLVDNA
ANYRMSDIRQLVLRPILRDGLLTAEGQVWKRSRKAVAPVFTPRHAKGFAGQMLRQSEDFVRKYENVDAAGQVF
DIASDMTDLTFAILAETLFSGEIVSSSGHFADDVNQLLHRMGRIDPMELLRAPAWVPRLTRIGGQKVLEKFRA
IVRDTMDMRLAKMKADRAAAPEDFLTLLLEQAGPDGLTKEEIEDNILTFIGAGHETTARALAWTLYCVANSPH
IREAMETEIDAVLATGAEPVEWLDLMPVTRAAFEEALRLYPPAPSINRAAIADDLWINAKGEKVEIPADITVL
IMPWTLHRHELYWEKPRAYMPERFLPGNRGSIGRFQFLPFGAGPRVCIGATFALQEAVIALAVMMHRFRFDQT
ATTNPWPVQKLTTQPQNGLPMRVTSRIISRTA

>CYP173A18(REMIM1_CH00052)*Rhizobium etli*

MDMRPDPFVPPAPLPRTVPPSRLDIIRIILRNPLELWGEPSYTLPWIKTSFFGQHTLIVNDPGLIKHVLVDNA
ANYRMSDIRQLVLRPILRDGLLTAEGQVWKRSRKAVAPVFTPRHAKGFAGQMLRQSEDFVRKYENGDAAGQIF
DIATDMTDLTFAILAETLFSGEIVSSSGHFADDVNQLLHRMGRIDPMDLLRAPSWVPRLTRIGGQKVLETFRA
IVRDTMDMRLAKMKADRAAAPEDFLTLLLEQAGPDGLTEEEIEDNILTFIGAGHETTARALAWTLYCVANSPH
IREAMETEIDAVLTTGAEPVEWLDLMPVTRAAFEEALRLYPPAPSINRAAIADDLWINAKGEKIEIPADITVL
IMPWTLHRHELYWEKPRAYMPERFLPENRGAIGRFQFLPFGAGPRVCIGATFALQEAVIALAVMMHRFRFDQT
ATTNPWPVQKLTTQPQNGLPMRVTSRIFSRTA

>CYP173A18(RLEG12_31535)*Rhizobium leguminosarum* bv. trifolii CB782

MDMRPDPFVPPAPLPRTVPPSRLDIIRIILRNPLELWGEPSYTLPWIKTSFFGQHTLIVNDPGLIKHVLVDNA
NNYRMSDIRQLVLRPILRDGLLTAEGQVWKRSRKAVAPVFTPRHAKGFAGQMLRQSEEYIGKYEGSGSAGQVF
DIASDMTDLTFAILAETLFSGEIVSSSGHFSDDVNQLLHRMGRVDPMDLLRAPSWVPRLTRIGGQKVLEKFRA
IVRDTMDLRLAKMKADRAAAPDDFLTLLLEQAGPDGLTKEEIEDNILTFIGAGHETTARALAWTLYCVANSPH
IREVMETEIDAVLATGAEPVEWLDLMPVTRAAFEEALRLYPPAPSINRAAIADDVWTNAKGEEVEIPADITVL
IMPWTLHRHELYWDRPRAYMPERFLPENRGSIGRFQFLPFGAGPRVCIGATFALQEAVIALAVMMHRYRFDST
DETNPWPVQKLTTQPQNGLPMRVTPRIISTKA

>CYP173A19(Arad_0067)*Agrobacterium radiobacter*

MDMIPEAFSPPAPIPRTVPPSRLTIIRTILRNPLELWGEPSYTLPWIMTRFIRERTLIVNDPGLIKHVLVDNA
ANYRMSDIRQLILRPILRDGLLTAEGPVWKRSRKAVAPVFTPRHAQGFAGQMLSQSEDYVRKYEDVGSEGEVF
DIAVDMTELTFAILAETLFSGEIVTEGASFADDVNQLLHRMGRVDPMDLLRAPSWVPRVTRIGGQKVLDKFRG
IVRDTMNLRLDKMRKDRASAPDDFLTLLLEKTGPDGLTMEEIEDNILTFIGAGHETTARALAWTLYCVANCPH
IREAMETEIDRVLATGVEPVAWLDLMPNVRASFEEAMRLYPPAPSINRASIADDEWTSPSGERVEIPAGVTVL
IMPWTLHRHALYWDKPRAFMPERFLPENRGKINRFQYLPFGAGPRVCIGATFALQEAVIALAVMMHRFRFDLT
DETNPWPVQKLTTQPQGGLPMRVTRR

>CYP173A19(RL0053)*Rhizobium leguminosarum* bv. viciae 3841

MDMRPDPFVPPAPLPRTVPPSRLEIIRIILRNPLELWGEPSYTLPWIRTNFFGQRTLIVNDPGLIKHVLVDNA
NNYRMSDVRQLVLRPILRDGLLTAEGPVWKRSRKAVAPIFTPRHAQGFAGQMLRQSEDYARKYEGAGEAGAIF
DISTDMTELTFAILADTLFSGEIVTSSGHFADDVNELLHRMGRVDPMDLMRAPSWVPRVTRIGGQKVLEKFRA
IVRNTMDMRLAKMKADRSSAPEDFLTLLLEQAGPDGLTKEEIEDNILTFIGAGHETTARALAWTLYCVSNSPH
IREGMEEEIDAVLATGAKPVEWLDMMPQTRAAFEEALRLYPPAPSINRAAISDDFWTSPKGERVELEAGVTVL
VMPWTLHRHELHWDRPRAYMPERFLPENRASIGRFQFLPFGAGPRVCIGATFALQEAVIALAVLMHRYRFDST
DQTNPWPVQKLTTQPKNGLPMRVTPRIISTKA

>CYP173A19(Rleg_4313)*Rhizobium leguminosarum*_bv. trifolii WSM1325

MDMRPDPFVPPAPLPRTVPPSRLEIIRIILRNPLELWGEPSYTLPWIRTNFFGQRTLIVNDPGLIKHVLVDNA
NNYRMSDVRQLVLRPILRDGLLTAEGPVWKRSRKAVAPIFTPRHAQGFAGQMLRQSEDYARKYEGAGEAGAIF
DISTDMTELTFAILADTLFSGEIVTSSGHFADDVNELLHRMGRVDPMDLMRAPSCVPRVTRIGGQKVLEKFRA
IVRNTMDMRLAKMKADRSSAPEDFLTLLLEQAGPDGLTKEEIEDNILTFIGAGHETTARALAWTLYCVSNSPH
IREGMEEEIDAVLATGAKPVEWLDMMPQTRAAFEETLRLYPPAPSINRAAISDDSWTSPKGERVELEAGVTVL
VMPWTLHRHELHWDRPRAYMPERFLPENRGSIGRFQFLPFGAGPRVCIGATFALQEAVIALAVLMHRYRFDST
DQTNPWPVQKLTTQPQNGLPMRVTPRIISTKA

>CYP173A19(RLEG3_32540)*Rhizobium leguminosarum* bv. trifolii WSM1689

MDMRPDPFVPPAPLPRTVPPSRLEIVRIILRNPLELWGEPSYTLPWIRTNFFGQRTLIVNDPGLIKHVLVDNA
NNYRMSDVRQLVLRPILRDGLLTAEGPVWKRSRKAVAPIFTPRHAQGFAGQMLRQSEDYARKYEGAGEAGAIF
DISTDMTELTFAILADTLFSGEIVTSSGHFADDVNELLHRMGRVDPMDLMRAPSWVPRVTRIGGQKVLEKFRA
IVRNTMDMRLAKMKANRSTAPEDFLTLLLEQAGPDGLTNEEIEDNILTFIGAGHETTARALAWTLYCVSNSPH
IREAMEEEIDAALATGAEPVEWLDMMPQTRAAFEEALRLYPPAPSINRAAISDDSWSSTKGERVVIEAGVTVL
IMPWTLHRHELHWDRPRAYMPERFLPENRGSIGRFQFLPFGAGPRVCIGATFALQEAVIALAVLMHRYRFDST
DQTNPWPVQKLTTQPQNGLPMRVTPRIISTKA

>CYP173A20(RTCIAT899_CH00270)*Rhizobium tropici*

MDMIPEAFSPPAPIPRTVPPSRLTIIRTILRNPLELWGEPSYTLPWIKTKFFNERTLIVNDPGLIKHVLVDNA
ANYRMADIRQLVLRPILRDGLLTAEGPVWKRSRKAVAPVFTPRHAQGFAGQMLSQSLEYLQKYEDIGAEGAVF
DIATDMTELTFAILAETLFSGEILTEGEHFADEVNELLHRMGRVDPMDLLRAPAWVPRITRIGGQRVLDKFRG
IVRTTMDQRLDKMRRDRASAPDDFLTLLLEKAGPDGLTMEEIEDNILTFIGAGHETTARALAWTLYCVANSPH
IRDAMEEEIDRVLATDAKPVAWLDLMPHVRAAFEEAMRLYPPAPSINRAAIADDEWTSAKGERVTIEKGITVL
IMPWTLHRHELYWEKPRAFMPERFLPENRAKINRFQYLPFGAGPRVCIGATFALQEAVIALAVMMRRFRFDTT
DETDPWPVQKLTTQPRGGLPMRVTRR

>CYP173A21(shn_20235)*Shinella* sp. HZN7

MDVRPEPFEPPAPIPRSVPPSRLEIIRTVFRNPLELWGEPSYTLPWIMTRFFGEKTLIVNDPGLIKYLLVDNA
QNYKMAVVRQLVLRPILRDGLLTAEGAVWRRSRKAVAPIFTPRHAKGFASQMLAQSELYAKKYEGSDGTVFDI
GNDMTELAFAVLSETLFSGEIVTRSESFADDVEDLLHSMGRVDPMDLLRAPSWVPRVTRIGGLKVLKKFRGIV
RDTMDLRLRRMKDSDTAPNDFLTLLLRAAGPEGLTMEEVEDNILTFIGAGHETTARALAWTLYCVANSPHVRD
AMEEEIDRVLESGADPVDWLDRMPWTRAAFEEAMRLYPPAPSINREAIADDAWISPKGERVEIPKDVTILVMP
WTLHRHALLWQKPRAYMPERFLPENREAIHRFQYLPFGAGPRICIGATFAMQEAIIALGVLMRRFRFDMTEET
RPWPVQRLTTQPANGLALRVTARRPASSIS

>CYP173A22(NT26_3879)*Rhizobium* sp. NT-26

MDMRPEPFVPPAPIPRTVPPSRLEIIRTVFNNPLELWGEPSYTLPWIETKFLKERTIIVNDPGLIRHVLVDNA
ANYEMSEIRQLILRPILRDGLLTAEGPVWKRSRKAMAPVFTPRHARSFADQMLYKSEDYVEKYSGVGEEGEVF
DISVDMTEITFNILSETLFSGEIVTESNDFSEDVDSLLHRMGRVDPMDLLRAPPWVPRLTRIGGKRVLGKFRE
IVAKTMALRQKKMRKNPGHVPQDFLTLLLELAGPDGLTMDEIEDNILTFIGAGHETTARALAWTLYCVANSPQ
IRKRMESEIDEVLASGAEPVEWMDRMPMVRAAFEEALRLYPPAPSLNRAAIADDEWTSPDGKTVRIRAGVTVL

IMPWTLHRHQLYWDNPRAFMPERFLPENRGKIGRFQYLPFGAGPRVCIGATFAMQEAVIALAVLMSRYRFDMT
PGTKPWPVQKLTTQPRDGLPMRVTRRVRQ

>CYP173A23(BSY16_3110)*Sinorhizobium* sp. RAC02

MDMRPEPFEPPAPIPRTVPPSKLEIIRTVFRNPLELWGEPSYTLPWIMTSFFGEKTLIVNDPGLIKYLLVDNA
ANYKMAVVRQLVLRPILRDGLLTAEGAVWRRSRKAVAPIFTPRHAKGFASQMLAQSELYAQKYEGADGAAFDI
GNDMTELAFAILSETLFSGEIVTSSNNFADDVDDLLHSMGRVDPMDLLRAPAWVPRVTRIGGLRVLGKFRKIV
RDTMDLRLKRMNSAAGDAPNDFLTLLLRAAGPEGLTMEEVEDNILTFIGAGHETTARALAWTLYCVANSPHVR
EAMEDEIDRVLASGADPVEWLELMPWTRAAFEEAMRLYPPAPSINREAIADDVWTSPSGERVEIPKDVTILVM
PWTLHRHALLWQKPRAYMPERFLPENRDAIHRFQYLPFGAGPRICIGATFAMQEAIIALGVLMHRFRFDMTET
TRPWPVQRLTTQPANGLALRVTARRPASSIS

>CYP173A24(BSY240_2823)*Agrobacterium* sp. RAC06

MDASLRKSTFEPPAPVPRTVPPSRLEIIRTVLRNPLELWGQPSYTWPWIVTRFFGQTTIIANDPGLIRHVLVD
NASNYEMSEIRQLVLRPILRDGLLTAEGPVWKRSRKAMAPVFTPRHARGFARQMREQSELFLERYEAGTEGVV
RDIAVDMTELTYAILSETLFSGGIVSEQGDFAKDVDDLLHSMGRIDPLDLLKAPRWIPRLTRIRGLGVLRKFR
ELVRRTMDHRRAIIKTDRENAPDDFLTLLLELEGPDGLTSEEIEDNILTFIGAGHETTARALAWTLYCVAHCP
DVRDAMEREIDEVIASGADPVDWLEQMPHVRASFEEAMRLYPPAPSINRDAIADDRYTAPSGETVEIAKGATV
LVMPWTLHRHELYWDKPRIFDPSRFLPGEREKIGRFQYLPFGAGPRVCIGATFALQEAVIALAVLMQRYRFAT
TPQTKPWPVQKLTTQPAGGLPMRVTPRKP

>CYP173A25(IMCC20628_04172)*Hoeflea* sp. IMCC20628

MTKPHTPFVPPAPVPRTTPPSRFQIIRTVMRNPLELWGEPSFRLEWMKTKFFNERTLIVNHPGLVRHVLVDNA
KNYKMAVIRQLILRPILRDGLLTAEGETWRRSRKAMAPVFTPRHAQGFAEQMLEQSRAFTGRYDIAAEKGEVR
DIAVDMTELTFDILSATLFSGQVAGTETEFAGDIERLLSTMGRVDPMDLLKAPSWVPRIRRLFGRRVLAKFRN
IVKQTMADRQHLIKTDPDAAPEDFLTLLIRLHGPDGLTLDEIEDNIITFIGAGHETTARALGWTLYCLANSPS
DREKVEAEIDEVLAREPDPVKWLDAMPWTRAAFEEAMRLYPPAPSINRAAIADDKWDNGKGETVTIEAGTTIL
VMPWTLHRHTKLWDQPGAYIPSRFLPENRGKIDRYQYLPFGVGPRICIGATFALQEAVIALGVLMSKYRFDCV
AETKPWPVQKLTTQPEGGLPMRVSRR

>CYP173B2(Mext_2663)*Methylobacterium extorquens* PA1

MLDTLPLAPPTTTPLFRPKVPPPLTQPLGLFAFLKKVRENPIATWMDAHFEEFVVAGETAMGRITVVSDPALV
RYLLVERAAHYRKDDLQKRVLAPGLGNGLLTAEGDEWRLQRRTLAPIFSARHVAGFVAQMDAAGARLGRRLAR
RDGATVDVALEMTRATLDVLERTIFTQGLPGDPDALGRAITRLLESIGPIDPLDVFGFPAFVPRLGRLRARPA
LRFFAEVVDTLLDERKAALARGEAPHDLMTLLLAAQDPETGRGLSDLEVKANIVTFIAAGHETTANALTWALY
CLSQDEAARTRVEAEVDAAAGPDGALQLDRLPFTKAVMEETMRLFPPVPFLSRQALREDRIGRVKIPRNSTVI
IAPWVMHRHRKLWDEPDAFIPERFLGERRESVERFAYLPFGAGPRVCIGQSFSVQEATLVLAHVARAVRLTLP
ADHPPVTPLHRVTLRPKDGLRMLTQRRA

>CYP173B2(Mex_1p2856)*Methylobacterium extorquens* AM1

MLDTLPLAPPTTTPLFRPKVPPPLTQPLGLFAFLKKVRENPIATWMDAHFEEFVVAGETAMGRITVVSDPALV
RYLLVERAAHYRKDDLQKRVLAPGLGDGLLTAEGDEWRLQRRTLAPIFSARHVAGFVAQMDAAGARLGRRLAR
RDGATVDVALEMTRATLDVLERTIFTQGLPGDPDALGRAITRLLESIGPIDPLDVFGFPAFVPRLGRLRARPA
LRFFAEVVDTLLDERKAALARGEAPHDLMTLLLAAQDPETGRGLSDLEVKANIVTFIAAGHETTANALTWALY
CLSQDEAARARMEAEVDAAAGEGGALQLDRLPFTKAVMEETMRLFPPVPFLSRQALREDRIGRVKIPRNSTVI
IAPWVMHRHRKLWDEPDAFIPERFLGERRESVERFAYLPFGAGPRVCIGQSFSVQEATLVLAHVARAVRFTLP
ADHPPVTPLHRVTLRPKDGLRMLAQRRA

>CYP173B2(METDI3422)*Methylobacterium extorquens* DM4

MLDTLPLAPPTTTPLFRPKVPPPLTQPLGLFAFLKKVRENPIATWMDAHFEEFVVAGETAMGRITVVSDPALV
RYLLVERAAYYRKDDLQKRVLAPGLGDGLLTAEGDEWRLQRRTLAPIFSARHVAGFVAQMDAAGARLGRRLAR
RDGATVDVALEMTRATLDVLERTIFTQGLPGDPDALGRAITRLLESIGPIDPLDVFGFPAFVPRLGRLRARPA

LRFFAEVVDTLLDERKAALARGEAPHDLMTLLLAAQDPETGRGLTDLEVKANIVTFIAAGHETTANALTWALY
CLSQDEAARTRVEAEVDAAAGEGGALQLDRLPFTKAVMEETMRLFPPVPFLSRQALREDRIGRVKIPRNSTVI
IAPWVMHRHRKLWDEPDAFIPERFLGERRESVERFAYLPFGAGPRVCIGQSFSVQEATLVLAHVARAVRFTLP
ADHPPVTPLHRVTLRPKDGLRMLAQRRA

>CYP173B2(Mpop_2785)*Methylobacterium populi*

MLDTLPLAAPVTNAPLFRPKVPPPLTRPLGLFAFLKAVRANPIATWMDAHFEEFIVAGETAMGRVTVVSDPAV
VRYLLVERAANYRKDDLQKRVLAPGLGDGLLTAEGDEWRLQRRTLAPIFSARHVAGFVAQMDAAGARLGRRLA
RRDGATVDVALEMTRATLDVLERTIFTQGLPGDPDALGRAITRLLESIGPIDPLDVFGFPAFVPRLGRLRARP
ALRFFAEVVDTLLDQRKAALARGEAPHDLMTLLLAAQDPETGRGLSDLEVKANIVTFIAAGHETTANALTWAL
YCLSQDEAARDRVEAEVDAAAGPDGALRLDRLPFTKAVMEETMRLFPPVPFLSRQALREDRIGRVKIPRNSTV
IIAPWVMHRHRKLWDEPDAFMPERFFGAARDSIERFAYLPFGAGPRICIGQSFSVQEATLVLAHVARAVRLTL
PADHPPVTPLHRVTLRPKDGLRMLAQRRV

>CYP173B2(Mchl_2890)*Methylobacterium extorquens* CM4

MLDTLPLAPPTTTPLFRPKVPPPLTQPLGLFAFLKKVRENPIATWMDAHFEEFVVAGETAMGRITVVSDPALV
RYLLVERAAHYRKDDLQKRVLAPGLGDGLLTAEGDEWRLQRRTLAPIFSARHVAGFVAQMDAAGARLGRRLAR
RDGATVDVALEMTRATLDVLERTIFTQGLPGDPDALGRAITRLLESIGPIDPLDVFGFPAFVPRLGRLRARPA
LRFFAEVVDTLLDERKAALARGEAPHDLMTLLLAAQDPETGRGLSDLEVKANIVTFIAAGHETTANALTWALY
CLSQDEASRTRVGAEVDTAAGPDGALQLDRLPFTKAVMEETMRLFPPVPFLSRQALREDRIGRVKIPRNSTVI
IAPWVMHRHRKLWDEPDAFIPERFLGERRDRVERFAYLPFGAGPRVCIGQSFSVQEATLVLAHVARAVRFTLP
ADHPPVTPLHRVTLRPKDGLRMLAQRRA

>CYP173B2(Y590_12775)*Methylobacterium* sp. AMS5

MHSVTAEVVRLPMLDTLPLAPPTDTPLFRPKVPPPLTRPLGLFAFLKKVRENPIATWMDVHFEEFVVAGETAM
GRITVVSDPALVRYLLVERAAHYRKDDLQKRVLAPGLGNGLLTAEGDEWRLQRRTLAPIFSARHVAGFVAQME
AAGARLGRRLARRDGATVDIALEMTRATLDVLEQTIFTQGLPGDPDALGRAITRLLESIGPIDPLDVFGFPAF
VPRLGRLRARPALRFFAEVVDTLLNERKAALARGEAPHDLMTLLLAAQDPETGRGLSDLEVKANIVTFIAAGH
ETTANALTWALYCLSQDEAARARVEAEVDAAAGPDGALQLDRLPFTKAVMEETMRLFPPVPFLSRQALREDRI
GRVKIPRNSTVIIAPWVMHRHRKLWEEPDAFIPERFLGERRDRVERFAYLPFGAGPRVCIGQSFSVQEATLVL
AHVARAVRLTLPADHPPVTPLHRVTLRPKEGLRMLAQRRA

>CYP173B3(Mrad2831_4522)*Methylobacterium radiotolerans*

MSATIDLPEPATRFRPAVPPPLKEPLGLFEFLRAARKNPITTWMDAHFKLPVVAAEGAMGRITLVSDPALIRH
LLIENADGYRKDDLQRRVLAPGLGNGLLSAEGDEWRLQRRTLAPIFNARTVQGFSDAMNAAGARLGRRLVRRD
GKPVDVALEMTRVTLDVLERTIFTQGLPSDPDALGRAITRFLEAVGPIDPLDVFGVPDFVPRIGRLRARPAGR
FFAEVVDELIARRKALMARGEAPRDLLTLLLAAQDPETGNGLTDLAVKANIVTFIAAGHETTANSLTWALYCL
SQDPAAQARVEAEIDAAGPGDFAVERLPFTRAVIEEAMRLFPPVPFLSRQAIRDDRLGRIKVPRGSLVMVAPW
VLHRHRLLWEDPEAFVPERFLPGNRDAIPRFAYLPFGAGPRVCIGQSFSIQEAVILLAHVVRAVRFRLPADHP
PVTPLHRVTLRPEHGLRMEATARR

>CYP173B3(MOC_5180)*Methylobacterium oryzae*

MSATIDLSEPAARFRPVVPPPLKAPLGLFDFLRAARKNPITTWMDAHFKLPVVAAEGAMGRITLVSDPALIRY
LLIENADGYRKDDLQRRVLAPGLGNGLLSAEGDEWRLQRRTLAPIFNARTVQGFSEAMNAAGARLGRRLVRRD
GKQVDVALEMTRVTLDVLERTIFTQGLPSDPDALGRAITRFLEAVGPIDPLDVFGVPDFVPRIGRLRARPAGR
FFAEVVDELIARRKALMARGEAPRDLLTLLLEAQDPETGNGLSDLAVKANIVTFIAAGHETTANSLTWTLYCL
SQDPAAQARIEAEVDAAGPGDFSAERLPFTRAAIEEAMRLFPPVPFLSRQAIRDDRLGRIKVPRGSLVMVAPW
VLHRHHLLWEDPEAFVPERFLPENRDTIPRFAYLPFGAGPRICIGQSFSIQEAVIVLAHVVRAVRFRLPADHP
PVTPLHRVTLRPEHGLRMDAVARG

>CYP173C2(Maq22A_1p37300)*Methylobacterium aquaticum*

MTSPAAAVAAPFRPAVPRPRTEPMGLFSFLRAARANPLTTWYEEHFEKPIVTGEGALGRVTVVSEPSAIRHVM
VDNAANYRKDDLQRRVLAPGLGNGLLTAEGDEWRLQRRTLAPIFSPRHVARFEAPMAEAAARLARRLKRRDGQ
SVDAGLEMTRVTLDVLERTIFTHGLAREPDALGRAITRYFEALGPIDPLDVFGMPDWLPRIGRLKARPALRFF
AEVVDELIERRRALVASGKEAPHDLLTLLLQAQDPETGRGLSDLEVRANIVTFIGAGHETTANALTWTLYCLS
QDETAREAVEAEIDAAFAQDPAPGLEALPHTKAALEEAMRLFPPVPFMSRQALAEDRIGRIKIPRGSLVTVAP
YVLHRHRRLWEEPDAFIPERFLPENRARIDRYAYLPFGAGPRVCIGASFSVMEATLVLAHVLHAVRLDRAPEA
GPVVPLHRVTLRPQDGLRMRVTARG

>CYP173C3(Mnod_0696)*Methylobacterium nodulans*

MSDAAVAARPAQSPFRPAVPRPRTEPMDLFAFLRAARANPLTTWFREHFEHLIVAGDGALGRVTVVSDPAAIR
HILVDNAANYRKDDLQRRVLAPGLGNGLLTAEGEEWRLQRRTLAPIFSPRHVAGFQAPMSEAADRLAGRLARR
SGQTVDVALEMTRVTLDVLERTIFTHGLPRKPEALGRAMTSYFEALGPIDPLDVFGLPDWVPRIGRIRARPAL
RFFAEIVDELIARRRALLAGGEAPHDVLTLLLRAQDPETGRGLSDLEVRANIVTFIGAGHETTANALTWTLYC
LSQDEAARERAEAEIDAAFAGDPAPCSEALPFTRAALEEAIRLFPPVPLMSRQALAEDRLGRIKIPRGSLVTI
APYVLHRHRRLWQDPDAFVPERFLPENRARIDRFAYLPFGAGPRVCIGMSFSLMEATLVLAHLMRAVRLDRSP
GAGPVVPLHRVTLRPRDGLRMRVTRRAAAPPE

>CYP173C4(M446_1094)*Methylobacterium* sp. 4-46

MIEARPPQAPFRPAVPRPRTEPMDLFAFLRAARANPLTTWFREHFEHLIVTGEGALGRITVVSDPAAIRHILV
DNAANYRKDDLQRRVLAPGLGNGLLTAEGEEWRLQRRTLAPIFSARHVAGFQAPMSEAAAVLARRLARRDGQA
VDVALEMTRVTLDVLERTIFTHGLPRDPDALGRAITRYFEALGPIDPLDVFGMPDWVPRIGRLRARPALRFFA
EVVDELIARRRALLAAGEAPHDLMTLLLRAQDPETGQGLSDLEVKANIVTFIGAGHETTANALTWTLYCLSQD
EASRARAEAEIDAAFAAEAQPRADAFPFTRAALEEAIRLFPPVPFMSRQAIADDRLGRIKIPRGSLVTVAPYV
LHRHRRLWDDPDAYRPERFLPEFRGRIDRYAYLPFGAGPRVCIGMSFSLMEATLVLAHLLRAVRLDRSPEAGP
VVPLHRVTLRPRDGMRMRVTRRESGR

>CYP173G1(Mmar10_0514)*Maricaulis maris*

MPKDVSMTVAFTPPKPPCAQYSGVGKLELSDYWTYFRAMQRNPLEVWGEQHFSIRMAPFQFLGRSSLLLNDPA
GIHHCFVTNAANYGMNPVRQAVLKPFLRDGLLTAEGDVWKTARKAVSPVFTPRRVNGFAPQIRTVCDATLSAL
AERDGQTVDASDLMVELTLDVLIETLFSGDEALDKARFTRGICDLLEISGIPHPFDLIHLPGWIPRIGQGKSR
RVIKDLREQVGTVASARRAAPSAKEAGRTPDFLDLLLGAGLDDTAVIDNLLTFLAAGHETTARTLAWTLYLLS
RDEEARTRLEAEVDGADLTGTDPANWSSLLPFTEAVLKESLRLYPSAPILARTSKAWDLVAGLPVPAGSDVLV
STWLLHRQRDIWEHADEFRPDRFLGEAASAIPRDAYLPFGLGPRVCIGARFAMMEMVIVMACLASRLRLDFCG
DREPVPVMRITLQPDTAMPMRVSRR

>CYP173H1(Hden_0697)*Hyphomicrobium denitrificans* ATCC 51888

MSLTLSLSEYVSTGPRPRPAAPRPPEKPQQLLSLLWTLNKNPLEAWPQSHYEEPIVTGNTVLGRFAMVSSPDA
IRHVLVEKTENYRKDDLQNLLLRPALGNGLLTSSGNEWRMQRRSFAPVFSPRKVTSYAPAMLNEAAALASRWR
SLDGKIVDICEEMARSMLDTLATTLFSRGIGRDPAEFRRAATRYFETQGRIDPLDLIGAPAWLPRIGRLLSRP
ALKFFPLVVNAILEERRRAADAPTNPEDWDFVDTLLNARDEATGAPISEEEIAANIITFIGAGFETPANALSW
TLYLLSIDETWRRKVEAESDRLLPDLQTGNVKIDEFIYTRALIEEAMRLYPPVAILSRTALHDHVLSGRKIVA
GTTVIIAPWILHRHRMLWNDPDCFDPARFLPQNRSNIERLSYIPFGAGPRVCIGATYAMQQIIITIATLARHY
RLDLVPGHAVLPVHRVTLRPEHGMKMSIMRRR

>CYP173J1(Bind_0833)*Beijerinckia indica*

MRQSINRATHTPSGQASKPLYPPAPRPQKAPLGPFTMLRLLRRNPIETWTEAHFQLPILIGPTILGQVAVLND
PAAIRRVFVDNASNYHKDRLQKRILGPGLGQSVLIAEDADWRLQRRILAPLFTPKTVATFAKATQECASEMVA
RWQRLPEESPLDIHQEMTKITLEILGRTLFADGLDRRPEEVTAAMTRYFETIGSLDPFDLLDLPNWLPRWNQW
RAKSALDFLGDTVSALISRRKRLLARGVPNLQRDLLTLLLEAEDPETGVGLTETEVQANIVAFIGAGHETTAN
ALTWSLYLLSLSEEWRARLAAEADRVLDMPVESQAEHLIETRAVIEEAMRLYPPVASLSREALDYDDLAGRRI
RKGTVVMVSQWVLHRHRLLWEDPDRFDPTRFLPGQREKIDRFAYLPFGAGPRVCIGAGFAMQEATLVLAQILR
AVRLETKKGFKAEPVQHITLRPRGGMPMILHHRLQRHGKIRH

>CYP173J2(Msil_1926)*Methylocella silvestris*

MMDFSPAAAVLPRPPAPAPRAGPLGPLQTMWVLRKNPIETWTRAHFELPILVGPTILGTIAVLSEPGAIRRFL
VDNAANYRKDALQKRVLGEGLREGLLMAEGDDWRVQRRTLAPLFTPKTIASFAPAMNLAAEKLVARWMNFRDG
RLFDVQPEMARVTLDALGRTLFSDGLGRDPSEFTTALTRYFLTLGTLDPFDLLDFPDWAPRFGRSGGRAALGF
FEDVVDTIVARRKRLIAQDAGAAPRDLLTLLLEAQDPQTGAGLTGAEVRSNIVTFIGAGHETTANALIWSLFL
LSISPEWRARLAEEADAVLAGPIEQYAERLVETRAVIEEAMRLYPPVASISREAIDFDTLAGRRIRKRTLVMV
SQWVLHRHRLLWDEPDVFDPRRFLPGAREKIDRFAYLPLGAGPRICIGGAFALQEAAIILAHIMRSFTLETRK
GYEVKPVQHITLRPEGGLPMILRRRRV

>CYP180D1(OSB_15900)*Octadecabacter temperatus*

MKPQLAADLSDQGHFDHGIPQDVFKDLRAMDGLAWNPLPSEGPDEGFWSVGRFDDVLAVSRDPETFSSATGHI
QMYNIDDDALSARASMIDMDPPDHTRLRRLVNPGFIPKVIRTYTDIVRERASALLDDMMSQGGGDWVSKVAKP
IPIGIICDMLGVPEEDRDLMIEMTDYLVAGTSAEPLDPNAYGNTTPLRLLPFNSPAAHGLREYARKLGEERRA
NPKDDLISQLVTMEIDGEMLTDEEYTNFFRLLIFAGNETTRTAMSHMALQLSQYPEQFARLKDEPELIDTAVE
EIVRYSSPIMYFRRTATKDTELSGTKIAAGDRVVMWYASANFDDTRFDDAQTFDIARPKMPIHAGYGGGGVHT
CLGAGLARIELKVLLEEILARDLKIEIDGEPEYVNTNFVNGVEVLNIRLTQGTGA

>CYP186K1(AMJ98_PE00057)*Rhizobium* sp. N1341

MNARSHAIFPRVNRAGWFDTLRVLTAVVAPTVAKGIIIRRPTMERLAERQRFDTNAVKQMQRLKHKYGPAPLL
LPIPLRPQLLILDPADVATVLQSSPEPFAAATAEKHAALVHFEPENVLISSGSRRAELRPAHEHALATADRLH
PFTVHFKNIVDAELLHLLNLPRELDWPAFSQAWFRIVRRVVLGERARNDVTLTETLNELRGRANWAFAAPVDQ
HKLAQFHEQVGRYLRDPEKHSLVNRFPSGDELAPENQVAQWLFAFDAAGIAVMRTLALLACHVSYWGKAVEEA
MGGNLDRPFTRSCLLEVLRLWPTTPAILRQLTKDIQSGDRVIARGTGVIIFVPFFHRDPALLYANRMEPSIWG
QNDALPAAGLVPFSAGPVICPAHNLVPAIASLAVGTFLSTADIVLLHPSLTVANLPGTLNHFDIRLKLTARRR
EK

>CYP186K1(AMK02_PD00059)*Rhizobium* sp. N731

MNARSHAIFPRVNRAGWFDTLRVLTAVVAPTVAKGIIIRRPTMERLAERQRFDTNAVKQMQRLKHKYGPAPLL
LPIPLRPQLLILDPADVATVLQSSPEPFAAATAEKHAALVHFEPENVLISSGSRRAELRPAHEHALATADRLH
PFTVHFKNIVDAEFLQLLNIPRELDWPAFSQAWFRIVRRVVLGERARNDVTLTETLNELRGRANWAFAAPVDQ
HKLAQFHEQVGRYLRDPEKHSLVNRFPRGDELAPENQVAQWLFAFDAAGIAVMRTLALLACHVSYWGKAVEEA
MGGNLDRPFTRSCLLEVLRLWPTTPAILRQLTKDIQSGDRVIARGTGVIIFVPFFHRDPALLYANRMEPSIWG
QNDALPAAGLVPFSAGPVICPAHNLVPAIASLAVGSFLSAADIVLLHPSLTVANLPGTLNHFDIRLKLTARRR
EK

>CYP186K2(IE4803_PC00042)*Rhizobium etli* bv. phaseoli IE4803

MNARSHAVFPRVNRAGWTDTLRVIMTIVAPTAAKGIIIRRPTIERLAERQDWNAKAVKQMQRLKKKYGPAPLL
LPIPFRPQLLLLDPADVSTVLQSSPEPFAAATPEKHAALAHFEPKNVLVSSASRRAELRPAHEQALATADRLH
PNSAHFKGVIDAEFLQLLGDIRSNRSGELDWPAFSQAWFRVVRRLVLGERARNDIKLTETLNDLRERANWASA
APVDRRELALFHQQVGDYLSEPDKHSLVSRFPKGGEFAPESQVAQWLFAFDAAGITVIRTLAMLACHVSYWGK
AVEESMSGDLDRPFTRSCLLEVLRLWPTTPVILRQLTEDIKCGGRIIPRGTGVIIFAPFFHRDPEFLYANRMD
PSIWGPNDALPAAGLVPFSAGPVICPAHNLVPTVASLAVGALLSAADVVLLQPSLAVDNLPGTLDHFAIRLRL
AARRREK

>CYP186K2(IE4771_PD00043)*Rhizobium etli* bv. mimosa IE4771

MNARSHAVFPRVNRAGWTDTLRVIMTIVAPTAAKGIIIRRPTIEHLAERQDWNAKAVKQMQRLKKKYGPAPLL
LPIPFRPQLLLLDPADVSTVLQSSPEPFAAATPEKHAALAHFEPKNVLVSSASRRAELRPAHEQALATADRLH
PYSAHFKGVIDAEFLQLLGDIRSNRSGELDWPAFSQAWFRVVRRLVLGERARNDIKLTETLNDLRGRTNWASA
APVDRRELALFHQQVGRYLSEPDKHSLVSRFPKGGEFAPESQVAQWLFAFDAAGITVIRTLAMLACHVSYWGK
AVEEAMSGDLDRPFTRSCLLEVLRLWPTTPVILRQLTEDIKCGGRIIPRGTGVIIFAPFFHRDPEFLYANRMD
PSIWGPNDALPAAGLVPFSAGPVICPAHNLVPTVASLAVGALLSAADVVLLQPSLAVDNLPGTLDHFAIRLRL
AARRREK

>CYP186K3(AMC79_PD00042)*Rhizobium phaseoli*

MNARSHAIFPRVNRAGWIDTLRVMTRVIVPTVAKGTIIRRQTMERLAERQSLHIRAVKEMQRLKDKYGPSPLL
LPIPFRPQLLILDPADLATVLRSSPEPFAAATAEKHAALAHFEPENVLISSTARRAELRPAHEHALATADRLH
PYSIHFKRIVDTEFLQLISGIGSNRPNELDWLGFSQAWLRVVRRVVLGERARNDVTLTETLNELRERANWPFT
ARVDRRKLALFHQQLAGYLGEPDKHSLVGRLPTSDQIAPEDQVAQWLFAFDAAGIAVMRTLALLASHASYWAP
AVEEAMGGRLDRPFTRSCLLEALRLWPATPAILRHLTTEIRSADRIVPSGAGVVIFAPFFHRNPALLYANRME
PSIWGENDALPDAGLVPFSAGPVICPAHNLVPTIASLAVGTVLSESGLALIDPVLPVDNLPGTLDHFAIRLRL
SARRREK

>CYP186K4(RLEG3_33000)*Rhizobium leguminosarum* bv. trifolii WSM1689

MNSRNQAVFSNARATGFVRINRASWIDTLRVVAAVMVPTAAKGVIIRRPIMERLAQRQDLDTGAVRQMQRLVK
KYGRAPLLLPIPFRPQLLVLDPADVSTVLECSPQPFATATWEKRAALAHFEPKNVLISPASRRAELRPIHEHA
LASADGLHPSSARFKTIVDSELLEILDGIGSGGPNELDWSAFSLAWFRIVRRTVLGDRARNDVTLTKTLNDLR
SRANWAFAASIDRHKLRQFQQQLGQYLRQPEEGSLIGRLPKGSDFDLESQVAQWLFAFDAGGIAVFRTLALLA
CHPSYWAKAVDEVKDPELERPFTRNCLLEALRLWPTTPVILRELTEDIRSGDRTVARGTGVIIFAPFFHRDPE
LAYANRMEPSIWGPNDTLPTVGFVPFSAGAAICPAHNLVPAIASFAIGALLSQADITLLQPSLTVGDLPGTLN
HFDIRLRLTRQRVGAGAPHAAAGFPPH

>CYP186K5(REMIM1_PF00058)*Rhizobium etli*

MNARSHAIFPRVNRAGWLDTLRVLTAVVAPTVAKGIIIRRPTMERLAERQGFDTTAVKQMQRLKSKYGPAPLL
LPIPLRPQLLILDSADVATVLQSSPEPFAAATMEKHAALVHFEPENLLISSNSRRAELRPAHEHALATADRLH
PYAMHFKKIVDAEFLEVLNLPRELDWPAFSQAWFRIVRRIVLGERARNDVTLTETLDELRGRANWAFAAPVDQ
HKLARFHEQVGRYLVDPEKHSLVNRFPRGDELAPENQVAQWLFAFDAAGTAVIRTLALLACHVSYWGKAVEEA
MGVDLDRPFTRSCLLEVLRLWPTTPAILRQLTKDIQTGDRVIARGTGVIIFVPFFHRDPALVYADRMEPTIWG
RNDALPAAGLVPFSAGPVICPAHNLVPAIASLAVGTFLSSADIVLLHPSLTVANLPGTLNHFDIRLKLTARRR
ENR

>CYP191B1(JI59_08790)*Novosphingobium pentaromativorans*

MDVTAIKAQDYDPFSPEVMRNPLPYYAELRRSDSIFYSAKYDAFFLSRFSDILELLGYTDNTFIASEGTLPVP
AALAVHNAGAPALPPTNPFPLSQRLGMPIHGEVRRAHMKPLLPRSVTELRDFVSKRANERLDELIAQRHFDLT
REFGGIVSGNVIARLLGMPLELGETVLDLVNAGSLADPVTGGVDTRAAAQKVIALILPWVERRFEAGADGSYP
LIDGMIGYRFENEALTPEQVAQQIACAVIGGVETVPKVVAHGLMELNARPEQMAAVRADLKAAVPKLAEEMIR
FCAPAQWFMRTAHKPVTIAGQRIEPGQRVFYIAASAARDEREFDEPDEFRWDRPIRRTLAFGHGMHFCIGTHL
ARMEVQVLVETFLSRVHRFHFDLEESVRLPSSFQWGWNSLSVSIEEAE

>CYP192A1(CC_2997) *Caulobacter vibrioides* CB15

MDADVRSAPLIPPAPKVHPRQLGGSFVGELRIALEMSRNLMGAWCEEDFDNLFTPYVFMGQPGMVVSDPAAAR
RILSSPNYVRPVKAARSVRPIAGDGLLLSEGETWRRQRKSLAPVFTPMAVEGLLPHFVAAGASLAEALSGHAR
ADLSEAFHHATLDAVLSALFSRRADAQGDQLAYMVRRYMEGPAHFNLMDFVSRGADDLTFLDVERRRQGAAWF
QAVEHLIAQRQAHPHAEARDLLDRLLAARDEDGAPLSNQEIRDQCGTMLVAGFETTSRLLFWATYLLALDPAT
QDRLRAEVLAAPAAAVRTLDDLQAWPLMRSVLFETLRLYPTAPLLAREAIGPDTVMGHAVVPGQIITISPWLI
HRHRKLWDAPTAFVPDRFIDQPHPWGIEAFLPFGAGPRVCIGASFALAEAQIVLASLLERFEIGLVSDRPVIP
IASITLGPDHAPAFTLTPVS

>CYP192A1(CCNA_03092) *Caulobacter vibrioides* NA1000

MDADVRSAPLIPPAPKVHPRQLGGSFVGELRIALEMSRNLMGAWCEEDFDNLFTPYVFMGQPGMVVSDPAAAR
RILSSPNYVRPVKAARSVRPIAGDGLLLSEGETWRRQRKSLAPVFTPMAVEGLLPHFVAAGASLAEALSGHAR
ADLSEAFHHATLDAVLSALFSRRADAQGDQLAYMVRRYMEGPAHFNLMDFVSRGADDLTFLDVERRRQGAAWF
QAVEHLIAQRQAHPHAEARDLLDRLLAARDEDGAPLSNQEIRDQCGTMLVAGFETTSRLLFWATYLLALDPAT
QDRLRAEVLAAPAAAVRTLDDLQAWPLMRSVLFETLRLYPTAPLLAREAIGPDTVMGHAVVPGQIITISPWLI

HRHRKLWDAPTAFVPDRFIDQPHPWGIEAFLPFGAGPRVCIGASFALAEAQIVLASLLERFEIGLVSDRPVIP
IASITLGPDHAPAFTLTPVS

>CYP192A2(Cseg_3488)*Caulobacter segnis*

MDAQVRDVKVGPGPLVPPVPKVHPRPLGGSPLDDLRIAWEMSRNLIGAWCEEDFDNLVTPYHFMGQPGLVVSD
PAGVRQVLASPNFRRPLKLGRPLKPLAGEGLLLSEGETWKRQRKSLAPAFTPAAIGGLLPHFVAAGASLTEGL
ANQTKANLSEAFHHGTLDAVLRALFSRRADRDGAVLAQIARRYLEGPAHFRLLDFIGRGRDDLTFADGDRRRL
GGRWLAEVDALIAQRRAAPAADDRPGDLLDRLLTARDEDGGPLPDQEIRDQCSSMLAAGFETTSRLLFWATYL
LARDPETQDRVRAEILAFPVERVNGLDDLKAWPLLRSVLFETLRLYPTAPTFGRQALADETVLGHKVPAGAMV
TISPWLIHRHRKLWDAPAVFRPERFLDQPHPWGIEAFLPFGGGPRVCIGAGFALAEAQIILATVLARLEIALA
DDRPVIPVASITLGPDREPDFLLTPIVQARA

>CYP193A1(bll7012)*Bradyrhizobium diazoefficiens* USDA 110

MARGCASRCSRPVSDASPAPRAGTLSGARLECRLCRFMALDERCLCVCRCPLLWVMRTRYRARTPCPNRWGQT
AAPERASLAGHSRETALSTAPRIDIDPAAFWADPYPMLANMRKEAPIAFVPQLGSTLLTSRDDISISEKQIDV
FSSHQPAGLMNRLMGHNMMRKDGEAHQVERRAMFPTVSPKTVKGYWTALFQAHADRIIDAIEPGRIDFMRDFA
LPFSGECLKSITGLTNIGFAEMDAWSQGMIEGIANYVGDPAVEARCHAATSGIDAAIDDMLPVMRKNPDQSIL
GVLLASGMPMESVRANVKLAISGGQNEPRKAIAGTVWALMTHPEQLDLVRRGEVTWLQAFEEYARWISPIGMS
PRRIAKPWSIRDVAFELDERVFLMFGSANRDEKHFERADQFDVRRDTSKSVAFGAGPHFCAGAWASRAMIADV
ALPTLFARAGRIEIADDEPVRIGGWAFRGLQNLPARWLH

>CYP193A1(BF49_4496)*Bradyrhizobium* sp. BF49

MERSLSTAPRIDIDPAAFWADPYPMLAKMRKEAPIAFVPQLGSTLLTSRDDISISEKQIDVFSSHQPAGLMNR
LMGHNMMRKDGEAHQVERRAMFPTVSPKTVKAHWAALFQAHADRIIDAIEPGRIDFMRDFALPFSGECLKSIT
GLTNIGFAEMDAWSQGMIEGIANYSGDAAVEARCHAATSGIDAAIDDILPVMRKHPDQSILGVLLASGMAMES
VRANVKLAISGGQNEPRKAIAGTVWALLTHPDQLDLVRQGEVTWLQAFEEYARWISPIGMSPRRIAKPWTIRD
VAFETDERVFLMFGSANRDEKHFERADQFDVRRDTAKSVAFGAGPHFCAGAWASRAMIADVALPTLFARASRL
EIADDEPVRVGGWAFRGLQNLPVRWLH

>CYP193A1(BJ6T_23930)*Bradyrhizobium japonicum* USDA 6

MSTAPRIDIDPASFWVDPYPMLAKMRKEAPIAFVPQLGSTLLASRDDISISEKQIDVFSSHQPAGLMNRLMGH
NMMRKDGEAHQVERRAMFPTVSPRTVKAHWTALFQAHADRIIDAIEPGRIDFMRDFALPFSGECLKSITGLTN
IGFQDMDAWSQGMIEGIANYGGDPAVEARCHAATSGIDAAIDDILPVMRKHPDQSILGVLLAAGMPMESVRAN
VKLAISGGQNEPRKAIAGTVWALLSHPEQLALVRKGEVTWLEAFEEYARWISPIGMSPRRIAKPWSIRDVSFE
LDERVFLMFGSANRDEKHFERADQFDVRRDTSKSVAFGAGPHFCAGAWASRAMIADVALPTVFARAKQLEIAD
DEPVRIGGWAFRGLQNLPVRWH

>CYP193A(RN69_11690)*Bradyrhizobium japonicum* E109

MSTAPRIDIDPASFWVDPYPMLAKMRKEAPIAFVPQLGSTLLASRDDISISEKQIDVFSSHQPAGLMNRLMGH
NMMRKDGEAHQVERRAMFPTVSPRTVKAHWTALFQAHADRIIDAIEPGRIDFMRDFALPFSGECLKSITGLTN
IGFQDMDAWSQGMIEGIANYGGDPAVEARCHAATSGIDAAIDDILPVMRKHPDQSILGVLLAAGMPMESVRAN
VKLAISGGQNEPRKAIAGTVWALLSHPEQLALVRKGEVTWLEAFEEYARWISPIGMSPRRIAKPWSIRDVSFE
LDERVFLMFGSANRDEKHFERADQFDVRRDTSKSVAFGAGPHFCAGAWASRAMIADVALPTVFARAKQLEIAD
DEPVRIGGWAFRGLQNLPVRWH

>CYP193A1(BCCGELA001_31425)*Bradyrhizobium* sp. CCGE-LA001

MSTAPRIDIDPAAFWADPYPMLAKMRKEAPIAFVPQLGSTLLTSRDDISISEKQIDVFSSHQPAGLMNRLMGH
NMMRKDGEAHQVERRAMFPTVSPRTVKAHWTAQFQAHADRILDAIEPGRIDFMRDFALPFSGECLKSITGLTN
IGFQDMDAWSQGMIEGIANYGGDPAVEARCHAATSGIDAAIDDILPVKRKHPDQSILGVLLASGMAMESVRAN
VKLAISGGQNEPRKAIAGTVWALLTHPDQLDLIRRGEVSWLQAFEEYARWISPIGMSPRRIAKPWTIRDVAFE
LDERVFLMFGSANRDEKHFQRADHFDVRRDTSKSVAFGAGPHFCAGASASRAMIADVALPTVFARAKQLEIAD
DEEVRIGGWAFRGLQNLPVRWRH

>CYP193A3(Jann_3733)*Jannaschia* sp. CCS1

MNKTYDIDPKAFWRDPYPDLARIQAQAPAVFVPQLGATLLTRRDDIFEQEKRIEVFSSEQPGGLMTMLMGENL
MRKDGDAHMAERKVIFPALSPRVVKATWKRQFEAATNSILDALEPQGHCDLVTDFAMPVCGEALKVITGLTQM
PTAEMDRVSQHMLDGCANYVGDNEVEARCHAATAFIDEHIDLMVPELIKTPDASVLSVQLAAGLPMASTRANV
KLVISGGQNEPRDAIAGTAWALLAHGHALAKIAEGSASFLDAFEEYARLISPIGMSPRQVARSEHVLGHDFEV
GDRVFLMFGAANRDPRIFEAPDVFDITRNNRKSVTFGAGPHFCAGAAASRALIAEVALPKLFERLPRLRLDGG
VTFGGWAFRGPLKVPVRWF

>CYP193A4(S23_12910)*Bradyrhizobium* sp. S23321

MSTAPRIDIDPAAFWADPYPMLAKLRQEAPIAFVPQLGSTLLTRRDDISISEKQIDVFSSHQPAGLMNRLMGH
NMMRKDGEAHQAERRAMFPTVSPKTVRAHWTALFQAHADRIIDAIAPGSRIDLMRDFALPFSGECLKSITGLT
NIRYQDMDAWSQGMIEGIANYGGDPAVEARCHAATSGIDAAIDDILPVMRERPDQSILGVLLASGMPMDSVRA
NVKLAISGGQNEPRKAIAGTVWALLSHPDQLALVRSGEVSWLQAFEEYVRWISPIGMSPRRITKPWSIRDVSF
ETDERVFLMFGSANRDEQHFERADQFDVRRDTSKSVAFGAGPHFCAGAFASRAMIADVALPTLFASANKLELA
DDEPVRIGGWAFRGLQNLPVRWLH

>CYP193A5(S58_18580)*Bradyrhizobium oligotrophicum*

MSTAPRFDIDTATFWADPYPALARMRKEAPIAFVPQLGSTLLCRRNDIFVSEKQIDVFSSHQPQGLMNRLMGH
NMMRKDGAAHMAERHAIAPAVSPRAVRVHWLAQFQAHADRLIDALDPAAEIDLVRDFALPFSAECLKLITGLT
NMRFEDMNAWSQAMIDGIANYTGDPAVEARCNAATSGIDAAIDDMLPVLEKHPNQSLLGVMLAGGMPMDSVRA
NIKLAISGGQNEPRDAIAGTVWALLLHPEQLALAVNGEVRWLQVFEEYARWISPIGMSPRRIARPWSIRDVAF
EPDERVFLMFGSANRDEAYFTDPDRFDIRRDVSKSIAFGAGPHFCAGAWASRAMIADVALPTLFARLQNLRLR
EAESARIGGWAFRGLLNLPVTWDATRH

>CYP193A6(BBta_6269)*Bradyrhizobium* sp. BTAi1

MSTAPHFDIDPAAFWADPYPALARMRREAPIAFVPQLGSTLLCRRDDIFVSEKQIEVFSSHQPQGLMNRLMGH
NMMRKDGAAHLAERQAIAPAVSPRAVRVHWLTQFQAHADRLIDALDPAADVDLVRDFALPFSAECLKLITGLT
NMRFEDMNAWSQAMIDGIANYTGDPAVEARCNAATAGIDAAIDDMLPVLEKTPNQSLLGVMLAAGMPMDSIRA
NIKLAISGGQNEPRDAIAGTVWALLTHPEQLALAVHGEVRWLQVFEEYARWISPIGMSPRRIAKPWTIRDVAF
APEERVFLMFGSANRDEAHFTAPDRFDIRRDVSKSIAFGAGPHFCAGAWASRAMIADVALPTLFRRLNNLRLR
DDEPVRIGGWAFRGLLNLPVKWEATQD

>CYP193A6(BRADO5757)*Bradyrhizobium* sp. ORS 278

MSTAPHFDIDPVTFWADPYPALARMRKEAPIAFVPQLGSTLLCRRDDIFVSEKQIEVFSSHQPQGLMNRLMGH
NMMRKDGEAHLAERQAIAPAVSPRAVRVHWLAQFQAHADRLIDALDPAAEVDLVRDFALPFSAECLKLITGLT
NMRFQDMNAWSQAMIDGIANYTGDPAVEARCNAATAGIDAAIDDMLPVLETAPNQSLLGVMLAAQMPMESIRA
NIKLAISGGQNEPRDAIAGTVWALLTHPEQLALANTGAVRWLQVFEEYARWISPIGMSPRRIAKPWTIRDIAF
APEERVFLMFGSANRDEAHFIDPDRFDIRRDVSKSIAFGAGPHFCAGAWASRAMIADVALPTLFARLQNLRLR
DAEPVRIGGWAFRGLLNLPVTWDATRR

>CYP193A7(RPB_1938)*Rhodopseudomonas palustris* HaA2

MSNAPHFEINVAAFWADPYPALAKMRAQAPIAFVPQLGSTIFTRRDDIFVNEKRIDVFSSHQPAGLMNRLMGH
NMMRKDGDAHLAERTAMFPAVSPRTVKEVWRKQFQAHADRILEDLAPRGAADLVKAFALPLSGECLKDVTGLT
NISYHEMDAWSQAMIDGIANYTGDRAVEDRCHAATAGIDAAIDDMAPVVRKHPDHSMLSVLIAAGMAMDSIRA
NIKLAISGGQNEPRDAISGCVWALLTHPSEYARVVAGEASWLDVFEEYARWIAPIGMSPRRVAQPFHYRGVDF
EPEDRVFFMFGSANRDEACFSDPDVFDVGRDHAKSIAFGAGPHYCAGAFASRAMVADVALPGVFARLTDLRLD
PREPVRIGGWAFRGLLNLPVVWNSAAPN

>CYP193A8(RPD_3440)*Rhodopseudomonas palustris* BisB5

MSNAPHFEIDVASFWADPYPALARMRAEAPIAFVPQLGSTIFTRRDDIFVTEKRIDVFSSHQPAGLMNRLMGH
NMMRKDGDAHIAERSALFPAVSPRTVKDVWRAQFQAHADRILDELAPQGHADLVKAFALPLSGECLKHITGLT
NISYHEMDSWSQAMIDGIANYTGDKAVEDRCHAATAGIDAAIDDMAPVVSKHSNHSMLSVLLASGMAMDSIRA
NIKLAISGGQNEPRDAIAGCIWALLTHPAEYAKVVAGDASWLAVFEEYARWIAPIGMSPRRVAQPFHYRGVDF
EPEDRVFFMFGSANRDEACFTDPDLFDVSRDHAKSIAFGAGPHYCAGAFASRAMVADVALPSVFARLKALRLD
EGEPVRIGGWAFRGLLNLPVAWSSAAPN

>CYP193A9(LMTR13_07025)*Bradyrhizobium icense*

MSNAPHFDIDVPTFWADPYPALAKMRKETPIAFVPQLGSTVFTKRDDIFTQEKRIDVFSSHQPNGLMNVLMGH
NMMRKDGEAHMTERQAMFPAVSPRTVRDTWVRQFQAHADRILDELTPKGEADLCKAFALPLSAECLKDVTGLT
NMRYQDMDAWSQAMIDGIANYTGNKEVEARCHAATAGIDAAIDDMIPVVKKHPNTSILSVLLASGQNMESIRA
NIKLAISGGQNEPRDAISGATWALLTHPEQLAVVREEKAKWIDVFEEYARWIAPIQMSPRRVAKPWTYHGVDF
EPEDRVFFMFGSANRDEACFSDPDRFDITRDTQKSIAFGAGPHYCAGAFASRAMVADVALPSLFARLKRLRLD
EREPVRIGGWAFRGLLNLPVKWGAG

>CYP193A10(BSQ44_19500)*Mesorhizobium* sp. B7

MSDAPRYEIDPAAFWRNPYPDLARMRESAPIAFVPQLGATLFSRRDDIFVCEKNVAVFSSHQPGGLMNRLMGH
NMMRKDGDAHLSERRAIFPSVSPKTVRDVWKQTFEAHADTVLDELDPRAGADLVKDFALRFSAECLKSLTGLV
NMRSQDMDAWSQAMIDGIANYAGDPAVEARCHSATSGIDSAIDDMVPVVRAAPNASLLSVMLDGGMPMESVRA
NVKLAISGGQNEPRDAIAGAVWALLTHPSERARVEAGEASWMQVFDEYCRWLAPIGMSPRRVAVRHRYGDITF
EPEDRVFFMFGSANRDEAHFIDPDRFDVSRATSKSIAFGAGPHFCAGAWASRCMVAEVALPKLFGRLRGLQIE
EGAQVRIGGWAFRGILNLPVRWKPR

>CYP193A11(SPO0226)*Ruegeria pomeroyi*

MTTRTPQNTHCVDMTTGSRAMSDAPVYEIDLAAFWADPYPDLKRMRAEAPIAYVPQLGATLITRRDEIFAQEK
RIEIFSSDQPQGLMTVLMGQNMMRKDGDAHMAERKAIFPTVSPKTVKQVWVAQFRAAAQRVLDDLAPRGACDL
VRDYAMPVSAEALKAITGLTNMTAAEMDRVSQGMIDGCANYAGDPLVEARCHDCTASIDAHIDAMIPVLDARP
DHSLLSVQRRAGLSDAQTRANIKLAISGGQNEPRDAIAGTAWAVLSHPDALARLQSGAVPWLRAFEEYARWIS
PIGMSPRRVAQRATVQGVTLEPEDRVFLMFGSGNRDESVFANPDRFDLDQDIAPAISFGAGPHFCAGAWASRA
LIGEVALPLLFERLPGLALDGEARFGGWAFRGPITVPVRWEPAPREG

>CYP193A12(BOO69_09450)*Sulfitobacter* sp. AM1-D1

MTDAPHFSIDPAAFAADPYPALARMRREAPIAFVPELGATLLTRRDDIHRQEKRIEVFSSHQPDGLMSVLMGE
NMMRKDGDAHQAERRALFPALSPRTVAQHWRSVFEAAANDILDDLAPLGACDLVTDYAMPVSAAALIAITGLS
GMTRAEMDRVSQHMIDGCANYLGDPEVEARCHAATALIDSHIDRSFDMPPPKSALAVQRAAGLPMQSLRANIK
LVISGGQNEPRDAIAGIAWALLTHPDQLALIRRGENTWQDAFAEYARWISPIGMSPRIVARADSVGGVRFDPG
DRVFLMFSSAGRDEAHFDRPDAFDVTRDTGAAIPFGAGPHFCAGAAAARCLIAEVALPLLFDRLPGLRLDGPV
PFKGWAFRGPTSMPVAW

>CYP193A14(BWR18_05930)*Tateyamaria omphalii*

MSSAPTVHIDPAAFHADPYPTLAAMRQNAPVCYVPEMGATLFTRRDDVFREEKRVDLFSSHQPNGLLTKVMGS
NLMRKDGEAHMRERKALFPALSPRTVRDVLGPKFREIVQGHIDGLQPLGACDLVTDYAMPVSADALRLITGLT
NMEAAEMDAASQAMLDAAANYQRDPEVDRLGYGYADHVLDLIKERLPQLRAAPDMSIISVLDQAGLTLDEIAG
NVRVIIGGGQNEPRDAIAGTAWALLTHPDQYAMMANGTADWNAAFDEYVRLVAPIGMSPRRVARPDRACDVEF
NADELIFFMFGSACRDADHFTDPNAYDLTRNTGPAIPFGAGPHFCAGAAASRALIAGHALPMLFDQLPNLRLT
DDVTFAGWAFRGPLSVPVAWDI

>CYP194A1(bll2905)*Bradyrhizobium diazoefficiens* USDA 110

MSDVSEPVAHPPVTDWVNDFDHTDPQWTDDPFPIWDELRAASPVVHTERFLGCYMPTTYEAVREIANDTEHFS
SRRIIVRDVRPEIARNAAPPITSDPPVHKPAKQLLLPPFTPDAMKKLEPRVRTICNELIDGFISDGKVDAAAR
YSKYIPVRAIAHMLGIPESDSDLFVNWIHMILELGIKDETMLLQAVQEMSAYFRTHIEERRSRPTDDLISYLM
NAKDKEGQPLEESHVLGSLRLLLIAGIDTTWSAIGSSLWHLARTPADRERLIAEPGLIPIAVEELLRAYSPVT

MAREVVKETTISGCPVKAGNMVLLSFPAANRDPKMFPDADKVVIDRRENRHAAFGLGIHRCVGSNLARMEMQV
ALEEWLKRIPDFRLDPAGTVTWSQGTVRGPRQLPFLLGKAM

>CYP194A1(S23_51100)*Bradyrhizobium* sp. S23321

MSDVSQPAAHPPVTDWVHDFDHTDPQWTEDPFPIWDELRAASPVVHTERFLGCYMPTTYEAVREIANDTEHFS
SRRIIVRDVRPEVARNAAPPITSDPPVHKPAKQLLLPPFTPDAMKKLEPRMRAICNELIDGFIADGKVDAAAR
YAKYIPVRAIAHMLGIPESDSDLFIDWIHMILELGIKDESKLLQAVQEMSAYFRTHIEERRSKPTDDLISHLM
NAKDKEGQPLEESHVLGSLRLLLIAGIDTTWSAIGSSLWHLARTPADRERLIAEPGVIPIAVEELLRAYSPVT
MAREVVKETTISGCPVKAGNMVLLSFPAANRDPKVFPDADKVVIDRRENRHAAFGLGIHRCVGSNLARMEMQV
ALEEWLKRIPDFRLDPAGTVTWSQGTVRGPRQLPFLLGKAM

>CYP194A2(RPA1732)*Rhodopseudomonas palustris* CGA009

MSERAPVTDWVNDFDHTDPRWTENPYPIWDELRSAGPLVHTDRFLGCYMPTTFAAVKEISYDTDHFSSRRVIV
RNVRSESPPPAPPITSDPPEHKPAKRLLLPPFTPDAVAKLEPRVRAICNELIDAFIEDEGCDAATAYTKHIPV
KTICHMLGIPEDDSDIFIRWIHEILELGINDDAILMKAVFEMSTYFQGHIAHRKQKPTDDLISTLMNARDDKG
QPLSDAHVLGSLRLLLIAGIDTTWSAIGAALWHLATHPADRERLLAEPELMPTAIEEFLRAYSPVTMAREVMK
ETSIAGCPVKPGNMVLLSFPAANRDPSVFPEADRVMIDRKENPHVAFGLGIHRCVGSNLARMEMTVAIEEWLK
RIASFRLDPSQKVRWSEGTVRGPRSLPLLFGKPS

>CYP194A2(Rpal_1932)*Rhodopseudomonas palustris* TIE-1

MSERAPVTDWVNDFDHTDPRWTENPYPIWDELRSASPLVHTDRFLGCYMPTTFAAVKEISYDTDHFSSRRVIV
RNVRSESPPPAPPITSDPPEHKPAKRLLLPPFTPDAVAKLEPRVRAICNELIDAFIEDEGCDAATAYTKHIPV
KTICHMLGIPEDDSDIFIRWIHEILELGINDDAILMKAVFEMSTYFQGHIAHRKQKPTDDLISTLMNARDDKG
QPLSDAHVLGSLRLLLIAGIDTTWSAIGAALWHLATHPAERERLLAEPELMPTAIEEFLRAYSPVTMAREVMK
ETSIAGCPVKPGNMVLLSFPTANRDPSVFPEADKVMIDRKENPHVAFGLGIHRCVGSNLARMEMTVAIEEWLK
RIASFRLDPSQKVRWSEGTVRGPRSLPLLFGRPS

>CYP194A3(RN69_33290)*Bradyrhizobium japonicum* E109

MSDVSQPAAHPPVTDWVHDFDHTDPQWTDDPFPIWEELRAASPVVHTERFLGCYMPTTYEAVREIANDTEHFS
SRRIIVRDVRPEISRNAAPPITSDPPVHKPAKQLLLPPFTPDAMKKLEPRMRAICNELIDGFIADGKVDAAAR
YSKYIPVQAIAHMLGIPESDSDLFINWIHMILELGIKDESKLLQAVQEMSAYFSTHIEERKKKPTNDLISYLM
NARDKEGNPLEDSHVLGSLRLLLIAGIDTTWSAIGSSLWHLAKTPADRERLIAEPGLIPIAVEELLRAYSPVT
MAREVVKETTISGCPVKAGNMVLLSFPAANRDPKMFPDADKVVIDRRENRHAAFGLGIHRCVGSNLARMEMQV
ALEEWLKRIPDFALDPAGTVTWSQGTVRGPRQLPFLFGKAM

>CYP194A4(BF49_1787)*Bradyrhizobium* sp. BF49

MSDISQPAAHPPVTDWVHDFDHTDPQWTEDPFPIWDELRAASPVVHTERFLGCYLPTTYEAVREIANDSEHFS
SRRIIVRDVRPEITSRNSAPPITSDPPVHKPAKQLLLPPFTPDAMKKLEPRVRAICNELIDGFITEDRLDAAE
RYTKHIPVRAIAHMLGIPETDSDLFIRWIHMILELGIKDENTLLQAVQEMTVYFSGHIEARKTKPTDDLISYL
MNARDKDGNPLEDSHVLGSLRLLLIAGIDTTWSAIGSSLWHLARTPADRERLVAEPGLIPTAVEELLRAYSPV
TMAREVVKETTISGCPVKAGNMVLLSFPAANRDPKMFPDADKVVIDRRENRHAAFGLGIHRCVGSNLARMEMQ
VALEEWLKRIPDFALDPAGTVTWSQGTVRGPRQLPFLLGKAM

>CYP194A5(RPD_1836)*Rhodopseudomonas palustris* BisB5

MTERTPVTDWVNDFDHTDPRWTDDPFPIWNEMRSAGPVVHTERFLGCYMPTTFAAVKEISYDTDHFSSRRVIV
RNSRPEPVQSAPPITSDPPEHKPAKRLLLPPFTPDAVAALEPRVRAICNELIDQFIENGSCDAAADYSKHIPV
KTICVMLGIPESDSDQFIQWIHEILELGINDDAVMMKAIQEMSVYFLGHIAKRKQHPTDDLISTLMNARDASG
QPLSDVHVLGSLRLLLIAGIDTTWSAIGATLWHLATHPDDRARLVAEPELMPTAIEEFLRAYSPVTMAREVMK
ETTISGCPVKPGNMVLLSFPSANRDPSVFPDADQVRIDRKENPHVAFGLGIHRCVGSNLARMEMTVAIEEWLK
RIPEFRLDPSQKVRWSEGTVRGPRKLPLLFGRAN

>CYP194A6(RPB_3631)*Rhodopseudomonas palustris* HaA2

MTERAPVTDWASDFDHTDPRWTENPYPIWDELRAASPVVHTDRFLGVYMPTTFAAVKEISYDTDHFSSRRIIV
RNSRPEPVQSAPPITSDPPEHKPAKRLLLPPFTPDAVAKLEPRVRSICNELIDDFIADGRCDAAKAYSKHIPV
KTICAMLGIPETDSDRFIQWIHEILELGIHDDAMLMKAIQEMSVYFAGHIAKRKQHPTDDLISTLMNARDADG
QPLSDVHVLGSLRLLLIAGIDTTWSAIGAALWHLATHPEDRVRLVAEPDLMSTAIEELLRAYAPVTMAREVMK
ETTIAGCPVKPGNMVLLSFPAANRDPDVFPDADRVKIDRQENPHVAFGLGIHRCVGSNLARMEMTVAIEEWLK
RIPEFRLDSSQNVRWSEGTVRGPRQLPLLLGQPS

>CYP194A7(Nham_2693)*Nitrobacter hamburgensis*

MSSPAPVTDWVRDFDHTDPRWTENPYPIWDELRAECPVAHTDRFLGVYLPTTYEAVKEICYDTEHFSSRRVVV
RNVRPEPPPPAPPITSDPPAHKHAKQILLSPFLPDAVRQLEPRVRAICNDLIDGFINDKSCDAAARYTKRIPI
RAITHMLGVPEKDGDLFIRWIHEMLELGVHDDNMVMKAIHEMVAYFAEQIESRKKHPTDDLISTLMNARGEDG
QPLTDLHVQGALRLLLVAGLDTTWSAIGSSLWHLAQTPADRARLVAEPELLPTAIEEFLRAFSPATSGREVMK
ETTVSGCPMKPGNTVLLSFPAANRDPAVFPDADKVIIDRKENRHVAFGVGIHRCVGAHLARMEMLVAIEEWLK
RIPDFRLDPSGEITWSEGSVRGPRQLPLLLGTG

>CYP194A8(Nwi_2279)*Nitrobacter winogradskyi*

MSSPAPVTDWVHDFDHTDPRWTENPYPIWDDLRARCPIAHTNRFLGVYLPTTYEAVKEISRDTEHFSSRRVVV
RNFRPEPPPPAPPITSDPPAHKHAKQILLPPFAPEAVAKLKPRIRAICNELIDGFINEKGCDAAARYTRYIPT
RAITIMLGVPEKDGDLFIKWIHEILEVGLNDDAAVMQAVREMVGYFAEQIEARKKHPTDDLIATLMNARSEDG
QPLTDMHVQGALRLLLVAGIDTAWSAIGSSLWHLAQTPADRERLIAEPELIPTAIEEFLRAFAPATSGREVMK
ETTVSGCPMKPGNMVLLSFPAANRDPAVFPDADKIIIDRKENRHIAFGVGIHRCVGAHLARMEMLVAIEEWLK
RIPEFRLDPSGGVKWSEGSVRGPRQLPLLLGKA

>CYP195A1(bll2856)*Bradyrhizobium diazoefficiens* USDA 110

MNADAKELAASFDLEKLTPEFYDNPYPTYRALRENEPVKRLPNGTVFLTRYDDLVTTYKNTKSFSSDKKREFA
PKYGNTPLYEHHTTSLVFNDPPAHTRVRRLIMGALSPRAIAGMEADLIKLVDGLLDAIAAKGSCELIEDFAAS
IPIEVIGNLLDVPHDERTPLRDWSLAILGALEPVVSPEAAARGNKAVKDFLSYLETLVARRRGKPGNPERDVL
TRLIQGEGNGEENGERLTEKELLHNCIFLLNAGHETTTNLIGNGLVALDRHPDQKQRLIDHPDMIKTAVEEML
RYESSNQLGNRMTTERVELGGVMLDAGTSVTLCIGAANRDPAQFPDPESFDIARTPNRHLAFATGAHQCAGMA
LARLEVAIAISRFLARFPNYAVNGRPVRGGRVRFRGFLSVPCAIG

>CYP195A1(RN69_33545)*Bradyrhizobium japonicum* E109

MNASAKELAANFDLEKLTPEFYDDPYPTYRALRENEPVKRLPNGTVFLTRYDDLVTTYKNTKSFSSDKKREFA
PKYGDTPLYEHHTTSLVFNDPPAHTRVRRLIMGALSPRAIAGMEPDIVKLVDGLLDAIAAKGACELIDDFAAS
IPIEVIGNLLDVPHDEREPLRDWSLAILGALEPVVSPEVAARGNKAVKDFLAYLETLVARRRQKPGNPERDVL
TRLIQGEGNGEENGERLTEKELLHNCIFLLNAGHETTTNLIGNGLVALHRNPDQKQRLIENSDMIKTAVEEML
RYESSNQLGNRMTTEKIELGGVMLDAGTSVTLCIGAANRDPAQFPEPERFDIARTPNRHLAFATGAHQCAGMA
LARLEGAIAISRFLARFPNYAVSGHPVRGGRVRFRGFLSVPCSIG

>CYP195A2(RPA3778)*Rhodopseudomonas palustris* CGA009

METAPAELAEAFDLARLTPDFYDNPYPTYHALRAHQPVKRLASGGYFLTRYDDLVAVYKNTTLFSSDKKREFT
PKYGDSLLFEHHTTSLVFNDPPSHTRVRRLIMGALTPRAIAGMEPDLIALVDRLLDAMAAKGRVDLIEDFASA
IPIEVIGNLLGVPHDERGPLRGWSLAILGALEPVIGPEAFALGNAAVAEFLGYLDTLIARRTAEPGDPERDVL
TRLIRGEAGGEKLTAKELLHNCIFLLNAGHETTTNLIGNGLVTLAANPDQKRRLIAEPALIKTAVEEILRYES
SNQLGNRITTAEVEIGGVSMPANTSLTLCIGAANRDPAQFADPDRFDVSRSPNRHLAFASGPHQCAGMALARL
EGAIALSRFLAHFPDYVLDGPPQRGGRVRFRGYLGVPCRLG

>CYP195A2(Rpal_4299)*Rhodopseudomonas palustris* TIE-1

METAPAELAEAFDLARLTPDFYDNPYPTYHALRAHQPVKRLASGGYFLTRYDDLVAVYKNTTLFSSDKKREFT
PKYGDSLLFEHHTTSLVFNDPPSHTRVRRLIMGALTPRAIAGMEPDLIALVDRLLDAMATKGRVDLIEDFASA
IPIEVIGNLLGVPHDERGPLRGWSLAILGALEPVIGPEAFALGNAAVAEFLGYLDTLIARRTAEPGDPERDVL

TRLIRGEAGGEKLTAKELLHNCIFLLNAGHETTTNLIGNGLVTLAANPDQKQRLIAEPALIKTAVEEILRYES
SNQLGNRITTAEVEIGGISMPANTSLTLCIGAANRDPAQFADPDRFDVSRSPNRHLAFASGPHQCAGMALARL
EGAIALSRFLARFPDYVLDGPPQRGGRVRFRGYLSIPCRFG

>CYP195A7(BF49_1839)*Bradyrhizobium* sp. BF49

MNASANELAASFDLEKLTPEFYDNPYPTYRALRENEPVKRLRNGTVFLTRYDDLVTTYKNTKSFSSDKKREFA
PKYGETPLYEHHTTSLVFNDPPAHTRVRRLIMGALSPRAIAGMEPDIIKLVDGLLDAIAVKGNCELIEDFAAS
IPIEVIGNLLDVPHDERAPLRDWSLAILGALEPVVSPEVAVRGNKAVTDFLGYLRTLVARRRERPGNPERDVL
TRLIQGEDNGERLTEKELLHNCIFLLNAGHETTTNLIGNGLVALERNPDQKQRLIDHPELIKTAVEEMLRYES
SNQLGNRMTTERVELGGIMLDAGTSITLCIGAANRDPAQFPDPESFDIARTPNRHLAFATGAHQCAGMALARL
EGAIAVSRFLARFPNYAVSGQPVRGGRVRFRGFLSVPCAIG

>CYP195A8(S23_51560)*Bradyrhizobium* sp. S23321

MNASAKELAASFDLTRLTAEFYDDPYPTYRALRENEPVKRLPNGTVFLTRYEDLVTTYKNTKSFSSDKKREFA
PKYGDTPLFEHHTTSLVFNDPPSHTRVRRLIMGALSPRAIAGMEPDIVRLVDGLLDAIAAKGACELIEDFAAS
IPIEVIGNLLDVPHDERGPLRDWSLAILGALEPVVSSEVAARGNKAVTDFLAYLQTLVARRRENPGNPERDVL
TRLIQGEENGERLTEKELLHNCIFLLNAGHETTTNLIGNGLVALDRNPDQKQRLIDNPDMIKTAVEEILRYES
SNQLGNRMTTEAVELGGVMLDAGTSVTLCIGAANRDPAQFTDPERFDIARTPNRHLAFATGAHQCAGMALARL
EGAIAISRFLARFPTYAVSGQPVRGGRVRFRGFLSVPCAIG

>CYP195A9(BBta_2844)*Bradyrhizobium* sp. BTAi1

MNDAAALAADFDLESLTPAFYADPYPTYRALRQYAPVKRLRNGSYVLTRYDDLVTAYKATKTFSSDKTREFAP
KYGASLLYEHHTTSLVFNDPPAHTRVRRLIMGALSPRAIAEMEPALIALVDGLLDRIAAEDRCELIEDFAAAI
PIEVIGNLLGVPHEERGPLRDWSLAILGALEPVVSEAQLSRGNRAVKAFLAYLEGLVARRRAAPGNPERDVLT
RLILGEADGERLSEKELLHNCIFLLNAGHETTTNLIGNGLVLLCEHPAERQRLIDAPSLIRTAVEEILRYESS
NQLGNRMTTEPVELGGVKLEAGTSVTLCIGAANRDPAQFEDPERFDIGRLANRHLAFGTGAHQCAGMALARLE
GAVAISRFLARFPNYQLDGAPVRGGRVRFRGFASVPCRIGVGGG

>CYP195A10(S58_51130)*Bradyrhizobium oligotrophicum*

MTDDAALASQFDLERLTPEFYADPYPTYRALRQHAPVKRLRNGSYFLTRYDDLVTAYKATKTFSSDKKREFAP
KYGASLLYEHHTTSLVFNDPPAHTRVRRLIMGALSPRAIAEMEPALIALVDGLLDRIAAKDRFELIDDFASAI
PIEVIGNLLGVPHDERAPLRDWSLAILGALEPVLSAEQLARGNQAVGDFLTYLRGLVARRRTTPGNPERDVLT
RLILGEADGERLSETELLHNCIFLLNAGHETTTNLIGNGLVLLCQHPAERQRLIEEPGLIRTAVEEILRYESS
NQLGNRMTTEAVELSGVRLEAGTSITLCIGAANRDPAQFDDPERFDIARPANRHLAFGTGAHQCAGMALARLE
GAVAIARFLSRFPNYQLDGSPVRGGRVRFRGFASVPCVVEG

>CYP195A11(BRADO2499)*Bradyrhizobium* sp. ORS 278

MTDAAALADDFDLERLTPGFYADPYRTYRALRQYAPVKRMRNGSYFLTRYDDLVTAYKTTKAFSSDKKREFAP
KYGDSLLYEHHTTSLVFNDPPSHTRVRRLIMGALSPRAIAEMEPALIALVDGLLDRIAAKPRFELIEDFAAAI
PIEVIGNLLGVPHEERGPLRDWSLAILGALEPVLTPEQLARGNQAVADFLDYLKTLVARRRARPGHPERDVLT
RLIQGESDDENGGERLSEKELLHNCIFLLNAGHETTTNLIGNGLVLLCQHPAERKKLIEAPALIRTAVEEILR
YESSNQLGNRMTTEAVELGGITLAPGTSVTLCIGAANRDPAQFDDPERFDITRIANRHLAFGTGAHQCAGMAL
ARLEGAVAIARFVSRFPNYQLAGEPVRGGRVRFRGFASVPCVVS

>CYP195A12(Rpdx1_1566)*Rhodopseudomonas palustris* DX-1

METAPAELAEAFDLARLTPDFYDNPYPTYQALRTHQPVKRLPGGGYFLTRYDDLISVYKNTTLFSSDKKREFA
PKYGESLLFEHHTTSLVFNDPPSHTRVRRLIMGALTPRAIAGMEPDLIALVDRLLDAMASKDKVDLIEDFAAA
IPIEVIGNLLGVPQDERAPLRGWSLAILGALEPVIGPEAFDRGNEAVREFLGYLETLIARRTAEPGDPERDVL
TRLILGEAGGEKLSAKELLHNCIFLLNAGHETTTNLIGNGLVTLADNPAQKQRLIAEPGLIRTAVEEILRYES
SNQLGNRITTAQVEIGGVTMPANTSLTLCIGAANRDPAQFADPDRFDVARSPNRHLAFASGPHQCAGMALARL
EGAIALSRFLARFPDYALDGAPQRGGRVRFRGYLSVPCRLG

>CYP195A13(RPB_3652)*Rhodopseudomonas palustris* HaA2

MEMPSRELAAEFELERLTPEFYDNPYPTYRALQTHQPVKRLRNGGYILTRYDDLVTVYKNTTLFSSDKKREFA
PKYGDSLLFEHHTTSLVFNDPPAHTRVRRLITGALSPRAIAGMQPDLIALVDRLLDAMAAKAGVDLIEDFAAA
IPIEVIGNLLGVPHDERGPLRDWSLAILGALEPVIGPETFSRGNEAVRDFLAYLEILITRRRAEPGDPEHDVL
TRLIQGDDGTGEKLSAKELLHNCIFLLNAGHETTTNLIGNGLVALADNPAEKQRLIGQPGLARTAVEEILRYE
SSNQLGNRITTTEVEIGGVTMQANTSLTLCIGAANRDPAQFPDPDRFDVGRTPNRHLAFATGPHQCAGMALAR
LEGVIALTRFLARFPNYTLDGTPSRGGRVRFRGYLRVPCRL

>CYP195A14(RPD_1807)*Rhodopseudomonas palustris* BisB5

METAPIELAAAFDLERLTRDFYDDPYPTYHALRTHEPIKRLRNGGYFLTRYDDLVEVYKNTTLFSSDKKREFA
PKYGDSLLFEHHTTSLVFNDPPAHTRVRRLITGALSPRAIAGMEPDLIALVDRLLDRMEAKGTLDLIEDFAAA
IPIEVIGNLLGIPHDERGPLRDWSLAILGALEPVIGPDTFARGNDSIRDFIAYLETLIARRRANPGDPERDVL
TKLIQGDGGGEQLSARELLHNCIFLLNAGHETTTNLIGNGLVALAVHPAEKQRLIAQPGLVRTAVEEILRYES
SNQLGNRITTADVEIGGVAMPANTSLTLCIGAANRDPEHFPDPDRFDVARSPNRHLAFATGPHQCAGMALARL
EGAIALRRFLARFPNYALAGAPTRGGRVRFRGYLQAPCRI

>CYP195A15(M446_2531)*Methylobacterium* sp. 4-46

MSAVDAAERARRFDLRRLPEGFIEDPYPVYAALRTHAPVHVFAPGQVLLTRYVDLERVYKDAATFSSDKVVEF
GAKFGPSPLYAHHTTSLVFNDPPRHTRVRRIIAGALAPRAIASMEASIVALVDGLLDAAEERGRIDLIEDFAA
AIPVEVIGNLLGVPRAEREPLRAWSLAILGALEPVLDAATMEAGNRAVTEFLDYLKRLVAERRRRLGDPERDI
LTRLIQGEMEGERLSPEELLQNCIFILNAGHETTTNLIGNGLHLLTQWPEARERLLSEPDLLRRAVEEMLRFE
SSNQLGNRIAAKPFALGGRDFPAGTQITLGIGAANRDPDQFPDPDRFDVAREPNRHLAFASGIHQCVGMTVAR
LEGRIAIGRFLARFPSYALDGPPLRSRRVRFRGFSRLPARVEA

>CYP195A16(SPO1622)*Ruegeria pomeroyi*

MILQPPGPGERRGAARQFTGRNVMSETVMQTQIGKLDLTAPPPGFLENPFPFYDALLAHAPVLAQPDGSVLLS
RHADLDRIYRDTTLYSSDKKAAFGPKFGVGSPLFEHHTTSLVFNDPPLHTRVRKIMTSALTPRAIARMEPGLF
ETVDHLLEAMAGRDRVDLIADFASTIPIQIIGNLLDVPMDERGPLRDWSLIILGALEPKLSAEQLAQGQQAVT
EFKTYLEDLVARRRARPGDPETDVLTRLIRGEGADQQLSEIELLQNCIFILNAGHETTSNLIGNGLALLHDYP
EERARLLADPSLLPSAVEEILRFRSPNQLGNRETTAEIELDGRRLPRGTNLHLCIGAANRDPAVFDDPTRFDI
ARKPNRHLAFAGGPHVCVGLTLARLEGRIALGRLLERFPDYCLLEGRVPGGRIRFHGYAELPARL

>CYP195A17(Mnod_1189)*Methylobacterium nodulans*

MTDAALARSFDLRRLPDGFCDDPYPVYAALRQHAPVHVFAEGHLLLSRYADLERVYKDAATFSSDKTVEFGAK
YGPSPLYDHHTTSLVFNDPPRHTRVRRIIAGALTPRAIASMEAGIVALVDGLLDAAAARGRIDLIEDFAAAIP
VEVIGNLLGVPRAERGPLRAWSLAILGALEPVLDPEAEAAGNRAVTEFLAYLDGLVAERRRRPGDPDKDILTR
LIRGEPGGERLAPEELLQNCIFILNAGHETTTNLIGNGLHLLTLWPQARERLRAEPDLIRKAVEEMLRFESSN
QLGNRIAAKPFTMGGRAFPAGTRITLCIGAANRDPEQFPDPDRFDIARDPNRHLAFASGIHQCVGMNVARLEG
RIAIGRFLARFPAYALDGPPLRSQRVRFRGFLRMPARLA

>CYP195A18(RHPLAN_24330)*Rhodoplanes* sp. Z2-YC6860

MAIVADMAADFDINRLDRAFLDDPYPTYRALRERDPVHRMPDGSYFLTRYDDLVAVYHDTKTWSSDKTVQFKP
KFGDSALYEHHTTSLVFNDPPIHTRVRKLLAPAFTPRALKALQPRIELLVDRLLDHAAEHGQIDLIEDFAAAI
PVQLIGDMLGIPQDERGPLRDWSLSILGALEPVLSEQQLGGGMTAVEEFKAFLRDLVARRQREGAQDPGEILS
TLIGATDLGGTEPSGDKLSELELLHNCIFLLNAGHETTTNLIGNGIDLLRRHPDVMRDLAAHPQTIETAIEEF
LRMESSNQLGNRRAAADTRIGETAMPAGTYVHLCIGAANRDPAQFPEPDTLDIRRTPNRHLAFGFGIHTCAGN
SLARMEAQVAIGKLVRRFKSIECTSEPDRGGRARFRGFLHYPVSVK

>CYP195A19(METH_10630)*Leisingera methylohalidivorans*

MSGGQHGFVNIEEFDLANPPEGFLEDPFPFYDALLRGAPVLPQPDGSLLVCRHADLDRIYRDTTLFSSDKKKV
FAPKFGAGSPLFEHHTTSLVFSDPPLHTRVRRIMTSALTPRALARMEPGLVETVDHLLDRMEGAAKVDLIEDF

AASIPIQIIGNLLDVPMAERAPLRDWSLAILGALEPALKADQLAAGHRAVEAFKAYLQDLIARRRARPGNIET
DVLTRLINGEGADGTLSEVELIQNCIFILNAGHETTTNLIGNGLALLNDHPDQKQRVLEQPDLIKPAVEEVLR
FRSPNQLGNRETTAEVEIGGMKVPAGTNLHLLIGAANRDPEVFDKPGDFDAGRTPNRHLAFAGGPHVCVGLTL
ARLEGRIAIERLLHRFPGYRLLTGRVPGGRIRFRGYAALPAEMG

>CYP195A20(Maq22A_c22575)*Methylobacterium aquaticum*

MSDPALAEGFDLRRLPDGFIANPYPVYAALREHAPVHRLGEGQILLSRYADLERVYKDAATFSSDKTVEFGAK
FGAGSGAGEAPSPLYRHHTTSLVFNDPPRHTRVRRIIAGALTPRAVAAMEAGIVALVDDLLDRAAARGTIDLI
EDFAAAIPVEVIGNLLGVPRDERGPLRDWSLAILGALEPVLPPEVEADGNRAVTAFLAYLDRLVDDRRRHPGD
PDKDILTRLIQGEVGGERLSPRSCCRTASSS

>CYP195D1(Jann_0677)*Jannaschia* sp. CCS1

MLDIAQFDLNQIDAAFIEDPYSTLAALRRTSPVHPNADGSVYLTRHDDCLATYRSRDMLSDKTEAFGQKFGEC
PLKVHHTTSLIFNDPPYHTVVRKLIAGAFTPRKLKEMERAIERIVSRLLDRVEDLGELDVIADFGMMLPTEII
SIMLGVPEAYRAQLRGYSTAILGALDPVVSSSQMEAGNRAVEEFGAVLDDLINHRRANPDAAAEGEVLESLIF
GEHDGRTLTQEELVQNCIFLLNAGHETTTSLVGNAVAVLLANPSEHRRLIDDPTLIGTAVEEILRVESPLQIG
NRLAGEDIALPSGPTIPKGTYIHTSIAAANRDPEVFADPDRFDVSRKPNPHIAFITGIHVCLGASLARMEGKI
AIGGFVKRFPKLAATGEGERLGLARFRGWTSLPIRVR

>CYP195E1(SL003B_4100)*Polymorphum gilvum*

MTVSTAADEVLSGQARGFNLFDPPEDYFDDPGPYFRLLRDGDPLHAQEDGSVLLTRYDDVRQVWRDLSGLVYK
GDQFRQRFGEGPLLEHHTSTMLFRDNPDHDRLRNIVNPFFAQTSIQQLSRFTQDVVDQEIEKVKRMGEFDFVR
DFAFRIPVTLICKIIGVPVEDADYIQAVGRKVLFPLNPKVPDSAIAEGHDAVAEFKTYLLPFLQEIKSRPSID
RAENILCAMAAAQREGVEISDDEILHMCIVTLNGGHETTTNLISQSIHFLMDDPESLKQLRDGEVPIATALEE
LIRFVTPLQLQGRRTTRDVVLDSGNGTIPAGTEVVLCQASANRDERIFDSPDRLNLARRPNNHVGFGAGIHVC
LGRSLARLEASIAIPTVLRELPTLARGGDVRFQRNTRFRGLEQFPVRIA

>CYP195E2(TQ29_17135)*Confluentimicrobium* sp. EMB200-NS6

MTEAARIDQTDSEKAQAFNLLAPPDDYFDDPSKYFRLLRDGDPVHRQADGTVLLTRYDDVRQVWRDLSGLVYK
GEYFEKKFGKGPLLEHHTSTMLFRDNPDHDRLRDIVNPFFSQTSIKSLTDFTQDVIDREIKKVKQMGEFDFVR
DFAFRIPVALICRVIGIPVEDADYIQGVGRKVLFPLNPQVSAEAIKQGHEAVTEFMGYLLPFLDEIKARRTID
PSESILCAMAAAQRNGVEVSDAEILHMCIVTLNGGHETTTNLISQSMHFLMDDPESKRQLVEGEVALPTALEE
LIRFITPLQLQGRRTTNDVELASGNGTIPAGTEVVLAQASANRDERVFEDPDRLNFARRPNNHVAFGAGIHVC
LGRPLARMEAPIALASVLKELPTLVRNGEARFQHNTRFRGLEELPVRLA

>CYP196A1(bll0894)*Bradyrhizobium diazoefficiens* USDA 110

MLPRSAGAGRNKVAVARCPFGQPTGALVMSMQNVAAPALQFTAPRRNELTHIPGDEGWPVIGKTFQVLADPKG
HIEANGAKYGPVYRTHVFGETNVVLLGPEANELVMFDQQKLFSSTHGWNKVLGLLFPRGLMLLDFDEHRLHRK
ALSVAFKSGPMKSYLSDLDRGISARVAQWKAKPGEMQLYPAMKQLTLDLAAASFLGADIGPEVDEINRAFVDM
VAAAVAPIRRPLPGTQMARGVAGRKRIVAYFRQQIPLRRGNHGGDDLFSQLCRATHEDGALLSEQDIIDHMSF
LMMAAHDTLTSSLTSFIGELAANPDWQDRLRAEVLALGLAPGAPSSFDDLEKMPLSEMAFKEALRIKPPVPSM
PRRAMRDFTFKGFRIPAGTAVGVNPLYTHHMKDIWPEPDRFDPLRFTEEAQRNRHRFAWVPFGGGAHMCLGLH
FAYMQAKCFARHFLQNIEVSLAPGYKPDWQMWPIPKPRDGLKVRVKAV

>CYP196A1(S23_69120)*Bradyrhizobium* sp. S23321

MSLQNVAAPALQFTAPKRNELTHIPGDEGWPVIGKTFQVLADPKGHIEANAAKYGPVYRTHVFGETNVVLLGP
EANELVMFDQQKLFSSTHGWNKVLGLLFPRGLMLLDFDEHRLHRKALSVAFKSGPMKSYLSDLDSGIAARVAQ
WKANPGEMQLYPAMKQLTLDLAAASFLGADIGPEVDEINRAFVDMVAAAVAPIRRPLPGTQMARGVAGRKRIV
AYFRQQIPLRRGNHGGDDLFSQLCRATHEDGALLSEQDIIDHMSFLMMAAHDTLTSSLTSFVGELAANPDWQD
RLRAEVLALGLAPGAPSSFDDLEKMPLSEMAFKEALRIKPPVPSMPRRAMRDFTFRGFTIPAGTAVGVNPLYT
HHMKEIWPEPDKFDPLRFTEEAQRNRHRFAWVPFGGGAHMCLGLHFAYMQAKCFARHFLQNIEVSLEPGYKPD
WQMWPIPKPRDGLRVRMKAV

>CYP196A1(BCCGELA001_35185)*Bradyrhizobium* sp. CCGE-LA001

MSMQNVAASALQFTAPKRNELTHIPGDEGWPIIGKTFQVLADPKGHIERNGAKYGLVYRTHIFGETNIVLLGP
EANELVLFDQQKLFSSTHGWNKVLGLLFPRGLMLLDFDEHRLHRKALSVAFKSGPMKSYLSDLDRGISARVAQ
WKTKQGEMLLYPAMKQLTLDLAAASFLGADIGPEVDEINRAFVDMVAAAVAPVRRPLPGTQMARGVKGRKRIV
AYFREQIPLRRGNHGGDDLFSQLCRATHEDGALLSEQDIIDHMSFLMMAAHDTLTSSLTSFIGELAANPDWQD
RLRAEVLALGLAPDAPSSFDDLEKMPLTEMAFKEALRLKPPVPSMPRRAMRDFSFKGFAIPAGTAVGINPLYT
HHMKEIWPEPDRFDPLRFTEDAQRNRHRFAFVPFGGGAHMCLGLHFAYMQAKCFARHFLQNIEVSLEPGYKPD
WQMWPIPKPKDGLRVRLKAV

>CYP196A2(RPA0785)*Rhodopseudomonas palustris* CGA009

MSIQVADSSLVARLSPPKPSALAHVPGDEGWPIIGRTLAVLADPKGEVEKMARTYGPVYRSRVLGETSITLLG
PEANELVLFDNTKLFSSTHGWGPILGRLFPRGLMMLDFDEHRLHRRTLSVAFKAGPMQSYLAELNAGIAHRVA
EWRARPGEMLCYPAMKQLTLDLAATSFLGTAIGAETEEVNRAFIDMVAASVAPIRKPWPGTAMARGVKGRQRI
VAYFAEQIPIRRAKGGDDLFSQLCRATHDDGALLSNQAIIDHMSFLMMAAHDTLTSSLTSFVAALAAHPEWQQ
KLREEIAGLGLKPGEPISFEQLDALPLTEMAFKEAMRLRPPVPSLPRRATRAFSFKGYTIPAGTMVAVNPLFT
HHMPEIWPNPDQFDPLRFTDEASRGRHRFAWIPYGGGAHMCLGLNFAYMQAKCFAVHLLQHLDLSLPPNYQAS
WQMWPIPKPKDGLRVNVAPLN

>CYP196A2(Rpal_0853)*Rhodopseudomonas palustris* TIE-1

MSIQVADSSLVARLSPPKPSALAHVPGDEGWPIIGRTLAVLADPKGEVEKMARTYGPVYRSRVLGETSITLLG
PEANELVLFDNTKLFSSTHGWGPILGRLFPRGLMMLDFDEHRLHRRTLSVAFKAGPMQSYLAELNAGIARRVA
EWRARPGEMLCYPAMKQLTLDLAATSFLGTAIGAETEEVNRAFIDMVAASVAPIRKPWPGTAMARGVKGRQRI
VAYFAEQIPIRRAKGGDDLFSQLCRATHDDGALLSNQAIIDHMSFLMMAAHDTLTSSLTSFVAALAAHPEWQQ
KLREEIAGLGLKPGEPISFEQLDALPLTEMAFKEAMRLRPPVPSLPRRATRAFSFKGYTIPAGTMVAVNPLFT
HHMPEIWPNPDQFDPLRFTDDASRGRHRFAWIPYGGGAHMCLGLNFAYMQAKCFAVHLLQHLDLSLPANYQAS
WQMWPIPKPKDGLRVNVAPLN

>CYP196A2(Rpdx1_0945)*Rhodopseudomonas palustris* DX-1

MSIQVADSSLVARPVPPKRSALAHIPGDEGWPIIGRTLAVLADPKGEVEKMGRTYGPVYRSRVLGETSITLLG
PEANELVLFDNTKLFSSTHGWGPILGRLFPRGLMMLDFDEHRLHRRTLSVAFKAGPMQSYLAELNAGIARRIA
EWRSRPGEMLCYPAMKQLTLDLAATSFLGTAIGAETEEVNRAFIDMVAASVAPIRKPWPGTAMARGVKGRQRI
VAYFAEQIPIRRAKGGDDLFSQLCRATHDDGALLSNQAIIDHMSFLMMAAHDTLTSSLTSFVAALAAHPEWQQ
KLREEIAGLGLKPGEPIGFEQLDALPLTEMAFKEAMRLRPPVPSLPRRATRAFSFKGYTIPAGTMVAVNPLYT
HHMPEIWPTPDRFDPLRFTDDASRGRHRFAWIPYGGGAHMCLGLNFAYMQAKCFAVHLLQNLDLSLPPNYQTA
WQMWPIPKPKDGLRVQVAPAK

>CYP196A3(Saro_3337)*Novosphingobium aromaticivorans*

MASIAPDSRTDLHTERANPHWVRLGGDHKLDHVPGEDGWPVLGTTLMQLADPLGFQRRMVETHGPVFRTRSFG
RRGVNLIGADANELVLFDRDRLFSNEQGWGPVLNLLFPRGLMLMDFEAHRVDRRALSIAFKPEPMRAYCSVLN
TGIAQAVQGWGGQMRFYDAIKALTLDTAASSFLGLPLGPEADRLNKAFVDMVQASGGVVRRPLPFTRMGKGVA
GRRLMVEYFGRLVRERRADPGQDMFSQFALATREDGSLLPEDVVVDHMIFLMMAAHDTITSSATVLFWQLARN
PDWQDRLRAEARAVTGGDGLPLAYEDLGRMELTEMAFKEALRFMPPVPNMPRRALRDFEFGGYRIPAGTPVGI
SPAAVHADPAHWPEPDRFDPLRFTPENVSGRHKYAWVPFGGGAHMCLGLHFAYMQVKLLVSHILTRYEVAMQP
GPAPSWQAWPIPKPRDGLRVEMRRIC

>CYP196A4(BJ6T_09060)*Bradyrhizobium japonicum* USDA 6

MSMQNVAPALPFIAPKRNELTHIPGDEGWPIIGKTFQVLADPKGHVEANGAKYGPVYRTHIFGETNVVLLGPE
ANELVLFDQQKLFSSTHGWNKVLGLLFPRGLMLLDFDEHRLHRKALSVAFKSGPMKSYLSDLDRGIAARVAQW
KAKPGEMKLYPAMKQLTLDLAAASFLGADIGPEVDEINRAFVDMVAAAVAPIRRPLPGTQMAAGVKGRKRIVA
YFREQIPLRRGNHGGDDLFSQLCRATHEDGALLSEQDIIDHMSFLMMAAHDTLTSSLTSFIGELAANPDWQDR
LRAEVLALGLAPGSPSSFDDLEKMPLSEMAFKEALRIKPPVPSMPRRAMRDFTFKGFTIPAGTAVGVNPLYTH

HMKDIWPEPDRFDPLRFTEEAQRNRHRFAWVPFGGGAHMCLGLHFAYMQAKCFARHFLQNIEVSLEPGYKPEW
QMWPIPKPRDGLRVRVKAV

>CYP196A4(RN69_04425)*Bradyrhizobium japonicum* E109

MSMQNVAPALPFIAPKRNELTHIPGDEGWPIIGKTFQVLADPKGHVEANGAKYGPVYRTHIFGETNVVLLGPE
ANELVLFDQQKLFSSTHGWNKVLGLLFPRGLMLLDFDEHRLHRKALSVAFKSGPMKSYLSDLDRGIAARVAQW
KAKPGEMKLYPAMKQLTLDLAAASFLGADIGPEVDEINRAFVDMVAAAVAPIRRPLPGTQMAAGVKGRKRIVA
YFREQIPLRRGNHGGDDLFSQLCRATHEDGALLSEQDIIDHMSFLMMAAHDTLTSSLTSFIGELAANPDWQDR
LRAEVLALGLAPGSPSSFDDLEKMPLSEMAFKEALRIKPPVPSMPRRAMRDFTFKGFTIPAGTAVGVNPLYTH
HMKDIWPEPDRFDPLRFTEEAQRNRHRFAWVPFGGGAHMCLGLHFAYMQAKCFARHFLQNIEVSLEPGYKPEW
QMWPIPKPRDGLRVRVKAV

>CYP196A7(LMTR13_01240)*Bradyrhizobium icense*

MSMQSIASPVYSFTPPRRNSLTHIPGDEGWPLIGKTLEVLADPKGQVERQSKKYGLVYRSHLFGETSLVLLGP
EANELVLFDQAKQFSSTLGWGRILGLLFPRGLMLLDFEEHRLHRRALSVAFKSGPMKSYLADLDTGIAARVAQ
WKAQPGPMLLYPAMKQLTLDLAATSFLGAGIGPEVDEITRAFVDMVAASVAVIRKPLPGTQMARGVKGRKRIV
AYFSEQIPIRRAKGGGDDLFSQLCQATHEDGALLSTQDIVDHMSFLMMAAHDTLTSSLTSFVGELAAHPEWQQ
KLRDEVASLGAAADAPTTFDNLEAMKVTEMAFKEALRLKPPVPSMPRRAIRDFTFKGYAIPAGTMVGINPLFT
HHMPEIWPEPEKFDPTRFTDEAQRNRHRFAWVPYGGGAHMCLGLHFAYMQAKCFARHFLQNLEVSLEPGYKPD
WQMWPIPKPRDGLRVLVKPV

>CYP196A8(BRADO0504)*Bradyrhizobium* sp. ORS 278

MVSEPIRLTSPRRNSLTHIPGDEGWPIIGKTFEVLADPKGHVERNARKYGPVYRSHMFGEVNVVLLGPEANEL
VLFDQAKLFSSAHGWGHILNLLFPRGLMLLDFEEHRMHRKALSVAFKAGPMKSYLSGLDHGIAARIAQWRERP
GEMLFYPAIKQLTLDLAATSFLGTEIGPEVDTINRAFIDMVAAAVAPIRKPLPGTQMARGVKGRQRIVAYFSE
QIPLRRARSDGDDLFSHLCRATDDEGALLSTQDIVDHMSFLMMAAHDTLTSSLTSFVAQLAANPAWQQKLRAE
VYSLGLAKDEPMTSEHLEKMKLTEMAFQETLRLMPPVPSLPRRPVRDFTFKGFKIPAGTGVGVNPMYTHHMPE
IWPEPQRFDPLRFTDEAQRGRHRFAWVPFGGGAHMCLGLHFAYMQAKCFARHFLSNVEVSLAPGYQPSWQVWP
IPKPRDGLKVQLKAV

>CYP196A9(BBta_7679)*Bradyrhizobium* sp. BTAi1

MVSDPIRLTAPRRNSLTHIPGDEGWPIIGKTFEVLADPKGHVERNARKYGPVYRSHMFGEVNVVLLGPEANEL
VLFDQAKLFSSAHGWGHILNLLFPRGLMLLDFEEHRMHRKALSVAFKAGPMKSYLTGLDRGIAARIAQWKQTP
GEMLFYPAIKQLTLDLAATSFLGTEIGPEVDAINRAFVDMVAASVAPIRKPLPGTQMARGVKGRQRIVAYFSE
QIPLRRAKGDGDDLFSHLCRATDDHGALLSTQDIVDHMSFLMMAAHDTLTSSITSFIAQLAAHPEWQQKLRDE
VASLGLVAGAPMASEHLEKMKLTEMAFQETLRLMPPVPSLPRRPIRDFTFKGYAIPAGTGVGVNPMFTHHMPE
IWPEPERFDPLRFTDEAQRGRHRFAWVPFGGGAHMCLGLHFAYMQVKCFARHFLSNIEVSFAPGYRPNWQVWP
IPKPRDGLRVMLKAV

>CYP196A10(S58_71560)*Bradyrhizobium oligotrophicum*

MSMQHVVSDAIRLTPPRRNSLTHIPGDEGWPIIGKTFDVLADPKGHVERNARKYGPVYRTHMFGDVNVVLLGP
EANELVLFDQAKLFSSAHGWGHILNLLFPRGLMLLDFDEHRMHRKALSVAFKAGPMKSYLAGLDRGIAARIAQ
WKQAPGEMPFYPAIKQLTLDLAATSFLGSDIGPEVDDINRAFIDMVAAAVAPIRKPWPGTQMARGVRGRQRVV
AYFSEQIPLRRAKGDGDDLFSHLCRATDEQGALLSTQDIVDHMSFLMMAAHDTLTSSLTSFVAQLAAHPQWQQ
KLRAEVASLGLANGDPMSSEHLEQMKLTEMAFQETLRLMPPVPSLPRRPIRDFTFKGYAIPAGTGVGINPMFT
HHMPEIWPEPETFDPMRFTDEAQRGRHRFAWVPFGGGAHMCLGLHFAYMQAKCFARHFLSNIEVSFAPGYQPS
WQVWPIPKPRDGLKVILKPV

>CYP196A11(LH19_03345)*Sphingopyxis macrogoltabida* 203

MNAPTKINWPEARFGPETQHWLPRNPDAALDHIPGEDGMPVIGNTLDQLRDYPAFTRRMVATYGRVYRNNSFG
GRSVALHGPEANELVMFDRDKIFSSEQGWGPVLNLLFPRGLMLMDFEKHRADRKLLSVAFKPEPMRHYADSLN
EGIARRVAEWSGKSFKFYPAIKELTLDLAATSFLGIPWGPEADKVNKAFVDMVQASIGIVRVPLPFTAMGRGA

RGRAYLVDYFGRMVPERREGSGEDMFSQICRTKDDNGDYMSVEAIVDHMNFLMMAAHDTITSSVTSLVYMLGK
HPEWQDRLREEMLAVAPAGEGVGHNSLGELEMTEWAFKEALRLVPPVPSFPRRALKDFEYGGYRIPAGTSVGV
SPAFTHMMEEHWPEPEKFDPMRFSPEVARERHKYAWVPFGGGAHMCLGLHFAYMQAKIFFHHVVTTHRIVLPD
GYAPDWQVLPIPRPKDGLTVTFAPL

>CYP196A12(LH20_02570)*Sphingopyxis* sp. 113P3

MNAPARIEWSEARFGPDTQHWLPRNPDSALDHIPGEDGLPILGNTLDQLRDYPAFTRRMVAKYGHVYRNNSFG
GRSVSLHGPDANELVMFDREKIFSSEQGWGPVLNLLFPRGLMLMDFEKHRADRKTLSVAFKPEPMRHYAESLN
EGIRHRVAAWSGSTFKFYPAIKELTLDLAATSFLGIPWGPEADKVNKAFVDMVQASIGVVRVPLPFTAMGRGA
KGRAYLVDYFSRMVPDRREGSGADIFSQICRALDDNGDHLPVDAIVDHMNFLMMAAHDTITSSITTLVWQLAD
HPEWQDRLREEMLRVAPAGEGVGHNSLGELELTEWAFKEALRLVPPVPSFPRRALKDFEFGGYRIPAGTSVGV
SPAFTHMMEEHWPEPEKFDPMRFSPEVARERHKYAWVPFGGGAHMCLGLHFAYMQAKIFFHHVLTTHRIVVAQ
GYEPEWQILPIPRPKDGLVVTFQPL

>CYP196A13(SKP52_05010)*Sphingopyxis fribergensis*

MNAPTNIQPVSSQWSEKRFGPDTLHWLPRNSDSAIDHIPGEDGMPVIGNTLEQLGDYRGFTNRMVAKYGRVYR
NNSFGGRSVALHGPEANELVMFDRDKIFSSEQGWGPVLNLLFPRGLMLMDFEKHRADRKILSVAFKPEPMRHY
AESLNEGIRDRIGKWSGKTFKFYPAIKELTLDLAATSFLGIPWGPEADKVNKAFVDMVQASIGVVRVPLPFTA
MGRGAKGRAYLVEYFGKMVPERRDGTGEDMFSQICRTRDDNGDHMSVEAIVDHMNFLMMAAHDTITSSITSMV
WELAKHPEWQDKLREEMLAVSPAGEGVGHNSLGQLEMTEWAFKEALRLVPPVPSFPRRALKDFEYGGYRIPAG
TSVGVSPAYTHMMAEHWPDPEKFDPMRFSPEVARERHKYAWVPFGGGAHMCLGLHFAYMQAKIFFHHVVTTHR
IVVAEGYAPEWQVLPIPRPKDGLTVSFVPLA

>CYP196A14(Sala_0663)*Sphingopyxis alaskensis*

MNAPTQIHPASSRWSAARFGPDTQHWLPRNPDSAIDHIPGEDGLPIIGNTLEQLRDYPAFARRMVAKYGPVYR
NNSFGGRSVALYGPEANELVMFDRDKIFSSEQGWGPVLNLLFPRGLMLMDFEKHRADRKLLSVAFKPEPMRHY
ADALNDGIRGRVASWSGRTFQFYPAIKELTLDLAATSFLGIPWGPEADKVNKAFVDMVQASIGIVRRPLPFTA
MGRGAKGRAFLVDYFSRMVPERREGAGEDMFSQICRTRDDNGDYMPVDAIVDHMNFLMMAAHDTITSSITTLV
WELARHPDWQDRLREEMLAVAPAGEGVGHNSLGELELTEWAFKEALRLVPPVPSFPRRALKDFEFGGYRIPAG
TSVGVSPAFTHMMEEHWPEPERFDPMRFSPEVARGRHKYAWVPFGGGAHMCLGLHFAYMQAKIFFHHVVTTHR
ISVAPGYAPEWQMLPIPRPKDGLTVTFEPLK

>CYP196A15(AOA14_12370)*Sphingopyxis terrae*

MNAPTTIEWSERRFGPETAHWMPRNAETALDHIPGDDGIPVLGTTLAQLKDPVGFTNRMVAQHGKVYRAKSFG
GRGVSLVGPEANELVLFDREKIFSSEQGWGPVLNLLFPRGLMLMDFEKHRADRKTLSVAFKPEPMRHYAGALN
EGIGSRIGAWSGKSFKFYPAIKQLTLDLAATSFLGIPWGPEAERINKAFVDMVQASIGIIRVPMPFTAMGRGV
AGRKFLVDYFTRLVPERRASEGEDIFTQICHAKDDDGEYLSVEAIVDHMNFLMMAAHDTITSSITSLVWLLAR
HPEWQDRLRDEMLSVAPAGEGVGHNSLGELELTEMAFKEALRLIPPVPAIPRRALRDFEFGGYHIPAGTNVGI
NPSYTHMMAEYWPDPERFDPLRFAPEAVRGRHKYAWVPFGGGAHMCLGLHFAYMQAKIFFHHVLTTHRIRIAD
GYAPDWHMWPIPRPKDGLTVTFDPL

>CYP196A16(SGRAN_1363)*Sphingopyxis granuli*

MNAPANSMWSERRFGPDTAHWLPRGPESALDHIPGDDGIPLLGTTLAQLRDPVGFTNRMVDRHGKVYRARSFG
GRGVSLVGPEANELVLFDRDKLFSSEQGWGPVLNLLFPRGLMLMDFDKHRADRRTLSVAFKPEPMRLYADALN
EGIRDRVARWSGTRFAFYPAIKQLTLDLAATSFLGIPWGPEAERINKAFVDMVQASVGVVRVPLPFTAMGRGV
AGRKFLVEYFTKMVPERRASTGDDIFTQICQAKDEEGEYLSVEAIVDHMNFLMMAAHDTITSSVTSLVWLLGR
HPEWQDRLREEMLRVAPAGEGVGHNSLGELELTEWAFKEALRMIPPVPVIPRRALRDFEFGGYRIPAGTNVGV
SPSYTHMMDEYWPDPERFDPMRFAPDAAKGRHKYAWVPFGGGAHMCLGLHFAHMQAKIFFHHVLTTHRIEIAD
GYAPDWQMWPIPRPKDGLTVTLTPL

>CYP196A17(BSY18_484)*Blastomonas* sp. RAC04

MATVLAPQAPAAPREMFPDHSIQRDSGNPHWTPLPGSNALDHIPGEDGLPILGVTLRMLADPEGFGNEMRAKY
GPVYRTNAFGRRQVALFGADANELVLFDRDKVFSSEQGWGPVLNLLFPRGLMLMDFDHHRADRRALSIAFKPA
PMRHYAESLNEGIGARVLQWSNKDITFYSEIKKLTLDLAATSFLGIPWGPEADKINTAFVDMVQASIGVVRRP
LPFTKMGKGVAGRKFLVDYFTPQVLQRREQQGPDMFSQFCRATRDDGSLLTTDEVVDHMNFLMMAAHDTITSS
ASSLIWLLAKNPEWQDKIRAELLEHAPAGAPMPYDALPNLELTEMAFKEALRLIPPVPSTPRRALKDFEFGGY
RIPAGTPVSISAAYTHKQEEHWPEPHRFDPLRFTPEATRNRHKYAWVPFGGGAHMCLGLHFAYMQIKVLLHHV
LSQHRIVIEPGYEPGWQAWPIPQPKDGLKVRFETL

>CYP196A18(LPB140_11945)*Sphingopyxis* sp. LPB0140

MSYAHSEEANPHWVRPPSLKDIEHIPGETGLPLIGNTFAVLADPIGFGNKMQANYGKIYRNRAFGQNTVMLLG
PDANELVLFDRNKLFSNEQGWGPVLNLLFPRGLMLMDFDHHRMDRKALSIAFKPEPMRAYCTALSQGIYRRIA
DWPNDLQFYPAIKQLTLDLAATSFLGIDWGPEADKINTAFVDMVAASVGVARTPIPFTKMARGVAGRKYLIDY
FTPQIAERRRNENMQDMFSQFCRATREDGEYMADDEIVDHMNFLMMAAHDTITSSITSMVWLLAKNPDWQEKL
REECLSIAPAGGAIAYEDLSKFTLTEMAFKESLRLIPPVPSIPRRAVKDFEFGGYHIPAGTAVGVAPSFVHHM
AEYWPEPEKFDPMRFTPEAIKERHKFAWVPFGGGAHMCLGLHFAYEQMKIFMHHLLTSRSIEVNNGYEPKWQA
WPIPKPKDGLHVTLRPL

>CYP196A19(AZE99_13075)*Sphingorhabdus* sp. M41

MATIAEKPHGYPYQESANPHWVKPPSQEQLAHIPGEQGLPIVGTTLKVIKDPIGFGQYMFQRYGSVYRTYSFG
KQNVMMLGADATELVMFNKDKIFSSEQGWGPVLNNLFPRGLMLMDFERHRADRKALSVAFKPGPMKHHCEVLG
TGILDRVGDWSGTEFEFYPAIKSLSLDTAASAILGIPWGPEADKINEAFVDEVQASIGVVRKPIPFTKMWKGV
KAREYLLEYFTPQVKERRAKGGEDIFTQICLATHENGELLTEDEVVDHMNFLMMAAHDTITSSATSMTYLLAK
HPEWQDRLREECLSVAPAGTVLTIEQLGKLQLTEMAFKEALRMIPPVPSIPRRALKDFTFRNYTIPAGTNIGI
SPQFVHKMEKHWPDPDKFDPLRFTPENSAGRHKYAWVPFGGGAHMCLGLHFAYMQIKILMHAMLTQHRVVLQG
GPDYEPNWQVFPIPQPRDGLPVRLERL

>CYP196A20(A6F68_00151)*Altererythrobacter dongtanensis*

MATLAEPPIRTNPTDEIPPANWRVGHPDDAALAHIPGERGLPLVGNTFRLLADPIAFGWRMYRTYGPVYKNWA
LGGWHVALIGAEANEMILMDRDKIFSSEQGWGPTLHRLFPRGLMLMDFEHHRVDRRALSIAFKPEPMRHYAAA
LDRGIASEVAGWTGDMLFYPAIKQLTLDLAAESFLGGGLGPDAPKINQAFVDMVQASVAPIRRPLPFTQMKRG
VDGRSYLVDLFTRETHRRRAGEGLGQDMFSQFATAIREDGSLLPVDAVVDHMNFLMMAAHDTITSSATSLVWL
LAKNPEWQERLRQEILAVTGGPDASGTPRGAAYDDLGKLELVEMAFKEALRLIPPVPSLPRRALRSFEYKGFR
IPAGTEVGFNVYLTHRLEEHWGDDVEAFDPLRFTPEKVKARHKYAWVPFGGGAHMCLGLHFAYMQVKILMAQL
LQRYRIEVADGYAPEWQPWPIPKPKDGLKITLKPL

>CYP196A21(RPB_4636)*Rhodopseudomonas palustris* HaA2

MSIQVADSSLVATLAPPPRSALAHIPGDEGWPIIGRTLHVLADPKGQVEMMGRLYGPVYRSRVLGETSITLLG
PEANELVLFDNTKLFSSTHGWGSILGLLFPRGLMMLDFDEHRLHRKALSVAFKAGPMQSYLAELNSGIARQVA
QWRAQPGEMLCYPAMKQLTLDLAATSFLGTAIGAETAEVNAAFVDMVAASVAPIRKPWPGTAMARGVRGRQRI
VAYFSEQIPIRRAEGGDDLFSHLCRATHDDGALLSTQDIVDHMSFLMMAAHDTLTSSLTSFVAALAAAPEWQR
RLCEEIGGLGLKPGEPIAFEQLDALPLTEMAFKEAMRLRPPVPSLPRRATREFSFKGYTIPAGTMVAINPLYT
HHMPEIWPAPDRFDPLRFTDEAQRGRHRFAWVAYGGGAHMCLGLNFAYMQAKCFAVHLLQNLSLDLPPNYQSS
WQMWPIPKPKDGLRVRIAPLQ

>CYP196A22(RPD_0772)*Rhodopseudomonas palustris* BisB5

MSIQVADSSLVATPIAPPRSALAHIPGDEGWPIIGRTLEVLADPKGQVQMMGRTYGPVYRSRVLGETSITLLG
PEANELVLFDNTKLFSSTHGWGSILGLLFPRGLMMLDFEEHRLHRRALSVAFKAGPMQSYLAELNSGIARRVA
QWRAQPGEMLCYPEMKRLTLDLAATSFLGTAIGAETAEINAAFVDMVAASVAPIRKPWPGTAMARGVKGRKRI
VAYFSEQIPIRRAKGGDDLFSQLCRATHEDGALLSTQDIIDHMSFLMMAAHDTLTSSLTSFVAALAADPEWQQ
RLRAEIAALGLKPGEPISFEQLDALPLTEMAFKEAMRLRPPVPSLPRRATREFSFRGFAIPAGTMIAINPLHT
HHMPEIWPDPDRFDPLRFTDEAQRGRHRFAWIPYGGGAHMCLGLNFAYMQAKCFAVHLLQNLSLSLPPNYQSS
WQMWPIPKPKDGLRVNVTPLH

>CYP196A23(RPE_0895)*Rhodopseudomonas palustris* BisA53

MSLQFADSSAGSMAPPKRSALSHIPGDEGWPMVGRTFQVLADPKGEVERMAATYGPVYRSRVLGETSITLLGP
EANELVLFDQAKNFSSTHGWGPILGRLFPRGLMMLDFDEHRLHRRALSVAFKSGPMQSYLAQLDSGIAARVKQ
WRGKPGEMLCYPAMKQLTLDLAATSFLGGDLGAEVDEITKAFVDMVAAAVAPIRKPWPGTAMARGVRGRQRIV
AYFSEQIPIRRAKGGDDLFSQLCQATHEDGALLSTQDIIDHMSFLMMAAHDTLTSSLTSFVALLAANPEWQQK
LRDEVHSLGIGPNEALPFAKLDEMPLTEMAFKEAMRLKPPVPSIPRRAVRDFSFRGFTIPAGTMVAVNPIYCH
HMAQVWPEPDKFDPLRFTEEETRKRHKFAWVPFGGGAHMCLGLHFAYMQAKCFTRHFLQNLSVSLEPGYQPQW
QLWPIPKPKDGLKVQIAPAR

>CYP196A24(RPC_4693)*Rhodopseudomonas palustris* BisB18

MSLQIADGSGRPSLAPPKRGALNHIPGDEGWPIIGRTLQVLADPKGEVERMAATYGLAYRSRVLGETSITLLG
PEANELVLFDQAKNFSSTHGWGPILGLLFPRGLMMLDFDEHRLHRRALSVAFKAGPMQSYLAALDAGIATRVA
QWRNEPGEMLFYPAMKQLTLDLAATSFLGAEIGPEVDDITRAFVDMVAAAVAPIRRPWPGTAMAKGVRGRRRI
VGYFSEQIPIRRAKGGDDLFSQLCQATHEDGALLSTQDIIDHMSFLMMAAHDTLTSSLTSFVALLAANPEWQR
KLRDEVAALDVPDGQTLPFDKLDAMPLTEMAFKEAMRLKPPVPSIPRRAVRDFSFQGFAIPAGTLVAVNPLFS
HHMPQIWPEPEKFDPLRFTEEAQKARHRFAFVPFGGGAHMCLGLHFAYMQAKCFTRHFVQNLEVSLAPGYAPE
WQMWPIPKPKDGLKLTLTPRRD

>CYP196A25(AM2010_2618)*Altererythrobacter marensis*

MATLAQESTPGTAQPAPPPRHWRERTLTEADLAHIPGEGGLPFVGNTFRMLADPHAFTRRMVETYGPVYRNKA
FGGWNIALVGADANELVLFDREKLFSSEQGWGPILDRLFPRGLMLMDFDHHRADRRALSIAFKPGPMRHYAGS
LNRGIAQSVKNWGGAPMQFYPAIKALTLDLAADSFIGIPWGPEADRINEAFVDMVQASVAPVRRPLPFTLMRK
GVTGREFLVEYFTRETHRRRAEGGGQDMFSQFATATREDDTLLPVDEVVDHMNFLMMAAHDTITSSATTLVQM
LARHPEWQERLRAEIVALTGGGGDVDYDDLGKFELTEMAFKEALRMIPPVPSTPRRALRAFEFGGYRIPAGAP
VGINAQYVHHMEEHWPDPERFDPMRFTPDQVKARHKYAWVPFGGGAHMCLGLHFAYMQVKILMAQLLTRYEIV
TEAGYSPDWQAWPIPRPKDGLKVTFRPL

>CYP196A26(A9D12_06330)*Porphyrobacter neustonensis*

MATLAQRPTEPAAPPAHWGEGRLTDADLAHIPGDAGWPVVGNTFTMLANPHAFAERMIKTHGKVYKNRAFGGW
QIALIGAEANELLLFNKDKIFSSEQGWGPVLDQLFPRGLMLMDFDHHRIDRRALSIAFKPEPMRHYSGALNRG
IAREVAGWAGPQEFYPAIKKLTLDLAADSFIGLPWGPEADRINEAFVDMVQASVAPVRKPLPFTKMKKGVDGR
AFLVDYFTRETLRRRAEGGGQDMFSQFATATREDGSLLPVDEVVDHMNFLMMAAHDTITSSATSLIYHLATNP
EWQEKLRAEIFAVTGGPDGEGNPRPLDYDDLAKLDLTEMAFKESLRMIPPVPSMPRRALREFEYGGYRIPAGA
MVGINIYWTHHSEEYWDNPHVFDPMRFTPDKVKARHKYAWVPFGGGAHMCLGLHFAYMQVKILLAQLLQRYRI
EAPAGYNPDWQDWPIPQPKDGLKVTFTPL

>CYP196A26(BG023_11724)*Porphyrobacter* sp. LM 6

MATLAEAPTARDAAPADAPRHWGEGRLTEADLAHIPGEAGWPVVGNTFTMLANPHAFADRMIKTHGKVYKNRA
FGGWQIALIGAEANELLLFNKDKIFSSEQGWGPVLDQLFPRGLMLIDFEHHRIDRRALSIAFKPEPMRHYSGA
LNRGIAREVAGWAGPMEFYPAIKKLTLDLAADSFIGLPWGPEADKINEAFVDMVQASVAPVRKPLPFTKMKKG
VDGRAFLVEYFTRETLRRRAEGGGQDMFSQFATATREDGSLLPVDEVVDHMNFLMMAAHDTITSSATSLIYHL
ATNPEWQEKLREEIMAVTGGPDGDGNPRPLDYDDLAKLDLTEMAFKESLRMVPPVPSMPRRALREFEYGGYRI
PAGAMVGINIYWTHHSEEYWDKPFTFDPMRFTPDKVKARHKYAWVPFGGGAHMCLGLHFAYMQVKILLAQLLQ
RYRIEAAPGYNPEWQDWPIPQPKDGLKVTFKPL

>CYP196A27(ELI_11310)*Erythrobacter litoralis* HTCC2594

MPDVADIAHIPGEKGWPIVGHTFTQLRDPHAFTRRMVETYGKVYRVHSFGGWNVALIGAEANELVLFNKDKIF
SSEQGWGPVLDQLFPRGLMLMDFDHHRIDRRALSIAFKPGPMRHYAGALNSGIAREVAKWDGTMLFQPAIKQL
TLDLAADSFLGIPWGPEADKINEAFVDMVQASVAPVRKPLPFTKMKKGVDGRAFMVDFLTRETNRRRAEGGGQ
DMFSQFATATREDGSLLPVDQVVDHMSFLMMAAHDTITSSATSMIHKLAQYPEWQEKMREEIFAVTGGPDGSG
TPRNLEYDDLGKLELTEMVFKETLRSIPPVPSMPRRALKTFEFGGYRIPAGQHVGINIHYTHHSEEYWDDPFA

FDPMRFTPELVKARHKYAWVPFGGGAHMCLGLHFAYMQIKVLMTHLLQRYRIVESEGYDPEWQAWPIPQPKDG
LKIELKTL

>CYP196A28(Ga0102493_11527)*Erythrobacter litoralis* DSM 8509

MATLAHPHAGADARSFDHWSEGRIDDSDLSHIPGEAGWPVVGNTFRMLADPHGFARRMIDTYGKVYKNRAFGG
WQVALIGAEANELLLFDRNKMFSSEQGWGPILDQLFPRGLMLIDFEHHRVDRRALSIAFKPEPMRHYSGALNS
GISREVESWEGPMTFYTAIKKLTLDLAADSFIGIPWGPEADRINEAFVDMVQASVAPVRKPLPFTKMKKGVDG
RAYLIDYFTRETHRRRAEGGGQDMFSQFATATREDGSLLPVDEVVDHMNFLMMAAHDTITSSATSLIYYLAKN
PEWQEKLREEVFAVTGGPDGSGKARALDYDDLGKLELTEMAFKEALRMVPPVPSMPRRALREFEYEGYRIPAG
TMVGINIYWTHHSEEYWEDPFTFDPMRFTPDKVKARHKYAWVPFGGGAHMCLGLHFAYMQVKILIAQLLQRYR
IEVADGYAPAWQEWPIPQPKDGLKVEFVKL

>CYP196A29(WG74_05250)*Citromicrobium* sp. JL477

MATLAAHQPANVRPQHWKDANPTKADLSHIPGEQGLPVVGNTLKMLADPVGFTKGMVEKYGRVYRNNAFGGTV
VALIGADANELVLFDRNKIFSSEQGWGPILDKLFPRGLMLMDFDHHRADRKALSIAFKPEPMRHYVGSLNRGI
SERMEEWGAGEMKFYPAIKQLTLDLAADSFIGIPFGAEADKVNQAFVDMVQASVAPIRQSLPFTKMKRGTDGR
AYLVEYFTKETQRRRAEGGGQDMFSQFATATRDNGELLPVDEVVDHMNFLMMAAHDTITSSATSLVYYLARSP
EWQDKLREELRAITGGEGRALNYEELAKAELTEMAFKEALRMIPPVPTIPRRALEAFEFGGYHIPAGTPVGVN
PTFVHNDPEIWDSPETFDPMRFTREAEKARHKYAWVPFGGGAHMCLGLHFAYMQIKILMAHMLTRYRIEVAED
YAPDWQAWPIPQPKDGLQIRLVRI

>CYP196A30(AMC99_02233)*Altererythrobacter epoxidivorans*

MATLAAHENAEFRPTHWSQRLPADALDHIPGEAGLPIVGNTFRMLADPHGFAQEMVAKYGKVYKGRAFGGWQV
ALIGAEANELLLFDRDKIFSSEQGWGPILDKLFPRGLMLIDFDHHRADRRALSIAFKPGPMRHYSGSLNAGIA
REIAKWADREMRFYPAIKQLTLDLAADSFIGIPWGPEADKINTAFVDMVQASVAPIRAPLPFTQMKKGVDGRK
YLVDYFTRETHRRRAEGGGQDMFSQFATATHEDGSLLPVDEVVDHMIFLMMAAHDTITSSATSLIYLLAKNPD
WQNKLRQEVISVTGGVDASGKPRPLSYDDLGKLELTEMAFKEALRMIPPVPSMPRRALKSFEYGGYRIPAGTS
VGINIHMVHHMEEYWPEPEKFDPMRFTPEQVKARHKYAWVPFGGGAHMCLGLHFAYMQVKILMAQLLPQYSIE
IAEGYDPKWQPWPIPKPKDGLKVAFRRL

>CYP196B1(RD1_3506)*Roseobacter denitrificans*

MPPLRPFKARSNMPTRAALSHIPGPPTPPLIGHTLKIARDSYGTQQDYIKRYGPVYKTKMLGVWRVNLCGPDA
LQHVLLDKDKIFSSTGGWDALRRIYPGGLILQDFDKHRQDRRILTAAFRASAIRDYRMRMGEIMLDLLQHWPK
DAPFDFYEAIKDLTLRMGGAVFMGLPLDGDLAKQINRAITDEIRASVTPIRTAIPFTPMWRGIRGRDFLRETF
RKMIPERRARGGDDFFSQMCMAKDEDGKGWSEDEILNHFNLLIMAAHDTTATSLTVIMAALGTHGDWQQRLID
EVDGLGDGDLDEEALGQMKETDKVFREALRLVPPVPFIPRMATRDFHWHGYDIPAGTSLALNPGVTMLSPELF
SNPTQFDPDRFAPDRAEDRIHRFAWTPFGGGAHKCIGMHFATMQVKLFIATLLRQRRIELPGGAPEWHRMPIP
KPKGGLPVLLPAR

>CYP196B1(RLO149_c027090)*Roseobacter litoralis*

MPTRAALSHIPGPPTPPLIGHTLKIAKDSYGTQQEYIKRYGSVYKTKMLGVWRVNLCGPDALEHVLLDKEKIF
SSAGGWDALRRIYPGGLILQDFDKHRQDRRILTAAFRASAIRDYRVRMGAIMLDLLQHWPKDAPFDFYEAIKD
LTLRMGGAVFMGLPLDGDLAKQINRAITDEIRASVTPIRAPIPFTPMWHGVRGRDFLRETFRKLIPERRKNGG
DDFFSQMCMATDEDGKGWSEDEILNHFNLLIMAAHDTTATSLTVIMAALGTHGEWQQRLIDEVDALGDAELNE
DALGQMKETDKVFREALRLVPPVPFIPRMTTQDFHWQGYDIPAGTSLALNPGVTMLSPELFTNPTQFDPDRFA
PDRAEDRIHRFAWTPFGGGAHKCIGMHFATMQVKLFIATLLRQRKIELPGGPPEWHRMPIPKPKGGLPILLPA
R

>CYP196C1(RGUI_0121)*Rhodovulum* sp. P5

MSYPIPDLSHIPAMPRLPVVGNSLEVVRDPYGFHQKARDRLGPVYRFHFLGRDVVCAHGAEVLEQALLNREGR
FSNVLGWQVIEDLFSGGLMLRDGADHRAHRRMLQPAFRADVMRNALTLLVDRLQAAIASWPVGQPFSFYTEFR
GLARAIAAEVFMGITDPAEADRIGRAFLDMLDASAAPIRRPLPFTRMRRGIRARRELRDIFEGLIDERRTGKG

RDLFSELCRLKTEDGDWIEKDVLVDHFNFFLFAAFDTATTSISAMVDQLSQHPDWQEAMAAEVATLDGDLSLE
ALDRLDVTERVLKEAIRLMPPVPFLPRGVVDGFEHDGHRLPKGTPLTVCPGLVMIDPEIWSDPLAFDPDRFSS
NRAEDQRHAYAWAPFGGGIHKCLGMNFAIMEAKAFFACLLPRYRIAPRAPAKWRRLPIPSPTDGLQVTLRPAS
A

>CYP197R1(METDI0881)*Methylobacterium extorquens* DM4

MLQDSNPASHLDRPVHTDASPPGPKGWPLLGSLIGFARDPLGFLTACSRQYGDVVAFRTAGQLMLVLTCPREI
ERVLVKEHQNFPKNEHFWRQVTALFGNGLLTSKGTFWQRQRRLAAPAFAGPMLASYADAMVHESMLTAEGWRA
GEVRDVHADMMALSLRIAAKALFGTEVEEDVLVIDDALNNILAELASRVVRPILIPDAVPLPGHVRYRRALCR
IDWIVARIINERRTLSAAGHDFLSALMGARDEDGTAMSDRQLRDEVITFLLAGHETTALVLSWTIHLLSRHPE
VDRVLAAEIGEIVVQRAAAIDDVPRLRFAEHTITESMRLYPPAWAVGREARCDCQIGGFDVRPGTPILISPWV
LHRDPRFFDEPEAFRPERWHQGLAQRLPRFAYMPFGGGPRICIGNRFAMIEAVLILTTLVQRFRFVADTNEPV
VPIPSMTLRPRGGVRVIIERREINSPYG

>CYP197R1(Mext_0777)*Methylobacterium extorquens* PA1

MLQDSNPASPLDRPVHTDASPPGPKGWPLLGSLIGFARDPLGFLTACSRQYGDVVAFRTAGQLMLVLTCPREI
ERVLVKEHQNFPKNEHFWRQVTALFGNGLLTSKGTFWQRQRRLAAPAFAGPMLASYADAMVHESMLTAEGWRA
GEVRDVHADMMALSLRIAAKALFGTEVEEDVLVIDDALNNILAELASRVVRPILIPDAVPLPGHVRYRRALCR
IDWIVARIINERRTLSAAGHDFLSALMGARDEDGTAMSDRQLRDEVITFLLAGHETTALVLSWTIHLLSRHPE
VDRVLATEIGEIVVQRAAAIDDVPRLRFAEHTITESMRLYPPAWAVGREARCDCQIGGFDVRGGTPILISLWV
LHRDPRFFDEPEAFRPERWHQGLAQRLPRFAYMPFGGGPRICIGNRFAMIEAVLILTTLVQRFRFVADTNEPV
VPIPSMTLRPRGGVRVIIERREISSPYG

>CYP199A1(blr1048)*Bradyrhizobium diazoefficiens* USDA 110

MSAPGSAASGVPHLDVDPFDMNFFADPYAAHELLREAGPVVYLDKWNVYGVARYAEVHAVLNDPATFCSSRGV
GLSDFKKETPWRPPSLILEADPPAHTRTRAVLSKVLSPTVMKQVRDRFAAAAEERVDALIEKRSFDAIADLAE
AYPLSIFPDALGLKSEGREHLIPYASVVFNAFGPPNQLRQEAIARSTPHQAYVAEQCQRENLAPGGFGACIHA
QVDEGAITASEAPLLVRSLLSAGLDTTVNGIGAAVYCLARFPEQWQRLRGDLSLARSAFEEAVRFESPVQTFF
RTTTREVELSGATIGEGEKVLMFLAAANRDPRRWDKPDSYDVTRRSSGHVGFGSGIHMCVGQLVARLEGEVML
TALARRIAKIEITGEPKRRFNNTLRGLDGLPVTITPA

>CYP199A1(RN69_05235)*Bradyrhizobium japonicum* E109

MSAPGSAVSGVPHLDVDPFDMNFFTDPYSTHERLREAGPVVYLDKWNVYGVARYAEVHAVLNDPATFCSSRGV
GLSDFKKETPWRPPSLILEADPPAHTRTRAVLSKVLSPTVIKQVRDRFAAAAEERVDALIERRSFDAIADLAE
AYPLSIFPDALGLKSEGREHLIPYASVVFNAFGPPNELRREAIARSTPHQAYVAEQCQRENLAPGGFGACIHA
QVDEGAITATEAPLLVRSLLSAGLDTTVNGIGAAVYCLARFPEQWQRLRSDLTLARSAFEEAVRFESPVQTFF
RTTTREVELSGATIGDGEKVLMFLAAANRDPRRWDKPDSYDITRRSSGHVGFGSGIHMCVGQLVARLEGEVML
TALARRIEKIEITGEPKRRFNNTLRGLDSLPVTITPA

>CYP199A2(RPA1871)*Rhodopseudomonas palustris* CGA009

MTTAPSLVPVTTPSQHGAGVPHLGIDPFALDYFADPYPEQETLREAGPVVYLDKWNVYGVARYAEVYAVLNDP
LTFCSSRGVGLSDFKKEKPWRPPSLILEADPPAHTRTRAVLSKVLSPATMKRLRDGFAAAADAKIDELLARGG
NIDAIADLAEAYPLSVFPDAMGLKQEGRENLLPYAGLVFNAFGPPNELRQSAIERSAPHQAYVAEQCQRPNLA
PGGFGACIHAFSDTGEITPEEAPLLVRSLLSAGLDTTVNGIAAAVYCLARFPDEFARLRADPSLARNAFEEAV
RFESPVQTFFRTTTRDVELAGATIGEGEKVLMFLGSANRDPRRWDDPDRYDITRKTSGHVGFGSGVHMCVGQL
VARLEGEVVLAALARKVAAIEIAGPLKRRFNNTLRGLESLPIQLTPA

>CYP199A2(Rpal_2077)*Rhodopseudomonas palustris* TIE-1

MTTAPSLVPVTTPSQHGAGVPHLGIDPFALDYFADPYPEQETLREAGPVVYLDKWNVYGVARYAEVYAVLNDP
LTFCSSRGVGLSDFKKEKPWRPPSLILEADPPAHTRTRAVLSKVLSPATMKRLRDGFAAAADAKIDELLARGG
NIDAIADLAEAYPLSVFPDAMGLKQEGRENLLPYAGLVFNAFGPPNELRQSAIERSAPHQAYVAEQCQRPNLA
PGGFGACIHAFSDTGEITPEEAPLLVRSLLSAGLDTTVNGIAAAVYCLARFPDEFARLRADPSLARNAFEEAV

RFESPVQTFFRTTTRDVELAGATIGEGEKVLMFLGSANRDPRRWDDPDRYDITRKTSGHVGFGSGVHMCVGQL
VARLEGEVVLAALARKVAAIEIAGPLKRRFNNTLRGLESLPIQLTPA

>CYP199A2(Rpdx1_3652)*Rhodopseudomonas palustris* DX-1

MTTAPSSVPVTTPSQHGAGVPHLAIDPFALDYFADPYPEQETLREAGPVVYLDKWNVYGVARYVEVYAVLNDP
LTFCSSRGVGLSDFKKEKPWRPPSLILEADPPAHTRTRAVLSKVLSPATMKRLRDGFAAAADAKIDELLARGG
SIDAIADLAEAYPLSVFPDAMGLKQEGRENLLPYAGLVFNAFGPPNELRQSAIERSAPHQAYVAEQCQRPNLA
PGGFGACIHAFSDTGEITPEEAPLLVRSLLSAGLDTTVNGIAAAVYCLARFPDEFARLRADPSLARNAFEEAV
RFESPVQTFFRTTTRDVELAGATIGEGEKVLMFLGSANRDPRRWDDPDRYDITRKTSGHVGFGSGVHMCVGQL
VARLEGEVVLAALARKVAAIEIAGPLKRRFNNTLRGLESLPIQLTPA

>CYP199A4(BRADO6810)*Bradyrhizobium* sp. ORS 278

MNPTALSSAAVASAIPRLAIDPFSYGFFDDPYPAHEQMREAGPVVYLDQWNVYGVARYAEVYTVFNDPQTFCS
SRGVGLSDFAKEKPWRPPSLILEADPPAHTRTRAVLSKVLSPAVMKRLRDGFMAAAEAKIDDLVARGSFDAIP
DLAEAYPLSVFPDALGLKAEGRDHLISYASLVFNAFGPPNELRQQAIERSAPHQAYVTEQCQRDNLAPGGFGA
CIHAFSDSGEITPAEAPLLVRSLLSAGLDTTVYGIGAAVYCLARFPDQWARLRADPSLARNAFEEAVRFESPV
QTFFRTTTRDIELGGVTIPGGEKVLMFLGSANRDPRRWEQPDSYDITRKVSGHVGFGSGIHMCVGQLVARLEG
EAVLTALARKVGSITMSGAPKRRYNNTLRGLESLPVTFTPA

>CYP199A26(S23_67580)*Bradyrhizobium* sp. S23321

MSAPGFATSGVPHVDVDPFDMNFFADPYPAHELLRETGPVVYLDKWKLYGVARYAEVHAVLNDPATFCSSRGV
GLSDFKKETPWRPPSLILEADPPEHTRTRAVLSKVLSPTVMKGLRDRFAAVAEQRVDALLDKGSFDAITDLAE
AYPLSIFPDALGLKPEGREHLIPYASVVFNAFGPPNQLRQEAIARSAPHQAYVTEQCQRDNLTPGGIGACIHA
HVDEGAITATEAPLLVRSLLSAGLDTTVNGIGAAVYCLARFPEQWQRLRGDLTLARNAFEEAVRFESPVQTFF
RTTTREVELSGATIGEGEKVLMFLAAANRDPRRWDKPDSYDITRRTSGHVGYGSGIHMCVGQLVARLEGETML
SALARRVASIEITGEPKRRFNNTLRGLDSLPVTITPA

>CYP199A27(Swit_3269)*Sphingomonas wittichii*

MASHAERSPYAIYDSDPPPGVPVLDVDPFSVDFFADPYPVHEQLREAGPFVWLSRYNIGAVARHEHVRDALTD
WKTFSSARGVGMEDFVRHGRFRLPSLILEADPPQHSRSRGVLSRVLSPPVLKGLRDRFEVEAEAMVEAVVRRK
RFDGVADLAEAFPLRVFPDAIGMGREGREKLLPHADLLFNSFGPRNELFQAAKEKVSFDWIEEQGKRGNLAPG
GLGLMIHDAVDRGEISEAEAPVLVRALLQAGLDTTINALGATLYCLARFPSEFDRLRADHKLAKAAFEEAIRF
EAPVQTFFRTATRATTLGGTPLNEGDKVLMFLAAANRDPRQWDRPDVYDIGRRTIGHVGFGAGIHACVGQLLA
RMEGELVLTALARRVKTLRIAGTVERRYNNTLRGLASLPLEIELA

>CYP199A28(RPB_3613)*Rhodopseudomonas palustris* HaA2

MISNSSAESISAPPNDSTIPHLAIDPFSLDFFDDPYPDQQTLRDAGPVVYLDKWNVYGVARYAEVHAVLNDPT
TFCSSRGVGLSDFKKEKPWRPPSLILEADPPAHTRPRAVLSKVLSPATMKTIRDGFAAAADAKVDELLQRGCI
DAIADLAEAYPLSVFPDAMGLKQEGREHLLPYAGLVFNAFGPPNELRQTAIERSAPHQAYVNEQCQRPNLAPG
GFGACIHAFTDTGEITPDEAPLLVRSLLSAGLDTTVNGIGAAVYCLARFPGELQRLRSDPTLARNAFEEAVRF
ESPVQTFFRTTTREVELGGAVIGEGEKVLMFLGSANRDPRRWSDPDLYDITRKTSGHVGFGSGVHMCVGQLVA
RLEGEVMLSALARKVAAIDIDGPVKRRFNNTLRGLESLPVKLTPA

>CYP199A29(BBta_0729)*Bradyrhizobium* sp. BTAi1

MNPPGLSAGAAGTSPPAIPHLGIDPFAISFFDDPYPAHQQMREAAPVVYLDRWNVYGVARYAEVYAVLNDPLT
FCSSRGVGLSDFAKETPWRPPSLILEADPPAHTRTRAVLSKVLSPAVMKRLRDGFMAAAEAKVDELVARGSFD
AIPDLAEAYPLSVFPDALGLQPEGREHLIPYASLVFNAFGPPNELRQQAIERSAPHQAYVTAQCQRDNLTPGG
FGACIHAFSDSGEITPAEAPLLVRSLLSAGLDTTVYGIGAAVYCLARFPDQWARLRADPALARNAFEEAVRFE
SPVQTFFRTTTRDVELGGVVIPEGEKVLMFLGAANRDPRRWERPDSYDITRKVSGHVGFGSGIHMCVGQLVAR
LEGEAVLTALARKVGAITMSDEPKRRYNNTLRGLESLPVTFTPA

>CYP199A30(S58_06520)*Bradyrhizobium oligotrophicum*

MNPTGLSASPAGAPAIPHLAIDPFSYGFFDDPYPAHHEMREAGPVVYLDTWNVYGVARYAEVYAVFNDPQTFC
SSRGVGLSDFTKEKPWRPASLILEADPPAHTRTRAVLSKVLSPAVMKRLRDGFMAAAEAKVDELVARGAFDAI
PDLAEAYPLSVFPDALGLQPEGREHLIPYASLVFNAFGPPNELRQQAIERSAPHQAYVTAQCQRDNLTPGGFG
ACIHAFSDTGEITPAEAPLLVRSLLSAGLDTTVYGVGAAVYCLARFPDQWARLRADPSLARNAFEEAVRFESP
VQTFFRTTTRDVELGGVVIPEGEKVLMFLGAANRDPRRWEQPDSYDITRKVSGHVGFGSGIHMCVGQLVARLE
GEAVLTALARKVGSITMSAEPVRRYNNTLRGLASLPVTFTPA

>CYP199B2(Avi_7683)*Agrobacterium vitis*

MIEGHRVFEPAEGIPVWDIDPYDEAVLADPNAYYAELRAKGPLVYIPKYSILASGRYEETKEIFSDHARFVSS
RGVGLNDFRYGTPWRPPSIILEVDPPEHARTRKVMARALSPKAISKFKEDFRVKAVEIVDALLEKRRFEAVVE
LAEAFPTAVFPEALGMVDHDPRPLVDYGAMVFNSMGPDNALRRKAGAMIPEIVPWIMSACARSRLRPDGMGAI
IYEAADAGEITQEEAGLLIRSFLSAGVDTTVTGIGNALWCLASNPAEYERLKVDPSLARPCFEEVLRYTSPVH
TFGRTAAVDTEIAGLKIAEGSKIICVLGAANLDPEKWEAPESFRIDRRPAGHMAFGAGIHGCVGQNIARLELE
TLLTVMAAKVDRIEFDGEVVWRPNNAIHALDRMPLEFIAK

>CYP199J1(Sphch_3292)*Sphingobium chlorophenolicum*

MCNMCQKSDECSLRQQKVSNLSDQAPPSFDIDPFDNAYLRDPYAWHGFLRQAAPVIHLSKYDCYAVGRYDEVK
EAMSNHEDFSSADGTGLGSLSKGTAQRSRSPIIEVDPPEHNAVRRPMNNILSPRLVKEWTDAFAASAASVVDA
ALAKDSFDIIGEIVEPFVLEAFPDFLGIGKEGRENFLLIGDFVLNSLGPDNELYRKTAKAAEPVLGWMMSKYD
RGAMAPGRLGALIWKGVDEGKIDPSIAETLIRTFLRGGTDTVISGLGTLLAQLVLRPDQWEILKNDRSKMSTA
IDEAIRLESPAQSMFRNTRRQVKLGGYVLEADKKILCSLGAANRDPDRWRNPDQFDFNRSVQGHLGFGVGVHF
CIGQRLARAETEAMLGALLDRVDSFEAVGEPEWRMNNVARRLDNFRVSATRLAECASS

>CYP199K1(RN69_13925)*Bradyrhizobium japonicum* E109

MTGIACSQADPFAPDFLIDPYPAYETLRALGPVFRLARYDVWAMAGYAGVEAALKDWKTFISGEGVGLNGMNP
ALPKPLTLQIDPPDHDKGRRVLGRTLSPGVARKLRETFQQEAERKVSELIDKGTFDAVADLAEAYPMKVFPDA
IGIRPDGREKLLAWSTFVFDSFGPENEMLAASRKAGLAAQSWIMECCARDALQPDGLGMMIYQAADDGEITEH
EATHLVRPFLTAGIDTTVNGIGNTLLALATHPDEYRKLHQRPELARNAFEEGLRYDSPVQTFFRTTSREVETS
GGVIPAHRKVLLFMASANRDPARWDNADRFEVERSATGHVGFGAGIHACVGQMIARLEGELIFGELARRVKTI
ELTAEPRRRLNNSLRGLESMPVRVTAA

>CYP199L1(EP837_03852)*Sphingobium* sp. EP60837

MSLPASAPAGVPTSEIDLFAPKVLEDPHRFDGELREIGPVVWLEKHGVYAVTRHKAIQSMYADWRNFHNFGSP
FNPDTLIPAVIIIDNPPQHTRLRSAIMPYVSPAALARYRKDFETTAQLLIEQILDEREFDARDLAASFVLKAF
PDLLGLPLEGRHFLLNFGEAILNTLGPMNDLTIKSLERAGPAFAWVQSECSREKVLPGKLASKVYALTETADI
SEEEAGLLVRTLLAAGFDTTILGITAAFYGFTQNPDQWQLLSDPKMVRAAFEEGIRYFPPNRFGGRLVAADTE
FEGAHFKSGDRITTLLAAAGRDPRRWDDADVFDITRPARSHLSFGHGIHACLGQVLSRTEYDVLITALFKHVA
RIEAAGPAERAVNNVALGWGKVPIRLTLR

>CYP199M1(Ga0080574_TMP462)*Pelagibaca abyssi*

MTTTDIPALDFDPFSVESIAAGPTVFAEIREMAPLVWLKPYGVYATCRHEFAQKALRDWQSFTSTVKAFGERE
HIPNIMVQEDPPDHSAHRNPVMKFFTPVALNNYRAFFEQSAADHADRLVETGAVDGFDDVASGYILQVFPDIL
GMKEMDRKQLLLFGDLAFNSTVPVNDLYKACKARSGDVLEWFNRQCQRESVTRDGLAEEIYRLGDSGEVSEQS
AQLLVRAVFSGGFDTTVLSITSGLKLFAENPDQWDLLREEPKLARNAFEEVIRMEPPSRFLGRGVAAETELAG
VPLKPGDKFATFLGGVGRDPRQWQDPDRFDIRRKKVLGHMSFGHGVHSCLGQGLARIEFASLFTALAKRVKRI
EITGEIKRNINNQANGFHVLPLRLHAG

>CYP200A1(blr3832)*Bradyrhizobium diazoefficiens* USDA 110

MAPRLDFTSEAFFRDPPAAIAALRASGPVVATRFPLVGDVWITTTHDATAEVLKDGTTFTLRKEDGKVAGLRW
WMPKLVTTIANNMLTMDEPDHTRLRSIVDEAFRRRAIVAMEPRIRAIADGLANDLFADGSPADLVQCYARILP
VSVICELLGLPAADRPRFIAWANKMSSLTNVVSFFRLLFAFRKMRAYLERQLQIARVRGGEGLIAELVQVELE

GGQITPDEMVSMVFLLLAAGSETTTHLISGSVYELLRNPALRDWLEEDWSRISLAVEEFLRFVSPVQFSKPRY
LRRDVELAGVRLKKGDRVMVMLAAANMDPAVHDRPERLDLTRKPNRHMSFGTGIHFCLGHQLARIEATCALQA
LLARWPKLELAVDPAQIHWRKRPGMRAIARLPVVAGGNRRPSRGAAAEPLLAD

>CYP200A1(BJ6T_60220)*Bradyrhizobium japonicum* USDA 6

MTPRLDFTSEAFFRDPPEAIAALRASGPVVATRFPLVGDVWITTTHDATAQVLKDGTTFTLRKGDGKVAGLRW
WMPKLVTTIANNMLTMDEPDHTRLRSIVDEAFRRRAIVAMEPRIRAIADGLANDLFADGSPADLVQRYARILP
VSVICELLGLPAADRPRFIAWANKMSSLTNVVSFFRLLLAFRKMRAYLEQQLQVARVQGGEGLIAELVQVERE
GGQITPDEMVSMVFLLLAAGSETTTHLISGSVYELLRNPGLRDWLEQDRSRIGLAVEEFLRFVSPVQFSKPRY
VRQDIELAGVPLKKGDRVMVMLAAANMDPAVHDQPERPDLTRKPNRHISFGTGIHFCLGHQLARIEAACALEA
LFARWPKLGLAVDPAQIHWRKRPGMRAIAELPVVPVDSQPVDFRDATTSRSQPNTERPSRSGEASRSPAM

>CYP200A1(RN69_29175)*Bradyrhizobium japonicum* E109

MTPRLDFTSEAFFRDPPEAIAALRASGPVVATRFPLVGDVWITTTHDATAQVLKDGTTFTLRKGDGKVAGLRW
WMPKLVTTIANNMLTMDEPDHTRLRSIVDEAFRRRAIVAMEPRIRAIADGLANDLFADGSPADLVQRYARILP
VSVICELLGLPAADRPRFIAWANKMSSLTNVVSFFRLLLAFRKMRAYLEQQLQVARVQGGEGLIAELVQVERE
GGQITPDEMVSMVFLLLAAGSETTTHLISGSVYELLRNPGLRDWLEQDRSRIGLAVEEFLRFVSPVQFSKPRY
VRQDIELAGVPLKKGDRVMVMLAAANMDPAVHDQPERPDLTRKPNRHISFGTGIHFCLGHQLARIEAACALEA
LFARWPKLGLAVDPAQIHWRKRPGMRAIAELPVVPVDSQPVDFRDATTSRSQPNTERPSRSGEASRSPAM

>CYP200A3(BF49_1210)*Bradyrhizobium* sp. BF49

MAPRLDFTSEAFFRDPPKAIATLRMSGPVVAVRFPLVGHVWITTTHEATAQVLKDGTNFTLRKEDGDVAGLRW
WMPNYVKTIANNMLTMDEPDHTRLRSIVDEAFRRRAIVAMEPRIRAIADGLADELFSAGSPADLVQRYARILP
LAVISELLGLPLADRPRFIAWANTMSSLTNVASFFRLLFAFRKMRAYLERQLQIARVRGGEGLIAELVQVERE
GGQITPDEMVSMVFLLLAAGSETTTHLISGSVYELLRNPSVRDWLAQDWSRLGLAAEEFLRFVSPVQFSKPRY
VRRDIELAGVRLKEGDRVMVMLAAANMDPAMHDHPDSLDLAREPNRHMSFGTGIHFCLGHQLARIEAACALEA
LFVRWPKLGFAVDVSQIHWRKRPGLRAIANLPVVPDGHGAAGSMMVHSQPAAC

>CYP200A4(HYPDE_32213)*Hyphomicrobium denitrificans* 1NES1

MKIDITNQEFFRDTAAAVAPLRAMGPVAEIHFPIVGKVWITTTYELAGRVLKDSETFTLRKNGGPVAGLRWWM
PRIVRAVSNNMLTMDEPDHTRLREIVDEAFRRRAILDMEPRIRAITDTLANELFADGSPADVVDRYSRRIPLA
VICEVLGLPEADRPRFMAWSNSFTRLTNLMGFFGLIPAMVSMKRYLEGQLDVARKNGGEGLIAELVRVEKEGG
RISGDEMVSMVFLLLGAGTETTTHLISGSVFELLRNSERRDWLEEDWSRINLAIEEFLRFVSPVQFSKPRYIR
RDIELGGVRLPAGDKIMAMIVAANMDPAESEHPEKLDLDRRPNRHLAFGTGIHFCLGHQLARIEGKCALEALL
TRWPKLELAVPETEIRWRARPGIRAIASLPVISR

>CYP200B1(USDA257_c38530)*Sinorhizobium fredii* USDA 257

MIEPHDFDVLSPEFHANPFPTLDRMRAEGAVVRMKLPIVGRTWFAVTHDTCAALLKDHESFARDPANAGSRTQ
ARILKLLPRTIGLLALNMIGYDDPEHRRLRGLVDQAFQRRSIEAMKPMITQIADRLLDRLDGKREVDLMSEFC
RDLPLSVICAVLGLPEQDHDRFKRWLGGLKDTANIGAIIRAVPGVVRVVRYLRRVSRAGCRALPDGLVAALRD
AETDGQTLSEDELVSMIFLLFGAGQETTTHLISGGLFALLSHEDELLRLQNDPSLMPTCVEECLRYVSPVQMT
KPRFARRDMRWQGQQFRRGDMIAAFLAAANCDPAKFEHPHRFDITRHPNPHLSFGTGVHFCLGFQLARAEAAI
AFERILTRFPEMSLNSDLSNIKWRKRLGIRALAQLPVKLAA

>CYP200B3(BF49_2022)*Bradyrhizobium* sp. BF49

MSDERRFDILSPEFHANPFTTLDRMRTEGPVVRLRLPIVGRTWLAASHESCADLLKNSQDFARDPANAGSRTQ
ERILKVLPRTLGLLASNMLGHDDPEHRRLRALVDHAFQRRTIAALKPTIVETADRLLDKLDGKPEADLMAEFC
RDLPLSVICAMLGLPEKDHDRFKNWLGGLKDTANVFAVIRAIPGVISVVRHLRCVSRPGGGARPEGLIAALRE
AEIEGQRLSEDEIVSMIFLLFGAGQETTTHLISGGLYALLTNRDQLSRLRQDPGLMPTCIEECLRHVSPVQMT
KPRFATRDLVWQGRQVRRGEMFAALLAAANCDPARFEKPHTFDIGRHPNPHLSFGTGAHFCLGFQLARAEASI
GIQRILDRFPALRLARQPEDISWHKRLGIRALARLPVRLAA

>CYP200C1(GL4_3357)*Methyloceanibacter caenitepidi*

MGAGYQKSYSLRAPLRVDLQSKASKQDPFSFFAGLREAGPVVPLKLPFVGRVWATTTYDATAAMLKDNALFVQ
EGRHAGKSGVAGLSWWMPHAIKVLSNNMLLKDEPDHRRLRKLVDQAFQRRLVRDMRGEIEGLADRILDDIDGR
DEIDLVASYARRFPLEVICWLLGLPEENRGRFEQWAGYMTSALRGFAVLRVVFTMPGVVNYVREQIEECRRNP
REGLISELVRAEEDGDKLSEDELLSMVFLLLFAGFETTTHLIADSVIALEQNPAQKAFLFADPATRMERAVEE
LGRYMTPVQSTKPRYVARNCDFLGQKLKRGENIVGLLAAANSDPAAFDAPDKLKLDRLPNPHLVFGRGIHFCL
GMQLARVEAQSALSRLYGRYPDLALAAPDDLPWIERFGLRGVFALPVRLNSARARKAA

>CYP201A1(bll0557)*Bradyrhizobium diazoefficiens* USDA 110

MNIASVRRPIVPPTPPRAPDDMSFLGRVAVIRQNMIATWGQRAYEEDVLEGRFFLHKSFILNRPDAIRHVLLS
NYENYTRTPAGIRMLRPVLGEGLLIAEGHAWTFQRRTLAPAFTPRATANLVPHMTAVLDETIAKLDARSGETV
DLRETMQRMTLEIAGRTMFSFGMDRHGPTLRNFVVEYGERLGRPYFLDMLLPVSWPSPMDFARARFRKRWTEF
VAMLIAERRAAGKKDGAPPRDLFDLMDEARDPETGKGFSDEQLIDEVATMILAGHETTATALFWALYLLALDP
DTQEEVASETRGEHLDSMADIDRQKFTRAVIEETMRLYPPAFLIARAARAKDNAAGIEIGRGDIIMIAPWLLH
RHEKLWDQPNAFVPKRFMSTEAPDRFAYLPFGAGPRVCVGAPFAQAESVLALARLIGAFRVELVDTVPVIPHG
VVTTQPDRSPMFRITRR

>CYP201A2(RPA0421)*Rhodopseudomonas palustris* CGA009

MSIAAIDDRPASRAPLIPPTPPRAPENLSALGRLAAIRHNAIASWGDRAYQDDVVRGRFFAHSSYILNTPDAI
RHVLVDNTDNYRRTATGIRVLRPMLGEGLLLAEGRAWKHQRRTLAPAFTPRAVATLVPHMASATDEVVEGLRR
KTGVPLDLRETMQHLALEIAGRTMFSFEMGTHGQALRGFVIDYGTRLASPRFLDLLLPLGWPTPQDVSRALFR
RRWTRFIGELIAARRAAGKAEGAPPRDLFELMLAARDPETGEAFSDAQLGDQVATMILAGHETTATALFWALY
LLALDPDAQERLANEVRRVGFGGTEIERLPFTRAVLDETLRLYPPAFLIVREAAGPDRVAGFAVRKHDVMLIA
PWLLHRHDKLWSDPNAFVPERFLPGVPSPDRFAYLPFGVGPRVCIGAHFALVEATLALAKIVGTFRIELIDTE
PVIPIGVVTTQPDRSPLFRLTPR

>CYP201A2(Rpal_0425)*Rhodopseudomonas palustris* TIE-1

MSIAAIDDRPASRAPLIPPTPPRAPENLSALGRLAAIRQNAIASWGDRAYQDDVVRGRFFAHSSYILNTPDAI
RHVLVDNTDNYRRTATGIRVLRPMLGEGLLLAEGRAWKHQRRTLAPAFTPRAVATLVPHMASATDEVVEGLRR
KTGVPLDLRETMQHLALEIAGRTMFSFEMGTHGQALRGFVIDYGTRLASPRFLDLLLPLGWPTPQDVSRALFR
RRWTRFIGELIAARRAAGKAEGAPPRDLFELMLAARDPETGEAFSDAQLGDQVATMILAGHETTATALFWALY
LLALDPDAQERLANEVRRVGPGGTEIERLPFTRAVLDETLRLYPPAFLIVREAAGPDQVAGFAVRKHDVMLIA
PWLLHRHDKLWSDPNAFVPERFLPGAPSPDRFAYLPFGVGPRVCIGAHFALVEATLALAKIVGTFRIELIDSE
PVIPIGVVTTQPDRSPPFRLTPR

>CYP201A4(S23_02750)*Bradyrhizobium* sp. S23321

MNIASVRRPIVPPTPPRAPDDMSFLGRVALLRQNMIATWGQRAYEEDVIKGRFFLQHSFILNRPDAIRHVLLS
NYENYTRTPAGIRMLRPVLGEGLLIAEGQAWTFQRRTLAPAFTPRATANLVPHMTAVLDETIAKLDAQSGAPV
DLREIMQRMTLEIAGRTMFSFGMDRHGPTLRNFVVEYGERLGRPYFLDMLLPVSWPSPMDFPRARFRKRWTEF
VAMLIAERRAMGKKDGAPPRDLFDLMDAARDPETGKGFSDEQLVDEVATMILAGHETTATALFWALYLLALDP
DTQEAVASETRGEHLDSMADIDRQKFTRAVIDETMRLYPPAFLVARAAREKDNAAGVEIGKGDIVMIAPWLLH
RHEKLWDQPNAFIPKRFMSKEAPDRFAYLPFGAGPRVCIGAPFAQAESVLALARLIGAFRVELADSIPVIPHG
VVTTQPDRSPLFRITRR

>CYP201A5(BJ6T_05270)*Bradyrhizobium japonicum* USDA 6

MNIASVRRPIIPPTPPRAPDDLSFFGRVAVLKQNMIATWGQRAYEEEVIKGRFFLHNSFILNQPDAIRHVLLS
NYENYTRTPAGIRMLRPVLGEGLLIAEGHAWTHQRRTLAPAFTPRATANLVPHMTAVLDETITKLDARTSEPV
DLREAMQRMTLEIAGRTMFSFGMDRHGPTLRNFVMEYGARLGRPYFLDMLLPVSWPTPMDFARARFRKRWTEF
VAMLIAERRTMGKKDGAPPRDLFDLMDAARDPETGKGFSDAQLVDEVATMILAGHETTATALFWALYLLALDP
ETQEEVASETHGEHLDSMADIDRQKFTRAVIEETMRLYPPAFLVARAAREKDNAAGVEIGKGDIIMIAPWLLH
RHEKLWDQPNAFIPKRFMSKEAPDRFAYLPFGAGPRVCIGAPFAQAESVLALARLIGAFRIELADRVPVIPLG
VVTTQPDHSPLFRITRR

>CYP201A5(RN69_02575)*Bradyrhizobium japonicum* E109

MNIASVRRPIIPPTPPRAPDDLSFFGRVAVLKQNMIATWGQRAYEEEVIKGRFFLHNSFILNQPDAIRHVLLS
NYENYTRTPAGIRMLRPVLGEGLLIAEGHAWTHQRRTLAPAFTPRATANLVPHMTAVLDETITKLDARTSEPV
DLREAMQRMTLEIAGRTMFSFGMDRHGPTLRNFVMEYGARLGRPYFLDMLLPVSWPTPMDFARARFRKRWTEF
VAMLIAERRTMGKKDGAPPRDLFDLMDAARDPETGKGFSDAQLVDEVATMILAGHETTATALFWALYLLALDP
ETQEEVASETHGEHLDSMADIDRQKFTRAVIEETMRLYPPAFLVARAAREKDNAAGVEIGKGDIIMIAPWLLH
RHEKLWDQPNAFIPKRFMSKEAPDRFAYLPFGAGPRVCIGAPFAQAESVLALARLIGAFRIELADRVPVIPLG
VVTTQPDHSPLFRITRR

>CYP201A6(BF49_0293)*Bradyrhizobium* sp. BF49

MNIASVRRPIVPPTPPRAPDDMSFLARLAVIKRNMIATWGQRAYEEDVIEGRFFFRNSFILNRPDAIRHVLLS
NYENYSRTPAGIRMLRPVLGDGLLIAEGHSWTFQRRTLAPAFTPRATANLVPHMTAVLDETIAKLDGRTSEPV
DLREIMQRMTLEIAGRTMFSFGMDRHGPTLRNFVMEYGARLGRPYLLDMVLPLSWPSPMDFARARFRKRWTEF
VAMLIAERREMGKQDGAPPRDLFDLMDEARDPETGKGFSDEQLVDEVATMILAGHETTATALFWALYLLALDP
DTQEEIASETRGEHLDSMADIDRQKFTRAAIEETMRLYPPAFLIARAAREKDNAAGIEIGKGDIIMIAPWLLH
RHEKLWDQPNAFIPKRFMSTEAPDRFAYLPFGAGPRVCVGAPFAQAESVLALGRLIGAFRVELADTSPVIPLG
VVTTQPDHSPRFRITPR

>CYP201A7(BCCGELA001_01405)*Bradyrhizobium* sp. CCGE-LA001

MNIASVRRPIIPPAPPRAPDEISFFARVAVIRRNMIATWGQRAYEEDVIKGRFFFRNSFILNQPDAIRHVLLS
NYENYTRTPAGIRMLRPVLGDGLLIAEGHSWTFQRRTLAPAFTPRATANLVPHMTAVLDETIAKLDPRTGEDV
DLREVMQRMTLEIAGRTMFSFGMDRHGATLRNFIMEYAARLGRPYFLDMLLPVSWPSPMDFARARFRKRWTEF
VAMLIAERRAMGKKEGAPPRDLFDLMDEARDPETNKGFSDEQLVDEVATMILAGHETTATALFWALYLLALDP
ETQEEVASETRGEHLDSMADIDRQTFTRAVIEETMRLYPPAFLVARAARDKDNAAGVEIAKGDIIMIAPWLLH
RHEKLWDQPNAFIPKRFMSKEAPDRFAYLPFGAGPRVCIGAPFAQAESVLALARLIGAFRVELADPTSPVIPL
GVVTTQPDHSPLFRITRR

>CYP201A8(LMTR13_38220)*Bradyrhizobium icense*

MSIAEAYDMPVARAPLVPPSPPRAPDDMTVFGRMKAIRESPIGSWGQRAYEEDIVQGRFFGRSTFILNTPDAI
KHVLVDNYENYTRTPAGIRVLRPILGEGLLIAEGRAWKYQRRTLAPAFTPRAVMPLVRHMLAATDETVAKLRA
ASNVPVDLREAMQRMALEIAGRTMFSFGMDRHGAALRDFVMEYGERLARPHFLDLLLPLSWPSPQDIARRRFR
KRWTAFVGMLMAERRAAGKQEGAPARDLFDLMGEARDPETGEAFTDEQLGDQVATMILAGHETTATALFWALY
LLALDPATQEQVASEVQTATVDGAHDIERLRFTRAVIDETMRLYPPAFLIARAAIGPDTIAGLPVKKKDIILI
APWLLHRHEKLWRDPNAFIPSRFMTATPPDRFAYLPFGVGARVCIGAHFALVEATLALAKMIGAFRVTLVGKD
PVMPIGVVTTQPDRSPMFAITPR

>CYP201A9(Rpdx1_0258)*Rhodopseudomonas palustris* DX-1

MSIAVIDDRPASRAPLIPPTPPRAPDDISALGRLAAIRHNAIASWGDRAYQDDIVKGRFFTHASYILNAPDAI
RHVLIDNADNYRRTATGIRVLRPMLGDGLLLAEGRAWKHQRRTLAPAFTPRAVATLVPHMLSATEEAIAGLHR
NAGAPVDLREAMQHLALEIAGRTMFSFEMGTHGRALRSFVIDYGTRLASPRFLDLLLPLGWPTPQDIARARFR
RRWTRFVGELIAARRASGKTADAPPRDLFELMLAARDPETGEAFSDAQLGDQVATMILAGHETTATALFWALY
LLALDPDAQERLASEVRRLGPGAVEIERLPFTRAVLDETLRLYPPAFLIVREAAGADQVAGFAVRKHDVMLIA
PWLLHRHEKLWHEPDAFVPARFLPGAAPPDRFAYLPFGLGPRVCIGAHFALVEATLALAKIVGTFRIELIDTT
PVMPIGVVTTQPDRSPSFRLTPR

>CYP201A10(RPB_0116)*Rhodopseudomonas palustris* HaA2

MSIAEVYAVADRPAPLVPPTPPRAPENISALGRLAAIRRNAIASWGARAYQDDIVKGRFFTHASYILNTPDAI
RHVLVDNDENYTRTATGIRVLQPMLGEGLLLAQGRVWKHQRRTLAPAFTPRAVGTLVPHMISATEETVAELRD
SAGAPVDLREKMQHLALEIAGRTMFSFEMGSHGKALRDFVIEYGTRLASPRFLDLLLPLGWPTPQDISRAFFR
RRWTKFVGELIAARRAAGKGEGAPPRDLFDLMVAARDPETGEAFSDDQLGDQVATMILAGHETTATALFWALY
LLALDPRTQDDLADEVRAAGSGETPALDRLPFTRAVLDETLRLYPPAFLIAREAAGPDTVAGHAIHRHDVLLI

APWLLHRHEKLWDNPSAFMPSRFLPGAPPPDRFAYLPFGVGPRICIGAHFALVEATLALARVVGAFRIELIDA
APVMPIGIVTTQPDRSPMFRLTRR

>CYP201A11(RPD_0687)*Rhodopseudomonas palustris* BisB5

MSIAEVYDVADRRAPLLPPTPPRAPDNMTALGRLAALRNNAIATWGQRAYQDDLIKGRFFNHASYILNTPDAI
RHVLIDNYENYTRTAAGIRVLQPILGQGLLLAEGRAWKHQRRTLAPAFTPRAVGTLVPHMISATEETIAELQG
SLGAPVDLREAMQRLALEIAGRTMFSFAMGRHGPTLRDFVMEYGMKLASPRFLDLLLPLGWPSPQDISRAFFR
RRWTRFIGELMAARRAAGKTEGAPPRDLFDLMVVARDPETGEAFSEDQLGDQVATMILAGHETTATALFWALY
LLALDPATQQDVADEARSAGKDETPAIERLAFTRAVLDETLRLYPPAFLIAREAAGPDSVAGQTIRRKDVMLI
APWLLHRHEKLWDNPSAFMPARFLPGTPPPDRFAYLPFGVGPRVCIGAHFALVEATLALARIVGAFRIELIDP
TPVMPIGIVTTQPDHSPMFRLTRR

>CYP201A11(RPC_0051)*Rhodopseudomonas palustris* BisB18

MSIAEASQMPLTRRPLLPPAPPRAPETMTAFGRLAAIRNNVIASWGDRAYQDDVVRGRFFGSSSFILNTPDAI
RQVLVDNYENYIRTAAGIRVLRPMLGDGLLLAEGRAWKHQRRTLAPAFTPRAVATLVPHMRSAIDEMLAEFDA
ARGRPIDLREAMQRMALEIAGRTMFSFAMGRHGRTLRDFVIEYGTRLARPNFLDLLLPLAFPTPQDFARAGFR
RRWTAFIAQLMAERRAAGKAEGAPPRDLFDLMGAARDPETGEAFSEPQLGDQVATMILAGHETTATALFWALY
LLALDQHSQDQLAAEVRGQPENHAREVEHLTFTRAVIDETMRLYPPAFLIARAAAGPDVVAGSAVRKGDVMLI
APWLLHRHEKLWHDPGAFIPARFMPGSPPPDRFAYLPFGVGARVCIGAHFALVEATLALSGIVGRFRVELLNA
APVMPIGVVTTQPDYSPMFRLTPR

>CYP201A12(RPE_0057)*Rhodopseudomonas palustris* BisA53

MSPARALLTPSERPPLVPPLPPRASETMSTLQRLSAISANAIASWGDRAYQDDIVVGRFFHRSSFILNAPDAI
RHVLVDNWENYARTPAGIRVLRPMLGQGLLLAEGRPWKHQRRTLAPAFTPRAVASLVPHMLSAIDDTVTALDA
ARGGPVDLREATQHMALEIAGRTMFSFGMAEHGKALRDFVMEYGWKLAQPSLLDLLLPLSWPTPKDVSRALFR
RRWTRFIAELMAARRAEGKSDSAPPRDLFDLMVAARDPETGEAFSEPQLGDQVATMILAGHETTATALFWALY
LLALDPQTQEELAAEARAQAPGPLDVESLKFTRAVVDETLRLYPPAFLIVRAAIGPDVVGGHKVAPHDAMLIA
PWLLHRHEKLWDQPNAFIPARFLPGAPPPDRFAYLPFGVGPRICIGAHFALVEATLALAKIVGQFRIELTDSV
PVLPVGVVTTQPDRSPPFRITRRSEMGSQS

>CYP201A13(BBta_0276)*Bradyrhizobium* sp. BTAi1

MSTAEIHYLPQARAPLVPPMPPRAPDSMGAFRRVLLMGENAIATWSQRAYEDEIVRGRFFGSSSYILNTPDTI
KHVLVDNWENYVRTDGAIRVLRPVLGEGLLIAEGRAWKHQRRTLAPAFTPRAVAGLIPHMIAVTDDTVTRLKQ
QCDAPVDLREIMQRMTLDIAGRTMFSFEMGRHGATLRDFVFEYGARLASPHLLDIVLPLSWPTPRDIARRRFR
KRWTAFVTLLMAERRAAGKLDDAPPRDLFDLMGAARDPETGAAFTDAQLADEVATMILAGHETTATALFWALY
LIALDPASQSKLAQEAGAASDLGDPDRLPFTRAVIDETLRLYPPAFLIARAASGPDRLADFPVKRGDVVLISP
WLLHRHEKLWRAPNAFMPERFLPGAAPPERFAYLPFGAGARVCIGAHFALVEAVLALARLVGAFKIELIDRAP
VMPIGVVTTQPDRSPLFRITPR

>CYP201A14(BRADO0283)*Bradyrhizobium* sp. ORS 278

MSTADVHYLPRSPLVPPMPPRAPDSMGALRRVLMMGENAISTWSQRAYEDEIVHGRFFGSSSYILNTPETIKH
VLVDNWENYARTVGAIRVLRPVLGEGLLIAEGKAWKHQRRTLAPAFTPRAVSGLIPHMVAVTEETVARLQQQC
GQPLDLREIMQRMTLDIAGRTMFSFEMGRHGATLRDFVFEYGERLASPHLLDIVLPLSWPTPRDFARRRFRKR
WTDFVGMLMAERRAAGKREDAPPRDLFDLMGAARDPETGAAFTDAQLADEVATMILAGHETTATALFWALYLI
ALDPANQEKLAQEAQAATDLSNPDLLPFTRAVMDETVRLYPPAFLIARAASGPDQIAAFEVKRGDVVLISPWL
LHRHERLWRSPNAFMPERFLPGAPPPERFAYLPFGAGARVCIGAHFALVEAVLALARLVAAFRIELVDPAPVM
PVGVVTTQPDRSPLFRITPR

>CYP201A15(S58_02890)*Bradyrhizobium oligotrophicum*

MSTADVHYLPQARLPLIPPTPPRAPDSMGAFRRVLMMGENAIATWSQRAYEDDIIRGRFFGNSSYILNTPDTI
KHVLVDNWENYVRTVGALRVLRPVLGEGLLIAEGKAWKHQRRTLAPAFTPRAVAGLIPHMVAVSDETVARLKQ
QCGQPLDLREIMQHMTLDIAGRTMFSFEMGRHGATLRDFVYEYGERLASPHLLDIVLPLSWPTPRDFARRRFR

KRWTDFVAMLMAERRAAGKLEDAPPRDLFDLMGAARDPETGAAFTDAQLADEVATMILAGHETTATALFWALY
LIALDPANQDKLAQEARAATDPSDPDQLPFTRAVLDETVRLYPPAFLIARAASGPDRIAAFQVKRGDVVLISP
WLLHRHEKLWEAPNAFKPERFLPGAPPPERFAYLPFGAGARVCIGAHFALVEAVLALARLVGAFKIELVDRVP
VMPVGVVTTQPSRSPLFKITPR

>CYP201A16(Y590_05405)*Methylobacterium* sp. AMS5

MDMQAPPGHAAPRLVPPVPVPPEEELPTLRFIAAMRVNGIACWPASAYESPLRRRRLFGRARFTVSDPDLVRR
VLVDNAANYARTPITIRLLRPMLGDGLLISEGTAWRHQRRALAPAFTPRAVETLVPHILSASDEAVAALEGPA
ARGPVDLFAALQRLALEIAGRTMFSVGMARHGDRLRGFLEAYAARLGRPHLTDLLVPLRFATPLDRARARFRR
DWVGFLDEVIADRDEGQSGRHVGGEARDLLDLLRAARDPETGRGFSHEELRDQVATMILAGHETTAVTLLWAC
TLLALAPETQGAVAAESGSVDKPFTRAVIEETMRLYPPAFVLARRALGPDELAGEAIRPGDSVTISPWLLHRH
RTLWRDPDAFDPGRFLPGAPPVPRFAYLPFGAGPRVCIGAAFALTEATLALSRIVGHFRIERADTRPVLPAAV
VTTQPDHAPAFRLTRRA

>CYP201A16(METDI1856)*Methylobacterium extorquens* DM4

MDMQAPPGHAAPRLVPPVPVPPEKELPTLRFIAAMRVNGIACWPASAYEAPLRRRRLLGRTRFTVSDPDLVRR
VLVDNAANYARTPITIRMLRPMLGDGLLISEGTAWRHQRRALAPAFTPRAVETLVPHILSASDEAVAALEGPA
SRGPVDLFAALQRLALEIAGRTMFSVGMARHGDRLRGFLEAYAARLGRPHLTDLVVPLRFATPLDQARARFRR
DWVTFLDEVIADRDEGQSGRQVGGHGGGEARDLLDLLRAARDPETGRGFSHEELRDQVATMILAGHETTAVTL
LWACTLLALAPETQGAVAAESASADKPFTRAVIEETMRLYPPAFVLARRALGPDELAGEAVRAGDSVTISPWL
LHRHRKLWRDPDAFDPSRFLPGAPPVPRFAYLPFGAGPRVCIGAAFALTEATLALSRIVGHFRIERADARPVL
PAAVVTTQPDHAPAFRLTRRV

>CYP201A16(Mext_1270)*Methylobacterium extorquens* PA1

MDMQAPPGHAAPRLVPPVPVPPEKELPTLRFIAAMRVNGIACWPASAYEAPLRRRRLLGRTRFTVSDPDLVRH
VLVDNAANYARTPITIRMLRPMLGDGLLISEGTAWRHQRRALAPAFTPRAVETLVPHILSASDEAVAALEGPA
AQGPVDLFAALQRLALEIAGRTMFSVGMARHGDRLRGFLEAYAARLGRPHLTDLVVPLRFATPLDRARARFRR
DWVGFLDEVIADRDEGQSGRQVGGHGGGEARDLLDLLRAARDPETGRGFSHEELRDQVATMILAGHETTAVTL
LWACTLLALAPETQGAVAAESASADKPFTRAVIEETMRLYPPAFVLARRALGPDELAGEAVRPGDSVTISPWL
LHRHRKLWRDPDAFDPRRFLPGAPAVPRFAYLPFGAGPRVCIGAAFALTEATLALSRIVGHFRIERADTRPVL
PAAVVTTQPDHAPAFRLTRRV

>CYP201A16(Mex_1p1055)*Methylobacterium extorquens* AM1

MDMQAPPGHAAPRLVPPVPVPPEKELPTLRFIAAMRVNGIACWPASAYEAPLRRRRLLGRTRFTVSDPDLVRH
VLVDNAANYARTPITIRMLRPMLGDGLLISEGTAWRHQRRALAPAFTPRAVETLVPHILSASDEAVAALEGPA
ARGPVDLFAALQRLALEIAGRTMFSVGMARHGDRLRGFLEAYAARLGRPHLTDLVVPLRFATPLDRARARFRR
DWVTFLDEVIADRDEGQSGRQVGGHGGGEARDLLDLLRAARDPETGRGFSHEELRDQVATMILAGHETTAVTL
LWACTLLALAPETQGAVAAESASADKPFTRAVIEETMRLYPPAFVLARRALGPDELAGEAVRPGDSVTISPWL
LHRHRKLWHDPDAFDPSRFLPGAPPVPRFAYLPFGAGPRVCIGAAFALTEATLALSRIVGHFRIERADARPVL
PAAVVTTQPDHAPAFRLTRRV

>CYP201A16(Mchl_1432)*Methylobacterium extorquens* CM4

MDMQAPPGHAAPRLVPPVPVPPEKELPTLRFIAAMRVNGIACWPASAYEAPLRRRRLLGRTRFTVSDPDLVRH
VLVDNAANYARTPITIRMLRPMLGDGLLISEGTAWRHQRRALAPAFTPRAVETLVPHILSASDEAVAALEGPA
ARGPIDLFAALQRLALEIAGRTMFSVGMARHGDRLRGFLEAYAARLGRPHLTDLVVPLRFATPLDRARARFRR
DWVTFLDEVIADRDEGQSGRQVGGHGGGEARDLLDLLRAARDPETGRGFSHEELRDQVATMILAGHETTAVTL
LWACTLLALAPETQGAVAAESASADKPFTRAVIEETMRLYPPAFVLARRALGPDELAGAAVRPGDSVTISPWL
LHRHRKLWRDPDAFDPSRFLPGAPPVPRFAYLPFGAGPRVCIGAAFALTEATLALSRIVGHFRIERADARPVL
PAAVVTTQPDHAPAFRLTRRV

>CYP201A17(Mpop_1222)*Methylobacterium populi*

MQAPPAGAVPRLVPPVPAPPERELPTLRFIAAMRVNGIDCWPASAYEAPLRRRRLFGRTRFTVSDPDLVRHVL
VDNAANYARTPITIRMLRPMLGDGLLISEGTAWRHQRRTLAPAFTPRAVETLVPHILSASDEAVAALEGPAAR
GPVDLFAALQRLALEIAGRTMFSVGMAHHGDRLRGFLEAYAARLGRPHLTDLLVPLRFATPLDRARARFRRDW
VAFLDGVIADRDQGQDGRHQGEARDLLDLLRAARDPETGRGFSHEELRDQVATMILAGHETTAVTLLWACTLL
ALSPETQAAVAAEAASAEKPFTRAVIEETLRLYPSAFVLARRALHADVLGGERVEAGDSVTISPWLLHRHRRL
WRDPDAFDPGRFLPGAPPVPRFAYLPFGAGPRVCIGAAFALTEATLALSRIVGHFRIERADARPVLPAAVVTT
QPDHAPAFRLTPRA

>CYP201A18(Mrad2831_3371)*Methylobacterium radiotolerans*

MPTFSDATATPDRTIPSLAGMPRVVPPHRAPPERELPLAAYLRSIRDNALAGFPERAFEEPVTRRGLLGRSSF
VLNDPEAIRRVLVENQANYARTTGTTRILRPILGDGLLISEGSAWRHQRRTLAPAFTPRAIDGLVPHIAAAVT
DGMERIAAEAAAGPVDLFTSFYRLALEIAGRAMFSVGMDEHGAELRQFIAEYAERMGRPHLLDIVIPPGWPVP
LDWSRGRFRRRWIPFLDRIIAARRAAERDRSRSGDLLDLLAAARNPDTGAAFTPDELRDQVATMILAGHETTA
GTLFWAAYLLALAPEVQERVAAEARAADLTDPTGCQSDGRLPLTRAVIDETLRLYPAAFVVVRRALGPDNVAG
HAVKASDIVMVSPWVLHRHRALWTDPDAFDPGRFLPGAPAPRRFAYLPFGAGPRVCIGAQFALSEAVLSLARV
LQRFRLVLDEREPVLPQAVVTTQPDRPARFRLIPREPLSP

>CYP201A18(MOC_3877)*Methylobacterium oryzae*

MPILSDASARPDRTTPSLAGLPRVVPPHRAPPERELPLAAYLRSIRDNALEGFPRRAFEEPVTRRGLLGRSSF
VLSDPEAIRRVLVENQANYARTTGTTRILRPILGDGLLISEGSAWRHQRRTLAPAFTPRAIDGLVPHIDAAVA
DGMDRIAAEAATGPVDLFTSFYRLALEIAGRAMFSVGMDEHGAELRRFIAEYAERMGRPHLLDIVIPPGWPVP
LDWSRARFRRRWIPFLDRIIAARRDAERDRPHAGDLLDLLAAARNPDTGAAFTADELRDQVATMILAGHETTA
GTLFWAAYLLALAPEMQERVAAEARSADLTDPTGCQSDGRLPLTRAVIDETLRLYPAAFVVVRRALGPDSVAG
HAVKASDIVMVSPWVLHRHRTLWSDPDAFDPGRFLPGAPAPRRFAYLPFGAGPRVCIGAQFALSEAVLSLARL
LARFRLVLDEREPVLPQAVVTTQPDRPARFRLIPRDPVSP

>CYP201B1(Mnod_5133)*Methylobacterium nodulans*

MGLGASAAGDEDELYAPPMPPLAERVGSLRLLRAFRRNTLDAFPRACLHEPAVTLRLPGGGCVVLLCVPEAVR
HVLVSAAGDFARLPAGRRVLGPIAGRGLVLAEGEAWRRQRRVLAPAFTPRTVPLMAGHIARATEASIRRLDAA
GGAPVDLQAEMQRLSLDIAASSMFSLETGPFEARIRRMVSGYLGGLGRPQVSDFLLPPGWPTPASPRRALFRR
RWLRLVGEIIAARRAQGHGEAPRDLFDLLSTAHDEADGLLADEVGTMIVAGHETTASTLFWAATLLARAPAVQ
DALAAEARALDLGEESAADSLPRLSLARAVVQETLRLYPPAYMIARRALRDGATCGAALPAGATVMIPTWVMH
RNPRWWARPGAFDPQRFLRPGTEPDRYVYLPFGAGPRVCIGAPLALAEATLVLARLARDFVVSLDGDRPVMPV
ATVTVRPDHAPRFRLRRRP

>CYP201B2(M446_4522)*Methylobacterium* sp. 4-46

MTSGAVLERADLYAPPMPPLAERVDWLRLLSAFRRNTLDAFPGACLTEPTVTLRLPGGGRVVLLCVPEAVRHV
LVAQGSDFARLPAGIRVLGPVAGRGLVTAEGEAWRRQRRVLAPAFTPRTVPLMARHIARATAACLRRLDAARG
GPVDLHAEMQRLSLDIAASSMFSLEAGPYEARIRAMVSAYLGGLGRPRVSDFLVPPGWPTPAAPRRWLFRRRW
RALIAELIAVRRAQVAGQGPRDLFDLLAEAHGEEPDGLLADEVGTMIVAGHETTASTLFWAATLLARAPAVQE
ALAAEARGLDLGEAGAAAALPRLALARAVVQEALRLYPPAYMIARRAVRDGAVCGAAIPAGTTVMIPTWVMHR
NPRWWPRADAFDPERFLRPGEEPDRFVYLPFGAGPQVCIGAQLALAEATLALAGLARRFALSLDGARPILPVA
TVTVRPDHAPPFRLARR

>CYP201B3(Maq22A_c18715)*Methylobacterium aquaticum*

MTLTFAAEAYAPPVPDLAERVGWLGLLGAFRRNTLEAFPRACLDAPVVTIPLPGGRMVLLSTPAGARHVLLTA
ADDFERLPAGRRVLGPIAGRGLVLADGEAWRHQRRVLAPAFTPRTLPLMLRFVARSAEESCRRLEAGLGAPVD
LRGEMQQVALDIATTSMFSLETGPLGAEIRALISGYLGRLGRPTVADFLLPPAWPTPSSPRRALFRRRWLKTV
RAVLAARRAQGQRQGQGPGQEDGMRRDAPRDLFDLLDAAHGDAPGGLLADEIGTLIVAGHETTASTLFWACTL
LARAPAVQDALAAEARGLDLTEAGAADTLPHLRLARAVAQEALRLYPPVYVIARRAARTSTVEGVTVPAGSIV
MVPTWILHRNPRWWDRPAAFDPGRFLDRPEPDRFLYLPFGAGPRICIGAQLALAEATLVLARLARDFRLALDG
NRPVLPVATTTVRPDHVPRFHLARRAG

>CYP201C1(GDI2592)*Gluconacetobacter diazotrophicus* Pal 5 (Brazil)

MLESSPLIPPMPPMLADRAGGLGVLAALRRNGYSAFPRRCFDQSVVSLRALGRPLVLAAGPDSVQNILVTQAG
GYRRLRVGQRVLSPIVGRGLLVSEGETWRRQRRAMAPAFTPRGVPVLAGHIAACADRACRALEPDRPVDLFST
MRRLALDVAAVSMFSLDIGSFDRPLRDSVTRFMTGIGTPRPGDFLLPPGMPTLLGWRRRRFRRRWVALIGQII
AARQALGPTDAPRDLFDLMQAAFGAEGGGTTSELIDEVATMIVAGHETTASVLFWACLLLAQSPRWQDEVARE
VRDMDLSPAGAATTLPALHVTTAVVRETLRLYPPAFMSGRESVDEADLCGHTVPKGAMVLLPFWMLHRNPALW
DNPGCFDPARFLDRPDPPRFAFLPFGAGPHVCIGAQLAMTEAVLVLARVVQDVSLALVDHAPVLPVGVLSTRP
SYAPAFRLTRRA

>CYP201C1(Gdia_0823)*Gluconacetobacter diazotrophicus* PA1 5 (JGI)

MLESSPLIPPMPPMLADRAGGLGVLAALRRNGYSAFPRRCFDQPVVSLRALGRPLVLAAGPDSVQNILVTQAG
GYRRLRVGQRVLSPIVGRGLLVSEGETWRRQRRAMAPAFTPRGVPVLAGHIAACADRACRALEPDRPVDLFST
MRRLALDVAAVSMFSLDIGSFDRPLRDSVTRFMTGIGTPRPGDFLLPPGMPTLLSWRRRRFRRRWVALIGQII
AARQALGPTDAPRDLFDLMQAAFGAEGGGTTSELIDEVATMIVAGHETTASVLFWACLLLAQSPRWQDEVARE
VRDMDLSPAGAATTLPALHVTTTVVRETLRLYPPAFMSGRESVDEADLCGHTVPKGAMVLLPFWMLHRNPALW
DNPGCFDPARFLDRPDPPRFAFLPFGAGPHVCIGAQLAMTEAVLVLARVVQDVSLALVDHAPVLPVGVLSTRP
SYAPAFRLTRRA

>CYP201C2(A0U92_00625)*Acetobacter aceti*

MAVLFSPPMPALPSDDITGLGVLFRLKRNGFGAFPERCFHEPVLSLRALGKPLVIAAGPEAIKAVLATEAASF
QRLRMGRRVLSPIVGEGILVSEGDVWRRQRRAMAPAFTPRTVPTLARHIAACADTTLLRISIEKPVDVFALTQ
TLALDIAAMTMFSMTSETFGVTLRRMVTSFMSGIGQPRPADFLLPPSVPTLTDWRRKHFRRRWVTLIEAIIAE
RPQRGTQEEARDLFDLLMQAFGGEGGLAGQAVVDEVATMIVAGHETTATALFWAVWLLANTPEWQDAIAEEVR
HIDLSPDHAAGALSHLPVTTSVVREALRLFPPAFMTGREAVSDGDLCGQAIPRGAMILLPFWMLHRNPALWKN
PAAFDPGRFLNGKEPDRFTFLPFGAGPHVCIGAQLAMTEAVLVLARLVQLYRFKAADEKPVLPVGVLSTRPSR
SVLFRFSPRWNCG

>CYP202A1(SMc01812)*Sinorhizobium meliloti* 1021

MSIAPGITIDGPARRVSLDVRNPRFFRNPLPAYAALHAQCPAFFWEEPQQWFFAGYEQVNSLLRDRRFGRQIL
HVATREELGMPEPKPHLKDFDALEAHSLLELEPPAHTRLRTLVNRAFVSRQIEELRPEIEALSHAVIDGFEKD
GETELLKTYAETIPVTIIARMLGIPVEAAPRLLDWSHRMVKMYVFNPSLETEFDANNASAEFADYLKGIIAEK
RTNPADDLLTHMITSEKDGERLSDAELISTTVLLLNAGHEATVHQIGNAVRTILQSGLSPAELFSDEKATERT
VEECLRFAAPLHIFQRYALMDIELENGIALRKGDKIGLMLGAANVDPRKFSSPDTFRPDRNEGANVSFGAGLH
FCIGAPLARLELQISLPILFRRLPGMRLKNEPPVKDAFHFHGLERLDLVW

>CYP202A1(SinmeB_0879)*Sinorhizobium meliloti* BL225C

MSIAPGITIDGPARRVSLDVRNPRFFRNPLPAYAALHAQCPAFFWEEPQQWFFAGYEQVNSLLRDRRFGRQIL
HVATREELGMPEPKPHLKDFDALEAHSLLELEPPAHTRLRTLVNRAFVSRQIEELRPEIEALSHAVIDGFEKD
GETELLKTYAETIPVTIIARMLGIPVEAAPRLLDWSHRMVKMYVFNPSLETEFDANNASAEFADYLKGIIAEK
RTNPADDLLTHMITSEKDGERLSDAELISTTVLLLNAGHEATVHQIGNAVRTILQSGLSPAELFSDEKATERT
VEECLRFAAPLHIFQRYALMDIELENGIALRKGDKIGLMLGAANVDPRKFSSPDTFRPDRNEGANVSFGAGLH
FCIGAPLARLELQISLPILFRRLPGMRLKNEPPVKDAFHFHGLERLDLVW

>CYP202A1(C770_GR4Chr1265)*Sinorhizobium meliloti* GR4

MSIAPGITIDGPARRVSLDVRNPRFFRNPLPAYAALHAQCPAFFWEEPQQWFFAGYEQVNSLLRDRRFGRQIL
HVATREELGMPEPKPHLKDFDALEAHSLLELEPPAHTRLRTLVNRAFVSRQIEELRPEIEALSHAVIDGFEKD
GETELLKTYAETIPVTIIARMLGIPVEAAPRLLDWSHRMVKMYVFNPSLETEFDANNASAEFADYLKGIIAEK
RTNPADDLLTHMITSEKDGERLSDAELISTTVLLLNAGHEATVHQIGNAVRTILQSGLSPAELFSDEKATERT
VEECLRFAAPLHIFQRYALMDIELENGIALRKGDKIGLMLGAANVDPRKFSSPDTFRPDRNEGANVSFGAGLH
FCIGAPLARLELQISLPILFRRLPGMRLKNEPPVKDAFHFHGLERLDLVW

>CYP202A1(SM2011_c01812)*Sinorhizobium meliloti* 2011

MSIAPGITIDGPARRVSLDVRNPRFFRNPLPAYAALHAQCPAFFWEEPQQWFFAGYEQVNSLLRDRRFGRQIL
HVATREELGMPEPKPHLKDFDALEAHSLLELEPPAHTRLRTLVNRAFVSRQIEELRPEIEALSHAVIDGFEKD
GETELLKTYAETIPVTIIARMLGIPVEAAPRLLDWSHRMVKMYVFNPSLETEFDANNASAEFADYLKGIIAEK
RTNPADDLLTHMITSEKDGERLSDAELISTTVLLLNAGHEATVHQIGNAVRTILQSGLSPAELFSDEKATERT
VEECLRFAAPLHIFQRYALMDIELENGIALRKGDKIGLMLGAANVDPRKFSSPDTFRPDRNEGANVSFGAGLH
FCIGAPLARLELQISLPILFRRLPGMRLKNEPPVKDAFHFHGLERLDLVW

>CYP202A1(DU99_06500)*Sinorhizobium meliloti* RMO17
MSIAPGITIDGPARRVSLDVRNPRFFRNPLPAYAALHAQCPAFFWEEPQQWFFAGYEQVNSLLRDRRFGRQIL
HVATREELGMPEPKPHLKDFDALEAHSLLELEPPAHTRLRTLVNRAFVSRQIEELRPEIEALSHAVIDGFEKD
GETELLKTYAETIPVTIIARMLGIPVEAAPRLLDWSHRMVKMYVFNPSLETEFDANNASAEFADYLKGIIAEK
RTNPADDLLTHMITSEKDGERLSDAELISTTVLLLNAGHEATVHQIGNAVRTILQSGLSPAELFSDEKATERT
VEECLRFAAPLHIFQRYALMDIELENGIALRKGDKIGLMLGAANVDPRKFSSPDTFRPDRNEGANVSFGAGLH
FCIGAPLARLELQISLPILFRRLPGMRLKNEPPVKDAFHFHGLERLDLVW

>CYP202A1(SM11_chr2345)*Sinorhizobium meliloti* SM11

MSIAPGITIDGPARRVSLDVRNPRFFRNPLPAYAALHAQCPAFFWEEPQQWFFAGYEQVNSLLRDRRFGRQIL
HVATREELGMPEPKPHLKDFDALEAHSLLELEPPAHTRLRTLVNRAFVSRQIEQLRPEIEALSHAVIDGFEKD
GETELLKTYAETIPVTIIARMLGIPVEAAPRLLDWSHRMVKMYVFNPSLETEFDANNASAEFADYLKGIIAEK
RTNPADDLLTHMITSEKDGERLSDAELISTTVLLLNAGHEATVHQIGNAVRTILQSGLSPAELFSDEKATERT
VEECLRFAAPLHIFQRYALMDIELENGIALRKGDKIGLMLGAANVDPRKFSSPDTFRPDRNEGANVSFGAGLH
FCIGAPLARLELQISLPILFRRLPGMRLKNEPPVKDAFHFHGLERLDLVW

>CYP202A1(Sinme_1049)*Sinorhizobium meliloti* AK83

MSIAPGITIDGPARRVSLDVRNPRFFRNPLPAYAALHAQCPAFFWEEPQQWFFAGYEQVNSLLRDRRFGRQIL
HVATREELGMPEPKPHLKDFDALEAHSLLELEPPAHTRLRTLVNRAFVSRQIEQLRPEIEALSHAVIDGFEKD
GETELLKTYAETIPVTIIARMLGIPVEAAPRLLDWSHRMVKMYVFNPSLETEFDANNASAEFAGYLKGIIAGK
RTNPADDLLTHMITSEKDGERLSDAELISTTVLLLNAGHEATVHQIGNAVRTILQSGLSPAGLFSDEKATERT
VEECLRFAAPLHIFQRYALMDIELENGIALRKGDKIGLMLGAANVDPRKFSSPDTFRPDRNEGANLSFGAGLH
FCIGAPLARLELQISLPILFRRLPGMRLKNEPPVKDAFHFHGLERLDLVW

>CYP202A1(BN406_00987)*Sinorhizobium meliloti* Rm41

MSIAPGITIDGPARRVSLDVRNPRFFRNPLPAYAALHAQCPAFFWEEPQQWFFAGYEQVNSLLRDRRFGRQIL
HVATREELGMPEPKPHLKDFDALEAHSLLELEPPAHTRLRTLVNRAFVSRQIEQLRPEIEALSHAVIDGFEKD
GETELLKTYAETIPVTIIARMLGIPVEAAPRLLDWSHRTVKMYVFNPSLETEFDANNASAEFAGYLKGITAGK
RTNPADDLLTHMITSEKDGERLSDAELISTTVLLLNAGHEATVHQIGNAVRTILQSGLSPAGLFSDEKATERT
VEECLRFAAPLHIFQRYALMDIELENGIALRKGDKIGLMLGAANVDPRKFSSPDTFRPDRNEGANLSFGAGLH
FCIGAPLARLELQISLPILFRRLPGMRLKNEPPVKDAFHFHGLERLDLVW

>CYP202A3(Atu1256)*Agrobacterium fabrum*

MTATFPFLKIDPATRRVSLNARDPAFYNDPNPVYAALHAQCPTFYWEEQRQWFFTCYDHVSTLLRDRRFGRQI
LHVASREEIGLPEPLEHVKHFDLAEQHSLLELEPPEHTRLRTLINRAFVSRHVDKMKPEIEELANRLIEAFEA
NGETELLSSYADIIPVTMIARMIGIPEEMGPQLLKWSHAYVGMYMFKRTPEDELLADKAAQEFSDYVRRVIAE
RRAEPKDDLLSHMIHTEHKGQYLTDDELVSTTIVLLNAGHEATVHQIGNSVRIILESGLDPKTLFHDETATER
TVEETLRICAPVHIFQRWVLEPVEIDGVQFKRGDKVSLILAAANLDPAKFSDPLAFQPDRNEGANVSFGAGIH
FCIGAPLARLELNLALPLLFKRLPGLKIAEPPKVKDVYHFHGLERLDLAW

>CYP202A6(Ach5_11600)*Agrobacterium tumefaciens* Ach5

MDMTATFPFLKIDPATRRVSLDARNPAFYNDPNPVYAALHAQCPTFYWEEQRQWFFTEYDHVSALLRDRRFGR
QILHVASREEIGLAEPLEHVKHFDAAEQHSLLELEPPEHTRLRTLINRAFVSRHVDKMKPEIEELANRLIDAF
EGNGKTELLSSYADIIPVTMIARMIGIPEEMGPQLLKWSHAYVGMYMFKRTPEDELLADRAAHEFSDYVRSVI
AERRAEPKDDLLSHMIHTEHKGQYLTDDELISTTIVLLNAGHEATVHQIGNSVRIILESGIAPDTLFHDETAT

ERTVEETLRICAPVHIFQRWVLEPVEIDGVEFKRGDKVSLILAAANLDPAKFSDPLAFRPDRNEGANVSFGAG
IHFCIGAPLARLELNLALPLLFKRLPGLKIAEPPRVKDVYHFHGLERLDLSW

>CYP202A6(AGROH133_05597)*Agrobacterium* sp. H13-3

MDMTATFPFLKIDPATRRVSLDARNPAFYNDPNPVYAALHAQCPTFYWEEQRQWFFTEYDHVSALLRDRRFGR
QILHVASREEIGLAEPLEHVKHFDAAEQHSLLELEPPEHTRLRTLINRAFVSRHVDKMKPEIEELANRLIDAF
EGNGKTELLSSYADIIPVTMIARMIGIPEEMGPQLLKWSHAYVGMYMFKRTPEDELLADRAAHEFSDYVRSVI
AERRAEPKDDLLSHMIHTEHKGQYLTDDELISTTIVLLNAGHEATVHQIGNSVRIILESGIAPDTLFHDETAT
ERTVEETLRICAPVHIFQRWVLEPVEIDGVEFKRGDKVSLILAAANLDPAKFSDPLEFRPGRNEGANVSFGAG
IHFCIGAPLARLELNLALPLLFKRLPGLKIAEPPKVKDVYHFHGLERLDLSW

>CYP202A6(B0909_07125)*Agrobacterium rhizogenes*

MTATFPFLKIDPATRRVSLNARDPAFYNDPNPVYAALHAQCPTFYWEEQRQWFFTGYDHVSALLRDRRFGRQI
LHVASREEIGLAEPLEHVKHFDAAEQHSLLELEPPEHTRLRTLINRAFVSRHVDKMKPEIEELANRLIDAFEA
NSETELLSSYADIIPVTMIARMIGIPEEMGPQLLKWSHAYVGMYMFKRTAGDELLADTAAREFSDYVRSVIAE
RRAEPQDDLLSHMIHTEHKGQYLTDDELVSTTIVLLNAGHEATVHQIGNSVRIILESGISPDTLFSDETTTER
TVEETLRICAPVHIFQRWVLEPVEIDGVTFKRGDKVSLILAAANLDPAKFSDPLAFRPGRNEGVNVSFGAGIH
FCIGAPLARLELNLALPLLFRRLPGLRIAQPPKVKDVYHFHGLERLDLAW

>CYP202A7(BN877_I1238)*Rhizobium* sp. IRBG74

MTATFPFLKIDSATRHVSLDARDPAFYNDPNPVYAALHAQCPTFYWEEQRQWFFTSYDHVSSLLRDRRFGRQI
LHVASREEIGLPEPLEHVKHFDLAEQHSLLELEPPEHTRLRTLINRAFVSRHVDKMKPEIEELANRLIDAFEE
KGETELLSSYADIIPVTMIARMIGIPEEMGPQLLKWSHAYVGMYMFKRTPEDELLADMAAKEFSDYVRSVIAE
RRAKPEDDLLSHMIHTEHKGQYLTDDELVSTTIVLLNAGHEATVHQIGNSVRIILESGISPQTLFSDEAATER
TVEETLRICAPVHIFQRWVLEPVEIDGVQFKRGDKVSLILAAANLDPSKFPDPLTFRPERNEGANVSFGAGIH
FCIGAPLARLELNLALPLLFKRLPRLKIAEPPKVKDVYHFHGLERLDLSW

>CYP202A12(Smed_0869)*Sinorhizobium medicae*

MSTAPGITIDGPVRRVSLDVRNPEFFRNPLPAYAALHAHCPAFFWEEPQQWFFAGYAQVNSLLRDRRFGRQIL
HVATREELGMPEPQPHLKDFDALEAHSLLELEPPTHTRLRTLVNRAFVSRQIEQLRPDIEALSHRVIDGFEKN
GAVELLKTYAETIPVTIIARMLGIPVEAAPRLLDWSHRMVKMYVFNPSLETELDANQASAEFADYLKGIIDEK
RVKPADDLLTHMITAEKDGERLSDAELISTTVLLLNAGHEATVHQIGNAVRTILQSGLSPADLFSNEEATERT
VEECLRFAAPLHIFQRYALMDMELEDGIVLKKGEKIGLMLGAANVDPRKFSDPDTFRPNRNEGANVSFGAGLH
FCIGAPLARLELQISLPILFQRLPGMRLKNEPPVKDSFHFHGLERLDLVW

>CYP202A13(NGR_c10310)*Sinorhizobium fredii* NGR234

MSAVPGITIDGPARRVCLDVRNPDFFRNPLPAYAALHAQCPAFFWEEPQQWFFAGYDQVNALLRDRRFGRQIL
HVATREELGLPEPKPHLGDFDALEAHSLLELEPPAHTRLRTLVNRAFVSRQIEQLRPDIEALSHAIIDSFEAD
GEVELLKTYAETIPVTIIARMLGIPVEAAPRLLDWSHRMVKMYVFNPSFETELEANQASAEFAGYLNDIIAEK
RAKPADDLLTHMISSEKDGERLSEAELISTTVLLLNAGHEATVHQIGNAVSTILQSGLFPAALFASEEATART
VEECLRFAAPLHIFQRYALSDIELEDGITLRKGDKIGLMLGAANVDPRKFTAPDMFMPDRNEGANVSFGAGLH
FCIGAPLARLELNLSLPILFRRLPGLRLKHEPPVKDSFHFHGLERLDLVW

>CYP202A13(SFHH103_00970)*Sinorhizobium fredii* HH103

MSAVPGITIDGPARRVCLDVRNPDFFRNPLPAYAALHAQCPAFFWEEPQQWFFAGYEQVNALLRDRRFGRQIL
HVATREELGLPEPKPHLKDFDALEAHSLLELEPPAHTRLRTLVNRAFVSRQIEQLRPDIEALSHAIIDSFEAD
GAVELLKTYAEAIPVTIIARMLGIPVEAAPHLLDWSHRMVKMYVFNPSFETELDANQASAEFAAYLKEIIAEK
RAKPADDLLTHMIGSEKDGERLSEAELISTTVLLLNAGHEATVHQIGNAVHAILQSGLSPATLFAGEEATART
VEECLRFAAPLHIFQRYALSDIVLEDGIALRKGDRIGLMLGAANVDPRKFMAPDTFMPDRNEGANMSFGAGLH
FCIGAPLARLELNLSLPILFRRLPGLRLKHEPPVKDSFHFHGLERLDLVW

>CYP202A14(USDA257_c32830)*Sinorhizobium fredii* USDA 257

MSAVPAFTIDGPARRASLDVRNPDFFRNPLPAYAALHAQCPAFFWEEPQQWFFAGYDQVNALLRDRRFGRQIL
HVATREELGLPEPKPHLKDFDALEAHSLLELEPPAHTRLRTLVNRAFVSRQIEQLRPDIETLSHAIIDSFETD
GEVELLKSYAETIPVTIIARMLGIPVEAAPRLLDWSHRMVKMYVFNPTFETELDANQASAEFAEYLKNIITDK
RTKPADDLLTHMINSEKDGERLSEAELISTTVLLLNAGHEATVHQIGNAVRTILQSGLSPTTLFAGEEATERT
VEECLRFAAPLHIFQRYALSDIELEDGIALRKGDKIGLMLGAANVDPRKFTAPGKFKPDRNEGANVSFGAGLH
FCIGAPLARLELNLSLPILFQRLPCLRLKHEPQVKDSFHFHGLERLDLVW

>CYP202A15(SAMCFNEI73_Ch1362)*Sinorhizobium americanum*

MSPAPGITIDGPARRVSLDVRNPDFFRNPLPAYGALHAQCPAFFWEEPQQWYFAGYDQVNALLRDRRFGRQIL
HVATREDLGLSEPKPHLKDFDALEEYSLLELEPPAHTRLRTLVNRAFVSRQIEQLRPDIETLSHAIIDGFKKD
GEVELLKAYAETIPVTIIARMLGIPVEAAPRLLDWSHRMVKMYVFNPSVETELDANQASTEFAAYLKDIIAEK
RVSPADDLLTHMIRSEKDGERLSEAELISTTVLLLNAGHEATVHQIGNAVSTILKSGFSPDQLFASEEATART
VEECLRFAAPLHIFQRYALSDIELEDGISLRKGDKIGLMLGAANVDPRKFTAPDTFRPDRDEGANVSFGAGLH
FCIGAPLARLELNLSLPILFRRLSGLRLKDEPPVKDSFHFHGLERLDLVW

>CYP202A16(OV14_2294)*Ensifer adhaerens* OV14

MPPKPAIIIDSAKRSVSIDVHDPDFYRAPLSAYAEIHAHCPAFYWSEREQWYFAGYDQVNALLRDRRFGRQIL
HVATREELGMPAPKPHLKDFDALEEHSLLELEPPTHTRLRTLVNRAFVSRQIEQLRPDIERLSHTIIDGFEQD
GEVELLKTYAETIPVTIIARMLGIPVDAAPNLLDWSHRMVRMYVFNRSHETEFDANQASADFADYLKTIIAEK
RRKPADDLLTHMITSEKDGERLSEAELVSTAALLLNAGHEATVHQIGNAVRTILQSDHAPGDLFATEATTDLA
IEECLRFAAPLHIFQRYALSDIELDEGIALRKGDKIGLLLGAANVDPRKFSDPDRFKPARDEGVNVSFGAGLH
FCIGAPLARLELKLSLPILFQRLPGMRLKHPPEVKDSFHFHGLERLDLVW

>CYP202A17(KKY_1509)*Pelagibacterium halotolerans*

MAAIVQVRPVTDVPDGLSASAQPSDPAFFQNPYPFYAARQATHPAFFWSEYGHWCFADFPTVSALLRDRRFGR
DILHVATREEIGLPQPKPHTADFDLTEKYSLLNLEPPAHSRLRTLVNRAFVSRQVELLRPRIADLVHSIIDDF
EDQGGVELIRAFAAPIPAIVIAEMIGLPAEMAPQLLDWSNRMVRMYMFDVSHETERDANRAAADFTAYLRQAI
AERRKLPREDLLSHMIAAEQGGERLSEDEMLSTAILLLNAGHEATVHTTGNAVKAILESGYDPRMLFADDTAT
AATVEEALRFDAPLHMFTRYALEDLDYNGIALRKGDVIGLMLGAANRDPNRFTNPNTFDPLRTDGANVSFGAG
IHFCIGAPLARLEMQIALKILFERLPKLRLTAPPRYADIYHFHGLERLDVSW

>CYP202A18(XM25_07155)*Devosia* sp. H5989

MISSTDAIAPARRASANPRDPVFYQNPYAFYDGIHATSPTFFWDNYGHWCFAGFKEVNQLLRDKRFGREILHV
ATREELGWPERKPHVADFDLAEKYSLLNLEPPAHTRLRTLVNRAFVSRQVEQLRPRVAALANELIDGFEKDGQ
VELIHAYAAPIPAIVISEMIGVPTEMAMQVVAWSNRMVTMYMFGVTEDTERDANQASADFIAYIRTLIAERRV
RPTEDLLTHMITAEQDGQKLTDDEIVSTAILLLNAGHEATVHTTGNSVKTIIESGIDRKAIFASDEQTAATVE
ECLRFDAPLHMFTRFALSDIELDNGVSLRKGDQIGLMLGAANRDPVRFANANTFDPFRTDGQNVSFGAGIHFC
IGAPLARIELQISLKTLFDRLPGLRLAKEPSYNNVYHFHGLEELQVAW

>CYP202A19(Mesop_4874)*Mesorhizobium opportunistum*

MTTLPYLAFDPATRHLRLDPHEPAFFLNPYEAYARLHGLSNAFFWEEFGCWCFGGFDDVNRLLRDRRFGRQNP
AGIPDSRGIGQDRSHLKAFDGIEANSMLELEPPVHTRLRTLVNRAFVSRQVERLRPRVEALANELIDRFDPAG
PVDLLPAFAAPLPITIIAEMLGVPVEMGPQLLDWSHQMVAMYIHGRSRETEETANRAAGEFADFLRGYITERR
KNPGDDLLSLLISAQEDGQRLSEDEMVSSAILLLNAGHEATVHQTGNAVRSILAQQGDPRRFFTSPEATAATV
EECLRFDAPLHMFTRYAYQEIEVAPGIVVQPGQTIGLLLGMANHDPRAFADPQDFRPDRTDQKNVSFGAGIHF
CIGAPLARLELQVSLKTLFERQPKLHLAESPRFRDTYHFHGLETLAVGF

>CYP202A20(Mesau_04483)*Mesorhizobium australicum*

MTKSTLSYLAFDPATRQLRLDPREPAFFLNPYEAYGFLHEASNAFFWEEFGFWCFGGFGDVNRLLRDRRFGRQ
NPAGIPDSRGVGQDRTHLKAFDGIEANSMLELEPPVHTRLRTLVNRAFVSRQVERLRPRVEALANELIDRFEP

GKAVDLLPAFASPLPITIIAEMLGVPVEMGPRLLDWSHQMVAMYIHGRTRETEETANRAAGEFSDFLRGYVTE
RRKKPGDDLLSLLISAQEDGQKLSEDELVSSAILLLNAGHEATVHQTGNAVHSILAQGGDPRRFFTSPEATAA
TVEECLRFDAPLHMFMRYAYEKIQIAPGIVVRPGQTIGLLLGMANHDPHAFAEPQTFRPERTDQKNVSFGAGI
HFCIGAPLARLELQVSLRTLFERHPQLHLAEQPRFRDTYHFHGLERLAVVFS

>CYP202A21(Mesci_4424)*Mesorhizobium cicero*

MSKLTPPALAFDPATRHLRLDPHEPAFFQNPYDAYAFLHGLSNAFFWEEFGFWCFGGFDDVNRLLRDRRFGRQ
NPAGIPDSRGIGQDRSHLRAFDGVEANSMLELEPPVHTRLRTLVNRAFVSRQVERLRPRVEALANELIDRFEP
GKTVDLLPAFAAPLPITIIAEMLGVPVDMGPQLLDWSHQMVAMYMHGRTRAIEETANRAAGEFADFLRFHVTE
RRKNPGDDLLSLLISAQEDGKKLSEDEMVSSAILLLNAGHEATVHQTGNAVRSILMQGGDVSRFFGSPEATAA
TVEECLRFDAPLHMFTRYAYQEIEVAPGIVVQPGQTIGLLLGMANHDPRAFAEPQAFRPDRPDQKNVSFGAGI
HFCIGAPLARLELQVSLKTLFERHPKLHLAERPSFRDTYHFHGLERLAVGF

>CYP202A22(IMCC20628_02331)*Hoeflea* sp. IMCC20628

MTSHQLFISQDPREPDFVQNPYRFYADMHRTSPVFYWEEYSMWCVAGYDLANRLLRDRRLGRENRWGAPLNPV
EGRQHLADFDRIESGSLLEREPPAHSRLRTLVNRAFVSRSVERLRPRIHALAQELIDALPAGQAFDLLPYFAT
PIPLTVICELLGVPVARANDLLGWSHAMCEMYVPQRSRETEKRANRASADFGAFLTAHIEEHRHTNADDLLSA
LIAVRDTGEKLSDDELISTCVLLLNAGHEATVHQTGNAVKTILDQGGDPRRFFETPAASAATVEEALRFDAPL
HMFTRYAYEEIDLGPVTLKPGEQVALLLGAANRDPSAFEAPDQFRPGRADQKNVSFGAGLHFCIGAPLARLEL
QESLAVLFSRLPDLKLTQPPRYRDSYHFHGLESLLARS

>CYP202A23(Rleg_1336)*Rhizobium leguminosarum* bv. trifolii WSM1325

MMIPSFLSIDRANRHVSLDSRNPAFYGDPNAVYAALHAHCPTFYWSEQKQWFFTGYDHVNGLLRDRRFGRQIL
HIASREELGLAEPTPHLASFDLSERYSLLELEPPEHTRLRTLVNRAFVSRHVEKMKPELAELANRLIDGFAEK
REVELLSAFADIIPVTMIARMIGIPEEMGPQLLTWSHAYVRMYMFGRTRGQEEEAERASKEFSDYVKTVIAER
RAEPRDDLLTHMIHTEHKGQYLTEEELVSTTIVLLNAGHEATVHQIGNSVRTILDSGGDPAELFRDEATTERT
VEETLRICAPVHIFQRWALEPAEIDGISFKRGDKVSLILAAANLDPAKFTDPLTFKPDRNEAANLSFGAGIHF
CIGAPLARLELNVVLPILFERLAGLRLAKTPVVKDVYHFHGLDRLDLQW

>CYP202A23(Rleg2_1245)*Rhizobium leguminosarum* bv. trifolii WSM2304

MMIPPFLSIDRSSRHVSLDARNPAFYGDPNAVYAALHAHCPTFYWSEQKQWFFTGYDHVNGLLRDRRFGRQIL
HIASREELGLAEPMPHLASFDLSERYSLLELEPPEHTRLRTLVNRAFVSRHVEKMKPELAELANQLIDGFADK
GEVELLSAFADIIPVTMIARMIGIPEEMGPQLLAWSHAYVRMYMFGRTREQEDEAERAAKEFSDYVRTVIAER
RAEPRDDLLTHMIHTEHKGQYLTEEELISTTIVLLNAGHEATVHQIGNSVRTILESGSDPADLFRDEATTERT
VEETLRICAPVHIFQRWALEPAEIDGVSFQRGDKVSLILAAANLDPAKFTDPLAFRPDRNEAANLSFGAGIHF
CIGAPLARLELNVVLPILFERLAGLKLAKTPVVKDVYHFHGLDRLDLQW

>CYP202A23(RL1686)*Rhizobium leguminosarum* bv. viciae 3841

MPHIFPRPGKSAKPRGMMIPSFLSIDRANRHVSLDSRNPAFYGDPNAVYAALHAHCPTFYWSEQKQWFFTGYD
HVNGLLRDRRFGRQILHIASREELGLAEPMPHLASFDLSERYSLLELEPPEHTRLRTLVNRAFVSRHVEKMKP
ELAELANRLIDGFAEKREVELLSAFADIIPVTMIARMIGIPEEMGPQLLAWSHAYVRMYMFGRTREQEEEAER
AAKEFSDYVKTVITERRATPRDDLLTHMIHTEHKGQYLTEEELVSTTIVLLNAGHEATVHQIGNSVRTILDSG
CDPAELFRDEATTERTVEETLRICAPVHIFQRWALEPAEIDGVSFKRGDKVSLILAAANLDPAKFTDPLTFKP
DRNEAANLSFGAGIHFCIGAPLARLELNVVLPILFERLAGLKMAKTPVVKDVYHFHGLDRLDLQW

>CYP202A23(RLEG3_17090)*Rhizobium leguminosarum* bv. trifoliiWSM1689

MMIPSFLSIDRANRHVSLDSRNPAFYGNPNAVYAALHAHCPTFYWSEQKQWFFTGYDHVNGLLRDRRFGRQIL
HIASREELGLAEPMPHLASFDLSERYSLLELEPPEHTRLRTLVNRAFVSRHVEKMKPELAQLANRLIDGFAEK
HEVELLSAFADIIPVTMIARMIGIPEEMGPQLLNWSHAYVRMYMFGRTREQEEEAERAAKEFSDYVKTVITER
RAEPRDDLLTHMIHTEHKGQYLTEEELVSTTIVLLNAGHEATVHQIGNSVRTILDSGSDPAELFRDEATTERT
VEETLRICAPVHIFQRWALEPAEIDGVSFKRGDKVSLILAAANLDPAKFTDPLTFKPYRNEAANLSFGAGIHF
CIGAPLARLELNVVLPILFERLVGLRLAKKPMVQDVYHFHGLDRLDLQW

>CYP202A23(RHECIAT_CH0001657)*Rhizobium etli* CIAT 652

MTIPPFLSIDPALRHVSLDARNPAFYGNPNAVYAALHAHCPTFYWTEQKQWFFTGYDHVNALLRDRRFGRQIL
HIASREELGLAEPMPHLESFDLSERYSLLELEPPEHTRLRTLVNRAFISRHVEKMKPELAELANRLIDGFADK
REVELLSAFADIIPVTMIARMIGIPEEMGPQLLAWSHAYVRMYMFGRTREQEDEAERAAKEFSDYVRTVIAER
RAEPRDDLLTHMIHTEHKGQYLTEEELISTTIVLLNAGHEATVHQIGNSVRTILDSGTYPAELFRDEATTERT
VEETLRICAPVHIFQRWALEPAEIDGVSFERGDKVSLILAAANLDPAKFTDPLAFRPDRNEATNLSFGAGIHF
CIGAPLARLELNVVLPILFERLAGLRLAKTPDVKDVYHFHGLERLDLQW

>CYP202A23(RHE_CH01585)*Rhizobium etli* CFN 42

MMIPPFLSIDRASRHVSLDARNPDFYGNPNAVYAALHAHCPTFYWTEQKQWFFTGYDHVNALLRDRRFGRQIL
HIARREELGLAEPMPHLESFDLSERYSLLELEPPEHTRLRTLVNRAFVSRHVEKMKPELAELANRLIDGFADK
REVELLSAFADIIPVTMIARMIGIPEEMGPQLLAWSHAYVRMYMFGRTREQEDEAERAAKEFSDYVRTVIAER
RNAPRDDLLTHMIHTEHKGQYLTEEELISTTIVLLNAGHEATVHQIGNSVRTILDSGSDPAVLFRDEATTERT
VEETLRICAPVHIFQRWALEPAEIDGVSFKRGDKVSLILAAANVDPAKFTDPLTFRPDRNEAPNLSFGAGIHF
CIGAPLARLELNVVLPILFERLAGLSLAKTPVVKDVYHFHGLDRLDLLW

>CYP202A23(REMIM1_CH01603)*Rhizobium etli*

MTIPPFLSIDRASRHVSLDARNPDFYGNPNAVYAALHAHCPTFYWTEQKQWFFTGYDHVNALLRDRRFGRQIL
HIARREELGLAEPMPHLESFDLSERYSLLELEPPEHTRLRTLVNRAFVSRHVEKMKPELAELANRLIDGFADK
REVELLSAFADIIPVTMIARMIGIPEEMGPQLLAWSHAYVRMYMFGRTREQEDEAERAAKEFSDYVRTVIAER
RNAPRDDLLTHMIHTEHKGQYLTEEELISTTIVLLNAGHEATVHQIGNSVRTILDSGSDPAVLFRDEATTERT
VEETLRICAPVHIFQRWALEPAEIDGVSFKRGDKVSLILAAANVDPAKFTDPLTFRPDRNEAPNLSFGAGIHF
CIGAPLARLELNVVLPILFERLAGLGLAKTPVVKDVYHFHGLDRLDLLW

>CYP202A23(RLEG12_17290)*Rhizobium leguminosarum* bv. trifolii CB782

MMIPPFLSIDPARRHVSLDARNPGFYGNPNAVYAVLHAHCPTFYWSEQKQYFFTGYDHVNGLLRDRRFGRQIL
HIASREELGLAEPMPHLASFDLSERYSLLELEPPEHTRLRTLVNRAFVSRHVEKMKPELAELANRLIDGFADK
GEVELLSAFADIIPVTMIARMIGIPEEMGPQLLAWSHAYVRMYMFGRTREQEDEAERAAKEYSDYVRTVIAER
RAAPRDDLLTHMIHTEHKGQYLTEEELISTTIVLLNAGHEATVHQIGNSVRTILDSGSNPAELFRDEATTART
VEETLRICAPVHIFQRWALEPAEIDGVTFRRGDKVSLILAAANLDPAKFTDPLAFRPDRNEAANLSFGAGIHF
CIGAPLARLELNVVLPILFERLAGLRLARTPVVKDVYHFHGLDRLDLQW

>CYP202A23(IE4803_CH01583)*Rhizobium etli* bv. phaseoli IE4803

MTIPPFLSIDPASRHISLDARNPAFYGNPNAVYAALHAHCPTFYWAEQKQWFFTGYDHVNGLLRDRRFGRQIL
HIASREELGLAEPMPHLASFDLSERYSLLELEPPEHTRLRTLVNRAFVSRHVEKMKPELAELANRLIDGFADK
REVELLSAFADIIPVTMIARMIGIPEEMGPQLLAWSHAYVRMYMFGRTRAQEEEAERAAKEFSDYVRTVIAER
RAEPRDDLLTHMIHTEHKGQYLTEEELISTTIVLLNAGHEATVHQIGNSVRTILDSGLDPATLFRDEATTERT
VEETLRICAPVHIFQRWALEPAEIDGVSFKRGDKVSLILAAANLDPAKFTDPLAFRPDRNEAPNLSFGAGIHF
CIGAPLARLELNIVLPVLFERLAGLRLAKSPVVKDVYHFHGLERLNLVW

>CYP202A23(IE4771_CH01635)*Rhizobium etli* bv. mimosae IE4771

MTIPPFLSIDPASRHVSLDARNPAFYGNPNAVYAALHAHCPTFYWAEQKQWFFTGYDHVNGLLRDRRFGRQIL
HIASREELGLAEPMPHLASFDLSERYSLLELEPPEHTRLRTLVNRAFVSRHVEKMKPELAELANRLIDGFADK
REVELLSAFADIIPVTMIARMIGIPEEMGPQLLAWSHAYVRMYMFGRTRAQEEEAERAAKEFSDYVRTVIAER
RAEPRDDLLTHMIHTEHKGQYLTEEELISTTIVLLNAGHEATVHQIGNSVRTILDSGLDPATLFRDEATTERT
VEETLRICAPVHIFQRWALEPAEIDGVSFKRGDKVSLILAAANLDPARFTDPLAFRPDRNEAPNLSFGAGIHF
CIGAPLARLELNIVLPVLFERLAGLRLAKSPVVKDVYHFHGLERLNLVW

>CYP202A23(AMC79_CH01615)*Rhizobium phaseoli*

MTIPPFLSIDPALRHVSLDARDPAFYGNPNAVYAALHAHCPTFYWTEQKQWFFTGYDHVNALLRDRRFGRQIL
HIASREELGLAEPMPHLESFDLSERYSLLELEPPEHTRLRTLVNRAFVSRHVEKMKPELAELANRLIDGFADK
REVELLTAFADIIPVTMIARMIGIPEEMGPQLLAWSHAYVRMYMFGRTREQEDEAERAAKEFSDYVRTVIAER
RAEPRDDLLTHMIHTEHKGQYLTEEELISTTIVLLNAGHEATVHQIGNSVHTILGSGADPVELFRDEATTERT
VEETLRICAPVHIFQRWALEPAEIDGVSFKRGDKVSLILAAANLDPAKFTDPLAFRPDRNEATNLSFGAGIHF
CIGAPLARLELNVVLPILFERLAGLRLAKTPDVKDVYHFHGLERLDLQW

>CYP202A23(AMJ98_CH01614)*Rhizobium* sp. N1341

MTIPPFFSIDPALRHVSLDARNPAFYGNPNAVYAALHAHCPTFYWTEQKQWFFTGYDHVNGLLRDRRFGRQIL
HIASREELGLAEPMAHLESFDLSERYSLLELEPPEHTRLRTLVNRAFVSRHVEKMKPELTELANRLIDGFADK
GEVELLSAFADIIPVTMIARMIGIPEEMGPQLLAWSHAYVRMYMFGRTRRQEEEAERAAREFSDYVRTVIAER
RAEPRDDLLTHMIHTEHKGQYLTEEELISTTIVLLNAGHEATVHQIGNSVRTILDSGCDPAELFRDEAATERT
VEETLRICAPVHIFQRWALEPAEIDGVSFKRGDKVSLILAAANLDPAKFTDPLAFRPDRNEAPNLSFGAGIHF
CIGAPLARLELNVVLPILFERLAGLRLAKTPVVKDVYHFHGLDRLDLRW

>CYP202A23(AMK02_CH01623)*Rhizobium* sp. N731

MTIPPFFSIDPALRHVSLDARNPAFYGNPNAVYAALHAHCPTFYWTEQKQWFFTGYDHVNGLLRDRRFGRQIL
HIASREELGLAEPMAHIESFDLSERYSLLELEPPEHTRLRTLVNRAFVSRHVEKMKPELTELANRLIDGFADK
GEVELLSAFADIIPVTMIARMIGIPEEMGPQLLAWSHAYVRMYMFGRTRRQEEEAERAAREFSDYVRTVIAER
RAEPRDDLLTHMIHTEHKGQYLTEEELISTTIVLLNAGHEATVHQIGNSVRTILDSGCDPAELFRDEAAAERT
VEETLRICAPVHIFQRWALEPAEIDGVSFKRGDKVSLILAAANLDPAKFTDPLAFRPDRNEAPNLSFGAGIHF
CIGAPLARLELNVVLPILFERLAGLRLAKTPVVKDVYHFHGLDRLDLRW

>CYP202A24(RGR602_CH01450)*Rhizobium gallicum*

MMIAPFFSIDPATRRVSLDSSDPAFYGNPNEVYKALHCHCPTFYWEQQKQWFFTGYDHVNGLLRDRRFGRQIL
HIATREELGLAEPEQHLANFDLAERYSLLELEPPEHTRLRTLVNRAFVSRHVEKMKPELAELANKLIDGFKDK
GEVELLSAFADIIPVTMIARMIGIREEMGPQLLAWSHAYIRMYLFGRTREDEDGAERAASDFSDYVKTVIAER
RANPRDDLLTHMIHTEHKGQYLTDAELVSTTIVLLNAGHEATVHQIGNSVRVILESGCDPAELFKDEAATECT
VEETLRICAPVHIFQRWALEPVEIDGISFKRGDKVSLILAAANLDPARFTDPLSFRPDRNEAPNLSFGAGIHF
CIGAPLARLELNVVLPILFERLPGLKLAKTPSVKDVYHFHGLDALNLAW

>CYP202A25(Arad_1827)*Agrobacterium radiobacter*

MTISSVFSIDPVSRRLSLNGRDPAFYSNPNAVYAALHAECPTFYWEEQRRWYFIGYDHVNGLLRDRRFGRQIL
HIANREELGMPAPLPHLKNFDLSEQHSLLEIEPPEHTRLRTLVNRAFVSRHVEKMKPELAELANRLIDKFEKN
GEVELLSAFADIIPVTMIARMIGIPEEMGPQLLKWSHAYVRMYMFGRTREDEDAADQAAKEFSDYVKSVIRER
RANPRDDLLTHMITTEHKSQYLTEDELVSTTIVLLNAGHEATVHQIGNSVRVILESGYAPSALFKDEATTERT
VEETLRICAPVHIFDRWCLEPTEIDGVSFQRGDKVAMILAAANLDPKKFSDPLTFKPDRNEAPNLSFGAGIHF
CIGAPLARLELNVVLPILFERLPGLKLQKTPEVKDVYHFHGLDKLELSW

>CYP202A26(Avi_1694)*Agrobacterium vitis*

MPHSSFFSVDPHQRSVSLDTGHPGFFEAPNAVYAMLHAQAPTFFWREQQQWFFTGYDLVNGLLRDKRFGRQIL
QIASREELGWPEPAAHTRAFDAAEAWSLLELEPPEHTRLRTLVNRAFVSRHVEKMTPDILALANSLIDDFEAQ
GKTELLSSFAEIIPVTMIARMIGIPDEMGPQLLAWSHDYVGMYVFNRTRAHEDAAERAAREFSDYLRGVIAER
RSEPRDDLLSHMIHTEHKGQYLTDDELISTTVVLLNAGHEATVHQIGNAVRIILESGLDPTGLFGDTAATERT
VEECLRICAPVHIFQRYCLEPCEIDGVSFSQGDKIGLILAAANLDPRKFPDPLAFKPQRNDGANLSFGAGIHF
CIGAPLARLELSLVLPLLFQRLPGLALAGTPKVKDLYHFHGLEKLDLVW

>CYP202A27(BSY240_1120)*Agrobacterium* sp. RAC06

MTQTSFLSIDPATRRVSLDARDPAFYSDPNRTYAALHDSCPTFYWEEQKQWFFTGYDHVNALLRDRRFGRQIL
HIATREELGMPEPAEHTRHFDKAEEHSLLEIEPPEHTRLRTLVNRAFVSRHVEKMTPEIELLANRLIDDFEKD
GKTELLSTFADIIPVTMIARMIGIPEDMGPQLLKWSHDYVGMYMFKRTRADEEAADRSAKEFADYVRTVIAER
RANPSDDLLSHMIHTEHKGQFLTEDELISTTIVLLNAGHEATVHQIGNSVRVILDSGIDPSTLFSDEKTTERT

VEETLRICAPVHIFQRWCLEPCEIDGVSFQRGDKVSLILAAANLDPQKFPEPLAFKPSREEGANLSFGAGIHF
CIGAPLARLELNIVLPLLFKRLPGLKIAETPTVKDVYHFHGLERLDLTW

>CYP202A28(BSY16_1583)*Sinorhizobium* sp. RAC02

MTTTDFPFLSIDPASRRVSLDGRNPDFYRDPNPVYAALHAHCPTFWWEEQKMWYFTGYDHVNGLLRDRRFGRQ
ILHVATREELGMPEPQAHLAHFDAAEAWSLLELEPPEHTRLRTLVNRAFVSRIIDRLTPEITDLCHQAIDRFE
KDGKVELLSAFADILPVTMIARMIGIPDEMGPQLLKWSHAYVRMYMFGRTPEDELNADKAAQEFSDYVRSVIA
ERRAEPRDDLLSHMVHTEHRGQLLTEDELISTTIVLLNAGHEATVHQIGNAVRTILENNADPALLFADEKSTE
RTVEECLRISAPVHIFDRFALEDVELDGIQFRRGDKVAMILAAANLDPKKFSDPLVFKPARNEGVNLSFGAGI
HFCIGAPLARLELNIALPILFQRLPRLKIAKKPVVKDVYHFHGLETLKLEW

>CYP202A29(RG540_CH11740)*Neorhizobium galegae* bv. orientalis HAMBI

MTILPDFLHINPQTRHVSLDGRNPAFYSDPNRVYALLHEHCPTFYWEEQRQWFFTGYDHVNGLLRDRRFGRQI
LHIASREELGLPEPLAHLENFNLAERYSLLELEPPEHTRLRTLVNRAFVSRHIEKLRPEIAKLAERLIDGFEG
QGETELLSSFADIIPVTMIARMIGIPDEMGPQLLKWSHAYVGMYMFKRTRQDEYAADRAAGEFSDYVKGLIAE
RRREPREDLLTHMIHTEHKGQYLTDDELVSTTIVLLNAGHEATVHQIGNSVRVILESGLPPADLFRDEAATER
TVEETLRICAPVHIFQRWALEDVEIDGLSFKRGDKVSLILAAANLDPNKFSDPLAFKPDRQEAQNLSFGAGIH
FCIGAPLARLELNTVLPILFRRLPGMRITRTPVVKDVYHFHGLERLDLAWRQGSPAL

>CYP202A29(RG1141_CH11450)*Neorhizobium galegae* bv. officinalis bv.
officinalis HAMBI 1141

MTALPDFLRIDPDTRRVSLDGRNPAFYSDPNRVYALLHEHCPTFYWEEQRQWFFTGYDHVNGLLRDRRFGRQI
LHIANREELGLPEPLPHLENFDLAERYSLLELEPPEHTRLRALVNRAFVSRHIERLRPEIAELAEGLIDGFEG
QGETELLSAFADIIPVTMIARMIGIPDAMGPQLLKWSHAYVGMYMFKRSADDEHAADRAAEEFSDYVKGLISE
RRREPREDLLTHMIHTEHKGQYLTDDELVSTTIVLLNAGHEATVHQIGNSVRVILESGLSPADLFRDEAATER
TVEETLRICAPVHIFQRWALEDVEIDGLSFKRGDKVSLILAAANLDPKKFSDPLVFKSDRQEAANLSFGAGIH
FCIGAPLARLELNTVLPILFRRLPGMRITRTPVVKDVYHFHGLERLDLAW

>CYP202A30(NT26_1249)*Rhizobium* sp. NT-26

MTDIPSFLPIDPTTRRVSLDGRDPAFYGDPNRVYAALHQHCPTFYWEEQRQWFFTGYHHVNALLRDRRFGRQI
LHIASREDLGLPEPQPHLANFDLVERHSLLEIEPPEHTRLRTLVNRAFVSRHIERLRPEIAVLAEQLIDGFEK
DGKTELLSSFADIIPVTMIARMIGIPDEMGPQLLKWSHDYVGMYMFKRTREDELAADRSAREFADYVKTLIAE
RRNAPRDDLLSHMIHTEHKGQYLSEDELVSTTIVLLNAGHEATVHQIGNSVRVILESGLPPAEMFRDEVATER
TVEETLRICAPVHIFRRWALEDVEVDGVVLKRGDKVSLILAAANLDPNKFAEPLVFNPERQEAPNLSFGAGIH
FCIGAPLARLELKTVLPILFRRLPGLRMAKTPRVKDVYHFHGLERLDLAW

>CYP202B1(RSP_1946)*Rhodobacter sphaeroides* 2.4.1

MQTLSQSPHDRRFLRNPYRFYREARAAGPFFHWEELGLVCTTSYAAANAILRDRRFGREVPPGRASAVPDHLA
PFAAVEAHSMLELEPPRHTRLRNLVLRAFTSRRIGTMQPEVAALSESLVAAVPEGPFDLLPAFSQRLPITLIA
RLIGIPESLAPELLRWSSAMVAMYQAGRTRKTEERAALAAADFSDFLRLHIEARRHAPADDLLTHLIAAEADG
QQLSTDEIVSTCILILNAGHEAAVHAIGNAAAVLLRHRTPPEALAPPHLLGTVEELLRFDPPLHLFRRMAYER
VEIMGRTIEEGCEVALLLGAANRDPGPWERPDRFLWNRPEKTHLAFGAGLHFCLGAPLARLELATALPILFGR
LPNLQLVKPPSYGDSWHFRGLERLIVSA

>CYP202B1(RSKD131_0258)*Rhodobacter sphaeroides* KD131

MQTLSQSPHDRRFLRNPYRFYREARAAGPFFHWEELGLVCTTSYAAANAILRDRRFGREVPPGRASAVPDHLA
PFAAVEAHSMLELEPPRHTRLRNLVLRAFTSRRIGTMQPEIAALSESLVAAVPEGPFDLLPAFSQRLPITLIA
RLIGIPESLAPELLRWSSAMVAMYQAGRTRKTEERAALAAADFSDFLRLHIEARRHAPADDLLTHLIAAEADG
QQLSTDEIVSTCILILNAGHEAAVHAIGNAVAVLLRHRTPPEALAPPHLLGTVEELLRFDPPLHLFRRMAYER
VEIMGRTIEEGCEVALLLGAANRDPGPWERPDRFLWNRPEKTHLAFGAGLHFCLGAPLARLELATALPILFGR
LPNLQLVKPPSYGDSWHFRGLERLIVSA

>CYP202B1(Rsph17029_0597)*Rhodobacter sphaeroides* ATCC 17029

MQTLSQSPHDRRFLRNPYRFYREARAAGPFFHWEELGLVCTTSYAAANAILRDRRFGREVPPGRAAAVPDHLA
PFAAVEAHSMLELEPPRHTRLRNLVLRAFTSRRIGTMQPEVAALSESLVAAVPEGPFDLLPAFSQRLPITLIA
RLIGIPESLAPELLRWSSAMVAMYQAGRTRKTEERAALAAVDFSDFLRLHIEARRHAPADDLLTHLIAAEADG
QQLSTDEIVSTCILILNAGHEAAVHAIGNAVAVLLRHRTPPEALAPPHLLGTVEELLRFDPPLHLFRRMAYER
VEIMGRTIEEGCEVALLLGAANRDPGPWERPDRFLWNRPEKTHLAFGAGLHFCLGAPLARLELATALPILFGR
LPNLQLVKPPSYGDSWHFRGLERLIVSA

>CYP202B10(Rsph17025_0342)*Rhodobacter sphaeroides* ATCC 17025

MARLRRQRGTLARPMKTLSQSPHDRRFLRDPYRFYRQARTAGPFFRWEELDVICTTSYAAANAILRDRRFGRE
VPAERRAPVPAHLAPFAAVAEHSMLELEPPRHTRLRNLVLRAFTTRRLGSMQPQIARLAHGLIDDMPGGPFDL
LPAFSQRLPITLIARLIGIPETLAPELLRWSSAMVAMYQAGRNRQTEERAAEAAAAFSDFLRLHIADRRDRPA
DDLLTHLIAAEADGQQLSTDEIVSTCILILNAGHEAAVHAIGNAVATLLQHRTPPEALGPARIAATVEEMLRF
DPPLHLFRRIAYAPVEIMGQTFAEGEEVALLLAAANRDPGPWERPDRFLWNRPEKPHLAFGAGLHFCLGAALA
RLELAMAVPILFTRLPGLHLVKPPRWSDSWHFRGLERLIVAP

>CYP202B11(LOKVESSMR4R_01170) *Yoonia vestfoldensis*

MQQLTQSPVDPAFVQNPYPFYDRARQTGDFFWWNDYAMPCAVSHKAVHAVLRDRRMGRECPSELAPAIPDALR
PFYEIEAHSMLELEPPRHTRLRALVLRAFTSRTIAALAPQITQLCHDLIDRFPDEPFDLLTAYAQPVPVVTIA
RLLGVPEDRADDLLRWSNAMVAMYQARRTPEIEAAAIAASTDFSDFMRGYIATRRKTPADDLISTLIAAEEQG
EKLTTDELITTCILLLNAGHEATVHTLGNGTKTLLEQNIRGITDACVEEIIRHDPPLHMFTRWVYEDVQIGDQ
HFKRGDQIACLLGAANRDPAVYPDPARFDPARSGPQNTSFGGGIHFCVGAPLARLELKIALDVLFTRCPDLRL
TAAPGYGDTYHFHGLTRLMVAAR

>CYP202B12(OSB_17550)*Octadecabacter temperatus*

MQSLSQSPTDPTFVQNPYPFYDLARAGRDLFFWEDYDAVCAVSHAAVAACLKDKRLGREVPAEFARAIPEHLA
PFYAVEAHSMLELNPPRHTRLRGLVLRAFTSRRIAELEPEIETLCHQLIDAFPTSEFDLLDAYARTVPVIIIA
RLLGVPENRADDLLAWSNAMVAMYVSGRTRAIEDHAIEATEAFVTFMRAHVDEKRKAPADDLMSHLIAAEEGG
EKLSTDELITTCILLLNAGHEATVHTLGNTVKTLLERQIRTADEKIVEEALRFDPPLHMFDRYVIEDCELFGH
AFKRGDTVKCLLGAANRDPAPYPDPNTFNPTRTGPVNVAFGGGIHFCVGAPLARLELRVALQVLFERCPNLAL
ATPPQYGNTYHFHGLEALRVAT

>CYP202B13(BVG79_01175)*Ketogulonicigenium robustum*

MDRISQSPTDPAFVANPYAFYDKARAAGDMFYWTDYDLPCAVTHRAVSALLRDRRLGREAPPECAPTYPDFTR
DFYALEAHSMLEVEPPRHTRLRGLVLRAFTSRRIAAMAPEIETLCHSLIDAFPPGPFDLLTHYAQHVPVTVIA
RLLGVPDSTAPRLLDWSNRMVGMYQARATEAVQRDANAAAAEFRAFMVDYIAQRRVTPGNDLLTELIAAEEDG
EKLTTDELITTCILLLNAGHEATVHTIGNGIKTLIEQGKPAIDETSVEEIIRFDPPLHMFLRWVYEDVDLFGH
TFRRGDRIACLLAAANHDPAAYDAPATFNPARKGPQNTSFGAGIHFCVGAPLARLELVIALRTLMARLPALSL
AEAPRYSNVYHFHGLQRLMVTAR

>CYP202B14(OA238_c12900)*Octadecabacter arcticus*

MQTLTQSPTDPAFVQDPYPFYDSARSAGDWGTGDLFYWTDYGAVCAVSHAAVAACLKDKRMGREIPTEFAPNI
PAHIAPFYAVESHSMLELNPPRHTRLRGLVLRAFTSRRIAELEPEITALCHKLIDQFPSGEFDLLDAYARTVP
VIIIARLLGVPETRVQDLLAWSNAMVAMYVSGRTRDIEDKAVAATERFVAFLRGFVDEKRQTPADDLLSHLIA
AEEDGEKLTTDELITTCILLLNAGHEATVHTLGNTVKTLLDRDIRAADAQIVEEALRFDPPLHMFDRYVIEDC
EVFGHAFKRGDTVKCLLGAANRDPVAYPDPNRFDPSRKGPVNVAFGGGIHFCVGAPLARLELRVALQVLFDRC
PTIRLTAPPQYGDTYHFHGLDALHVRT

>CYP202B15(MALG_01977)*Marinovum algicola*

MAELSQSPTDPGFVQNPYPFYAEARARGAVHQWRDYGIHCAFSHAAVNAALRDSRMGREAPDELKEPRPAHLR
DFYAIDDNSMLELEAPRHTRLRKLVLHAFTARGIAGLAPEIEALCHHLIDAFPEGDFDLLPQYATQVPVIVIA

RLLGVPDDMADRLLAWSNDMVAMYQARRDRTIEDGANAAAREFSAFLADYIATRRRTPSDDLITRLIAAEADG
EKLSLDELIATCVLVLNAGHEATVHTLGNGVKAMLETGMRPEWTSDSMIDATVEEILRFDPPLHMFTRWVYED
LEIGRSPLRRGDRIGLLLGAAGRDPEMWVDPDRFDPNRHLKPHVAFGAGRHFCVGAPLARLELRIALQVLFAR
LPGLRLAASPRYANLYHFHGLERLMLCAQ

>CYP202B16(METH_11685)*Leisingera methylohalidivorans*

MKTLTQSPTGPAFVQDPYLFYAAAREQGQLHHWQDYGMAAAFGHSAVHMLLRDRRFGREVPEEMRQDGPAHLA
PFLTAEAHSMLDAEPPRHTRLRRLVLRAFTSREIAALQPGLEVLCHRLIDAFPADPFDLLDAYCTQVPVIAIC
RLLGVPEEMAPQLLDWSHRMVAMYQASRTEQTEHAAAEASQQFTEFLRGYIGKRRNDPRDDLITRLIAAEEEG
DKLSTDELIGTCILLLNAGHEATVHSLGNGVKTMLQQGWQPDWLAPDGIEGLVEEILRFDPPLHMFTRYAYEE
AEVFGHTFQRGDQVALLLAAANRDPSALDRPEVFDPSRPPKVNKSFGGGLHFCVGAPLARLEMQIALPILFGR
CPDLKLAAEPEYSNTYHFHGLTRLLVSS

>CYP202B17(PGA2_c19550)*Phaeobacter inhibens* 2.10

MKTLTQSPTDPDFVQSPYGFYAKARANGHLHYWQDYKMVAAFSHPAVHTLLRDRRFGREIPPEMARESPAHLA
PFLQVEAHSLLDAEPPRHTRLRRLVLRAFTSREIKALAPGLDTLCHELIDAFPQDAPFDLLTAYCTQVPVIAI
CRLLGVPTDMAPQLLDWSHKMVAMYQASKTVETEHAAARASQEFIDFLTTYVDERRKTPGEDLITRLIQAEED
GESLSTDELIGTCILLLNAGHEATVHSLGNGVKTMLQQGWDKSWLAPDGIDGLVEEILRYDPPLHMFTRYAYE
EVELFGHTFQRGDEVALLLAAANRDPEMLEDPERFDPTRPAKVNTSFGAGLHFCVGAPLARMEMQIALPILFE
RCPDLKLAAAPEYNNTYHFHGLTGLMVQV

>CYP202B17(Gal_01332)*Phaeobacter gallaeciensis* DSM 26640

MKTLTQSPTDPDFVQSPYGFYAEARAMGHLHHWQDYNMVAAFSHPAVHTLLRDRRFGREIPPEMARESPAHLA
PFLQVEAHSLLDAEPPRHTRLRRLVLRAFTSREIKALAPGLDTLCHELIDAFPTDAPFDLLTTYCTQVPVIAI
CRLLGVPTEMAPQLLDWSHKMVAMYQASKTVETEHAAAKASQEFIDFLTAYVDERRKTPGEDLITRLIQAEED
GESLSTDELIGTCILLLNAGHEATVHSLGNGVKTMLQQGWDQSWLAPDGIDGLVEEILRYDPPLHMFTRYAYE
EIELFGHTFQRGDEVALLLAAANRDPEMLEDPERFDPTRPAKVNTSFGAGLHFCVGAPLARMEMQIALPILFE
RCPDLTLAAAPEYNNSYHFHGLTRLMVQF

>CYP202B17(PGA1_c20710)*Phaeobacter inhibens* DSM 17395

MKTLTQSPTDPDFVQSPYGFYAEARAMGHLHHWQDYKMVAAFSHPAVHTLLRDRRFGREIPPEMARESPAHLA
PFLQVEAHSLLDAEPPRHTRLRRLVLRAFTSREIKALAPGLETLCHELIDAFPKDAPFDLLTTYCTQVPVIAI
CRLLGVPTEMAPQLLDWSHKMVAMYQASKTVETEHAAARASQEFIDFLTTYVDERRKTPGEDLITRLIQAEED
GESLSTDELIGTCILLLNAGHEATVHSLGNGVKTMLQQGWDKSWLAPDGIDGLVEEILRYDPPLHMFTRYAYE
EIELFGHTFQRGDEVALLLAAANRDPEMLEDPECFDPTRPAKVNTSFGAGLHFCVGAPLARMEMQIALPILFE
RCPDLKLAAAPEYNNTYHFHGLTRLMVQV

>CYP202B17(PhaeoP97_02067) *Phaeobacter porticola*

MGHLHHWQDYKMVAAFSHPAVHTLLRDRRFGREIPPEMARESPAHLAPFLQVEAHSLLDAEPPRHTRLRRLVL
RAFTSREIKALAPGLETLCHELIDAFPKDAPFDLLTTYCTQVPVIAICRLLGVPTEMAPQLLDWSHKMVAMYQ
ASKTVETEHAAARASQEFIDFLTTYVDERRKTPGEDLITRLIQAEEDGESLSTDELIGTCILLLNAGHEATVH
SLGNGVKTMLQQGWDKSWLAPDGIDGLVEEILRYDPPLHMFTRYAYEEIELFGHTFQRGDEVALLLAAANRDP
EVLEDPERFDPTRPAKVNTSFGAGLHFCVGAPLARMEMQIALPILFERCPDLKLAAAPEYNNTYHFHGLTRLM
VQV

>CYP202B18(TM1040_1816)*Ruegeria* sp. TM1040

MRHITKPPLPRPETSAKTDFMKTLTQSPTDPDFVQNPYPFYDAARAAGKLGYWEEYKMMAAFGHDVVHMLLRD
RRFGREIPAEMATEGPAHLAPFLQVEAHSLLDAEPPRHTRLRKLVLRAFTSREIKALAPGLETLCHELINAFP
KDAPFDLLSSYCTQVPVIAICRLLGVPEAMAPQLLDWSHKMVAMYQASKTTETEHAAAQASAEFIAFLTDYVD
ERRKTPGEDLITRLIQAEEDGESLSTDELIGTCILLLNAGHEATVHSLGNGVKTMLEQGWSSEWLQPDGISGL
VEEILRYDPPLHMFTRYAYEEIEIMGHRFRRGDQVALMLAAANRDPEALERADQFDPTRPPKVNKSFGGGLHF
CVGAPLARMEMEIALPILFERCPELKLAEPPQYSNTYHFHGLTRLWVQTR

>CYP202B19(RCA23_c09530)*Planktomarina temperate*

MKIFKQSPTDTSFVQNPYPAYDRARAMGDLVWWDDYKMPAAVSYRAVRGLLTNRKFGREALTAPQCPAHLADW
QANESHSMLELEPPRHSRLRGLVLRAFTSKKVAAMAPQITETCHRLIDAFPAQPFDFLNAFARPLPVHIIAKL
LGVPTDRSSDLLSWSNAMVAMYQSNRSYEVEIAANQATLEFTAFLDSYIAHRRSQPRDDLLSDLIAAEEAGEK
LTGAELISTCILLLNAGHEATVHTLGNALKTLLPSPHREVTPQLVEEVLRFDPPLHLFTRFAYEDTTCFGFDI
HKGQEVACLLAAANRDPAFVENPHVFDPHRKSTAHQSFGAGLHFCVGAPLARLEISLSLQLLFDRCPNLHISQ
APTYANSYHFHGLDALMVQT

>CYP202B20(Dshi_1193)*Dinoroseobacter shibae*

MGAGLPRVTQDPTDAAFVQNPYPVYDRMRALGDLVWWDAYAMPVTPTYAATGAILKDRRFGREPLSPPPVPDH
LRDFYAVEAHSMLELEPPRHTRLRGLVTRAFTSARIAALAPGIEALCHALIDAFPDTPFDLLPAYCTQVPVRI
IARLLGVPEDTAPQLLAWSNAMVGMYQAGRSRAMEEAANTAAHDFRAWLLDLIDARRASPADDLLSALIAAQA
EDGKLTRDELVTTVILLLNAGHEATVHTLGNGVKTLLETGLRQITPATVEEVLRFDPPLHLFTRIARQEVEVF
GHRFAPGDTVGCLLAAAGRDPGFAPDPHRFDPTRPTKPPHMAFGAGLHFCVGAPLARLEIEIALRVLFARCPG
LRLAGAPVYAPIYHFHGLQALRVSR

>CYP202B21(P73_1592)*Celeribacter indicus*

MKRFSMPPSDPAFVQDPYPFYDRLRAGGRMVHWEELDLPVIAGHDGVSMALRDTRFGREAPEGFAPAIPDHLR
PFYEIEAHSMLELEGERHRRLRSLVLRAFTSRRVRALLPDLEEITDGLIDRFPAGEAFDLLPAFCQQVPIRVI
SRLLGVPEEMGPRLLGWSHAMVAVYTPTRTRAMEEAAARAATEFAEFLRGYVEERRARPADDLITHLIAAESE
GERLSTDELISTCVLLLNAGHEATVHALGLAVKTLLERRTPPAFLGADRIDGTVEELLRFDPPLHLFQRWVNE
DTVFLGQELKRGDRIGLLLGAANRDDDAWDDPHAFDPARKVKQNMSFGAGLHFCVGAPLARLEMRVALPRLFD
RCPGLHLTEPPVYADTWHFHGLDRLMVRL

>CYP202B22(IMCC12053_727)*Celeribacter marinus*

MLHFDQDPKDPAFVQDPYGFYARLRDSGPLAYWTELGVPVLTGYDGVSMALRDNRFGREAPQGFARPIPKGLQ
HFYEIDAHSMLELEGERHRRLRGLVLRAFTSRRIKDLMPEITAICDDLLDKIDTHTGETDLLQSFCQPLPVRV
ISRLLGVPEEMCDQLLAWSNAMVAVYTPLRTPEIEAAAEQAAREFSTFMRRYVEARRTTPRDDLITHLIAAEE
DGEKLSTDELISTCILLLNAGHEATVHSLGIAVKTLLETETPPDALTDGAIDATLEEVLRYDPPLHFFDRWVY
KDMCFMGHEFKRGDKVGLLLASANRDETAWDEADRFRPARPIKQNMAFGVGLHFCVGAPLARLEMRVALPRLF
ERYPDLALAAPPDYANTWHFHGLRSLRVRLGQK

>CYP202B23(Jann_1726)*Jannaschia* sp. CCS1

MITLHQSPTDDDFVQNPYPFYAAARAHGPLVHWADYAMPCTTTFAATNAILRDRRFGREPVDALPVPDHLQPF
YDVEAHSMLELEPPRHTRLRGLVLRAFTNRRIKALAPDITQLTHDLIDAFPDGAHDLLDTFARPLPVTLIARL
LGVPEEMAPDLLRWSNAMVMMYQAGRTRSDEDVAVKATEEFVAFMRDYARQRRKDPRDDLITALIAAEEDGEK
LSTDELITTCILLLNAGHEATVHSIGNGVKLCLQQQHRPPRDPAKFTEELLRIDPPLHMFTRTAYEDVTLHGH
TFQRGDQVALLLAAANRDPAAYPDPDTFDPARKGPAQLAFGAGLHFCVGAPLARLELQIALPILFDRCPNLTL
TAPPRYADIYHFHGLDALMVQA

>CYP202B24(RD1_2962)*Roseobacter denitrificans*

MPLLSQADLKAAMQRLIQSPLDPAFVQNPYAFYAEVFTGDTIRYWQDYDMPAIFDAANVQAVLRNRRFGRALP
ADRIGPPRPHLAAFDAVEAVSMLDLEPPDHTRLRGLVLRAFTSRRIKALAPDIHDICDRLIADLPKGGCNLIP
AFCTALPVRIIARLLGVPAERCGDLLRWSNAMVAMYQAGRTLEIEHAANAAASEFTAFLSDYIDIKRRAPQDD
LISHLLLAEENGQKLTRQELIGTCILLLNAGHEATVHTMGNAVKALLEHQTPPQAFTDQNIAATVEEVLRFDP
PLHMFTRYSYEDTEIGDHTIRSGQQVALMLGAAGRDPALCKDPDVFDPFRAPVPHAAFGGGLHFCVGAPLARL
ELQIGLQRLFAARPNLTLSSPPRFANAYHFHGLERLDVTT

>CYP202B25(RLO149_c014660)*Roseobacter litoralis*

MQHLIQSPLDPTFVQNPYALYAEVFTGDTIRYWQDYEMPAIFDAANVQSVLRDRGFGRATPADQIEPLRPHLA
AFNAVEAVSMLDLEPPDHTRLRGLVLRAFTSRKIKALAPDIHDICDTLIAALPRHGCDLIPAFCTALPVRIIA
RLLGVPEDISGDLLRWSNAMVAMYQAGRTLQTEQAANAAAIEFTAFLSDYIDIKRRNPQDDLISQLLRAEDNG
EKLSRDELIGTCILLLNAGHEATVHTMGNAVKALLEHETPAYALTDQNIATTVEEVLRFDPPLHMFTRYSYKD
TTIGGHIIRSGQQVALVLGAAGRDPTLCKDPDVFDPFRAPVAHAAFGGGLHFCVGAPLARLELQIGLQRLFAA
CPHLALSRSPEFANAYHFHGLKRLDVTY

>CYP202B-fragment1(roselon_02610)*Roseibacterium elongatum*

MIVIARLLGVPEGMAPDLLRWSNAMVMMYQAGRTPEDEARAVAATEEFVAFMRDYVRRRRSEPRDDLISALIA
AEADGDKLTTDELITTCILLLNAGHEATVHTMGNGVKLALEMGVRPGTAPEAFVEEVLRLDPPLHLFTRWVYD
EVNLFGHRFRRGDAVGLLLASAGRDADCYAQPDRFQPTRQGPPNLAFGAGLHFCVGAPLARLELGIAMSALFQ
ACSGLRLVEAPRYAEIYHFHGLERLMVTR

>CYP202B-fragment2(AKL17_1763)*Defluviimonas alba*

MPEARAPELLAWSNAMVGMYQARRDRATEDAAVAAAIAFSGYLRELVALRRRTPGDDLISHLIAAGDALSPEE
LISTCILLLNAGHEATVHTLGNGVKTLLEAKVAPEALAPDRIEALVEEILRFDPPLHLFWRWIHEDMEIAGHA
FARGSRIGCLLAGGNRDPQVWERPGRFMPDRLVKPNLAFGGGAHFCIGAPLARLELQVALPILFARLPGMRLK
GKPRYADLYHFHGLETLRVVR

>CYP203A1(RPA1009)*Rhodopseudomonas palustris* CGA009

MFSFDPYSPIVDADPFPLYKTLRDEYPVFWSEPAQMWILSRYLDVAGAGSNWQVFSSAKGNLMTELPNRAGAT
LGTTDPPRHDRLRGLVQHAFMKRNLEALAEPMREIARDAAEALRGRDQFDFISDFSSKFTVRVLFAALGLPMG
DEQTVRDKAVLMVQSDPVTRAKGPEHLAAYAWMQDYASSVIAQRRAEPKNDLISHFSMAEIDGDRLDEREVLL
TTTTLIMAGIESLGGFMSMLALNLADFADARRAVVADPALLPDAVEESLRYNTSAQRFKRCLQSDLTLHGVTM
KAGDFVCLAYGSANRDERQFPNPDVYDVKRKPKGHLGFGGGVHACLGSAIARMAIRIAFDEFHKVVPDYTRTE
QQLNWMPSSTFRSPLRLDFAVEQAASRSAA

>CYP203A1(Rpal_1201)*Rhodopseudomonas palustris* TIE-1

MFSFDPYSPVVDADPFPLYKTLRDEYPVFWSEPAQMWILSRYLDVAGAGSNWQVFSSAKGNLMTELPNRAGAT
LGTTDPPRHDRLRGLVQHAFMKRNLEALAEPMREIARDAAEALRGRDQFDFISDFSSKFTVRVLFAALGLPMG
DEQTVRDKAVLMVQSDPVTRAKGPEHLAAYAWMQEYASGVIAQRRAEPKNDLISHFSMAEIDGDRLDEREVLL
TTTTLIMAGIESLGGFMSMLALNLADFADARRAVVADPALLPDAVEESLRYNTSAQRFKRCLQSDLTLHGVTM
KAGDFVCLAYGSANRDERQFPNPDVYDVKRKPKGHLGFGGGVHACLGSAIARMAIRIAFDEFHKVVPDYTRTE
QQLNWMPSSTFRSPLRLDFAVEQAAARSAA

>CYP203A1(Rpdx1_1170)*Rhodopseudomonas palustris* DX-1

MFSFDPYSPVVDADPFPLYRTLRDEYPVFWSDAAQMWILSRYIDVAGAGTNWQVFSSAKGNLMTELPNRAGAT
LGTTDPPRHDRLRGLVQHAFMKRNLEALADPMREIARDAAEALRGREQFDFISDFSSKFTVRVLFAALGLPIG
DEQTVRDKAVLMVQSDPVSRAKGPEHLAAYAWMQDYASSVIAQRRAEPKNDLISHFSMAEIDGDRLDEREVLL
TTTTLIMAGIESLGGFMSMLALNLADFGDVRRAVVADPALLPDAVEESLRFNTSAQRFKRCLQSDLTLHGVTM
KAGDFVCLAYGSANRDERQFPNADVYDLKRKPKGHLGFGGGVHACLGSAIARMAIRIAFDEFHKVVPDYTRTE
QHLNWMPSSTFRSPLRLDFAVESATARSAA

>CYP203A2(Saro_1648)*Novosphingobium aromaticivorans*

MATVIERPQFRFDPYSPAIDADPFPAYKVLRDEYPCFWSEEAGKWVLSRYDDVLAALQDWRTYSSAKGNLVDE
FPGRAGSTLGSSDPPRHDRLRALIQSAVTKRALEHIIAPARASAQAHLAALADKPVFDLVGDYTSKLTVDLLF
YLFALPDEGAQQVRENAVLMVQTDPVTRQKSPEHLAAFHWMADYAEKLVASRKANPGDDLLSSFITAEIDGEK
LLDKEVQLTVTTLIMAGIESLSGFMAMFGLNLADYPEARSALVADPSLIPDAIEESLRFNTSAQRFKRTLTRD
VELHGQVMKAGDAVILAYGSANRDERMFENPDVYDITRKPRRHLGFGGGVHACLGSMIGRLATQIAYEELLKA
VPDFRRADAPLDWVPSSNFRSPKSLMLEKKA

>CYP203A6(RPB_1062)*Rhodopseudomonas palustris* HaA2

MTRWEVQPHQHPRNRPMFSFDPYSPAVDADPFPLYKTLRDEHPVYWSEPAQMWVLSRYIDVASAGTNWQVFSS
AKGNLMTELPGRAGATLGTTDPPRHDRLRGLVQHAFMKRNLEALAEPMRDIAREAAEALRGREQFDFIADFSS
KFTVRVLFAALGLPLGDEQTVRDKAVLMVQSDPVSRAKGPEHLAAYAWMQDYAAKVIAQRRAEPQNDLISHFS
MAEIDGDKLDEREVLLTTTTLIMAGIESLGGFMSMLALNLADYADARRAVVADPARLPDAVEESLRYNTSAQR
FKRCLQTDLTLHGVTMKAGDFVCLAYGSANRDERQFPNPDVYDLARKPKGHLGFGGGVHGCLGSAIARMAIKI
AFDEFHKVVPDYTRVQPRLDWMPSSTFRSPLRLEMAVERAESRNAA

>CYP203A7(BRADO2350)*Bradyrhizobium* sp. ORS 278

MFTFDPYSPAVDADPFPFYKTLRDEHPCFWSPQAQVWILSRYADVAAAGTNWQTYSSAKGNLMTELPNRAGAT
LGTTDPPRHDRLRGLVQHAFMKRNLESLSGPIRDIARDSAESLRGQERFDFIEAFSSKFTVRVLFAALGLPLG
DEQTVRDKAVLMVQSDPVSRAKGPQHIAAYNWMQDYAAGVIAERRARPQNDLISHFSMAEIDGDKLDEREVLL
TTTTLIMAGIESLGGFMSMLAYNLADHPDARRAVVANPDLLTDAVEESLRFNTSAQRFRRCLQKDLTLHGQTM
REGDFVCLAYGSANRDERQFPNPDVYDITRKPRGHLGFGGGVHACLGSAIARMAIRIAFDELHKVVPEYRRTQ
EQLPWMPSSTFRSPLYLELTVH

>CYP203A7(S58_52490)*Bradyrhizobium oligotrophicum*

MFTFDPYSPAVDADPFPFYKTLRDEHPCFWSPQAQVWILSRYADVAAAGTNWQTYSSAKGNLMTELPNRAGAT
LGTTDPPRHDRLRGLVQHAFMKRNLESLSGPIRDIARDSAEALRGQDRFDFIEAFSSKFTVRVLFAALGLPLG
DEQTVRDKAVLMVQSDPVSRAKGPQHIAAYNWMQDYAAGIIAERRARPQNDLISHFSMAEIDGDKLDEREVLL
TTTTLIMAGIESLGGFMSMLAYNLADHAEARQAVVANPDLLADAVEESLRFNTSAQRFRRCLQKDVPLHGQTM
REGDFVCLAYGSANRDERQFANPDVYDIRRKPRGHLGFGGGVHACLGSAIARMAIKIAFDELHKVVPNYRRTQ
EQLPWMPSSTFRSPMYLELAVH

>CYP203A7(BBta_2708)*Bradyrhizobium* sp. BTAi1

MFTFDPYSAAVDADPFPFYKTLRDAHPCFWSQQAQMWILSRYADVAAAGTNWQTYSSAKGNLMTELPNRAGAT
LGTTDPPRHDRLRGLVQHAFMKRNLESLSGPIRDIARASAEALRGQERFDFIEAFSSKFTVRVLFAALGLPLG
DEQTVRDKAVLMVQSDPVSRAKGPQHIAAYNWMQDYAAGVIAERRARPQNDLISHFSMAEIDGDRLDEREVLL
TTTTLIMAGIESLGGFMSMLAYNLADHPDARRAVVANPDLLADAVEESLRFNTSAQRFRRCLQKDVTLHGQTM
REGDFVCLAYGSANRDERQFPNPDVYDITRKPRGHFGFGGGVHACLGSAIARMAIRIAFDELHKVVPEYRRTQ
EQLPWMPSSTFRSPMYLELTVH

>CYP203A8(PP1Y_Mpl10365)*Novosphingobium* sp. PP1Y

MAVLENPGFTFDPYSPAIDADPFPAYKVLRDKYPCFWSEQAGKWVLSRYDDILAALSNWRVYSSAKGNLVDEF
PGRAGSTLGSSDPPRHDRLRGLIQSAVTKRALDHLIAPAKASARMHLAALDGCDSFDFVGEFGSKVTVDLLFH
LFALPLDNADEVRRNAVLMVQTDPVTRQKAPEHLAAFEWMADYAQDLVHERKKSPGDDLLSNFITAEIDGEKL
LDKEVQLTVTTLIMAGIESLSGFFGMFALNLADYPEARHALVADPSLIADAMEESLRFNTSAQRFKRTLTCDV
ELHGQTMKAGDAVILCYGSANRDERMFVNPDTYDIERRPKRHLGFGGGVHSCLGSMLARVATQAAFEELLTAI
PDFSRFDETLAWTSSSNFRSPKALRLTVG

>CYP203A9(MGMAQ_2657)*Magnetospira* sp. QH-2

MFRFDPYSPAIDADPFPAYKTLRDEHPCFWSEEAGMWVYSRYADIVTALNDWETYSSAKGNLVDELPNRAGNT
LGTTDPPRHDRLRALIQKAVTKRALNYLEAPTQAVCGKHLDALAGAKSFDFVNDFSSKVTVDLLFFLFNMPTE
GQQTVRDKAVLMVQTDPKTRKKGPEHIEAYEWMANYAADLVAERKKNPGDDLLSNFITAEIDGEKLKDKEVQM
TVTTLIMAGIESLSGFMSVFGMNLADHPDARRALVADPSLIPDAMEESLRYNTSAQRFRRCVQKDVAIHGQTM
KAGDFVMLAYGAGNRDERQFANPDVYDIARRPKGHLGFGGGVHACLGTAIARLACKITFETFLGRYPEFTRVQ
EQLPWVPSSNFRSPLKLELAVD

>CYP203A10(AZOBR_p330136)*Azospirillum brasilense* Sp245

MFRFDPYSPAVDADPFPFYKTLRDEHPCFWSEEANMWVLSRYDDIVTALNDWETYSSAKGNLMDEMPNRAGNT
LGTTDPPRHDRLRSIVQFAFTKKAVEGLTEPVRASANRALDAVQGERTFDFVSDVSSKVTVDVLFGLFNLPRE
NERMVRDKAVLMVQSDPRTRQKGPEHLAAFQWMSEYAKELVELRKREPGDDLITALIQAEVAGEKLADREVQM

TITTLIMAGIESLSGFLAMFALNLADHADARRRLAANPALIPDAIEESLRFNTSAQRFRRCLQKDVELHGQTM
RAGDFVCLAYGSGNRDERRFANAHLYDIDRKPKGHLGFGGGVHACLGTAFARLAARVACEEFLKRVPEFVRVQ
DQLPWMPSTTFRSPTRLELAVG

>CYP203A10(ABAZ39_27945)*Azospirillum brasilense* Az39

MFRFDPYSPAVDADPFPFYKTLRDEHPCFWSEEANMWVLSRYDDIVTALNDWETYSSAKGNLMDEMPNRAGNT
LGTTDPPRHDRLRSIVQFAFTKKAVEGLTEPVRASANRALDAVQGERTFDFVSDVSSKVTVDVLFGLFNLPRE
NERMVRDKAVLMVQSDPRTRQKGPEHLAAFQWMSEYAKELVELRKREPGDDLITALIQAEVAGEKLADREVQM
TITTLIMAGIESLSGFLAMFALNLADHADARRRLAANPALIPDAIEESLRFNTSAQRFRRCLQKDVELHGQTM
RAGDFVCLAYGSGNRDERRFANAHLYDIDRKPKGHLGFGGGVHACLGTAFARLAARVACEEFLKRVPEFVRVQ
DQLPWMPSTTFRSPTRLELAVG

>CYP203B1(P73_4763)*Celeribacter indicus*

MPLFDIYSPEFDSNPYPVYATLRNEHPCYWHSGVKKWFLTRHADVSAAAANWKVFSSSQGNLVDEFASRAGNT
LGTMDPPRHDRLRALVQMAFTRKHLSALEPFVREQVREIIGRQAEARSFEFVSQVSTQITVSALSRLLGIDHD
DPNELREKALLMVQTDPVLRTKAPIHLEAMDWMKALAVRHIAARRAQPQEDVITSLIQAEIDGEGLTEDELIL
TISTVIMAGVESLSGFLTMVALNLADHSQVRRSVAADPSLIPAFIDEVLRYNTNAQRFKRVAMQDIELHGQTI
KKGDEVVLCFGAANRDERKFENPDSFDLDRPERGHLGFGGGVHMCLGVVLARMVTKLFLEELLKIMPEFSRND
DTLDWLPSTNFRAPLKLVLTREGTAWQGIEKVGSDA

>CYP204A1(Saro_3659)*Novosphingobium aromaticivorans*

MARAATAAGNGLPLLDGGVPLLGHLAQFFRDPVSVLKRGYRSKGRLFAMNFMGQRMNVMLGPEHNRFFFEETD
KLLSIRESMPFFLKMFSPEFYSFAEMDEYLRQRSIIMPRFKAASMKQYVPVMVEESLNLVERLGEEGEFDLIP
TLGPVVMDIAAHSFMGREFHEKLGHEFFELFRDFSGGMEFVLPLWLPTPKMVKSQRAKRKLHAILQSWIDKRR
AAPLDPPDFFQTMIETKYPDGRPVPDEIIRHLILLLVWAGHETTAGQVSWALADLLQNPDYQKVLRGEISSLL
GGSDGRDLGWEQAVAMEKMDLALRETERLHPVAYMLSRKARADIERDGYVIRKGEFVLLAPSVSHRMEETFRN
PDAYDPERFNPANPDAQIESNSLIGFGGGVHRCAGVNFARMEMKVLVAILLQNFDMELMDEVRPIAGASTYWP
AQPCRVRYRRRKLDGSEAGADMAALARAAGCPAHT

>CYP204A2(PP1Y_Mpl3478)*Novosphingobium* sp. PP1Y

MASTAHTSRPLPLVGGASPVIGHLAQFFRDPVSVLKRGYATKGRLFALNLMGRRMNVMLGPEHNRFFFEETDK
LLSIRESMPFFLKMFSPEFYSFAEMDEYLRQRAIIMPRFKAASMKQYVPIMAEEALALVKRLGDEGTFDLIPT
LGPVVMDIAAHSFMGREFHKKLGHEFFDLFRDFSAGMEFVLPLWLPTRKQIRSQRAKKKLHTILQDWIDNRRA
NPVDPPDFFQTMIASTYPDGTPVPDETIRHLILLLVWAGHETTAGQVSWALADLLQNPDYLQTIRAEIAAVMG
SDDGSAFSWEQALAMGKMDFSLRETERLHPVAYILSRKASADIEREGYRIRKGEFVLLAPSVTHRMEETFHNP
DAYDPERFNPDNPAAQLESNSLIGFGGGVHRCAGVNFARMEMKVLIAILLQHYDMELIDEVRPISGASTYWPA
QPCRVRYRKRNRGDARPADTATLARAAGCPAHA

>CYP206A1(Atu1569)*Agrobacterium fabrum*

MTEIGFRTPSTDTTGAQPVSKLATARLALSLIRNPLKALPPEIFSEPAVFTRLGGVMRVHLADPVLIHEALVK
NAALLGKGEDVRRALGPALGQGLLTADGDHWKWQRQSVAAAFRHEKLLELLPVMIETARRTQKRWRSSSTADI
DIGHEMMRTTFDIIVETMMSGGYGIDIARVEQSITDYLKPTGWTFALAMLGAPEWLPHPGRRKSRAAVDYLRA
SLATVITGRRKNPTDRPDLVSMLLEAKDPETGRMMSDEEIIDNLLTFITAGHETTALGLAWTFHLLSQNPETE
RKAVEEIEAVTGGEPVAAEHIANLAYVRQVFSEAMRLYPPAPVITRTALQDFRLGEHDIPAGTVLYVPIYAVH
RHTALWDEPERFDPSRFEPEKVKARHRYAYMPFGAGPRVCIGNAFAMMEAVAILAVILQKNHLENRTMASAEP
LMRVTLRPQERLMMKITQRQNKSPAV

>CYP206A2(B0909_05300)*Agrobacterium rhizogenes*

MIGTGLSTPSMDVTETEPLSKFAAARLAISLVRNPLKALPPEIFSKPAVFTRLGGTMRVHLADPALIHEALVK
NAHLLGKGEDVRRVLGPALGQGLLTADGDHWKWQRQSVAAAFRHEKLLDLLPVMIDTARRTQARWRAPSTGNI
DIGHEMMRTTFDIIVETMMSGGYDIDIARVEQSITDYLRPTGWTFALAMLGAPEWLPHPGRRKSRAAVDYLRA
SLAKVIAGRRANPTDRTDLVSMLLEARDPETGRMMSDEEIIDNLLTFITAGHETTALGLAWSFHLLSQNRETE

RKIIEEIEAVTAGEPVAAEHIANLTYTRQVFSEAMRLYPPAPVITRTARQDFRLGEHDIPAGTILYVPIYAVH
RHAALWDEPERFDPSRFEPEKVKARHRYAYMPFGAGPRVCIGNAFAMMEAVAILAVLLQTIHLENTSPAAAEP
LMRVTLRPQDRLMMRITQRKNRSPAA

>CYP206A3(AWN88_12890)*Agrobacterium tumefaciens* S33

MTDTGLRTPSPEAIGTQTVSKLATARLALSIIRNPLKALPPEIFHEPAVFTRLGDTMRVHLADPVLIHEALVK
NAHLLGKGEDVRRVLGPALGQGLLTADGDHWKWQRQSVAGAFRHEKLLDLLPVMIETARRTEARWRSPSIGDI
DVGHEMMRTTFDIIVETMMSGGYGIDVARVEQSITDYLKPTGWTFALTMLGAPEWLPHPGRRKARIAVGYLRE
SLAKVIAGRRKNPSERNDLISMLLEARDPETGRMMTDTEIVDNLLTFITAGHETTALGLAWTFHLLSKNRETE
RKILEEIDTVTAGEPVAAEHIAGLTYTRQVFSEAMRLYPPAPVITRTALEDFRLGGHDIPAGTVLYVPIYAVH
RHSALWDEPERFDPSRFEPEKAKARHRYAYMPFGAGPRICIGNAFAMMEAVAILAVLLQGLHLENRSTATAAP
LMRVTLRPESRLTMRIVQRKNKSPAA

>CYP206A3(BN877_I1537)*Rhizobium* sp. IRBG74

MTDTGLRTPSPEAIGTQTVSKLATARLALSIIRNPLKALPPEIFHEPAVFTRLGDTMRVHLADPVLIHEALVK
NAHLLGKGEDVRRVLGPALGQGLLTADGDHWKWQRQSVAGAFRHEKLLDLLPVMIETARRTEARWRSPSIGDI
DVGHEMMRTTFDIIVETMMSGGYGIDVARVEQSITDYLKPTGWTFALTMLGAPEWLPHPGRRKARIAVGYLRE
SLAKVIAGRRKNPSERNDLVSMLLEARDPETGRMMTDTEIVDNLLTFITAGHETTALGLAWTFHLLSKNRETE
RKILEEIDTVTAGEPVAAEHIAGLTYTRQVFSEAMRLYPPAPVITRTALEDFRLGGHDIPAGTVLYVPIYAVH
RHSALWDEPERFDPSRFEPGKAKARHRYAYMPFGAGPRICIGNAFAMMEAVAILAVLLQGLHLENRSTATAAP
LMRVTLRPESRLTMRIVQRKNKSPAA

>CYP206A4(Ach5_15090)*Agrobacterium tumefaciens* Ach5

MPGTDLRTPLPDTSAAAPVSKFATARLALSLIRNPLNALPPEIFNEPAVFIRVGGTMRVHLADPALIHEALVK
NAHLLGKGEDVRRALGPALGQGLLTADGDHWKWQRQSVAAAFRHEKLLELLPVMIEAAERTGRRWRASPFGDI
DIGHEMMRTTFDIIVETMMSGGHGIDIGRVEQSITDYLKPTGWTFALAMLGAPEWLPHPGRRKARAAVGYLRS
SLSTVIADRRRNPVERHDLVSMLLQAKDPETGRMMSDKEIIDNLLTFITAGHETTALGLAWTFNLLSQHSDVE
RKVVEEIAAVTGANPVAAEHIAQLTYTRQVFSEAMRLYPPAPVVTRTALQDFKLGDHDIPAGTVLYVPIYAVH
RHTALWDEPERFDPSRFEPEKTKARHRYAYMPFGAGPRVCIGNAFAMMEAVSILAVLLQNVHLENRSASPAEP
LMRVTLRPQSRLMMLITDRKNKSPAA

>CYP206A4(AGROH133_06357)*Agrobacterium* sp. H13-3

MPGTDLRTPLPDTSAAAPVSKFATARLALSLIRNPLNALPPEIFNEPAVFIRVGGTMRVHLADPVLIHEALVK
NAHLLGKGEDVRRALGPALGQGLLTADGDHWKWQRQSVAAAFRHEKLLELLPVMIEAAERTGRRWRASPFGDI
DIGHEMMRTTFDIIVETMMSGGHGIDIGRVEQSITDYLKPTGWTFALAMLGAPEWLPHPGRRKARAAVGYLRS
SLSTVIADRRQNPVERNDLVSMLLEAKDPETGRMMSDKEIVDNLLTFITAGHETTALGLAWTFNLLSQHSDVE
RKVVEEIAAVTGGNPVAAEHIAQLTYTRQVFSEAMRLYPPAPVVTRTALQDLKLGDHDIPAGTVLYVPIYAVH
RHTALWDEPERFDPSRFEPEKTKARHRYAYMPFGAGPRVCIGNAFAMMEAVSILAGLLQNVHLENRSASPAEP
LMRVTLRPQSRLMMRITDRKNKSPAA

>CYP206A5(Avi_2585)*Agrobacterium vitis*

MNMPVLSATSEGVVASRRSPVRVIADLIRNPLDALPPQIYDHKLVYAQFGDEVRLHVMDPELIHEALVKNAAV
LGKGEDVRRALGPALGQGLLTADGAHWKWQRQSVAAGFQWEKLRGFLPAMVAAAERRRDSWQAGETVDIGHEM
MQTTFDIIVETMMTGRDRIDVARVEQSVTDYLEPTGWMFALGMLKAPEWIPYPGKGKASRAVDYLRSAMATMI
AERRAEGVERADLISMLLSAHDPETDREMNDEQIIDNLLTFISAGHETTALGLAWTFHLLAKHPAIEQKLLDE
LDRVVGDGPVMAEHVEKLTYTKQVFNEAMRLYPPAPVITRTANEAFTLGQHHIQAGTVLLVPIHAVHRHSLIW
DRPEVFDPDRFAPEAVKARHRYAFMPFGAGPRICIGSAFATMEAVAILAVLAKTFRLRNKSEVTPEPTMRITL
RPKKKLIMEIENRREDVPVSG

>CYP206A6(BHK69_15760)*Bosea vaviloviae*

MMAAASAEIAPEIPFRSRRSSLSVLTSLLRNPLEAIPPEAFSEKLVLSRVAEQDSLYICDPALIQEALLRNAG
ALGKGDTLRRVLAPALGQGLLTADGAHWRWQRRAVAAGFQHDSLTGFLPAMIAAAEATRDGWLGKAGPLDIGH

EMMHTTFAIIVETMLSGSGDFDTVKVESAITDYLEPTNYMFAYSLFRAPEWLPYPGRRRAFASVAYLRETLGA
MVAKRRASGEHRNDLIDMLLASADPESGRAMSDAEIVDNLLTFVTAGHETTALGLAWTFHLLTQNRAVEDAVL
AEIEAVTGGGALQPEHVMALPLTKAVFQEAMRLYPPAPVVTRQVEEPFQLGGIALKQGMVLYVPIQAVHRHEQ
LWDQPDLFDPARFAPDAVRARHRYAYLPFGAGPRTCIGSAFATMEAVAILAVLLRAIRLEPLTQEPPEPTLKV
TLRPKQPLLMTAAPRA

>CYP206A7(BSY19_1991)*Bosea* sp. RAC05

MTTLEALAATTPDGLVESRRPALSVIASLLRNPLQAMPPEVYRERLVLATTGGRTSLYVCDPALIQEVLTRQA
GRLGKGNLIRKVLGPAIGEGLLTADGAHWRWQRQAVASGFQHERLLGFLPAMIEAAEATRGRWQAASAPLDIG
HEMMRTTFDIIIETMLSGAQALDVGKVEQAISDYLEPSNFMVIYNLFGLPGWLPYPGRARARASVTYLRENLR
ALVAQRRAEPPEHGDLLDMLLASADPETDRAMRDEEIVDNLLTFVTAGHETTAVGLAWTFHLLAQNRDWEARV
LEEIAAVTEGGPLLPRHVAALPLTRAVFQEAMRLYPPAPVITREVEEPFTLGGVALKQGMVLYVPIQAVHRHE
RLWDRPEQFDPSRFLGDAGRGRHRYAYLPFGAGPRICIGSAFATMEAVAILAVLLPALRLDPLPGQVPEPMLK
ITLRPKQPLRMHASPRG

>CYP206B1(Bind_1807)*Beijerinckia indica*

MNSATAPIMTKDSPGIAVNHESTLHFLRKIISNPLQTIPPEAFHEPLVYSDIGGKPRIYLSDPAIIQDVFINK
ADFIVKGTMVQRILGPALGNGLLTTDEGSNWRRQRQSIGPEFQHARLLDFQAEMIKAAERTRDRWSALGSQAQ
IDLRHEMMLTTFDVIGETMLSGRQEMDVFAIEQDIATYLKAAGWLMALEIMHAPAWTPFPGRRRSMAAARSMR
QAVVAMVARRRKENSRRDDLVSRLLATQDLESGKSMSDEEITDNLLTFIAAGHETTAQGLSWTFYLLSQHPEI
ETKVIKEIENVTKGQALRPDHIAQLVYTRQVFSEAIRLYPPVPLFTRKVVKNFTLGDFTIPADAILITPIFAV
HRHTSLWDQPDQFIPERFDPEQVKARHRFSFLPFGAGPRTCIGNAFAMMEAVAILAVLLPVFHLVYRSRQAPV
PTLQVTLQPKHKLYIQVHGR

>CYP206C1(Maq22A_c17130)*Methylobacterium aquaticum*

MNAHPSHVRPAPEGARPVLARITPPAQPLGPWRFLSTVVRNPLEAWPETIYREPVYTSTVLGNSSLFVMAPNL
IRRVMVDDADAFEKSEVLRRALSPALGDAILTADGARWRWQRRAAAPIFRAERIRSFVPAMIAAAERTRDALA
ADPGTEVDLAQTMMRTTFEIIVETMLPGAGGIDAARVERGITDYLESTSWVVALTLLRAPAWLPFPGRAKAER
AKTYLRDELLRLAAEGRRTGTEGRNDLLSLLLAARDPETGQAMDDRDVADNLLTFVTAGHETTALALTWALYL
LSHHPESEALIAAEVEAATAGGPLAPEHVDALPYTRQVILEAMRLYPPVPVVVRAALRDVDLDGIPVRAGTPI
TIPIYAVHRHAALWDEPERFDPARFAPEAAKARDRYAYLPFAAGPRICIGMGFALSEAVAILAVLVRSLRFSL
RPGYLPVLKQRITLRPSEGMPMRVSRR

>CYP206C2(M446_6661)*Methylobacterium* sp. 4-46

MTAQPTLPLDWPTTARLDPPEAPLAPLRYIRTVVRNPVETWPRAVYRDRVYQSTFLGRATLFVMDPGLVRTVL
VDRAESFEKSEVLRRSLSPALGDAILTADGARWRWQRRAAAPIFRNERILGFLPAMIAAAERTREALKGLPPG
TEADLAQVMMRTTFDIIAATMLSSHGRIDVARVERGITDYLESTSWIFALTLLRAPAWMPFPGRRRSEAARTY
LRDELLRLVAEGRAGGVEGRDDLLSLLVAARDPESGRAMDDRDVADNLLTFVTAGHETTALALAWTLYLLALH
PAIEARVVAEVEAVTGGGPLAPHHVEALGFTRQAIQEAMRLYPPAPVLVRAALEEVDVGGHRIGPGTPVTVPI
YAIHRHALLWDEPDRFDPDRFAPEAAKARDRYAYLPFGAGPRICIGMSFALMEAVAILAVLIRDLRFALRPGF
VPTLKQRITLRPAEGMPMRVAPRAG

>CYP206C3(Mnod_6754)*Methylobacterium nodulans*

MNAHPTLPLERPPTARLVPPEAPLSPLRFFRTVLRNPFETWPRAVYRDPVYRSSFLGRPTLYVMDPALVRAVL
VDQAESFEKSEVLRRSLSPALGDAILTADGARWRWQRRAAAPIFRNERIVSFLPAMIAAAERTRDALAAGPPE
AETDLAQMMMRTTFDIIVATMLSGDGQIDVARVEQGITDYLESTSWIFAMTLLRAPAWMPYPGRTRSERARGY
LRDELLRLVADMRRTGVEGRNDLMSLLVAARDPETGRAMDDRDVADNLLTFVTAGHETTALALAWTLYLLALH
PGIEARVVAEIEAVTGGGSVEPGHVEALAFTRQTILEAMRLYPPAPVIVRAALADVEIGGHRVPRGTPVTVPI
YAIHRHARLWDDPDRFDPDRFAPEAAKARDRYAYLPFGAGPRICIGMSFAMLEAVAVLAVLIRSLHFRLAPGF
VPTLKQRITLRPAEGMPMRVARRDG

>CYP206D1(MOC_4866)*Methylobacterium oryzae*

MAEAIALPSVLVTMLGSLIEAWPRQIYTQPLVTTRFLGQRTTYVCDPGHIRSLLVDQAEALEREPFMLRALAP
ALGSGVLTADGSHWRGQRRTASPMFRPDRVRSFVPAMARAAAATRTRWHGADPAGAERDVLHEMMRTTFEVIV
ATMVSGDPHLEVEPFGRAMDAYLGQTSWKIALAMLRAPAWVPHPGARGGAAAVRHLRGEVARTIARRRARGEP
GTDLLGLLLEARDPETGQPLPEARVVDNLLTFVAAGHETTALALAWTLRVLAEHPEVEARVVAEMAQLGDDPA
ADPEAADRLPYTRQVLLEVMRLYPPAPLIVRRTRAPVRLGDRVVPAGQSVHVPVYALHRHALLWDRPEVFDPD
RFAPERAAARDRYAYLPFGAGPRVCIGMSLALTECLVILATLLPAFRFRPVTRAMPATQFRVTLRPKGGLTMR
VEPRHVA

>CYP206D2(Mrad2831_4212)*Methylobacterium radiotolerans*

MPAVGTFRVCAPGRPPFAAAVAEGVAEAVALPSVLVTMLGSLIEAWPRQIYTQPLVTTRFLGQRTTYVCDPGH
IRSLMVDQAEALEREPFMLRALAPALGAGVLTADGARWRRQRRTAIPMFRPDRVRSFVPAMARAAAATRARWR
DADPAGAERDILREMMRTAFDVIVATMVSGDPHLEVEPFGAAMDAYLGQTSWKIALEMLRAPSWVPHPGARAG
AEAVRYLRGEVARTIARRRARGEPGSDLLGLLLQARDPETGAPLPEASLVDNLLTFVSAGHETTALALAWTFR
VLAEHPEVERRVVAEMARLGDDLAADPEAADRLPYTRQVLLEVMRLYPPAPLIVRRTRAPVRLGDTVVPAGQS
VHVPVYALHRHALLWDRPEAFDPDRFAPERAAARDRYAYLPFGAGPRVCIGMSLALTECLVILATLLPAFRLR
PVTAEMPAAQFRVTLRPKGGLTMRVAPRPGAGHVA

>CYP206D3(METDI1976)*Methylobacterium extorquens* DM4

MVAEGRDGEVADMSSAGTFRVCEAGGPPLQEALAEAVALPSVLATMLRSLIEAWPDEVYTRPLVTTRFLGQRT
TYVCAPGHIRSLIVDQAGALEREPFMLRALAPALGSGILTADGPHWRGQRRTAAPMFRPDRVRAFIPAMAEAA
HATRLRWLGQMSAHGSADARFDGERDILPEMMRTTFDVIVATMVSGDSQLKVEPFGRAIDAYLGQTPWKIALS
MLGAPAWMPHPGARAGARAVRYLRSEVARTVERRRARGEPGADLLGLLLQAKDPETGERLSDESLIDNLLTFV
AAGHETTALALTWTLRVLADHPAVEARILDEIAGLGADPAPEALDRLAFTRQVVLEVMRLYPPAPLIVRRTAE
EVRLGDTVIPAGESVHVPVYALHRHQSLWDRPDVFDPDRFAPELTASRDRYAYLPFGAGPRVCIGMGLALTEC
LVILATLLPAFRFVPAKAEMPATQFRVTLRPKGGMKMKVVPRHAH

>CYP206D3(Mchl_1531)*Methylobacterium extorquens* CM4

MSSAGTFRVCEAGGPPLQEALAEAVALPSVLATMLRSLIEAWPDEVYTRPLVTTRFLGQRTTYVCAPGHIRSL
IVDQAGALEREPFMLRALAPALGSGILTSDGPHWRGQRRTAAPMFRPDRVRAFIPAMAEAAHATRLRWLGQMS
AHGSADARFDGERDILPEMMRTTFDVIVATMVSGDSQLKVEPFGRAIDAYLGQTPWKIALSMLGAPAWMPHPG
ARAGARAVRYLRSEVARTVERRRARGEPGADLLGLLLQAKDPETGERLSDESLIDNLLTFVAAGHETTALALT
WTLRVLADHPAVEARILDEIAGLGADPAPEALDRLAFTRQVVLEVMRLYPPAPLIVRRTAEEVRLGDTVIPAG
ESVHVPVYALHRHQSLWDRPDVFDPDRFAPELTASRDRYAYLPFGAGPRVCIGMGLALTECLVILATLLPAFR
FVPAKAEMPATQFRVTLRPKGGMKMKVVPRHAH

>CYP206D3(Mext_1330)*Methylobacterium extorquens* PA1

MSSAGTFRVCEAGGPPLQEALAEAVALPSVLATMLRSLIEAWPDEVYTRPLVTTRFLGQRTTYVCAPGHIRSL
IVDQAGALEREPFMLRALAPALGSGILTADGPHWRGQRRTAAPMFRPDRVKAFIPAMAEAAHATRLRWLGQMS
AHRSADARFDGERDILPEMMRTTFDVIVATMVSGDSQLKVEPFGRAIDAYLGQTPWKIALSMLGAPAWMPHPG
ARAGARAVRYLRSEVARTVERRRARGEPGADLLGLLLQAKDPETGERLSDESLIDNLLTFVAAGHETTALALI
WTLRVLADHPAVEARILDEIAGLGADPAPEALDRLAFTRQVVLEVMRLYPPAPLIVRRTAEEVRLGDMVIPAG
ESVHVPVYALHRHQSLWERPDVFDPDRFAPELTASRDRYAYLPFGAGPRVCIGMGLALTECLVILATLLPAFR
FVPAKAEMPATQFRVTLRPKGGMKMKVVPRHAH

>CYP206D3(Mex_1p1130)*Methylobacterium extorquens* AM1

MLRSLIEAWPDEVYTRPLVTTRFLGQRTTYVCAPGHIRSLIVDQAGALEREPFMLRALAPALGSGILTADGPH
WRGQRRTAAPMFRPDRVRAFIPAMAEAAHATRLRWLGQMSAHGSADARFDGERDILPEMMRTTFDVIVATMVS
GDSQLKVEPFGRAIDAYLGQTPWKIALSMLRAPAWMPHPGARAGARAVRYLRSEVARTVERRRSRGEPGADLL
GLLLQAKDPETGERLSDESLIDNLLTFVAAGHETTALALTWTLRVLADHPAVEARILDEIAGLGADPAPEALD
RLAFTRQVVLEVMRLYPPAPLIVRRTAEEVRLGDTVIPAGESVHVPVYALHRHQSLWDRPDVFDPDRFAPELT
ASRDRYAYLPFGAGPRVCIGMGLALTECLVILATLLPAFRFVPAKAEMPATQFRVTLRPKGGMKMKVVPRHAH

>CYP219A1(Saro_2451)*Novosphingobium aromaticivorans*

MEAEAAIPPLDTSDPALIPDPWPTFTTLRERDPFHWSKYGYWVVSRHEHVRDVLMNRKDFGTGDFAANLRLFY
GPDFDVLANPAYRWLSEVFIMQDPPQHTRIRNLVVGSLTAKRVRAMEPRIREIAQALTDGFKARGSADLITEF
AYKFPVMVICDLMGIDYEASEMADLIAAIPEAFTVFEARILSPEELALANRRILELEAFFQAQFENRLAHPRG
DLLTSLARTGQEPGGLSVHEAITVTIGLFGAGFETTANIIGNGLHALHANPEQWARLVADPSGMASGACEEAL
RHQSSLIATYRTALADTSVCGHPVSAGQRVLTLIGAANRDPRKFADPDRFDIARNDADHLTFGGGIHFCVGAE
LARIEARVAFEHLARELPQMQVDTGGACWRENFLFRGLTGLEARWPAQA

>CYP219A2(AMC99_02081)*Altererythrobacter epoxidivorans*

MAREDVTSFAPDAPGMSLDPYPAFAELREKDPLHRSDLGYWVVSRHEDVRGVLMGRDSFGQGDFVENIRLFYG
PDFDVLGQRSYKWLSEVFLMQDPPHHTRVRGMVTGALTAKRVRAMEGRICEIADELIDAMLAAGSADLITDFA
YQLPVRVMCDMLGIDPEDPRLPAVIGAIAQSFIVFEARALTDTELATANEQISILQAFFEDLFEQRMAEPRDD
LATALVQSGGNEDSLSHDELVTVAIGLFGAGFETTAHMIGNAVLSFARFPDEWAKLVADPSGMAAGAVDETLR
FESSLIATYRTALAETQLRGETILPGEKVLTLIGAGNRDPRVFDDPDRFDIARDARGHLSFGGGIHFCAGAEL
ARLEGRVAFEQLARRIPTLKADASNPQWRDGFLFRGLSSLPVSW

>CYP223A1(Saro_3832)*Novosphingobium aromaticivorans*

MHRAMTTTVQDFDPEVPEDFDSPHAEYARLRRECPVAHTNGLGGFWALTRYEDVKRAASDSTTFITSVQNVVP
KVAFTGRRPPLHLDPPEHTPYRKALNPLLSLERSEAFAGKARELTRKLLAPMVENGGGDICVELSSYLPVHVF
GEWMRMPEEWLDTLHDAGRAFILAVHSNTPERMKETSLRLYDMARGLIAVRRENPQDPALDPTSALLAARHEG
EPLPEELLVGTVRQVLVVGMVAPMVMIGNICVHLSRDKALQQQLRADPSLVPAAIEEFLRLYTPYRGFARTAV
CDVDMGGRTIPKDEAIALVYASANRDEDVFPDGDKFILNRPNIAQHLAFGRGPHNCPGVHLGRMQLRVALEEI
LAATREFELSGPVSVSRWPEVGALSVPLRFV

>CYP223A2(PP1Y_Mpl590)*Novosphingobium* sp. PP1Y

MKEIMQDFDPERPEDFDSAHAEYARLRRECPVAHTDGLGGFWALTRYDDVKRAASDPLFVTSVQNVVPKVAFT
GRRPPLHLNPPEHTPYRKALNPLLSQERAEALAPHTRTLARELLAPMVAKGGGDICVEFSSHLPVHVFGEWMR
MPEESLATLHDAGRAFILAVHSTKPESMKETSLRLYEMARDLIALRHREPQDPAIDPTSALLAARHEGEPLPD
ELVLGTVRQVLVVGMVAPMVMIGSIAVHLSRDLALQQRLRSDPSLIPSAIEEFLRLYTPYRGFARTPVCPVEM
GGRMIDKDEAVALVYASANRDEAVFPNPGEFIMDRPNIAEHLAFGRGPHNCPGLHIGRMQLRVALEELLVATK
QFDLDGEIMMSRWPEIGALSVPLRFD

>CYP223E1(WYH_00908)*Altererythrobacter atlanticus*

MTEDNPNLPDRPEDFDAEAPETFESPHQLYRHLRAECPVAHSNAYGGFWALTRYQDIVDAVTDNETFITSKIN
VIPNMSMGKRRPPLGKDPPEHTPFRRALDKTLRNARIQALEPVLRAHAVREMSRIVADGVTDISLDFATIFPA
WVAVEWLNLDETHAPLLAEVARLYNVAWRAEDTEAVVRTSEKLYDIAASVVKDRKANPRSPETDPASALLAER
TEFGPIPEEYVIATVRQVLVVGLMAPPPLFGSMCVHLASDPELQQLLRDEPQRIPAAIEELLRLYSPYRGFAR
TTTRDYERHGRTIPAGEPVTMVYSSANRDERVFDDPDSFRFDRENIRSHLAFGRGVHQCAGSGLVRLELRIFL
EELLSRTSEIHLNGEIEMARMPELGPAATPLRLVPAR

>CYP224A1(Saro_3682)*Novosphingobium aromaticivorans*

MTLLFQPSPPDHVPGERMVDFDMFHVPEGQDDPVEIWHDLVRRGVPRIFYTPRNGGHWVFLDYADIVEAYRDH
TVFSTYQTPVPPIEPFPVVQPQGVDPPAHNVFRRLLAPMFTPTAVRGMIGELERRASELIDRFAARGECDFIT
EFAERFPTSTFLHLFGLPEEQLDAFLALANVFFRSTDAETRARNIGEIYAVLDTLFREKERNPGNDIASAIVA
ARDEEGRQHPWEDILNCGFLLFVAGLDTVTNTMAYIWRYLATTPAARRHFRERLDDPDAFLRAIEELMRINAV
SNLFRRVTHDCEYKGVQLRRNDRVVLPNTVANRDPRVFSDPQAIDLDREVNVHLTFGVGPHRCIGSVLAKREV
MVSLQQWLRRIPEFELAPEQPAGSAFGGSVMGFTALRLRWVRVEA

>CYP225A1(Saro_2608)*Novosphingobium aromaticivorans*

MQFPFSRSTNPNVDLSSLDAFNEGAPFATFDRMRREDPMAWSEMVNGDRGFWSVTRHADLLELNRQADLLSSA
KGIRMEDQTEEEYEARKTFQETDAPHHRGFRALVSKAFSKGTVAGFEDQIRKIVTDLLDVALAEGEFDAVDRI
ARRLPMQMLAQIMGVPQEDGPWLVEKGDALISNSDPDYTDFVVDQVDTEAYRMLPFRSPAAVELFDYANGLLD

RMDAGEQIGVLNLVREPTSTGTRMSRDEFRNFFCLLVAAGNDTTRYSISATIHALANNPHLLQALKDGDFTSW
EAAADEMIRYASPTTHFRRTATRDFTFHDRHVKAGDKVLLWFISGNRDETAILDPYTINLRRERNPFLSFGQG
GPHICLGMWLAKLEVAIVMQELAKRLSSIEQVAEHSYLRSNFIHGIKHLPVRIVAR

>CYP225A4(METH_03565)*Leisingera methylohalidivorans*

MTVWTPADDGHADLSSHDAFVHGPPHNTFARLRREDPLHWTEYAGGENFWSVTRYEDITVMNKNTEVFSSARG
IRMEDQSYEEYLARRTFQETDPPEHSKVRMKLLKAFSKTTMAQYEQDIRDLCADILDQALAKGEFDATREIAR
QLPMRMLGRVVGLPEADLPWLVEKGDALIANTDPDFTSHVLDRMQTDEFRMMPFNSPAGAELYVYAKELMEAK
AKTGDTAGVLNLILAPAKDGSVITETEFRNFFCLLVAAGNDTTRYSIAAGIQAMCRQPELLAQMQAGGAVWDT
AADEIIRWATPALYFRRTATRDVEMHGKTIGAGDKVLYWFASANRDDGYFEDPFHVNLHRSPNRHLSFGQFGP
HVCLGMWLARLEVTVLFQELAKRIRHIESAGPHKFLRSNFVGGIKALPVRVEAA

>CYP225A5(TM1040_3721)*Ruegeria* sp. TM1040

MTIWTPTDDGYADLSSHDAFANGAPHNTFARLRRDDPLHWTEYSDGENFWSVTRYDDITKMNKNTEIFSSARG
IRMEDQTYEEYLARRTFQETDPPEHSQVRMKLLKAFSKTTMAQYEADIRDLCAEILDEALAKGSFDATKEIAR
QLPMRMLGRVVGLPDADLPWLVEKGDALIANTDPDFTSHVLDKMDTDEFRMMPFNSPAGAELYIYAKELMEAK
ERAGDTSGVLNMILQPARDGSVITETEFRNFFCLLVAAGNDTTRYSIAAGIQAMCHQPELLAQMQAGGEIWET
AADEIIRWATPALYFRRTATQDVEMHGKTIREGDKVLYWFASANRDDSYFDDPFRVNLMRNPNRHLSFGQFGP
HVCLGMWLARLEVTVLFQELSKRIKSIEPNGAHKFLRSNFVGGIKELPVRVEAA

>CYP225A6(SPO0764)*Ruegeria pomeroyi*

MTVWTPTDDGYADLTSHDAFAGGAPHNTFARLRREDPLHWTEYADGQDFWSVTRHADIAEMNRNTAVFSSARG
IRMEDQSYEEYLARRTFQETDPPEHSQTRMLLMKAFSRTTMAQYEDDIRAICVDILDEVLDKGTFDATKEIAR
QLPMRMLGRIVGLPDADLPWLVEKGDALIANTDPDFTTHVLDKLDTDEYRMMPFNSPAGAELYLYAKDLMEQK
DKAGDTSGVLHMILQPSKDGSVISDTEFRNFFCLVVAAGNDTTRYSIAAGIQAMCHQPELLEQMRAGGDIWET
APDEIIRWATPALYFRRTALQDHEMHGKTIRAGDKVLYWWSSANRDETVFDDPFRVDLFRNPNRHLSFGQGGP
HVCLGMWLARLEVKVLFQELAKRLSSIEPAGPHRFLRSNFVGGIKELPVTVRRA

>CYP225A7(LOKVESSMR4R_00419) *Yoonia vestfoldensis*

MTIWTPTDDGHADLTSHDTFTAGAPHNTFKRLRHEDPIHWTEWDQGKGFWSITRHADIMEMNRNTAVFSSEHG
IRMEDQSPEEVIARRTFQEIDPPEHMKTRIKLAKAFSKGVIEGFNSDIRDLCVTILDDVLHEREFDATKRIAR
VLPMLMLGRIIGTPEDDLPWLVEKGDELIANTDPDFTDHVLDKITTDEFRMMPFNSPAGAELFAYAKEMLERK
KREGDTSGVLHMILQPGPDGDVISETEFRNFFCLLVAAGNDTTRYSIAGGIQAMCHQPELLAQMQAGGDIWET
APDEIIRWATPALYFRRTALSDYEIHGKTIKAGDKVLYWFSSANRDEAVFDDPFRVDLFRTPNKHLSFGQGGP
HLCLGMWLARLEVRVLFQELSKRITHIEAAGPHQFLRSNFVGGIKSLPVRVTLA

>CYP225A8(Jann_2917)*Jannaschia* sp. CCS1

MTIWNAVDDGHADLSNHDTFVAGPPLATFDRLRRDDPLSWTDWDGGTGFWSVTRYHDILEMNRNTKVFSSARG
IRMEDQSYEEYLARRTFQETDAPDHMQMRIKLARAFSKNVIAEFEEDIRGLCRDILDEVLEDESFDATKRIAR
ELPMRMLGRILGTPDDDLPWLVEKGDALIANTDPDFTSHVLDKMSTDEFRMMPFNSPAGAELYAYAKDLMATK
TASGDTNGVLHLILQPGPDGSVISETEFRNFFCLLVAAGNDTTRYSIAAGMQAMCYQPELLDQMKQGDIWETA
ADEIIRWACPTSYFRRTATQDYDMHGKTIREGDKVLYWFASGNRDTAYFDSPNRLDLARSPNKHLSFGHGGPH
LCLGMWLARLEVTVLFQELSRRITKIEADGPQQFLRSNFISGLKSLPVRITRA

>CYP225A9(RCA23_c26300)*Planktomarina temperate*

MHDDGYLNLLSHDSFAGGVPHKTFARLRKDDPLHWNEGDADTKGFWNLTRHADIAFANKENQIFSSAQGIRLE
DQSAEEYLARRTFQETDPPEHTKVRRLVNPNFAKPIVGEFEPVIRELSSNIVDKALRNTEFDAVEVIAKQLPM
LMLGRILGVPDEDLDWLVEKGDALIGNSDPDFTEHVIDQVDTSAYRMMPFRSPAGLELYDYASDLLSNRRPLA
QEGILAKITGDENGLNELEFKNFFCLLVAAGNDTTRYSIAQALSLLAHNPDLIAQLKTGKYWDSCADEFIRLA
SPTMHFRRTATQDFEMHGKKIRAGDKVLLWFVSGSRDEALFEDPNECILGRSPNRHLAFGQGGIHVCLGMHLA
KLEVRIAIEELVKRVASFEATADPTWTRSNFICGVKQQHVRVTKVA

>CYP226C1(Caul_1912)*Caulobacter* sp. K31

MSTAQMTPNKDLAEGFAQVGALFAGNDKNIDAIYRDHRQNMPVMRGDICAELGAASFAGQTGRPIYTIFRHAD
VMKVLRDTKTFTSGILMETGLGQFLDGLMITGLDGDEHRQLRGILQPSFTPAVMEEWRETYIRPLIQRSFVEP
LVALGKTELIGSVGVMFPIHVVYAVLGFQDNDPAALETFATKALKVLGGMADDPDAKRAAFQAFQELYDPTLA
AVQARRASGAEGADLISRLIRAEFEGRTLNDHQITNFVRMMLPAASETTSRTFATMLTHLFDHPEVLERLRAD
RSLMRKVLDESVRHDAVATFKVRECQADVTLQDVTIPKGSIISACVASANRDELVFDKPEVFDIDRKQMPAFG
FGFGAHMCVGMWLAKVEIEEAVGLLLDMLPNLRLDPDHPRPEVRGVSLRGPDAVHVIWDIP

>CYP226D1(BSY18_2515)*Blastomonas* sp. RAC04

MTVKLDPLDQMVADYSLVTNGYAGKAPNNPYPMLAEKRSTCPVMHGDILQQHQVPSMADYMMTGRPIVSLFRY
KDIHAVLMNPADWQSYIVGDGFGAAVDNMLLTAMDGSEHGAFRSTLQKPFMRPQIRKLIDTLIRPVVMNDFVN
RLRPNGKADLLREFALPFPIRAIYAYFGFPDDKDLLANLASWAIQVVAAPQSDPELAKITVPQSMVAGQSMYD
TLLPVVQAYRARGEMREDILGYMMQTQHEGQMFTDEEITAYIRMLLLAAGETTTRSFANMMVQLLENPDVLEE
VRQDRTLIAKAVTETMRRDPTAGALGRIAARDMEIDGITIPKGTAVLLAITAANRDPQVYEAPDRLWLKRPMR
PLLSFGFGPHMCMGMHMAQAEMEVALDAMLDLPNLRFDPAFPHPEIRGLNLRGPDAIHVLWDA

>CYP278A4(Plav_3499)*Parvibaculum lavamentivorans*

MQEIRKSDRADDLAALVAALPAGDTIASPYALYDRLRPYGPVYGYRDYPPGTVPDADEAVTAWVLLNYDHVSA
AARDHRIFSSRDPLQEQSSAPTLMLVNHDNPEHDRLRSIVNLAFSRPRIEALDPWVREIVGGMVAGLGDGDTE
VMEALAARIPARIMVGLLGLPEEVVDRFRHWATAFMLSADLDPAEREASNAELVRYFTETVDSLYGALEAGDP
VPDGLIAALLKAEVDGEQLTLDEVIRFCITLVVAGSETTTFLLGNLLHNLAVMPDIRAQLAANRALIGPFIDE
SLRHSGPPQRLFRIATQDVEVGGARISKGDWVALFFAAANHDPAMFPDPEKFDISRPNLNKQLTFGVGIHHCL
GSALARMEGRALIEAILDRMDDVSLGALPPVPQRASLLNHGFDSLTLRFTAREDETQ

>CYP278C1(Hbal_2472)*Hirschia baltica*

MSNIAEVNIDPDFKVKDLYSPEVIANPYPYYDHLRDKPVQFGLEDFPPGTIPGQDKPFPAWVFLKHADVVEVC
RRADVFSSRDVMQEESDAPTLMLVNHDDPRHAELRKIAQIAFAPKRVMTDVAPWMEKTVAGQLEEYGEEELDF
MQDLAPNLPALVMTKLIGTPESDYKLLRRWANAFMVTSEFTLEERNQCNIDLWQYYSQKVEERYSDIESGKDV
PDDLMSAFIKAESDGNKLTKEEVVRFCLTLVVAGAETTGYLLGNLIDVLVDNPKLFKLLQDDRTQVRAFIDES
LRRDGPVQRLHRVCVKDVEVGGASIKAGDWVAIFHASANRDPAVFENPNEFIMGRPNIGKHATFGHGIHHCMG
SGIARNEAAQMINGILDRYRGVESNGERVRQSGGLLNYGLETCPIKFLK

>CYP286C2(G432_16275)*Sphingomonas* sp. MM-1

MATLLERPMAPETSDPSLARPASEIPLVSVVPFLGNTLEMAKDPAAFFLRCYREYGSVFKVKVFGRESIVIAG
PESALFMTTREGRDALRSKEYNQGVADEYGATEVINGVDGERHVRLRTLMRQGFSREAVHGQYDDLVRITDSA
IARRWQVGSRLPVVSAIQYMIVEQLGMILTGQAPLDYVEDIRFAILTILNVLVTRQRPKIMLHDPRFRKARRR
VEELGHTMIADFQRKVADGTAPKNLITDIIRANLDDKDIMPDQNLILTVTGPYVAGLDTAANTMAACIYGVLK
NPEVLARVQAEADELFAKETLNEDDIRKLPVINGAVMEAMRLWPIAVAQMRVANFDFEYAGYTIPKDEMIYIG
TSVPHFMEEFFPDVNKFDVDRYQRPRAEHLQKGAYSPFGRGPHSCLGQGLAEVLMAMSIARIFHRLDLALPHP
NYELKTKTAPTPGPAMDFAVKVIGERRPSGI

>CYP288B1(BBta_1606)*Bradyrhizobium* sp. BTAi1

MPGSAAQLARCPYHDAATDFDPFEMRDPFPFYEWARAEAPVFFSDELKYFVVARHADIKAVFDDWRTFSSENA
QAPLRPMCEEGRRIMREGGFTAYSGLSARVPPDHTRIRKLVQGCFGPRRFRAIEPEIKAIVNRAIDTFAERGH
ADFFREFAYDVPALVLFKLVGIPNIDVPRVKSWAVSRALLTWGDLSDDEQIVHARNMVEYWNYCRALVRQRHD
DPTDDLPGDLVRLQKDGAEISDEEIAGVLYSALFAGHETTTTLMANGMRELLQRRENWEALIAEPQLIPNAVE
ECLRFSPSIVAWRRRALKDTEIGGVPVPKEANILLLIGSANRDETVFSAPARFDVRRTDARSHLAFGYGIHTC
VGQQLARIEFAIALGELTRRLPGLRLAADQTDDFVHNISFRVPTALRIEWDVA

>CYP288B1(S58_10750)*Bradyrhizobium oligotrophicum*

MPGSAAQLARCPYSDAAKDFDPFEMRDPFPFYEWARAEAPVFFSDELKYFVVARHADIKAVFDDWRTFSSENA
QAPLRPMCEEGKRIMREGGFTAYSGLSARVPPDHTRIRKLVQGCFGPRRFRAIEPEIKAIVNQAIDGFADRGH
ADFFREFAYDVPALVLFKLVGVPNMDVPRVKSWAVSRALLTWGDLSDDEQIVHAHNMVEYWNYCRELVRQRHD
DPTDDLPGDLVRLQKDGAEISDEEIAGVLYSVLFAGHETTTTLMANGMRELLQRRENWEAIIAEPRLIPTAVE
ESLRFSPSIVAWRRRALKDARIGGMPVPKDSNILLLIGSANRDETVFSAPARFDVRRSDARSHLAFGYGIHTC
VGQQLARIEFAIALGELTRRLPGLRLAADQSIDFVHNISFRVPTALRIEWDMA

>CYP289A1(BMEII0879)*Brucella melitensis* bv. 1 16M

MRAGGIMTSPTERPKQDWDPRSEAVLSDQIGAYDAMRHQCPVAHSDYLGWSLFSYDDVVRVLDDHETFSSVVS
AHLSVPSGMDPPQHTAFRQLVERYFEPERIKAFEPICREISKKLVCELPRDAEIDLVTQFAQLYAVRIQCAFL
GWPDSLQGPLLDWVHKNHAATLARDTKAMAAIALEFDEYIRDLLDERRKLGADAPDDVTTRLLRDRIDRRNLT
HEEIVSILRNWTVGELGTITACVGILCHYLAKSQQTQALMRGGPDLLPAAIDEILRLHAPLISNRRVTTRAVV
VGGREIPAGEKITLMWASANRDEAVFDKPDELRLNRDPALNLLYGRGIHVCPGAELARAGLRILMEELLGQTR
KLDLVPGSVPALAVYPASGFSRLPARIS

>CYP289A1(DK63_2369)*Brucella melitensis*_bv. 1 16M

MTSPTERPKQDWDPRSEAVLSDQIGAYDAMRHQCPVAHSDYLGWSLFSYDDVVRVLDDHETFSSVVSAHLSVP
SGMDPPQHTAFRQLVERYFEPERIKAFEPICREISKKLVCELPRDAEIDLVTQFAQLYAVRIQCAFLGWPDSL
QGPLLDWVHKNHAATLARDTKAMAAIALEFDEYIRDLLDERRKLGADAPDDVTTRLLRDRIDRRNLTHEEIVS
ILRNWTVGELGTITACVGILCHYLAKSQQTQALMRGGPDLLPAAIDEILRLHAPLISNRRVTTRAVVVGGREI
PAGEKITLMWASANRDEAVFDKPDELRLNRDPALNLLYGRGIHVCPGAELARAGLRILMEELLGQTRKLDLVP
GSVPALAVYPASGFSRLPARIS

>CYP289A1(BM28_B0360)*Brucella melitensis* M28

MTSPTERPKQDWDPRSEAVLSDQIGAYDAMRHQCPVAHSDYLGWSLFSYDDVVRVLDDHETFSSVVSAHLSVP
SGMDPPQHTAFRQLVERYFEPERIKAFEPICREISKKLVCELPRDAEIDLVTQFAQLYAVRIQCAFLGWPDSL
QGPLLDWVHKNHAATLARDTKAMAAIALEFDEYIRDLLDERRKLGADAPDDVTTRLLRDRIDRRNLTHEEIVS
ILRNWTVGELGTITACVGILCHYLAKSQQTQALMRGGPDLLPAAIDEILRLHAPLISNRRVTTRAVVVGGREI
PAGEKITLMWASANRDEAVFDKPDELRLNRDPALNLLYGRGIHVCPGAELARAELRILMEELLGQTRKLDLVP
GSVPALAVYPASGFSRLPARIS

>CYP289A1(BM590_B0358)*Brucella melitensis* M5-90

MTSPTERPKQDWDPRSEAVLSDQIGAYDAMRHQCPVAHSDYLGWSLFSYDDVVRVLDDHETFSSVVSAHLSVP
SGMDPPQHTAFRQLVERYFEPERIKAFEPICREISKKLVCELPRDAEIDLVTQFAQLYAVRIQCAFLGWPDSL
QGPLLDWVHKNHAATLARDTKAMAAIALEFDEYIRDLLDERRKLGADAPDDVTTRLLRDRIDRRNLTHEEIVS
ILRNWTVGELGTITACVGILCHYLAKSQQTQALMRGGPDLLPAAIDEILRLHAPLISNRRVTTRAVVVGGREI
PAGEKITLMWASANRDEAVFDKPDELRLNRDPALNLLYGRGIHVCPGAELARAELRILMEELLGQTRKLDLVP
GSVPALAVYPASGFSRLPARIS

>CYP289A1(BMNI_II0352)*Brucella melitensis* NI

MTSPTERPKQDWDPRSEAVLSDQIGAYDAMRHQCPVAHSDYLGWSLFSYDDVVRVLDDHETFSSVVSAHLSVP
SGMDPPQHTAFRQLVERYFEPERIKAFEPICREISKKLVCELPRDAEIDLVTQFAQLYAVRIQCAFLGWPDSL
QGPLLDWVHKNHAATLARDTKAMAAIALEFDEYIRDLLDERRKLGADAPDDVTTRLLRDRIDRRNLTHEEIVS
ILRNWTVGELGTITACVGILCHYLAKSQQTQALMRGGPDLLPAAIDEILRLHAPLISNRRVTTRAVVVGGREI
PAGEKITLMWASANRDEAVFDKPDELRLNRDPALNLLYGRGIHVCPGAELARAELRILMEELLGQTRKLDLVP
GSVPALAVYPASGFSRLPARIS

>CYP289A1(BAB2_0833)*Brucella abortus* 2308

MTSPTERPKQDWDPRSEAVLSDQIGAYDAMRHQCPVAHSDYLGWSLFSYDDVVRVLDDHETFSSVVSAHLSVP
SGMDPPQHTAFRQLVERYFEPERIKAFEPICREISKKLVCELPRDAEIDLVTQFAQLYAVRIQCAFLGWPDSL
QGPLLDWVHKNHAATLARDTKAMAAIALEFDEYIRDLLDERRKLGADAPDDVTTRLLRDRIDRRNLTHEEIVS
ILRNWTVGELGTITACVGILCHYLAKSQQTQALMRGGPDLLPAAIDEILRLHAPLISNRRVTTRAVVVGGREI

PAGEKITLMWASANRDEAVFDKPDELRLNRDPALNLLYGRGIHVCPGAELARAELRILMEELLGQTRKLDLVP
GSVPALAVYPASGFSRLPARIS

>CYP289A1(BAbS19_II07720)*Brucella abortus* S19

MTSPTERPKQDWDPRSEAVLSDQIGAYDAMRHQCPVAHSDYLGWSLFSYDDVVRVLDDHETFSSVVSAHLSVP
SGMDPPQHTAFRQLVERYFEPERIKAFEPICREISKKLVCELPRDAEIDLVTQFAQLYAVRIQCAFLGWPDSL
QGPLLDWVHKNHAATLARDTKAMAAIALEFDEYIRDLLDERRKLGADAPDDVTTRLLRDRIDRRNLTHEEIVS
ILRNWTVGELGTITACVGILCHYLAKSQQTQALMRGGPDLLPAAIDEILRLHAPLISNRRVTTRAVVVGGREI
PAGEKITLMWASANRDEAVFDKPDELRLNRDPALNLLYGRGIHVCPGAELARAELRILMEELLGQTRKLDLVP
GSVPALAVYPASGFSRLPARIS

>CYP289A1(DK55_2790)*Brucella abortus* bv. 2 86/8/59

MTSPTERPKQDWDPRSEAVLSDQIGAYDAMRHQCPVAHSDYLGWSLFSYDDVVRVLDDHETFSSVVSAHLSVP
SGMDPPQHTAFRQLVERYFEPERIKAFEPICREISKKLVCELPRDAEIDLVTQFAQLYAVRIQCAFLGWPDSL
QGPLLDWVHKNHAATLARDTKAMAAIALEFDEYIRDLLDERRKLGADAPDDVTTRLLRDRIDRRNLTHEEIVS
ILRNWTVGELGTITACVGILCHYLAKSQQTQALMRGGPDLLPAAIDEILRLHAPLISNRRVTTRAVVVGGREI
PAGEKITLMWASANRDEAVFDKPDELRLNRDPALNLLYGRGIHVCPGAELARAELRILMEELLGQTRKLDLVP
GSVPALAVYPASGFSRLPARIS

>CYP289A1(DO74_2104)*Brucella abortus* bv. 6 870

MTSPTERPKQDWDPRSEAVLSDQIGAYDAMRHQCPVAHSDYLGWSLFSYDDVVRVLDDHETFSSVVSAHLSVP
SGMDPPQHTAFRQLVERYFEPERIKAFEPICREISKKLVCELPRDAEIDLVTQFAQLYAVRIQCAFLGWPDSL
QGPLLDWVHKNHAATLARDTKAMAAIALEFDEYIRDLLDERRKLGADAPDDVTTRLLRDRIDRRNLTHEEIVS
ILRNWTVGELGTITACVGILCHYLAKSQQTQALMRGGPDLLPAAIDEILRLHAPLISNRRVTTRAVVVGGREI
PAGEKITLMWASANRDEAVFDKPDELRLNRDPALNLLYGRGIHVCPGAELARAELRILMEELLGQTRKLDLVP
GSVPALAVYPASGFSRLPARIS

>CYP289A1(DK49_2450)*Brucella abortus* 63 75

MTSPTERPKQDWDPRSEAVLSDQIGAYDAMRHQCPVAHSDYLGWSLFSYDDVVRVLDDHETFSSVVSAHLSVP
SGMDPPQHTAFRQLVERYFEPERIKAFEPICREISKKLVCELPRDAEIDLVTQFAQLYAVRIQCAFLGWPDSL
QGPLLDWVHKNHAATLARDTKAMAAIALEFDEYIRDLLDERRKLGADAPDDVTTRLLRDRIDRRNLTHEEIVS
ILRNWTVGELGTITACVGILCHYLAKSQQTQALMRGGPDLLPAAIDEILRLHAPLISNRRVTTRAVVVGGREI
PAGEKITLMWASANRDEAVFDKPDELRLNRDPALNLLYGRGIHVCPGAELARAELRILMEELLGQTRKLDLVP
GSVPALAVYPASGFSRLPARIS

>CYP289A1(DK48_3007)*Brucella abortus* BDW

MTSPTERPKQDWDPRSEAVLSDQIGAYDAMRHQCPVAHSDYLGWSLFSYDDVVRVLDDHETFSSVVSAHLSVP
SGMDPPQHTAFRQLVERYFEPERIKAFEPICREISKKLVCELPRDAEIDLVTQFAQLYAVRIQCAFLGWPDSL
QGPLLDWVHKNHAATLARDTKAMAAIALEFDEYIRDLLDERRKLGADAPDDVTTRLLRDRIDRRNLTHEEIVS
ILRNWTVGELGTITACVGILCHYLAKSQQTQALMRGGPDLLPAAIDEILRLHAPLISNRRVTTRAVVVGGREI
PAGEKITLMWASANRDEAVFDKPDELRLNRDPALNLLYGRGIHVCPGAELARAELRILMEELLGQTRKLDLVP
GSVPALAVYPASGFSRLPARIS

>CYP289A1(DK53_2792)*Brucella abortus* bv. 9 C68

MTSPTERPKQDWDPRSEAVLSDQIGAYDAMRHQCPVAHSDYLGWSLFSYDDVVRVLDDHETFSSVVSAHLSVP
SGMDPPQHTAFRQLVERYFEPERIKAFEPICREISKKLVCELPRDAEIDLVTQFAQLYAVRIQCAFLGWPDSL
QGPLLDWVHKNHAATLARDTKAMAAIALEFDEYIRDLLDERRKLGADAPDDVTTRLLRDRIDRRNLTHEEIVS
ILRNWTVGELGTITACVGILCHYLAKSQQTQALMRGGPDLLPAAIDEILRLHAPLISNRRVTTRAVVVGGREI
PAGEKITLMWASANRDEAVFDKPDELRLNRDPALNLLYGRGIHVCPGAELARAELRILMEELLGQTRKLDLVP
GSVPALAVYPASGFSRLPARIS

>CYP289A1(DK51_2277)*Brucella abortus* BER

MTSPTERPKQDWDPRSEAVLSDQIGAYDAMRHQCPVAHSDYLGWSLFSYDDVVRVLDDHETFSSVVSAHLSVP
SGMDPPQHTAFRQLVERYFEPERIKAFEPICREISKKLVCELPRDAEIDLVTQFAQLYAVRIQCAFLGWPDSL
QGPLLDWVHKNHAATLARDTKAMAAIALEFDEYIRDLLDERRKLGADAPDDVTTRLLRDRIDRRNLTHEEIVS
ILRNWTVGELGTITACVGILCHYLAKSQQTQALMRGGPDLLPAAIDEILRLHAPLISNRRVTTRAVVVGGREI
PAGEKITLMWASANRDEAVFDKPDELRLNRDPALNLLYGRGIHVCPGAELARAELRILMEELLGQTRKLDLVP
GSVPALAVYPASGFSRLPARIS

>CYP289A1(DO78_2265)*Brucella abortus* NCTC 10505

MTSPTERPKQDWDPRSEAVLSDQIGAYDAMRHQCPVAHSDYLGWSLFSYDDVVRVLDDHETFSSVVSAHLSVP
SGMDPPQHTAFRQLVERYFEPERIKAFEPICREISKKLVCELPRDAEIDLVTQFAQLYAVRIQCAFLGWPDSL
QGPLLDWVHKNHAATLARDTKAMAAIALEFDEYIRDLLDERRKLGADAPDDVTTRLLRDRIDRRNLTHEEIVS
ILRNWTVGELGTITACVGILCHYLAKSQQTQALMRGGPDLLPAAIDEILRLHAPLISNRRVTTRAVVVGGREI
PAGEKITLMWASANRDEAVFDKPDELRLNRDPALNLLYGRGIHVCPGAELARAELRILMEELLGQTRKLDLVP
GSVPALAVYPASGFSRLPARIS

>CYP289A1(DK62_3038)*Brucella melitensis* bv. 3 Ether

MTSPTERPKQDWDPRSEAVLSDQIGAYDAMRHQCPVAHSDYLGWSLFSYDDVVRVLDDHETFSSVVSAHLSVP
SGMDPPQHTAFRQLVERYFEPERIKAFEPICREISKKLVCELPRDAEIDLVTQFAQLYAVRIQCAFLGWPDSL
QGPLLDWVHKNHAATLARDTKAMAAIALEFDEYIRDLLDERRKLGADAPDDVTTRLLRDRIDRRNLTHEEIVS
ILRNWTVGELGTITACVGILCHYLAKSQQTQALMRGGPDLLPAAIDEILRLHAPLILNRRVTTRAVVVGGREI
PAGEKITLMWASANRDEAVFDKPDELRLNRDPALNLLYGRGIHVCPGAELARAELRILMEELLGQTRKLDLVP
GSVPALAVYPASGFSRLPARIS

>CYP289A1(BAA13334_II01526)*Brucella abortus* A13334

MTSPTERPKQDWDPRSEAVLSDQIGAYDAMRHQCPVAHSDYLGWSLFSYDDVVRVLDDHETFSSVVSAHLSVP
SGMDPPQHTAFRQLVERYFEPERIKAFEPICREISKKLVCELPRDAEIDLVTQFAQLYAVRIQCAFLGWPDSL
QGPLLDWVHKNHAATLARDTKAMAAIALEFDEYIRDLLDERRKLGADAPDDVTTRLLRDRIDRRNLTHEEIVS
ILRNWTVGELGTITACVGILCHYLAKSQQTQALMRGGPDLLPAAIDEILRLHTPLISNRRVTTRAVVVGGREI
PAGEKITLMWASANRDEAVFDKPDELRLNRDPALNLLYGRGIHVCPGAELARAELRILMEELLGQTRKLDLVP
GSVPALAVYPASGFSRLPARIS

>CYP289A1(BSSP1_II0342)*Brucella suis* bv. 2 Bs143CITA

MTSPTERPKQDWDPRSEAVLSDQIGAYDAMRHQCPVAHSDYLGWSLFSYDDVVRVLDDHETFSSVVSAHLSVP
SGMDPPQHTAFRQLVERYFEPERIKAFEPICREISKKLVCELPRDAEIDLVTQFAQLYAVRIQCAFLGWPDSL
QGPLLDWVHKNHAATLARDTKAMAAIALEFDEYIRDLLDERRKLGADAPDDVTTRLLRDRIDGRNLTHEEIVS
ILRNWTVGELGTITACVGILCHYLAKSQQTQALMRGGPDLLPAAIDEILRLHAPLISNRRVTTRAVVVGGREI
PAGEKITLMWASANRDEAVFDKPDELRLNRDPALNLLYGRGIHVCPGAELARAELRILMEELLGQTRKLDLVP
GSVPALAVYPASGFSRLPARIS

>CYP289A1(BSPT1_II0341)*Brucella suis* bv. 2 PT09143

MTSPTERPKQDWDPRSEAVLSDQIGAYDAMRHQCPVAHSDYLGWSLFSYDDVVRVLDDHETFSSVVSAHLSVP
SGMDPPQHTAFRQLVERYFEPERIKAFEPICREISKKLVCELPRDAEIDLVTQFAQLYAVRIQCAFLGWPDSL
QGPLLDWVHKNHAATLARDTKAMAAIALEFDEYIRDLLDERRKLGADAPDDVTTRLLRDRIDGRNLTHEEIVS
ILRNWTVGELGTITACVGILCHYLAKSQQTQALMRGGPDLLPAAIDEILRLHAPLISNRRVTTRAVVVGGREI
PAGEKITLMWASANRDEAVFDKPDELRLNRDPALNLLYGRGIHVCPGAELARAELRILMEELLGQTRKLDLVP
GSVPALAVYPASGFSRLPARIS

>CYP289A1(BSPT2_II0343)*Brucella suis* bv. 2 PT09172

MTSPTERPKQDWDPRSEAVLSDQIGAYDAMRHQCPVAHSDYLGWSLFSYDDVVRVLDDHETFSSVVSAHLSVP
SGMDPPQHTAFRQLVERYFEPERIKAFEPICREISKKLVCELPRDAEIDLVTQFAQLYAVRIQCAFLGWPDSL
QGPLLDWVHKNHAATLARDTKAMAAIALEFDEYIRDLLDERRKLGADAPDDVTTRLLRDRIDGRNLTHEEIVS

ILRNWTVGELGTITACVGILCHYLAKSQQTQALMRGGPDLLPAAIDEILRLHAPLISNRRVTTRAVVVGGREI
PAGEKITLMWASANRDEAVFDKPDELRLNRDPALNLLYGRGIHVCPGAELARAELRILMEELLGQTRKLDLVP
GSVPALAVYPASGFSRLPARIS

>CYP289A1(BSSP2_II0347)*Brucella suis* bv. 2 Bs364CITA

MTSPTERPKQDWDPRSEAVLSDQIGAYDAMRHQCPVAHSDYLGWSLFSYDDVVRVLDDHETFSSVVSAHLSVP
SGMDPPQHTAFRQLVERYFEPERIKAFEPICREISKKLVCELPRDAEIDLVTQFAQLYAVRIQCAFLGWPDSL
QGPLLDWVHKNHAATLARDTKAMAAIALEFDEYIRDLLDERRKLGADAPDDVTTRLLRDRIDGRNLTHEEIVS
ILRNWTVGELGTITACVGILCHYLAKSQQTQALMRGGPDLLPAAIDEILRLHAPLISNRRVTTRAVVVGGREI
PAGEKITLMWASANRDEAVFDKPDELRLNRDPALNLLYGRGIHVCPGAELARAELRILMEELLGQTRKLDLVP
GSVPALAVYPASGFSRLPARIS

>CYP289A1(DK65_2119)*Brucella pinnipedialis* 6/566

MTSPTERPKQDWDPRSEAVLSDQIGAYDAMRHQCPVAHSDYLGWSLFSYDDVVRVLDDHETFSSVVSAHLSVP
SGMDPPQHTAFRQLVERYFEPERIKAFEPICREISKKLVCELPRDAEIDLVTQFAQLYAVRIQCAFLGWPDSL
QGPLLDWVHKNHAATLARDTKAMAAIALEFDEYIRDLLDERRKLGADAPDDVTTRLLRDRIDGRNLTHEEIVS
ILRNWTVGELGTITACVGILCHYLAKSQQTQALMRGGPDLLPAAIDEILRLHAPLISNRRVTTRAVVVGGREI
PAGEKITLMWASANRDEAVFDKPDELRLNRDPALNLLYGRGIHVCPGAELARAELRILMEELLGQTRKLDLVP
GSVPALAVYPASGFSRLPARIS

>CYP289A1(V910_200853)*Brucella ceti* TE10759-12

MTSPTERPKQDWDPRSEAVLSDQIGAYDAMRHQCPVAHSDYLGWSLFSYDDVVRVLDDHETFSSVVSAHLSVP
SGMDPPQHTAFRQLVERYFEPERIKAFEPICREISKKLVCELPRDAEIDLVTQFAQLYAVRIQCAFLGWPDSL
QGPLLDWVHKNHAATLARDTKAMAAIALEFDEYIRDLLDERRKLGADAPDDVTTRLLRDRIDGRNLTHEEIVS
ILRNWTVGELGTITACVGILCHYLAKSQQTQALMRGGPDLLPAAIDEILRLHAPLISNRRVTTRAVVVGGREI
PAGEKITLMWASANRDEAVFDKPDELRLNRDPALNLLYGRGIHVCPGAELARAELRILMEELLGQTRKLDLVP
GSVPALAVYPASGFSRLPARIS

>CYP289A1(BMEA_B0369)*Brucella melitensis* ATCC 23457

MTSPTERPKQDWDPRSEAVLSDQIGAYDAMRHQCPVAHSDYLGWSLFSYDDVVRVLDDHETFSSVVSAHLSVP
SGMDPPQHTAFRQLVERYFEPERIKAFEPICREISKKLVCELPRDAEIDLVTQFAQLYAVRIQCAFLGWPDSL
QGPLLDWVHKNHAATLARDTKAMAAIALEFDEYIRDLLDERRKLGADAPDDVTTRLLRDRIDRRNLTYEEIVS
ILRNWTVGELGTITACVGILCHYLAKSQQTQALMRGGPDLLPAAIDEILRLHAPLISNRRVTTRAVVVGGREI
PAGEKITLMWASANRDEAVFDKLDELRLNRDPALNLLYGRGIHVCPGAELARAELRILMEELLGQTRKLDLVP
GSVPALAVYPASGFSRLPARIS

>CYP289A1(DK67_2394)*Brucella suis* bv.3

MTSPTERPKQDWDPRSEAVLSDQIGAYDAMRHQCPVAHSDYLGWSLFSYDDVVRVLDDHETFSSVVSAHLSVP
SGMDPPQHTAFRQLVERYFEPERIKAFEPICREISKKLVCELPRDAEIDLVTQFAQLYAVRIQCAFLGWPDSL
QGPLLDWVHKNHAATLACDTKAMAAIALEFDEYIRDLLDERRKLGADAPDDVTTRLLRDRIDGRNLTHEEIVS
ILRNWTVGELGTITACVGILCYYLAKSQQTQALMRGGPDLLPAAIDEILRLHAPLISNRRVTTRAVVVGGREI
PAGEKITLMWASANRDEAVFDKPDELRLNRDPALNLLYGRGIHVCPGAELARAELRILMEELLGQTRKLDLVP
GSVPALAVYPASGFSRLPARIS

>CYP289A1(IY71_13010)*Brucella suis* ZW043

MTSPTERPKQDWDPRSEAVLSDQIGAYDAMRHQCPVAHSDYLGWSLFSYDDVVRVLDDHETFSSVVSAHLSVP
SGMDPPQHTAFRQLVERYFEPERIKAFEPICREISKKLVCELPRDAEIDLVTQFAQLYAVRIQCAFLGWPDSL
QGPLLDWVNKNHAATLACDTKAMAAIALEFDEYIRDLLDERRKLGADAPDDVTTRLLRDRIDGRNLTHEEIVS
ILRNWTVGELGTITACVGILCHYLAKSQQTQALMRGGPDLLPAAIDEILRLHAPLISNRRVTTRAVVVGGREI
PAGEKITLMWASANRDEAVFDKPDELRLNRDPALNLLYGRGIHVCPGAELARAELRILMEELLGQTRKLDLVP
GSVPALAVYPASGFSRLPARIS

>CYP289A1(IY72_12550)*Brucella suis* ZW046

MTSPTERPKQDWDPRSEAVLSDQIGAYDAMRHQCPVAHSDYLGWSLFSYDDVVRVLDDHETFSSVVSAHLSVP
SGMDPPQHTAFRQLVERYFEPERIKAFEPICREISKKLVCELPRDAEIDLVTQFAQLYAVRIQCAFLGWPDSL
QGPLLDWVNKNHAATLACDTKAMAAIALEFDEYIRDLLDERRKLGADAPDDVTTRLLRDRIDGRNLTHEEIVS
ILRNWTVGELGTITACVGILCHYLAKSQQTQALMRGGPDLLPAAIDEILRLHAPLISNRRVTTRAVVVGGREI
PAGEKITLMWASANRDEAVFDKPDELRLNRDPALNLLYGRGIHVCPGAELARAELRILMEELLGQTRKLDLVP
GSVPALAVYPASGFSRLPARIS

>CYP289A1(BCAN_B0391)*Brucella canis* ATCC 23365

MTSPTERPKQDWDPRSEAVLSDQIGAYDAMRHQCPVAHSDYLGWSLFSYDDVVRVLDDHETFSSVVSAHLSVP
SGMDPSQHTAFRQLVERYFEPERIKAFEPICREISKKLACELPRDAEIDLVTQFAQLYAVRIQCAFLGWPDSL
QGPLLDWVHKNHAATLACDTKAMAAIALEFDEYIRDLLDERRKLGADAPDDVTTRLLRDRIDGRNLTHEEIVS
ILRNWTVGELGTITACVGILCHYLAKSQQTQALMRGGPDLLPAAIDEILRLHAPLISNRRVTTRAVVVGGREI
PAGEKITLMWASANRDEAVFDKPDELRLNRDPALNLLYGRGIHVCPGAELARAELRILMEELLGQTRKLDLVP
GSVPALAVYPASGFSRLPARIS

>CYP289A1(BCA52141_II0654)*Brucella canis* HSK A52141

MTSPTERPKQDWDPRSEAVLSDQIGAYDAMRHQCPVAHSDYLGWSLFSYDDVVRVLDDHETFSSVVSAHLSVP
SGMDPSQHTAFRQLVERYFEPERIKAFEPICREISKKLACELPRDAEIDLVTQFAQLYAVRIQCAFLGWPDSL
QGPLLDWVHKNHAATLACDTKAMAAIALEFDEYIRDLLDERRKLGADAPDDVTTRLLRDRIDGRNLTHEEIVS
ILRNWTVGELGTITACVGILCHYLAKSQQTQALMRGGPDLLPAAIDEILRLHAPLISNRRVTTRAVVVGGREI
PAGEKITLMWASANRDEAVFDKPDELRLNRDPALNLLYGRGIHVCPGAELARAELRILMEELLGQTRKLDLVP
GSVPALAVYPASGFSRLPARIS

>CYP289A1(DK60_2113)*Brucella canis* RM6/66

MTSPTERPKQDWDPRSEAVLSDQIGAYDAMRHQCPVAHSDYLGWSLFSYDDVVRVLDDHETFSSVVSAHLSVP
SGMDPSQHTAFRQLVERYFEPERIKAFEPICREISKKLACELPRDAEIDLVTQFAQLYAVRIQCAFLGWPDSL
QGPLLDWVHKNHAATLACDTKAMAAIALEFDEYIRDLLDERRKLGADAPDDVTTRLLRDRIDGRNLTHEEIVS
ILRNWTVGELGTITACVGILCHYLAKSQQTQALMRGGPDLLPAAIDEILRLHAPLISNRRVTTRAVVVGGREI
PAGEKITLMWASANRDEAVFDKPDELRLNRDPALNLLYGRGIHVCPGAELARAELRILMEELLGQTRKLDLVP
GSVPALAVYPASGFSRLPARIS

>CYP289A1(DA85_12335)*Brucella canis* SVA13

MTSPTERPKQDWDPRSEAVLSDQIGAYDAMRHQCPVAHSDYLGWSLFSYDDVVRVLDDHETFSSVVSAHLSVP
SGMDPSQHTAFRQLVERYFEPERIKAFEPICREISKKLACELPRDAEIDLVTQFAQLYAVRIQCAFLGWPDSL
QGPLLDWVHKNHAATLACDTKAMAAIALEFDEYIRDLLDERRKLGADAPDDVTTRLLRDRIDGRNLTHEEIVS
ILRNWTVGELGTITACVGILCHYLAKSQQTQALMRGGPDLLPAAIDEILRLHAPLISNRRVTTRAVVVGGREI
PAGEKITLMWASANRDEAVFDKPDELRLNRDPALNLLYGRGIHVCPGAELARAELRILMEELLGQTRKLDLVP
GSVPALAVYPASGFSRLPARIS

>CYP289A20(HYPDE_25418)*Hyphomicrobium denitrificans* 1NES1

MKADSNKREQDWNPRSDLVLSNQIKAYDTMRRRCPVAYSDYLGWSLFRHEDVVRVLNDHHTFSNVVSRHLSVP
SGMDPPQHTAYRRLIERHFEPMRVQAFEPRCRAISADLVSRLERDAEIDLVTQLARLFAVRIQCAFLGWPEEL
HDPLLRWVRKNHEATLARNVEAMAAVALEFDGYISELLDARRAGAAAPDDITTDLLREKIDGRPLSHEEIVS
ILRNWTVGELGTITACVGILAHYLAERPQIQQQLRLNLHQLPAAIDEILRIHAPLISNRRVAISPIEIGGRKI
AAGERITLMWASANRDEAVFGDPDEFRLDRDPAQNLLYGRGIHVCPGALLARLELRVIMEELLKQTRQIAFVA
DRPPTLAVYPAGGFATLPMRIS

>CYP289A21(Hden_0569)*Hyphomicrobium denitrificans* ATCC 51888

MTADGKLLARGGRNDAKQQSISIVPAECFQEMTVKTELTKREQDWNPRSDLVLSNQIKAYDAMRQRCPVAYSD
YLGWSLFRHEDVVRVLDDHETFSSVVSSHLSVPSGMDPPEHTAYRKLIDRHFEPARVEAFEPQCRAISRDLVS

RLQRNAEIDLVVDLARLFAVRIQCAFLGWPDELHEPLLQWVRKNHEATLARDVEAMAAVALEFDGYITALLDA
RRQAGASAPDDITTDLVREKINGRPLKDDEIVSILRNWTVGELGTITACVGILAHYLAEHSQVQKQLRANLDQ
LPAAIDEILRIHAPLISNRRVAHRPVEIDGRKIAAGERITLMWASANRDEAVFENTDELRLDRDPEQNLLYGR
GIHVCPGAGLSRLELRVIMEELLRATETMAFIPGRPATLAVYPAGGFAALPMRIA

>CYP289A22(OV14_a0758)*Ensifer adhaerens* OV14

MESAAGEASNQHKSSMETVSDWDPRSREVLANQIQAYDQMREACPVAYSDYLGWSLFRHTDVLQVLNDHETYS
NVVSRHPSVPNGMDPPEHKHYRQMIEPYFAVERIEAFEPACRWIARDLVASLKKDPSSEFLMGFAEPYAIRIQ
CGFLGWPRSLHEPLRQWVRKNREATLVRDANALASIALEFDGYIRSQLEIRQQAGAAAPDDITTSLVRQAVDG
RLLRHAEIVSILRNWTVGELGTISACVGIIVHFLAEHPEVQSQLRRDRAFLPQAVDEILRLHGPLISNRRVAS
RSVAIGERTIKAGERMTVIWASANRDGAVFEDADQFRPDRDQSQNLLYGAGIHICPGAALARMQLSVVLDELL
SGIGNFVLPLGTEAIRAVYPAGGYASFNLQIN

>CYP290A1(CC_0946) *Caulobacter vibrioides* CB15

MDLISQTVVDGKAGPGAPPTYPTLKDVDLADIFRFTKGQPWADFARMRQEAPVMWHPEPMGGPGFWALTRYED
VHRVNGDPETFSSQRGGILMSMGAPEKRHALLFRASMDTMINMDAPHHLQLRREHMPYFTPSYLRGLTERVKG
EVTRLLDEMEPLLANGAEIDMVEHFSSVLPLFTLCEILGVPPEDRPKFLTWMHYLERAQDLAVKQANAPMQPT
LELMQFVMDFNNNVEEMFEYGRTMLHKRREDPKEDLMTAIARAQLDGAVLPDEYLDGSWLLIVFAGNDTTRNT
LSGAMRLLTEFPDQKQKLIADPSLLGGAVDEFIRMVSPVVYMRRTATRDVEVNGQLIREGEKAIMYYGAANRD
PAMFENPDQLDVTRANAGKHIAFGYGPHTCIGKRVAQIQLEEAYRQILARFPDLNWTGNIEIAPNNFVHAISK
LGVKRG

>CYP290A1(CCNA_00995) *Caulobacter vibrioides* NA1000

MDLISQTVVDGKAGPGAPPTYPTLKDVDLADIFRFTKGQPWADFARMRQEAPVMWHPEPMGGPGFWALTRYED
VHRVNGDPETFSSQRGGILMSMGAPEKRHALLFRASMDTMINMDAPHHLQLRREHMPYFTPSYLRGLTERVKG
EVTRLLDEMEPLLANGAEIDMVEHFSSVLPLFTLCEILGVPPEDRPKFLTWMHYLERAQDLAVKQANAPMQPT
LELMQFVMDFNNNVEEMFEYGRTMLHKRREDPKEDLMTAIARAQLDGAVLPDEYLDGSWLLIVFAGNDTTRNT
LSGAMRLLTEFPDQKQKLIADPSLLGGAVDEFIRMVSPVVYMRRTATRDVEVNGQLIREGEKAIMYYGAANRD
PAMFENPDQLDVTRANAGKHIAFGYGPHTCIGKRVAQIQLEEAYRQILARFPDLNWTGNIEIAPNNFVHAISK
LGVKRG

>CYP290A1(Cseg_3349)*Caulobacter segnis*

MDLISQTVVDGVAGPGTPPTYPTLEGVDLADIFRFTKGQPWADFARMRREAPVMWHREPFDGPGFWALTRYED
VHRVNGDPEAFSSQRGGILMSMGAPEKRHALLFRASMDTMINMDAPHHLQLRREHMPYFTPAYLRGLTERVKG
EVTRLLDEMEPKLAGGAAIDMVEHFSSVLPLFTLCEILGVPPEDRPKFLTWMHYLERAQDLAVQQANAPLQPT
LELMQFVMDFNNNVEEMFEYGRTMLHKRRQDPKEDLMTAIARAQVDGEVLSDEYLDGSWLLIVFAGNDTTRNT
LSGAMKLLTEFPEQKQQLIADPSLLPGAVDEFIRMVSPVIYMRRTATRDVEVNGQRIREGEKVIMYYGAANRD
PAMFENPDQLDVTRANAGKHIAFGYGPHTCVGKRVAQIQLEEAYRQILARFPDLNWTGDIEIAPNNFVHAISK
LGVKRG

>CYP290A2(Caul_4405)*Caulobacter* sp. K31

MTMQLVSETATDGVPGPGAPTLFPILADIDLSNIQQFTQGQPYGDFARLRAQAPVMWHHEPYGGPGFWAITRH
ADIMAVEADPQSFSSRKGGILMAHGDPQKRHALLYRASMDAMINLDGAEHMQLRREHMAYFTPAYLRGVTEAV
RAEVGRLLDEMAPLGHCDLVEAFSSKLPLFTLCEILGVPAEDRGKFLTWMHYLEMAQDLALKQHLAPITPTLE
LMQFVQDFNTNVEEMFEYGRAMLHKRRLEPKNDLMSAIARAQVDGNLLSDQYLDGSWLLIVFAGNDTTRNTLS
GVMKLLTEFPDQKARLIADPSLMPGAVNEFIRMVSPVIYMRRTATRDAEIAGQAIREGEKVVMYYGAANRDQA
VFDNPDQLDITRSNADKHIAFGYGPHACIGKRVAQIQLEEAYRQILARFPDIHWTGEIEIAPNNFVHAISKLD
VAFTPGGRA

>CYP290A3(HNE_0938)*Hyphomonas neptunium* ATCC15444

MNVTETYQVESPPKEPKFGKAYALTPPLDLTQPTVFSEHGGYTYADFAKMREKAPVMWHPEEYGAGFWAVTSY
DLVKKVELTPETFSSQKGGILMNYGMEGVPRHPLLHSSSLNSLINLDRPYHTPLRMEHMAYFRADFVAELRKR

VDVEVNRLLDTMEAQGPVVDMVEMFSAELPLFTLCEILGVPPADRPKLVHWMHFLETSQYQAQQEGLGNVSQE
QIMAFLNEIQAMFEYGRYLLEERRKNPQPDLLSAIANVQVDGKPLSPEFLDGSWLLIVFAGNDTTRNSLSGTM
RLLTENPELREQLKEKPERFPNFVHEAIRMVTPVTYMRRTATQDTELGDQKIAEGEKVIMYYAAANRDPSKFE
NPDRFDIDRSNAKEHLAFGHGPHVCLGQRVANMQLESAYRQILSRFPNVRWTGEQTIAPNNFVHAISSLMVDL
GK

>CYP290B2(Mmar10_1670)*Maricaulis maris*

MQLVSDVILEDRPQTEPGFGATYPGFDDFKLWDPAAWTSGHPHDFFRKMREQAPVMWQSADKSKAGYWSVTRY
DDIKTVELAPEIYSSERGSINMFVMPRKEWKPKKLIPAAFNSIINLDQPHHMQMRIQQKDFFIPRFVSELQER
VDAKIDDLLDEMERRGPVVDFVKLFSQELPLFTLCEMLGVDEADRPKIIEWMHYLELANQYFVNPWSLLSRRP
WFPIKFYHHVNAMFAYGERVMAERRANPRRDLMTAIANTEMAGKPLPQEYLDGSWLLIIFAGNDTSRNSLSGT
IRLMTQFEDQREAILSDPSLIPAMTNEALRMISPVQHMRRTAMEDTELNGQRIARDEKVVLWYPAANRDPSVF
PDPDRFDMTRENGDKHLAFGHGAHKCLGSRIAQMQLQSAFRKIFERFPKIAWTGRQTIAPNTLVHAISSLEVN
LYGRDGERPRPVQERWP

>CYP1010A1(Plav_0082)*Parvibaculum lavamentivorans*

MAMRAERFDEEAPEIFIPARPVPPKEAPGIGAAVFRPQLLANLIASYPEFWYSARSCPFRVGIARGGRGMLVN
DPDAIRRILVSDAEHFPKDDNQLAILKPLLGNGLLTAEGATWRRNRKLAAPIFQHSSVRDFAPLFVRAAERSA
RRALEQQGFFPLDREMTKLTLEIIGETVLSANLEDDIDGISHTVTSVLDKFPAMFLASAFLPGQLRNRVIDTV
VRPGRRALDVFARRIIDEARKSGEETTLLQRLMVSQSKAGHEMTLDQVRDEVATFLLAGHETTATTMSWVWYL
LTVHPEWQERLYEEVWAVTEGRRLTIDDVPALVETRAVIEEALRLYPPVANLMRRAIKTTELTPDITIERGQT
VLISPWLLHRHRFFWREPDRFDPTRFLGEEAATRPRHLYIPFGGGPRICIGASFALLEAVLILATFMQRARVK
VINADQVMPQARIVLRPNVALQAVVTPRRPGG

>CYP1017A1(Swit_1021)*Sphingomonas wittichii*

MSGSRDIDHHAVAFARDHLAIYRDARAHCPVLRSDAHGGFAILTRYADNRAALRNHDTFASGRIRAGDRLGGG
VAIPPNGLRIGMIEMDGAEARALRALLQPWFTIAAVEAAAPRMAQLSAWLVDRIIARGACDVVEDLAKPMPSL
LILDILGLPLDRWRDYGRVLHEAVAKSSGSIDGLRWLASDLRASVERREVHPEGLIAAMIAAEVDGRPLGDAL
VCELAMMLLFGGTDTTIAAIGHALRHLTEHPADRAALVARPALIPAAVEEILRLYSPSTGVARTVTAPVEIGG
ERFEPGQRVLCAINSANRDEEMFADAERFDLDRPKRPHLAFGWGVHACLGQNLARADLRILLGELLARMPDFA
VDVAASERYASIPLVNGHARMPMRFTPGTPAGTGGGELPILTAPRLRPLP

>CYP1036B2(BF49_6347)*Bradyrhizobium* sp. BF49

MSTVTNCPAYDVDLYDDDVLRDPYPHYRALRELGAVVWLPRNGLYALGRFEDVRAALRNPGSFSSAEGVAANA
AVNEMSKGTTLASDAPLHDRLRAIIAAPMLPRALEEIGPQIRAEARQLVDGLVRRGQFDAVADLARHLPLTIV
SKLVGLEDHGRGSMLRWAAATFNVLGAMNERACAAMTDVQEMRGYLGGAAIRERLRPGSWGERIFEAGDRGEV
EPERCPVLMRDYLGPSLDTTIFATANLILLFGRHPEQWDLVRQDPALIPNAINEALRLESPIRGFTRYLAADT
KIGEAVIPAGNRVLLLYASANRDERKWQDPERFDVRRRASDHLGFGNGTHMCAGLHLARLEMTALLEVLVEKV
ARFDIGEPVLALNNVLRGLASLPVRVA

>CYP1036C2(Swit_3025)*Sphingomonas wittichii*

MAAQAASIDIDLFDAETRDDPYAAYRRLRDAGPVCHIERHGLYAMGRYRDLRDALTNWTDFSSGSGVAMNDLM
NRLMAGTVLASDPPQHKRLRQILGMPIGPARLGKLRPHMRGLAMERVRELKGGGRFDAMRRLAMLLPLSVVSD
LVGLPEEGRERMLDWAAAAFNGMAPAGVPLSDGAFPVMKEMVDYITDPDLIGKLRPDGWAAQLWSSVADGTLT
EEEYRSIIQGYVSPSLDTTIFAVGNLLWLLATHPDQWRALKATPALQARCINESLRHESPALGFSRLAKRDLV
IDGVAVPAGARIMTVLPAANRDERHYADPERFDIQRDASDHLAFGGGVHRCIGGSLAMLEIGAVLEALVELVD
HIEPGDGRRADNAVLRGFDHLEISLH

>CYP1061B1(Jann_2272)*Jannaschia* sp. CCS1

MAWPHIITDMHDISAGLRHPDLKQALYDDGAVIMADTLLTLHGEPHRNRRKLEFRVFRKDYFHWYERTVFPDT
VRSSLSDDLAAGRSELVDLGYRVTMNLTADFAGIDRPEQSAEETGRLLRLVATFSEGATIVHSKRPKDDVRAE
VRAAIADFEPHFLSASVARRRALLADHAAGRIDEDALPRDVLTVILRDGDPTEFTPEVLTREIGFYLQAGAHS

TANSTIHAFHDIYTWAEERSERWDQLREDPIFFQRCVHESLRLHPASPVAWRAATCPMSLPGAGEVAEGEMVE
FRLAEANRDPAVFGPDADVFNPYRTVKAPTLPFGHTFGTGVHTCLGRDLDGGAVPKGDVDPETHPYGTITLLL
RELFAHGARPDPTDPPQISTKTERPNWGHYPIIFDPKKAWR

>CYP1061B2(A6F65_02129)*Altererythrobacternamhicola*

MQVHRLADYRDCEAALRNTGLRQALYDAGEVITKDTLLVLHGEAHQKRREVEIRVFRRNFFKYYEKEVFPATL
EETIAPFVAAGGGDLIELGYRLTVNLTADFAGVDRPARTPEETAQLIRLVKKMSEGATMVHSTRDKDELTAEV
REALAEFDRDFLQASIARREDLIAQVERGEVEEDTLPRDVLTVILKAQDELQFTQAQRVREIGFYMQAGSHST
ANSVVHALEEIQTWAGEDQARWKRLEDPVFVQHCVHESLRLHPASPEAWRKSVCPMSVQGAGDMPEGDRVELD
LFNANRSEAIFGSDAGTFNPDRDVPVGVMRSGLAFGIGVHVCLGRELDGGLVAKPGTDPAKAQFGIVPLIVIK
LLELGARRLRDDPPTPDTKTKRPNWGRYPVKFTKEIA

>CYP1068A4(ACP90_17935)*Labrenzia* sp. CP4

MSPSTDLSFAPLSDAFARDPYPFYAALRDRKGLTYFEDFDIWLAATFDDVSEIVQNGKMVRSMDHIASAEEIA
DMKRAQNWHDMPHHSRFVQFSLLDSDGDVHDRLRKQVFRLFTPVMVGKLRDDIQAYVDRLFDSLSDRTEIDFI
EDLAAHVPGHIIGRVLGVPDEDCPQLRIWSENIVQYFDIDRTDERKELAERNTTEFYLYLQTLKAERERAPQD
DLLSLMIEAERAGQMNEDEFISTAMLILMAGHGSTIDVLGSGMHALLKFPKELQRLREDPTLMKTAVQEMFRY
ESPLPFFHRYSTENMTVCGDAYPRGTKFGVLYGAANRDPAQFPDADRFDVGRTPNWHIAFGRGAHFCLGNHLA
RLDMDIIFSTLLKRFSTIELIDDNVTYKRGLSVRGPEALRLAWQPA

>CYP1068A5(OV14_a0406)*Ensifer adhaerens* OV14

MAETLSTESQVEVAFTPLSPDFARDPYPTYKALRDKDGLHYFEDFDIWLASRFEDVSAIVMDKRMVRSLEDSA
TSEEIARIQRAANWHDMPHHSRFVQFSLLDSDGAVHDRLRRQVFKLFTPAMIAKLRDQIQAYVDRLIDSLADR
GQIDFVDDLAAHVPGHIIGRLLGVPDEDCPQLRIWSENIVQFFDVDRSDERKRIAETNTTEFYEYLTALKHER
ERNPKDDLISVLIEAERAGAMNSDEFISTCMLILMAGHGSTIDVLGSGMHALLRFPEQMARLRKDPALIQTAV
QEMFRYESPLPFFHRHCIEDVEIGGRTFARGTKFGVLYGAANRDPKQFENADAFDIGRTPNRHLAFGGGVHFC
LGNHLARLDMDIIFSTLLRRFPTIELVDEEPEYKRGLSVRGPKRLQIAIKPA

>CYP1068A6(RTCIAT899_PC02800)*Rhizobium tropici*

MAMATPAFDPLSQAFTDDPYASYRVLRASPGPVYYEQFDIWLLSRYQDVAAAAADKTLVRSLEFFLSPEEIRE
QKVRQNWHDMPNHSRFVQFSLLDSDGETHDRLRRKVFREFTPALVARQRGMIQDFVDRLLDRLLQAGTIDFVE
DLASQVPGHIIGSVLGVPEEYAGQLRKWSEDVVQYFDVDRSDERKAIAERATTEFYELLKELIVERISTPRDD
LLCRLSEAHVAGELTEDELVSTCMLILMAGHGSTIDVLGSGMHALLQYPEALKKLRETPTVMSTAIQEMFRFE
SPLPFFHRYLTEERTIAGKLLPKGTKIGLLYGAANRDPEQFESPDEFDVGRTPNRHLAFGSGAHFCLGNHLAR
LDMEIIFSTLLQRTRSIELLDDNPAYKRGLSVRGPKRLDIRLIAA

>CYP1068A7(Astex_0364)*Asticcacaulis excentricus*

MRLPDPSAADPALFDPLGPEFTRNPYPAYARLRALNTPYYFAPFDIWLFARFADVQAAALNPLSLRSLDDRLT
PEEIEADRRAQNWHDMPYHARFVQFSLLNSDGAMHQRLRRLVFGEFTPPQVERLKSGVEAYVSDLFDAVGSEF
DFINDLAVHVPGRVIGRFLGVPDADCAQLRIWSEDIVQYFDIDRTDARKALAERTTRTFYDYLIDLSNERRKR
PQDDLISRLLRRWDAGELSEDEYISTCMLILMAGHGSTIDAMGNGLTALLNHPEQLQRLRADPGLMPTAIQEM
FRYDAPLPFFHRLASEDMDVNGWEIPKGTKIGLLYASANRDPEAFDNADRFEIGRTPNRHLAFAAGPHFCLGN
HLSRMNMEATFMTLLSRFQSIALTAEPQFKPSLSLRGPKALPLRVN

>CYP1075A1(Swit_0997)*Sphingomonas wittichii* RW1 Sphingomonas wittichii

MPDRNFTQADYNHHDPKIAQDPYGFLAKVRGGQCPLGHSDELGGFYFPTTYDGVKRVFSDYRTFTSNEGAGLP
AQLVRFFPVDLDPPNHTRWRRTLNRFFTQEAAEADRPRIQGLADKLIDAFVERGSADLVNEFTRPFLAMTMLP
VIGCPDDDRPMLSEKLMWMVHNRLLDHDGWVRRYTEIGDYLMGLAAARRVAPRRDDLLQCLIEEEFDGKRLSD
MEVYQVLILALFGALDSTSSAMSGSLFHLGQHPEDKRRLLSGEVAWPVAIEEFLRFTTPIQTLRRTVVQETEL
DGGALHPGDIVLCINGAANRDPDKFVDPDRCIIDRDARDHMSFGSGAHVCIGRHYARVMIDTCLKTMLGRIGD
YRIAPGFTPDYTSSEARALKTLMVEFTPGPRLLAG

>CYP1076A1(Swit_1743)*Sphingomonas wittichii* RW1 Sphingomonas wittichii

MSYQEHQAAQRLETIPPHVPADRVVDLDYFSPPGVAEDPHLAWKRLHDGPDIIYTPHYGGHWIVTRADDMFAV
MQDYETFSNWEFTVPKRGPGNPVLMPNQLDPPRHGPIRSVVLRPMSPKAVEPIEPVIRKIMADRIASIVPKGG
CEFVGEFGGDIPPELFFEHARIPKDKLPEMKRFADAVARSDNEEDRRAARIGGANYMMAILDERRQVEELDDD
VFSVIVRAEREGRITREESTSIALNVFFGGLETVSSALAFIVLFLARHPEHRRQLIEDASLTQDATEEFLRRF
GILNLARIATRSTTFKGLTIEQGEQVLLPLHLAGLDERKFPNPLEVDFHRKRAGHFNFGTGPHRCLGSNLARP
EIRIFIEEWLRQIPDFSVDPDDKPVGASGVAMTMTRVPLVWPSR

>CYP1077A1(Swit_1964)*Sphingomonas wittichii* RW1 Sphingomonas wittichii

MASAPDDQADKAVMDFDPAVCTYDRWIEKFGEAREKCPVFASEAHGGYWVAASYEHATNISRDWETFSSRKVW
DPEAGKLEGGSVIPPFAGPPFVPVETDPPEWKLYRHLLNPFFGPKAVETYRTRARQVMRALVDRVIETGACDI
VNDLANPLPALSTLDILGIPYDQSNWRRFSDPFHKLAYARGTAEFPQCLADLDWIRGQLAEQVERDRANPREG
LFSSLCNGEVAGKAFSVPEIVGLGMMVLVGGVGTTNALFSNSCVWLSKHPEVRRKLIDNPDMLPIAREEFVRF
YSPVHSAARLVTREVVVNGQRLEPGEQIMVAFSSANHDETVFENAGEIDVERFPNPHVGFGSGIHRCLGSFLA
RMFFETMFAEVMARMPDFLVDEERAVRYPDISTKNGWITIPATFTPGPRMGEGPTL

>CYP1078A1(NGR_b07660)*Sinorhizobium fredii* NGR234

MAHAVPVVSDIAVSDLLADPYPIYKRLREMAPAVYVDSARLALVTRFDDIMAIERDSETFASSNPGSLVNKVM
GHTMMRKDGEAHAMERRAVEISFRPGTIKNHWAAQFEATSDRLIADIESQGAADLFDTLAAPMASLALIELLG
LKDVSWQTLACWSQALMDGAGNYSGDPEISARATEAAAGVDAAIDAVIAWHRENPNPAVLSSMVNAENPMNIE
QIRANIKVIIGGGLNEPRDSILSLTLGLLSNPAQRAEVMANPDLGPLAFEEAIRWISPIGMYPRRVTRDVELS
GVELRAGDQIGLCVGAANRDDRRFDNPDSFDLFRPKQPHLAFGAGPHFCAGAWVARQMVGRIVVPMLFSRLKN
LRLRPDAPARIHGWVFRGPVSLPVAWDI

>CYP1078A2(SAMCFNEI73_pC1877)*Sinorhizobium americanum*

MAHAVLVVSDIAFSDLLSDPYPIYKRLRDMAPAVYVEAARLALVTRFDDIMTIERDCETFASSNPGSLVNKVM
GHTMMRKDGEAHAMERRAIEVSFRPGTIKNHWAARFEAISDRLISQIESDGEADLFDALAAPMASLALIELLG
LKDVSWQTLARWSQALMDGAGNYSGDPEISARATEAAAGVDAAIDAVLAWHRENPNPAVLSSMVNAEHPMNLE
QIRANIKVIIGGGLNEPRDSILSLTLGLLSNPLQRAEVMANPDLWPLAFEEAVRWISPIGMYPRRVTRDVELS
GVKLRAGDQIGLCVAAANRDDRRFANPDNFDVFRPKQPHLAFGAGPHFCAGAWVARQLVGRIVVPMLFSRLKN
LRLRPDAPARIHGWVFRGPVSLPVAWDL

>CYP1078A3(SFHH103_06380)*Sinorhizobium fredii* HH103

MAHAVPVVSDIAFSDLLADPYPIYKRLRDTAPAVYVDAARLALVTRFDDIMAIERDSETFASSNPGSLVNKVM
GHTMMRKDGEAHAIERRAIEVSFRPGTIKNHWAAQFEAISDGLISAIENQGEADLFDALAAPMASFALIELLG
LKDVSWQTLARWSQALMDGAGNYSGDPEISARATEAAAGIDAAIGAVLAWHRENPNPAVLSAMVNAENPMNLE
QIRANIKVIIGGGLNEPRDSILSLTLGLLCSPAQLAEVIANPDLWPLAFEETVRWISPIGMYPRRVTRDVELS
GVQLRAGDQIGLCVGAANRDDRRFEKPDSFDLFRPKQPHLAFGAGPHFCAGAWVARQMVGRIVVPTLFSRLKN
LRLRPDAPARIHGWVFRGPVSLPVTWDV

>CYP1078A4(USDA257_c26500)*Sinorhizobium fredii* USDA 257

MALAVPVVSDIAFSDLLADPYPIYKRLRDMASAVYVDTARLALVTRFDDIMTIERDSETFASSNPGSLVNKVM
GHTMMRKDGEAHAIERRAIEVSFRPGTIKNHWAAQFEAISDRLVSEIESRGEADLFDALAAPMASLALIELLG
LKDLSWQTLAHWSQALMDGAGNYSGDPAISARATGAAAGVDAAIDAVLPWHRQNPNPAVLSSMVNADHPMNLE
QIRANIKVIIGGGLNEPRDSILSLTLGLLSNPVQLATVMANPDLWPLALDEAVRWISPIGMYPRRVTRDVELS
GVELRAGDQLGLCVGAANRDDRRFENPDNFDLFRPKQPHLAFGAGPHFCAGAWVARQMVGRIVVPMLFSRLKN
LRLQPDAPARIHGWVFRGPVFLPVAWDV

>CYP1078A5(IE4771_PD00147)*Rhizobium etli* bv. mimosa IE4771

MLTEETAATPPVISDVTFAELLADPYPTFKRARDMAAVVRIEAANMLVATRYEDIMAMERDPELFSSENPQSL
VNKVMGHTMMRKDGEAHARERKAVEPALRPGAMKSCWGPRFETIFDEIVSSFEKHGEADLFDALAAPMAGRAL

AEVLGFLDVDWQTMAFWSQSLIDGAGNYSGDPEISHKAVEAAKGVEEAINRALPYHRQNPNTSVLSSMIYAED
PHTIEQIYANIKVAVGGGLNEPRDSILTMVLGLLENPEQLARVKADPNLWPTAFEESVRWISPIGMYPRRVTR
DVVVSGVRLREGDQVGLCVSAANRDDSRFVDPDRFDVFREKKSHLAFGAGAHFCAGTWVARHMVGRLVVPRLF
ERLQNLHLKNTAAARIHGWVFRGPVSLPVVWDA

>CYP1078A5(IE4803_PC00147)*Rhizobium etli* bv. phaseoli IE4803

MLTEETAATPPVISDVTFAELLADPYPTFKRARDMAAVVRIEAANMLVATRYEDIMAMERDPELFSSENPQSL
VNKVMGHTMMRKDGEAHARERKAVEPALRPGAMKSCWGPRFETIFDEIVSSFEKHGEADLFDALAAPMAGRAL
AEVLGFLDVDWQTMAFWSQSLIDGAGNYSGDAEISHKAVEAAKGVEEAINRALPYHRQNPNTSVLSSMIYAED
PHTIEQIYANIKVAVGGGLNEPRDSILTMVLGLLENPDQLARIKADPNLWPTAFEESVRWISPIGMYPRRVTR
DVVVSGVRLREGDQVGLCVSAANRDDSRFVDPDRFDVFREKKSHLAFGAGAHFCAGTWVARHMVGRLVVPRLF
ERLQNLRLENTAAARIHGWVFRGPVSLPVVWDA

>CYP1078A6(A6B35_25075)*Mesorhizobium amorphae*

MSEAVKVVDDVTFEELLANPYPVFKRVRDLGSVVKVDAANIMMVTRFDDIMAIERDPETFSSINPQSLLNTVI
GPNLMRKDGEDHARERAAMDPSLRPGVVKRCWAEPMQVICDDVISQIEHKGEAELFEELAAPIAARALADILG
FDGVDWQTMALWSQSLIDGSGNYSHDPEIARKAREAASGVESAVDRMLPHHRANPNPSMLSSMLQADPPQTVE
QIYANIKVAIGGGLNETRDSILTLILGLLQNPDQLARVRAEPSLWGQAFEEAVRWISPIGMYPRRLTRDTEIS
GTLVHKGEQVGLCVGAANHDDRRFSDPDKFDVFREKKSHLAFGAGSHFCAGTWVSRALVSQIVVPMVFERLRN
LRLKDPELVRVRGWVFRGPTHMPVVWDA

>CYP1078C1(SL003B_3164)*Polymorphum gilvum*

MTAPNRRINAETVDFDQLWRDPYPTFARLRATNPVAFVPQADHYFITRFDDIAHIERHPAIFRASEPRSLVNR
VMGHSFMRKDDEEHMVERKIVAPSFSPAAAKKVWSDTFREIAADLLDDLQDRGSADLVADFATPMAARSLCTV
TGLTNIAWQDIARWSQALMDAVQNYARDPELARLGQEASDGIDAAIDAMIPVRRANPDRSILSMMLAGDMPLD
SIRANLKVIIGGGQNEPRDANSGLIWALLSNPDQLSAVKADPSLWLRGFEEYVRWVSPIGMYPRTVGQETELA
GVPLKPGDRLFLLVGSANRDEAQFRDPDRFDLHREESRHLAFGSGPHFCAGAWVARAQVADIAVPMLFERLKN
LELDDSGEPARCGGWAFRGLLTLPARWSA

>CYP1081B2(AZL_b06160)*Azospirillum* sp. B510

MAESAEQGPYVDANAIFRRVPAPPHIPADRMIDFDYRHPAGLEEGDVYTALKRLQDGPEIAWTPHNGGHWIVT
RGEDIKWVQETYEIFSHEEFTIPRGAAPIKMPPLTTDPPDHGRFRSVLNPAFTPVQVNKMRDKARTLAIDLIE
SLRPEGTCEFVGDFARVMPVTVFLGIVDLPLDRREEFVEWALTFMVADDMADRLAAQGKIVAYLRTVLDERER
NPGDDLLSRIVAWRKDPRFGGEHEVMGMALLVFFGGLDTVASMLSFVAHHLASHPGHVRRLREEPGIIPRAAT
EYLRRFGLSNTGRLIKKDTTYKGVTFLKDEMIMVPIGLSSIDDRLYDDPFTVNFDREVSVLNTFGNGPHRCAG
APLARVELEIFLAEWTSRMPDIRLDPDRPAITHAGVVNGVERLHLLWDV

>CYP1081B3(BSY18_3670)*Blastomonas* sp. RAC04

MANVLTPKPDHVPDHLVVDHDYVDLAGMAELGVYAASKRLHDGPDIIWTQRHGGHWLVTRAEDVKFVQENYAI
FSHEEFMIPRLLNPFKVVPLSVDPPNHARYRAVLNPAFAPSKIAKMRGDAKVLTIELIEQIRPKGRCEFVSEF
ARIMPVAMFLRIVDLPLDRREEFVEWGLAIMSSYEPDERMKANMRVREYLKTVLDEREGGEGEDLLTRVANWR
RNPRFESDEETLSMATLLFVGGLDTVASSLSYITHYLAQHPEQQARLRKDKDIIPKAAEEFLRRFGLSNTGRI
LTRDFEYKGVLFKKDDMIMVPNNLSGIDDRAYPNPLVVDFDRGVGPMDHNTFGNGPHKCIGAGLARAEIQVFL
DEFVGRMPEFRLDPDQVNVEHCGSVPGFDELHLRWD

>CYP1082A2(RG1141_PB00370)*Neorhizobium galegae* bv. officinalis bv. officinalis HAMBI 1141

MTIDVNEINLADHSFWRRPDRDEGFAILRRKRPVSWHEFPEGPEQGMQGFWSVTRYDDIVAVSQDAKTFRNSP
TTYIGDQSVEEARQEGWFLNMDAPEHFKLRQVVSKIFSPTGVQTLARTAAQHAEGLVLAAKEKAACDFATEVA
QPFPVAVICDILGAPASDRKHLHRLTVTALAGDAPEMGGADKVPAAFAELNVYGAELSRERRKNPKDDVVSLF
IATEIDGRKFNDDEVGYFFQLLVTAGMETTGTVGGHMMRLFLENPDQMAIWAADPDAIAQTGVEELVRMVSPV

QHMRRTAAANIEIGGQQIAEGDKVVVWYNSGNKDDAKFENPFSFDVKRNPNPHIGFGGGGRHTCLGAHLARLE
LPMLAKASLQHLKNIEPAGDAVFVPSRFVNGLASLPIRYTA

>CYP1083A2(TZ53_18235)*Sphingobium* sp. YBL2

MMFPEQPIPDHVPPELVSRDFPFIFGMTTTEDPFDDLAMQVHEGPDIIYATHAYPGGTPAWVPRRMAHLREIY
LDTDHFSSTDFSPFSKLVGGNWINSPAELDPPAHGPFRQMVNPAFTPKAMAALEDKIRLYAVEYIDAFAPRGQ
CEFMGDFAFEFPIKVFMELMGLPHDRMSEFMEWEMNLLHNHDLSKIAEATRSVLAYLGGEIEDRRANPRDDLI
SFGVKGQINGRALTDDELLGFMFNLFIGGLDTVSTNMGLHFRHLGSHPQDQAFLRANPSEIPHAIDELMRAYG
AVTTFRTCKKETEVAGIRFMPGDKVAMSTTLAGRDPAEFDAPAEVRLDRRPRHVGFGFGIHTCVGMHLARREL
RIAMEEFLARIPDFRIAPGHQVECHLGMIQPVALPLVWTPAS

>CYP1083A3(TQ29_17635)*Confluentimicrobium* sp. EMB200-NS6

MELDLSPKEVEIPAHVPDELVLPFPFLFGVKTKQDPFGELAAQVHAETPPIFYSKHAYPGGTPAWVVRTAEDL
KTIHFDTENFSNKGFSPFSMIIGETWNLVPAESDPPIHAHYRAFVSRVFSPGAIAKLEDKIRGYAREYVEGLK
DKGKCEFMRDFAFEFPIKVFMELMGLPQSRTKQFLEWEEAMLHSHDLKKMAVATRSVVDYLREELADRRENPT
DDLFTYGVQAKIDGRELTDDELVGFTFNLFIGGLDTVSTQMGLQFLHLARNPEHQKMLRDDPSLIPAAIDELM
RAYPAVTVFKTVTQPCELKGVKFMPGDKVVMSATLAGRDPAEYDAANEIRFDRESDGHLSFGYGPHLCLGMHL
ARREMRIAMEEFLRAVPEFRLKDGVEVEHYLGMIQPTAVDLVW

>CYP1083A4(BSY18_2514)*Blastomonas* sp. RAC04

MLGVQVTEAPPHVPADLVRDYPIKMGGLVEENPFDRMIPEIHRTMPPIFYALDAYPGGTPAWIVRRVKDLQAI
YYDTESFSNKDFAPFALLVGESWSSLPAETDPPMHGLYRKWVNPLFSPKEMNKLDRKIRDYARDYCTAIKARG
SCEFMGEFAFEFPIKVFLELMGLPQEMTAEFLEWEMGLLHTNDLAVIAASTRKVVDYLRGEIADRKANPRDDL
ISYGVTMPIDGRPLTDDELVGFTFNLFIGGMDTVSTNMAWQFRHLATHPEDQAMLRAKPAMIPDAIEEMMRAY
PAVTTFRTCSREVTIGGVTFQPGDKIAMPTSLAGRDPEEYPDADKVILGRKSRYLSFGYGPHLCVGMHLARRE
MRIALEEFLAAIPPFRIQDGCTVKTHTGGILQPETLPLAWD

>CYP1083C2(BSY18_2436)*Blastomonas* sp. RAC04

MTTTLPPPATLPTHVPADKAMRLPFFAREVVRDCPQETLIPQMHSSLGPITYVTNIFPGDKPGWLLTGYEDTM
AMLRNADDFTKNGMGQWAQGIGEDWLVIPTEVDPPMHTAYRKALNPHFSPQKMAAMKDDLRKRARDLIDKFQD
RGSCDFVNEFSERYPIYIVLDLLGLPQDRMAEFLKWEKEMLHTNDWEVRSNAVRAVKNYLVEEIAARRANPQD
DYITRIFDFEAEDGRKWNDQEVLGHCFNLYLGGLDTVTTMLGNSFAWLARHPEQQQELRENPDRIVLAVEEFL
RIFGPVTAFRIATREIEIHGQKIMPGEYVSVCTPVANQDPKIHDHPSQVRFDRKAAHIALGGGIHKCLGMHLA
RLELQVALEEMLKAVPLFRVRDGFDLGYFVGNITFVPALELQWD

>CYP1083C3(TZ53_18635)*Sphingobium* sp. YBL2

MMTQVAQQSLPDHVPPELAMALPLFSRTVIYDNPQEVLIPAMHAELPPITYVTNIFPGDQPGWLLKNAEDVQA
MLRDADNFTKNGMGKWAQNIGENWLVIPTEADPPIHTGYRKALNSHFAPQKMFAMKEQVRERAQALIDAFKDR
GQCDFIDEFSEKFPIFIVLDLLGLPQERMTQFLKWEKEMLHSNDWQVRGNAVRCVKDYLLEEIEARRQQPRDD
YISKVLTFEVDGRLWNDDEVFGHCFNLYIGGLDTVTSLLGNIFNYLASHPDKQNELRADPSLIVLAVEEFLRA
FAPVTAFRIATKVIEIHGQKIMPGDYVAFSSPVVGRDPAFYEDPQAIRFDRKAPHMSLGSGIHKCLGMHLARL
ELQIAVEEFVKAIPEFRIKDGFRVPYYVGNILHVPDLHLQWG

>CYP1086B1(K663_17251)*Sphingobium* sp. MI1205

MSANDIASQGAIASFNPFDPDHLSEPAAGWALMRREAPIMSLNLPGPVPVFVVSRRKEIEAIARDTTLFSSKP
VPSVWRWGDFEPAIADIFATSGYKIVYTLQTTDPPTSLQYRRIAEAALTRKRVLDLEPEINSIIGKLMDAIPS
GGRINFVDAFSVPLTLELICMILGLPYSDATFIKHFSDEFTFLVDPTHSTERAMEATRTVVEGYRYLADYMRR
FEEAPAENLLSAIAHADIDGRPLTIEEKLSMAHVLTIAGNETTRNALSSAMYMLAIRPDLWAALKAAPERVPD
YVEEILRVHAPAVTTPRTATADTEIDGVAIPKGSALFLLWASAGHDETLFDNPYEIDLDRKNKRSHITFGMGI
HHCVGSFLARAELNAAVLRWITDFDTVELAVPQSEIRYDPVFAFHALSDLPIKVTRVKEKQVVRSEE

>CYP1088B5(LH19_02885)*Sphingopyxis macrogoltabida* 203

MTAAQLAAAPVPPHIPAHLVFDFDIYADPRIGEDVQGTYAAALEDAPGIFWTRLNGGHWIVKSFDAISQIVLD
PEHFSVREMQIPRVQNPPFMIPLSLDPPENLPYRRAMMPMFGPAAIKALEPRIREWAAEFVEAVADKGACDFQ
ADVSKVFPVSVFMELMGMDLARLQDFRHLAEDFFSAQNDEAELGRLSALILGELKGLIAEKRAVPDGKLMTHF
ITVEVDGRTMSDDEILAMSFVLFLGGMDTVTNVTGFAFQQLAQMPEVQARLAADPSLIPAFADEAVRLYGVVN
TPRLVVQDQQIGEARFREGEMVLNVLCLGSRDPAKFEQPNIFDLDRKKTAHLTFSSGPHLCVGHVLGRAELRI
LTEEWVKRVPAFRPVPGEKHGFRIGTVMALESLPVEWEVAG

>CYP1088B6(WG74_06155)*Citromicrobium* sp. JL477

MNAPSKLAPVIPDHVPTDLVFDFDIYADPRIDSDVQGSYQRAINGAPDIFWTPANGGHWMVQRMDSIAAITTD
PEHFSVREMQIPRVENPPFMIPLSLDPPENLPYRRAMSPMFGPKAIKALEPRLRYWAEKIVDSVADKGECDFM
AEVAKIYPVSIFMELMGMDLARLPEFRDLAERFFDAQNNSALLDQLSAQILGILSELIEEKRKNPDEQLMSHF
VHVDMGEGKPMSDNEILAMSFVLFLGGMDTVTNVTGFAFQQLAQMPDLQKPMAADPAGLSTAFADEAVRAFGV
INTPRLVVKDIDLLGVPFRTGDMVLNVLPVGSRDDRKFEHPNLLDIDRKRAAHITFSTGPHLCIGHVLGRAEI
RVMAEEWFKRIPSFELVPDSERTFRTGTVMALENLPLRWDS

>CYP1089A2(BSY17_3856)*Sphingobium* sp. RAC03

MDHALSPAAPKPAHVPDALVYDFDIHADPGLLDDPHVRIMDLLRNAPPVFWTPRNGGGWMILTHAANYEASRD
TEAFSSEFVPQDKMKAMLASLPPGAPHIPQPIPITLDPPEHTKYRQPLQKVFSPKTISALRDSIRELAGDLIE
AIKPDGRCEFMSAVAEPLPVQVFLKMLGLPLDRLPEYRAIVKEHMEAIDGDREGSMRRLQTIAAAMRDTLLDR
RDNPRDDIISMLWKLEIDGQPTTLADMENYGVLLFIAGLDTVMNGMGLAMRGLAVDTDLQRKLRAEPKLIAEA
AEEMLRRYTFTVPMRVVKRPVELSGAKMMPGDMVKLFLPAADLDAKEFPQPDVYDLDRENNVHIAFGVGPHRC
LGSHLARVELQVLYEEMLARMPEFRLDPDHPPVFHGGHVIGIESMHLVWDV

>CYP1090C1(Ga0102493_111098)*Erythrobacter litoralis* DSM 8509

MATQAEGSGPQVNLFDPEVQQCPWDAYRTLREEAPVYRIPGTDIFVVSRYEHVREVLMDPQRFPSSAKNELMR
ASPTDVERGRKVAERFKAKGWLPAPTLAGRDDPNHKQMRAMFNEAFKPSRIKEIDPRVENLAYELIDGFLDEG
ECDWVSRFCIPLPLFIIGEQMGAKREDMWRIKGWTDAFFHRISMMLPEDRHLEMVDREIEAQHYFQPIFEKLR
EQPDESLISVLVNTVIEGWGRPLNDNELHAEMMADTFVGGSETTTNALAAGMKLLIENKDVWAKLKSDPDRYM
RTFVEEVLRLESPVQSLMRFAHEDVELAGVTIPAGSIINVRYGAANRDESAFECPEKLDLDRPRAGAHMAFGS
GTHHCLGAPLARRELTWGFTAVVDRFEDMDFAEGKNDFTYHPHFLLRSLKQLHITFEPKKR

>CYP1090C2(BG023_11286)*Porphyrobacter* sp. LM 6

MNSPTINLFDPQLQQCPYEAYKTLRDEAPVYNIPGTQIYVVSRYDHVREVLMDPARFPSTAANELMRASPTDM
ERGRKVAERFKEKGWLPAPTLAGRDDPNHKQMRAMFNEAFKPSRIKEIDPRVETLAYELIDEFLDDGHCDWVR
QFCVPLPLFIIGEQMGAKREDMWKIKGWTDAFFHRISLMLPEDKHLEMVDREIEAQHYFQPIFERLREHPDGS
LISVLVNTVIEGWGRPLNDNELHAEMMADTFVGGSETTTNALAAGMKLLIENKDVWAKLKADPDKYLRNFVEE
VLRLESPVQSLMRFTHKDVELDGVTIPAGSVVNVRYGAANRDERFFECPEKLDLDRPKAGAHMAFGSGTHHCL
GAPLARRELTWGFQAVVDRFEDMEFAEGKNDFTYHPHFLLRSLKQLHITFEPKRR

>CYP1091A2(Sala_0903)*Sphingopyxis alaskensis*

MTSLAERCPVARAGADAPPAPAHVPPDLVLDTRFAAGIVPNDLAEPYRPMDVVRDEAFPRIHYYPWPISGNRH
GAWVVTRYEDIRRVYEDNELFSSEGVAQFQALAGETFPSIPLGIDPPDHGRYRKFLNPWFTPVAMNAREPRIR
EIIGGMIDQFADRGEVDIAYDFGRVFPVRVFLDLMGFPFTMFDQFLDWEFAILHDDRIDKKVEALTAVLAYLR
GFIADKQASPDANLGSYIANGSIDGVPLTDDEKIGITWFLWLGGLDTVASTVSQMFRRFAMHPQLQKRIREDK
SLINSAVEEFLRTQPLVNSGRKVKRDFEWHGVQIKAGDWVTAFNTSGNFDPQQFECPHQFDPERRNNRHFTLA
GGVHICLGAHLARRELRVLLDEWFTRIPYEFRVREGADTTVVPSLLSIRNLPIVWDCKAA

>CYP1091A3(LH20_13075)*Sphingopyxis* sp. 113P3

MTSLAERCPVARAGADAPPAPAHVPPELVLDTRFAAGIVPNDLIEPYRPVDVVRGDGFPRIHYYPWPISGNRH
GAWVVTRYEDIRRVYEDNDLFSTEGVAQFQLLAGETFPSIPLGIDPPDHGKYRKFLNPWFTPVAMNEREPRIR
EIIGSMIDQFATRGEVDIAYDFGRVFPVRVFLDLMGFPFTMFEQFLDWEWNILHEEDLEKKANALRGVLAYLR

GFIAEKQAAPDDALGSYIANGSVDGRKLTDDEIIGMTWFLWLGGLDTVASTVSQMFRRLAMHPELQAEIRTNK
GLINSAVEEFLRTQPLVNSGRKVKRDFEWHGVEIKAGDWVTVFNTSGNFDEQQFECPHQFNPARKNNRHFTLA
GGVHICLGAHLARRELRVLLDEWFDRIPQPFRVKQGADTTVVPSLLSIRNLPIVWDTGAAS

>CYP1091A4(LH19_08465)*Sphingopyxis macrogoltabida* 203

MTETTLSRCPIAHAPADAPPVPAHVPPELVLDTRFAQGQIAYDLPDPYLPSDVMRDPEVPRILYYPWPTSGRQ
HGAWTVSHYEDIRRVYEDNDIFSTEGAAQFQALAGETFPSIPLGIDPPDHGRYRKFLNPWFTPVAMNDMEPKI
RAIVTEMIDAVVKDGEVDIAYDFGRIFPVRVFLNLMGFPFSMFDQFLEWEYAILHNPDIAAKAEAVKGILAYL
RGFIAEKQANPDETLGSYIANGQMDGQPLTADETIGMTWFLWLGGLDTVASTISQMFRRMALDTELQARIRAN
PEIINSAVEEFLRVQPLVNSGRRIKKDFTWHGVDLKEGDWIACINSSGNFDEKQFACPRDFDPERKNNRHFTL
IGGVHICLGAHLARRELRVLLAEWLSRVPPFRLKPGADTTVIPGLLSIRNLPIVWDTKAVS

>CYP1094A3(Ga0102493_111086)*Erythrobacter litoralis* DSM 8509

MNKPEGNVFAPQTLVDPFDYYRAVHDAGIAIEHLPEMNTFVVYSYDLCSEANTKPDIFSNDFSTLMGREADEE
IKEILKEGWDDVPTLLTADAPVHTRNRKLVNLAFSAPRVNAIEEDMRAKSIELIEAFADAGECEFVEQFAVPL
PVAMIAGQIGLDDNPKKVKEWSDAAVDRFSQMVDHERKKECARSLVEFQHYMKGKIDDRRENGGDDLLTDLVE
ARVEGETPLTDAEIMSIMQQFMVAGNETTTSTLAGGLLQLIRNPDQMEKAKAAAGGRDPKVITNLVEEALRYE
TPTAGMWRVVKQDTELGGVKMPAGSVAQLRYAAANRDPAKFEDPDRFDIERKNARAHLAFGKGPHMCVGNMLS
RKEMLVAFDELLERLDDFAVADEDGITILPNILLRGVTRLPITFTNTGTQKA

>CYP1094A4(BG023_11299)*Porphyrobacter* sp. LM 6

MNKPEGNVFAPETLIDPFDYYRAVHEAGIAIEHLEGMNTWVVYSYDLCSEAASKPEIFSNDFTALMGREADEE
IQAILAEGWPDLPTLLTADHPVHTRNRKLVNLAFSAPRVNAIEADMRQKSIDLIEAFADRGECEFVEEFGVPL
PVAMIAGQIGLDDDPKRVKRWSDAAVDRFSQMVDFERKKECARSLVEFQHYMKGLIDDRRANGGNDLLTDLVE
ARVEGETPLSDPEIMSLMQQFMVAGNETTTSTLAGGLLQLIRNPDQMAKAKAAAGGRDPKVIMNLVEEALRYE
TPTAGMWRIVKADTELGGMKIPNGAVVQLRYAAANRDPAKFPDPDKFDIERANARTHLSFGKGPHMCVGNMLS
RKEMLVAFDELLERLDNFAVADENEIRILPNILLRGVTHLPITFTRKG

>CYP1094A5(A9D12_03595)*Porphyrobacter neustonensis*

MNKPEGNVFAPETLVDPFDYYRAVHDAGIRIEHLEEMNTWVVYAYDLCSEAAARPEIFSNDFTALMGREADAD
IQAILAEGWPDLATLLTADAPVHTRNRKLVNLAFSAPRVNAIEAEMRAKSIALIEGFADRGSCEFVEEFGVPL
PVAMIAGQIGLDDDPGRVKRWSDAAVDRFSQMVDHERKKECARSLVEFQHYIKGLIDDRRAHGGNDLLTDLVE
ARVEGETPLEDAEIMSLMQQFMVAGNETTTSTLAGGLLQLIRNPDQMAKARAATGGRDPKVIVNLVEEALRYE
TPTAGMWRIVKQDTELGGMAIPAGSVVQLRYAAANRDPSKFPDPDRFDIERANARTHLAFGKGPHMCVGNMLS
RKEMLVAFDELLERLDDIAVTDDSAIRILPNILLRGVTHLPITFTRKA

>CYP1094B2(BSY18_2736)*Blastomonas* sp. RAC04

MTVAAEQKSFFHPDVLSDPFAFYQAMIPSQPVYHDAASGLYLVLSHKLVSEATARTDEFSNNFQAVLSGARSE
DPQVKAILEDGWPQVDTLLTADPPVHTRFRKLVNLAFSAPRVNKLEAHIREIAHDLIDIMLAKDDIDFVRDFA
IPLPIRMISEQLGLTHEDAGTIKRWTDAFVDRLGGMVPLERELQCAREVVEFQHAIKAQMDLRRATPTDDLLS
DLVHAQVDGERPLDDGELLSIVQQLLVAGNETTTATLAEGIILLARNPDELAKAKADPKIIPNMIEEMLRLAS
ASSGIWRVMKQDAELGGVTLPRGAMVMMRYAAANRDPEKYADPDAFIADRANARTHLAFGRGIHMCVGNMLSR
KEMTVAFEELLPRISAITLTDEAAIAYHPNMMLRGPSSVPVRFGLAS

>CYP1098A2(Swit_4559)*Sphingomonas wittichii*

MADAPEGEGPFTPPYPVPHRSKASLFKRFLTGWNSWIHTLFERSYTMQMGEIRLPRLDFFIANDLSVVERVMD
DREKVFPKHRFQHELLDPLIGNSVFSANGKDWEEQREMVNPAFAHTNLKRVFPAMQGAVDDLLATIATMDRSK
PIAVDPLMTHVAADIIYRTIFSVKLDQASADRIYHAFHRYQKNIQPGAMLKLYGLPLFGYRRRAARAAAEIHA
VFGPIVKARYDAWHSGGGEAGNDILQSLLEARHPASGEPFSYQAIMEQVSTIFLAGHETAASAMTWALYLLAE
CPHWQDALLAEIDRETKGEPIAFEHLRALGGVRNLFRETLRLYPPVSFLVREVTAPTTMRDKLLHKGAMIVIS
PWLIQRNRNNWKCPHAFAPERFDDPEEAEACRRAYMPFGKGPRICIGAGFAQQEGMLILASIVRAFRLRYPDI
PRPEPVSRLTLRPKSAVQLWFEDRR

>CYP1098A3(BSY17_1544)*Sphingobium* sp. RAC03

MTDLFTPPYPQPPRTKRGLIKRFLRGWHSWIHVLFDKSYTMKMGEIRLPGRTMYIANELSLVDQILRGGTAFP
KHSELVRNLDPLIGNSVFSANGTDWESQRAMVNPAFAHTALGRTMPLMVAAADDLVARLEAADRTAPVDIDPM
MTHVAADIIFRTLFSQALDAARSNIIHIAFGKFQRLAHSASMLRLYGIPAGWFETRSKGPARAIHDVFRPIVE
ARYEGWHARGEAPHRDILQSLIEAKHPDTGEAFTCNQVMEQVSTIFLAGHETSASTMTWALYMLAECRHIQDR
VRAEISAVAGDAPLTAAMLKEMGQLRNIFRETLRLYPPVSFLPREVTCPMDMRDKHLETGSMLVVAPWLTQRN
KDNWACPHAFDPDRFDDPANADMVKQAWFPFGRGPRVCVGAGFAQQEVMTVIAAVVWRFALSVPPGFKPEPIS
RLTIRPRTGMPLLFGDLGD

>CYP1098A4(SIDU_16355)*Sphingobium indicum*

MTQPFTPPYPEPPRTRRGLVKRFFRGWHSWIHVLFEKSYTMKLGEIRMPGQTMYIANELPLVDQILRGGTAYP
KHRELVRNLSPLIGNSVFSANGEDWESQRAMVNPAFAHTALNRSMPLMAAAADDLLARLDAADRSKPVDIDPM
MTHVAADIIFRTLFSEALDERRSNIIHTAFGKFQRLAHSASMLRLYGFPARWFEKRSLGPAKAIHDVFRPIVE
ARHEGFHQRGEAPHEDILQSLIEAKHPETGDPFTLQQVMDQVSTIFLAGHETSASTMTWALYMLAECSHIQNA
ARAEVTEIAGNAPFAATMLKDMGAIRNIFRETLRLYPPVAFFPREVTCPMPMRDKQLEEGAMLVVAPWLTQRN
EDNWACPHSFDPARFDDPANAEMVRQAWFPFGRGPRVCIGAGFAQQEVMTVIAAVLRRYRLSVPEGFKPEPIS
RLTIRPRTGMKLIFDAVPVDRRTA

>CYP1098A4(SJA_C1-33850)*Sphingobium japonicum*

MTDLFTPPYPEPPRTKRGLMKRFLRGWHSWIHVLFEKSYTMKLGEIRMPGQTMYIANELPLVDRILRGGTAYP
KHRELVRNLSPLIGNSVFSANGEDWESQRAMVNPAFAHTALNRSMPLMVAAADDLLARLDAADRSKPVDIDPM
MTHVAADIIFRTLFSEALDERRSNIIHTAFGKFQRLAHSASMLRLYGFPARWFEKRSLGPAKAIHDVFRPIVE
ARHEGFHQRGEAPHEDILQSLIEAKHPETGEPFTLQQVMDQVSTIFLAGHETSASTMTWALYMLAECSHIQNA
ARAEVSEIAEDAPFAATMLKDMGAIRNIFRETLRLYPPVAFFPREVTCPMPMRDKQLEEGAMLVVAPWLTQRN
EDNWACPHSFDPARFDDPANAEMVRQAWFPFGRGPRVCIGAGFAQQEVMTVIAAVLRRYRLSVPEGFKPEPIS
RLTIRPRTGMKLMFDAVPPSVDRRTA

>CYP1098A4(TZ53_03240)*Sphingobium* sp. YBL2

MTQPFTPPYPEPPRTRRGLVKRFFRGWHSWIHVLFEKSYTMKLGEIRMPGQTMYIANELPLVDQILRGGTAYP
KHRELVRNLSPLIGNSVFSANGEDWESQRAMVNPAFAHTALNRSMPLMVAAADDLLARLDAADKGKPVDIDPM
MTHVAADIIFRTLFSEPLDERRSNIIHTAFGRFQRLAHSASMLRLYGFPARWFEKRSLAPAKAIHEVFRPIVE
ARHEGFHQRGEAPHKDILQSLIEAKHPETGDPFTLQQVMDQVSTVFLAGHETSASTMTWALYMLAECAHIQNA
ARTEVQQLAGDAPLTAAMLKDMGAIRNIFRETLRLYPPVAFFPREVTCPMPMRDKSLEEGAMLVVAPWLTQRN
KDNWACPHSFMPERFEDPANAEMVRQAWFPFGRGPRVCVGAGFAQQEVMTVIASVLRRYRLSVPDGFRPEPIS
RLTIRPRTGMKLMFDAVRL

>CYP1098A5(ATN00_04210)*Sphingobium baderi*

MTDPFIPPYPEPPRSKRGLVKRFLRGWHSWIHVLFEKSYTMKMGEIRMPGQTMYIANELPLVDRILRGGTAFP
KHRELVRNLSPLIGNSVFSANGEDWESQRAMVNPAFAHTALHRSMPLMVAAADDFLARIDAADRGRPIDIDPM
MTHVAADIIFRTLFSQTLDAHRSNIIHTAFGKFQRLAHSASMLRLYGLPARWFERRSLRPAKAIHDVFRPIVE
ARFEGFHQRGDAPHRDILQSLIEAKHPETGAPFTVQQVMDQVSTVFLAGHETSASTMTWALYMLAECPHIQNL
ARAEVQDVAGGQPLAAGMLKDMGAVRNIFRETLRLYPPVAFFPREVPCPMDMRDKHLEEGAMLVVAPWLTQRN
KDNWACPHAFDPERFDDPANAEMAKQAWFPFGRGPRVCVGAGFAQQEVMTVIASTLRRYRLSVPQGFRPEPIS
RLTIRPRTGMKLMFEDMKG

>CYP1101A5(SPO1898)*Ruegeria pomeroyi*

MIPPKPPARPDKVSLWRYLRLFRQDLLSAQPARLYRAWMAEFRTPFFRSYLVNQPDLVDTVLKGRPDDFPKSN
RISEGLRPLLGNSVFLTNGETWKRQRRIIDPAFEGGRLRDTFPAMWAASEAAVNRLAAQAGQETEIEEVTSHA
AADVIFRTLFSIPIEHEVAAQVFTRFRAYQRAQPLLNLAAFVPVPQWVPRFHSRETRRNAAVIRGLIRQLTDT
RMGEIRAGTAPDDLATKIMTTTDPQTGDRFDTDEMVDQVAIFFLAGHETSASALAWTLYLMALYPDWQEKLAE
EARTLDSGTDFSVMSKLRLSRDVFREALRLYPPVPMMVRETTCPERFRDRDVPKGAQIVLSPWHLHRHERLWE

RPDDFDPARWQTENGKTCQRNAYMPFSAGSRVCTGAGFAMVEGPLILSMLLLRFRFERIAGREPVPVAHLTVR
AADGIWLRVVER

>CYP1101A6(AKL17_1859)*Defluviimonas alba*

MTLPPKPPSRPDKVSLWRYLKLFRADILSAQPARLYRAWMAEFRTPFFRSYLCNDPQLIDIVLKDRPGDFPKS
NRIREGLAPLLGNSVFVTNGEVWKRQRRIIDPAFEGGRLRDTYPAMLEASLAARDRLMKNASQDVEIEQETSH
AAADVIFRTLFSIPIDHHVAAEVFTQFRDYQRTQPLLNLAAFLPVPKWMPRFHRRSTRETAKAIRALITGLTT
ARMQEIAAGTAPDDLATKIMTTADPVTGDRFGIEEMVDQVAIFFLAGHETSASALAWALYLLATHPDVQDKVA
AEVADLGETPDFSAVSRLKFTRDVFREALRLYPPVPMMVRENSCPETFRGRAVAPGAQVVISPWHVQRHERIW
TDPDAFDPERWQAEACRRAAREAWLPFSAGPRVCTGAGFAMIEGPLILAVLVRAFRFEPVAGRVPVPVAHLTV
RAKDGIWLRPVPRLPCSKYPRGPRGAESPPRLRRKAVLR

>CYP1101A7(IMCC12053_2062)*Celeribacter marinus*

MSDRAKFTPPKPAARPEKTSLRNYLRLFRKDILSAQPAKLYRAWMAEFKTPFFRSFLVNQPDLVKTVLNTRPM
EFPKSNRIAEGLRPLLGNSVFLTNGETWLRQRRIIDPAFEGGRIKDAFPAMLAAGHDMVARLGEMDGGFDVEP
HTSHAAADVIFRSLFSIPITDETARAVFHEFQAHQKAQPILNLAAFIPLPKWVPRFHSRETKRTARVIRGLIT
RLTRERMAQIAAGTAPDDLATKIMTTADPQTGARFDMDEMVDQVAIFFLAGHETSASALAWALWLMAANPDVA
DRVAAEGAALGDAPTFSDLSKLRYTKDVFRETLRLYPPVPMMVREAACPEHFRDRDVPKGSQIVLSPWHLQRH
ERFWEDADVFDPDRFKTDNGKQCLRDAFIPFSSGARVCPGAGFAMVEGPLILALVARAFHVSTLAQHTPVPVA
HLTVRAQNGIWISCTKRDASNAALV

>CYP1101A8(P73_1746)*Celeribacter indicus*
MTEAGPDAGMPPRPASRPEKTSLRTYLKLFRRDILSAQPDKLYGAWMAEYRTPFFRSYLVNQPALVKTVLNER
PMDFPKSARIAEGLRPLLGESVFLTNGETWLRQRRIIDPAFEGGRLKDTFPAMLAAGREMVARLGAEEGDFDV
EPHASHGAADVIFRTLFSIPITHHTARAVFDEFQSYQRAQPILNLAAFVPLPKWVPRFHRRAARRGARAIRRL
ITGLTAERMAAIRAGTAPDDLATKIMTTTDPVTGARFSTEEMVDQVAIFFLAGHETSATALGWALWLLAAYPE
VQERVAAEAATLPEVPEFSDLSKLRFTKDVFRETLRLYPPVPMMVREAACPVRFRARDVPKGAQIVLSPWHLG
RHRHLWERPDAFDPDRFATANGRQCLRDAFIPFSAGPRVCTGAGFAMVEGPLFLALILRAFRIETVEGRRPVP
VAHLTVRSRDGIWLRLAPRG

>CYP1101A9(RCAP_rcc03256)*Rhodobacter capsulatus*

MKDAAADRATEAPQCPFAHRRPVKPAPRPERVGPRDFFRLIRQDILAVQPARMFRAWMAEFHSPFFTSFLCND
PDLVDLILTRRPEDFPKSLRLFEGLTPLLGHSIFITNGPEWERQRRIIDPAFDGGRTKDVLPAMWDAALSGVE
RLKPLADGRPVDVEGQASHIALDVIFRTLFSIPVENATANAVFDAFRAHQASRPMVNTGTLFSLPRWLPRLRF
GATAKTAKQIRGFISALVEARAAEIAAGTAPDDLATRIMSTPDPEDGKCFSPGEMVDQVGIFLLAGHETGAAA
LAWSLYLMALYPEWQDKLAEEAETVIDVEKIYASSVPRLKLARAVFRESMRLYPPVPMYLRETVQEETFRGRK
VPKGALVVVSPWHLHRHERIWKDPDDFDPTRWDTPEGRESSRLAYVPFSAGPRVCPGAGFAMAEGPLLLAMML
KHFRVALVPGREPMPVAQLTVRGRDGIWLSLTPR

>CYP1101A10(TM1040_1455)*Ruegeria* sp. TM1040

MTLRPPKPPVRPDRVSLWRYLKLFRADILSAQPQRLYRAWMAEFRTPFFRSFLVNQPALLDVILKERPDDFPK
SNRVGEGLRPLLGNSVFLTNGETWKRQRRIIDPAFEGGRLKESFPAMRAAAEAGVARLRPHADGSELEIEAEA
SHIAADVIFRTLFSIPIEHEVAAEVFSRFRAYQQAQPILNLAAFVPVPRWMPRFYPKGTRQNARHIRRLIADL
TKARMAEIAAGTAPDDLATKIMTTLDPETGKGFGAEEMVDQVAIFFLAGHETSASALGWALYLLALYPEWQEK
LAAEVAEHGAEEFADLSKLRLTRDVFRETLRLYPPVPMMVREAVQTERFRDREVLRGSQMVLSPWHLHRHERL
WERPDEFDPGRWQSENGKACARNAYMPFSAGSRVCTGAGFAMVEGVLILAQILRHYRITPVEGRSPEPVAHLT
VRSRTGIWLRFSHR

>CYP1101A11(roselon_02748)*Roseibacterium elongatum*

MILPPKPPARPDRVSLRQYLKLFRQDILSAQPARLYRAWMAEFRTPFFRSYLVNQPSLVDEVLKARPMDFPKS
DRVGEGLRPLLGNSVFLTNGAAWLRQRRIIDPAFEGGRLRDTFPAMWAAGEAAVARMGTGEVEVEAEMSHAAA
DVIFRTLFSIPIENEVAAETFHQFRAYQRTQPILNAAAFVPLPRWMPRGFAAETKRTARAIRGLIETLTAERM
ALIQAGTAPDDLATKIMTTSDPETGECFTTEEMVDQVAIFFLAGHETSASALGWTLYLLALFPDWQDKVAAEA

EAVLAGGMDFAVMSRLKLARDVFREALRLYPPVPMMVRETVRPERFRDRDLPVGSQVVLSPWHLHRHERVWDN
PDGFDPGRWATENGKTCLREAWIPFSTGARVCTGAGFAMVEGPLFLAMLLRAFRFERIDGKDPVPVAYLTVRA
KDGIWLKLAPR

>CYP1101A12(PGA2_c13430)*Phaeobacter inhibens* 2.10

MVDLPQSDDHPNSAPVAPVASSAIIPPKPAARAARVSLWRYLRLFRADLLSAQPAKLYRAWMAEFRTPFFRSF
LVNQPDLVRTVLKDRPEVFPKSNRIGEGLRPLLGNSVFLTNGEVWKRQRRIIDPAFEGGRLRQIYPAIHAAAE
AAVTRLSDQVHQTPNAPIEIEAETSHAAADVIFRTLFSIPIEHHMAQEVFHRFRAYQQAQPILNLAAFVPLPR
WVPRFHRPATRRNAARIRGLIRDLTQQRMEAIASGHAPDDLATKIMTTQDPETGELFTTDEMVDQVAIFFLAG
HETSASALAWALYLVALAPEWQDKIAKEAAGLSLDQFAEAGRLRISRDVFREALRLYPPVPMMVREASCPQQF
RGRTLPQGAQVVLSPWHLHRHERLWENPDGFDPGRWQTENGKTCQREAYIPFSAGPRVCTGAGFAMIEGPLIL
SMILQRFRVEPVAGQEPIPVAHLTVRSQNGIWLRLIPR

>CYP1101A12(PGA1_c13490)*Phaeobacter inhibens* DSM 17395

MGDLPHVDDRPDSEAAASAGGPVIIPPKPAARAARVSLWRYLRLFRADLLSAQPAKLYRAWMAEFRTPFFRSF
LVNQPDLVRTVLKDRPEVFPKSNRIGEGLRPLLGNSVFLTNGEVWKRQRRIIDPAFEGGRLRQIYPAIHAAAE
AAVTRLSDQVHEIPDAPIEIEAETSHAAADVIFRTLFSIPIEHHMAQEVFHRFRAYQQAQPILNLAAFVPLPR
WVPRFHRPATRRNAARIRGLIRDLTQQRMEAIASGHAPDDLATKIMTTQDPETGELFTTDEMVDQVAIFFLAG
HETSASALAWALYLVALVPEWQEKIAKESAGLSLDQFAEAGRLRISRDVFREALRLYPPVPMMVREASCPQQF
RGRTLPQGAQVVLSPWHLHRHERLWENPDGFDPGRWQTENGKTCQREAYIPFSAGPRVCTGAGFAMIEGPLIL
SMILQRFRVEPVAEQEPVPVAHLTVRSKNGIWLQLIPR

>CYP1101A13(Ga0080559_TMP3540) *Salipiger profundus*

MTPPKPKSRPDRVSLRRYVKLFREDILSAQPARLYRAWMAEFRTPFFRSYLVNQPELVRTVLRERPLDFPKSS
RISEGLRPLLGDSVFLTNGAQWQRQRRIIDPAFEGGRLRETYPAMWEAAQAALARLRGQAGGVVEIEEHTSHA
AADIIFRTLFSIPIEDALAQKVFHEFRAYQRTQPILNLAAFVPLPRWMPRFFRSDTKAAAKRIRALITELTER
RMAEIAAGTAPDDLATKIMTTRDPETGETFSTEEMVDQVAIFFLAGHETSASALAWALYLVATHPEWQARLAD
EAQVLGDTPDFGDMAKLRLSRDVFREALRLYPPVPMMVRETTCPERFRDRDVPKGAQIVLSPWHQHRHARLWD
NPDGFDPARWSTENGRACLRDAYMPFSAGPRVCTGAGFAMVEGPLMLSLILRDLKLTALPDRVPEPVAFLTVR
ARDGINLRVDPR

>CYP1101A14(Ga0080574_TMP4053)*Pelagibaca abyssi*

MSLRRYVKLFRADILSAQPARLYRAWMAEFKTPFFRSYLINQPDLVKTVLKDRPLDFPKSGRISEGLRPLLGD
SVFLTNGALWQKQRRIIDPAFEGGRLRETYPAMWEAAQAALARLTARTGEVVEIEEQTSHAAADIIFRTLFSI
PIEDALAQKVFHEFRAYQRTQPILNLAAFLPLPRWMPRFFRADTKAAAKRIRGLITELTERRRAEIAAGRAPD
DLATKIMTTRDPETGERFSTEEMVDQVAIFFLAGHETSASALAWALYLVATHPEWQERLAEEAQALGDAPDFA
DISKLKLSRDVFREALRLYPPVPMMVRETTCPERFRDRDLPRGAQVVLSPWHQHRHERLWDNPDGFDPARWST
ENGRQCMREAYMPFSAGPRVCTGAGFAMVEGVLMLSLILRDLKLTALPDKVPEPVAFLTVRARDGIHLAVDRR

>CYP1101A15(Gal_02054)*Phaeobacter gallaeciensis* DSM 26640

MMTDLPHPKRPPDSVPVTPAEGTPVIPPKPASRAAKVSLWRYLRLFRADLLSAQPAKLYRAWMAEFRTPFFRS
FLVNQPDLVRTVLKDRPELFPKSNRIGEGLRPLLGNSVFLTNGEVWKRQRRIIDPAFEGGRLRQIYPAMYAAA
EAAVARLSTQLEQQPDRPIEIEAETSHAAADVIFRTLFSIPIDHETAQEVFHRFRAYQQGQPILNLAAFVPLP
RWLPRFHRPATRRNAARIRGLIKGLTEQRMAAIDAGHAPDDLATKVMTMQDPETGELFTTDEMVDQVAIFFLA
GHETSASALAWALYLVALAPDWQEKIAAEAAALSLDQFAAAGRLRISRDVFREALRLYPPVPMMVREASCPQQ
FRGRAVPKGAQVVLSPWHLHRHERLWENPDGFDPGRWQTENGKTCQREAYIPFSAGPRVCTGAGFAMIEGPLI
LSMILERFRVEPVADQQPIPVAHLTVRSKNGIWLQLIPR

>CYP1101A16(PhaeoP97_01348) *Phaeobacter porticola*

MDNAPISEPATAPIGAADTTSASQSGQQEVLPPKPTARQEKVSLWRYLRLFRADLLSAQPAKLYRAWMAEFRT
PFFRSYLVNQPELVDTVLKSRPDEFPKSNRVSEGLRPLLGNSVFVTNGATWKRQRRIIDPAFEGGRLKHSYPA

MHAAAQAAVARLTDRIAAAPDNSIEIEAETSHAAADVIFRTLFSIPIEHHLAQEVFHRFRAYQQGQPILNLAA
FVPLPRWLPRFHRRDTRRNAAHIRALITRLTEERMQAIAAGSAPRDLATKIMTTADPETGERFSTDEMADQVA
IFFLAGHETSASALAWALYLVALAPKWQDRIAEEAADLPLETFSAASKLRISRDVFREALRLYPPVPMMVREA
TCPQQFRGRTLPKGAQVVLSPWHLHRHTRLWDNPDGFDPGRWETDNGKTCQREAYIPFSAGPRVCTGAGFAMI
EGPLILSMILQRFRVEPVAGQEPIPVAHLTVRSKNGIWLHLAPRQREAQPDNDR

>CYP1101A17(Jann_2396)*Jannaschia* sp. CCS1

MNDLPPKPPARADHVSLWRHMKLFRQDILSAQPARLYHAWMAEFRTPFFRSYMINEPALIDEVLRARPMDFPK
SDRVGEGLRPLLGDSVFLTNGETWARQRRIIDPAFEGGRLRDTFPAMWAAGEASVARMVPGLHEIEEEMSHTA
ADVIFRTLFSMPIEADIAGRVFHQFRAYQRTQPILNAAAFVPLPRWLPRGHRAPTRETARNIRALITEMTRAR
TVEIEADIAPDDLATKIMTTPDPETGACFTTDEMVDQVAIFFLAGHETSASALAWALYLLARYPDWQDKVAAE
ASGLPETPEFSSMSRLKHTRDVFRETLRLYPPVPMMVRETTGPETFRNRAIRRGSQVVISPWHLHRQNRLWDN
PDGFDPGRWETENGKACLRDAFIPFSAGARVCTGAGFAMVEGVLLLAMLVRAFRFERITGDDPVPVAHLTVRA
KDGIRLRVTAR

>CYP1101A18(TQ29_08885)*Confluentimicrobium* sp. EMB200-NS6

MTTPPKPASRPERVGLLRYLRLFRRDILSAQPARLYRAKMAEFRTPFFRSYLINQPDLVDLVLKERPMDFPKS
GRVGEGLRPLLGDSVFLTNGADWQRQRRIIDPAFEGGRLRDTFPAMVAAGKAASARLSEGVTEIEAQTSHAAA
DVIFRTLFSIPIEHEVAAQVFTRFRDYQRSQPVLNLAAFVPLPGWMPRFHRSRTKASARAIRALITRLTADRA
AAIAAGTAPDDLATKIMTTADPETGRRFGTGEMVDQVAIFFLAGHETSASALAWALYLLALYPDAQERVAAEA
TALGENPAFSDISKLTFTRDVFREALRLYPPVPMMVRQATRGERFRNRDVAPGSQIVISPWHLHRHALLWDRP
DAFDPGRYGTEAGRACLRAAYIPFSTGPRVCPGAGFAMLEGPLLLALLLKDWRLEPVQGRAPVPVAHLTVRAK
DGIWLRLHRR

>CYP1101A19(METH_10465)*Leisingera methylohalidivorans*

MLPPKPPARPDRVSFWRYLKLFRQDILSAQPARLYRAWMAEFRTPFFRSFLVNQPDLVKTLLKERPDDFPKSD
RIAEGLKPLLGNSVFLTNGEAWKRQRRIIDPAFEGGRLKDTFPAMRAAADAAVARLKGRQGPVEIEEETSHAA
ADVIFRTLFSIPIEHEVAVQVFSRFRDYQRSQPLLNLAAFVPLPRWMPRFFRRGTRRNAEMIRGLIAQLTCER
MNAIRAGTAPDDLATKIMTTRDPETGSTFDSAEMVDQVAIFFLAGHETSASALAWALYLMALYPEWQEKVAVE
AEALEDESFAAVSKLKISRDVFRETLRLYPPVPMMVRQAACPEQFRNRNVPKGAQMVLSPWHLHRHERLWENP
DRFDPARWGTENGRQCQREAYIPFSAGARVCTGAGFAMVEGPLILSMILRQFRITPVAGRVPVPVAHLTVRSK
NGICIHLTER

>CYP1101A20(MALG_01463)*Marinovum algicola*

MKRPPKPPARADKVSLLRYMSLFRKDILSAQPARLYRAWMAEFRTPFFRSFLVNQPDLVRAVLKERADDFPKS
DRIGEGLRPLLGNSVFVTNGETWKRQRRIIDPAFEGGRLRHGFPAMRAAAEAAVQRTAAHSGETLEVEAETSH
AAADVIFRTLFSIPIEHEIASQVFAEFRHYQRSQPILNLAAFVPLPKWMPRFFRRDTRASAKTIRRLITRLTT
ARMAEIAAGTAPDDLATKIMTTPDPETGETFTTEEMVDQVAIFFLAGHETSASALAWTLYLMALYPEWQERLA
EEAQCLPELLSEANGSAQAAPDFGVMAKLRLSRDVFREALRLYPPVPMMVREAQCPESFRDREVPKGSQIVLS
PWHLHRHERLWERPDEFDPGRYATENGKTCLREAYLPFSAGARVCPGAGFAMVEGPLLLSYLLRHFRFALGAG
PAPVPVAHLTVRSDNGIWLRVAPRLSPDP

>CYP1101A21(RCA23_c13370)*Planktomarina temperate*

MSPPLLPPKPPARPGRVSLWRYVKLFRRDLLSAQPAKLYRAWMAEFKTPFFRSYLVNQPELVDLVLKRRPRDF
LKSNRVSEGLRPLLGNSVFLTNGAEWERQRRIIDPAFEGWRLREAYPAISAAAEAAVARLAPKAGGGPVEIEA
ETSFAAADVIFRTLFSIPIEDRMARQVFEEFKTYQRSQPVVNLAAFFSLPSWVPRPFSRRTRRSAEVIRRLIT
ELTDKRAEALAAGTAPNDLATKIMTTKDPITGQLFSASEMVDQVAIFFLAGHETSASALSWALYLMATHPDYQ
EKVAREGAALGPQARFEDLRALRVTRDVFRETLRLYPPVPMMVREAAQAERFRGRDVASGSQVVLSPWHLHRH
MRLWDNPDGFDPERWHSENGKHCQRAAYIPFSAGPRVCTGAGFAMLEGVLLLARLCAAYELTPIAEQVPQPVA
HLTVRAQSGIWLNLRPRGI

>CYP1101A22(OA238_c12850)*Octadecabacter arcticus*

MYAAGEAAVARMAANTGVQDIEPETSHAAADVIFRTLFSIPIEDETATAVFEKFRDHQRTQPILNLRAFIPGP
RWMPRFFSRATKSTARDIRRLITDLTERRMFEIDAGTAPDDLATKIMSTADPVTGQTFSTQEMVDQVAIFFLA
GHETSASALAWTLYLLAMHPEWQDRLAVEAAAVTPDFASVSKLRLSRDVFREGLRLYPPVPMMVRETKCREEM
RGRAAPKGAQIVLSPWHLHRHERLWDNPDGFDPTRWGTENGKQCQRVAYIPFSAGPRVCTGAGFAMVEGPLLL
SMLVARFRFELTDRVPVPVAHLTLRAKDGIWLKITERD

>CYP1101A23(BVG79_01360)*Ketogulonicigenium robustum*

MAEMRTPFFHSFMVNDPALTKVVLKERPDDFPKSDRIAAGLIPLLGRDSVFLTNGAQWKQQRRIIDPAFEGGR
LREIFPVIWESAAAAVARLGATPAGSVVEIDHHASHAAADVIFRTLFSIPIESDVAREVFEAFRDHQRSRPVM
NLGAFINLPAWVPRFPSRKTGETARRIRRLISKMVDERQAQIAAGTAPDDLATKIMTTADPETGALFTPAEMV
DQVAIFFLAGHETSASALAWALYLVALYPEWQDQAAREAQGMAAEFSALSKLRINRDIFRETLRLYPPVPMMV
REATAPETFRDRAVPVGAQIVLSPWHLHRHTRFWQSPDDFDPARWSTPECRASARDAYMPFSEGPRVCVGAGF
AMLEGTLILAMLLRDFRFEVVEGRDPVPVAYMTVRAKDGIWLRVTPRGPVNPPAQTTG

>CYP1101A24(EIO_1648)*Ketogulonicigenium vulgare* Y25

MKPPKPEAREGKVSIWRYLRLFRQDILSAQSGKLYRAWMAEMKTPFFHSFMINDPALTKTVLRERPDDFPKSG
RIAAGLIPLLGKDSVFLTNGAQWKQQRRIIDPAFEGGRLRHIFPVIWDAAESATHRLQEAVGDEATEIDMHAS
HAAADVIFRTLFSIPIESDTAREVFDAFREHQRSRPVANLGAFVKLPAWVPQFPSFHTRRTARQIRRLITAIV
EERRAQIAAGTAPDDLATKIMTTPDPETGTLFTPAEMVDQVAIFFLAGHETSASALAWSLYLVALYPEWQEQA
ASEAAGMAPAFSALSKLRINRDIFRETLRLYPPVPMMVREATQREDFRGRTVPEGAQIVLSPWHLHRHERFWD
TPDDFDPGRWSRPEARLSARDAYMPFSEGPRVCVGAGFAMLEGTLILAMLLRDYRFELVAGRDPVPVAYMTVR
AKDGIWLKVTRRAAIAPADPPAQTDG

>CYP1101A24(KVU_1119)*Ketogulonicigenium vulgare* WSH-001

MKPPKPEAREGKVSIWRYLRLFRQDILSAQSGKLYRAWMAEMKTPFFHSFMINDPALTKTVLRERPDDFPKSG
RIAAGLIPLLGKDSVFLTNGAQWKQQRRIIDPAFEGGRLRHIFPVIWDAAESATHRLQEAVGDEATEIDMHAS
HAAADVIFRTLFSIPIESDTAREVFDAFREHQRSRPVANLGAFVKLPAWVPQFPSFHTRRTARQIRRLITAIV
EERRAQIAAGTAPDDLATKIMTTPDPETGTLFTPAEMVDQVAIFFLAGHETSASALAWSLYLVALYPEWQEQA
ASEAAGMAPAFSALSKLRINRDIFRETLRLYPPVPMMVREATQREDFRGRTVPEGAQIVLSPWHLHRHERFWD
TPDDFDPGRWSRPEARLSARDAYMPFSEGPRVCVGAGFAMLEGTLILAMLLRDYRFELVAGRDPVPVAYMTVR
AKDGIWLKVTRRAAIAPADPPAQTDG

>CYP1101A25(Dshi_2337)*Dinoroseobacter shibae*

MRPPAPKPVARQDRVSLWQYLKLFRKDILSAQPARLYRAWMAEFRTPFFRSYLVNDPVLVREVLHDAPEDFPK
SDRIAEGLRPLLGESVFLTNGATWARQRRIIDPAFERGRVRESFAAMQDAARNAVARLEARRAADLVEIEEVC
AHAAADVIFRTLFSVPIDDAMAAVVYRAFRAHQRSQPILNLGAFLPLPRWMPRMHRAETKETAAHLRALIAQM
TARRQAEIAAGTAPRDLATKIMTTPDPETGARFSTDEMVDQVAIFFLAGHETSASALAWALYLLGAAPDVQAR
VRAEVAAVAPLEMTFSDLSKLPRTRDVYREALRLYPPVPMMVRQVEAARVWRGRKIRRGAQVVLSPWHLQRHT
RLWSAPDVFDPDRWQREETQQSQRDAYMPFSAGRRVCPGAGFAMAEGTLMLAAFLQAFRFEADPNHVPVPEAH
LTVRARHGIWLRVGAAGQEF

>CYP1101A26(RCAP_rcc03254)*Rhodobacter capsulatus*

MTDETAAVPQGAILPPKPQARAGKVSLLEYLRLFQRDILSAQPAHLYRAWMAEMKTPFFRSFLCNEPELVHRV
LRGSPHDFPKSERIRFGLKPLLRDSVFITNGALWEHQRRIIDPAFEGGRLREVFPAMWDSGVAAVERLRAKAD
GQPLEIEAETSHVAMDVIFRTMFSMPIEHEIAQAAFEGFRAHQAAHPVANLAAILPLPKWVPAFHSKQTRDTA
RAIRALIGQLATMRAGEIENGTAPDDLATKIMTTPDPETGRVFETGEMVDQVAIFFLAGHETSAAALAWSLYL
LATHPDWQDKLAAEATEVLGDDVPVFSIINRLKLSRAVFREAMRLYPPVPMFVREAACPMTFRGRSVKKGAQI
IVSPWHLHRHERIWDNPDAFDPGRWETENGKAGLRDAYVPFSEGPRVCPGAAFAMAEGPLILSMFLRAFRFEP
VPGAVPMPVAHLTVRSDAGIRLKLTPRA

>CYP1101A27(P73_0363)*Celeribacter indicus*

MSAALASAAPLPPKPAARAGRVSLFGYLRLFRRDILSAQPGHLFRAWMAEMRTPFFRSFLCNDPELVHRVLRG
PVRDFPKSERVRIGLRPLLRSSVFISNGALWEHQRRIIDPAFEGGRLREVLPAMWQAGRAAVARLAPLADGRA
LEIEAQTSHVAMDVIFRALFSQPIESGIAQAALVAFRDHQAAHPVANAAAILPFPKWVPRCHSRAVRASAARI
RTLIVRLTTMRAAEIAAGTAPDDLATRIMTTPDPETGRVFDTAEMVDQVAIFFLAGHETSAAALAWALYLLAT
HPDWQERVAAEAGAVLGAGAEPDLATLKRLETSRAVFREALRLYPPVPMYVREAACPMRFRARRVPKGAQIVI
SPWHLHRHERIWENPDGFDPARWKTGNGREGLRLAYIPFSEGPRVCPGAGFAMAEGPLLLSMLVHVFRFDPVA
GDVPVPAARLTLRSADGIRLRLRPRLPTPSA

>CYP1101A28(Pden_3985)*Paracoccus denitrificans*

MIPPKPPARQGRGSVWRFIRDFRRDILSAQSDKLYRAWMAEFRGPFIHSFTCNDPALVDMILRERPMDFPKSP
RMAAGLQPLLGNSSFISNGETWLHQRRIIDPAFEGGRLREAFPAMWEAALAGTARLQPLADGAEIDIEPQTSH
IAADVIFRTLFSIPIEDEIAVQVFQAFREHQEAQPMVNLGGLMALPRWARLHTRRVRETARRIRGLIRQLVSV
RAAQIRAGTAPQDLATKIMTTPDPQTGDLFTEAEMIDEVATFFLAGHETGASALAWALYLLAENPDWQDRLAE
EARLNLAADFAATRNLPLARAVFREALRLYPPVAMTVRQCRQAETLRDRKAPRGAQLVLSPWHLHRHERLWVN
PDGFDPSRWETENGKTCLRRAFIPFSAGARACPGAGFAMVEGPLILAALVRAYRLEPGRERLVPIMRLTLRGK
DGIRLRLLPRG

>CYP1101A29(JCM7686_1890)*Paracoccus aminophilus*

MIPPKPPARSGKPSLWRFIRDFRRDILSAQSGKLYRAWMAEFKSPLIHSFTCNDPALLDLILKKRPLDFPKSG
RMAAGLTPLLGNSSFISNGETWRHQRRIIDPAFEGGRLREAFPAMLAASDAAVARIAPLATGHEIDIEPETAH
VAADVIFRTLFSLPIEDETASGVFTAFRAHQDAQPMVNLGGLMALPRWLHFHSRRTKRTAKHIRGLIERLVRA
RAGQIAVGTAPDDLATKIMTTPDPETGTRFTEVEMVNEVATFFLAGHETAASALAWSLYLLSQSPEWQDRVAA
EPEVRDFGGIRNLGAARAVFREALRLYPPVAMTVRECKHAETLRDRQAPKGAQLVISPFHIHRHERLWDNPDG
FDPARWETENGKACQRDAFLPFSAGPRACPGAGFAMIEGPLLLSALVRAYHIAPGQEVPVPVMRLTLRGKGGI
RLKLIPRQA

>CYP1101A30(BHK69_25865)*Bosea vaviloviae*

MERRGPGEFVPCHPEPLRKDPNLVQIVLGARHNLIGGWMAEHYRSGIDSFRIARRQIVVVNSPEYIKYVLVTR
HANFERKSPQMRRALEALLGDGLFISDGDVWKKRRPLVADIVHKRRLPEFGQTMEEAALSVADEWARLPEGAE
VELTEEMGRLTAAIISRAVFGKSIAREAAQQVVDGFSAYQRAADSFNLGYFLGADEGWPALGGRKRRQAIAMV
HGVVENVVNAHLAGEGDAGSMVDLLIRRNQRSAAAPLDVTALRNEAATIFMAGHETTATTLTWAWYLLANAPW
VAQKLHEEIEAVCGSRPPTLSDLPQLGWCRAVIQEALRLYPPVPLLPRQAIEADRIGHINVEKSALIMIAPWL
LHRAADLWDKPNHFLPERFLNGASINPFAFIPFAVGPRICPGMNFGQDEATLCLAILAQRFDVIPRKDYKVEP
VCRLTLRPNGGLPARVMQRSTERRM

>CYP1104A2(AZC_0056)*Azorhizobium caulinodans*

MLQLLQKPAPILEERGPDWFAPARPRPHDKRLPPLDLILAAKRNLISLWHVNDYRPTHGDMRLFGRQVFLANS
PETVKYVMATRHDNYERKSPQMRRALENLLGDGLFISDGETWRRRRPLVSDIVHKNRVPAFGLTMEAVTLSFV
ERWRARPAGEPFNALTEMAELTAEIISRAVFGNELGARAAHDVVDAFTRYQGLIDNFNIGYFLGANEGLPVFR
GPRLRRSVAQLDRVIDKVVTDHLEGRGEHDSMLDLLVKRQQKSPDLGLDVSALRNEAATIFMAGHETTAGVLT
WAWYLISNAPWVEEAVLAELDRVCGSRTPTVDDVAQLDWCRSVIEETLRLYPPVPILARQAKDADTIAGIDID
PAALVVVVPWLLHRSEGLWDQPERFRPERFLTDRPTPYTYIPFAIGPRICAGLAFGLTEAILCFAILIQQFRI
RVLPGTKVEPVSRLTLRPAGGLPVTAERRV

>CYP1104A3(RTCIAT899_PC05630)*Rhizobium tropici*

MLAWLDQKTEIVEHTDQSSRYFEPAHAAYYNGKPGQPLKLMLQARSDFLSIWRRADYREHVAEFKLLGRQLII
VNSPEAIRYVVAKRHENFERKTPQMRRALEYLLGDGLFISDKETWKQRRPLVSDIVHKNRVPAFGAIMQSTAS
ELADRWQSLGEGAEVNALFEMAGLTAEIISRSVFGNDLGEESANAVTEGFASYQSLVDSVNFGYFLGFDEGLP
IVKTPSLSRSVKRIHRIIDQVIEDHLAGKGDNSSMVELLIRRQQRNPELKLDVVALRNEAATIFMAGHETTAA
TLTWAWYLLAGAPWVEEAVHAEIDAVCGDRVPSIDDVAKLDWCRAVIEETLRLYPPVPILARQAAEADQIGDV
KVRKAALVLIVPWILHRTDSLFPEPHRFHPERFLGEARPAPYSYIPFAAGPRVCPGLQFGLTEAILCLAILAQ
RFRVRITDGHKVQPQCRLTLRPREGMPVTLHRRRG

>CYP1104A4(FA04_04645)*Ensifer adhaerens Casida* A

MLAWLQQSVNFTEELLEGKFAPVRADYYTGPPGKPLRLLLQARTDFLSIWRASDYTETISQTRILGRQIIVVN
SPDLIRQVVVKRHENFERKSPQMRRALEFLLGDGLFISDGDTWKQRRPLVADIVHAKRVPSFGPVMENTTSEL
VERWNRMPDGAEVNALHEMAGLTAEIIARSVFGNQLGDGSAAAVTEGFTSYQSLVDSINIGYFLGFDDGLPVL
RTPSLRRSVKRIHATIDKVVEDHLAGRGDHNSMVELLIRRQQRNPELKLDLVALRNEAATIFMAGHETTAATL
TWTWYLLSKAGWVEKAVHDEIARVCGDRTPTIDDVPQLEWCRAVIEETLRLYPPVPILARQTREADMIGDVAA
EPGSLVLIVPWLLHRTESLFQDPHHFHPERFTEGRRPTPYSFIPFASGPRVCPGLHFGLTEAILCLAIIAQRY
RVRVSQRHAVEPICRLTLRPKNGLPVTLHKREAPSHGAG

>CYP1104A5(OV14_2228)*Ensifer adhaerens* OV14

MAGWFHQPVKFREELSEEKFEPARAEYYAGPPGKPLRLLLQARTDFLSIWRNNDFTEKVSQIRLIGRQVILVN
SPDLIRQVVVKRHENFERKSPQMRRALEFLLGDGLFISDGETWKQRRPLVADIVHAKRVPAFGPVMERTTTEL
VERWNGMPDGTQVNALHEMAGLTAEIIARSVFGNQLGDDSAAAVTDSFTSYQSLVDSINLGYFLGFDDGLPIL
RTPTLRRSVKRIHQIIDKVVEDHLEGRGDHNSMVELLIRRQQRNPELKLDLVALRNEAATIFMAGHETTAATL
TWAWYLLSRAKWVEKALHEEIGRVCGDRVPTIDDVPQLEWCKAIIEETLRLYPPVPILARQTRQAEMIGDIAA
ERGSLVLIVPWLLHRTESLFEDPHHFRPDRFMGGRRPTPYSYIPFASGPRVCPGLHFGLTEAILCLAIIAQQF
RVTVVDGHKVEPICRLTLRPKGGLPVTLHRRQKASHDH

>CYP1104C1(ACP90_15940)*Labrenzia* sp. CP4

MTVSAQISGDTQKAEPTATKAEDFVPPYPFRYEKMPPVWSLIGMAKRNFLSIWGVDDFQSRLRSKKIFTRELV
ICNRPDVVREAFQTNHEVLQRKSPQMRHALQPLLGDGLFISDTDTWAKRRKVVAPIIHGSRVKGFAPIMIETI
EEQRADWASQGEGAEVDALADMAHLTAEIICRTIFGRQLGKDHAAEVVQGFSDYQRHIDQVDILSLFGLPEWL
PRFRGRAIKKPVERIMTVLDKIIANYEAQKEKGEASVIGGLLEARDENGEPLSREAIISEAAVIFMAGHETTA
NTLAWAWFLLSQCDKSRAKLQAELDTVLAGRSPTFQDVPNLPYTKAVIEETLRLYPPVPILAREAMADTSIGG
KSVPKGSLVMVVPWLMHRNPVLWSKPDVFDPGRFLNPKSKKPNKYGYVPFSIGPRICAGLQFGMTEAILSLAI
LAQDFELKLKDGTDVQPVARLTLRPGENLPMTLHPRHS

>CYP1104C1(B0E33_27775)*Labrenzia aggregata*

MTVSAQISGDTQKAEPTATKAEDFVPPYPFRYEKMPPVWSLIGMAKRNFLSIWGVDDFQSRLRSKKIFTRELV
ICNRPDVVREAFQTNHEVLQRKSPQMRHALQPLLGDGLFISDTETWAKRRKVVAPIIHGSRVKGFAPIMIETI
EEQRADWASQGEGAEVDALADMAHLTAEIICRTIFGRQLGKDHAAEVVQGFSDYQRHIDQVDILSLFGLPEWL
PRFRGRAIKKPVERIMTVLDKIIANYEAQKEKGEASVIGGLLEARDENGEPLSREAIISEAAVIFMAGHETTA
NTLAWAWFLLSQCDKSRAKLQAELDTVLADRSPTFQDVPNLPYTKAVIEETLRLYPPVPILAREAMADTSIGG
KSVPKGSLVMVVPWLMHRNPVLWSKPDVFDPGRFLNPKSKKPNKYGYVPFSIGPRICAGLQFGMTEAILSLAI
LAQDFELKLKDGTDVQPVARLTLRPGENLPMTLHPRHS

>CYP1104C2(SL003B_1954)*Polymorphum gilvum*

MDQSVAAVVAEREPDYVPPHPYRHETMPSVIELMGLARKNFLSIWSKGNFSSRLMSQQVLRRLLVVCNSPDVV
QEAFQTNHAVLQRKSPQMRHALEPLIGDGLFISDSDIWRRRRKVVAPIIHGSRVPGFAPIMVDTIEEKRAEWA
ARGAGGEVDALAEMAHLTAEIICRTIFGRNLGRNYASEIVEGFSDYQRYIDQVDLPAMLGLPEWLPRFRRPAV
HRSVKRILGVLDEIIDSYQAMKDSGEVSVIGGLLEARDEDGAPLSREAIRNEAAVIFMAGHETTANTLAWAWF
LLSQAPRVRERLHAELDSVLGDAPPSFADVARLPYTKAVIEETLRLYPPVPILAREAMADTTVGGKRIPKGTI
LMVVPWLLHRNPTLWPDADAFRPERFLEGEGTRPSKYGYVPFSIGPRICAGLQFGLTESILSLAILARAFDLR
LKPGADIQPVCRLTLRPGDALPMTLHPRTA

>CYP1104E1(GDI2364)*Gluconacetobacter diazotrophicus* Pal 5 (Brazil)

MNFRLCADRWNSMEFIPPFPPRPKTSLSVFELLRRGTQNFLNIWEEKAFEYQTMSMQVLARQVFICNSPDTVR
HAFITRAENFQRKSPQIRNALSPLLGDGLFVSDGETWKQRRQMVSPVLHTSRMDQFAPAMVETVGELADRWAA
LPDGATFDVLKVMAQLTAEIICRAVFGRTLGAEHAREVAEAFTEYQKYVDQSDLASFGLPSWVPRRNGAKTRR
ATARIHAVLDGIIADLQRTEDDGSVIRMLMRDGVLDATALRNEAAVIFLAGHETTANCLSWVWYLLSQAPEVE
ARLHEELDTVLGSRAPTFADVSQLVYTRAIVEETLRLYPPVPLLAREAKEDDTIRSRKVKAGALVMVVPWLLH

RHRLYWRKPDHFMPERFLPGSPDAPQKYTYVPFSIGPRICPGLSFGLVEAIICLASLARGTTLRLAPGAVVEP
VCRLTLRPGDTLPMTVWKRTAAAGTRPVPAAASAQRCPVHHG

>CYP1104E1(Gdia_0609)*Gluconacetobacter diazotrophicus* PA1 5 (JGI)

MEFIPPFPPRPKTSLSVFELLRRGTQNFLNIWEEKAFEYQTMSMQVLARQVFICNSPDTVRHAFITRAENFQR
KSPQIRNALSPLLGDGLFVSDGETWKQRRQMVSPVLHTSRMDQFAPAMVETVGELADRWAALPDGATFDVLKV
MAQLTAEIICRAVFGRTLGAEHAREVAEAFTEYQKYVDQSDLASFGLPSWVPRRNGAKTRRATARIHAVLDGI
IADLQRTEDDGSVIRMLMRDGVLDATALRNEAAVIFLAGHETTANCLSWVWYLLSQAPEVEARLHEELDTVLG
SRAPTFADVSQLVYTRAIVEETLRLYPPVPLLAREAKEDDTIRSRKVKAGALVMVVPWLLHRHRLYWRKPDHF
MPERFLPGSPDAPQKYTYVPFSIGPRICPGLSFGLVEAIICLASLARGTTLRLAPGAVVEPVCRLTLRPGDTL
PMTVWKRTAAAGTRPVPAGASAQRCPVHHG

>CYP1104E2(CAK95_26735)*Pseudorhodoplanes sinuspersici*

MWEEDAYDDYEFVSTQVLARKVILCNSPESIQFAFSTRNASFERKSPQERFALAPIIGDGHFITDGETWKRRR
RLIAPIIHVSHLAEFAPTMVEVAGETRDRWLKRPAGQSFDVLTEMAQLTAEIICRSVFGRELGHEHAQQVVDS
FSEYQRQIAQLDMFSFLGLPDSFPRPRSRALRRAVQGIHDVLDGIIDSYQTRKKDKNDASLVGRLMEAHDEET
GERIQGEALRNEVAVLFMAGHEGNANALTWTWYLLSQAPDVEARLHDELDRVLGDSMPTFSDLPRLTYTRAVL
DESMRLYPPVPFLSREAIVDENYKGESIPRGSLIFVVPWLIHRHKKFWDKPDHFIPERFLPGNGPTPSKFAYI
PFSIGPRVCSGLGFGSAEGILCVATIAQALRLRLKQGYKVEPVCRLTLRPAGGMPMSVEPRQHAAKVAPPDEP
AAMACPFGHG

>CYP1107A3(A6J80_12355)*Paracoccus yeei*

MTRYPARVPLHRKPLGVIGSALVARRNVLELIPEIAVRQPMVSGKVGKRWHMVMDPDALRVVLKDRVEDYPKS
LVTKLLLEPAVGESLFVAEGAHWRWQRRAAAPAFAQRHVEALAPVMTAAAEASARRLSAAAGGTVDVFAETVA
ATFEVISDVTFSGDEGFDRDAVHHAIDAYIESTARISVLDILGLPGWIPRPGRLRSGGDLRRMKRVADDAIRA
RARSGPRPVPDLLDLLRAGEDPETHRRMTPGELRDNLLTFIVAGHETTALTLAWAFYLLAFDPAVQDRAAAEA
RAALGDRAATAADLPRLPYVRQIVDEALRLYPPAAFLSRTARIHDSLGGREVLPGDTVMLPIYALHRHHLLWD
DPDAFDPDRFAPGVTRDRFAFLPFGAGPRICIGASFALQEAVIILATLVARLRFDLTARQPQPRMILTLRPHG
GLWLSVRARA

>CYP1107A4(Pden_2272)*Paracoccus denitrificans*

MSPDHPPERYPARVALHRQPMGVIASAMTARRNVLELIPEIATRQPMVSGKTGKRWHMVMDLGALRRVLKDRV
EDYPKSLVTRLILEPAIGNSMFVAEGAHWRWQRLAAAPAFAQRHVEALGPVMTAAAEASAQRLAAADGPVDVF
AETVAATFEVISDVTFSGDEGFDRDAVHHAIDAYIAGTARISVLDILGLPGWIPRPGRLFSGGDLRRMKRVAD
EAIRARARSGPRPVPDLLDLLRAGEDPETHRRMTPAELRDNLLTFIVAGHETTALTLAWALYLLAFDAQVQHR
AASEAVAALGGRAATAADLPRLTYIRQVVEEALRLYPPAAFLSRTARIHDRLGGREVRPGDTIMMPIYALHRH
HMLWDDPDRFDPGRFAPGVTRDRFAFLPFGAGPRICIGASFALQEAVIILATLVSRFRFELTGRQPQPRMILT
LRPHGGVWLKVAERS

>CYP1107A5(BMG03_18115) *Thioclava nitratireducens*

MDYAATDDTHRLPARAPLAEHPLGILASFRAARRNLLEIIPELATHAPIISGNTGARWHMVMDPDSLRHILKT
RVEDYPKSVVTKLILGPAVGEGLFVAEGAEWRWQRRAASPVFAARNMEALAPVMGAAAEASAARISTAADTGR
AVDLHQEMVAATFEVISDVTFSGDEGFDRAAIHRAIDAYIAQTAKVSLLDLFALPAWVPRPHRVISSGVVRDL
KRHADSAIEARRASEAKAVPDLLDLLDGAQDPETGREMSPAELRDNLLTFIVAGHETTALTLAWALYLCAFDP
AVQEAARSEAQAALGAHAATVDDLPKMPLVAQIVNESLRLYPPAGFLSRTARKPDTLLGREVRAGDTVMLPIY
ALHRHHALWDDPHAFRPDRFADPTHDRFAFLPFGAGPRICIGAGFAMQEAQIILATLLARFRFTAVPGREPVP
EMILTTRPKGGIWLTVKRA

>CYP1107A6(JCM7686_0408)*Paracoccus aminophilus*

MLSDPALLPPRVPAHTKPLGVIGSALTARRNVLELIPEIAYRQPMVSGRTGKRWHMVMDPEALRRVLKDRVAD
YPKSLVTKLILGPAVGQSMFVAEGAHWRWQRLAAAPAFAQRHVEAMAPVMTAAAAASLRRIAAAQGEVDVFAE

MVAATFDVISDVTFSGGEGFDRDAVHTAIDSYIAGTARISLLDILGLPSWVPRPGRLVAGRELRRLKRTADHV
IDERKRQGAQPVPDLLDLLQAGEDPETGRRMTTPELRDNLLTFIVAGHETTALTLAWALYLCAFAPEVQERAS
AEARAALGDRIAGAADLPALPYLRQIVEEALRLYPPAAFLSRTALAADELGGREIRPGDTVMLPIYALHRHHL
LWEEPDRFDPERFAPGVTRDRFAFLPFGAGPRICIGAGFAMQEAVIILASLLARFRFETTARVPTPRMILTLR
PEGGVWLRAIPR

>CYP1107A7(AKL17_0406)*Defluviimonas alba*

MDARTPPATPVQVPLATVPLGILGSLRVAQRNLLELIPEIALRQPMVSGRTGKRWHMVMDPAALRRVLKDRVD
DYPKSDVTKLILGPAIGDSLFVAEGAHWRWQRRAAAPAFSVRNVESLAPVMSLAADRASARIAAAHGRAANLF
DEMVTATFEVIADVTFSGGEGNGGLERDAVHKAIDSYMAQTAKLSVLDIIGAPMWVPRPSRMRSGPVMRQMKQ
VANAAIDQRRSDGPRPVPDLLDLLLSGVDPETRRTMDTAELRDNLLTFIVAGHETTALTLAWALYLCAFDQDV
QDQARAEAQGVLGDRAATAADLSQLPLIHRIVEETLRLYPPAAFLSRTALAQDELCGREIRPGDTVMLPIYAL
HRNHALWKDPHCFDPSRFEDRSFDRYAFLPFGDGPRICIGASFAMQEAVIILATLLARHHFALVPHRAPKPVL
ILTLRPEGGVWLMVEPARNAGVGQGSNLGEERS

>CYP1107A8(ROSMUCSMR3_03777)*Roseovarius mucosus*

MSPIPRQTPQTLPAHVPLVTEPMGVLASLKAARRNILSIIPEIATRQPMVSGRTGKRWHMVMDPGAIRRMLLE
ELDNYPKSIVTKNLLRPAIGESLFIAEGAHWRWQRRTAAPVFSHRNVMNLAPIMTAAAEQSAARVAAAGPRAV
DMAAEMVRTTFDVIADVTFSGDGMFDVDAVHRGIDAYISEAGKISLFDILGFPDWVPRPGRVMSGGSVAEMKR
VADEAVEARRARGSEGVPDLLDLLLDGEDPETKRRMSTPELRDNLLTFIVAGHETTALTLGWSLYLCAFDQAV
QDRARAEAQAVLGGRAATGADVARLPYIRQIIDEALRLYPPAGIISRTAQVADTLCGRNIRPGDTVIIPIYAL
HRNHQLWPEPDAFNPDRFADRKTIERYAYLPFGDGPRICIGASFALQEAVIILATLLSRFRFTPVPGRDPDPV
MILTLRPQGGVWLMAEPV

>CYP1107A9(MALG_03152)*Marinovum algicola*

MPDTTLPPRLPVHVPLGTRPLGLLASLSAARRNLLSIIPKIATTQPMVSGRTGARWHMVMDPDAIRAMLLERL
DDYPKSLVTKNLLRPAIGESLFIAEGAHWRWQRRAAAPVFSHRNVMNLAPIMSAAAERCCARIAAAGPRAVNL
HDEMVTTTFDVISDVTFSGDSVFDKSGVHRAIDDYIAAAGKISLFDMLGFPDWVPRPGRLLSGNAVRQMKSVA
DSAIDARAARGHDGVPDLLDLLLAGEDPETKRRMSVAELRDNLLTFIVAGHETTALALSWSLYLCAFDQEVQE
RARAEAQSLLQGRAAGGEDVAELPYIRQIVDEAMRLYPPAGIISRTARKADTLCGREVRPGDTVMVPIYALHR
NALLWQEPDAFRPERFADRKAVPRYAYLPFGDGPRICIGASFAIQEAVIILATLLSRFRFTPVDGRTPEPVMI
ITLRPEGGVWLEAEAV

>CYP1107A10(BOO69_01850)*Sulfitobacter* sp. AM1-D1

MSTTLPVRVPLVTEPWGILKSLAEARRNVLSIIPDIATRQPMVSGKTGKRWHMVMDPQAIREVLLDRLDDYPK
SLVTKNLLKPAIGESLFIAEGAHWRWQRRAAAPVFSHRNVMNLSPIMTAAAERTVARIEAAGPRAVNMLDEMV
TTTFDVIGDVTFSGGDTFDRDKVHGAIDDYISEAGKISLFDVLGFPDWVPRPGRLMSGAALKEMKSMADSAIE
ARAERGHDGVPDLLDLLLEGVDPKTKRQMSTAELRDNLLTFIVAGHETTALTLSWAMYLLGFDPAVQARARAE
VQDVLQGRACTGDDVEKLPYIRMIIDETLRLYPAAGIISRTAQKHDTLCGREIRPGDTVMIPIYALGRNKLLW
DDPDRFDPDRFADRKSIDRYAYLPFGDGPRICIGASFALQEAVIILATLLSKFEFAPVPGKEPKPVMILTLRP
EGGVWMTAKPVQRTAAG

>CYP1107A11(AYJ57_06345)*Yangia* sp.CCB-MM3

MIETSSEPQSPAPLPVRAPLVTEPLGILASLRAARRNLLAIIPDLATRTPMVSGRTGARWHMLMEPGAIREVL
LERLDDYPKSMVTKNLLRPAIGESMFVAEGAQWRWQRRAAAPAFSARNMSNLGPIMAGAADRAAQRIAAQQGR
AVDLYEEMVTTTFDVISEVTFSGGEAFARDAVHRAIDGYIAEAGRISLFDVLGLPDWVPRPGRMRSAGGLTEM
KRVADAAIDDRAARGPREVPDLLDLLLAGEDPETTRRMNTAELRDNLLTFIVAGHETTAITLSWALYLLAFDP
EAQERARDEVMAGVSGGTASAAEVAELPYLRRVIDETLRLYPPAGIVSRTAQVPDELCGREIKPGDTVMIPIY
ALHRSHRLWEDPDAFRPDRFLEKPPRYAYLPFGDGPRICIGAAFALQEALIVLASLLKRFRFSAVEGRAPEPV
MILTLRPEGGVWLEAEPL

>CYP1107A12(RLO149_c033920)*Roseobacter litoralis*

MAKDELTDKGRIPVRVPLVNAPVGILKSWQMMRENVLNIIPEQATEQPVVSGKTAKRWHMVMDPAAIQRMLLD
RVEDYPKSTVTKNLLRPAIGESLFIAEGAHWRWQRRASAPVFSHRNVAALGPVMTAAADRTSQRVAQAGPRAV
NMLDEMITATFDVIAEVTFSDDGSFDRQAVHDALETYVNDAGRISLLDVLGLPDWIPRPGRTTASAEMTQMKQ
DADAAIEARAARGPGDVPDLLDLLLTSQDPKSGRSMTTSELRDNLLTFIVAGHETTALTLAWSFYLCAFDQDV
QDKARAEAQDVLGGRAATTTDVDHLPYIRQIIDEALRLYPPAGVVSRTAQKPDTLCGADIQPEDTVMIPIYAL
GRHKLLWENPDQFDPDRFEDRKSIKRYAYLPFGDGPRICIGASFAVQEAVIILATLLSRFRFTPVRGRDPKPV
MVITLRPEGGVWLMAEPV

>CYP1107A13(RD1_1394)*Roseobacter denitrificans*

MAKDAFTDDNRTPVRIPLATEPVGILKSWQMMRENVLNIIPEQATEEPVISGKTAKRWHMVMDPGAIQQILLD
RVEDYPKSTVTKNLLRPAIGESLFIAEGAHWRWQRRASAPVFSHRNVAALGPVMTAAAERTAQRVAQAGPRAV
NMLDEMITATFDVIAEVTFSDDGSFDRNAVHKALETYVNDAGRVSLLDVLGMPDWIPRPGRTTASAEMAQMKQ
DADAAIEARAARGPGDVPDLLDLLLAGQDPKSGRSMTTSELRDNLLTFIVAGHETTALTLAWSFYLCAFDSRV
QDKARAEAQKVLQGRAATTADVENLPYIRQIIDEALRLYPPAGVVSRTAQKRDTLCGADIQPDDTVMIPIYAL
GRHKLLWDNPDQFDPDRFQNRKSIKRYAYLPFGDGPRICIGASFAVQEAVIILATLLSRFRFTPVKGRDPKPV
MVITLRPEGGVWLMAEPV

>CYP1107A14(BWR18_13465)*Tateyamaria omphalii*

MPDTALPRPLPVRVPLVNEEWGIWKSLQMARSNVLRIIPELSTRLPIVSGKTGKRWHMVTDPAGIKRMVLDNL
DIYPKSVVTKNLLKPAIGESIFIAEGADWRWQRRTAAPVFTHRNMMNLAPIMTGAAERCADRIAAAGPRAVNL
LDEMVNTTFDVIADVTFSGGEAFDRSGVHRAIEDYISEAGKISLFDILGAPDWVPRPGRVVSGQAMRETKAVA
DRAIEERAARGHDGVPDLLDLLLAGEDPKSGRKMTTAELRDNLLTFIVAGHETTALTLAWSMYLLAFDPAIQD
RARAEAQAVLQGRAATGEDVEHLPFIRCIIDEALRLYPPAGMVSRTAMEDDVLCGREIRKGDTCIIPIYALHR
SKLLWDDPDTFDPDRFADRKSVDRYAYMPFGDGPRICIGASFALQEAVIILATVISRFRFTPVPGKEPEPVMI
LTLRPEGGVWLTAEPA

>CYP1107B1(Ga0080559_TMP3064) *Salipiger profundus*

MSDFVPPTVTPPGKPLGIMASLRAVRRNVLNIIPAISYTQPIVTGTTGSARWHMVQGPEALRRIYLDNVDNYP
KSEVMLRMLRPAVGNSLFTSEGAHWRWQRRAIAPVFAARHVEALAPMMTQTAERAAGRLAASGGQAELVREML
SATFDVICDVALSGREHFDAEVYGEAITRYFLTVGRASLLDFLEVPPWVPRPAELFGRSAVKTMHSMVSRAID
ARRVRGAGPMDDLLDHMLKAKDPETGRTMSPQELLHNMQFFIVAGHETTALAITWALYLLANDPKAQERAHAE
ARAVLGDRPAEHADLARMPYIEQVLDESMRLYPPVGFLARNVLATDQLYDREIRRGETVFLNVYALHRHRMLW
NAPDAFDPDHFSPEAKAGRDRYSYSYLPFGAGPRVCVGANFAMMQAQIILSTLLARFRFHSTGERPEPVMHMT
VRPDPGVTLRVEPV

>CYP1107B2(Ga0080574_TMP976)*Pelagibaca abyssi*

MTQPLPPRIEPATQPLGMLASLRAARSNVLRIIPAIAYTQPIVTGTTGHARWHMVQGPEGMKRLFLDNVENYP
KSEVMLRMLRPAVGDSLFTSVGAQWRWQRRAIAPVFAARNVEALAPMMTATAERACERLAGAGSAEMVAEMLS
ATFDVICDVALSGREHFDAETYGEAITRYFLTVGRASLLDFLEVPHWVPRPAELFGRGAVKTMHTMVARAIEA
RRREATGGADDLLDHMLAARDPESGRTMSPRDLLHNMQFFIVAGHETTALSLAWALYLLAHDQPAQDRAREEA
KDVLQGGPCGHEELDRMPGITAILDETMRLYPPVGMLARNVREADTLYDREIRPRETIFVNTWSLHRHHDYWE
RPDHFDPTRFGPDQQRNRYLHLPFGAGPRVCVGANFAMMQAGIILATLLSRYRFAPKGPRPEPIMHMTVRPDP
GVTLEITPH

>CYP1107B3(Ga0080559_TMP4197) *Salipiger profundus*

MQPVMDPAVPDFVNKLQRIDTYDEIEEIMKSQDFVMAGADERFIFLEDTLIMAEGEQHSELTRLFAPLMSRQA
VAYYELKLVEPAIEASINDMRSKRGEDGKVRTDVVPLIHAALVRIASKVTGVDGVDSLEDTERFRDMVLAVSA
ATTASYSSASDPGRIIQKGKDALQALVDEWMQASLDRRIELAAKHKAGEIPAEELPRDVLMSLCLAEDLSRPD
DGTRIPYVWRQCAQFLTGSIKTTSHMLPHAFIHIDEWIKEHPEDRPNLTDPEWLHKAAAESFRLHQTTPARFR
KAVKDLELSTGRKVDAGEMVAIHAPVANVEAFGDDGRYFNPYREVPKGKKAWGMTFGIGKHSCIGQNLVTGIM
NKGDAKHGTHGTAVRLLKAMYELGAELDPDNPPQRPHDNLHDTWESVPVILHKA

>CYP1107C1(RGUI_1731)*Rhodovulum* sp. P5

MIHGGAGQWGAAVALDSGAMTRVPVGVRPANGPLGPLAAYRTARRNVLELIPAEAYSAPFVFHDGPSPWLYVT
EPAAVQHVLTEHEAYPKSAMLLRLMQPRRGSNLAVTSGADWRRQRKALAPAFTPQALAAMGPATGAAARDAAE
RLATRSGEVVDVLPHMVEATTDVICEVMLSGRDVIDRRALTQSVDRLVGAITRVSVFDLFKIPNRVPRPAEVF
DQSRARMDRLADRLIADRRSRGPSDPPDVLDLMIGPRDSDCGLDDLEIRNNLLGFLFAGHEPTALSLTWALYL
LAFDPEVQAQARGEADAAISGGVARHDDLPRLGYIRQVVEEALRLYPPAGFLTRTAARDGEIGPHFVPKGANV
VLPVYAMHRHEGLWTAPNAFDPGRFGPDRLTPREKSAFLPFGGGPRICIGATMAVTEATLILATLLTRLSFAL
PPGFSPQPQMWFTLRPGTGMKLRISKRPS

>CYP1111A4(BSY18_3618)*Blastomonas* sp. RAC04

MMPSPQLEDLSDPGFDPFVIEKLSTGDCHDPYPQIHALMQRGPVVEGSYRSQFTSVPDVQMGHLPQVMVLGYD
AVLHVLTHPEIFTNKEAFSPNLGRAFGNTVTVMDAPEHPRYRKIFQKAFLPQVVSKWGETVVDPVVDRLMAKF
LPHGRADLIEDFTHHYPFQVIYAQVDLDPAQAPVFHKLAIAQLLSSIGAPQGQEASKKLGDFFGALVEQKRAK
PGTDLISHLATVEADGERLPDDVLISFLRQLMNAGGDTTYRGTSVLLTGLLTHPEQLKAVAEDRSLIPQAIDE
ALRWEGPVASTFRYASVDTEIAGVPIKKGTFVNTVLASANRDPSKFPKPDSFDIFRSRTPRHLAFASGPHLCI
GQHLARVEMTRALSALLDRLVNLRLDPDKPEPRVTGHILRAPEHLWVRFDS

>CYP1111A5(Swit_0364)*Sphingomonas wittichii*

MESAVRIQEFDDPNYDPFNSDEINFGDHADPYPLIARWRAEAPVIEGGYRPLMGLPAALYPDRKMFTVVGSRE
VNEVLTDTDRFSNAGYKFNLGVTFGQGSISTMDNPEHGRWRRIFQKIFLPQYVKKWGETIVDPVVHGLMGKFL
PLGRADLIEQFTLRYPFEVIYHQLDLPEEDVRTFQRLALGQTDYVNHEKAIEAGRKLGDYFTALVEERRRDPG
DDLVSLLASTEADGEYLPHEVLISFLRQLMNAAGDTTYRGTSILLTALLEHPDQMEAIRADRGLIPQAIEEAL
RWDGPVAVQMRMAAVDTELAGVAIPAGSLLDVVAGAANRDPEIYSDPDRFDIFRDRKPHFAFSRGPHICVGQH
LARVEMTRALHAVLDHLPGLRFDPDEPRPRIQGSMMRVPRHLHVRFGD

>CYP1111A6(AZL_b01530)*Azospirillum* sp. B510

MNASAPRIRDFDDPSFDPFSAVDEGFGPHVLDPYQRLAELRREAPVHHIDYRVLFGLEPDITLGGLPHALVVS
YDAVAQVYNDPVTFSNTIYERNLGIAFGRSISVMDAPEHPRYRRIFQKAFLPNIVSKWGESLVAPVIDRLLER
FIDRGHADLVKEFTALYPFQVIYEQLGLPKGEVEVFHKLAAALTTFSVDPRYAMEASAKLGEYYDALTDLRAQ
QPGDDLISLLATAEVDGERLPKDVVVSFLRQLNNAAGDTTYRATSCMMMGLLSNPEQFDAVRRDRSLIPAVIE
EAIRWECPVLVGSRQAIRDVTLCGVDIPAGTVIDVGNGAANRDEARYPDPDRFDIFRPAGGARHFGFAYGPHV
CIGQHLARVEMTRALTGILDRLPNLRLDPERPAPQIRGINLRAPSALHVVFG

>CYP1111A7(Sphch_3576)*Sphingobium chlorophenolicum*

METAVRIQEYDDPSYDPFGADDLNFGDMLDPYPEIAKWRAQAPVLEGDYRPKMGLQSATYPDRKMFTVVGSDE
ILTVLTDTDRFSNHGYKFNIGETFGQGSISVMDNPEHGRWRKIFQKIFLPQYVKLWGETIVDPVVHKLMDKFV
GTGRADLIEQFTLLYPFEVIYQQLDLPESDVNTFQRLAIGQTDYMHIDKAQEASRKLGEYFTALVDERRRNPG
DDLVSLLANTEVDGDYLPPLVLISFLRQLMNAAGDTTYRGTSVLLTALLENPDQLDAIRADRKLIPVAIEEAL
RWDGPVAVQLRMAKQDVVLGGLEIPAGSLLDVVAGAANRDPAIFPDPDRFDIFRERKAHFSFSRGPHICVGQH
LARVEMTRALHAVLDNLPNLRFDPDKPRPEIRGSMMRVPKHLHVRFGD

>CYP1111A7(TZ53_05360)*Sphingobium* sp. YBL2

MEPAVRIQEYDDPSYDPFSADDINFGDTLDPYAKIAKWRAQTPVLEGDYRPKMGLQSATYPDRKMFTIVGSDE
ILTVLTDTDRFSNHGYRFNIGETFGQGSISVMDNPEHGRWRKIFQKIFLPQYVKTWGETIVDPVVHKLMGRFM
GTGRADLIEQFTLLYPFEVIYQQLDLPESDVRTFQRLAIGQTDYMHIDKAQEASRKLGEYFTALVDERRRNPG
DDLVSLLANTEVDGDYLPPLVLISFLRQLMNAAGDTTYRGTSVLLTALLENPDQLDAIREDRKLIPVAIEEAL
RWDGPVAVQLRMAKQDVVLGGVEIPAGSLLDVVAGAANRDPAIFPDPDKFDIFRERKAHFSFSRGPHICVGQH
LARVEMTRALHAVLDHLPNLRFDPDKPRPEIRGSMMRVPRHLHVRFGD

>CYP1111B1(JI59_21480)*Novosphingobium pentaromativorans*

MATIAPIEYFDEADFDPFVIDDDVFGEIEDPYAIFDRARANGPVQPGTILELLGAPADSIVAGIPQFRVIGHA
EVQAVMRNAALFSNDVLKMNLGVTFGDTISAMNPPKHTQVRGLFQSTFMPKQVAKWSETLVDPVVNDLIGRFI

GDGRAELVEQFAKRYPFEIIYRQLGLPQRDVQTFHRLAVTLTFNSDYIRYGKEASRKLGVYFQNLLEERRRNP
ADDLVSLLGQLGGDNGLSDETIVSFFRGLINAAGDTTYRATGCMLMALLENPEQLEAVRQDRSLVPLVLEETL
RWNGPIIFTQRMVMQDTELGGVRMPAGAIVNVSMAAANNDPAVHENPRKFDLFRGTKSHFGFGLGPHMCLGQH
LARLEMSRALNAILDRLPGLRFDPDMPKPTVKGGLMRTPRDIHVLFDPS

>CYP1111C1(JI59_21655)*Novosphingobium pentaromativorans*

MKHQPVIQGFDDVDFDPFAFFAASFGEIRDVYSLYDRLRQQGTVVEGSVLDLLCGVTEPQLEGLRQFTVLGYE
EAMAVNRDPALFSNATLNDYLSITFGPTLTALDPPVHTEVRRVFQRIFLPAQVAKWSETFIDPVIDRLVTDLA
TAGKAELVEDFAKKYPFQIIYRQLALPEQDIETFHKLAVAQGLMGEYRHHAVEASQKLGAFLTDIVAERRRNP
GADLISTLATSEVDGARLEDQFIIAFLRQLTLAAGDTTFRATGTLFAGLLSTPGLLDEVRNDRSLVAPLIEET
LRWDGPSGYTLRTATRDTELGGVAIPAGSVVNVSFSAANRDPNAFANPNAFDVHRPKKPHMGFNTGPHMCVGQ
HLARLEMSRAVNAMLDRFPNMRFDPAQPRPHIEGVVFRTPYNVHVLLD

>CYP1116B1(Mesci_0689)*Mesorhizobium cicero*

MSFAENITVEALEADPYPIYAELRRSAPVAFVPAVNLWFVTRWKDVESVAKSPEIFSAVVGSSPVERSFGKPT
ILTTDGETHKQLRQGVDPKYRPRTVAAYAGDLVRSIAEPFLDRIANQGSAELMADYFEPVSTLSLARSLGLAG
IDMPTLRRWFYGLAQGAINFENDPKRQEIADAISAEVGDAILPTLERLARQPDDSALSHMLHDGMPEGTTRPI
DFLMPSIKVILLGGMQEPGHGAGSILAGLLAHPKQLRQVLADPETFVPRAVDEGLRWVAPIGTQTRQTTRAVE
LGGVTIPAGAAVAALVASASRDESRFADPDRFDINRNEGNHAAFGFGHHFCSGRFFAREQMCLAVRLLLQRFP
DLSLVPSREPVFRGWEFRAPTTLHVSLGVRHQ

>CYP1116B2(Mesop_0704)*Mesorhizobium opportunistum*

MSFAENITVEALEADPYPIYAELRRSAPVAFVPSVNLWFVTRWKDVELVAKSPDIFSAIVKAGVSPVERSFGK
PTILTTDGEIHKHLRQGVDPRYRPRTVASYAGDLVRSIAEPYLDRIASQGSAELMADYFEPVSTLCLARSLGL
ADIDMPTLRRWFYGLAQGAINFEKDPKRQEIADAIGAEVGDAVTPTLERLVREPDDSALSHMLHDGMPEGKTR
PIEFLMPTVKVILLGGMQEPGHGAGSILVGLLRSPDQLRKVLDDPETFVPKAVDEGLRWVAPIGTQTRETTRA
VEIGGATIPAGAPVAALVSSASRDERRFADPDRFDIDRDEGNHAAFGFGHHFCSGRFFAREQMCLAVRLLLER
FPDLRLVPGKEPVFRGWEFRAPTTLHVSFGVGPK

>CYP1116B3(Rleg2_4431)*Rhizobium leguminosarum*_bv. trifoliiWSM2304

MTSPETVTVEDLEADPYPIYAQLRREMPVAYIPAVNLWFVTRFKDVEYLSKTPEIFTAAVESSPLDRTWGKPT
ILTADGPVHKNLRSGVDPKYHPKRVAAYMDDLVLPIAREYHDRLLASGGGDLMSDYFEPISILSLTRSLGLGH
VDLETLRRWFFGLAQGAINFECDPERQAVADAISTEITGIVTPVMQKLIEEPDDSALSHMLHDGMPEGETRTI
DFVLPTLKVLLLGGMQEPGHGAGSIMAGLLQNPDQFAEVKGDPDALLPRAVDEGLRWVTPIGTQTRQTTKAVE
IGGVTIPANQPVAAVLASASRCESRFTDPGRFNIHRNEGSHAAFGFGHHFCAGRWFAREQITSALRYLIDRSP
EIALRDDGPVQFRGWEFRAPSTLNVTLR

>CYP1118B1(A6F68_01696)*Altererythrobacter dongtanensis*

MAQTTPLPTGIRLTSLDAEYRENPAGIYDRLRSEDPVHFDEQFQRYFVTRAADIQEVLKDRSMGRDPDKARPD
SYVKFFIDEASFEKSMLFLDDPDHARLRSLVTQAFGFRAISELRPHIAEVANRLLDQLEGQDEFDVISEFSSP
LPIIIIAEMLGVDPADKDDFIRWSQATDVAFSPMRTEDQNRQLITARDSMSEYFYAAIAERRREPRDDLLSAL
IEAADGQHRLSDQEIVVSARLLLVAGNVTTTDLIGNAVRLLLEDDEQRRRLEAEPELIDNAVEEVLRLDPPVT
AVARITSQPVTVADTAVPAGENLFLATHSAALDPDLNPNPGRFDIARDKPKHIAFGGGAHFCLGAQLARAEAQ
IAIPLLLARFPGLRLVEAPERKIAPGFNGYKSLKVAVR

>CYP1135A1(Jann_2276)*Jannaschia* sp. CCS1

MAEHFVTEYRDAEQTLRLPDLHQALYDADTVFLPRTVVCLHGEAHARKKRIFSGVFNRRFFKHYQNHVFPRAI
KESIDPVLAEGRGDMAKFAYRVLINLTADSAGIDRRHTQEDTDRLLALLAKLGHAPTMGQLRDPSERETLLAE
LEDALAAFRDEFFEPSLARRRGLVAQYEAGEIERSDLPQDAIVAQLLAMPTDEDIPYDERMKDSAFFILAGAF
TTANVLMNFTKNLLDWFDDHPEDRANLLADPIMMQRFIWENTRLHPASPVTKRRALCPMHLPDGHDAEQDEYV
TMDLTRINRNAELFGEDPEAFDPNRDLADNMPLHGLSFGGGAHVCLGKLLAVGVKITDDNKDAEETEVGTMAL
VARELLRLGIARDPGHEAEIDESTDRRHFKTLPFIFDPAVAVG

>CYP1137A1(G432_10785)*Sphingomonas* sp. MM-1

MNQPRFSGAYTDAANWLYRMAREHNGENIRAEVNGADVLLVQRLEDAEHVLRRRADNYRKNMHWFRHMLGPSR
FSEVGEAWEIRRELTQHYLAKFDRERTHGLAVRYADEAVGGLAGRSAAGDAVLDESLLRRMTVSVLIENFFGV
PLAETRVDIDRLATLMEFGSEYAFAPTVDTSALNHRHLAALPRLRREIFDDFRLFREGALPDNAMLEGMLAAD
RDPENDIMLEHELLTFFAAGAETTASGIGWACYLLARHPDVQRGLRDALASFWESPRRDWAHLSTIAPLEAFV
SEALRLYPPTPLVTRLATGPDRIGDHAIAEGDNIFVSIPGVQHDARLHPDPWALHLDRYRNNASAGSNIAFIF
GPRVCGGKKFALVELAAFVATFLKRADFALTSDAPPRFHWKSLMLREGGQPVRVTLRD

>CYP1138B1(Xaut_0387)*Xanthobacter autotrophicus*

MSAATEGPATGCNGTASPTPLLDRLAALRPAERWPLARDFIANEARAFFAELRAYAPIYETSEVTLIAKRADV
IEVLSLPKVFTVDLYKPKMGDFMLAMDETIVNYRDKSVMRAILAWDDLPKIRAVAAEVADAALDAAGCEIEAV
QQLARHVPMRIVQRCFGIHGPDQDLLDWSYANQMDQFNNLPYDDRPDADAIHQAAEDSRVKLRNLFAQLIPAR
IAEIKAGTAPDDSLTRILSLNLPAADHFGMDRVVINVGGLLIGAIETTAEAVINALAELFARPEALAGARAAA
EAGDDAAFDGYVWEALRFSPIVAFMFREAASTITIGQGTDREKVIGTGTVVLPLSLSAMFDPDFVPDPEAFRP
DRPFHTYLHFGYGHHECLGRYVGGVMIPEIVKRVLKRPAARPLAPVDMAGTPFPQNYRIGLE

>CYP1138B2(Mrad2831_5959)*Methylobacterium radiotolerans*

MRAAAPETPCLTFLEALPVSERWQRARDWIARAPRPFFAQLRARRTALDCGAAILVAGRAEVEEILSLPQVFS
VALYKPKMGEFMLALDGTEVNYRDKAVMRAVLSWRDLPAIRDLAGAVTDAALDAGEGSIDVVASLSRLVPLRI
VQRFFGFAAPDADLLRWSYANQFDQFNNLPFDARPDAEAIHAAADRARQEMRAAFARIIPARVAAIEAGADLP
DDVLTRLLRLHLPAAAGFGMDRVVINVGGLLIGAIETTSEAVVNALAELLGRPQVLEAARAAARTGPAAFDGY
VWEALRFAPIVAFMFRQAETDHVLGRGSPAESRIAKGRIVLPLSLSAMFDATGVPEPDRFDPTRPDQTYLHFG
RGHHECLGRYVAGAMIPEIVRRILLRDAVRAEGAVEDGGTPFPTAFRISYAGRSAGAAAPG

>CYP1141A1(MGMAQ_0954)*Magnetospira* sp. QH-2

MFSDLPAFRRDTLGFFLDKCKNATAPLEKLHVGPHPIYLVADPDLVKPVFNAPESEIDKGRLIYKMREVLGRS
SLTLNGDEHKERRAAIHARLAKGMTTTYVPEISAIVRRYTMMAAQQGSFDAHGIGAPLALRVICDVLFGREAL
SRGDEAALVNTIKLVEDDVAEKIFRVFPDLPWVAKRKKEKLRQGREIMMQVVERARQKASDASILQGLLELNL
DDEGLRDEILLIILAGHHTTGSAAAWIFYYMALYPDLVENLAKEAADIGDETGEIIPEKLYRDAPVSRAFANE
VLRLFPSSYWMSRETMADVELGGMPVKAGTSLIICPWAFGRDPKFWDDPEEFRLDRSYANRAFVPFGAGPRVC
VGGQLAMVELQLMALEFASAFEFTGVTPNPAPLPKPSLTLVPPRMEVTVKPRAHRAEPLISTHAA

>CYP1145A1(PSE_0379)*Pseudovibrio* sp. FO-BEG1

MPEPILYRPPAPTPCKPLRGLRLFRVFWARNVLNLIPEEFFNSPMERVYFTRRPCFLVNAPDLVNEVLVEKRL
SFPKSDVMVDTLKPVIGESTIIASGPKWEEQREMIAPVFSHVGVRSAFKHIQDACNEFLAYLDKAAEFGKSIS
LRDEMSRLTADIIFRTIFSHPIDSMRARKVFTQAMQYQAITTTWARKMILRGTGEGPNRPMPKEAEILRDEIR
LLLTDLVDEHTADRFGQHDDMCQRLLDARHPQTGLPFSKEQLVDHIATFFLAGHETTSAVLNWAFFILSQRPD
YSERIRRETDSITNGEMLEYKHLSKLRFTRNIFRETTRLYPPIAFLSRMAVEETTIGGHKIPRGALIIISPWI
VHRHRSYWHHPDHFDPDRFERDDDPMTGAYMPFGIGPRVCAGASFSTVEATSVLAGVTRHYNFKAEKPEEVFP
ETGIALRSQSEIRCKLIKRSHMTH

>CYP1155B1(BG023_112535)*Porphyrobacter* sp. LM 6

MGNVAAFGRLAPATDKKSAAKDPGSFDPTLLQSSGITARIVEWVVRHPFALMAFARHFWPIPTFKGWAIITRF
DDVAEALQNDKAIAVPFGEQIKTLNGGPNFVLGMADGPEYQALHKATMAVFPRADNAAIVGPMAYHEASNLLE
LGEGKIDGVRGLLTLVPTRICEKYYGLTIPDEPNFARWTIAMSSYMFGNPNNDPNLEKAALAAGALVRPIMDD
AIAKAKAAPDADTIAARLVQKQMEDPSAMPDEIIRAILIGMVTGFVPTNTMASGHMLDLLLDNPDWMAQAQEA
ARSDDDDLLQRCLFEAMRFWPLNPGPFRVAAEDVTIAAGTRRAKTIKKGTKLLVSTQSAMFDPRRVEHPMRFD
PNRKPVDYMLMGFGLHWCIGAPLAYAQITQTFKPLLKRRNVRRAPGKDGKMTRFGPFPESLWVLYDH

>CYP1170A1(HYPMC_4126) *Hyphomicrobium* sp. MC1

METTPSHTLGLGDESSIWFNLTPLLRGDPDNPMRILMQMMDRYGPVLPVNMANQRVVLISEPEYFKHVLVTKA
DNYIKYFDGLKPIFGKSMITNDGALWQKIRMPQQPAFHPDMFAEYIPYFLRAIDTKMALWADLAKSGETVEMV
EQTWTLAADMICKALFDRDMPFNPHVVFKCVKTYTDVMNHRDIRLRKQAGEVFEMTDEDPAKAMEVWASVPPA
VMGADPREMRERTLLKMIEATVADPSVPEFDQQQAIDELKQYLWAGTETTALTLAWALYETSRRPEMAERIRQ
EGEQVYGDREPTAADYSGLAYTRAVIQETMRIYPPVWSLIRIAAAEDEIGGVKINPGDRVSIFSYGAHHNPKF
WPEPESFQPERWMAGNAKKQVKYSYLPFGAGKRSCIGGAMSQVENTLALSRLLRRFRPEYVGKDPAGLNATVT
LTPKGGLKFKIHELS

>CYP1170A2(GL4_2417)*Methyloceanibacter caenitepidi* BAQ17853.1
Methyloceanibacter caenitepidi

MADMTVAYSPSHGLGEEDSIWFKLSALLNQEDPMQVLMALCEKYKGVLPVNLKNQRIVLLSEPEHAERVLVTN
ADNYTKYFDGLRPVFGRSMITIDGALWQKIRVPQQAAFHPKMFEEYFPYLMSAIDSKMDRWQKLADSGETVEM
VEETWTMAADMVCRALFDREMPFNPHFVFKQVKTYTDVVNHKNVRLKNVRGELEEITAEDPAKAMEIWHSVPD
TVISANVIDHREKTLLTAMQEAEADPDFPEFDHRQVIDEMKQYLWAGTETTALTLAWSLYLLSQHPEAMQRVR
EEAHRVCGDADPDWNQVQQLSYTRMVIQETMRLYPPIWGLIRIAAGDDEIAGHKINAGDKVSILTYIAHHSPK
YWEEPEKFDPERFAPERAKKRRKYSYLPFAAGKRACIGGALSQIENTLALVQLLRRFTPEYVGPTPAKIRATV
TLCPQGGLPFKIRKLS

>CYP1170A3(Hden_2852)*Hyphomicrobium denitrificans* ATCC 51888

METTPSHTIGLGEESSIWFKLTPLLRGDPENPMRILMHMMDRYGPVLPVNMANQRVVLISEPEYFKHVLVTKV
DNYVKYFDGLKPIFGKSMITNDGVLWQKIRMPQQPAFHPDMFAEYIPYFLKAIDTKMALWGDLAKSGETVEMV
EQTWTLAADMICKALFDRNMPFNPHVVFKCVKTYTDVMNHRDIRLRKQAGEVFEMTEENAAKAMEVWASVPPA
VIGAHPREERERTLLKMIQDAVDDPSVPEFDAAQAVDELKQYLWAGTETTALTLAWALYETSRRPEAAERIRR
EGEEVYGDREPTAADYSGLVYTRAVIQETMRIYPPIWGLIRVAVGEDEIGGVKINPGDRVTLFAYAAHHNPKF
WPEPESFQPERWMAGNAKKQVKYSYIPFGGGKRSCIGGAMSQVENTLALSLLLRRFRPEYVGTDPARLNATVT
LTPKGGLPFKIRELG

>CYP1170A3(HYPDE_37808)*Hyphomicrobium denitrificans* 1NES1

METTPSHTIGLGEESSIWFKLTPLLRGDPENPMRILMQMMDRYGSVLPVNMAHQRVVLISEPEYFKHVLVTKV
DNYVKYFDGLKPIFGRSMITNDGVLWQKIRMPQQPAFHPDMFAEYIPYFLKAIESKMALWADLAKSGETVEMV
EQTWTLAADMICKALFDRDMPFNPHVVFKCVKTYTDVMNHRDIRLRKQAGEVFEMSEENAAKAMQVWGSVPPA
VIGADPREERERTLLKMIQAAVEDPSIPEFDAAQAIDELKQYLWAGTETTALTLAWALYETSRRPEAVERIRR
EGQEVYGDREPTAADYSGLAYTRAVIQETMRIYPPIWGLIRVAVGEDEIGGVKINPGDRVTLFAYAAHHNPKF
WPEPETFQPERWMAGNAKKHVKYSYIPFGGGRRSCIGGAMSQVENTLALSRLLRRFRPEYVGADPVRLNATVT
LTPKGGLPFKIRELS

>CYP1170A4(W911_06550)*Hyphomicrobium nitrativorans*

MTAESTAQQSGFGLGDESSVWFNLTPLLKDPDNPMRLLMQMAERYGPVIPVNMANQRIVLVTEPELFKHVLVT
KVDNYVKYFDGLKPIFGKSMITHDGALWQKIRMPQQPAFHPDAFAEYIPYFIEAIKSKMAHWAEFARTGETFE
MVEQTWTLAADMICKALFDRDMPFNPHFVFKCVKTYTDVSNHKDIRLKRQADALFEVGDEDTAKAVEAWWSVP
PALFAADPREEREKTLLKMIQNAMADPSIPEFDEQQAIDEIKQYLWAGTETTALTFAWALYLTSKNPEAAERI
RREGETVYGDREPTAADYSALQYTRAVIQETMRLYPPVWSLIRVATEPDVIGGKEIKPGDRIVLFSYGAHHNP
RFWKDPEEFIPERWMDKTQKQVKYSYVPFGAGKRSCIGGAMSQVENTLALSILLRRFRPEYVGLEPPGLNATV
TLTPRGGLLFRVQELS

>CYP1170A5(BN1229_v1_3930)*Filomicrobium* sp.W

MSMIESPSSQFGEESIWFKLSPLLKDPTNPMGMLMQMSERYGPVIPVNMARQRVVFISEPEYFKHVLVTKTDN
YVKYFDGLIPIFGKSMITLDGALWQKIRMPQQAAFHPDMFAEYIPYFNQAIQAKLQRWADLAKSGEEIEMVEQ
TWTLAADMICKALFDRDMPFNPHFIFKCVKTYTDVSSHKEIRVKKQHGELFEVAGADTAKAVESWWSVPPAVM
GADPREEREQTLLKMIEAAAADPEMPEFDHQQAIDELKQYLWAGTETTALALAWALYLVSKHPEAAERIRREG
EMVCGDREPTAHDYSALVYTRAVIQETMRMYPPVWSLIRTAAGPDEIDGKKIEAGDRIVLFSYGAHHNPRFWD
APEEFRPERWMGDAAKKRVKYSYIPFGAGKRSCIGGAMSQVENTLALSVLLRRFRPEYVGPEPAPLNATVTLT
PKGGLKFRIRELS

>CYP1170A5(BN1229_v1_3918)*Filomicrobium* sp. Y

MSMIESPSSQFGEESIWFKLSPLLKDPTNPMGMLMQMSERYGPVIPVNMARQRVVFISEPEYFKHVLVTKTDN
YVKYFDGLIPIFGKSMITLDGALWQKIRMPQQAAFHPDMFAEYIPYFNQAIQAKLQRWADLAKSGEEIEMVEQ
TWTLAADMICKALFDRDMPFNPHFIFKCVKTYTDVSSHKEIRVKKQHGELFEVAGADTAKAVESWWSVPPAVM
GADPREEREQTLLKMIEAAAADPEMPEFDHQQAIDELKQYLWAGTETTALALAWALYLVSKHPEAAERIRREG
EMVCGDREPTAHDYSALVYTRAVIQETMRMYPPVWSLIRTAAGPDEIDGKKIEAGDRIVLFSYGAHHNPRFWD
APEEFRPERWMGDAAKKRVKYSYIPFGAGKRSCIGGAMSQVENTLALSVLLRRFRPEYVGPEPAPLNATVTLT
PKGGLKFRIRELS

>CYP1171A1(Jann_2670)*Jannaschia* sp. (strain_CCS1) ABD55587.1 Jannaschia sp. CCS1

MLRKWARFMVYKLNDPAFLENPADQLARMRAEGPLVRMKIPLLGTMWATTTDEATRKLLKSPDLFRRDPGPIT
GRSLAQKFWWLPRVIKPMLHTMIVTDDPAHARQRRLVEIAFARTSIEDMRPRIEAIAHRLLDALPQTGPVDIV
AHYTRQLPFLAICELLGIPESAHAALTRRIAPLSAVSNPITAVYAMARLGGVQRDFHAMFARARAEPPGPGLI
SALVHTDDNGAKLSEEELLSLTLLLFLAGHETTVHLINAGIVALAGDAALTRHFINTPDTRHLFVEEIMRSTT
PVTLTKPMFAAQDTDVLGANIRKGEMVAALLIAANHDPDRVDAPQELRPERRPNAHLGFGFGPHVCLGMQLAR
VEAVTALTALFARHPDLRLARPPGWLNRAGFRAPGRVVLDLRR

>CYP1173A1(TM49_07375)*Martelella endophytica* AJY45550.1 Martelella endophytica

MPMFSSSINVPVVAACDLEADAHETFAWYRRSFPFIALDTGGYVVLRHGDVARLMHDPRLQATEIAMPAQAGI
TQGALFDIFAHGMLTANGEAHARRRSAVSRALAGQVSEQFRQHMRRAAQGLIEDCYARGRLELVSGYAEKLPV
MALASLLGVGDADLLAFMQYVYAMNAFFRPKPTADQVAAAEAAGSWIRDYLDGLLADAERGRSHGFLARYAEF
AGQERLSRLEALVQIVQLIIGGTESVRTALVAQIAHLMTNRDQWRAVCVDPTLVGKAVSEGLRFEPGIAGAIR
VSVAEIEIDGLTLPAGQLVLLSSLSALRDETVFDRPNVFDISRPNLELSRLVFGGGAHRCVADVMGRIELEEA
LLALVERLPWLRIDGLPAFHGHMFVRSVGECWVCWE

>CYP1174A1(BN69_1943)*Methylocystis* sp. SC2

MFHDPDFMPFAPNERPSWRSAKRNFLENWPPAAYRETYFTLDGFWPFVGRTHYLADPELIEDMLITRAESFTR
DFITIGALSSVINRDALFFVEGADWKWQRRALAPAFRHDNLLALTPTFVDCAKAQAEVLRASPHATPVDVMNA
TSNVTLAVIEKAVLGVTGAFDRGKFVAALQESFAAISWQRIFILLRLPTRLPFPGSSKINRDMRYLYDETARL
LAARRASGGRTQAIIDRLLAAKDPESGRSMTDAELIANLYGFLVAGHETSAVALGWSLWLLAKDQASQERVRE
EARAIAGDADISDDTIEKLQFTKQVIQESMRLFPPAAAIGRQPREDMMLGPHKISKNEPVYAALWALHRHEKL
WDAPNAFDPDRFAPDKAKTRHRCAYMPFGAGPRICIGMGFAMLEMVAILATLVREFRFTPVDGHRLELAPDFT
TRAKGGLPLHVRPL

>CYP1174A2(B1812_07240)*Methylocystisbryophila*

MFHDPDLMPFTPLEKPSLWAMRENFLVNYPRAAYREDRSVLPGFPPVTPKINLIMSPALIEEMLVTRAEEFSR
DRLTVSTLSGPINKESLFFAEGADWKWQRRAVSAAFRHENLLAQVETFAACAREQAALWRRLEAGRPVDVAPA
MSRVTFEVILRSVLGAAPSFDRGRFVAELAPALGAIGWRRICAIIGLPPDLIPHPGSFRAAAALRYLSQATRA
LIEERRASGVARNDLLQLLLSARDPETGRVMTDAELEGNLYTFLVAGHETSAVAMSWSLWLLAKDQATQERLR
EEVRAVAGEEEIGPATVEKLVFTRQVLQESMRLFPPAAGFARQPRERTTLGPYHFSKGETIAVLIWCLHRHEK
LWDDPHGFDPDRFAPEKVKARHRYAYIPFGAGPRICLGMSFAMLEMTTLLATFVREFRFSTAPGHRLVIDARF
TTRAKGGMPLLIEPIRPTAEGELAPAA

>CYP1175A1(BN69_1113)*Methylocystis* sp. SC2

MFETINSSPFYPPFVKPAARPLRFPANLIALLNNNLEAIPEQAYFETLVVAPGPPRMAFFTGPDLVKELLVTR
PNDFPKGKLQVEVLRPMFGDAMISSEGGDWRWQRSVAAPLFRHEEILQFGSIMSDAAAGIVAQWRAAAPGVTH
PIHRQMMRAAFHVISNTMLVGGARQMLDAIEVGHADYYRGANWWVAYKLLGLPHWLPRPGGRRMRAHERRLRE
AVSQIVRQRKDSAAADNDLLGRMLRARDPDTGQSITHELLVDNIVSFLVAGYDTTAFALTWTLYLLSQSPKWE
ARILEEVERVVGDAPVASIHVANLVVVQQVLNESLRLFPTAPIVIRDIVEDMEFNGTIVPAGSIGIIPIYAIH

RHRRIWEDPNRFDPSRFAPDSTKKPTRFEYLPFGAGPRICIGASFATIEATIMLATFVRAAHFELAQEQQVRP
AGRMFLFPKDGMPMRVALRKTAPHA

>CYP1181A1(PSE_2866)*Pseudovibrio* sp. (strain_FO-BEG1)_AEV37374.1

MENKISLYRPPAPYPGEAAEGWRNFSNYLSSDPLGVIPKELYECAIFQFDDDDPRKFLVNEPKLLSRVLEEQQ
AIFPKGGGIAELLKPILHNGLVSAEGVDWQDQHEMVAPIFSNSSVRISFGYLQEALSSFVERLLTYSETGQTI
KLAEEINFLVADVNFRCVFSKPIEIQLEHQCFNSIMKYEEEVAGTALDLKRFSGGGQPAFTEAALKTAENVRA
VISQIIDEHMYNKPPNTQNFFQIFQEARVPSTGKRFTKSQLVDQVASIFLSSHETTSAALCWSVYILAQCPEI
VERIRTEVQLVTACGRISYEHLPRLRFTRDVFKEVLRLYPPVLYLSRIAREDTTLGDTLIPKESMVIISPWVV
HRHRSFWENPDMFDPDRFSRGEAKATLGSYFPFGLGPRMCTGASYVTFLGIKVIARLCSMFDFETVNTDEVRP
DSTLYLKPSRPVLCKVHKRKFSK

>CYP1182A1(PSE_0230)*Pseudovibrio* sp. (strain_FO-BEG1) AEV39812.1

MDTNNSLYRPPAPHPYTEGTGDFDGVETNLLKHLPEELYKADVVDISTSDRTNYLVNDPDLISVIFEEKPEIF
VKAEGMIELLRPLLGDHPIVTSNNRCLDQRDMIRPIGTMRTQQVNFEHIQDSIGRFSDYCEEIQNENRVIDLS
LLLRRLNADIAYRMLFSNELPKDVFKEINSHARDFELRISQLIRETAEQGVAWQLPKLPQTAMYAAQQLRYLV
SMLIDGNIQSNSFSHHSFLHKFLIARSPETNIPFTRKQLIDHVTTILNSQELAAAVLMWAFFVLSQCPEAKQK
IREEVRKVAGHDDIHFEQLSRLRYTRNVFREILRLYPPIPHLTRRASRPLKLGKLDLQEGTQITVSPWIVHRH
KQYWFNPDRFDPERYMRKDQHIIMKSFLPFGRGLRMCTGASSAILQGTLILARIVSKFDIDILNADKVEPEGV
LFLEPTRPIVCKVTPV

>CYP1186A1(SLG_20150)*Sphingobium* sp. SYK-6 BAK66690.1 Sphingobium sp.
SYK-6

MARDIPDYPLDIFTHDAVRNARDVDDALREFAPAVRLADGTVMIGRHAHVAPGLMDWKSFSSTSRPWHDPASL
RPEILLTDDPPRHTRVRRVIGDALSPRTLDAMKTVFTAGAQALVAELKARDGKEIDAVEDVTRRFVYTVFPDA
LGLPPGDRTHMHGFSNMVWATMGPENALFREAMVEDFGPVITWLNEVCDREVLNPDGFGMLLFRAADEEKITL
DEAKLLLQTILSAGADTTYITMANALRAWAEFPQEFAKVQADPKLVRNAFDESLRWDSPSRMAGRITTVDVPV
DDMVVPAGTRVGLMFAAANRDPRFWDDPLDYRIGRDLRHSLGFGYGIHACVGRTLAYMEADALLGALAQELDS
IELTGPVEPWMTTVGHGPARVPVRLHFR

>CYP1186A2(WYH_02150)*Altererythrobacter atlanticus*

MTSPIPEFPADIFTPEAVRNAREVDDELREFAPVVRLRDGTVMLGRHEHVAAGLLDWKAFSNTSRPWHDPGSP
RPEILLTDDPPRHTQVRKVVADALSPRALERVKAIFQQAAREFVDELRARQGEVVDGVGDITQAYIFRVLPDI
LGLPQEGRHHMHGFSQMVWATMGPPGELYDEAMQNDFSGVIAWLERTCEREALDLEGIGAEFFRAADRGQVTL
DEAKLLLQTVLSAGTDTTYLTMANAIRAWASFPDEYAKLRADPAKLRGAFDESLRWDSPSRMAGRITMRDVAI
EDYVIPSGTRCGLMFGAANRDPRFWDAPGEYRIERDTKHSLGWGYGVHGCVGRVLAQMEAQALLGAIVREVES
FELAGDYEPWMTTVGHGPIRLPVRLNFA

>CYP1187A1(SKP52_13590)*Sphingopyxis fribergensis*

MTSTVQDADPAQLDFANPQVMECPYPIYESLREKAPVYFAEKLGFWLITRYDDCLNAIRDPQVFSSKMGFRPG
SVPDEVMRIYNEEGFGDLPDTLVSNDPPSHTRYRKLVDRAFTAGRVRQMEDYMVVVVRELIDAFIEKGRLSVM
QDFAVPVPMYVIADQLGVPRSHRDRFKEWSDSAVAPLGLLLSDERKIECAKSAVEMQHYFVEVFADRRANPRD
DMISDLVTKEIDGAPLSIPELLSILNQLLVAGNETTTSAIAASIVRLANEPELVELLAKEPGKCDNFAEEILR
HESPVQGLFRMTTADVEVAGVTIPKGSLVNLRYGSANRDERKYECPEVFNVARANASSHLAFGAGIHHCIGAQ
LARREIAIAARELTARLKDIRLVEADKISHAPSVILRALNEFEIEFSKR

>CYP1204A3(Ga0102493_111100)*Erythrobacter litoralis* DSM 8509

MERELENASGSRAAPPRRLEDVDLFAPGAQEHWYDAYAILHEHAPVQRLPGEGLTPESDAFVLTKYEDIKRVV
RDWERFPPTLSLLVAQILESGEMPAHIPDIDAMVASIVSLRPTPELWRAHRKELTDPWVGPGCTRHAAMIERH
VDDLLGPMIDRARAGETIDFVAEFARPLPQRVMASVLGFPQVDIPELEEWGNAQVMSYVHGRTHRNLLTPEQT
AEKFRLLDGMKDYVAEKTREKRANPKEDMISFLTQTRYEALGRTLTDDEVGGIVYAMVIGGLETTQYAIAEQA

QLACEREGLFDAIRADRTAIRTFIEEGMRLRSPTQGLSTRICARDEVFQGVRVPAGSMLHLRWAAANIDPDEF
ENPLEVKLDRRAATRHLAFSQGPRSCPGSNLSRLEQTIAWNRLCDTLSGIAYAPGNSFAHQGGIMLGIRELWL
DLTPA

>CYP1204A4(BG023_11284)*Porphyrobacter* sp. LM 6

MGTEGAPRSLEEVDLFAPGAQEHWYDAYAILHAESPVQRLAGEGLTPGTDAFVLTKYDDIRRVVMDWDRFPPT
LSLLVAHIQQSGEMPTHIPDIDAMVASIVSLRPDPELWRAHRKELTDPWVGPGCTRHAAMITGHVDDLIAGML
AKARAGEPVDFVADFARPLPQRVMASVLGFPQEDIPRLEQWGNAQVMSYVIGTTHKNILTPEQSAEKFRLLAG
MKEYVAEQTAKKRARPQDDMVSFLTQVEYQALGRKLTDDEINGVVYAMVIGGLETTQYALAEQAQLLCERPGM
FGTLRGDRGAIRTFIEEGMRLRSPTQGLSTRICAHDEVFQGVHVPAGSMLHLRWAAANIDEEEFEDPLDLKLN
RKAATRHLAFSQGPRSCPGSNISRLEQMIAWDKLADAFADMEYAAGNDFRHQGGIMLGIFRLLLNLTPA

>CYP1206B1(HNE_2797)*Hyphomonas neptunium* ATCC 15444

MPDIASLTSLESLGFTGGRDSFRAFCARVFADDAPRLLRTEAGELVVFRHGDLRAFGALPEVGALPPGVMFPG
LHQLPPGAPLPPGGGIGGVISHQVFTANPPIHMPVRLTLLRQLSPKQAALLEPVARNVVQGLLDTLKHRDEID
LVEDIAERLTARFWGALIGMTETEMAEAALAVRDMTAMFLIQMTRGDLQTADTATATYGRLVETAALRSLAAG
THPFVTELAADLAKIEAQDDPDEAGIVPPNVGKLLAGNLVDGFHTAAIGAVNTLFALLQHPDTFTEVLAAPEK
LPAVIMEALRLEPPVLFLRRYMLRDLDYAGIHIPKGTQVLMLWAAGNHDPAVFPKPQIFRLDRDHRGITTFGG
GAHICPGRHVALMLIRLLLEEIAARGLRLSFTDAPYAWLGNHAMSQLPHMRFRVG

>CYP1221B1(HYPMC_2525)*Hyphomicrobium* sp. MC1

MSTSIATSHTAKLHKGLPPGPKGTLIGGNLSQIGPRRVDFFLKLAQTYGPIASFRAGRWRLFLVSDPELIQQV
LVTDARSYIKHFGARTFKPVLGNGLVTSEGDFWLTQRRLLQPAFLKTQVLSYAPVMAELTEAMLAKWVPGTSV
DIAFEFSALTSAIALKTLFGLDDHGDRVRVDESLRLTFELLTHRLDSLFQVPLVFPTPANLRLKRAIADVNAV
IDGFIAQKQAASPGNDLLSTMIAAQREDGTQMTAQQLRDEAMTLYLAGHETTALTLTWSWYLLSQHPDVEQKL
VAEWQRVLGGRAARASDLSALTYTAAVINEAMRLYPPVYVIGREATTDLELGGYRVKKGYTILMSQWVNHRDP
KYFPEPERFLPERWLDGLASRLPKFAYYPFGGGQRLCIGVHFALMEAAIVLATVGQQFKFTLAPDAVIDIMPQ
ITMPPKYGMPATLMRR

>CYP1221B2(HYPDE_29763)*Hyphomicrobium denitrificans* 1NES1

MSKAVIVSRHGLPPGPKGSLIGGNLGQVGPRRVDFFLDLARTYGPLASFRIGRWRLFLASDPDLIHQVLVTDA
KCYIKHFGARTFKPVLGNGLVTSEGDFWLRQRRLLQPAFLKAQVQSYAPVMANLAEAMLAKWHTGKSVNLEFE
FSSLTSAIALKTLFGLDDQGDRERIDESLRQVFDLLTARLDMPFQWPLWLPTPTNIRLNRALTDVRHVVDGFI
AAGRARPRGSDLLSTMIAAQHDDGTGMSDQQLRDEAMTLYLAGHETTALTLTWSWYLLSQHPAIEKKLVEEWQ
RVLSGRAPTPSDLTALPYTAAVINEAMRLYPPVYVIGREATTDLELGGYRVKRGYTVLMSQWVNHRDPKYFAE
PERFSPERWLNGLAARLPKFVYYPFGGGQRICIGSHFALMEAAIILSTVGQKYKFTLSPDAVIDIKPQITLPP
KYGMPATLERR

>CYP1229A2(M446_3515)*Methylobacterium* sp. 4-46

MAAPDPAPADTAPVARAALEADPHGMLARLRAAVPFIRTDDGVVMLLRAADIQALVTDPRTRQVETEFAMLRG
ITEGPLYEFFRLTALLSNGEVHRRRRAPMARSFAFQAVAALRPRVRALAEDLVAARAARGRMNLVADYASPLP
ARAIAAILGLPERDVPDFTRLVYAMARTLSSAYRPEDRPEAEAAACRLTDYVDRLVAERRRAPRDDLISAFVP
LIDAGDAEAAAETRMQIVSLILAGSDTTRAALAIQIGLLLSHPAQWQAVCRDEALIPGAVAEALRYEPAVGSY
PRFTLEAIPLDGGVLPAGALVSFSTLSALRDPALCARPEVFDIRRSDHPRWHLVFGGGAHRCLGEALARMELE
EGMRALTARLPGLRLDAPLTVRGSAGIRRVEEVPVSWPA

>CYP1229B1(Mnod_4829)*Methylobacterium nodulans*

MERQVRRSDDDPRTAEQPDAIPILSVAELEADPHGVFRHYRPRMPFVAHEAGGFLVLRSRNVEQLMRDPRARA
TETAYPEMRGIHDGALFDLFEHGMLTANGTVHRRRRSPFTRTFAARLIEDLRPRIRATAEALVGSWMDEGEVD
LVEHYTALIPARTIAGLLGLPQADIPHFTRLAYEVSRVLSFAFGPEDIPDLQAAAGELKDYVARLIEERRRAP
GDDFLSRYRAEAEEASDLSPLEIIAQIVQLIIGGTDTTRVAGAMQVALLLQHREQWEAVCRDPALIPRAVTEA

LRFEPSAASSARLTQEDIALDGHVLPAGAVIILSTMSAMRDEQVYARPDVFDIHRTDHPRLHPVFGGGAHRCI
GEALARAELEEGLAVLARHLPQLRLTGAMPKLQGHSGIRRIDRMCVTSGTPRMLRATLDAGWPQTMRLDK

>CYP1229B2(Mrad2831_2099)*Methylobacterium radiotolerans*

MNATEDLTRQAGPPGSPADVPTLTVAELEADPHGVFRAWRPRLPFVGHEIAGVLILRARDVDPLMRDPRAQAT
ETLYPESRAIPEGALFDIFAHGMLTANGAVHRRRRSPFTRTFAARLIEGLRPRIRVAAEGLVRDWLGDGEVDL
VARYTAQIPAQAIADILGLPRDDIPRFTHLAYEVSRVLSFTFGPEDIPDLEAAAVALQDYVAAILAARRSAPR
DDFLSRFLAEAEAAGELTPQEVVIQLVQMIIGGTDTTRVAGAMQVALLLQHPEQWAAVSRDPELIPAAVAESL
RYEPSAGSAGRVAREDIPLDGHVVPAGSLMLLSTLSAMRDETVYARPDTFDIRRTDLPRLHPVFGGGAHRCIG
EALARIELEEGLGAVLRLAPRLRLTGAMPVLRGHSGIRRIDALRVAA

>CYP1229C1(BSQ44_01320)*Mesorhizobium* sp. B7

MTATATRKRPTKVDPDDFRPDPHAGFARHRPLSGVLQIGAGFPTVVRYADIMALMTDTHTRQLETEALVHRGI
TKGSLFDFYANSLLLSNAPAHARRRAPLSRAFAFRLIQDLRPHIRALVGGMLDEIGTRGETDFLAAFASPLPA
RLIADILGIPREEAPDFAAKVYTMTRGLGGFRDEDFPAIEAAAADLVRYVENLLEERRRAPREDFLSSYLARV
DEDGTLSAIETLMQIVSVIVGGSDTTRFGLTSTVSQLLQRRAQWEALTADPALAPGAVLEGLRYEPPVGAIGR
VVTVPVEIDGVRLEPGTVVNLSILSGQRDETVFAEPDVFDIGRQDHPRWSLSFGHGVHRCVGEALARAEMEEA
LIVLSQRLPELRLAGDPPIAKGHTGIRGITAMPVAWG

>CYP1246A3(BSY19_2521)*Bosea* sp. RAC05

MTESRATHLTTACPHLEGASLCPAAGESTAPARPTGAPDYEPPRPRPLGPLSALLRAAWRGDGDLLSLLPKEA
YEVDVSPLGYSRRSILIVNAPELTRTVLTNPTEIYPKNDLMVGALRPLVGDSIFVSDGAPWRRQRRMIDPAFS
HIRLTRAFGAMTAALDDCEARLASAARSGESFSIDLSMSQLTADIICRTVFSTSLDTDVARDVFTSFAHFERR
VAHVEIRRLIFDRAFKDIPQKADVLESCRLIRAHMGTLLDSHLAAGASFDDICSAVIAARDAETGAAFTREEL
IDQLGVFFLAGHETTASALTWAMFIAAERPEVAQRIRAEVEDVAGTGEIGFEAVKRLTYTRNVFKESMRLYPP
ITFIPRVAAEATTIGRYKVKRGAMIMIAPWTIHRHEKLWRDPHRFDPDRFLPEREHEMVPGAYLPFGFGPRIC
VGAAFATLEATLILARLFRRFDVHPLEPGKVEPVARLTTRPKHEIMAQARLRAG

>CYP1246A4(AEM38_14625)*Hyphomonadaceae bacterium* UKL13-1

MGPLASLVRVIRQGDGDLLSLMPADAYLLPMGPLGYSRRSIQIVNDPALTKPILADPEDIFPKNDLMVGALTP
LVGDSMFVSSGSLWRQQRRMIDPAFSHIRLAKAFPSMVGALDDFEVRLQAAAASGQEMSLDLAMGQLTADIIC
RTVFSASLANDTARDVFESFAFFERSVAHVELVRLIFGKPFDEIPQRADVLEACARIRGHIGRLLDTHLEAGA
QFDDIAAAVIAARDLETDTRFTREELIDQLGVFFLAGHETTASALTWAFFIAAMQPAVADKVQAEAVAVAGSG
PIEFDHVKRLTYARNVFKEALRLYPPITFIPRVAARATMIGSKKIKRGTMVMIAPWAVHRHQSLWPSPDVFDP
DRFLPEREHEILPGSYIPFGAGPRVCVGAAFATTEATLILARILRTYRIEVQQPELVRPVARLTTRPAQEVMM
KVSLRT

>CYP1250A3(BSY18_805)*Blastomonas* sp. RAC04

MRIDPANPVAPTNIVRRFGMDQVPDHVPPELVRQAGLIYSPEFLADPYTFFPAMHERFPPIFYDVGPFGNAWV
LTKHEDALFALRHAEYFSNEDATPFPRDPNNYFYFIPIEIDPPEHRKYRNIVDPVVSPQGVLKLEERIRALAN
ELIDEVIEKGECEFDEVFGRPLPVLVFLDLMGLPRDMCDTFVSWAMALLHSNDRKIMGDTLKTIGDYLTTAIA
EKEANPDDGLVSRIVHAEFDGKRISDKEAFGFVTFLFIAGLDTVFATLNNIWLWLARNPERRQEIIDNPDNIN
AQVEELLRVFSVTFSGRTVAQDVEVRGVQMKKGDKVTSILPACNYDPDVFPNPTVVDFNRPKKIILAFTVGIH
SCMGAHLARLEIKIALQEWLKRIPDFKVRPGAETVYRPGGVIGPESVPLVW

>CYP1258A1(Mex_2p0017)*Methylobacterium extorquens* AM1

MFIKVSQVSGLELNKPTHIEVEGIDLVLLGTGSKISLFESRCPHQGAFLHEGAVRNGALVCRAHGWAFDADTG
VCRGKQGIALTSIPLRIEGDDILADAEAVRALADRRKTEGRSSETVASVIQSQRTINDLPGPAGLPVLGNAHQ
INLEQLHVTMENWHEQYGSFFKFRILRQNAIAVAHPHLIRMVLRERPHNFRRISSIESVFQEMHAQGLFSVEK
EEWRYQRRIVAQALNARQIRLFFPKMQQITERLMRRWSRASEDGRVVDVQEDLMRYTVDVTSNLSFNYDMNTV
EKEGDVIQDHLEHVFPMISRRVNSAVPYWRYFKFQRDRDLDKALVALKGIIDDIIDKSRDRLKQNGGNVEAGN
LVEAMLQLQETEGAITNDAIFGNVFSLLIAGEDTTANTMVWILHFLSSRPEVFKRMQEEADEVLGGSDVLPDL

ETANRLIYHDAVINEALRLKPVASIWPMEACSDVVMDGVAVPAGTPVFLLTRIPTLVDENGVPAPEFDPERWL
RKDASTTHFDNVMVPFGAGPRLCPGRSLSLLEIRAVMSMVSRRFELELPTDGPSVGERFAFTLIPTNVRLRFK
ERNKYPAGPQFIAPTAESVRLGA

>CYP1275B1(HNE_1343)*Hyphomonas neptunium* ATCC15444

MPAAPALQKLTVGGQAFLRDPHARLTCALEAGPLAQTRLLVFGRITAVLSHAGITEMMKDAGRFTVDARNAGH
SSALGMPFLPKSLKLLAENLLTLDEPHHTRLRRLADAPFRRAAIEGMRPAVEAQCARLLDEMARSGNTDLVSG
LCRPLPIQVICDLLGLSDARREGLVRVFSGFSGAGTLEAIGVLMKTGWVQSELRQEFAEVRAVPRPGLITELV
HAEAEDGRLTEDELLSMVFILFAAGHETTTHFISSSVWTMLTQPGAREAIAAMDGEALNVAVDELMRFSGPVQ
FTKPRYAREDTEFLGQPIKRGKPVMALLAAGNLDPAAFDDPLTLNLGRRPNRHLSWGWGPHLCLGMHLAKAEC
EIALRQILERWPGVEIDGDPAALKWSQRPGVRGLARLPLQLKS

>CYP1281A1(Sphch_2114)*Sphingobium chlorophenolicum*

MSARSMAELVESGELTLKDEEIVKCPYEAYDYLLEERPVYYDAGGDFYVVTRYDDLRAALKDTTMFNSDGASA
KHRANVNPERNKKIEDLFLEQGWPRERPIGNYEGAEHKDRRMIFESFLRASKAREYDDVIEQIANSLVDGFVE
DGHAEIVSQYSEMLSIKTICHLLNAPDDAIPIVKRSMDAMLANIGGIMTDEEAFENARREIDAQRYFKAMIDR
LREAPEDTLLSALVNAKLPSGEMTDAQLLRHVMLDMFMAGAETSAKAITSGVLILARNPQLRAEIAADIKGKL
QPFIEEVLRLEGPASQATRIAVKDVELHGVAIPKGSMVIFRLAAANRDSRKFGCPANLDIGRKNAAQHLSFGS
GPHACVGAPLARRELWYGFKALLDRMEDIALAPDATVNYLPNMVFRGIDALKITFRPRADRPTPQ

>CYP1291B1(HYPMC_1141)*Hyphomicrobium* sp. MC1

MTPALYPPTVTPPAQPLPLLPFLRRFIRNPLRAIPQAVYEEPVVTYGKKRPLLAWVTNPALIENILVKNAEQF
PKTRLDLRVLRPIVGDGLLTAQNESWRWQRKIASPPFRHTELLTYVPTMVDAADECIAQWRQRGSAPFTTDVE
AAMTDATFAVVARTVLAGINTAEGDRIKASSRSYLDPISWEVAASMLQYPETYWHPGKARMRRSADELRAIMR
RLVAHRRSGGASGNDLVARMLAAKDPDTGEPMSDDMLADNLATFLFAGHETTAKALTWTLYLLARAPEWQDRL
RDEISAVAGSAPITPETVTKLPTVSRVLKEAMRLYPPAPVMTRVTAVEMDIGGKTLPKSTLIVMPIFIIHRHR
ALWEDPNRFDPDRFLPEKEARYARTQFMPFGYGQRICIGSSFAILEATAILATLLRHARFEWDGKFAPEPVSR
VTLRPKGGMPLTVRPL

>CYP1291B2(Hden_0645)*Hyphomicrobium denitrificans* ATCC 51888

MVGVRDVNTIPPPEKLYPPTVQPPERALPLPRFIARFIRNPLQALPRAVYTEPVVTYGSKRPLVTWVTDPALI
ERILLKDVEHFPKTPLDRRVLTPMLGNGILTAEGESWRWQRKIASPMFRYAEILAYVPAMVEATEQLLDTWKQ
RGHAFTTDVEDAMTETTFSVIARTVLAGIDETEASAVKHAARTYLDRISWEVAAAILRLPPTMWHPGKANMRT
SAKEVRTIVERLLAQRRAQPGSGNDLVARLISARDPATGEQMSDATIVDNLATFLFAGHETTAKALTWTLYLL
ARAPQWQDRLRDEVRHALRTSQRVSPGTIERLPLTLRVLKESLRLYPPAPVMTRLANQDLDLAGTHVPRGSLI
VIPIFVLHRHQRLWDDPGRFDPDRFLPENEAKYPRTQFMPFGFGPRICIGSSFALIEATAILATLLQGARFEW
DGRHAPEPVSRVTLRPKGGMPLIVKPL

>CYP1291B3(HYPDE_35128)*Hyphomicrobium denitrificans* 1NES1

MNALPTSETFYPPTVQPPDRALSLPRFFARFIRNPLQALPRSVYSEPIVAYGKKRPLVTWVTDPELIERILLR
DVERFPKTPLDRRVLTPMLGNGILTAEGDSWRWQRKIASPMFRYAELLSYVPTMVEAAEQLLETWKPRGQKFT
TDVEEAMTETTFSVIARTVLAGIDENEAGAVKRSARIYLDRISWEVAAAILHLPATMWHPGKANMRSSAREVR
AIVQHLLAQCRKARGPNGDLVARMIAARHPTTGEQMSDTAIVDNLTTFLFAGHETTAKALTWTLYLLARSPQW
QDRLRHEVQHAILSSQRVGPGTIERLPLTLRVLKEALRLYPPAPVMTRLAKEDTDLAGTHVSRGSLIVIPIFV
LHRHRRLWDDPDRFDPDRFLPENEAKYPRTQFMPFGYGPRICVGSSFALIEATAILATLLQSARFEWDGRHVP
EPISRVTLRPKGGMPLIVKAL

>CYP1291C1(BN1229_v1_2138)*Filomicrobium* sp. W

MPTRLTPNTDVQFVPATSDGNGLYPPSITPPDQPLGFFDFLRQFIANPLRGLPRQVYEEDLTLYRPTKKLTVL
WVSGPAIVEELLVGKANLLVKSPLEKQVFAATLGDGVLTSDGALWRWQRRVMAPLFRHNEILSYVPTMAQVAE
EQVTEWRRAFAGSQAIDEQMMTSTFKVIMRTMLVGGDRSEGEAIMRGGSEYLARVSWEMAHALLHLPTWLPHP
ASRQMRRASQTVRSALSDIITRRRQSAVEGTDLLARLLNARDPDGGEPMTQDQLVNNLSTLLEAGHETTAKAL

TWTLYLLARAPEWQQSVRDEVNRIVGPTSIRAEHIDKLVLTTRVLKEAMRLYPPVPVVARQPKEAFELAGTPV
PANAQIVVPIFAIHRHRKLWKDPDRFDPDRFLPDRETEMARTQFMPFGAGPRICIGQSFAMIEAVTLLATFVR
GARFDWDGEHLPEPISRITLRPKGGMPLTVTPIL

>CYP1291C1(BN1229_v1_2138)*Filomicrobium* sp. Y

MPTRLTPNTDVQFVPATSDGNGLYPPSITPPDQPLGFFDFLRQFIANPLRGLPRQVYEEDLTLYRPTKKLTVL
WVSGPAIVEELLVGKANLLVKSPLEKQVFAATLGDGVLTSDGALWRWQRRVMAPLFRHNEILSYVPTMAQVAE
EQVTEWRRAFAGSQAIDEQMMTSTFKVIMRTMLVGGDRSEGEAIMRGGSEYLARVSWEMAHALLHLPTWLPHP
ASRQMRRASQTVRSALSDIITRRRQSAVEGTDLLARLLNARDPDGGEPMTQDQLVNNLSTLLEAGHETTAKAL
TWTLYLLARAPEWQQSVRDEVNRIVGPTSIRAEHIDKLVLTTRVLKEAMRLYPPVPVVARQPKEAFELAGTPV
PANAQIVVPIFAIHRHRKLWKDPDRFDPDRFLPDRETEMARTQFMPFGAGPRICIGQSFAMIEAVTLLATFVR
GARFDWDGEHLPEPISRITLRPKGGMPLTVTPIL

>CYP1302A1(LH19_08305)*Sphingopyxis macrogoltabida* 203

MTESSAQPVRTPVHEFDYIADPGVLADCHARYWELKETAPPVFWTNAHGGHWVCNTGSSVQNVVRHPEIFSSR
YLSIPPNPDQPKMIPEMLDPPEHRPYRQMLRPFFESKAIEPLEDRVTAWTDQLLDGVTGKGECEFVEAIGSRL
PVAVFMELFGFPMEKFDEFRHLVTGFFHSQASNEVRNNLAQQIVGHLAELIQQRMAEPRDDMISKIIVSEVDG
RKLGFEELMSIGFLMFLAGLDTVTNAMSFGMRHLAHDDTLRQRAIDDPSVIPNLVEELLRRYAFVATPRYVVE
DTELEGVKLFAGDCILAPLPLVGWDEGLTEDPKTVLVERQFYRHAAFGSGIHTCLGLHLARMELIVFYRAWFG
RIGHFRQVTKGDETCRGGSVMALEHLHLAWDA

>CYP1302A2(Sala_0872)*Sphingopyxis alaskensis*

MSESPVQPARSPVFEFDYIADPGILADCHARYWELKETAPPVFWTNAHGGHWVCNTGASVQHVVRHPEIFSSR
YLSIPPNPNQPKMIPEMLDPPEHRPYRQMLRPFFESKAIEPLEGRVAEWTDRLLSEVADAGECEFVAAVASRL
PVAVFMELFGFPMERFDEFRHLVVDFFGARASPEERNLLAQRILGHIAELIQARMAAPEDDMISKIIVSEVDG
RRLQFDELMSIGFLMFLAGLDTVTNAMSFGMRHLAHDSALRQRAIDDPGVIPDLVEELLRRYAFVATPRYIVQ
DTELEGAQLRAGDCILAPLPLVGWDEALNPDPATVSVERQFYRHAAFGSGIHTCLGLHLARMELIIFYRAWFS
RIGHFRQVETGDESCRGGSVMALEHLHLAWDA

>CYP1312A1(K663_17821)*Sphingobium* sp. MI1205

MTLSDLAPVPPHVDPALVRDFDIYNDMGLLTDPHARYVELLRDAPSFFWTPRNGGHWVACGYQTVSKVYRDFR
VFGNDNLGIPKPPVMPARKAFIPSQLDPPEHAAYRRLVDTLFTPARLAAMEEDVRALCNSLIDEFIDKGRCEF
VSQFALPLPVLIFLNRMGIPSDRYEELAGWVQEHNSGKTAEARLAALDRIASFMRAILDRAIDGHHDDWVSEL
LQMRLGDQLLPRDDVVLPMAITIFFGGLDTVKNALAHIALVLASRPDLQTMIARREVNLANAVEELLRFRGLV
NQQRIVRQDTAFEGAQIRVGEMVHICNGTSGLDPKANACPMAIDLERTGIKHNTFGQGRHFCAGAPLARLELR
IFLEEWFARIPQFRLANDKIERSTGLINTIESLHLAWKD

>CYP1326B1(TMO_0531)*Tistrella mobilis* KA081020-065

MTVDYDPRDPQIRNNPFPSFRRLQDEDPVHWNPHLRAWVITRYADVRKVALSRRMSPDRLTPFYDSLPEARRG
VVGELIRYLNLWMVFRDPPEHTRLRRLMNEAFTPKAVERLRPNVERIVGRLLDDLLPRGEMDLVRDFAYPLPA
TVIMDMLGVPHDQLDRFKDWSDDLALFLGSARDVPDKYERARRGALEMSSYFREVIADRRRRPGEDVLSALIA
VGTPEERLTEDELIAAAMLFLFAGHETTTNLISNGIYSMRMHADAWARLVADPSPELLATAVEECLRYDGPSG
GIARVVRVTHEMEGRELKEGDRVFAMLNAANRDPRVFEAPDDFVIDRSPNRHLTFGQGIHFCLGAPLARLEAE
IACREIARRIPDLTLGTTAPDWHDSLIMRGITSLPVHFRPVKSAAA

>CYP1326B2(RPD_0041)*Rhodopseudomonas palustris* BisB5

MPNLDFADPKINADPFPVFAQLRESDPVHWSPVLKAWVITRYDDVRRVAVSNADMSAERLAPFFATVPAESQS
GFANLMTYLGKWMVFRDPPEHTRLRRLFTKAFTSRSVMALEPNVGEIVALLFDEMEQKARSTGVVDWIADFAY
PLPATVMMDLLGVPRDDLHRVKDWSNDIALFIGTSRATADKYLRAEAGAKAMAEYFRGIIASRTVDPQDDIIS
QLVTEPDKREALTDDEVIATCILLLFAGHETTTNLLGNGFYYTMNAPEQWARVKDDPSLAETAVEEWLRYDGP

SGALVRVVTADVEFGGRTMLQGQRVFAFINSANRDPEQFGDADRLDLGRSPNPHLTFGHGIHFCLGAQLARLE
GQIALRALIERFPGISLATDSAPGWRDSIILRGMQSLPIRLR

>CYP1330A2(K663_17521)*Sphingobium* sp. MI1205

MSKATDTADAPLSFADPAVQRCPFSSYDRLREERPVYHDPVTGNYVLTRYEDVRKALLNVKALRNRTGINSTR
SDPTANRIYEEKGWLPIDTLVSNDPPDHRIYRTLVDKVFTPPRVAALEPRIKEIIDELIDNFVDAPEIDFLEA
FAVRLPMYVIAEQLGVSRAHMDRFKLWSDVSVESVSPVLTSEREVEIAEILTEMQRYLAAEIESVRQTPNDTL
ISHLANTDTDGRLLEMRELLSIIHQLLVAGNETTTTLASGMKLFIEQPALAEEIRQDPARAKPFVEEVLRTL
APIQTLFRRVAEDVEIAGVTIPAGSLVEVRYGAANRDPKQYAEPAKVDLDRTNAASHLAFGAGPHLCIGNQLA
RGELRLAFQMLTRRLTNFRATRGDDSYSWMTSYIAHGPDQLWMAFDRL

>CYP1337A2(K663_20558)*Sphingobium* sp. MI1205

MEITQDDLGTYASGEVQRCPFPFLNRLLEEAPVYRDPGTGMYIVSRYDDIHYVNSHPEIFSSKTPIMINRQTS
VSEEVAKRYRERGWPEEHVLAFADPPEHGLHRALVDKVFTPSYVKKLEPYALKLVDELIDGFIEQGESDLGSG
FAVHLPMYIISDQLGVAREDYEQFKLWSTAWVDRNDPNCPPDRELMLTDRMIDMQNYLAARAREYEAAPNDNM
LSRLVHAEVNGEKLTMGQLLMIAQLILVAGNETTTGITSAMYMILTDPALKARLLGDPTMIPAVIEEMLRAH
APVPHQYRYTTQDTELQGVSIPKESVVQVSYLAGNYDKAKWECPEKIDIDRKGVRNHLAFGRGIHFCVGNQLA
RMEMRVAIARLLERLQNIQISDKHPAPKFMEHFQIHALESLNVTFTPGPRTAG

>CYP1349A2(Ga0102493_111520)*Erythrobacter litoralis* DSM 8509

MKPRAAGFMRAPWEAYAAMREEAGPLWTEPETGVVFVLDYALAEEVMRNHQRFSSFADRAAMRKGGLPAEVLE
IKAQGWPLALTMVQNDAPDHDDYRKLVGPFFLPRRLKLMEPFVAGRIGELLDAIDDKGGRSDFFPDFAVPLPV
SVIGEYLGMRHLGDDTVKRWSDAFADEIGFLTSDERAVEIAKLTLECHRAMVALCDGRRRGEGKDIITALANA
TRPDGERLGNPELLSILTQLMVAGNETTTSTLGFALLRLARDPALYARLSAEPEKIAPFLEEVLRLDSPIQGQ
FRKAVGDQQLGGHTIPDGTMLHVRFGAANRDPRVWGAGAGDVQLDRRPPKPHMAFGNGIHFCVGAALSRLEMR
VALGEILSRYRSVRLACAEEDLPFRTNFHQRGMTSLPLEFER

>CYP1350A2(K663_18056)*Sphingobium* sp. MI1205

MDGTVPHAFYERDNLANPFPLMAQMRAEAPVERTIDPAGREIFVVTDVRLIEEVAKRTGDFSNRFGHLLMAGG
GADQEVASILASEPLEATLLLTSDDPEHQRYRALVNAAFATGRVSHMEDRIGELIDELIDDFIEDGRVDFVDQ
FAVLLPTYIIADILGLPRDKYDKVKLWSDAVITVVGRMGTREQEIDAARLMVEFRRYVKQTVRERRQEPRDDL
ISDLVTARVDGVSPLTDDEAAALAFETGVAGNETTRNTLMSGMVQLLRRPDQMQALIDDPKLVGNAVEEILRY
ETPASSMWRIARHDTKLAGVSIPAGATLLLRYDGGNRDPQRFEEPDSFDIRRKNARLHIAFGAPSMHRCLGQM
LARKELTMAFPRLLARMKNIRIAEGSVTDYQPSLLFHTIGSLNLSFDPGPRLTKRAG

>CYP1376B1(IE4771_PB00087)*Rhizobium etli* bv. mimosa IE4771

MSFLQEYDSISTNDPSAQAAFVIKWLRGNPTAMFDELRADRPIFINPVFTMVVRATDVLDILSQHQLFSVRLN
AKSMDPAVGPFMLARDETELNWHEKSIMRAVLRHDDLPRLKAFVGSIVTSELSTSEGGLDVVARISRLVPLRI
VQSFFGFAAPDELMLKWSFATQHAMFRNLGGDPVVERACIDAGVEMRNWLWPFLAAKWADPGLSDDTPVDRLI
RLSRNCDLAIATDRVVSNVCGLLVGAIETMSQAIVQSLEQLLQRNDVLPAAIKAAKDLGSDDIHSYALEALRF
NPITAMQIRYAERDCQLGVGTPYATKITRGSRIAACTGPAMFDEGLFREASAFKVDRPRESYLHLGFGHHECL
GKYVALVAIPETVRHLLSMPGIRRAAGDSGKIDFAGGPFPEHFSIEWDQAKLQ

>CYP1376C1(Maq22A_c15690)*Methylobacterium aquaticum*

MTFLDNFGSIDPENGPARTAMFFRHLRADWRGLFAELRAERPILDLAPFAVVSRWSDVMDALSRPSTFQVTYR
PHMDPSVGPFMLARDDSELNWQDKAVMRSLLRWDDLPAIRDFVGRTVAAALAVEGDTVDVVGLASRLAPLRVV
QQSFGFYGPDDAAMLRWSRATQADMFHNLTGDPAVKADNVAAGTEMRAWIRRFLARRQPWAEARGEDAVSRLL
RLAGTGGAGFDAERVVSNIAGLLVGAVETTSQAIANATEQILLRPDVRAEAVEAARSPDTDRFDAIVWEALRF
NPMTTFVLRIAAEPARLAPGTEHEVAVPPGRVVALAIGSAMFDPALFPDPESFKPRARLDYLHMGFGQHRCLG
QYVGYAIIPETVRQILRLPGVRLLGGEAGRIDDRGGPFAERFVLGITP

>CYP1384A1(TMO_1336)*Tistrella mobilis* KA081020-065

MTVIGQGNKAGNGGQAEPIDFGSAAFRTDPWPAYASARRDRPVWWSDRLGQLCVVRHADVMRVLTDPVFTVEY
PFKRTRQAFGETLLDIDGARHLAQRRAAQHLLGFEGVAAAVAGLAPGIIGATISGLPAGRPVDAVAALAEPIA
RGVIRGLFGIDAETGDRLARHLSRVSRYIETADVDLGDVVEDRRALEAEIGRLIDEGLSPRPAALALRVQRMA
SELERTQLMRLMLLMMAAGVETPLSAIANALAVLLDHRHLIDRLAVEPSLADSFALEVMRFQPPQHDTTRFVR
GHTMLGGIEVRRGTSVRVFLASANRDESVFRAADRFDPERFLPGRPPVPVLSFGAGRHACPGRPLASAIIAEA
LRRLAGGFQDIRPAEDCDPTITGEGFRRPQRLTLVLDPRRRPATASLPGAAVA

>CYP1396A1(Mesop_5506)*Mesorhizobium opportunistum*

MFRTKYSDGVKNAREAQKPVAELPPFDPERLRSKGLLAGLLSRVLENPRGWLALLRRFWPTVVIGRIALVTKA
ADVRDILERQEFETPFGRDMAEMAGGVNFILGMQDGSDYRRMKSVILSAFPPVEVEARVRPIARLHSRALART
AMPGFNAIELLKIVPVRICRDYFGLAFDNDEEFADWSIALSSQLFFNPFEKAATRELASVAAYRMRDVINQSI
ETVLAGSKTDKPLARLVAIHEAGRLSLDDIRSIMMGMVSSFAPTNLLAGGNALDVVLNRTDARKAVQDAIDSG
NDAALDKAILEAMRFKPIYLGPLRYVARDAVIASGTRREKTLRAGTIVIPSILSAMFDKDAVIDPERFDTSRP
GRDYLLYGHGLHLCIGAEVARVQIAESFRALFAKAGLQRAAGSIGRLTRRGTYPERLCVEFDVADAWKTSLDL
SQYDPSISSLCELIIRFLIARSSTHSDRISLSRLCGITGKRAPDVDLMTAITILTQSRQPVLSVAYYSIDKYG
RFEKVNRKDINTESFIDDIDKQPLMFKDTFPIFYASDEFRKVNIGVKRT

>CYP1405B1(Mesop_2125)*Mesorhizobium opportunistum*

MIRLKYFDAVRAGQENQRPLAEMPPFDLERLRSKGIVSRIIGVLFNDPRWMLALLRRFWPNLAIGNFLLVTRN
ADVREILERGDEFETPYGPEMAELARGSNFILGMQDGADYRRMKSSVLGAFPPGEVEALVRPIAARHSQEIMM
RASPGFDAITDLIKIVPVRICRDYFGLEIDDESEFADWAIALSALFFSDPTASPTTRQLAVVAGDRLIRVIDR
SIDAIRERKGAKDRLPLARLVDLLDQDRLSLPDIHSIMLGMIAGFVPTNVLAGGNCLDVILSRADARQAIDAA
LAKNDTGSLDRAILEAMRFKPIWIGPWRYTARDATIAMGTRRERLVKAGTVVMPATLSAMFDPEAVQSPNHFD
TSRPHRDYLVFGYGIHQCIGAEIARIQIGESLRALFQKNNVRRARGKAGRMTHIGAYPDSLKVDFERPALCRT
VIHSMATVVCPITRPASLDAIREEVAGFGNPASDEIRAALNATGIIHFASLAVVPTGEVGNAPADEKGALVLE
VSGDGSTEDVINAVAHAIGDRLRPILKDVCDLPDGLVLDAFLRKHNVEISPSFGSNAGLVFSGTPGHSVQRIL
AEAELADAVRLLVEQPCATKGGAAAALDEARRHVRSLEKFDWAFGPAESLLERPPGKWWQAITTTLLAPAVLA
AVALLVLAFWWMTYVLVFGDPPGLTLGTIAIAGTALLLAVLGILALAAVILGLCFLALRRLEDIDVPHSTPVD
LGDLDKILVREDQPGQAQNHLTAISTMKVGILRRLALRLSFYLISISAQKVFRPGFLATINTIHFARWVLLPG
TNRLMFFSNYGGSWESYLEDFIAKAAAGLTGVWSNTEGYPRTRWLFLDGARDGDRFKRWARRQQVPTLFWYSA
YPALNTLRIRANSRIRHGIATATGSEARDWLSLFGSLPRPQARKADAGPAAPDPLPVEQLETGEIQSLFFGPF
GSLGHAHMLAIAIPENLSRGRRRAWLEFIVEQTSFGDGVPAGRAMSVAFGPDGLRRLGLDGSVDDNPLDTFPV
AFRHGMGNPERSRILDDLGESVPQKWQWGSPQKPVDAVVVCYAESPERLKVEVGTAKAKLTAAGMDLVSELPL
ATKRDGKRAVEHFGFVDGVSQPIVRGTARANVGAAPMHLVAPGEFLFGYRDEHGFYPSSPSVEASLDRTGILS
AVRRNRQIPGLPPPRRDFGRNGTFLVMRQFEQHVDTFNAYCKAAAVQAASETDDPAITPRWVAAKMLGRWPDG
SSLVRNPDGRPGRGADNDFAFGTEDPQGHRCPLGAHVRRSNPRDSLGEDRETQIRIGKRHRIIRVGRSYEKQD
KKGGKVEKGLLFMCLNADIERQYEFIQQTWVSSTSFQGLVAEKDPTIGSRDGNGRFTIPSWEKVTVLKGIPQF
VTTRGGGYFFMPSRSALRFLISRL

>CYP1406B1(RPB_0862)*Rhodopseudomonas palustris* HaA2

MFTLKYYDAIKKAQSDTASAPPAPILPFNLDDLGADTTLKRWTSWGFGWLMRGALHVFREVWPNPQFGRLIIV
TRETDVRDVLAQPGLYEVPYGPEMTELAGGTNFVLGLEGPEHDRQNAIIRSVLRPADLDRIRELSAHYTTILL
DASGGRIDVMKDLMTRVATETCCGYFGLEPEDPDAFAEWAMSISALLFADPFGDAATRRLALNGAAQVREVID
RAIARAKAAPETDTVVGRLVAQAHDGVVTEDEIRAIIVGLVTGFIPTNTLAAGKMLDELLRRPKVWAEAIACA
GRDDVAGLQAILLEAGRLNPALAPGQWRYATQDGVIAHNTSRQRRVKAGSVLMVATMSALRDKRAFVAPGSFR
ADRPNDSGLMFGDGAHVCLGKHVAITQITQVFRGLLQQPNLRTASGKDGAIGWVGPFPRRLDMEFESRVAPQT
QNMVVICAPVRPGADLDTLRTQITALGNPARPELVAAFEATGIVHFASMTLIDAGTPEQPAPHLLLELNVDGT
PDGAIRAVAEVAGQWLAPIFAQADAPAGAALIDILRDNTLDLQTRPWGAIGLNFNGTPEFAVGDIIRQRELAQ
FAQDALEDYLENHACLGSRAMVALGYVRKLIRQDPALKRTIDESPDSPRRARLQALFARGAAFTQYLIRPSRR
RLKISDWVPRSGTDSLLSLLGSPTFQWIGAIVAALVLIAGQAIYFAIEPFSDATYLGRIALALVGGILLVALI
LAALGGLFLLVLNDYESRDVPDDSDPDLGKVREIAASENHPGFIQNHITAVTTLKPGWFRKLTLALSLWGIKE
LVTHWYRPGFVLNMGTIHKAKWFRPPGTDKLIFLANYDGSWESYLEDFVMKAHAGQSAAWSNGVGFPRTRFLI
FDGAQDGDRFKRWVRRQQVPTQFWFNRYPQLTTDDIRRNAMIHDGLVRASTDSAARAWLDCFGSMTRPNYAIE
TPEVQSLVFRAMGQLDHTATALLRLPADRGAGKEWLRAIMPEAGLLQDPQAPRPAIGAITFGDRPFVGGDAAH

NVATFVAFSASGLGKLGLSARNANDGLTTFPTAFNIGMSQRANILRDTGASKPERWDWVDAALEGSDGAAAAD
ATLFVYGKSAEVCRKALDAHAALLGGRDALLYVVETSPPTVDTPDGPKTSLEYEHFGFVDGISQPVIKGTQRF
AKGVPARDIVEPGEFILGYRNNQGYFPPSATVASSSDPANHLPILPDLLPSRFPNFRADTPAKPVRDFGRNGT
FLAIRQFVQDVDGFKAFTEAKAQELSKYRDLAAVIGETPTAEWVAAKMMGRWRNGVPLVDKPNSTTFNTRRGK
SRSDARDPYDRDNDFAYGQDDPQGLHCPFGAHIRRANPRDSLQPDDPTQQQLTARHRLLRRGRSFEAGQGSGG
PGRGGKPEKGLLFVAVCADVERQFELVQQSWVSSPSFHGLSHEPDPIIASAPDDPAKKRVFTIPTAAGPLTLH
GIHSYVTVTGGGYFFMPSRSALQYLIDLE

>CYP1406B2(RPD_0969)*Rhodopseudomonas palustris* BisB5

MFTLKYYDAIKKAQSDAASAPPPPLVPFDLDDLGADTTLKRWSTTAFSYVLRGALYLFREFWPNPQFGRLVIV
TRETDVREVLAQPGVFEVPYGPEMTELAGGTNFVLGLEGPEHDRQNAIIRSVLRPTDLDRIKSLARHYAQILI
DGSGGRIDVMKDLMTRVATETCCGYFGLAPEDPDAFAEWAMSISALLFADPFGNAATRRLALNGAAQVREVID
RAIARAKAAPETDTVVGRLVAQSSDGAATEGEIRAILVGLVTGFIPTNTLAAGKILEELLRRPKVWAEAIDCA
GRDDSAGLEAILLEAGRLNPALAPGQWRYATKDGVIAHNTSRQRKVRAGSVLMVATMSALRDKRAFVSAGSFR
ADRPNQSSLMFGDGVHACLGMHVAIAQITEVFRVLLRQPNLRRASDRSGAIGWVGPFPRRLDMEFEPKIAPQT
QNMIVICAPVRPDTDLDALRAQITALGNPARPDVVAALQATGLIHFASMTLIDAGTPDQPAPHLLLELNVDGS
PESAIRAVVNEAGEWLAPIFAHADERAGAALGDILRRNMLDLQTKPWGAIGLNFNGTPEFAVADIVRQRELAR
FTQDALEAYLENHAGLGSRAMVALGYVRKLIRQDPALKRIIDQSPDSTRKARLQALFTRGAAFTDYLIRPSRR
RLQISDWVPRSGAESLLAMFNSTTFRWIGAIVIGLVLIASQAIYFAIEPYSDATYIGRIALAVVGGLLLVALK
LAALAGLFLLVLRYYENGDVPDDSDPDIARVREIAASENHPGFVQNHITAVTALKPGAFRKLTLALSLWGIKE
LVTNFYRPGFVLNMGTIHKAKWFRPPGADKLIFLANYDGSWESYLEDFVMKAHAGQSAAWSNGVGFPRTRFLI
YDGAQDGDRFKRWVRRQQVPTQFWFNRYPRLTTDEIRRNALIHDGLVRASTDSAAQAWIDCFGSMTRPVDAIE
TPEVQSLVFRGMGQLAYTATALLRLPADKAASKTWLRAIMPEPGLLPDSSGAAPRPAVGAVTFGDRPFAGGDA
PHHVATFVAFSASGLARLGMSRSNANDGLTTFPTAFNIGMANRANILRDTGASAPERWDWVDAALDGRDDVAA
ADAALFVYGRSAEDCRAALDNHAALLGGSDALLYVVETRPATVETEDGPKTSLDYEHFGFVDGISQPVIRGTQ
RFAKGVAARDIVEPGEFILGYRNNQGYFPPSATVRSSSDPADHLPILPDALPGRFPKFCSDTPAKPVRDFGRN
GTFLAIRHFVQDVDGFRSFTEAKAAELGRYRDLAAVIGEEPTAEWVAAKMMGRWRNGVPLVDQPNSKTFNNRR
GPSRDDVDRAYDRDNDFSYGQDDPQGLHCPFGAHIRRANPRDSLQPDDPTQQRLTARHRLLRRGRSFESQQGD
AGRPEKGLLFVAVCADVERQFELVQQSWVSSPSFHGLSDEPDPIISATPDDPAEQRVFTIPTAAGPLTLHGIQ
SYVTVKGGGYFFMPSRSALQYLIDLE

>CYP1406C1(Saro_3798)*Novosphingobium aromaticivorans*

MFEPKYLRMQWLGARRKKERGSMDFSLDFMRPKGVAGQVAAALFRRLLVPVLFVLQCFWPVLRWRRFLLVTRA
DDVAAILGDPEGFPVPFGPEMQSLGAGATFLLGLEGPDHDRQRRIITSVVKRSDLAGLEAQADSFAKALLESS
KGRIDAQRDLVQRVAAETCARYFGLPIDDPDLYAEWTIACSQLLFADPLGDPVARDMAEAAAARIGALIDRTV
AATQSGGHEHAAPPETLVARLVELQAAGGADAPTNEEIRAILLGLSVGFVPTNSMAGGKILDLLSRKGEARRE
AIEAARAGDAQHFEQVLREAMRLAPAIAPGQWRWTRQDTQWIRPGGRTFRVRRNTLVMVATQIALRDPRKVVR
PWRFEYDRTDTPPLVFGNHAHSCIGEHIAIAQLRGSLMPLLAQDGIEDSLNRLRVKWLGAFPEHAWLQFDHDE
GAQKGQFIVIEAKAGTTAELNARIAEIDARLERLRPRLDQAGLLHFCSMTAIDAPTTRPPEERGDGRTTDTLL
VIEVNGDGEGTRAIAAFVRAMNAELRALLAAAGVEPGLDLVTYLLDKRLDLTSAPWGATALQFYGHQGLSARD
IAAQDELAGIASEALQATLQANIGLVASASATLREVRRRIVARPDGARWAAYMLKPSQARMDLSWWDDPLDGG
FVLRLLRTRAMKRVGLVLVAVALASAWAIGRSEGFTWGGLVSLPHLLWLGIAGVMVSLSGGALVAGGFVAMLR
LKESRDVPDGAAPSLDHLRECAAHEDKPDHVHNHILVVTPLKKGLIRRLALALALWGIAMIVTFRFRPGFVLN
MGTIHFARWVRLPGRDTMVFQSNYDGSWESYLEDFITRAHWGQSAAWSNGEGFPRTRFLISGGAEDGDNFKRY
VRRKQVLTRFWYARFRNLTSAAMRRNALIHDGLARARTESEARSWLALFGSGQLTRDRLESDEIQSLVFTGFG
KLRHSTALLIRFGEQADDNRKWLRNLTGFRNATPDKSLLFEMDEVRERRLGRVTFGEAERFGTAVSLGLSAEG
LRVLEMPASHPERGFNALPGPFAFGMTNRARRLGDDGEEAPQNWRWRDAEDGAVHAILLLYAADEEGLARLAA
LHRRFAERAGVSVVDEVPTTALPQNGHAYDHFGFRDGIVQPVIAGTAKAALNRVPSDIIPPGEMVLGYANAQG
YLTPGIPVDKADDPFDRLPEMPREPQRYPRFGGDEGARGPRDFGRNGAFLAVRQLEQHVVRFSEAMEAAANQI
HDNYPELPSLLGHDVDANWVAARLVGRWKNGAPLMRNPLEPDRKSPELPMLFGADDPSGLQCPLGAHVRRANP
RDSFEPGDPTELGIVNRHRLVRRGRSYERPASDGGAPEQGLLFMAVCEDLERQFEFVQRSWLDSPAFHDLDRE
NDPIVGRCPAGHKRSFRIPTSSGPIQVDGLPQFVTLRAGGYFFLPSRSAMLWLARV

>CYP1460C1(AZL_f00700)*Azospirillum*_sp. B510

MSVTSERAILPGPFAPPGVGHRWQGLQMLSDQIGFFVRMQRRYGEICSWAKNDQKFINIYGPRYNRLLLSDPE
TFVIDAFREHHLPVGSSFERLTLSLMRLNGNAHQRHRKMIQPAFRTYQLERYRQIIVDETDRFLAGWQPGESR
QLDEDFLKLVTLISMRTMFGMEAETDGERLVALIKQLIRQFSSPLILLLPFNVPGLPFRAILDTTDKIERFVM
DAIERKRADLDRYDDVLAEMMKARDETGSGFTNEELVAHAYTILCQEAAASALLWTFFLLDCHPDVYRRVAAE
IDEKLHGAPPGMNDLKELTYLDRVLKEALRLFPSSPFGLRYAVRDCTLGDHDLKKGTGIFFSSYVTHRMPEIF
ENPLAFDPDRWTSAACSPFEYLPFGAGAHHCIGQGLALVELKVILAMILQRFMVRLQPKTRIDLTVKISLVPK
QGLAAVLRRREAGHDGTVPEISGNVLSAVIVSGSTAIEGS

>CYP1515A1(SGRAN_3518)*Sphingopyxis granuli*

MMTDTNMDMVAGVGREDWERRTTSRTGAGRVADPYPKLAELRRQGPIHRGSTFDLFGVPDPMAEVWPDAPRFI
SVGYDVTEQILQDNIGFSNAGIRKMTEYVFGEVSLMGSDDPEHRKFRALVQPAFTRHGLDLWRGFVRPRLDHL
IEGFKAKGRTDLYFEYCAEFPVYVIAMVLGIRPQDLERFHEWAAMLQIAAATPDEARNARLAVEEYMRGIIED
RRRNPRDDIVSMLLEREIELDGVHQKISERHLLGLINNLLPAGAGTTYRSLGILLVTLLERPELLERLYHDRD
RIPRVIEELLRWNGPVLFAPPRLATRDMVVAGVEIPAGSIVEPAIGAANRDPARFEDPDSFIADRNPRVTLSF
STGVHYCAGSQVARMELTAALNALLDHLPHLRFDDSQPPPQITGLMYRMPTGVPAMWG

>CYP1515A1(Swit_1995)*Sphingomonas wittichii*

MSGSACPVAPYPAKELLSNAFARCPFAKLGALQAEAPVHQVENLPWYLVTRFDDCVEALRNPQVFSSEHRDFG
PALKAIGLTPTPQTHARMLEIGGDRGAMFDIVLHRDPPAHSRQRRLINKALTARVNLWERFIEDQVRTLLARF
GEEGEADFMTDFAVPLPIAVIADILGVAEEHHGKIKSWSDDSARASGRLSSDEEWLAMARSMREQGDFFASEL
RKRLAEPSDDLVGMLARATQEGPSPETGDEPLTFDEAVEMLVLLLVAGNETTTQLLGQVMMELATRPGLIARV
RADDSIANDLIEECLRIATPIATMMRFVKQDTEIGGLPIPKGSIVSFCFNQANRDPRMFEDGDGFRPDRDRVR
HHLSFGSGIHNCPGARLARLEARIAARAAVRHFKDLSLAGKDAARYDMASLAVRGMTGLRLRYVTESAEARAA
LSGPTPA

>CYP1591A2(Swit_2020)*Sphingomonas wittichii*

MDSKAQSGCPVAGIDIKELMSREFAACPYPKLAELQGEAPVHQVGDKPWYLVTRHDECLRVLMDPEHFSSEHD
DFGPALKAIGITPTPDQVERMQALGGNRSNMVDVIPHRDPPVHTRQRKVIIKAVTARFRRWEEFIEEQSARLL
ARFEGRTEIDLIAEAAAPLPIAVIADILGIPDDYLPKIKQWSDDSSQTSGRYASDEDWMALAKAMSEQRAMFA
SEVRKRLADPSDDLIGRIAAMTQEPVDEATGEGPLDFDEGVELLTLAMLGGNETTTQLIGAAMFYLATEPGLL
DRVRTQPDIIPGIVEESLRLTAPVFTMMRFCKQDAEIGGVPIPAGSIVSVCFNQANRDPKVFPDPDTFVAERT
NTRRHLSFGQGIHVCPGAQLARLETRIWIARLAERVKHMELAGPDAVRYDLASLAVRGMTSLRVRYELFDKQS
GQLGEAA

>CYP1597A2(MGMSRv2_1643)*Magnetospirillum gryphiswaldense*

MAETTTGPVTGHDWWAMLTDRAFLENPYADLRRLQQQGPIHLDEQSGIYFVLGHEAFGAIAKSPKIGRDTHYW
NPGWNNDEYRQRDPVGHELYQGVQPQMINVDGADHKRMRAVWEDAFKLPAVKDMAPMIEAEAARLVAELPDEG
EIDLIRDFAAPMPMRVLCNLLNLPSSMDDTIHGWSEALIRVADVMISPEDKQRALDAMKASKDFLRGFLAERR
AAGDASLTGIIIRAQDDGVVSEEETLTNMMSMLIAGHETTVTLIGNGMRLLLDNPEQMARLRADRSLVRSAVE
EFMRCEPGGNMILRVAREEVEICGTVIPAGAPILGMIGAVNRDPARFDNPDLFDIGRVGNAHFTFGGGPHVCL
GAPLARLEGLVAINALLDRWPSITLAGTPVWRTDRLNARGLATLPVKVGR

>CYP1371B1(REMIM1_PF00891)*Rhizobium etli*
MSDIVERARGCPVMSFDYAKIRPCGSYRQKAMELRDDGRPIWVNTFAQGFWVVTTQELVREVYLKADIFTTDS
VMAVEPETNPRHILLPLNVNPPHHRKYRNLLAPWFSQAKVKELEPLLRASARDLVEGFLPAGKVDAAWDFCAL
LPINSLLGAANMPMSVAPQLMQAINDFTRGFSGLEASETGGTAGMDAAVEAIHAVTDEMIAERRARPLDPKGD
FYTYLTTELIEGRLLTDDEIRQIGLIYVVAGMETIRAQLGWLLYHMAKVPEDRRRVLADPSLIPAAVEESIRY
YTVIWGVGRKIGQDVHWHGVDLKKGDMIYALNDAFNRDPKRFEDPDRFDLDRKMAPHLSFGFGAHSCIGMHLA
RTQLEVALEEFHRLIPDYAIEDSAVIEERGSEVTIQSLPLVWNAGA

>CYP1732A1(NGR_b14810)*Sinorhizobium fredii* NGR234

MEMASDASALTGANLYNDPYPVYARLRREAPVAFFAGTNEYFVTRYEDCRTVGANDRVFGPSGAPDRPEARVM
GMPNVLTMSGEEHACLREGIDLNLTQERVRSYVDRLTRPVVQRFIDEIRPKGAANLTTELFEPISVRCIGDVI

GLTETSNDNLVEWFHAMALGLQNVSNDQAVWDRLDAALADIDNQLGALYDAALSKPNHTLMSHVMHGGMPEGK
ARSLKEISPTMRVIILGGLQEPGHAAANACAGLLSNPEQAKVMAEDPSAHALKAFDEGLRWIAPIGVTPRVAA
QDFEIASTVIPAGSSVAIVMGSANRDEARFENADQFDMLRKKKQHVSFGFRPHFCSGHFLSRAMGEIALEEAF
RQLPNLRLDPEQEVKAKGWRFRGVNVLPAKWDA

>CYP1732A2(NGR_b16290)*Sinorhizobium fredii* NGR234

MANDASALTGANLYNDPYPVYARLRSEAPVALFEGTNEYFVTRYDDCRTVGATDRVFGPSGAADRPEARVMGM
PNVLTMSGEEHACLREGIDLNLTQERVRSYVERLTLPVVQRFLDEIKPKGEANLTTELFEPISVRCIGDVIGL
TETSNDNLVEWFHAMALGLQNVSNDQTVWDRLDVALADIDDQLGALYEAALSKPNSTLMSHVMHGGMPEGQVR
SLKEISPTMRVIILGGLQEPGHAAANACAGLLSNPEQAKVMAEDPSAHALRAFDEGLRWVAPIGVTPRVALED
FEIAGTTIPAGSSVAIVMGSANRDEARFENPDRFDMFRKKKQHVSFGFRPHFCSGHFLSRAMGEIALQEAFRQ
LPNLRLDPEQEIKAKGWRFRGVNVLPAKWDA

>CYP1732A3(RGR602_PC02305)*Rhizobium gallicum*

MTGSHTELTGENLYRDPYPVYSRLRSEQPVAFFEGTKEYFITRFDDCRTVGGNDKVFGPSGSTDRPEARVMGM
PNVLTMSGEDHQCLREGIDQNLTQERVRSFVEGLTRPVVQRYIDSIKKNGEANLTVELFEPISVRCIGDVIGL
TETPNETLVEWFHAMALGLQNVSNDAAIWKRLDDALAEIDRQMGDLYQACLTKPNNSLLSHVMYGGMQKGEVR
SWEEISPTMRVIILGGLQEPGHAAANACAGLLSSSDQAKIMAEQPQENAMRAFDEGLRWIAPIGVTPRVARED
FEIAGTVIPQGASVAIVMGSANRDATRFEDADKFDMFRKKKQHLSFGFRPHFCSGHFLSRAMGEIALAEVFRQ
LPELRLDPSREMKGKGWRFRGISDLPAKWNA

>CYP1733A1(SGRAN_0231)*Sphingopyxis granuli*

MLDTRTRPFDLTRSETFRDGHPFAVYDAIRAAEPVYRNPGDGRSPPIWVLTRHRDVEAVSRDTANFTSTQGFR
VFEQGGVAALKPHIREAITSTLLTMDPPDHTRIRRPMQAHFLPSALHRLEARIDSFVRDMVAALPANAEIEFV
TRVAAVVPIRTLCLLLDIPEADRARVFDWTNRLVGTSDPEYGASAEDSNAVFEEVFDYGTRLLERRSAEPGDD
LLSVVAQMFGGEEAGMRNVRRGMFTLLLAAGNETTRNSLSGSIVALSAAPDQRRMLAENPDLIARAIPELLRF
VTPVIQMMRVARSDVEVGGQPIAAGDRVAMLYGAANRDPEIFDDPHRLDIARPNAARHLSFGIGIHHCLGARI
AALQLNAILRALLGRFPEIGAVRDPAYLQSNFVCGIKHLAVHLGPERR

>CYP1733B1(SGRAN_0226)*Sphingopyxis granuli*

MTVAAAPSPAPSADAAAGATGRFDLVDPDCFRAGHPHAAYDALRRQGPVVPVEGADGERLWLATDHEAIRAIS
ADSAHFSTARGFRVHTAHRASMDPEIGKVLSRFMLSMDEPEHAPFRRLVTPFFMPSGIAAVEPLVRQGVDRLM
AGLAGRDRVEFVEEIGARVPISTICAIIGVPPEDEWRVFEFTNAVFGTDDPELAPSKAVANERYLAIFDYGWS
LLESRRREPRDDVISCIAHATLPDGRPLSRTEQVSYFSNLIAAGNETTRSSLSGAIWLLANHPDQRCALVDDP
ALIPRAVQEILRRFSPVIHMARTATQDVEVGPAQVVAGERVAMLYGAANHDPALFADPYALDVGRANANRHIA
FGYGIHHCLGSRLAMLQLALILEALLRRFPRYEVVSDPAYIRSNFVLAMKRLDISLSPGGR

>CYP1734A1(BSY18_3531)*Blastomonas* sp. RAC04

MEVRRGNPPRALRTQAPDVSTAPGASGLAPEVHFAQLRAQCPVALQAGSGTNGAGRAAWVLTRYDDIVAAAND
PKTFGQSQRFAGERRPPLESNPPEHRIWRRMLQPYFLPKAINQMEPFTRALARDLLAPILATGSGDAAHGVAR
PLPPQILLRWLGQPSEDWETIKLACEHAYFQGSPDPAERAAFEEAEALLWRYARDTVASRIERGTHEADDDPV
SAMLAAMPTEPAVTRPLIDGVVRLLLAAGHDSTTSALGMCIGFVAGDGDLQQSLRSDPTRIPAAIEEMLRLRT
PVLQMPRTVMADTVVGGRELAAGDSVLLAFASGNRDASAFENAECFDMGRRSGQHIAFGVGIHRCIGNILARQ
EIRVVLEELLAATHSFRPDGEIEHEFWHPYGLRRLPLSLVPR

>CYP1735A1(K663_17741)*Sphingobium* sp. MI1205

MTSTAEPIDPIDFTSVMQQGCPYQTYTRMHADGRKVYQDPGNGYYVVTDYAEVRKIAADTKRFSNKTGILVQR
DTPLQKQIDAMYAKDAWPQMHTLVTNDPPSHRMFRSLVDRLFTAQRVAQMETYIQMLVDEAIDRVIDTGEIDV
ARDLAGVFPAIIAGDFLGLPREDQEKLKDWTNASNALTEPTFDEENEIAMNRKVIELLNYMVERANVSAAAED
GSFMHHARTFEVDGRRLTDQELAWFLQPLYVGGHDSITLLIPTGMLRIIEQPGLEQRLREDPALITNFIEELM
RFDAPVQALWRRALQEVEIDGVVIPEGGIIQLQWAAANRDPDKFDHPDEFIVDRPNARQHLAFGTGAHLCIGN
QLARAEMRIAFETMLRRMRNFRLSDEEGAVQMRRHYMQWGAEKLKLRFDKIA

>CYP1736A1(Cseg_3016)*Caulobacter segnis*

MSHLPRRIEDLPSLGAAELAGWAADLEGSLARLFERYPDGLAQGAGSRLLVFGAEPLRRIAATEAAGNMPADV
LSSSAFNVGPDGAPVEPEISAASSLAKLVSNQVFTANPPLHGPTRQLFMRQFMPKQLPRFAALIEGAVTALLQ
EAGARGRVDFGTDVAERLTARFWAQVLGLTADEETRARHLISALSPMFYLVRTPEETLSADAAAAEYLAMVSA
AVERGLAQGDQPILRDMAADFSAIVEPGRPESLGMMLAANLIDGFHTAAVAVVNAVYGLACNPEVLAQVRADQ
SLVPAVFFETIRLNPPVVLTQRYALRDFEYDGLMVPADTPLVMLWIAGNRDPKAFAEPHRMDLSRPQRGDVTF
GGGAHICPGRNIARSLVEAVLRGVTAPGVQIEAVAAMDDWTPRSSMRQLSQSPMRVSVGI

>CYP1737A1(Swit_0359)*Sphingomonas wittichii*

MTGAAQARPRDEAVERELKTGADHAFDPLDPETVRNPGPAYSELAAKCPFYHYQGDEYQFYITSDYKEIRDKV
LSDNPVWSFKWGDGPIDWAEFSDFGILTDPPFHLEYIAALRKGMTPSRMKFYQPEVEAIAEELVAGLEARADK
SGNFHDLFALPLPARTMCFMLGADQAMYADYKRWADELQSLLFNDKNPAAEQSLIAEIMPHFMGLIEERKALL
RDAGIDDPTHEHWGTVLPFDYISLGLVSRVEGRRFNDQELFQICTAFLTGGQETTTSLLTNVVWRLLEKPELW
EQLKAHPELVENAIEESLRFDPPINSHFRTSLCPVTMHGAELPERSKLMFSMMGANRDPAIFEDPDTFRIDRP
LNQVKRHLSFGYGVHFCLGAPIARLEAQIGLRKLVERLPNLRLTGETERIDSWMYWGRRRLPVAWG

>CYP1737A2(Sphch_3675)*Sphingobium chlorophenolicum*

MTEITEAKAQEPEERRLKTAADHIFDPLDPETVRNPGPAYSALAGQCPFYHYKGEEYQFYITSDYKEIRDKIL
SDNPTWSFKWGDAPMDWETFSDFGILTDPPFHLEYIAALRKGMTPARMRYYLPEVEAIAEDLVSGLENRTDRQ
GNFHDLFCLPLPARTMCFMLGADQALYADYKRWADELQSLLFNDKNPSAEQTLIAEIQPHFMGLIEARKQMLR
DAGIDEPTHEHWGTVLPFDYISLGLVSRVEGRRFNDMELFQICTALLTGGQETTTSLLTNVIWRLLEVPERWE
RLKAEPHLVENVIEESLRFDPPINSHFRTSLCPVKMHGAELPERSKLMFSMMGANRDPDIFPDADSFIMDRPL
NQVKRHLSFGYGVHFCLGAPIARLEAQVGLRKLVERLPNLRLTGPTERINSWIYWGQRELPVAWG

>CYP1738A1(HNE_2953)*Hyphomonas neptunium* ATCC 15444

MADDSRPAGAVQSILELSAFNPAARDNPHPMLKTMREDCPVFRDEAAKVWLLSRYKDVRETVNDRSFVRHPTK
AEEGSLSARFVEERQERGTSILFLDDPDHARIRQPLAKAFYARINRMRPEIEAMIDAIIEKAPASGPFELMAE
IAVPVPVLVIARILGVDEARLDDFRQWSEEVILGLNPVRTPEEDEALIRGAYALDAYFAELMEARRKAPQDDL
VSDMVQLQGSGEAEISDGELRNNLEALLVGGNLTTTDLIGNGVWLFLTHPGQIRTFRDNPGLAAQAVEEVLRF
EAPVSITSRILPDDRDVGGCPMKARQPVWMSLAGANRDPEVFEEAETFDITRKRASHVAFGGGPHICIGAPLA
RIEARHVYLKLFERYPDLRLAEQELAWRTLPFFRGLEKLIVEG

>CYP1739A1(OV14_b1284)*Ensifer adhaerens* OV14

MSEPIPEALRGGAAAELFGRLVRLRDDEAHGKLKEAVTNALRSVDLGQVANLARMRAAELDNELGRPLDEAKI
MQFMFAMPVQVVAQLLGIPRERFGDAMAWLGDYGAAAAAAGTGIPAPTPELLARGHRGAQALFDLVSAIKNDD
EQRGSLLDALAREATHVGCDDEKDIIANAIGYMIQGYAGMASLIGLTLLALARRPALHAQVDADRTLLRPLIQ
EVVRCDTVTSSTFRFMARDGEIAGHKLRQGEMIIVLLAAANRDPALNPDPDRFDIARADRKYLEFGAGAHACP
ADKAASLIVEIAVDHLLAGGAPLERLETSLSYAASGHVRTPLFKR

>CYP1739A2(OV14_a0596)*Ensifer adhaerens* OV14

MRLRDDDIGNTLRRAVASALRGVDLGQLAVVAHRRAMELDGDAQEMFSVDRTMTFMFALPVQVVAQLLGIPKA
SFGDVMAWLGDYGAAAAAAATGIPALTAELLARGHHGAQALFELVMKIRRDSQQCGPLLHALANEARRVGCDD
ETDVVVNAIGYLVQGYVAMASLIGSTLLALARQPALRTQVVADPTLLRPLIQEVLRCDPVTNSTFRFMARDAE
IAGHRVRQGEMIIVLLAAANRDPALNPDPDRFDITRADRKFLEFGAGVHACPAEKFAPLIVEVAVAHLLRRGL
PFEQLESSLTYAASGHVRTPLFKR

>CYP1740A1(RHPLAN_40630)*Rhodoplanes* sp. Z2-YC6860

MQPTRLNHLESAWCLTRFLFDPLGTLARCHRERGRVVIITAPIRWPIKRRSRHSKPFIISGVGADFNRAVLSD
PTIWRTRSLGPGGPRGSAAREVSTGIFSLHDETHKHYRSLIVPPLSQRSISAQSSRMGDLAAAEVERWPIHET

IDLWAYSRRLIQTLSISLLFGNDRERGLPVAELVDHFYNHAFSWKVFLCPFNVRGAPYHDMLRDGRHLKVRLL
DWLKRKRGSADSNDLFAIVANNPDEKGRSLSDIETIGHIPPLMVAAFETCQNALMWTLVLLCQHPKVARDLLD
ELEGRLAGATPSVDRIGDLPLFDGVINESLRLFPPVPQQFRVAARDTTIGDIPVAQGTKAVLSPFLTNRDPDL
YPDPDCFKPERWANLNPSLYEFFRFSAGPYTCPGSNFGTSLLKVMVATILTRFRVALAPDIPIDYKVTLTLTP
QGRIPATLFPQDRAFAGSHVRGSIRKLVQLPN

>CYP1741A1(SJA_C2-04160)*Sphingobium japonicum*

MDDMIISAPPILEFDPYAEDVILDPAPFLESVRNAGVAYLPANEAYAVGGMDEAVQLSKDFDSFTSSHGIGLL
DITKPGLLRPTNALQECDPPQHSQLRKVVQKILSPSRVRGFQELLAERARILVDDLLEKGSVDGTRDIAQAFI
LDVFSSVAGIDLPAEPAIATSNMMLNLMGPVNAVSQRAMAAAEPHLPWFNEAITRRNAAPGGLVDLAYQAEEE
GLLPEGIGGNMAIVFVGGGFDSTIAGIANALRLLALHPAEWARLRSDPTLVPAAINEAIRFDPPFRVYYRMTT
RPVKLGAFDLAPQTKIGVWMGAVNRDPRHFDRPDEFDVSRRGAHAGLGFGTGPHNCVGQILAKAETEAIISAL
VRKVARLELTGTPQYEAHNQVRMPGKLPLRLHPG

>CYP1742A1(RCAP_rcc02437)*Rhodobacter capsulatus*

MVDFGSFNSLAPLLRDDPYPVYAQLRARAPVFWTETEQAWVLLRHQEVSAAFCNPGLLTLDLGQFVADVSQAL
GDHPVELLRLLDMAIFFRNPPAHGPLRALVARLLALRPQAACAAEVARIARSLHAPLVRDGGLDLMTGFADPL
PPLFMGWLLGLADADALWLAKTLSGVPVILNRGASIRDFRAAERRLTEAHAFLRAEITARRLAPRDDGLSLLV
SRNDASEAPFDDDRLTALTSFVFMAGFETTSALIGNALWLLLDHPDQCDRVAADRKLLASAAEEALRLEAPIQ
QVRRRAATDMTLAGQEIRAGQQIVLMIAAANRDPQAYPDPDLYLPGRTGRPVQSFGGGLHHCLGAWVARMEAE
IALGTVLDGPRPRLCIDRPGWRALHNQRRMTNLPVQV

>CYP1743A1(LH19_26540)*Sphingopyxis macrogoltabida* 203

MQQDLDVFRMVPLETDPPIHTKIRSVLTPFFVPSALDPLDDAIRKITVDLIDECVRNSPVDFATQFSIILPSR
VFFEIILGEDPKEIQWIVDLTQVLITDPATAEQGAPKLLQWCADTLEGRRKRGQTEDLIGVVAHMGNGPELFL
EERERIEILNLLILAGMETTANGISSVVHTFATNPDARAELRDADPQRIDKVVDEFLRYASPVTSAGRTLVSN
DESFGCPMQKGERVTLSWTAANHDPATFPNPDVLDLNRNASQHLAFGVGHHKCLGMHLAKREMRIAIQELRKL
KIFELVENTRIRYRSGPQQGIISLPIHCAR

>CYP1744A1(RHPLAN_11270)*Rhodoplanes* sp. Z2-YC6860

MSSPAEFDRLAELDRRNVVDVDLGSDTFKANAHRHMAEWASRPPFYVLGKGPPQVVVGRYADVHRVFSDTETF
ASEMPTGPGWEQFHKFMDAQFITQMDGSQHARLRRLLMPAFSSRRIDQLKASITTIIEKMLDRIEAKGPDFDG
MGDLGSHLVVDALLEAMIQMDPRRKAVFVAFHDVLPATTYTKPGEPFPENCRVAMQHVMDEIKVIIEERRTKP
HSDFISDLVNAREAGDKLTDKELFDQIFGLAGGSLSATSRAAGGALLLLYTHDDQRQQLIDNPALIPDALEEC
LRLGSNGYFTFPRIAVKDTEVGGTRILKGMVVRPSPLSPNYDADVFPDPLRFDIHRKPKRILSFGAGPHHCIG
NILGRTAITIAITRLIARFPKAHISEPNFTPVYGGAVGELRLQRLPMRTH

>CYP1745A1(Arad_7831)*Agrobacterium radiobacter*

MVAGVAIEEAPTHMRLIFGPALQSDPHSTYRLFRENWPVARIDWPHRGSGEYLITRYSDVISIATSQGVSIDR
SLAALPLSKQNRIEEPLFGTVLTLDNSVHANARRVLSSRLHRRTSALEPVLERTFAQLVGNYADGCELDIIED
LAEPICETMVAWITGFDLAAVKAMQVPSEKFVSKGFTFGSEFVSPGVLDCVAYLKDAIRRQLMSSLPDETLLE
ARLASGDRDVASYDINTCLLVITAGLQTTKHSIGNVLATLLEHPKLIEELRTSPTSVPSAVEECLRFDGPSQA
VGRVLLRDTFMRDVLLKKGAFVRLALSAANRDPEHFPDPDVPNIHRESCRHLAFGFSQHACLGARITRICLNV
VVQGLLNEFPAIELSPRGSERLLGSSLRGYRSYFVTVKRA

>CYP1746A1(Swit_1027)*Sphingomonas wittichii*

MGAQEAMDERTGGIEDVRFADPAFLADPWTPLIRLQEEAPVFYSRNQGGWIISRYDDVRAAFADRRLSASRVD
QLFRGMPEELQKQLEAVRLYTGLIVNRLDGRDHTRIRVLMLKAFDAAVVRTVQGFIGDVIDEILDECERLGEF
DFMQAVSATLPTRVMQKLFDLPDEYRPLLFKLASDFTSASSAANVTPELLLKLDATIRTMNDVFNAIIPEREK
NPGDDLISMMVNARDGLNKLSNDEMLAQLHGMVVAGAETTAHTLATQLVQIVRQEKLLATLRETPERAFDLVT
ELLRYPGTVKCMTRFAGEDIELHGQTITKGDLLWIMHAGANVDTRHWDDPFGTDIDRHNLRDSMAFGPGLHFC
VGHMLARTELTEFFTRALRRFDVEILQQDFEMVPSYIFYGYKELRVRFTPRAA

>CYP1747A1(Hbal_0119)*Hirschia baltica*

MSVIDFTDKDYLRSPQQVLFELSQKGPILPVKIPKIGKAVLISGNDALIDLLKSPHRFTTDSKTVGKDAIFAS
AGIIPEFNPGNGSLFLKDGKDHRDIRQHLDQALRKSGLKSLRPNIALLADQLLETHTPAKRLAANQRDLVLQF
AMPLPAMVMIELLGIPKDVAPNILKWAIQISFPSGILGNRHAQSARKHLYDYLKEFFSTPSLLPPESLGAKLL
ENKPAASADQNANIVDIAFFLILAGHATTSSLISLGCLTLLDHPAQLELLKQDWTRVPDVVEEILRFASPAQT
TAPRYAKDTFDFHGHTFKRGDIAFGFLAASNMDPASNKNPLEFDITRDQNRQISFGAGAHFCGGAQLAHIIAE
IALQRLFSKWPEMRLSSEAAGSLAWSKKFGVRNLNKMIVETQQSTSRF

>CYP1748A1(TMO_3386)*Tistrella mobilis* KA081020-065

MIDIASAFGVLPPGAEYAADPHPVLSALRNTPPIRTTGGDVWLVARHADILSVLLAEPGSTASFNAFDTAGGD
RHRGLVRRLRSWVSRLSDADVMPAVQGVVDDLTARLVREGTADLVRDFADRVPMAVMRDLLGIPPADVPVLET
MAGRILSGYDPGWTGQSASVGPAKTMLDAYFRQRLAAARKGGATPLLATILETGTADGLDDAALADICSKLLV
TGTTTTAGCIANILVRRLRDGIALPSDPENHMVLVDELVRLDTPVLGVRRRLLRPFHLSDGADGTILLPKDAV
VYLMTSIADRDPAIVGDVGTSCPLGSRVPHLAFGRGAFHCLGAPLARLEIAALLVAGAPLMDRLCTAGPIRWR
EAWLLHEPSALPVQIIANPSESASR

>CYP1749A1(NHU_00153)*Rhodovulum sulfidophilum*

MPAVNSSAWTDPDRFLTDPDDVAAVLRDPHFTVSQLTAHLDEIVARGGPRFPHLEAMARHAFAFQSGEPHLAA
RRATAPFFAAPALAAWTPVAELAIAAALASLETSRFPDLMRDYCEAAFLDFIRAFCGCPGGADDRVLDLIRLA
NETTRPMLSLKALGRIDAALGELLDHLSTAPVPGLEPGLVSFPAFLGRRADRLADPEAAPYIALSALVGSHTV
AQSLALALHGMLLGDLGAWQAAARPGWTDTGLDRAISLYPSTLTLVRIAGDGARIGGCPCHKGETAVMDVVGA
NAALRRRVPDGSAHLSFGAGRHKCPGAPLSRMFLARAIPALARRFPRLALHRDGVRFHVTPLVQYPVALPVTR
DARSRRLTGRMVEIRDLDEARAIVNDDATWSPPRMEPYLETLAERSGRDLGLARLVARNAMFFMSGPRHAAIR
RAVADSLGGNRLACWQPLIEAETRTALTRLASVPAPDLIADYAEPLFRGIAGPVLGLAPVDPERFDALAPILQ
DVLEPWLPIRELDRLQEVIGEILAGLGLPATEGLPSTPLLTRLMEEDLPETDPEDLRALVLVMYGASFNMAHT
LGNILHHLLSLPPEDRRDAADPAWIDAELEGLISLCASPKYIYRMARGPARIGEIEIRTDETLRLQLLSIDRG
IGTGNLAFGHGLHRCVGAALSKRMLRTALPALFARFPGLALRPQRHRYLDMTQTVALATLPCRLGPSGKDRP

>CYP1749B1(ROSMUCSMR3_02121)*Roseovarius mucosus*

MRGNIWADPTSFFSTPSDAAAVLRDLELPRIDQYLAALSDNSDIKLENLNRLSANTLVAMTGDDHLKTRRVIA
PFFSKDGLKHWADMLDAGVSHALAQLAAAPKPDLVTNFTIPLFLRVMPQMFGLKIDESEAHFRAIETVQRLTE
PYLSVPTLRKLEHAVGLLIRTFPDPSEHQSPDKPETLIEYLYRRRSDLPAGLDARYLVLGILVGSNSATQSVA
FALYGLLTGPPRNWQDAARPGWAERELPRLLSLYQSTRTLVRVAPKATTIAGCPFHKGQAAVVDIVKTNSCLR
SDTKSGVRHMSFGSGAHKCPGSFLSEMVFAQSIPALARRFPSIVLNKEQCSFVQTPMMQAPTALPCETAQGSH
RATSRLCDIRDTALAHEVVRDNDRFSPPQMTNYLSTLAQHSGRDLTIAVKISQNALFFMDGPRHEALRTAIGR
RLGATRLQDWTDLADTAIADALDNLTRMPNPDLVTGFTDLLRQTVIAPILGVFPSDPSQFERLAPELQAVLEP
WLSMRDLLAVQEKFNEAMALMTVPTTANCSLLSDLLAEPPEGFSTDDLKAVVLVLYGASFNLSHTLANILHLI
LSDPVEERANIETPDWISTRLEELIALCASPKYIYRMARADLELAGMAMKAGDTARLTLQAINRDRNPGALHL
SFGQGMHRCVGAALSRLIIRRAIPAVFERFPNLSLVNQGQRYFPMSQTVALSALPCVLSS

>CYP1750A1(Swit_1847)*Sphingomonas wittichii*

MEYDPFSDEAMRDPHALYTRLRQAPGPHFIEKYNAWALVHFEDVWTVSVKHEADITFTAGQPLTNVLLGEPIP
HAFPSMDMSEHRKWRRVVHADYTKGNVEKDRDRITALARELLAPLAARKRFDFYTDYVNRLFAINAGHNLGID
RDNAIEWRRLIDETMHRNPGQVGTSSARNQQAGMELHRQLHAYVERIRRDPTLAGGHCAKYLDAEVEGRRLEN
QDIVNIIIIFLTVGSGTTPNVCAGTVYYLARHPEQRDAVLADPALIPKAFFEAARIDQPTNMLCRRAVNDFSV
RGAEIKAGQNLLMIYASANRDEAEFERADAFDIFRSYPRDLTYGIGGHKCLGMHLATMAGTIALEEFFKVSGR
YEVDAAHCTRAYGEFLSGFDHLPVILGA

>CYP1751A1(K663_20498)*Sphingobium* sp. MI1205

MDTAPQAPQSKCPVSRVTIDTLDTMQTVLRSKAVEENADVSADNYVCPSALAEINVREFQQGNLAMSNGDVHR
QRRRLLNSLVRPEQLVRFREDIILPSVDRWMGRAVKRNEEGAFGCDLAGVVELIFLEFAAKIIGIEGVESEEG

MARLRGCALPIFGGLSAAHFEDREGVTTAGIEAKKVFVEEFYRPSLEHTRRQLQRLAAGELTEDDVPLNLLQM
IATQAHETYADEDVGIKEAILFFVATTGTSVQAVLSTVEYLLDWFEKYPADRACIEDMEFVSNALQEALRLRA
PFVPFLTRLAVDDLDAGDVQLKAGDEVQAWVARAGRDRAIFGDDANEFNPRREIPEGISRYGLAFATGAHQCL
GLRAVLGNDGKSGSHLKLVQGLFRAGIRRDPKLQAQTLPLKASDDPRDQIPTYISFPVILDNWSARA

>CYP1752A1(PhaeoP97_01374) *Phaeobacter porticola*

MTRKEIPIFDANSTMFQNIGLLKVAETATRQHGDIVAIRVSDTRDLYIAGSPECFHYWRGHQGHFQTDLGDIA
SNQAITRMLLGDELQDPRWSDVWVLTANRMSAIARDFDGWLETALADATRTLIAELPQDGTAADLRDICRDWS
IRAVCPAIFGADLDVAEIAQGVADVEGFYFAMSTKDAAETADHETLAEFKAARGFLDKAITTALRNSTPSDPN
LIAQIATVIPQDVSEADRLNLLRPTVGHVITEKLNIGGLSLLWTLVQLAQDPQLAADISAECHGNDPLTMRDA
DSPLALSSVKEGLRLFPELPFIYRITSEEVPIGDYVIPSGATVVFAPWLVHRDPRFWESPKRFQGDRFLDEAQ
HRAHYYPFGVGARIRSRANFMLHQLTLAARSICAAHSLALSPDCPRGNIRPLLRSALIPRGVVPVVFAPRDAS
AMAADDQSFADATQ

>CYP1752A2(PGA2_c13670)*Phaeobacter inhibens* 2.10

MTRKEIPIFDANSTMFQNIGLLKVAETATRQHGDIVAIRVSDTRDLYIAGSPECFHYWRGHQGHFQTDLGDIA
SNQAITRMLLGDELQDPRWSDVWVLTANRMSAIARDFDGWLETALADATRTLITELPQDDTAADLRDICRDWS
IRAVCPAIFGADLDVAEIAQGVADVEGFYFAMSTKDAAETADHENLPEFKAARGFLDKAITTALRNSTPSDPN
LIAQIATVIPQDVSEADRLNLLRPTVGHVITEKLNIGGLSLLWTLVQLAQDPQLAADISAECHGNDPLTMRDA
DSPLALSTVKEGLRLFPELPFIYRITSEEVPIGDYVIPSGATVVFAPWLVHRDPRFWEAPKRFQGDRFLDEAQ
HRAHYYPFGVGARIRSRANFMLHQLTLAARSICAAHSLALSPDCPRGNIRPLLRSALIPRGAVPVVFAPRDAS
AMAADDQSLADAIQ

>CYP1752A3(PGA1_c13770)*Phaeobacter inhibens* DSM 17395

MTRKEIPIFDANSTMFQNIGLLKVAETATRQHGDIVAIRVSDTRDLYIAGSPECFHYWRGHQGHFQTDLGDIA
SNQAITRMLLGDELQDPRWSDVWVLTANRMSAIARDFDGWLETALADATRTLITELPQDGTAADLRDICRDWS
IRAVCPAIFGADLDVAEIAQGVADVEGFYFAMSTKDTAETADHENLPEFKAARGFLDKAITTALDNSTPSDPN
LIAQIATVIPQDVSEADRLNLLRPTVGHVITEKLNIGGLSLLWTLVQLAQDPQLAADISAECHGNDPLTMLDA
DSPLALSTVKEGLRLFPELPFIYRITSEEVPIGDYVIPSGATVVFAPWLVHRDPRFWESPKRFQGDRFLDEAQ
HRAHYYPFGVGARIRSRANFMLHQLTLAVRSICAAHSLALSPDCPRGNIRPLLRSALIPRGAVPVVFAPRDAS
AMAADDQSLADAIQ

>CYP1752A4(Gal_02028)*Phaeobacter gallaeciensis* DSM 26640

MTQKEIPIFDANSTMFQNIGLLKVAETATRQHGDIVAIRVSETRDLYIAGSPECFHYWRGHQGHFQTDLGDIA
SNQAITRMLLGDELQDPRWSDIWVLTANRMSAIARDFDGWLETALAEATQTLIRDLPQDGSAVDLRDLCRDWS
IRAVCPAIFGADIDVAEIAQGVADVEGFYFAMSTKDASETADHEMMPEFQAARGFLDKAITAALNDSSASDRN
LIAQIATVIPPDVSEADRLNLLRPTVGHVITEKLNIGGLSLLWTLVQLAQDPQLAADIATECHGSDPMTLRDA
ESPLALSTVKEGLRLFPELPFIYRITSEAVPIGDYVIPSGATVVFAPWLVQRDPRFWDNPKRFQGDRFLDEGR
HRAHYFPFGVGARIRSRANFMLHQLTLAARAICAAQTFALAPDCPRGNIRPLLRSALIPRGAVPVVFHPRAQA
DATTGDRALADAAQ

>CYP1753A1(RPE_0081)*Rhodopseudomonas palustris* BisA53

MPLPRFDPFHPDFGRDPFAVYRRYRDAGPLHWGKPSNPGLPGTWYAFDQRGSSIILKNDAFGREAHRNADKDL
LAPELRWLIDHVDRWVMFRDPPYHRHLRAPLSGFFSAPNAARYDAQRADLVATRLAALRDRSAFDAVHHLAAP
LSAAALAMIVGLPADELAAIRGWTRYLTQVLDLKRDRRLYAYCGAVAHDLGGRLRHKIINSDRDGGDHLLGRL
HDRVATEQLTLDEASSTLMLLLATGETTVRGVIANGLLTLLEHPDQLALLAAQPELAEAAVEEVLRFAPPVHM
AGRIARDDIELSGTLLRRGDAVVACLASANRDGRMVREPDRFDITRARNRHISFGSGRHICLGMWFARAMARD
TFRAVAPWLVDYRLSGAPLWRDSILFRALASLPLERRPSAVPIRKKTRHEPVSAV

>CYP1754A1(BSY17_3197)*Sphingobium* sp. RAC03

MTSITDPDAPISPAMPPFRSEGNGPWVITRHADASLILRSPAFRTASPMMGIERIADRAKRDYSDLLALLDQT
MVFQNGSAHLVSRQAVRHIVQQSMARWPASTILAEARHIATSVPSGGAIDAVVHLADPLPSRILASMLGLPVD

QAMALRADAMLVTRAWSHSCSLRDYDACQDAARRLRRGLRSGLQRDSIETDDASFDLIAFLLVAGADSIAGTI
SAALDLLARNPAWQARLRSEPTLMPGFIRETLRLAGPLRRLNRRVAQAAVDIGGVSIAPGDLVLLRIDRAHRD
AEAFADPDQIDPGRKNAPLLAFGGGAHMCQGPLLGAMEAEMMVTAIIDRFQIHPAAARGVLLDHEDWRVFAHL
PLILTPVAAEQ

>CYP1755A1(Msil_0731)*Methylocella silvestris*

MTGADKEIFWFKTATRRGPRGWPLLGILPHLRRDVLKTVREACAYGDLVELKSPLFSLLLLNSADHVARVLQT
NSANYGRTRFHAMLKPVLGNGLLTSEGDLWRAQRRIIQPAFLFARLPAYAVFEAKALARLCQRWEQAARSQEP
VDVAAEMSALSVEVIVGALFGDSAPGAALNVRRDINLLQDYITGRFWSVVPAKLAELLPTAGNLAYQGARQRL
DRLIYSLIEAHRQTPVPGAILSLMLDYRDPETGAGMGERQIRDEVMTLFLAGHETTATALTWLWLLLAENPES
RARVEGEADAAQGLAGNLPEQNFIKRAAQEALRLYPPIWSFSRHALGDDMLGEHFVGKDSNLMVCPYTLHRHP
DYWTDPERFDPDRFLPARAERRPRFAYVPFGGGPRICVGGLFALAELVNTTTTIAARFRLEPISADVQPEALV
TLRPKGGLLMKISER

>CYP103A4(aro:B0909_23830)*Agrobacterium rhizogenes*

MISASIDPDNVSIADLDRFGHAIFKEWRPKRPFLRRQDGVYLVLRADDVLGLSSDPRTRQ
IETELMLNRGVNEGAVFDFVRYSMLFSNKETHSRRRSPFTRTFAFRMIENLRPQIRQLTE
ILFRDLKELGSFNFVGEYASKLPAVTIASLLGLPPSDIPYFTQLVYRVARCLSPSWRDAD
FPDIEKSAVEFKNYVQAVIDDRRTDPRDDFLSSYINATREAEDLSPVEGLTQLMLIILAG
TDTTKTGLTALTGQLLQHRQAWDALLNDGTLVAAAVEEGLRFEPPVGSYPRSTLADIDLD
GFILPKNSLLALCTMSALRDEKHYVHPQLFDIHRKQMRWHMVFGTGEHRCLGEALARLEL
QEGLATVLRHAPNLSIEGEWQAVQGHGGVRRIAEMRVGFNREN

>CYP1101A28(pye:A6J80_18645)*Paracoccus yeei*

MIPPKPASRQGKGSVWRFVRDFRRDILSAQSERLYRAWMAEFRGPGVHSFTCNDPALVAL
ILKQRPMDFPKSPRMAAGLQPLLGNSSFISNGETWLHQRRIIDPAFEGGRLREAFPAMWD
AAQSGVVRLGKLADGQDFDIEPHTSHIAADVIFRTLFSIPIEDATAAQVFSAFRDHQDAQ
PMVNLGGLIALPRWARYHSRRVRHSARRIRGLIRQLVSRRASQIAAGNAPQDLATKIMTT
PDPQTGAVFSDREMIDEVATFFLAGHETGASALAWALYLLAENPEWQEKLAKEARENLRP
DFAAIKSLPLSRAVFREALRLYPPVAMTVRQCAAPETLRGRKAPRGAQLVLSPFHLHRHE
RLWNNPDHFDPARWETENGRACMRDAYIPFSAGARACPGAGFAMIEGPLILSALLAAYRL
EPGVERPVPIMRLTLRGKNGIHLRLLKR

>CYP152E(pye:A6J80_21040)*Paracoccus yeei*

MPETPLNERLPIPSDGVLDSTLAFLRDGYLFVSKRCDRLDSDLFSTRLMLRRAVCMRGAV
AAELFYGSDHFTRVGAMPVTVARLLQDYGSVQTLDGAPHRQRKRMFMSLVKPEAVARLAE
LTEAEWLRRLPNWARAPKIVLFDEVRAILAQAVCAWAGVPLEGAETARRTRELSEMIESA
GSVGPRNWRGQLLRARSERWARDLVRRARAGTLPLGEDAPLRVIATHLDHDGRLLDVKVA
AVELLNVLRPTVAVARFMVFAALVLHRHPDVRQRVLNEGEPYLEAFAQEVRRQAPFFPII
GGRVKEPFDWRGHRFAQGDWVILDLYGTNHDPRSWSAPKAFRPERLLERRPSPFDLIPQG
GGEHLQGHRCPGEGIAIELTKLATRMLTGRMRYDVPAQDLLIDLARVPALPESGFVIKHV
AATRA

>CYP2140A1(sbd:ATN00_08815)*Sphingobium baderi*

MTAESITLESSYWDTADKPPFDFYEEVQSMGGIAWDDRMRSWIVANYNLAQEVLKDDELF
QHPYMSMKAGGDYMKIRSDNPRSSMFLKGERHRAFHKWWLVELLSPKVVREYRKDLVDPW
IDHLIKGLEDRPAFDLVEDFAERVPMGIFAQLLGLPQHDHAYLDHVKALNDAIGRFASVA
NALQLEGEASAEARRVADEAIAAAEELNDILKPLVSERKNGEGSDFISQLWRGGHRVFDD
WNELDTLDACRRLLFAGVDTTTHAISNAFHMLLTDDSLMAQVRDEGSRYLDRFVEEVLRL
NGSIQFRPRRASASTELNGKSIAQGDMLTVAIIGANRDPRHYGCPHAVDLERARPHDHLA
FAYGPRTCLGAHLARAEIASAVSKMLVRFPAMRLDPSRPAPAFSGFLMRSWRPLHVLAG

>CYP1311A1(spmi:K663_03440)*Sphingobium* sp. MI1205

MSYNPLDPAVTANPYPHYAKLRAEEPVKWLDMMQGFAVSRWDDVEEVLGDGKTFSSAQFW
PALLGEYDPVPEVQPMISLDPPGHVRIRKLANKAFVPSRVGALQDRIRTVTHELIDGIFA
RHGKEGEFDFVWEFSGLFPVSVIAEVLGVDIERRVDFKRWVDDLLAAGNRAAYGPERLAE
IDSSSRSIRAYFEQVYDERSANPGDDLISGFIQAEVNGERLSRSEVLNMAILLLIGGVET
TTNLLGITFAHFKTHPEIAAAIWADPTRIPLLLEEMLRFDGPVQMLFRHTTRDTELAGVA
IPKGSLVLPLLGSANHDERKFDRSEELVLDRDPKEIMSFGQGPHFCLGSYLSRMEARSAL
EILTSRFESLEAVSDTVKWSDSYFARGPKTLPVRFKAR

>CYP1311A1(K663_02370)*Sphingobium* sp. MI1205

MNTLQISGNFYDRETLANPYPLMAEFREQAPVMRTVDPAGRAIFVVTEMALIEQVAKRTDAFSNDFGHLFVAG
NEHPEVAGILDQEPLRATLLLGSDAPDHTRYRALVNAVFATGRVAHLTPMIEALTDELIDGFIEKGQCNFVDE
FAVLLPTYIIADILGLPRDKYDQVKKWSDAVIMVVGRTGTKEDEIHAAHEIVDFRRYLRETVAARRIEPRDDL
ISNLVTVKTEGVQPFDDIEATALAFEIAVAGNETTRNTLMSGLVQLLRHPDQMQALIDDSSLVDNAVEEILRY
ETPASSMWRIAREDMTLGGTDIPAGSTLLLRYDGGNRDPRRFEDPDRFDIRRRNARTHIAFGAPSVHRCLGQM
LARKELVVGFRQLLKRLRNIRIVEGSDTSYQPSLMFHTIGSLHIAFDPGERVHR

>CYP1326A2(APE42395)*Sulfitobacter* sp. AM1-D1

MPDTSRPEELVYDPTDPAVMADPFPVYARLREEDPVHWSPSLRSWIITRYTDVRDLLLSD
TMSVNRLLQFYESLPPADAMLLRDIVHYLNLWLAFRDPPDHTRVRRIMRHAFTADAIATM
RPQIHEISDLLLDRLAAAGTDDVDLIREYALQLPAFVIMDLLDVPRDMLDEFKEWSDDMA
VFIGGARNSDDKYERAARGCRHMSDYFRKLIEERTANPKPGFLMDLINARDEGDKLSDDE
LVATCILVLFAGHETTTNLIGNATLLLLRHPEQLDRLRADPELVDPMIEEVLRFDGPTNA
LVRVVARDHDLHGRTLREGERVFVMVNSANRDPRMFDDADRFDITRDQNRHLTFGQGIHL
CLGAKLAREEGRIAVRKLFERFPDLTLNPAEPPEWLDAM

>CYP195A21(APE42395)*Sulfitobacter* sp. AM1-D1

MFDLSHPPVDFLENPFPHYDRLLAEAPVCPQPDGSVLVSRHADLNAIYRDTGLYISDKKQ
AFAPKFGPGSPLYEHHTTSLVFNDPPLHTRVRRIMTSALTPKALARMEPGLIATVDTLLD
SMPENPDLVEDFASTIPIQIIGNLLDVPMAERAPLRDWSLAILGALEPTLTDAQLERGHA
AVRDFKAYLQDLIARRRAAPGDPETDVLTRLIKGDDTGRLTEVELIQNCIFILNAGHETT
TNLIGNGLALLHDHPDQKRRLMDDPALIDTAVEEVLRLRSPNQFGNRETTAPVEIGGHAI
PAGTNLHLCIGAANRDPAVFDAPGTFDIARRPNRHLAFAGGPHVCVGLTLARMEGRIAIA
RFLRRYPGYALTGDRVPGGRIRFHGYRHLPARLGKARGQ

>CYP2334A1(APE42395)*Sulfitobacter* sp. AM1-D1

MRTSEINSQAFGPSTIREAADANRDPILVGTPWRRGYGHWQVSRMRDVRAVLAHPGTTTD
DLAFRVFSRFRERRGGLDSIFHLSRQISRAHRTPNDAERMDAITVTKRLMECLPAQDFRS
PLAALVSSGETEADAMVTVVQKPIMEWRAAALGINAALGQEIEIDSIDILASLDRTAIPD
FIKLEPKAARITGNLSAMNVTWPAGQTIPLMHLISPAFLAIEPLSKTAGLMLAHLADNPD
LQERLRNRAELRTAYLQEVERLLGAVRYVARQIGPAGLDLGEIRLPPHSLVSSDLAAANR
DPELWDDPEAFRFDRPRLQTATFSFGSLACTGSQLSRLFLSTLLDATLATVRLAAPFTGQ
QNDRKFSRWSIIRGYESCRLRFAAT

Short P450s

**Species name**	**Short P450s protein ID**	**Count**
Bradyrhizobium oligotrophicum	(aol:S58_14600)	1
Bradyrhizobium japonicum E109	(bjp:RN69_37425)	1
Bradyrhizobium diazoefficiens USDA 110	(bja:blr2143)	1
Sphingopyxis macrogoltabida 203	(smaz:LH19_26550)	1
Sphingobium sp. MI1205	(spmi:K663_02365)	3
	(spmi:K663_17471)	
	(spmi:K663_18021)	
Sinorhizobium fredii NGR234	(rhi:NGR_a02720)	1
Rhizobium etli bv. phaseoli IE4803	(rep:IE4803_PB00358)	2
	(rep:IE4803_PB00400)	
Rhizobium tropici	(rtr:RTCIAT899_PB00445)	1
Rhizobium sp. N1341	(rhn:AMJ98_PC00305)	1
Rhizobium phaseoli	(rpha:AMC79_PC00282)	1
Rhizobium sp. N731	(rhx:AMK02_PC00300)	1
Methylobacterium aquaticum	(maqu:Maq22A_c28670)	1
Porphyrobacter sp. LM 6	(porl:BG023_11390)	1
Rhizobium etli CIAT 652	(rec:RHECIAT_PB0000292)	1
Sinorhizobium meliloti GR4	(smeg:C770_GR4pB068)	1
Methylorubrum extorquens AM1	(mea:Mex_1p4500)	7
	(mea:Mex_1p4502)	
	(mea:Mex_1p4503)	
	(mea:Mex_1p4504)	
	(mea:Mex_1p4505)	
	(mea:Mex_1p4506)	
	(mea:Mex_1p4508	
Salipiger profundus	(tpro:Ga0080559_TMP4501)	9
	(tpro:Ga0080559_TMP4502)	
	(tpro:Ga0080559_TMP4503)	
	(tpro:Ga0080559_TMP4504)	
	(tpro:Ga0080559_TMP4505)	
	(tpro:Ga0080559_TMP4507)	
	(tpro:Ga0080559_TMP4508)	
	(tpro:Ga0080559_TMP4509)	
	(tpro:Ga0080559_TMP450)	
Pelagibaca abyssi	(paby:Ga0080574_TMP4500)	
	(paby:Ga0080574_TMP4501)	
	(paby:Ga0080574_TMP4504)	
Sinorhizobium meliloti SM11	(smx:SM11_pC0152)	2
	(smx:SM11_pC0153)	
Phaeobacter inhibens 2.10	(pgl:PGA2_95p450)	1
Octadecabacter temperatus	(otm:OSB_05620 K20497)	1

Azospirillum brasilense Sp245	(abs:AZOBR_p450001)	11
	(abs:AZOBR_p450002)	
	(abs:AZOBR_p450003)	
	(abs:AZOBR_p450006)	
	(abs:AZOBR_p450007)	
	(abs:AZOBR_p450008)	
	(abs:AZOBR_p450009)	
	(abs:AZOBR_p450010)	
	(abs:AZOBR_p450011)	
	(abs:AZOBR_p450013)	
	(abs:AZOBR_p450014)	
Brucella abortus 2308	(bmf:BAB1_0684 K03584)	1
Candidatus Puniceispirillum marinum	(apb:SAR116_0659)	1
Bradyrhizobium sp. ORS 278	(bra:BRADO7133)	1
