# Supplementary material for: Ancient Bacterial Class Alphaproteobacteria Cytochrome P450 Monooxygenases Can Be Found in Other Bacterial Species
Source: Int J Mol Sci. 2021 May 24;22(11):5542. doi: 10.3390/ijms22115542 (PMC8197338; doi:10.3390/ijms22115542)
Supplement: Supplementary file 1 [file ijms-22-05542-s001.zip › Supplementary Information.pdf]

Type of the Paper (Article)

Ancient bacterial class *Alphaproteobacteria* cytochrome P450 monooxygenases can be found in other bacterial species

Nomfundo Nzuza [1], Tiara Padayachee [1], Puleng Rosinah Syed [2], Justyna Dorota Kryś [3], Wanping Chen [4], Dominik Gront [3*], David R Nelson [5*], Khajamohiddin Syed [1*]

[1] Department of Biochemistry and Microbiology, Faculty of Science and Agriculture, University of Zululand, KwaDlangezwa 3886, South Africa; nomfundonzuza11@gmail.com (N.N.); teez07padayachee@gmail.com (T.P.); khajamohiddinsyed@gmail.com (K.S.)
[2] Department of Pharmaceutical Chemistry, College of Health Sciences, University of KwaZulu-Natal, Durban 4000, South Africa; prosinah@gmail.com (P.R.S.)
[3] Faculty of Chemistry, Biological and Chemical Research Center, University of Warsaw, Pasteura 1, 02-093 Warsaw, Poland; juchxd@gmail.com (J.D.K); dgront@gmail.com (D.G)
[4] Department of Molecular Microbiology and Genetics, University of Göttingen, 37077 Göttingen, Germany; chenwanping1@foxmail.com (W.C.)
[5] Department of Microbiology, Immunology and Biochemistry, University of Tennessee Health Science Center, Memphis, TN, 38163; drnelson1@gmail.com (D.N.R.)
* Correspondence: dgront@gmail.com (D.G.), drnelson1@gmail.com (D.N.R.) and khajamohiddinsyed@gmail.com (K.S.)

Table S1. Information on Alphaproteobacterial species and their respective genera used in the study. Species abbreviations, their genome IDs (GenBank) and presence or absence of P450s in different species were also presented in the table.

Genus	Total no. of species	Species have P450s	Species don't have P450s	Species code	Species name	Genome IDs
Rickettsia	47	0	47	rpr	*Rickettsia prowazekii Madrid* E	AJ235269
				rpo	*Rickettsia prowazekii* BuV67-CWPP	CP003393
				rpw	*Rickettsia prowazekii* Chernikova	CP003391
				rpz	*Rickettsia prowazekii Dachau*	CP003394
				rpg	*Rickettsia prowazekii* GvV257	CP003395
				rps	*Rickettsia prowazekii Katsinyian*	CP003392
				rpv	*Rickettsia prowazekii* RpGvF24	CP003396
				rpq	*Rickettsia prowazekii* Rp22	CP001584
				rpl	*Rickettsia prowazekii Breinl*	CP004889
				rpn	*Rickettsia prowazekii* NMRC Madrid E	CP004888
				rty	*Rickettsia typhi Wilmington*	AE017197
				rtt	*Rickettsia typhi* TH1527	CP003397
				rtb	*Rickettsia typhi* B9991CWPP	CP003398
				rcm	*Rickettsia canadensis* McKiel	CP000409
				rcc	*Rickettsia canadensis* CA410	CP003304
				rbe	*Rickettsia bellii* RML369-C	CP000087
				rbo	*Rickettsia bellii* OSU 85-389	CP000849
				rco	*Rickettsia conorii*	AE006914
				rfe	*Rickettsia felis*	CP000054
				rak	*Rickettsia akari*	CP000847
				rri	*Rickettsia rickettsii Sheila Smith*	CP000848
				rrj	*Rickettsia rickettsii Iowa*	CP000766
				rra	*Rickettsia rickettsii Arizona*	CP003307

				rrc	*Rickettsia rickettsii Colombia*	CP003306
				rrh	*Rickettsia* rickettsii *Hauke*	CP003318
				rrb	*Rickettsia rickettsii Brazil*	CP003305
				rrn	*Rickettsia rickettsii Hino*	CP003309
				rrp	*Rickettsia rickettsii* Hlp#2	CP003311
				rrm	*Rickettsia rickettsii Morgan*	CP006010
				rrr	*Rickettsia rickettsii* R	CP006009
				rms	*Rickettsia* massiliae MTU5	CP000684
				rmi	*Rickettsia massiliae* AZT80	CP003319
				rpk	*Rickettsia peacockii*	CP001227
				raf	*Rickettsia africae*	CP001612
				rhe	*Rickettsia heilongjiangensis*	CP002912
				rja	*Rickettsia japonica*	AP011533
				rsv	*Rickettsia slovaca* 13-B	CP002428
				rsw	*Rickettsia slovaca* D-CWPP	CP003375
				rph	*Rickettsia philipii*	CP003308
				rau	*Rickettsia australis*	CP003338
				rmo	*Rickettsia montanensis*	CP003340
				rpp	*Rickettsia parkeri*	CP003341
				rre	*Rickettsia rhipicephali*	CP003342
				ram	*Rickettsia amblyommatis* GAT-30V	CP003334
				rab	*Rickettsia amblyommatis* Ac37	CP012420
				rmc	*Rickettsia monacensis*	LN794217
				ric	*Rickettsia* sp. MEAM1 *(Bemisia tabaci)*	CP016305
Orientia	2	0	2	ots	*Orientia tsutsugamushi Boryong*	AM494475
				ott	*Orientia tsutsugamushi Ikeda*	AP008981
Phycorickettsia	1	0	1	ptc	*Candidatus Phycorickettsia trachydisci*	CP027845
Wolbachia	10	0	10	wol	*Wolbachia* wMel *(Drosophila melanogaster)*	AE017196
				wri	*Wolbachia* wRi *(Drosophila simulans)*	CP001391

				wen	*Wolbachia* wHa (*Drosophila simulans*)	CP003884
				wed	*Wolbachia* wNo (*Drosophila simulans)*	CP003883
				wpi	*Wolbachia* wPip *(Culex quinquefasciatus)*	AM999887
				wbm	*Wolbachia* wBm *(Brugia malayi)*	AE017321
				woo	*Wolbachia* wOo *(Onchocerca ochengi)*	HE660029
				wcl	*Wolbachia* wCle *(Cimex lectularius)*	AP013028
				weo	*Wolbachia* wFol *(Folsomia candida)*	CP015510
				wpp	*Wolbachia pipientis*	CP031221
Anaplasma	10	0	10	ama	*Anaplasma marginale* St. Maries	CP000030
				amf	*Anaplasma marginale* Florida	CP001079
				amw	*Anaplasma marginale* Dawn	CP006847
				amp	*Anaplasma marginale* Gypsy Plains	CP006846
				acn	*Anaplasma centrale*	CP001759
				aph	*Anaplasma phagocytophilum* HZ	CP000235
				apy	*Anaplasma phagocytophilum* HZ2	CP006616
				apd	*Anaplasma phagocytophilum* Dog2	CP006618
				apha	*Anaplasma phagocytophilum* JM	CP006617
				aoh	*Anaplasma ovis*	CP015994
Ehrlichia	14	0	14	eru	*Ehrlichia ruminantium Welgevonden* (South Africa)	CR767821
				erw	*Ehrlichia ruminantium* Welgevonden (France)	CR925678
				erg	*Ehrlichia ruminantium* Gardel	CR925677
				ecn	*Ehrlichia canis*	CP000107
				ech	*Ehrlichia chaffeensis* Arkansas	CP000236
				echa	*Ehrlichia chaffeensis* Heartland	CP007473
				echj	*Ehrlichia chaffeensis* Jax	CP007475
				echl	*Ehrlichia chaffeensis* Liberty	CP007476
				echs	*Ehrlichia chaffeensis* Osceola	CP007477

				echv	*Ehrlichia chaffeensis* Saint Vincent	CP007478
				echw	*Ehrlichia chaffeensis* Wakulla	CP007479
				Echp	*Ehrlichia chaffeensis* West Paces	CP007480
				Emr	*Ehrlichia muris*	CP006917
				Ehh	*Ehrlichia* sp. HF	CP007474
Neorickettsia	3	0	3	Nse	*Neorickettsia sennetsu*	CP000237
				Nri	*Neorickettsia risticii*	CP001431
				Nhm	*Neorickettsia helminthoeca*	CP007481
Midichloria	1	0	1	Mmn	*Candidatus Midichloria mitochondrii*	CP002130
Fokinia	1	0	1	Fso	*Candidatus Fokinia solitaria*	CP025989
Unclassified rickettiales	2	0	2	Rbt	*Rickettsiales bacterium* Ac37b	CP009217
				Ren	*Rickettsiales* endosymbiont *of Stachyamoeba lipophore*	CP033611
Paracaedibacter	1	0	1	Paca	Candidatus Paracaedibacter acanthamoebae	CP008941
Paracaedimonas	1	0	1	Caq	*Candidatus Paracaedimonas acanthamoebae*	CP008936
Nucleicultrix	1	0	1	Naf	*Candidatus Nucleicultrix amoebiphila*	CP008743
Unclassified *Holosporaceae*	1	0	1	Eaa	Endosymbiont of *Acanthamoeba* sp. UWC8	CP004403
Mesorhizobium	8	7	1	Mlo	*Mesorhizobium japonicum* MAFF 303099	BA000012
				Mln	*Mesorhizobium loti* NZP2037	CP016079
				Mci	*Mesorhizobium ciceri (biovar Biserrulae)*	CP002447
				mop	*Mesorhizobium opportunistum*	CP002279
				mam	*Mesorhizobium australicum*	CP003358
				mamo	*Mesorhizobium amorphae*	CP015318
				meso	*Mesorhizobium* sp. B7	CP018171
				mesw	*Mesorhizobium* sp. WSM1497	CP021070
Chelativorans	1	1	0	mes	*Chelativorans* sp. BNC1	CP000390
Hoeflea	1	1	0	hoe	*Hoeflea* sp. IMCC20628	CP011479
Aminobacter	2	1	1	aak	*Aminobacter aminovorans*	CP015005
				amih	*Aminobacter* sp. MSH1	CP026265

Phyllobacterium	1	0	1	pht	*Phyllobacterium zundukense*	CP017940
Parvibaculum	1	1	0	pla	*Parvibaculum lavamentivorans*	CP000774
Unclassified *Rhodobiaceae*	1	0	1	rbs	*Rhodobiaceae bacterium* SMS8	CP030277
Sinorhizobium	15	14	1	sme	*Sinorhizobium meliloti* 1021	AL591688
				smk	*Sinorhizobium meliloti* AK83	CP002781, CP002782, CP002783
				smq	*Sinorhizobium meliloti* BL225C	CP002740
				smx	*Sinorhizobium meliloti* SM11	CP001830
				smi	*Sinorhizobium meliloti* Rm41	HE995405
				smeg	*Sinorhizobium meliloti* GR4	CP003933
				smel	*Sinorhizobium meliloti* 2011	CP004140
				smer	*Sinorhizobium meliloti* RMO17	CP009144
				smd	*Sinorhizobium medicae*	CP000738
				rhi	*Sinorhizobium fredii* NGR234	CP001389
				sfh	*Sinorhizobium fredii* HH103	HE616890
				sfd	*Sinorhizobium fredii* USDA 257	CP003563
				six	*Sinorhizobium* sp. RAC02	CP016450
				same	*Sinorhizobium Americanum*	CP013107
				sino	*Sinorhizobium* sp. CCBAU 05631	CP023063
Ensifer	3	2	1	ead	*Ensifer adhaerens* OV14	CP007236, CP007237
				eah	*Ensifer adhaerens Casida* A	CP015880
				esj	*Ensifer sojae*	CP023067
Agrobacterium	8	8	0	atu	*Agrobacterium fabrum*	AE007869 (circ), AE007870 (lin)
				ara	*Agrobacterium radiobacter*	CP000628, CP000629
				atf	*Agrobacterium tumefaciens* Ach5	CP011246 (circ), CP011247 (lin)
				ata	*Agrobacterium tumefaciens* S33	CP014259 (circ), CP014260 (lin)

				avi	*Agrobacterium vitis*	CP000633, CP000634
				agr	*Agrobacterium* sp. H13-3	CP002248 (circ), CP002249 (lin)
				agc	*Agrobacterium* sp. RAC06	CP016499
				aro	*Agrobacterium rhizogenes*	CP019701, CP019702
Rhizobium	21	18	3	ret	*Rhizobium etli* CFN 42	CP000133
				rec	*Rhizobium etli* CIAT 652	CP001074
				rel	*Rhizobium etli* bv. *mimosae* Mim1	CP005950
				rep	*Rhizobium* etli bv. *phaseoli* IE4803	CP007641
				rei	*Rhizobium* sp. IE4771	CP006986
				rle	*Rhizobium leguminosarum* bv. *viciae* 3841	AM236080
				rlt	*Rhizobium leguminosarum* bv. *trifolii* WSM2304	CP001191
				rlg	*Rhizobium leguminosarum* bv. *trifolii* WSM1325	CP001622
				rlb	*Rhizobium leguminosarum* bv. *trifolii* WSM1689	CP007045
				rlu	*Rhizobium leguminosarum* bv. *trifolii* CB782	CP007067
				rtr	*Rhizobium tropici*	CP004015
				rir	*Rhizobium* sp. IRBG74	HG518322
				rhl	*Rhizobium favelukesii*	HG916852
				rga	*Rhizobium gallicum*	CP006877
				rhn	*Rhizobium* sp. N1341	CP013505
				rpha	*Rhizobium phaseoli*	CP013580
				rht	*Rhizobium* sp. NT-26	FO082820
				rhx	*Rhizobium* sp. N731	CP013601
				rhv	*Rhizobium* sp. S41	CP016320, CP016433
				rhk	*Rhizobium* sp. Kim5	CP021124
				rez	*Rhizobium esperanzae*	CP013500

Neorhizobium	4	2	2	ngl	*Neorhizobium galegae* bv. *officinalis* bv. officinalis HAMBI 1141	HG938355
				ngg	*Neorhizobium galegae* bv. *orientalis* HAMBI 540	HG938353
				neo	*Neorhizobium sp. SOG26*	CP025512
				nen	*Neorhizobium* sp. NCHU2750	CP030827
Liberibacter	7	1	6	las	*Candidatus Liberibacter asiaticus* psy62	CP001677
				laa	*Candidatus Liberibacter asiaticus* gxpsy	CP004005
				lat	*Candidatus Liberibacter asiaticus* Ishi-1	AP014595
				lso	*Candidatus Liberibacter solanacearum*	CP002371
				lcc	*Liberibacter crescens*	CP003789
				lar	*Candidatus Liberibacter americanus*	CP006604
				lau	*Candidatus Liberibacter africanus*	CP004021
Shinella	1	1	0	shz	*Shinella* sp. HZN7	CP015736
Brucella	43	30	13	bme	*Brucella melitensis* bv. 1 16M	AE008917, AE008918
				bmel	*Brucella melitensis* bv. 1 16M	CP007763, CP007762
				bmi	*Brucella melitensis* ATCC 23457	CP001488, CP001489
				bmz	*Brucella melitensis* M28	CP002459, CP002460
				bmg	*Brucella melitensis* M5-90	CP001851, CP001852
				bmw	*Brucella melitensis* NI	CP002931, CP002932
				bmee	*Brucella melitensis* bv. 3 Ether	CP007760, CP007761
				bmf	*Brucella abortus* 2308	AM040264, AM040265
				bmb	*Brucella abortus* bv. 1 9-941	AE017223, AE017224
				bmc	*Brucella abortus* S19	CP000887, CP000888
				baa	*Brucella abortus* A13334	CP003176, CP003177
				babo	*Brucella abortus* bv. 2 86/8/59	CP007765, CP007764
				babr	*Brucella abortus* bv. 6 870	CP007709, CP007710
				babt	*Brucella abortus* 63 75	CP007663, CP007662
				babb	*Brucella abortus* BDW	CP007681, CP007680

				babu	*Brucella abortus* bv. 9 C68	CP007705, CP007706
				babs	*Brucella abortus* BER	CP007682, CP007683
				babc	*Brucella abortus* NCTC 10505	CP007700, CP007701
				bms	*Brucella suis* 1330	AE014291, AE014292
				bsi	*Brucella suis* 1330	CP002997, CP002998
				bsf	*Brucella suis* bv. 1	CP006961, CP006962
				bsui	*Brucella suis* bv. 2 Bs143CITA	CP007695, CP007696
				bsup	*Brucella suis* bv. 2 PT09143	CP007691, CP007692
				bsuv	*Brucella suis* bv. 2 PT09172	CP007693, CP007694
				bsuc	*Brucella suis* bv. 2 Bs364CITA	CP007697, CP007698
				bmt	*Brucella suis* ATCC 23445	CP000911, CP000912
				bsz	*Brucella suis* bv. 3	CP007719, CP007718
				bsv	*Brucella suis* VBI22	CP003128, CP003129
				bsw	*Brucella suis* ZW043	CP009094, CP009095
				bsg	*Brucella suis* ZW046	CP009096, CP009097
				bov	*Brucella ovis*	CP000708, CP000709
				bcs	*Brucella canis* ATCC 23365	CP000872, CP000873
				bsk	*Brucella canis* HSK A52141	CP003174, CP003175
				bol	*Brucella canis Oliveri*	HG803175, HG803176
				bcar	*Brucella canis* RM6/66	CP007758, CP007759
				bcas	*Brucella canis* SVA13	CP007629, CP007630
				bmr	*Brucella microti*	CP001578, CP001579
				bpp	*Brucella pinnipedialis* B2/94	CP002078, CP002079
				bpv	*Brucella pinnipedialis* 6/566	CP007743, CP007742
				bcet	*Brucella ceti* TE10759-12	CP006896, CP006897
				bcee	*Brucella ceti* TE28753-12	CP006898, CP006899
				bvl	*Brucella vulpis*	LN997863, LN997864
				bru	*Brucella* sp. 2002734562	CP016979, CP016980
Ochrobactrum	4	3	1	oan	*Ochrobactrum anthropi* ATCC 49188	CP000758, CP000759

				oah	*Ochrobactrum anthropi* OAB	CP008820, CP008819
				ops	*Ochrobactrum pseudogrignonense*	CP015775, CP015776
				och	*Ochrobactrum* sp. A44	CP022604, CP022603
Bradyrhizobium	14	10	4	bja	*Bradyrhizobium diazoefficiens* USDA 110	BA000040
				bju	*Bradyrhizobium japonicum* USDA 6	AP012206
				bjp	*Bradyrhizobium japonicum* E109	CP010313
				bra	*Bradyrhizobium* sp. ORS 278	CU234118
				bbt	*Bradyrhizobium* sp. BTAi1	CP000494
				brs	*Bradyrhizobium* sp. S23321	AP012279
				aol	*Bradyrhizobium oligotrophicum*	AP012603
				brc	*Bradyrhizobium* sp. CCGE-LA001	CP013949
				brad	*Bradyrhizobium* sp. BF49	LN901633
				bic	*Bradyrhizobium icense*	CP016428
				bro	*Bradyrhizobium* sp. ORS 285	LT859959
				brk	*Bradyrhizobium* sp. SK17	CP025113
				bot	*Bradyrhizobium ottawaense*	CP029425
				brq	*Bradyrhizobium* sp. 2 39S1MB	CP029426
Rhodopseudomonas	7	7	0	rpa	*Rhodopseudomonas palustris* CGA009	BX571963
				rpb	*Rhodopseudomonas palustris* HaA2	CP000250
				rpc	*Rhodopseudomonas palustris* BisB18	CP000301
				rpd	*Rhodopseudomonas palustris* BisB5	CP000283
				rpe	*Rhodopseudomonas palustris* BisA53	CP000463
				rpt	*Rhodopseudomonas palustris* TIE-1	CP001096
				rpx	*Rhodopseudomonas palustris* DX-1	CP002418
Nitrobacter	2	2	0	nwi	*Nitrobacter winogradskyi*	CP000115
				nha	*Nitrobacter hamburgensis*	CP000319
Oligotropha	3	0	3	oca	*Oligotropha carboxidovorans* OM5 (Mississippi)	CP001196

				ocg	*Oligotropha carboxidovorans* OM5 (Goettingen)	CP002826
				oco	*Oligotropha carboxidovorans* OM4	CP002821
Bosea	4	2	2	bop	*Bosea* sp. PAMC 26642	CP014301
				bos	*Bosea* sp. RAC05	CP016464
				bvv	*Bosea vaviloviae*	CP017147
				boi	*Bosea* sp. Tri-49	CP017946
Variibacter	1	0	1	vgo	*Variibacter gotjawalensis*	AP014946
Bartonella	20	0	20	bhe	*Bartonella henselae* Houston-1	BX897699
				bhn	*Bartonella henselae* BM1374163	HG965802
				bhs	*Bartonella henselae* BM1374165	HG969191
				bqu	*Bartonella quintana Toulouse*	BX897700
				bqr	*Bartonella quintana* RM-11	CP003784
				bbk	*Bartonella bacilliformis*	CP000524
				btr	*Bartonella tribocorum* CIP 105476	AM260525
				btx	*Bartonella tribocorum* BM1374166	HG969192
				bgr	*Bartonella grahamii*	CP001562
				bcd	*Bartonella clarridgeiae*	FN645454
				baus	*Bartonella australis*	CP003123
				bvn	*Bartonella vinsonii*	CP003124
				banc	*Bartonella ancashensis*	CP010401
				bapi	*Bartonella apis*	CP015625
				bart	*Bartonella* sp. JB15	CP019787
				bara	*Bartonella* sp. A1379B	CP019780
				barw	*Bartonella* sp. WD16.2	CP019781
				barr	*Bartonella* sp. Raccoon60	CP019786
				baro	*Bartonella* sp. 1-1C	CP019489
				barj	*Bartonella* sp. JB63	CP019788
Xanthobacter	1	1	0	xau	*Xanthobacter autotrophicus*	CP000781

Azorhizobium	1	1	0	azc	*Azorhizobium caulinodans*	AP009384
Starkeya	1	0	1	sno	*Starkeya novella*	CP002026
Methylorubrum	6	5	1	mex	*Methylorubrum extorquens* PA1	CP000908
				mea	*Methylorubrum extorquens* AM1	CP001510
				mdi	*Methylorubrum extorquens* DM4	FP103042
				mch	*Methylorubrum extorquens* CM4	CP001298
				mpo	*Methylorubrum populi*	CP001029
				mza	*Methylorubrum zatmanii*	CP021054
Methylobacterium	11	6	5	mrd	*Methylobacterium radiotolerans*	CP001001
				met	*Methylobacterium* sp. 4-46	CP000943
				mno	*Methylobacterium nodulans*	CP001349
				mor	*Methylobacterium oryzae*	CP003811
				meta	*Methylobacterium* sp. AMS5	CP006992
				maqu	*Methylobacterium aquaticum*	AP014704
				mphy	*Methylobacterium phyllosphaerae*	CP015367
				mee	*Methylobacterium currus*	CP028843, CP028844
				metd	*Methylobacterium* sp. DM1	CP029173
				metx	*Methylobacterium* sp. XJLW	CP016429
				mets	*Methylobacterium* sp. 17SD2-17	CP029550
Microvirga	2	0	2	moc	*Microvirga ossetica*	CP016616
				miv	*Microvirga* sp. 17 mud 1-3	CP029481
Beijerinckia	1	1	0	bid	*Beijerinckia indica*	CP001016
Methylocella	1	1	0	msl	*Methylocella silvestris*	CP001280
Chelatococcus	2	0	2	chel	*Chelatococcus* sp. CO-6	CP012398
				cdq	*Chelatococcus daeguensis*	CP018095
Hyphomicrobium	4	4	0	hdn	*Hyphomicrobium denitrificans* ATCC 51888	CP002083
				hdt	*Hyphomicrobium denitrificans* 1NES1	CP005587
				hmc	*Hyphomicrobium* sp. MC1	FQ859181
				hni	*Hyphomicrobium nitrativorans*	CP006912

Rhodomicrobium	1	0	1	rva	*Rhodomicrobium vannielii*	CP002292
Pelagibacterium	1	1	0	phl	*Pelagibacterium halotolerans*	CP003075
Filomicrobium	2	2	0	fil	*Candidatus Filomicrobium marinum* W	LN829118
				fiy	*Candidatus Filomicrobium marinum* Y	LN829119
Devosia	2	1	1	deq	*Devosia* sp. H5989	CP011300
				dei	*Devosia* sp. I507	CP026747
Blastochloris	2	0	2	bvr	*Blastochloris viridis*	CP012946
				blag	*Blastochloris* sp. GI	AP018907
Rhodoplanes	1	1	0	rhz	*Rhodoplanes* sp. Z2-YC6860	CP007440
Maritalea	1	0	1	mmyr	*Maritalea myrionectae*	CP021330
Methylocystis	3	2	1	msc	*Methylocystis* sp. SC2	HE956757
				mbry	*Methylocystis bryophila*	CP019948
				mros	*Methylocystis rosea*	CP034086
Methylosinus	1	0	1	mtw	*Methylosinus trichosporium*	CP023737
Pleomorphomonas	1	0	1	pleo	*Pleomorphomonas* sp. SM30	AP017626
Martelella	3	1	2	mey	*Martelella endophytica*	CP010803
				maad	*Martelella* sp. AD-3	CP014275
				mmed	*Martelella mediterranea*	CP020330
Aureimonas	1	0	1	aua	*Aureimonas* sp. AU20	CP006367
Breoghania	1	0	1	brn	*Breoghania* sp. L-A4	CP031841
Hodgkinia	4	0	4	hci	*Candidatus Hodgkinia cicadicola* Dsem	CP001226
				hct	*Candidatus Hodgkinia cicadicola* TETULN	CP008699
				hcc	*Candidatus Hodgkinia cicadicola* TETUND1	CP007232
				hcd	*Candidatus Hodgkinia cicadicola* TETUND2	CP007233
Methyloceanibacter	1	1	0	mcg	*Methyloceanibacter caenitepidi*	AP014648
Tokpelaia	1	0	1	thd	*Candidatus Tokpelaia hoelldoblerii*	CP017315
Pseudorhodoplanes	1	1	0	psin	*Pseudorhodoplanes sinuspersici*	CP021112
Hartmannibacter	1	0	1	hdi	*Hartmannibacter diazotrophicus*	LT960614
Unclassified *Rhizobiales*	1	0	1	rbm	*Rhizobiales bacterium* NRL2	CP016093

Caulobacter	7	4	3	ccr	*Caulobacter vibrioides* CB15	AE005673
				ccs	*Caulobacter vibrioides* NA1000	CP001340
				cak	*Caulobacter* sp. K31	CP000927
				cse	*Caulobacter segnis*	CP002008
				chq	*Caulobacter henricii*	CP013002
				cmb	*Caulobacter mirabilis*	CP024201
				cfh	*Caulobacter flavus*	CP026100
Phenylobacterium	2	1	1	pzu	*Phenylobacterium zucineum*	CP000747
				phb	*Phenylobacterium* sp. HYN0004	CP029479
Brevundimonas	7	0	7	bsb	*Brevundimonas subvibrioides*	CP002102
				brd	*Brevundimonas* sp. DS20	CP012897
				bne	*Brevundimonas naejangsanensis*	CP015614
				brg	*Brevundimonas* sp. GW460-12-10-14-LB2	CP015511
				brl	*Brevundimonas* sp. LM2	CP019508
				bvc	*Brevundimonas vesicularis*	CP022048
				bdm	*Brevundimonas diminuta*	CP035093
Asticcacaulis	1	1	0	aex	*Asticcacaulis excentricus*	CP002395, CP002396
Unclassified *Caulobacteraceae*	1	0	1	cbot	*Caulobacteraceae bacterium* OTSz_A_272	CP013244
Ruegeria	4	2	2	sil	*Ruegeria pomeroyi*	CP000031
				sit	*Ruegeria* sp. TM1040	CP000377
				rue	*Ruegeria* sp. NKC1-1	CP031092
				rua	*Ruegeria* sp. AD91A	CP031946
Epibacterium	1	0	1	rmb	*Epibacterium mobile*	CP015230
Rhodobacter	7	5	2	rsp	*Rhodobacter sphaeroides* 2.4.1	CP000143, CP000144
				rsh	*Rhodobacter sphaeroides* ATCC 17029	CP000577, CP000578
				rsq	*Rhodobacter sphaeroides* ATCC 17025	CP000661
				rsk	*Rhodobacter sphaeroides* KD131	CP001150, CP001151
				rcp	*Rhodobacter capsulatus*	CP001312

				rhp	*Rhodobacter* sp. LPB0142	CP017781
				rbl	*Rhodobacter blasticus*	CP020470
Jannaschia	1	1	0	jan	*Jannaschia* sp. CCS1	CP000264
Roseobacter	2	2	0	rde	*Roseobacter denitrificans*	CP000362
				rli	*Roseobacter litoralis*	CP002623
Paracoccus	10	3	7	pde	*Paracoccus denitrificans*	CP000489, CP000490
				pami	*Paracoccus aminophilus*	CP006650
				pye	*Paracoccus yeei*	CP020442
				pcon	*Paracoccus contaminans*	CP020612
				pzh	*Paracoccus zhejiangensis*	CP025430
				paro	*Paracoccus* sp. BM15	CP025408
				paru	*Paracoccus* sp. CBA4604	CP025583
				pamn	*Paracoccus aminovorans*	LN832559
				pmut	*Paracoccus mutanolyticus*	CP030239
				pars	*Paracoccus* sp. SC2-6	CP030918
Dinoroseobacter	1	1	0	dsh	*Dinoroseobacter shibae*	CP000830
Ketogulonicigenium	3	3	0	kvu	*Ketogulonicigenium vulgare* Y25	CP002224
				kvl	*Ketogulonicigenium vulgare* WSH-001	CP002018
				kro	*Ketogulonicigenium robustum*	CP019937
Pseudovibrio	1	1	0	psf	*Pseudovibrio* sp. FO-BEG1	CP003147
Phaeobacter	6	4	2	pga	*Phaeobacter inhibens* DSM 17395	CP002976
				pgl	*Phaeobacter inhibens* 2.10	CP002972
				pgd	*Phaeobacter gallaeciensis* DSM 26640	CP006966
				php	*Phaeobacter porticola*	CP016364
				ppic	*Phaeobacter piscinae*	CP010681
				phq	*Phaeobacter* sp. LSS9	CP031956
Octadecabacter	3	2	1	oat	*Octadecabacter antarcticus*	CP003740
				oar	*Octadecabacter arcticus*	CP003742
				otm	*Octadecabacter temperatus*	CP012160

Leisingera	2	1	1	lmd	*Leisingera methylohalidivorans*	CP006773
				lej	*Leisingera* sp. NJS204	CP035417
Roseibacterium	1	1	0	red	*Roseibacterium elongatum*	CP004372
Planktomarina	1	1	0	ptp	*Planktomarina temperata*	CP003984
Celeribacter	4	2	2	cid	*Celeribacter indicus*	CP004393
				cmar	*Celeribacter marinus*	CP012023
				ceh	*Celeribacter ethanolicus*	CP022196
				cmag	*Celeribacter manganoxidans*	CP021404
Marinovum	1	1	0	malg	*Marinovum algicola*	CP010855
Confluentimicrobium	1	1	0	con	*Confluentimicrobium* sp. EMB200-NS6	CP010869
Rhodovulum	3	2	1	rsu	*Rhodovulum sulfidophilum*	AP014800
				rhm	*Rhodovulum* sp. MB263	CP020384
				rhc	*Rhodovulum* sp. P5	CP015039
Pannonibacter	1	1	0	pphr	*Pannonibacter phragmitetus*	CP013068
Halocynthiibacter	1	0	1	hat	*Halocynthiibacter arcticus*	CP014327
Labrenzia	3	2	1	lap	*Labrenzia* sp. CP4	CP011927
				lagg	*Labrenzia aggregata*	CP019630
				labr	*Labrenzia* sp. VG12	CP022529
Defluviimonas	1	1	0	daa	*Defluviimonas alba*	CP012661
Yangia	2	1	1	yan	*Yangia* sp. CCB-MM3	CP014595, CP014596
				ypac	*Yangia pacifica*	CP022189, CP022190
Sulfitobacter	5	1	4	suam	*Sulfitobacter* sp. AM1-D1	CP018076
				spse	*Sulfitobacter pseudonitzschiae*	CP022415
				sulz	*Sulfitobacter* sp. SK012	CP025804
				suli	*Sulfitobacter* sp. JL08	CP025815
				suld	*Sulfitobacter* sp. D7	CP020694
Donghicola	1	0	1	don	*Donghicola* sp. JLT3646	CP018572
Salipiger	1	1	0	tpro	*Salipiger profundus*	CP014796
Tateyamaria	1	1	0	tom	*Tateyamaria omphalii*	CP019312

Pelagibaca	1	1	0	paby	*Pelagibaca abyssi*	CP015093
Thioclava	1	1	0	thw	*Thioclava nitratireducens*	CP019437
Roseovarius	2	1	1	rmm	*Roseovarius mucosus*	CP020474
				rok	*Roseovarius* sp. AK1035	CP030099
Roseitalea	1	0	1	rpod	*Roseitalea porphyridii*	CP036532
Yoonia	1	1	0	lvs	*Yoonia vestfoldensis*	CP021431
Antarctobacter	1	0	1	aht	*Antarctobacter heliothermus*	CP022540
Rhodobaca	1	0	1	rbg	*Rhodobaca barguzinensis*	CP024899
Sagittula	1	0	1	sagu	*Sagittula* sp. P11	CP021913
Thalassococcus	2	0	2	thas	*Thalassococcus* sp. SH-1	CP027665
				thaa	*Thalassococcus* sp. S3	CP022303
Gemmobacter	1	0	1	geh	*Gemmobacter* sp. HYN0069	CP028918
Tabrizicola	1	0	1	taw	*Tabrizicola* sp. K13M18	CP034328
Silicimonas	1	0	1	salo	*Silicimonas algicola*	CP034588
Sedimentitalea	1	0	1	sedi	*Sedimentitalea* sp. W43	CP033219
Unclassified *Rhodobacteraceae*	1	0	1	rbz	*Rhodobacteraceae bacterium* G7	CP021114
Maricaulis	1	1	0	mmr	*Maricaulis maris*	CP000449
Hyphomonas	1	1	0	hne	*Hyphomonas neptunium*	CP000158
Hirschia	1	1	0	hba	*Hirschia baltica*	CP001678
Glycocaulis	1	0	1	gak	*Glycocaulis alkaliphilus*	CP018911
Hyphomonadaceae	1	1	0	hbc	*Hyphomonadaceae bacterium* UKL13-1	CP012156
Zymomonas	8	0	8	zmo	*Zymomonas mobilis* subsp. *mobilis* ZM4	AE008692
				zmn	*Zymomonas mobilis* subsp. *mobilis* NCIMB 11163	CP001722
				zmm	*Zymomonas mobilis* subsp. *mobilis* ATCC 10988	CP002850
				zmb	*Zymomonas mobilis* subsp. *mobilis* ATCC 29191	CP003704

				zmi	*Zymomonas mobilis* subsp. *mobilis* CP4 = NRRL B-14023	CP006818
				zmc	*Zymomonas mobilis* subsp. *mobilis* CP4 = NRRL B-14023	CP003715
				zmr	*Zymomonas mobilis* subsp. *mobilis* NRRL B-12526	CP003709
				zmp	*Zymomonas mobilis* subsp. *pomaceae* ATCC 29192	CP002865
Novosphingobium	7	3	4	nar	*Novosphingobium aromaticivorans*	CP000248
				npp	*Novosphingobium* sp. PP1Y	FR856862
				npn	*Novosphingobium pentaromativorans*	CP009291
				nre	*Novosphingobium resinovorum*	CP017075
				nov	*Novosphingobium* sp. P6W	CP030352, CP030353
				not	*Novosphingobium* sp. THN1	CP028347
				ntd	*Novosphingobium tardaugens*	CP034179
Sphingopyxis	11	7	4	sal	*Sphingopyxis alaskensis*	CP000356
				sphk	*Sphingopyxis fribergensis*	CP009122
				sphp	*Sphingopyxis* sp. 113P3	CP009453
				smag	*Sphingopyxis macrogoltabida* EY-1	CP012700
				smaz	*Sphingopyxis macrogoltabida* 203	CP009429
				ster	*Sphingopyxis terrae*	CP013342
				sgi	*Sphingopyxis granuli*	CP012199
				sphl	*Sphingopyxis* sp. LPB0140	CP018154
				sphq	*Sphingopyxis* sp. QXT-31	CP019449
				spho	*Sphingopyxis* sp. MG	CP026381
				sphu	*Uncultured Sphingopyxis* sp. UC10	LT598653
Sphingomonas	17	3	14	swi	*Sphingomonas wittichii*	CP000699
				sphd	*Sphingomonas wittichii* DC-6	CP021181
				sphm	*Sphingomonas* sp. MM-1	CP004036

				stax	*Sphingomonas* taxi	CP009571
				sphi	*Sphingomonas* sp. WHSC-8	CP010836
				ssan	*Sphingomonas sanxanigenens*	CP006644
				snj	*Sphingomonas* sp. NIC1	CP015521
				smy	*Sphingomonas melonis*	CP017578
				span	*Sphingomonas panacis*	CP014168
				skr	*Sphingomonas koreensis*	CP018820
				splm	*Sphingomonas* sp. LM7	CP019511
				splk	*Sphingomonas* sp. LK11	CP013916
				sphj	*Sphingomonas* sp. JJ-A5	CP018221
				spkc	*Sphingomonas* sp. KC8	CP016306
				sphc	*Sphingomonas* sp. Cra20	CP024923
				sphf	*Sphingomonas* sp. FARSPH	CP029985
				spha	*Sphingomonas* sp. YZ-8	CP032829
Sphingobium	14	9	5	sjp	*Sphingobium japonicum*	AP010803, AP010804
				sch	*Sphingobium chlorophenolicum*	CP002798, CP002799
				ssy	*Sphingobium* sp. SYK-6	AP012222, AP012223
				syb	*Sphingobium* sp. YBL2	CP010954
				sbd	*Sphingobium baderi*	CP013264
				spmi	*Sphingobium* sp. MI1205	CP005188, CP005189
				sphb	*Sphingobium* sp. EP60837	CP015986, CP015987
				sphr	*Sphingobium* sp. RAC03	CP016456
				sinb	*Sphingobium indicum*	CP013070
				spht	*Sphingobium* sp. TKS	CP005083, CP005084
				shyd	*Sphingobium hydrophobicum*	CP022745, CP022746
				sya	*Sphingobium yanoikuyae*	CP023741
				sclo	*Sphingobium cloacae*	AP017655
				spyg	*Sphingobium* sp. YG1	AP018518, AP018519
Citromicrobium	1	1	0	cij	*Citromicrobium* sp. JL477	CP011344

Sphingorhabdus	3	1	2	sphg	*Sphingorhabdus* sp. M41	CP014545
				sfla	*Sphingorhabdus flavimaris*	CP022336
				sphy	*Sphingorhabdus* sp. YGSMI21	CP022548
Blastomonas	2	1	1	blas	*Blastomonas* sp. RAC04	CP016460
				bfw	*Blastomonas fulva*	CP020083
Rhizorhabdus	1	0	1	rdi	*Rhizorhabdus dicambivorans*	CP023449
Sphingosinicella	2	0	2	smic	*Sphingosinicella microcystinivorans*	AP018711
				sphs	*Sphingosinicella* sp. BN140058	CP035501
Erythrobacter	7	2	5	eli	*Erythrobacter litoralis* HTCC2594	CP000157
				elq	*Erythrobacter litoralis* DSM 8509	CP017057
				ery	*Erythrobacter* sp. s21-N3	CP011310
				egn	*Erythrobacter gangjinensis*	CP018097, CP018098
				efv	*Erythrobacter flavus*	CP022528
				erk	*Erythrobacter* sp. KY5	CP021912
				err	*Erythrobacter* sp. YH-07	CP031357
Altererythrobacter	6	5	1	aay	*Altererythrobacter atlanticus*	CP011452
				amx	*Altererythrobacter marensis*	CP011805
				aep	*Altererythrobacter epoxidivorans*	CP012669
				anh	*Altererythrobacter namhicola*	CP016545
				ado	*Altererythrobacter dongtanensis*	CP016591
				alb	*Altererythrobacter* sp. B11	AP018498
Croceicoccus	2	1	1	cna	*Croceicoccus naphthovorans*	CP011770
				cman	*Croceicoccus marinus*	CP019602
Porphyrobacter	3	2	1	pns	*Porphyrobacter neustonensis*	CP016033
				porl	*Porphyrobacter* sp. LM 6	CP017113
				phz	*Porphyrobacter* HT-58-2	CP022600
Gluconobacter	4	0	4	gox	*Gluconobacter oxydans* 621H	CP000009
				goh	*Gluconobacter oxydans* H24	CP003926
				goy	*Gluconobacter oxydans* DSM 3504	CP004373

				gal	*Gluconobacter albidus*	CP014689
Granulibacter	4	0	4	gbe	*Granulibacter bethesdensis* CGDNIH1	CP000394
				gbh	*Granulibacter bethesdensis* CGDNIH2	CP003183
				gbc	*Granulibacter bethesdensis* CGDNIH3	CP003181
				gbs	*Granulibacter bethesdensis* CGDNIH4	CP003182
Acidiphilium	2	0	2	acr	*Acidiphilium cryptum*	CP000697
				amv	*Acidiphilium multivorum*	AP012035
Gluconacetobacter	2	2	0	gdi	*Gluconacetobacter diazotrophicus* PA1 5 (Brazil)	AM889285
				gdj	*Gluconacetobacter diazotrophicus* PA1 5 (JGI)	CP001189
Komagataeibacter	5	0	5	gxy	*Komagataeibacter medellinensis*	AP012159
				gxl	*Komagataeibacter xylinus*	CP004360
				kna	*Komagataeibacter nataicola*	CP019875
				keu	*Komagataeibacter europaeus*	CP021467
				ksc	*Komagataeibacter saccharivorans*	CP023036
Acetobacter	17	1	16	apt	*Acetobacter pasteurianus* IFO 3283-01	AP011121
				apw	*Acetobacter pasteurianus* IFO 3283-01-42C	AP011163
				apf	*Acetobacter pasteurianus* IFO 3283-03	AP011128
				apu	*Acetobacter pasteurianus* IFO 3283-07	AP011135
				apg	*Acetobacter pasteurianus* IFO 3283-12	AP011170
				apq	*Acetobacter pasteurianus* IFO 3283-22	AP011142
				apx	*Acetobacter pasteurianus* IFO 3283-26	AP011149
				apz	*Acetobacter pasteurianus* IFO 3283-32	AP011156
				apk	*Acetobacter pasteurianus* 386B	HF677570
				asz	*Acetobacter senegalensis*	LN606600
				asv	*Acetobacter oryzifermentans*	CP011120
				aace	*Acetobacter aceti*	CP014692
				aper	*Acetobacter persici*	CP014687
				apom	*Acetobacter pomorum*	CP023657

				ato	*Acetobacter tropicalis*	CP022699
				aasc	*Acetobacter ascendens*	CP015164
				acet	*Acetobacter* sp. JWB	CP030871
Asaia	1	0	1	abg	*Asaia bogorensis*	AP014690
Kozakia	1	0	1	kba	*Kozakia baliensis*	CP014674
Roseomonas	2	0	2	rgi	*Roseomonas gilardii*	CP015583, CP015584
				ros	*Roseomonas* sp. FDAARGOS_362	CP024588
Neoasaia	1	0	1	nch	*Neoasaia chiangmaiensis*	CP014691
Commensalibacter	1	0	1	coq	*Commensalibacter* sp. AMU001	CP033087
Rhodospirillum	3	0	3	rru	*Rhodospirillum rubrum* ATCC 11170	CP000230
				rrf	*Rhodospirillum rubrum* F11	CP003046
				rce	*Rhodospirillum centenum*	CP000613
Pararhodospirillum	1	0	1	rpm	*Pararhodospirillum photometricum*	HE663493
Magnetospirillum	5	1	4	mag	*Magnetospirillum magneticum*	AP007255
				mgy	*Magnetospirillum gryphiswaldense* MSR-1 v2	HG794546
				mgry	*Magnetospirillum gryphiswaldense* MSR-1	CP027526
				magx	*Magnetospirillum* sp. XM-1	LN997848
				magn	*Magnetospirillum* sp. ME-1	CP015848
Azospirillum	9	3	6	azl	*Azospirillum* sp. B510	AP010946
				ali	*Azospirillum lipoferum*	FQ311868
				abs	*Azospirillum brasilense* Sp245	HE577327
				abq	*Azospirillum brasilense* Az39	CP007793
				abf	*Azospirillum brasilense* Sp7	CP012914
				ati	*Azospirillum thiophilum*	CP012401, CP012402, CP012403, CP012404
				ahu	*Azospirillum humicireducens*	CP015285
				azt	*Azospirillum* sp. TSH58	CP022364
				azm	*Azospirillum* sp. M2T2B2	CP029829
Tistrella	1	1	0	tmo	*Tistrella mobilis*	CP003236

Endolissoclinum	2	0	2	thal	*Candidatus Endolissoclinum faulkneri* L2	CP003539
				efk	*Candidatus Endolissoclinum faulkneri* L5	CP006745
Thalassospira	3	0	3	txi	*Thalassospira xiamenensis*	CP004388
				thac	*Thalassospira marina*	CP024199
				tii	*Thalassospira indica*	CP031555
Magnetospira	1	1	0	magq	*Magnetospira* sp. QH-2	FO538765
Haematospirillum	1	0	1	hjo	*Haematospirillum jordaniae*	CP014525
Nitrospirillum	1	0	1	nao	*Nitrospirillum amazonense*	CP022110, CP022111, CP022112, CP022113
Niveispirillum	1	0	1	ncb	*Niveispirillum cyanobacteriorum*	CP025611, CP025612
Parvularcula	1	0	1	pbr	*Parvularcula bermudensis*	CP002156
Magnetococcus	1	0	1	mgm	*Magnetococcus marinus*	CP000471
Pelagibacter	2	0	2	pub	*Candidatus Pelagibacter ubique*	CP000084
				pel	*Candidatus Pelagibacter* sp. IMCC9063	CP002511
Unclassified *Pelagibacteraceae*	2	0	2	apc	*Alpha proteobacterium* HIMB59	CP003801
				apm	*Alpha proteobacterium* HIMB5	CP003809
Micavibrio	2	0	2	mai	*Micavibrio aeruginosavorus* ARL-13	CP002382
				man	*Micavibrio aeruginosavorus* EPB	CP003538
Polymorphum	1	1	0	pgv	*Polymorphum gilvum*	CP002568
Phreatobacter	1	0	1	phr	*Phreatobacter* sp. S-12	CP027668
Puniceispirillum	1	1	0	apb	*Candidatus Puniceispirillum marinum*	CP001751
Unclassified *Alphaproteobacteria*	1	0	1	abaw	*Alphaproteobacteria bacterium* WS11	CP032509
Methylovirgula	1	0	1	mlg	*Methylovirgula ligni*	CP025086
Haematobacter	1	0	1	hml	*Haematobacter massiliensis*	CP035510

Table S2. Comparative analysis of P450s and those associated with secondary metabolite biosynthetic gene clusters in the bacterial class *Alphaproteobacteria*. Standard abbreviations representing type of clusters as indicated in anti-SMASH [39] were used in the Table.

**Species name**	**Species code**	**No. of P450s**	**No. of P450 families**	**No. of P450 subfamilies**	**No. of BGCs**	**No. of BGCs have P450s**	**BGC type**	**No.of P450s in BGCs**	**P450 name**
Bradyrhizobium oligotrophicum	aol	17	12	14	14	1	NRPS	1	CYP108L2
Bradyrhizobium japonicum E109	bjp	16	13	14	8				
Novosphingobium aromaticivorans	nar	16	11	14	2	1	terpene	1	CYP153C1
Sphingomonas wittichii	swi	16	16	16	4				
Bradyrhizobium diazoefficiens USDA 110	bja	15	13	13					
Bradyrhizobium japonicum USDA 6	bju	12	10	10	9				
Bradyrhizobium sp. BF49	brad	12	10	11	3				
Blastomonas sp. RAC04	blas	12	10	12	4				
Bradyrhizobium sp. ORS 278	bra	11	10	11	8				
Sphingopyxis macrogoltabida 203	smaz	11	8	9	10	1	NRPS	1	CYP1302A1
Sphingobium sp. MI1205	spmi	11	10	10	6				
Bradyrhizobium sp. BTAi1	bbt	10	10	10	15				

Bradyrhizobium sp. S23321	brs	10	9	9	7	1	hserlactone	1	CYP199A26
Rhodopseudomonas palustris HaA2	rpb	10	10	10	6				
Parvibaculum lavamentivorans	pla	9	5	5	3				
Sinorhizobium fredii NGR234	rhi	9	9	9	5				
Rhizobium sp. IE4771	rei	9	9	9	5				
Rhodopseudomonas palustris BisB5	rpd	9	9	9	6				
Sphingopyxis granuli	sgi	9	6	9	6				
Mesorhizobium opportunistum	mop	8	8	8	7				
Rhizobium etli bv. *mimosae* Mim1	rel	8	8	8	6				
Jannaschia sp. CCS1	jan	8	8	8	5				
Hyphomonas neptunium	hne	8	6	6	3				
Sinorhizobium americanum	*same*	*7*	*7*	*7*	*6*				
Ensifer adhaerens OV14	ead	7	6	6	8				
Rhizobium etli bv. *phaseoli* IE4803	rep	7	7	7	4				
Rhizobium tropici	rtr	7	7	7					
Rhizobium gallicum	rga	7			6				
Rhizobium sp. N1341	rhn	7	6	6	6				
Rhizobium phaseoli	rpha	7	7	7	5				
Rhizobium sp. N731	rhx	7	7	7	6				

Bradyrhizobium icense	bic	7	7	7	9				
Rhodopseudomonas palustris TIE-1	rpt	7	7	7	6				
Methylobacterium radiotolerans	mrd	7	6	6	9				
Methylobacterium sp. 4-46	met	7	7	7	15				
Methylobacterium aquaticum	maqu	7	6	6	7				
Erythrobacter litoralis DSM 8509	elq	7	7	7	2				
Porphyrobacter sp. LM 6	porl	7	7	7	1				
Mesorhizobium japonicum MAFF 303099	mlo	6	6	6	8	1	hserlactone	1	CYP206A1
Mesorhizobium ciceri (biovar Biserrulae)	mci	6	6	6	6				
Mesorhizobium amorphae	mamo	6	6	6	7				
Rhizobium etli CIAT 652	rec	6	6	6	5				
Rhodopseudomonas palustris CGA009	rpa	6	6	6	7				
Rhodopseudomonas palustris DX-1	rpx	6	6	6	8				
Methylobacterium nodulans	mno	6	6	6	11				
Methylobacterium oryzae	mor	6	4	4	10				
Sphingobium sp. YBL2	syb	6	5	6	5				
Erythrobacter litoralis HTCC2594	eli	6	4	6	1				
Sulfitobacter sp. AM1-D1	suam	6	6	6	6	1	acyl_amino_acids, bacteriocin, NRPS-like	3	CYP1326A2, CYP195A21

									, CYP2334A1
Mesorhizobium australicum	mam	5	5	5	7				
Sinorhizobium meliloti GR4	smeg	5	4	4	4				
Rhizobium etli CFN 42	ret	5	5	5					
Methylorubrum extorquens PA1	mex	5	5	5	7				
Methylorubrum extorquens AM1	mea	5	5	5	7				
Methylorubrum extorquens DM4	mdi	5	5	5	7				
Hyphomicrobium denitrificans 1NES1	hdt	5	5	5	3				
Caulobacter sp. K31	cak	5	4	4	4				
Caulobacter segnis	cse	5	5	5	2				
Celeribacter indicus	cid	5	4	4	6	1	ectoine	1	CYP1101A27
Novosphingobium sp. PP1Y	npp	5	5	5	3				
Novosphingobium pentaromativorans	npn	5	4	5	5				
Sphingopyxis alaskensis	sal	5	5	5	6				
Sphingopyxis fribergensis	sphk	5	4	4	12				
Sphingopyxis sp. 113P3	sphp	5	4	4					
Agrobacterium fabrum	atu	4	4	4	1	1	terpene	1	CYP206A1
Agrobacterium tumefaciens Ach5	atf	4	4	4	1	1	terpene	1	CYP206A4

Agrobacterium vitis	avi	4	4	4	8				
Rhizobium sp. NT-26	rht	4	4	4	6	1	NAGGN	1	CYP107JE1
Bradyrhizobium sp. CCGE-LA001	brc	4	4	4	11				
Rhodopseudomonas palustris BisA53	rpe	4	4	4	6				
Methylorubrum extorquens CM4	mch	4	4	4	7				
Methylorubrum populi	mpo	4	3	3	7				
Hyphomicrobium denitrificans ATCC 51888	hdn	4	4	4	4				
Caulobacter vibrioides CB15	ccr	4	4	4	4				
Caulobacter vibrioides NA1000	ccs	4	4	4	4				
Ruegeria pomeroyi	sil	4	4	4	6				
Leisingera methylohalidivorans	lmd	4	4	4	9				
Marinovum algicola	malg	4	4	4	5				
Confluentimicrobium sp. EMB200-NS6	con	4	4	4	5				
Salipiger profundus	tpro	4	3	3	5				
Pelagibaca abyssi	paby	4	4	4	6				
Sphingomonas sp. MM-1	sphm	4	3	3	4				
Sphingobium chlorophenolicum	sch	4	4	4	6				
Sphingobium sp. RAC03	sphr	4	3	3	5				
Citromicrobium sp. JL477	cij	4	4	4	2				

Altererythrobacter epoxidivorans	aep	4	4	4	1				
Azospirillum sp. B510	azl	4	4	4	2				
Tistrella mobilis	tmo	4	4	4	11				
Hirschia baltica	hba	3	3	3	3				
Mesorhizobium sp. B7	meso	3	3	3	5				
Hoeflea sp. IMCC20628	hoe	3	3	3	5				
Sinorhizobium meliloti SM11	smx	3	3	3	5				
Sinorhizobium fredii USDA 257	sfd	3	3	3	8				
Agrobacterium radiobacter	ara	3	3	3	3				
Rhizobium leguminosarum bv. trifolii WSM2304	rlt	3	3	3	4				
Rhizobium leguminosarum bv. trifolii WSM1689	rlb	3	3	3	5				
Rhizobium sp. IRBG74	rir	3	3	3	1	1	terpene	1	CYP206A3
Rhodopseudomonas palustris BisB18	rpc	3	3	3	8				
Methylobacterium sp. AMS5	meta	3	3	3	7				
Hyphomicrobium sp. MC1	hmc	3	3	3	4				
Candidatus Filomicrobium marinum W	fil	3	3	3	3				
Candidatus Filomicrobium marinum Y	fiy	3	3	3	3				
Rhodoplanes sp. Z2-YC6860	rhz	3	3	3	7				
Phenylobacterium zucineum	pzu	3	3	3	3				

Ruegeria sp. TM1040	sit	3	3	3	5				
Rhodobacter capsulatus	rcp	3	2	2	3				
Roseobacter denitrificans	rde	3	3	3	6				
Roseobacter litoralis	rli	3	3	3	7				
Pseudovibrio sp. FO-BEG1	psf	3	3	3	6				
Phaeobacter inhibens DSM 17395	pga	3	3	3	6				
Phaeobacter inhibens 2.10	pgl	3	3	3	6				
Phaeobacter gallaeciensis DSM 26640	pgd	3	3	3	5				
Phaeobacter porticola	php	3	3	3	4				
Planktomarina temperata	ptp	3	3	3	1				
Labrenzia sp. CP4	lap	3	3	3	7				
Defluviimonas alba	daa	3	3	3	3				
Hyphomonadaceae bacterium UKL13-1	hbc	3	3	3	2	1	terpene	1	CYP1246A4
Sphingobium japonicum	sjp	3	3	3	7				
Altererythrobacter atlanticus	aay	3	3	3	2				
Polymorphum gilvum	pgv	3	3	3	8				
Agrobacterium rhizogenes	aro	3	3	3	3	1	terpene	1	CYP206A2
Neorhizobium galegae bv. *officinalis* bv. officinalis HAMBI 1141	ngl	3	3	3	3				

Paracoccus yeei	pye	3	3	3	5				
Mesorhizobium loti NZP2037	mln	2	2	2	8				
Chelativorans sp. BNC1	mes	2	2	2	4				
Sinorhizobium meliloti 1021	sme	2	2	2	4				
Sinorhizobium meliloti AK83	smk	2	2	2	9				
Sinorhizobium meliloti BL225C	smq	2	2	2	4				
Sinorhizobium meliloti Rm41	smi	2	2	2	4				
Sinorhizobium meliloti 2011	smel	2	2	2	4				
Sinorhizobium meliloti RMO17	smer	2	2	2	4				
Sinorhizobium medicae	smd	2	2	2	5				
Sinorhizobium fredii HH103	sfh	2	2	2	7				
Sinorhizobium sp. RAC02	six	2	2	2	1				
Ensifer adhaerens Casida A	eah	2	2	2					
Agrobacterium sp. H13-3	agr	2	2	2	1	1	terpene	1	CYP206A4
Agrobacterium sp. RAC06	agc	2	2	2	8				
Rhizobium leguminosarum bv. viciae 3841	rle	2	2	2	5				
Rhizobium leguminosarum bv. trifolii WSM1325	rlg	2	2	2	5				
Rhizobium leguminosarum bv. trifolii CB782	rlu	2	2	2	4				
Neorhizobium galegae bv. *orientalis* HAMBI 540	ngg	2	2	2	2				

Bosea sp. RAC05	bos	2	2	2	3				
Bosea vaviloviae	bvv	2	2	2	6	1	T1PKS	1	CYP1101A30
Azorhizobium caulinodans	azc	2	2	2	6				
Beijerinckia indica	bid	2	2	2	10	1	NRPS,T1PKS	1	CYP173J1
Methylocella silvestris	msl	2	2	2	12				
Hyphomicrobium nitrativorans	hni	2	2	2	4				
Methylocystis sp. SC2	msc	2	2	2	13				
Methyloceanibacter caenitepidi	mcg	2	2	2	6				
Rhodobacter sphaeroides 2.4.1	rsp	2	2	2	5				
Rhodobacter sphaeroides ATCC 17029	rsh	2	2	2	6				
Rhodobacter sphaeroides ATCC 17025	rsq	2	2	2	5	1	hserlactone	1	CYP152C2
Paracoccus denitrificans	pde	2	2	2	7				
Paracoccus aminophilus	pami	2	2	2	7				
Dinoroseobacter shibae	dsh	2	2	2					
Ketogulonicigenium robustum	kro	2	2	2	3				
Octadecabacter arcticus	oar	2	2	2	5				
Octadecabacter temperatus	otm	2	2	2	2				
Roseibacterium elongatum	red	2	2	2	7				

Celeribacter marinus	cmar	2	2	2	2				
Rhodovulum sp. P5	rhc	2	2	2	7				
Labrenzia aggregata	lagg	2	2	2	7				
Tateyamaria omphalii	tom	2	2	2	5				
Thioclava nitratireducens	thw	2	2	2	5				
Roseovarius mucosus	rmm	2	2	2	8				
Yoonia vestfoldensis	lvs	2	2	2	5				
Maricaulis maris	mmr	2	2	2	1				
Sphingobium sp. EP60837 Chromosome 1	sphb	2	2	2	3				
Altererythrobacter marensis	amx	2	2	2	2				
Altererythrobacter dongtanensis	ado	2	2	2	4				
Porphyrobacter neustonensis	pns	2	2	2	2				
Gluconacetobacter diazotrophicus PA1 5 (Brazil)	gdi	2	2	2	5				
Gluconacetobacter diazotrophicus PA1 5 (JGI)	gdj	2	2	2	4				
Azospirillum brasilense Sp245	abs	2	2	2	4				
Magnetospira sp. QH-2	magq	2	2	2					
Sphingobium baderi	sbd	2	2	2					
Aminobacter aminovorans	aak	1	1	1	5				
Agrobacterium tumefaciens S33	ata	1	1	1	3				

Rhizobium favelukesii	rhl	1	1	1	3					
Liberibacter crescens	lcc	1	1	1						
Shinella sp. HZN7	shz	1	1	1	2					
Brucella melitensis bv. 1 16M	bme	1	1	1	4					
Brucella melitensis bv. 1 16M	bmel	1	1	1	4					
Brucella melitensis ATCC 23457	bmi	1	1	1	3					
Brucella melitensis M28	bmz	1	1	1	4					
Brucella melitensis M5-90	bmg	1	1	1	3					
Brucella melitensis NI	bmw	1	1	1	4					
Brucella melitensis bv. 3 Ether	bmee	1	1	1	3					
Brucella abortus 2308	bmf	1	1	1	4					
Brucella abortus S19	bmc	1	1	1	5					
Brucella abortus A13334	baa	1	1	1	5					
Brucella abortus bv. 2 86/8/59	babo	1	1	1	5					
Brucella abortus bv. 6 870	babr	1	1	1	5					
Brucella abortus 63 75	babt	1	1	1	6					
Brucella abortus BDW	babb	1	1	1	5					
Brucella abortus bv. 9 C68	babu	1	1	1	5					
Brucella abortus BER	babs	1	1	1	5					

Brucella abortus NCTC 10505	babc	1	1	1	5				
Brucella suis bv. 2 Bs143CITA	bsui	1	1	1	5				
Brucella suis bv. 2 PT09143	bsup	1	1	1	5				
Brucella suis bv. 2 PT09172	bsuv	1	1	1	5				
Brucella suis bv. 2 Bs364CITA	bsuc	1	1	1	5				
Brucella suis bv. 3	bsz	1	1	1	5				
Brucella suis ZW043	bsw	1	1	1	6				
Brucella suis ZW046	bsg	1	1	1	6				
Brucella canis ATCC 23365	bcs	1	1	1	4				
Brucella canis HSK A52141	bsk	1	1	1	5				
Brucella canis RM6/66	bcar	1	1	1	5				
Brucella canis SVA13	bcas	1	1	1	5				
Brucella pinnipedialis 6/566	bpv	1	1	1	5				
Brucella ceti TE10759-12	bcet	1	1	1	5				
Ochrobactrum anthropi ATCC 49188	oan	1	1	1	7				
Ochrobactrum anthropi OAB	oah	1	1	1	7				
Ochrobactrum pseudogrignonense	ops	1	1	1	6				
Nitrobacter winogradskyi	nwi	1	1	1	4				
Nitrobacter hamburgensis	nha	1	1	1	3				

Xanthobacter autotrophicus	xau	1	1	1	9	1	T1PKS	1	CYP1138B1
Pelagibacterium halotolerans	phl	1	1	1	4				
Devosia sp. H5989	deq	1	1	1	2				
Methylocystis bryophila	mbry	1	1	1	9				
Martelella endophytica	mey	1	1	1	4				
Pseudorhodoplanes sinuspersici	psin	1	1	1	9	1	T1PKS	1	CYP1104E2
Asticcacaulis excentricus	aex	1	1	1	5				
Rhodobacter sphaeroides KD131	rsk	1	1	1	5				
Ketogulonicigenium vulgare Y25	kvu	1	1	1	3				
Ketogulonicigenium vulgare WSH-001	kvl	1	1	1	3				
Rhodovulum sulfidophilum	rsu	1	1	1					
Pannonibacter phragmitetus	pphr	1	1	1	9				
Yangia sp. CCB-MM3	yan	1	1	1	5				
Sphingopyxis terrae	ster	1	1	1	6				
Sphingopyxis sp. LPB0140	sphl	1	1	1	2				
Sphingomonas sp. WHSC-8	sphi	1	1	1					
Sphingobium sp. SYK-6	ssy	1	1	1	7				
Sphingobium indicum	sinb	1	1	1	2				
Sphingorhabdus sp. M41	sphg	1	1	1	3				

Altererythrobacter namhicola	anh	1	1	1	2				
Croceicoccus naphthovorans	cna	1	1	1					
Acetobacter aceti	aace	1	1		2				
Magnetospirillum gryphiswaldense MSR-1 v2	mgy	1	1	1					
Azospirillum brasilense Az39	abq	1	1	1	3				
Candidatus Puniceispirillum marinum	apb	1	1	1	2				
Rickettsiales bacterium Ac37b	rbt				1				
Rickettsiales endosymbiont of *Stachyamoeba lipophora*	ren				1				
Candidatus Paracaedibacter acanthamoebae	paca				2				
Candidatus Paracaedimonas acanthamoebae	caq				1				
Candidatus Nucleicultrix amoebiphila	naf				2				
Endosymbiont of *Acanthamoeba* sp. UWC8	eaa				1				
Mesorhizobium sp. WSM1497	mesw				7				
Aminobacter sp. MSH1	amih				5				
Phyllobacterium zundukense	pht				2				
Roseitalea porphyridii	rpod				7				
Rhodobiaceae bacterium SMS8	rbs				2				
Sinorhizobium sp. CCBAU 05631	sino				7				
Ensifer sojae	esj				5				

Rhizobium sp. S41	rhv				1				
Rhizobium sp. Kim5	rhk				4				
Rhizobium esperanzae	rez				7				
Neorhizobium sp. SOG26	neo				3				
Neorhizobium sp. NCHU2750	nen				3				
Candidatus Liberibacter asiaticus psy62	las				2				
Candidatus Liberibacter asiaticus gxpsy	laa				2				
Candidatus Liberibacter asiaticus Ishi-1	lat				2				
Candidatus Liberibacter solanacearum	lso				2				
Candidatus Liberibacter americanus	lar				2				
Candidatus Liberibacter africanus	lau				2				
Brucella abortus bv. 1 9-941	bmb				2				
Brucella suis 1330	bms				2				
Brucella suis 1330	bsi				2				
Brucella suis bv. 1	bsf				2				
Brucella suis ATCC 23445	bmt				2				
Brucella suis VBI22	bsv				2				
Brucella ovis	bov				3				
Brucella canis Oliveri	bol				2				

Brucella microti	bmr				3				
Brucella pinnipedialis B2/94	bpp				3				
Brucella ceti TE28753-12	bcee				3				
Brucella vulpis	bvl				3				
Brucella sp. 2002734562	bru				2				
Ochrobactrum sp. A44	och				4				
Bradyrhizobium sp. ORS 285	bro				12				
Bradyrhizobium sp. SK17	brk				12				
Bradyrhizobium ottawaense	bot				7				
Bradyrhizobium sp. 2 39S1MB	brq				7				
Oligotropha carboxidovorans OM5 (Mississippi)	oca				4				
Oligotropha carboxidovorans OM5 (Goettingen)	ocg				3				
Oligotropha carboxidovorans OM4	oco				3				
Bosea sp. PAMC 26642	bop				2				
Bosea sp. Tri-49	boi				5				
Variibacter gotjawalensis	vgo				4				
Bartonella henselae Houston-1	bhe				1				
Bartonella henselae BM1374163	bhn				1				
Bartonella henselae BM1374165	bhs				1				

Bartonella quintana Toulouse	bqu				2				
Bartonella quintana RM-11	bqr				2				
Bartonella bacilliformis	bbk				1				
Bartonella tribocorum CIP 105476	btr				2				
Bartonella tribocorum BM1374166	btx				2				
Bartonella grahamii	bgr				2				
Bartonella clarridgeiae	bcd				2				
Bartonella australis	baus				2				
Bartonella vinsonii	bvn				2				
Bartonella ancashensis	banc				2				
Bartonella apis	bapi				2				
Bartonella sp. JB15	bart				1				
Bartonella sp. A1379B	bara				2				
Bartonella sp. WD16.2	barw				1				
Bartonella sp. Raccoon60	barr				2				
Bartonella sp. 1-1C	baro				2				
Bartonella sp. JB63	barj				1				
Starkeya novella	sno				4				
Methylorubrum zatmanii	mza				7				

Methylobacterium phyllosphaerae	mphy				10				
Methylobacterium currus	mee				7				
Methylobacterium sp. DM1	metd				8				
Methylobacterium sp. XJLW	metx				9				
Methylobacterium sp. 17SD2-17	mets				6				
Microvirga ossetica	moc				4				
Microvirga sp. 17 mud 1-3	miv				7				
Methylovirgula ligni	mlg				6				
Chelatococcus sp. CO-6	chel				6				
Chelatococcus daeguensis	cdq				5				
Rhodomicrobium vannielii	rva				7				
Devosia sp. I507	dei				6				
Blastochloris viridis	bvr				6				
Blastochloris sp. GI	blag				3				
Maritalea myrionectae	mmyr				4				
Methylocystis rosea	mros				14				
Methylosinus trichosporium	mtw				9				
Pleomorphomonas sp. SM30	pleo				4				
Martelella sp. AD-3	maad				5				

Martelella mediterranea	mmed				5				
Aureimonas sp. AU20	aua				4				
Breoghania sp. L-A4	brn				7				
Candidatus Tokpelaia hoelldoblerii	thd				3				
Hartmannibacter diazotrophicus	hdi				8				
Rhizobiales bacterium NRL2	rbm				8				
Caulobacter henricii	chq				4				
Caulobacter mirabilis	cmb				5				
Caulobacter flavus	cfh				5				
Phenylobacterium sp. HYN0004	phb				1				
Brevundimonas subvibrioides	bsb				3				
Brevundimonas sp. DS20	brd				5				
Brevundimonas naejangsanensis	bne				3				
Brevundimonas sp. GW460-12-10-14-LB2	brg				2				
Brevundimonas sp. LM2	brl				5				
Brevundimonas vesicularis	bvc				2				
Brevundimonas diminuta	bdm				7				
Caulobacteraceae bacterium OTSz_A_272	cbot				3				
Ruegeria sp. AD91A	rua				7				

Epibacterium mobile	rmb				5					
Rhodobacter sp. LPB0142	rhp				4					
Rhodobacter blasticus	rbl				3					
Paracoccus contaminans	pcon				3					
Paracoccus zhejiangensis	pzh				6					
Paracoccus sp. BM15	paro				8					
Paracoccus sp. CBA4604	paru				4					
Paracoccus aminovorans	pamn				5					
Paracoccus mutanolyticus	pmut				2					
Paracoccus sp. SC2-6	pars				3					
Phaeobacter piscinae	ppic				5					
Phaeobacter sp. LSS9	phq				7					
Octadecabacter antarcticus	oat				8					
Leisingera sp. NJS204	lej				8					
Celeribacter ethanolicus	ceh				4					
Celeribacter manganoxidans	cmag				3					
Rhodovulum sp. MB263	rhm				5					
Halocynthiibacter arcticus	hat				5					
Labrenzia sp. VG12	labr				6					

Yangia pacifica	ypac				3				
Sulfitobacter pseudonitzschiae	spse				9				
Sulfitobacter sp. SK012	sulz				8				
Sulfitobacter sp. JL08	suli				5				
Sulfitobacter sp. D7	suld				5				
Donghicola sp. JLT3646	don				5				
Roseovarius sp. AK1035	rok				8				
Antarctobacter heliothermus	aht				9				
Rhodobaca barguzinensis	rbg				3				
Sagittula sp. P11	sagu				3				
Thalassococcus sp. SH-1	thas				3				
Thalassococcus sp. S3	thaa				9				
Gemmobacter sp. HYN0069	geh				6				
Tabrizicola sp. K13M18	taw				5				
Silicimonas algicola	salo				6				
Sedimentitalea sp. W43	sedi				5				
Haematobacter massiliensis	hml				3				
Rhodobacteraceae bacterium G7	rbz				5				
Zymomonas mobilis subsp. mobilis ZM4	zmo				2				

Zymomonas mobilis subsp. *mobilis* NCIMB 11163	zmn				2				
Zymomonas mobilis subsp. *mobilis* ATCC 10988	zmm				2				
Zymomonas mobilis subsp. *mobilis* ATCC 29191	zmb				2				
Zymomonas mobilis subsp. *mobilis* CP4 = NRRL B-14023	zmi				2				
Zymomonas mobilis subsp. *mobilis* CP4 = NRRL B-14023	zmc				2				
Zymomonas mobilis subsp. *mobilis* NRRL B-12526	zmr				2				
Zymomonas mobilis subsp. *pomaceae* ATCC 29192	zmp				1				
Novosphingobium resinovorum	nre				4				
Novosphingobium sp. P6W	nov				4				
Novosphingobium sp. THN1	not				2				
Novosphingobium tardaugens	ntd				1				
Sphingopyxis macrogoltabida EY-1	smag				8				
Sphingopyxis sp. QXT-31	sphq				8				
Sphingopyxis sp. MG	spho				4				
Uncultured Sphingopyxis sp. UC10	sphu				6				
Sphingomonas wittichii DC-6	sphd				6				
Sphingomonas taxi	stax				3				
Sphingomonas sanxanigenens	ssan				5				
Sphingomonas sp. NIC1	snj				3				

Sphingomonas melonis	smy				5				
Sphingomonas panacis	span				4				
Sphingomonas koreensis	skr				6				
Sphingomonas sp. LM7	splm				1				
Sphingomonas sp. LK11	splk				3				
Sphingomonas sp. JJ-A5	sphj				4				
Sphingomonas sp. KC8	spkc				5				
Sphingomonas sp. Cra20	sphc				3				
Sphingomonas sp. FARSPH	sphf				4				
Sphingomonas sp. YZ-8	spha				4				
Sphingobium sp. TKS	spht				4				
Sphingobium hydrophobicum	shyd				4				
Sphingobium yanoikuyae	sya				10				
Sphingobium cloacae	sclo				1				
Sphingobium sp. YG1	spyg				4				
Sphingorhabdus flavimaris	sfla				5				
Sphingorhabdus sp. YGSMI21	sphy				4				
Blastomonas fulva	bfw				5				
Rhizorhabdus dicambivorans	rdi				7				

Sphingosinicella microcystinivorans	smic				9				
Sphingosinicella sp. BN140058	sphs				6				
Erythrobacter sp. s21-N3	ery				4				
Erythrobacter gangjinensis	egn				3				
Erythrobacter flavus	efv				2				
Erythrobacter sp. KY5	erk				3				
Erythrobacter sp. YH-07	err				4				
Altererythrobacter sp. B11	alb				4				
Croceicoccus marinus	cman				4				
Porphyrobacter sp. HT-58-2	phz				2				
Gluconobacter oxydans 621H	gox				2				
Gluconobacter oxydans H24	goh				4				
Gluconobacter oxydans DSM 3504	goy				1				
Gluconobacter albidus	gal				1				
Granulibacter bethesdensis CGDNIH1	gbe				6				
Granulibacter bethesdensis CGDNIH2	gbh				6				
Granulibacter bethesdensis CGDNIH3	gbc				6				
Granulibacter bethesdensis CGDNIH4	gbs				6				
Acidiphilium cryptum	acr				4				

Acidiphilium multivorum	amv				6					
Komagataeibacter medellinensis	gxy				3					
Komagataeibacter xylinus	gxl				3					
Komagataeibacter nataicola	kna				6					
Komagataeibacter europaeus	keu				6					
Komagataeibacter saccharivorans	ksc				4					
Acetobacter pasteurianus IFO 3283-01	apt				3					
Acetobacter pasteurianus IFO 3283-01-42C	apw				3					
Acetobacter pasteurianus IFO 3283-03	apf				3					
Acetobacter pasteurianus IFO 3283-07	apu				3					
Acetobacter pasteurianus IFO 3283-12	apg				3					
Acetobacter pasteurianus IFO 3283-22	apq				3					
Acetobacter pasteurianus IFO 3283-26	apx				3					
Acetobacter pasteurianus IFO 3283-32	apz				3					
Acetobacter pasteurianus 386B	apk				3					
Acetobacter senegalensis	asz				4					
Acetobacter oryzifermentans	asv				4					
Acetobacter persici	aper				7					
Acetobacter pomorum	apom				4					

Acetobacter tropicalis	ato				4				
Acetobacter ascendens	aasc				3				
Acetobacter sp. JWB	acet				4				
Asaia bogorensis	abg				2				
Kozakia baliensis	kba				3				
Roseomonas gilardii	rgi				6				
Roseomonas sp. FDAARGOS_362	ros				6				
Neoasaia chiangmaiensis	nch				4				
Commensalibacter sp. AMU001	coq				2				
Rhodospirillum rubrum ATCC 11170	rru				9				
Rhodospirillum rubrum F11	rrf				11				
Rhodospirillum centenum	rce				5				
Pararhodospirillum photometricum	rpm				3				
Magnetospirillum magneticum	mag				4				
Magnetospirillum gryphiswaldense MSR-1	mgry				4				
Magnetospirillum sp. XM-1	magx				6				
Magnetospirillum sp. ME-1	magn				4				
Azospirillum lipoferum	ali				2				
Azospirillum brasilense Sp7	abf				3				

Azospirillum thiophilum	ati				2				
Azospirillum humicireducens	ahu				2				
Azospirillum sp. TSH58	azt				2				
Azospirillum sp. M2T2B2	azm				2				
Candidatus Endolissoclinum faulkneri L2	thal				4				
Candidatus Endolissoclinum faulkneri L5	efk				4				
Thalassospira xiamenensis	txi				6				
Thalassospira marina	thac				5				
Thalassospira indica	tii				5				
Haematospirillum jordaniae	hjo				2				
Nitrospirillum amazonense	nao				2				
Niveispirillum cyanobacteriorum	ncb				3				
Parvularcula bermudensis	pbr				4				
Magnetococcus marinus	mgm				2				
Candidatus Pelagibacter ubique	pub				1				
Candidatus Pelagibacter sp. IMCC9063	pel				1				
Alpha proteobacterium HIMB59	apc				1				
Alpha proteobacterium HIMB5	apm				1				
Micavibrio aeruginosavorus ARL-13	mai				3				

Micavibrio aeruginosavorus EPB	man				2				
Phreatobacter sp. S-12	phr				2				
Alphaproteobacteria bacterium WS11	abaw				5				

Table S3: List of new P450 families identified in Alphaproteobacterial species.

CYP1010A1

CYP1017A1
CYP103A1
CYP104A1
CYP1075A1
CYP1076A1
CYP1077A1
CYP1078A1
CYP112A1
CYP1135A1
CYP1137A1
CYP1141A1
CYP1145A1
CYP1170A1
CYP1171A1
CYP1173A1
CYP1174A1
CYP1175A1
CYP1181A1
CYP1182A1
CYP1186A1
CYP1187A1
CYP1258A1
CYP127A1
CYP1281A1
CYP1302A1
CYP1311A1
CYP1312A1
CYP1384A1
CYP1396A1

CYP1515A1
CYP152AA1
CYP1732A1
CYP1733A1
CYP1734A1
CYP1735A1
CYP1736A1
CYP1737A1
CYP1738A1
CYP1739A1
CYP173A1
CYP1740A1
CYP1741A1
CYP1742A1
CYP1743A1
CYP1744A1
CYP1745A1
CYP1746A1
CYP1747A1
CYP1748A1
CYP1749A1
CYP1750A1
CYP1751A1
CYP1752A1
CYP1753A1
CYP1754A1
CYP1755A1
CYP192A1
CYP193A1

CYP194A1
CYP195A1
CYP196A1
CYP199A1
CYP200A1
CYP201A1
CYP202A1
CYP203A1
CYP204A1
CYP206A1
CYP2140A1
CYP219A1
CYP223A1
CYP224A1
CYP225A1
CYP2334A1
CYP289A1
CYP290A1
